# Construction of a radiation hybrid panel and the first yellowtail (*Seriola quinqueradiata*) radiation hybrid map using a nanofluidic dynamic array

**DOI:** 10.1186/1471-2164-15-165

**Published:** 2014-02-27

**Authors:** Jun-ya Aoki, Wataru Kai, Yumi Kawabata, Akiyuki Ozaki, Kazunori Yoshida, Tatsuo Tsuzaki, Kanako Fuji, Takashi Koyama, Takashi Sakamoto, Kazuo Araki

**Affiliations:** 1 National Research Institute of Aquaculture, Fisheries Research Agency, 224-1 Hiruta, Tamaki-cho, Watarai-gun, Mie 519-0423, Japan; 2 National Research Institute of Aquaculture, Fisheries Research Agency, 422-1 Nakatsuhamaura, Minamiise-cho, Watarai-gun, Mie 516-0193, Japan; 3 Goto Laboratory, Seikai National Fisheries Research Institute, Fisheries Research Agency, 122-7, Nunoura, Tamanoura-cho, Goto, Nagasaki 853-0508, Japan; 4 Faculty of Marine Science, Tokyo University of Marine Science and Technology, 4-5-7, Konan, Minato-ku, Tokyo 108-8477, Japan

**Keywords:** Yellowtail, Radiation hybrid (RH) panel, RH map, Synteny

## Abstract

**Background:**

Yellowtail (*Seriola quinqueradiata*) are an economically important species in Japan. However, there are currently no methods for captive breeding and early rearing for yellowtail. Thus, the commercial cultivation of this species is reliant upon the capture of wild immature fish. Given this, there is a need to develop captive breeding techniques to reduce pressure on wild stocks and facilitate the sustainable development of yellowtail aquaculture. We constructed a whole genome radiation hybrid (RH) panel for yellowtail gene mapping and developed a framework physical map using a nanofluidic dynamic array to use SNPs (single nucleotide polymorphisms) in ESTs (expressed sequence tags) for the DNA-assisted breeding of yellowtail.

**Results:**

Clonal RH cell lines were obtained after ionizing radiation; specifically, 78, 64, 129, 55, 42, and 53 clones were isolated after treatment with 3,000, 4,000, 5,000, 6,000, 8,000, or 10,000 rads, respectively. A total of 421 hybrid cell lines were obtained by fusion with mouse B78 cells. Ninety-four microsatellite markers used in the genetic linkage map were genotyped using the 421 hybrid cell lines. Based upon marker retention and genome coverage, we selected 93 hybrid cell lines to form an RH panel. Importantly, we performed the first genotyping of yellowtail markers in an RH panel using a nanofluidic dynamic array (Fluidigm, CA, USA). Then, 580 markers containing ESTs and SNPs were mapped in the first yellowtail RH map.

**Conclusions:**

We successfully developed a yellowtail RH panel to facilitate the localization of markers. Using this, a framework RH map was constructed with 580 markers. This high-density physical map will serve as a useful tool for the identification of genes related to important breeding traits using genetic structural information, such as conserved synteny. Moreover, in a comparison of 30 sequences in the RH group 1 (SQ1), yellowtail appeared to be evolutionarily closer to medaka and the green-spotted pufferfish than to zebrafish. We suggest that synteny analysis may be potentially useful as a tool to investigate chromosomal evolution by comparison with model fish.

## Background

The genus *Seriola,* which includes yellowtail (*Seriola quinqueradiata*), amberjack (*Seriola dumerili*), and kingfish (*Seriola lalandi*), accounted for >60% of the total Japanese mariculture production in 2011 [1]. Yellowtail, in particular, are very popular with Japanese consumers. At present, culturists rely on the capture of wild juveniles to provide the seed as commercial scale methods for captive reproduction and early rearing have yet to be developed. Thus, there is a clear need to develop methods for breeding to preserve wild stocks and facilitate the selection of economically important traits. The latter requires genomic information such as the chromosomal locations of genetic polymorphisms and expressed genes. Currently, there are a limited number of yellowtail families available for linkage analysis. Even though a female linkage map with 180 microsatellite markers has been reported [2], there remains a need for maps of expressed sequence tags (ESTs) and cDNAs to inform yellowtail genomic structure. Recently, linkage maps were developed for several, commonly cultured species, including Atlantic salmon [3], Atlantic cod [4], common carp [5], and catfish [6]. These maps were created with microsatellite markers and gene-associated single nucleotide polymorphisms (SNPs) derived from ESTs. SNPs, the most abundant type of DNA sequence polymorphism, are suitable for high-throughput genotyping and provide enhanced possibilities for genetic and breeding applications, linkage map development, assessment of genetic variability, and marker-assisted breeding. Furthermore, SNP correlation analysis can be used to facilitate selective breeding for desirable traits. To place marker DNA sequences on a linkage map, there must be polymorphisms within the families that are used for the linkage analysis. ESTs sometimes have SNPs, but many ESTs lack SNPs in linkage analysis families obtained by mating one male and one female. To address this, we physically mapped a large number of ESTs using radiation hybrid (RH) cell lines.

An RH map is a powerful tool for building a physical map of the whole genome. This technique can be used to determine the order and distance between DNA markers by analyzing their presence or absence in hybrid cells, and to localize both polymorphic markers and non-polymorphic markers, such as ESTs. Furthermore, RH maps serve as a valuable tool for the molecular identification of mutations using a candidate gene approach, and can be used to facilitate the construction of gene orthology relationships through conserved synteny analysis. In mammals, RH maps have been reported for humans [7,8], mice [9], dogs [10], cats [11], horses [12], and pigs [13]. In addition, six RH panels have been published for five teleost fish species: two zebrafish RH panels were derived from the fibroblast cell line AB9 [14,15] one gilthead seabream RH panel was derived from primary fin fibroblasts [16], a medaka panel was derived from a fin fibroblast cell line [17], a European seabass panel from splenocytes [18], and a Nile tilapia panel from a homozygous clonal line [19]. In the current study, we developed the first RH panel derived from a yellowtail fin fibroblast cell line.

We used a nanofluidic dynamic array (Fluidigm, USA) high-throughput genotyping system. This system was originally developed for SNP genotyping assays and gene expression analyses, and is able to perform 9,216 real-time polymerase chain reactions (PCRs) (96 primers × 96 samples) on a single chip. We adapted this system for use as an expression assay platform to map EST and SNP sites as DNA markers.

Here, we report on the production of a yellowtail fin radiation hybrid cell line and the construction of a 580-marker map that includes 97 microsatellite markers.

## Results and discussion

### Visual analysis of chromosomes in the fibroblast cell line

**Figure 1:**
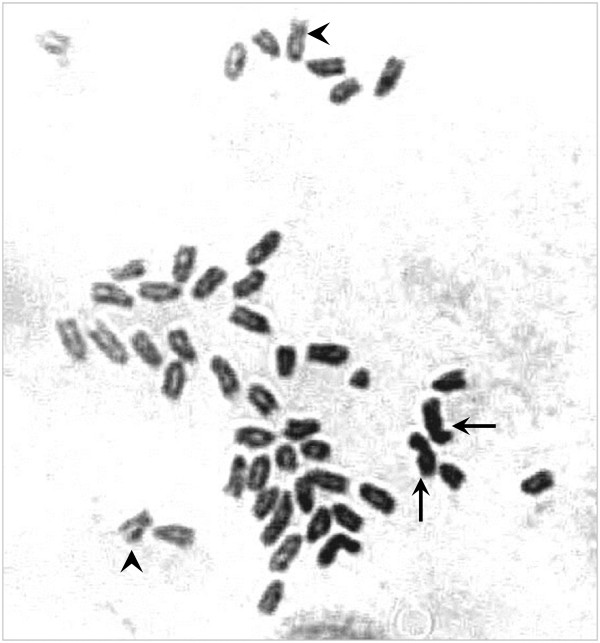
**Yellowtail metaphase chromosomes (*Seriola quinqueradiata*).** Arrows indicate submetacentrics and arrowheads indicate subtelocentrics, while other chromosomes are acrocentric with respect to chromosomal karyotype.

A photomicrograph of mitotic metaphase chromosomes from a yellowtail fibroblast cell is shown in Figure 1. The modal number of chromosomes was 48. This number is consistent with a previous report that noted the diploid chromosome number in yellowtail gill cells was 48 with a karyotype consisting of one pair of submetacentrics, one pair of subtelocentrics, and 22 pairs of acrocentrics [20]. Therefore, we divided the RH map into 24 linkage groups.

### Construction of an RH panel

A fibroblast cell line with an integrated Geneticin® resistance gene was irradiated with 3,000, 4,000, 5,000, 6,000, 8,000, or 10,000 rads (1 rad = 0.01 Gy) to induce chromosome breakage. Hybrid clones maintaining yellowtail genome fragments were selected based on their growth in DMEM medium containing 1 mg/mL Geneticin®. We were able to obtain hybrid clones at all radiation doses. Specifically, we recovered 78, 64, 129, 55, 42, and 53 clones from those exposed to 3,000, 4,000, 5,000, 6,000, 8,000, or 10,000 rads, respectively. In total, 421 hybrid cell lines were obtained by fusion with mouse B78 cells. Those numbers were similar to what has previously been obtained using human (220) [7], zebrafish (357) [15], gilthead seabream (170) [16], medaka (290) [17], European seabass (290) [18], and Nile tilapia cells (381) [19]. Thus, we felt confident that the number of yellowtail hybrid cell lines we obtained would be a sufficient starting point from which to select 93 hybrid clones for the RH panel.

**Figure 2:**
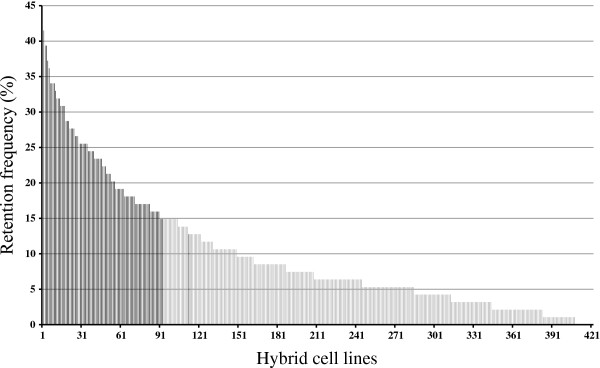
**Retention frequencies of the yellowtail hybrid cell lines. Hybrid cell lines are numbered from 1 to 421 on the *x*-axis.** Their retention frequencies, expressed as the percentage of microsatellite markers per cell line, are represented on the *y*-axis.

The retention frequency was estimated for each clone by genotyping a set of 94 microsatellite markers that were selected from the linkage map [2, unpublished observations], and by scoring them as present, absent, or ambiguous. The retention frequency of the 94 microsatellite markers in the 421 hybrid cell lines is shown in Figure 2. The average retention frequencies at each radiation dose were approximately 10.2, 10.2, 10.9, 7.4, 8.2, and 9.7%, respectively. The average retention frequency of the 421 hybrid cell lines was approximately 9.8%. These values were not correlated with the radiation dose. However, we did expect the size of the chromosome fragments retained within the hybrid cell lines to differ depending on the intensity of radiation received by the cells.

A set of 93 hybrid cell lines was selected to form the RH panel, including 20 lines from the 3,000-rad dose, 19 from the 4,000-rad dose, 35 from the 5,000-rad dose, 6 from the 6,000-rad dose, 5 from the 8,000-rad dose, and 8 from the 10,000-rad dose. In general, the hybrid cell lines were selected based on high-retention frequency values; however, in one case a cell line was substituted to obtain a better representation of the whole genome. The retention frequencies of each clone in the RH panel were in the range of 12.8–41.5%, and averaged 23.2%. For comparison, the average retention frequencies for the RH panel clones in previous studies were 22% in zebrafish [15], 30% in gilthead seabream [16], and 30.6% in European seabass [18]. Thus, by analogy to those studies, the yellowtail RH panel was an adequate starting point for the construction of a physical map.

### Construction of an RH map

**Figure 3:**
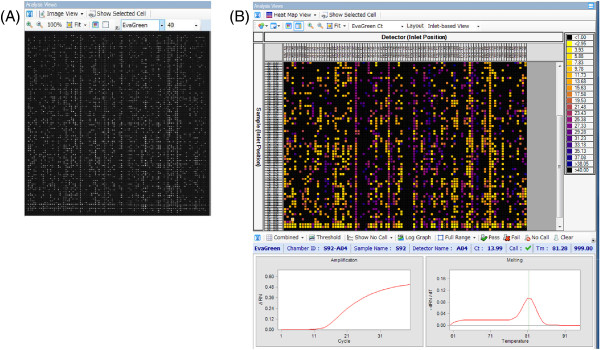
**Images of results in the BioMarkTM HD system. (A)** Image view of the raw fluorescence for a dynamic array. Each white point indicates one reaction as represented by the fluorescence concentration. **(B)** A heat map view converted from the Image view.

The genomic DNA that was extracted from the RH panel was pre-amplified with 96 pooled primer pairs. The pre-amplified DNA was used as the template for the second PCR with each nested primer pair for each of the 96 markers using the BioMark™ HD system (Fluidigm, USA). The BioMark™ HD system is able to perform high-throughput Tm shift reactions, SNP analysis, and gene expression analysis. Therefore, we took advantage of this system to conduct marker genotyping of the physical map using a 96.96 nanofluidic dynamic array. The results of the reaction were estimated from the Ct (threshold cycle) values, the melting temperature of amplification products, and the amplification curves of the PCR products (Figure 3). We then confirmed the existence of the amplification products using these three analytical measurements. The proportion of useful primer pairs among the 96 markers was approximately 25–50%, owing to the sensitivity and specificity of the system. We did not detect any non-specific amplification products. The BioMark™ HD system provided results from ~24–50 primer pairs in 6 h, and clearly had higher throughput relative to electrophoresis. Moreover, the reproducibility of the dynamic array data for replicates within a chip and between different chips is very good [20]. By performing PCR on a dynamic array, one can obtain high-throughput gene expression data that are essentially identical in quality to conventional microliter reverse-transcription-quantitative PCR and are of superior quality to publicly available array data from the same tissue type [21].

**Figure 4:**
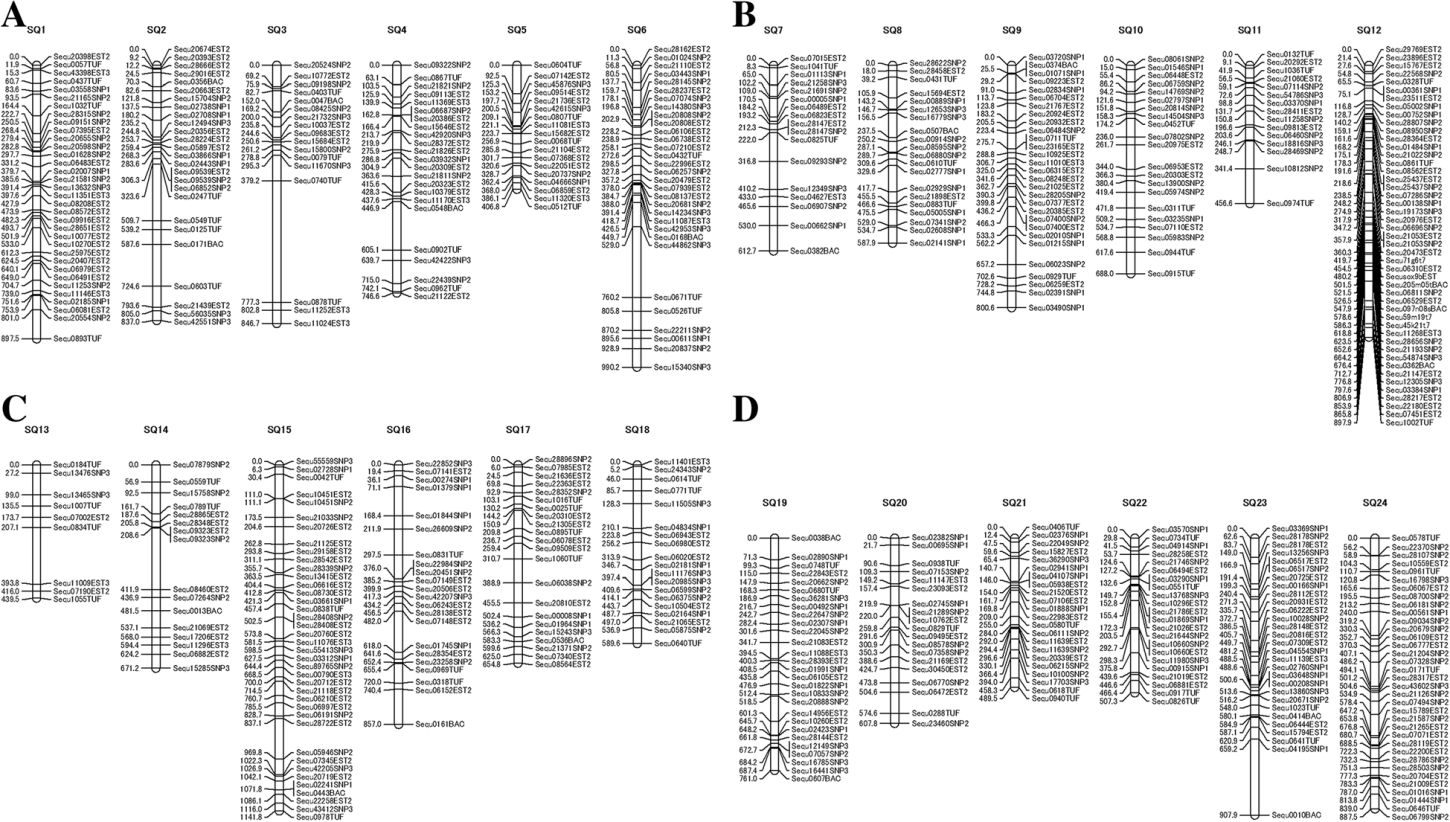
**An RH map of the yellowtail genome (A: SQ1–6, B: SQ7–12, C: SQ13–18, D: SQ19–24).** The RH group is symbolized by a vertical bar. The position of each marker is symbolized by a horizontal bar. Distances between markers are expressed in centi Rays (cR).

We genotyped 631 markers to construct the RH map. The two-point analysis, performed at an LOD score of 4.0 and a distance threshold of 45, yielded 158 groups using CarthaGene software [22,23]. Furthermore, by reference to the locations of several markers on the previously constructed genetic linkage map for yellowtail, 580 markers were assigned to 24 linkage groups. Fifty-one markers in 32 groups were not distributed among the genetic linkage groups because of the LOD score or the distance threshold error. Thus, the current yellowtail physical map was constructed with a final set of 580 markers consisting of 483 genes and 97 microsatellite markers that were genotyped by PCR and electrophoresis (Table 1, Figure 4A–D). For individual groups, the RH map had a size range of 406.8–1141.8 cR (1 centi Ray, cR = 1% frequency of a breakage occurring between two markers after exposure to a radiation dose) with an average of 720.1 cR. The combined size of all RH groups was 17283.4 cR. The yellowtail genome is estimated to be 800 Mbp [2], which yields a value of 1 cR = 46.3 kbp (800 Mbp/17283.4 cR).

**Table 1:** Characteristics of the yellowtail RH map

**Group**	**Size (cR)**	**No. of markers**		
		**Genes**	**Microsatellite**	**Total**
1	897.5	30	4	34
2	837.0	20	6	26
3	846.7	12	5	17
4	746.6	19	4	23
5	406.8	14	4	18
6	990.2	27	4	31
7	612.7	14	3	17
8	587.9	16	4	20
9	800.6	26	3	29
10	688.0	17	4	21
11	456.6	12	3	15
12	897.9	40	7	47
13	439.5	5	4	9
14	671.2	13	3	16
15	1141.8	35	4	39
16	857.0	18	4	22
17	654.8	17	5	22
18	589.6	16	3	19
19	761.0	24	4	28
20	607.8	16	3	19
21	489.5	18	4	22
22	507.3	18	4	22
23	907.9	25	4	29
24	887.5	31	4	35
Subtotal	17283.4	483	97	580
Unlinked		51		51
Total	17283.4	534		631

We were able to quickly obtain a large quantity of data using the BioMark™ HD system, highlighting its utility for developing RH maps. Furthermore, the Janus™ (Perkin Elmer, MA, USA) and the Biomek™ (Beckman Coulter, CA, USA) units can pipet sub-microliter volumes accurately into hundreds or thousands of PCR plates per day. Alternatively, assays can be transferred to a completely different genotyping platform. For instance, Taqman™, Molecular Beacon™, and Scorpion® assays can be run on Fluidigm instruments that use automation and microfluidics to perform 96 single assays on 96 individual samples in a single plate. The reduction in assay volumes substantially decreases reagent costs, although the instruments themselves require the purchase of consumables [24]. The Dynamic Array does have some limitations, including the inability to run specific real-time PCR conditions for individual SNPs, and the need for specialized hardware. Nevertheless, the accuracy, efficiency, and cost savings in time, reagents, and DNA for a nanofluidics Dynamic Array make it suitable for medium- to high-throughput genotyping against targeted SNPs [25].

**Figure 5:**
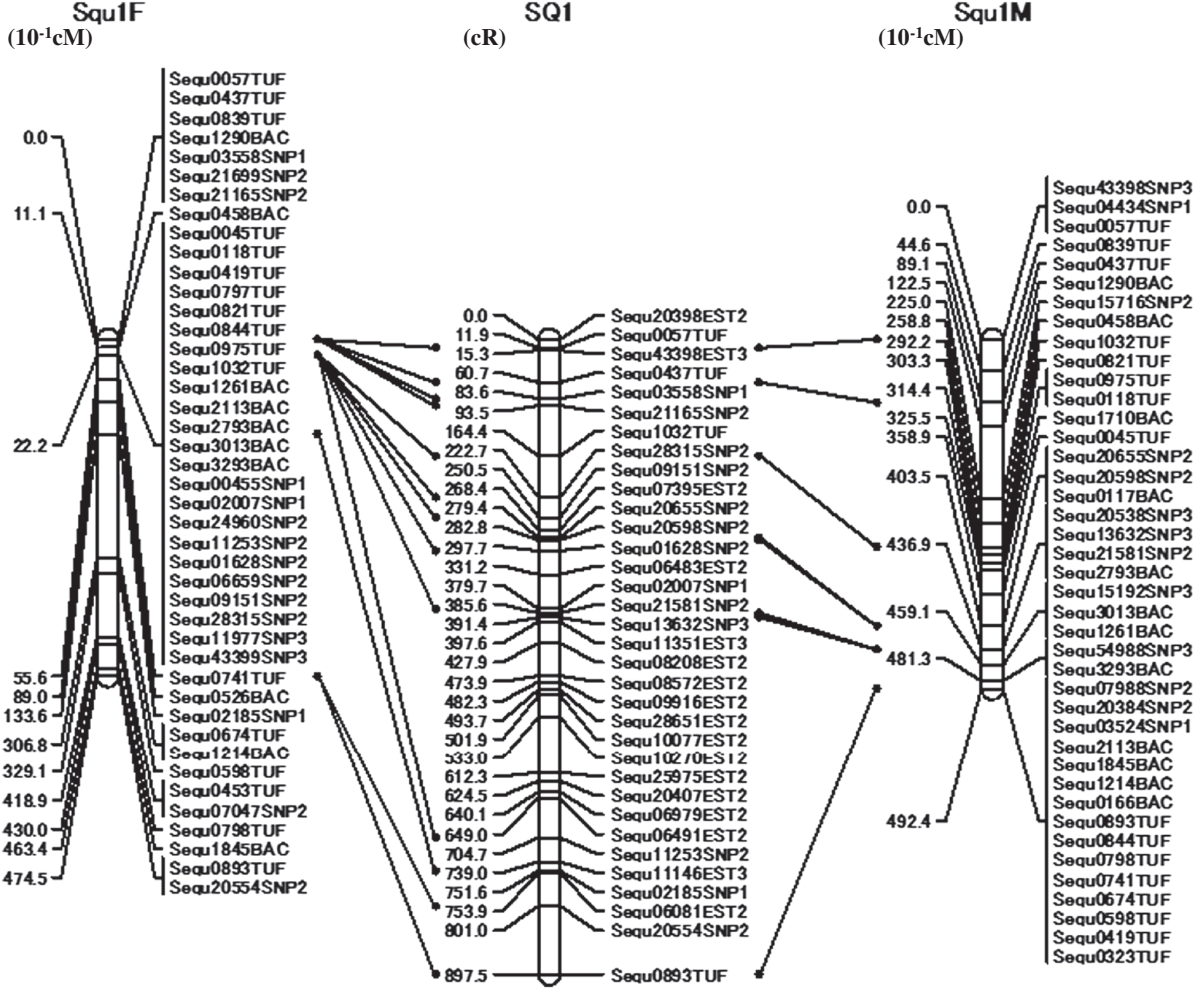
**Comparison of the RH group 1 (SQ1) and the linkage group 1 (Squ1).** The female Squ1 genetic linkage map is on the left, the male Squ1 map is on the right, and the RH SQ1 map is in the center. Solid lines connect the same markers.

After developing our RH map, RH group 1 (SQ1) and 23 other RH groups were compared with the genetic linkage groups (Figure 5 and Additional file 1). The distances between adjacent markers and the local order of markers were compared to confirm the accuracy of SQ1 in the yellowtail physical map. The order in which genes were arranged in SQ1 was the same as that observed in the yellowtail linkage map, demonstrating the local accuracy of the physical map. A manuscript fully describing the genetic linkage map data is in preparation, thus, other map data are not shown here. Once a high-density yellowtail RH map is constructed, this RH map will be compared with the 24 genetic linkage maps to confirm the accuracy of the local order of markers. To anchor the RH map to the karyotype, ESTs and microsatellite markers should be used simultaneously in FISH (fluorescence *in situ* hybridization) experiments and RH mapping [26].

### Synteny relationship with medaka

The 30 yellowtail marker sequences within the SQ1 RH map were compared with the cDNA sequences of three fish species: zebrafish (*Danio rerio*), medaka (*Oryzias latipes*), and the green-spotted pufferfish (*Tetraodon nigroviridis*), using the TBLASTX algorithm. Of the 30 sequences, 19 (63%) genes had identified orthologs in zebrafish and the green-spotted pufferfish, and 21 (70%) had recognizable orthologs in medaka (Table 2). Furthermore, we found syntenic relationships among SQ1, chromosome 5 of medaka, and chromosome 11 of the green-spotted pufferfish. However, the syntenic relationship between SQ1 and the zebrafish sequence was rather low. Medaka are a member of the order *Beloniformes,* which includes freshwater and marine fish such as Pacific saury and flying fish. Zebrafish are a member of *Cypriniformes*, which consists almost exclusively of freshwater fish. Notably, our SQ1 synteny results reflect the known taxonomic relationships of these fish. In Teleostei, it is thought that a whole-genome duplication and eight subsequent major rearrangements occurred about 314–404 million years ago [27]. Furthermore, it is thought that zebrafish and medaka diverged after the eight major rearrangement events. Therefore, the availability of the yellowtail RH panel enabling the construction of a high-density map offers an opportunity to investigate chromosomal evolution after the eight major rearrangements.

**Table 2:** Blast search of SQ1 gene sequences generated by model fish species

	***Danio rerio***		***Oryzias latipes***		***Tetraodon nigroviridis***	
	**Gene**	**Chr**	**Gene**	**Chr**	**Gene**	**Chr**
Sequ10077EST2	*CSE1L*	11	*CSE1L*	5	*CSE1L*	11
Sequ10270EST2	*RUVBL1*	6	*RUVBL1*	5	*RUVBL1*	7
Sequ11146EST3	–	11	*CSNK2A1*	16	*CSNK2A3*	–
Sequ11253SNP2	–	–	*SHQ1*	5	–	–
Sequ11351EST3	*AKR7A3*	11	*AKR7A3*	5	*AKR7A3*	11
Sequ13632SNP3	*TRIM62*	11	*TRIM62*	5	*TRIM62*	11
Sequ01628SNP2	–	–	–	–	–	–
Sequ02007SNP1	–	–	–	–	–	–
Sequ20398EST2	*AHCY*	6	*AHCY*	5	*AHCY*	–
Sequ20407EST2	*PSMD6*	11	*PSMD6*	5	*PSMD6*	11
Sequ20554SNP2	*TP53RK*	11	*TP53RK*	–	TP53RK	–
Sequ20598SNP2	*IGFN1*	6	*IGFN1*	5	*IGFN1*	11
Sequ20655SNP2	*IGFN1*	6	*IGFN1*	5	*IGFN1*	9
Sequ21165SNP2	–	–	–	–	–	
Sequ21581SNP2	–	–	–	–	–	
Sequ02185SNP1	–	–	–	–	–	
Sequ25975EST2	*UBAP1*	21	*UBAP1*	5	*UBAP1*	11
Sequ28315SNP2	*ZGC*	23	*MYL6*	7	–	11
Sequ28651EST2	*WDR77*	–	*WDR77*	5	*WDR77*	11
Sequ03558SNP1	–	–	–	–	–	–
Sequ43398EST3	–	–	–	–	–	–
Sequ06081EST2	*ALDH1L1*	6	*ALDH1L1*	5	*ALDH1L1*	–
Sequ06483EST2	*TIMM17A*	11	*TIMM17A*	5	*TIMM17A*	11
Sequ06491EST2	*RPN1*	11	*RPN1*	5	*RPN1*	11
Sequ06979EST2	*SEC61A1*	6	*SEC61A1*	5	*SEC61A1*	11
Sequ07395EST2	*CAPZA1*	8	–	5	–	13
Sequ08208EST2	*GNAT1*	6	*GNAT1*	–	*GNAT1*	11
Sequ08572EST2	–	–	*ATAD3A*	–	*ATAD3A*	11
Sequ09151SNP2	–	–	–	–	–	–
Sequ09916EST2	*BYSL*	22	*BYSL*	5	*BYSL*	11

The production of a high-density RH map with about 2,000 ESTs is underway and will provide information on conserved synteny and chromosome evolution in fish species. Furthermore, the high-density yellowtail RH map can be used to highlight associations with economically important breeding traits using SNP chips, and identify their chromosomal location.

## Conclusions

A yellowtail RH panel was constructed to facilitate the localization of markers from 421 hybrid cell lines. Then, the framework RH map was constructed with 580 markers containing ESTs and SNPs. Information from the high-density physical map will facilitate the discovery of linked regions associated with economically important traits. This will, in turn, aid the development of yellowtail strains suitable for aquaculture.

## Methods

### Ethical statement

Field permits are not required for yellowtail (*Seriola quinqueradiata*) in Japan. The fish handling, husbandry, and sampling methods were approved by the Institute for Animal Care and Use Committee of the National Research Institute of Aquaculture (IACUC-NRIA No. 3).

### Visualization of chromosomes in a fibroblast cell line

A yellowtail fin fibroblast cell line was derived and cultured. Then, cell cycle progression was inhibited by the addition of 10 μM TN-16 (Calbiochem, CA, USA) at 22°C for 24 h. The cells were trypsinized, washed twice in phosphate-buffered saline, treated with Optimal Hypotonic (Genial Genetics, Chester, UK) at 37°C for 5 min, and fixed in a methyl alcohol/acetic acid solution (3:1). One drop of the cell suspension was placed on a warm slide and dried. This slide was stained with Giemsa Stain Solution (Wako, Osaka, Japan) for 12 h [28].

### Production of the RH cell lines

Both the calcium phosphate precipitation method [29] and the lipofection method using the Lipofectamine 2,000 Reagent (Invitrogen, CA, USA) were used to transfer the Geneticin® resistant gene, pSV2neo, into the yellowtail fin cells. The treated cells were cultured in L-15 medium (Invitrogen, USA) containing Geneticin® (1 mg/mL, Invitrogen, USA) at 22°C to select the transformed cells. More than 300 independent Geneticin®-resistant clones were pooled for the fusion experiments. These cells were irradiated by exposure to 3,000, 4,000, 5,000, 6,000, 8,000, or 10,000 rads (MBR-1520R; Hitachi Power Solutions Co., Ltd., Ibaraki, Japan), and were fused with derivative mouse B78 cells at a 2:1 ratio in the presence of polyethylene glycol 1500 (Roche, Basel, Switzerland). The cells were cultivated with DMEM medium (Invitrogen, USA) containing Geneticin® for 3–4 weeks until hybrid clone colonies appeared. DNA was extracted from individual clones using a DNAdvance Kit (Beckman Coulter, CA, USA).

### Selection of an RH panel

The DNA extracted from hybrid cells was expanded by whole genome amplification using a GenomiPhi V2 DNA Amplification Kit (GE Healthcare, Buckinghamshire, UK). PCR was performed on 60 ng of amplified DNA using BIOTaq HS DNA Polymerase (Bioline, London, UK) in a final volume of 15 μL. Microsatellite DNA markers were used to genotype the 94 markers mapped in the yellowtail linkage map [2, unpublished observations]. Amplification was performed over 40 cycles: 30 s at 95°C, 10 s at 62°C, and 30 s at 72°C, in addition to a 10-min pre-dwell at 95°C and a 3-min post-dwell at 72°C. The PCR products were analyzed by electrophoresis in a 2% agarose gel, and were scored as present, absent, or ambiguous. After analysis of the retention profiles of the 94 microsatellite markers in each cell line, a total of 93 RH cell lines were selected for the RH panel. Each cell line within the RH panel was cultured to confluence in two 75 cm^2^ dishes, yielding 1 × 10^7^ cells. Then, genomic DNA from each cell line was extracted using the Blood & Cell Culture DNA Midi Kit (Qiagen, Hilden, Germany).

#### Genotyping

Genotyping analysis was performed on 96 DNA samples including material from 93 hybrid RH cell lines. Yellowtail DNA and a mixture of yellowtail and mouse DNA were used as two independent positive controls. Mouse DNA alone was used as a negative control.

For expressed sequences, total RNA purified from the brain, muscle, liver, heart, intestine, kidney, spleen, gonad, bladder, and gills of one immature yellowtail was sequenced using a Roche/454 FLX Pyrosequencer employing a next-generation sequencing technique with reference sequences [unpublished observations]. Furthermore, total RNA was prepared from 500 juvenile yellowtail and from the kidneys and gills of 100 young yellowtail individuals and sequenced using a Roche/454 FLX Pyrosequencer. Then, these sequences and the reference sequences were assembled and analyzed using a CLC Genomics Workbench (CLC bio, Aarhus, Denmark). Oligonucleotide primer pair DNA markers were derived from the yellowtail expressed sequences, and were designed using Primer 3 software [30] (see Additional files 2 and 3).

Genotyping was carried out using the BioMark™ HD system (Fluidigm, USA), which requires a pre-amplification reaction. Primer pairs for the pre-amplification PCR and genotyping reactions were designed based on the expression sequences of the ESTs. Pre-amplification PCR product size was designed to be in the range of 100–150 bp, and the second PCR product size in the BioMark™ HD system was designed to be in the range of 50–100 bp. Pre-amplification PCR was carried out using a Multiplex PCR Assay Kit (TaKaRa, Shiga, Japan) with 96 pooled primer pairs tested against each DNA sample (60 ng) in the RH panel, and against the positive and negative controls. Cycle conditions were as follows: a pre-dwell for 1 min at 94°C; 20 cycles of 30 s at 94°C, 90 s at 62°C, and 90 s at 72°C; and a post-dwell for 10 min at 72°C. The products of the pre-amplification PCR were diluted 1:5 with TE.

Genotyping reactions were carried out on the Fluidigm platform using a BioMark™ 96.96 nanofluidic dynamic array (Fluidigm, USA) for gene expression analysis. A 5 μL sample mixture was prepared for each sample containing diluted pre-amplified DNA, TaqMan Gene Expression Master Mix (Applied Biosystems, CA, USA), EvaGreen (Biotium, CA, USA), and DNA Binding Sample Loading Reagent (Fluidigm, USA). An assay mix of 5 μL was prepared with each nested primer from the pre-amplification PCR, and Assay Loading Reagent (Fluidigm, USA). An IFC controller HX was used to prime the fluidic array with control line fluid, and then the samples and assay mix were combined in the inlets. After loading, the array was placed in the BioMark™ HD system for PCR under the following cycle conditions: a pre-dwell for 10 min at 95°C, 40 cycles of 15 s at 95°C, 10 s at 62°C, and 20 s at 72°C. The results were analyzed using the Real-Time PCR Analysis software, which is integrated into the BioMark™ HD system.

#### Data computation

We used CarthaGene software to perform two-point linkage analyses and to determine marker order and inter-marker distances in centi Rays (cR). Linkage groups were determined using the group command at an LOD threshold of 4.0 and a distance threshold of 45, and referenced the genetic yellowtail linkage map [2, unpublished observations].

EST alignments of linkage group 1 (SQ1) were performed with the TBLASTX algorithm searches against the cDNA database of zebrafish (*Danio rerio*), medaka (*Oryzias latipes*), and the green spotted pufferfish (*Tetraodon nigroviridis*) using Ensembl [31]. The sequences with the lowest E-value (< 1.0 × 10^−5^) were adopted in each EST alignment.

## Competing interests

The authors declare that they have no competing interests.

## Authors’ contributions

JA and KA conceived and designed the study, and inspected yellowtail chromosome preparations. JA constructed the RH cell lines, the RH panel, and the RH map, performed the comparison of the RH and genetic linkage maps, and wrote the draft manuscript. WK, YK, AO, KF, TK, and TS contributed to the construction of the genetic linkage map including the microsatellite markers and SNPs. KY and TT bred the experimental fish. All of the authors read and approved the final version of the manuscript.

## Supplementary Material

Additional file 1 A comparison of the RH and genetic linkage maps. Click here for file

Additional file 2 The names and nucleotide sequences of markers located on the RH map. Click here for file

Additional file 3 Primer pairs of markers on the RH map. Click here for file
