# Supplementary material for: Construction of a radiation hybrid panel and the first yellowtail (Seriola quinqueradiata) radiation hybrid map using a nanofluidic dynamic array
Source: BMC Genomics. 2014 Feb 27;15:165. doi: 10.1186/1471-2164-15-165 (PMC3943507; doi:10.1186/1471-2164-15-165)
Supplement: Additional file 1 — A comparison of the RH and genetic linkage maps. [file 1471-2164-15-165-S1.pdf]

Additional file 1. Comparison of RH and genetic linkage maps. Genetic linkage maps of female is on the left and that of male is on the right, and the RH map is in the center. Solid lines connect the same marker placed on maps.

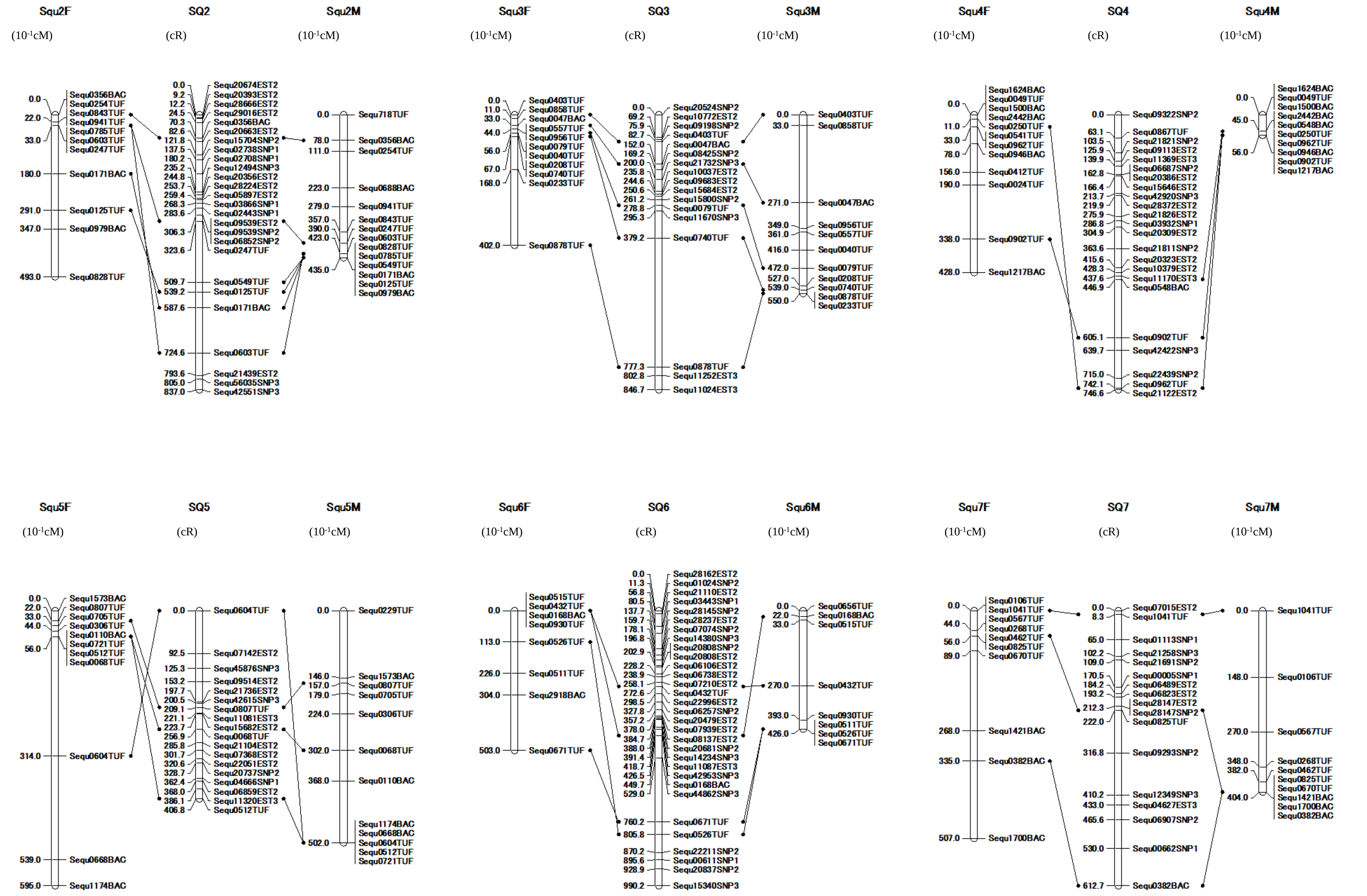

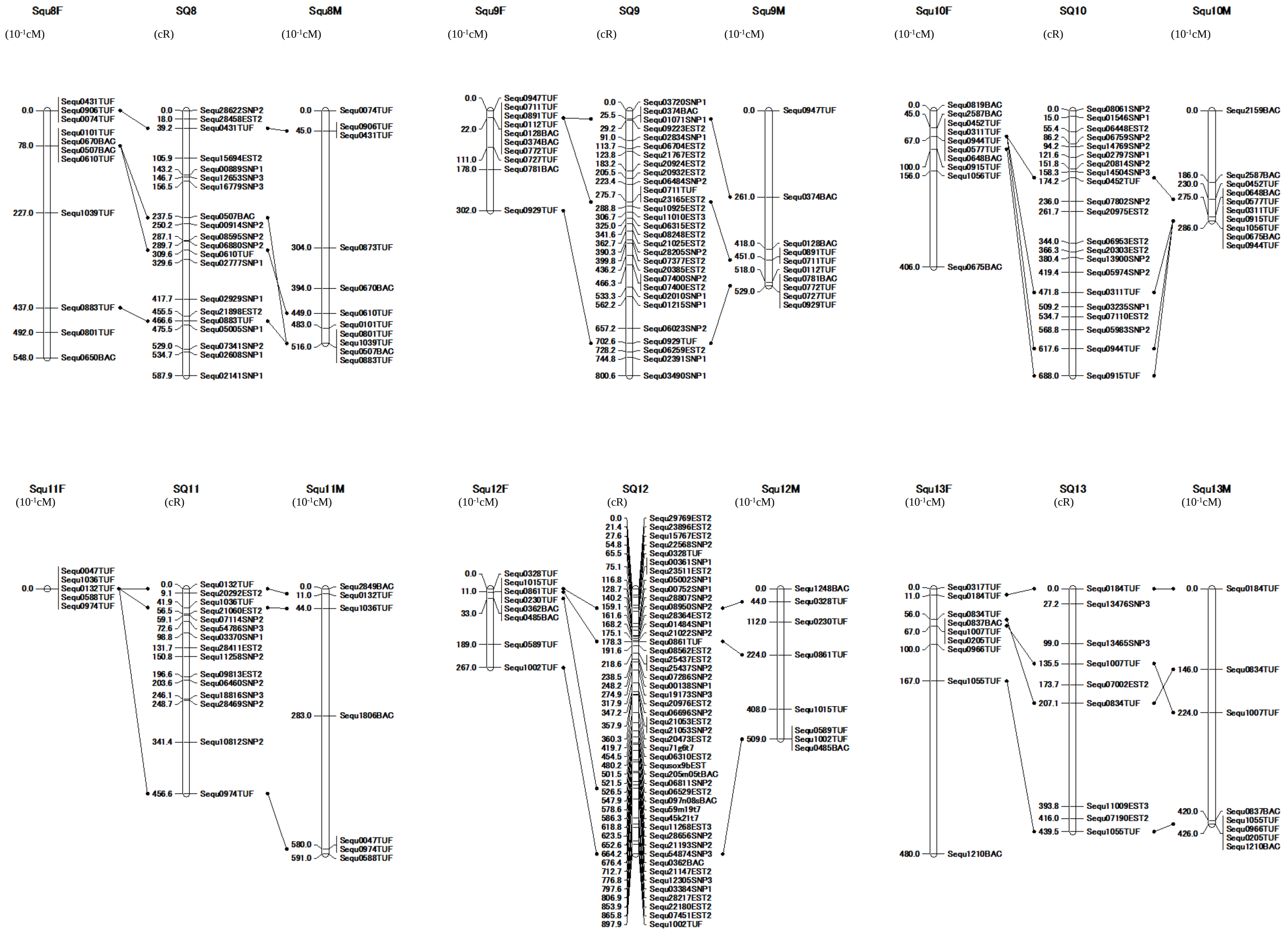

Squ8F
(10^{-1}cM)
0.0 Sequ0431TUF Sequ0906TUF Sequ0074TUF
78.0 Sequ0101TUF Sequ0670BAC Sequ0507BAC Sequ0610TUF
227.0 Sequ1039TUF
437.0 Sequ0883TUF
492.0 Sequ0801TUF
548.0 Sequ0650BAC
SQ8
(cR)
0.0 Sequ28622SNP2
18.0 Sequ28458EST2
39.2 Sequ0431TUF
105.9 Sequ15694EST2
143.2 Sequ00889SNP1
146.7 Sequ12653SNP3
156.5 Sequ16779SNP3
237.5 Sequ0507BAC
250.2 Sequ00914SNP2
287.1 Sequ08595SNP2
289.7 Sequ06880SNP2
309.6 Sequ0610TUF
329.6 Sequ02777SNP1
417.7 Sequ02929SNP1
455.5 Sequ21898EST2
466.6 Sequ0883TUF
475.5 Sequ05005SNP1
529.0 Sequ07341SNP2
534.7 Sequ02608SNP1
587.9 Sequ02141SNP1
Squ8M
(10^{-1}cM)
0.0 Sequ0074TUF
45.0 Sequ0906TUF Sequ0431TUF
304.0 Sequ0873TUF
394.0 Sequ0670BAC
449.0 Sequ0610TUF
483.0 Sequ0101TUF
516.0 Sequ0801TUF Sequ1039TUF Sequ0507BAC Sequ0883TUF
Squ9F
(10^{-1}cM)
0.0 22.0 111.0 178.0
Sequ0947TUF Sequ0711TUF Sequ0891TUF Sequ0112TUF Sequ0128BAC Sequ0374BAC Sequ0772TUF Sequ0727TUF Sequ0781BAC
302.0 Sequ0929TUF
SQ9
(cR)
0.0 25.5 29.2 91.0 113.7 123.8 183.2 205.5 223.4 275.7 288.8 306.7 325.0 341.6 362.7 390.3 399.8 436.2 466.3 533.3 562.2
Sequ03720SNP1 Sequ0374BAC Sequ01071SNP1 Sequ09223EST2 Sequ02834SNP1 Sequ06704EST2 Sequ21767EST2 Sequ20924EST2 Sequ20932EST2 Sequ06484SNP2 Sequ0711TUF Sequ23165EST2 Sequ10925EST2 Sequ11010EST3 Sequ06315EST2 Sequ08248EST2 Sequ21025EST2 Sequ28205SNP2 Sequ07377EST2 Sequ20385EST2 Sequ07400SNP2 Sequ07400EST2 Sequ02010SNP1 Sequ01215SNP1
657.2 Sequ06023SNP2
702.6 Sequ0929TUF
728.2 Sequ06259EST2
744.8 Sequ02391SNP1
800.6 Sequ03490SNP1
Squ9M
(10^{-1}cM)
0.0 Sequ0947TUF
261.0 Sequ0374BAC
418.0 451.0 518.0 529.0
Sequ0128BAC Sequ0891TUF Sequ0711TUF Sequ0112TUF Sequ0781BAC Sequ0772TUF Sequ0727TUF Sequ0929TUF
Squ10F
(10^{-1}cM)
0.0 45.0 67.0 100.0 156.0
Sequ0819BAC Sequ2587BAC Sequ0452TUF Sequ0311TUF Sequ0944TUF Sequ0577TUF Sequ0648BAC Sequ0915TUF Sequ1056TUF
406.0 Sequ0675BAC
SQ10
(cR)
0.0 Sequ08061SNP2
15.0 Sequ01546SNP1
55.4 Sequ06448EST2
86.2 Sequ06759SNP2
94.2 Sequ14769SNP2
121.6 Sequ02797SNP1
151.8 Sequ20814SNP2
158.3 Sequ14504SNP3
174.2 Sequ0452TUF
236.0 Sequ07802SNP2
261.7 Sequ20975EST2
344.0 Sequ06953EST2
366.3 Sequ20303EST2
380.4 Sequ13900SNP2
419.4 Sequ05974SNP2
471.8 Sequ0311TUF
509.2 Sequ03235SNP1
534.7 Sequ07110EST2
568.8 Sequ05983SNP2
617.6 Sequ0944TUF
688.0 Sequ0915TUF
Squ10M
(10^{-1}cM)
0.0 Sequ2159BAC
186.0 230.0 275.0 286.0
Sequ2587BAC Sequ0452TUF Sequ0648BAC Sequ0577TUF Sequ0311TUF Sequ0915TUF Sequ1056TUF Sequ0675BAC Sequ0944TUF
Squ11F
(10^{-1}cM)
0.0 Sequ0047TUF Sequ1036TUF Sequ0132TUF Sequ0588TUF Sequ0974TUF
SQ11
(cR)
0.0 Sequ0132TUF
9.1 Sequ20292EST2
41.9 Sequ1036TUF
56.5 Sequ21060EST2
59.1 Sequ07114SNP2
72.6 Sequ54786SNP3
98.8 Sequ03370SNP1
131.7 Sequ28411EST2
150.8 Sequ11258SNP2
196.6 Sequ09813EST2
203.6 Sequ06460SNP2
246.1 Sequ18816SNP3
248.7 Sequ28469SNP2
341.4 Sequ10812SNP2
456.6 Sequ0974TUF
Squ11M
(10^{-1}cM)
0.0 Sequ2849BAC
11.0 Sequ0132TUF
44.0 Sequ1036TUF
283.0 Sequ1806BAC
580.0 591.0
Sequ0047TUF Sequ0974TUF Sequ0588TUF
Squ12F
(10^{-1}cM)
0.0 11.0 33.0
Sequ0328TUF Sequ1015TUF Sequ0861TUF Sequ0230TUF Sequ0362BAC Sequ0485BAC
189.0 Sequ0589TUF
267.0 Sequ1002TUF
SQ12
(cR)
0.0 21.4 27.6 54.8 65.5 75.1 116.8 128.7 140.2 159.1 161.6 168.2 175.1 178.3 191.6 218.6 238.5 248.2 274.9 317.9 347.2 357.9 360.3 419.7 454.5 480.2 501.5 521.5 526.5 547.9 578.6 586.3 618.8 623.5 652.6 664.2 676.4 712.7 776.8 797.6 806.9 853.9 865.8 897.9
Sequ29769EST2 Sequ23896EST2 Sequ15767EST2 Sequ22568SNP2 Sequ0328TUF Sequ00361SNP1 Sequ23511EST2 Sequ05002SNP1 Sequ00752SNP1 Sequ28807SNP2 Sequ08950SNP2 Sequ28364EST2 Sequ01484SNP1 Sequ21022SNP2 Sequ0861TUF Sequ08562EST2 Sequ25437EST2 Sequ25437SNP2 Sequ07286SNP2 Sequ00138SNP1 Sequ19173SNP3 Sequ20976EST2 Sequ06696SNP2 Sequ21053EST2 Sequ21053SNP2 Sequ20473EST2 Sequ71g6t7 Sequ06310EST2 Sequsox9bEST Sequ205m05tBAC Sequ06811SNP2 Sequ06529EST2 Sequ097n08sBAC Sequ59m19t7 Sequ45k21t7 Sequ11268EST3 Sequ28656SNP2 Sequ21193SNP2 Sequ54874SNP3 Sequ0362BAC Sequ21147EST2 Sequ12305SNP3 Sequ03384SNP1 Sequ28217EST2 Sequ22180EST2 Sequ07451EST2 Sequ1002TUF
Squ12M
(10^{-1}cM)
0.0 Sequ1248BAC
44.0 Sequ0328TUF
112.0 Sequ0230TUF
224.0 Sequ0861TUF
408.0 Sequ1015TUF
509.0 Sequ0589TUF Sequ1002TUF Sequ0485BAC
Squ13F
(10^{-1}cM)
0.0 11.0 Sequ0317TUF Sequ0184TUF
56.0 67.0 100.0 Sequ0834TUF Sequ0837BAC Sequ1007TUF Sequ0205TUF Sequ0966TUF
167.0 Sequ1055TUF
480.0 Sequ1210BAC
SQ13
(cR)
0.0 Sequ0184TUF
27.2 Sequ13476SNP3
99.0 Sequ13465SNP3
135.5 Sequ1007TUF
173.7 Sequ07002EST2
207.1 Sequ0834TUF
393.8 Sequ11009EST3
416.0 Sequ07190EST2
439.5 Sequ1055TUF
Squ13M
(10^{-1}cM)
0.0 Sequ0184TUF
146.0 Sequ0834TUF
224.0 Sequ1007TUF
420.0 426.0
Sequ0837BAC Sequ1055TUF Sequ0966TUF Sequ0205TUF Sequ1210BAC

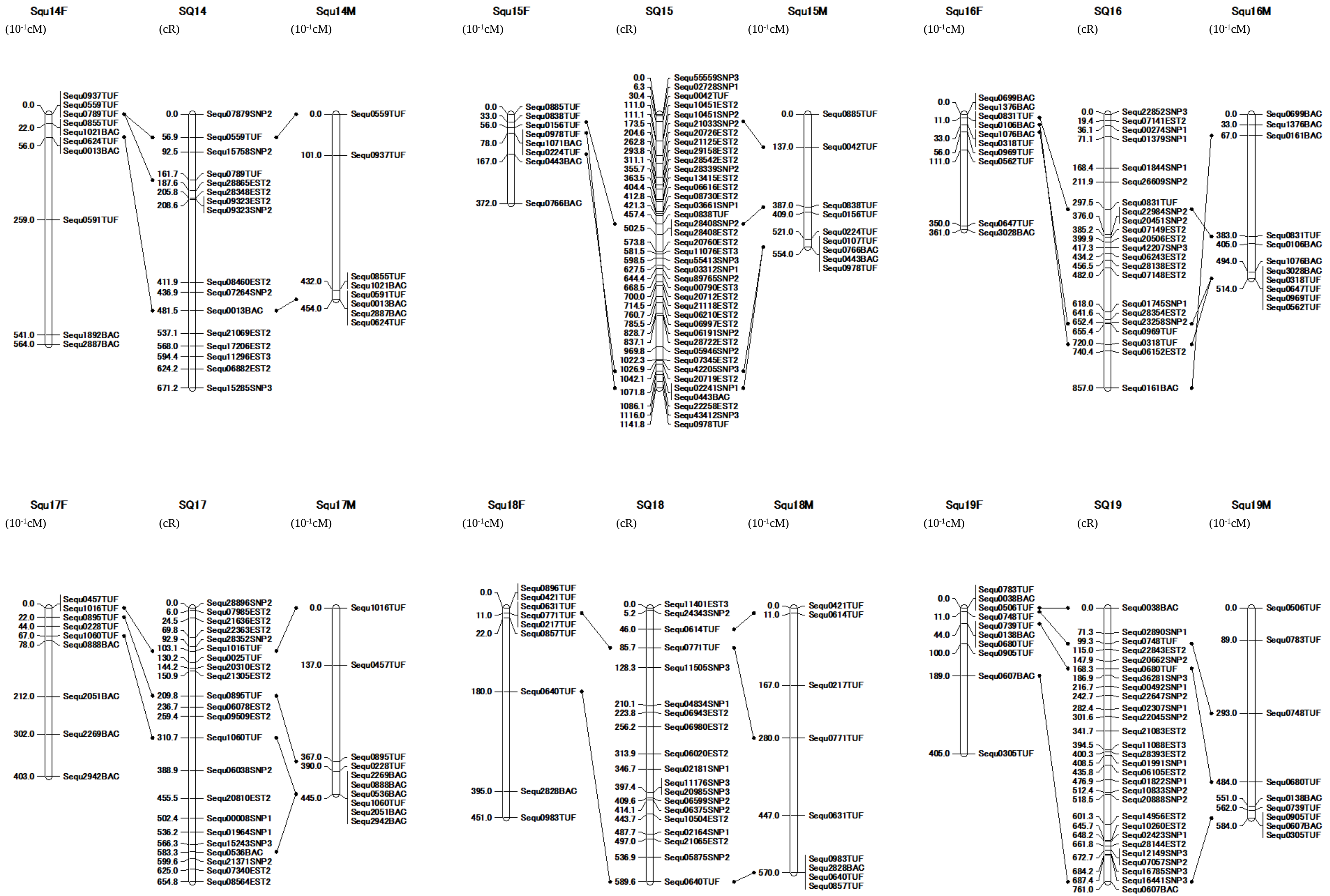
Squ14F
(10⁻¹cM)
SQ14
(cR)
Squ14M
(10⁻¹cM)
Squ15F
(10⁻¹cM)
SQ15
(cR)
Squ15M
(10⁻¹cM)
Squ16F
(10⁻¹cM)
SQ16
(cR)
Squ16M
(10⁻¹cM)
Squ17F
(10⁻¹cM)
SQ17
(cR)
Squ17M
(10⁻¹cM)
Squ18F
(10⁻¹cM)
SQ18
(cR)
Squ18M
(10⁻¹cM)
Squ19F
(10⁻¹cM)
SQ19
(cR)
Squ19M
(10⁻¹cM)

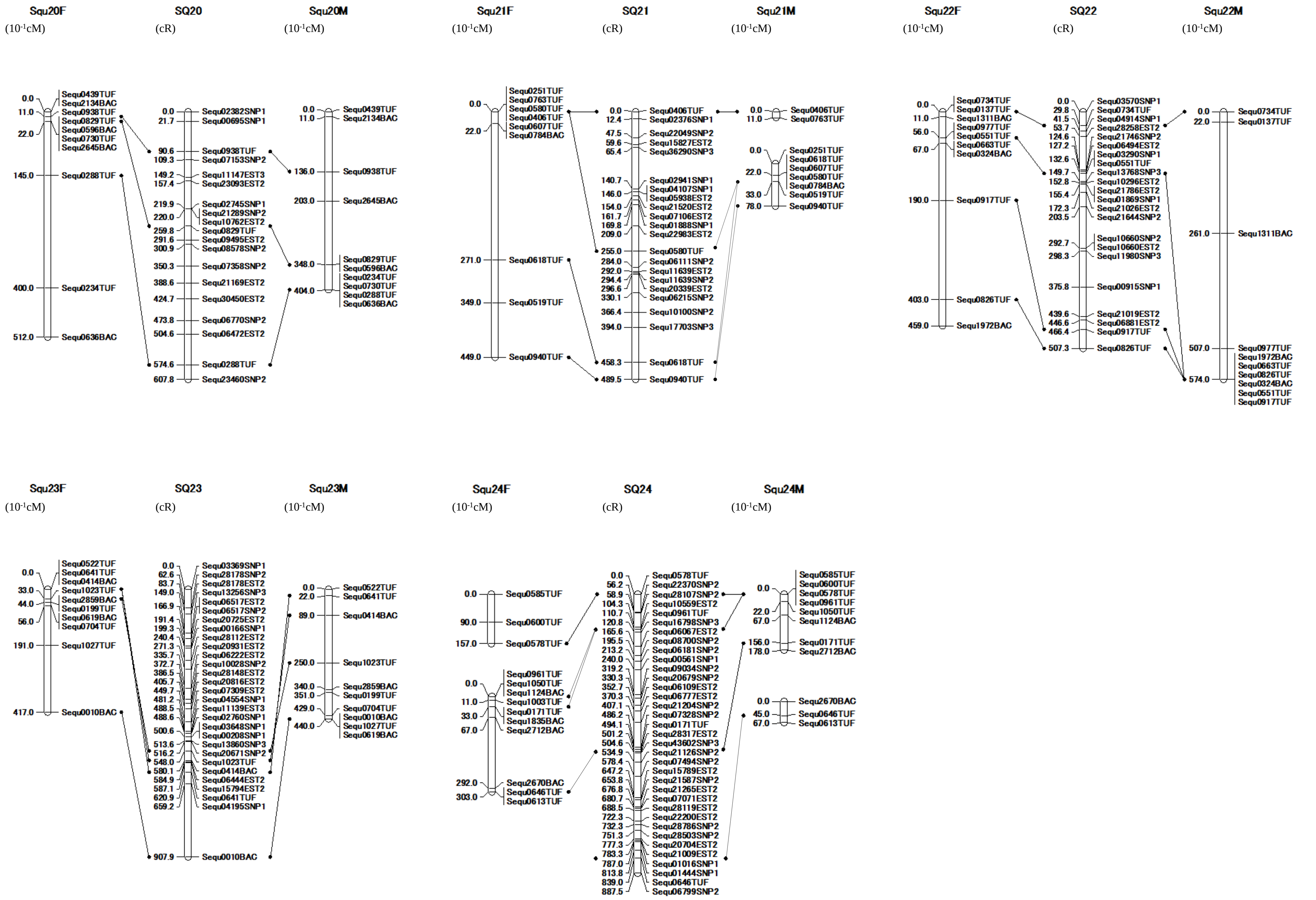

Squ20F
(10⁻¹cM)
0.0 Sequ0439TUF
Sequ2134BAC
11.0 Sequ0938TUF
Sequ0829TUF
22.0 Sequ0596BAC
Sequ0730TUF
Sequ2645BAC
145.0 Sequ0288TUF
400.0 Sequ0234TUF
512.0 Sequ0636BAC
SQ20
(cR)
0.0 Sequ02382SNP1
21.7 Sequ00695SNP1
90.6 Sequ0938TUF
109.3 Sequ07153SNP2
149.2 Sequ11147EST3
157.4 Sequ23093EST2
219.9 Sequ02745SNP1
220.0 Sequ21289SNP2
Sequ10762EST2
259.8 Sequ0829TUF
291.6 Sequ09495EST2
300.9 Sequ08578SNP2
350.3 Sequ07358SNP2
388.6 Sequ21169EST2
424.7 Sequ30450EST2
473.8 Sequ06770SNP2
504.6 Sequ06472EST2
574.6 Sequ0288TUF
607.8 Sequ23460SNP2
Squ20M
(10⁻¹cM)
0.0 Sequ0439TUF
11.0 Sequ2134BAC
136.0 Sequ0938TUF
203.0 Sequ2645BAC
348.0 Sequ0829TUF
Sequ0596BAC
404.0 Sequ0234TUF
Sequ0730TUF
Sequ0288TUF
Sequ0636BAC
Squ21F
(10⁻¹cM)
0.0 Sequ0251TUF
Sequ0763TUF
Sequ0580TUF
Sequ0406TUF
22.0 Sequ0607TUF
Sequ0784BAC
271.0 Sequ0618TUF
349.0 Sequ0519TUF
449.0 Sequ0940TUF
SQ21
(cR)
0.0 Sequ0406TUF
12.4 Sequ02376SNP1
47.5 Sequ22049SNP2
59.6 Sequ15827EST2
65.4 Sequ36290SNP3
140.7 Sequ02941SNP1
146.0 Sequ04107SNP1
Sequ05938EST2
154.0 Sequ21520EST2
161.7 Sequ07106EST2
169.8 Sequ01888SNP1
209.0 Sequ22983EST2
255.0 Sequ0580TUF
284.0 Sequ06111SNP2
292.0 Sequ11639EST2
294.4 Sequ11639SNP2
296.6 Sequ20339EST2
330.1 Sequ06215SNP2
366.4 Sequ10100SNP2
394.0 Sequ17703SNP3
458.3 Sequ0618TUF
489.5 Sequ0940TUF
Squ21M
(10⁻¹cM)
0.0 Sequ0406TUF
11.0 Sequ0763TUF
0.0 Sequ0251TUF
Sequ0618TUF
22.0 Sequ0607TUF
Sequ0580TUF
Sequ0784BAC
33.0 Sequ0519TUF
78.0 Sequ0940TUF
Squ22F
(10⁻¹cM)
0.0 Sequ0734TUF
Sequ0137TUF
11.0 Sequ1311BAC
56.0 Sequ0977TUF
Sequ0551TUF
67.0 Sequ0663TUF
Sequ0324BAC
190.0 Sequ0917TUF
403.0 Sequ0826TUF
459.0 Sequ1972BAC
SQ22
(cR)
0.0 Sequ03570SNP1
29.8 Sequ0734TUF
41.5 Sequ04914SNP1
53.7 Sequ28258EST2
124.6 Sequ21746SNP2
127.2 Sequ06494EST2
Sequ03290SNP1
132.6 Sequ0551TUF
149.7 Sequ13768SNP3
152.8 Sequ10296EST2
155.4 Sequ21786EST2
Sequ01869SNP1
172.3 Sequ21026EST2
203.5 Sequ21644SNP2
292.7 Sequ10660SNP2
Sequ10660EST2
298.3 Sequ11980SNP3
375.8 Sequ00915SNP1
439.6 Sequ21019EST2
446.6 Sequ06881EST2
466.4 Sequ0917TUF
507.3 Sequ0826TUF
Squ22M
(10⁻¹cM)
0.0 Sequ0734TUF
22.0 Sequ0137TUF
261.0 Sequ1311BAC
507.0 Sequ0977TUF
Sequ1972BAC
Sequ0663TUF
Sequ0826TUF
574.0 Sequ0324BAC
Sequ0551TUF
Sequ0917TUF
Squ23F
(10⁻¹cM)
Sequ0522TUF
0.0 Sequ0641TUF
Sequ0414BAC
33.0 Sequ1023TUF
Sequ2859BAC
44.0 Sequ0199TUF
Sequ0619BAC
56.0 Sequ0704TUF
191.0 Sequ1027TUF
417.0 Sequ0010BAC
SQ23
(cR)
0.0 Sequ03369SNP1
62.6 Sequ28178SNP2
83.7 Sequ28178EST2
149.0 Sequ13256SNP3
166.9 Sequ06517EST2
Sequ06517SNP2
191.4 Sequ20725EST2
199.3 Sequ00166SNP1
240.4 Sequ28112EST2
271.3 Sequ20931EST2
335.7 Sequ06222EST2
372.7 Sequ10028SNP2
386.5 Sequ28148EST2
405.7 Sequ20816EST2
449.7 Sequ07309EST2
481.2 Sequ04554SNP1
488.5 Sequ11139EST3
488.6 Sequ02760SNP1
500.6 Sequ03648SNP1
Sequ00208SNP1
513.6 Sequ13860SNP3
516.2 Sequ20671SNP2
548.0 Sequ1023TUF
580.1 Sequ0414BAC
584.9 Sequ06444EST2
587.1 Sequ15794EST2
620.9 Sequ0641TUF
659.2 Sequ04195SNP1
907.9 Sequ0010BAC
Squ23M
(10⁻¹cM)
0.0 Sequ0522TUF
22.0 Sequ0641TUF
89.0 Sequ0414BAC
250.0 Sequ1023TUF
340.0 Sequ2859BAC
351.0 Sequ0199TUF
429.0 Sequ0704TUF
440.0 Sequ0010BAC
Sequ1027TUF
Sequ0619BAC
Squ24F
(10⁻¹cM)
0.0 Sequ0585TUF
90.0 Sequ0600TUF
157.0 Sequ0578TUF
0.0 Sequ0961TUF
Sequ1050TUF
Sequ1124BAC
11.0 Sequ1003TUF
33.0 Sequ0171TUF
Sequ1835BAC
67.0 Sequ2712BAC
292.0 Sequ2670BAC
303.0 Sequ0646TUF
Sequ0613TUF
SQ24
(cR)
0.0 Sequ0578TUF
56.2 Sequ22370SNP2
58.9 Sequ28107SNP2
104.3 Sequ10559EST2
110.7 Sequ0961TUF
120.8 Sequ16798SNP3
165.6 Sequ06067EST2
195.5 Sequ08700SNP2
213.2 Sequ06181SNP2
240.0 Sequ00561SNP1
319.2 Sequ09034SNP2
330.3 Sequ20679SNP2
352.7 Sequ06109EST2
370.3 Sequ06777EST2
407.1 Sequ21204SNP2
486.2 Sequ07328SNP2
494.1 Sequ0171TUF
501.2 Sequ28317EST2
504.6 Sequ43602SNP3
534.9 Sequ21126SNP2
578.4 Sequ07494SNP2
647.2 Sequ15789EST2
653.8 Sequ21587SNP2
676.8 Sequ21265EST2
680.7 Sequ07071EST2
688.5 Sequ28119EST2
722.3 Sequ22200EST2
732.3 Sequ28786SNP2
751.3 Sequ28503SNP2
777.3 Sequ20704EST2
783.3 Sequ21009EST2
787.0 Sequ01016SNP1
813.8 Sequ01444SNP1
839.0 Sequ0646TUF
887.5 Sequ06799SNP2
Squ24M
(10⁻¹cM)
Sequ0585TUF
Sequ0600TUF
0.0 Sequ0578TUF
Sequ0961TUF
22.0 Sequ1050TUF
67.0 Sequ1124BAC
156.0 Sequ0171TUF
178.0 Sequ2712BAC
0.0 Sequ2670BAC
45.0 Sequ0646TUF
67.0 Sequ0613TUF
