# Supplementary material for: Construction of a radiation hybrid panel and the first yellowtail (Seriola quinqueradiata) radiation hybrid map using a nanofluidic dynamic array
Source: BMC Genomics. 2014 Feb 27;15:165. doi: 10.1186/1471-2164-15-165 (PMC3943507; doi:10.1186/1471-2164-15-165)
Supplement: Additional file 2 — The names and nucleotide sequences of markers located on the RH map. [file 1471-2164-15-165-S2.pdf]

Additional file 2.
Names and nucleotide sequences of markers located on the RH map.

>Sequ00005SNP1
CCATCATTCATTTTATTATTTATACTCATGCTTTACATTCTGTGACACATCGGAAAGTTTTTCCATTAGCTGGACTG
ATAAATTTACCCCTTGACTCGATCATATCTTTGCTTAAAATTATAATGTGCCATGATAGATTTTTGCAAACAAGTAG
CCTAAATTTTAAAGTCTCTTTATTTTCTGATTACTTTGCCAAATTATGAAATGGCCCTTTAGTCTTTCATCACATAC
GTGGCAAAGTCAGAAGTCAAATTCCTACAGACTAACTGCTGAATGAAACCCACCCAGTGTCGTCGCCGTCCTTTCAG
GAGGCACACAATACTCATGTGCAATGTTCATATATATTTAGATGTTTTATTTTGTTCTTTGCCAAACAAAGGCAGAA
ATACCTGTTTACACGATTTAACTTGTTGGATTCGATCCAAAAATGAATGATGTAATCCAAGGAGTTACATTACAAGT
CCAGGCAAACAGTAACAGAGTAATTGCTAATATTGCATTATAACATCATTTCAGTGATAAGAATGACGCAGTGGGAT
CTGAATAATTCAATAAGTACATTTCAAAAAATCAATCAGTTCAACAAAACAACCCAAAAGGACTTTGTGTATATACA
ATATATATCATCAAACAAAATAATATAGCTCTATTTGTTCATCACCATCTTTCCAATTCTCTTACATTTACAAAAAT
GTCTCAGTACATGATTTTCAGTCTTTGCATAGCTGTGTATTCTCTCTGATAACTATTTGATATTGCTTGGCTTTTCT
TTGCAGCACTACATTTGGATTTTAACATTATTACGTTTGTTCTGAATATGTACAAGTTAATCTGTCAAGGAGCTTTA
GAGAAAGACAGTGTGAT

>Sequ00008SNP1
AAGCAGTCAAAGCAGATTTATTTCTTGTTTAGCCTAATGGCTGATGGTTAGCTTTATAACAAACGTTGTTAAAACATA
AGCATTTTGGGAAGATATTGGCTTTAAAGCTTGCAGAAAATAAATTATACCCCGCCTCTTTTGTATCTTCTTCATCTT
TTGTGTTTTCTGTATACTTTAGATCAATTTCCCATATACTATATTAAATTTGTTACCACAATATACTCCATTAGAAAT
GTGTTTTTGTCAGAAAGAGGGAATGTGATGCTTAAGTTATTGTTGCTGGGATAAGAGCTTTATTAGAGCATCAACTTA
AAAAGACTGTTTGGTGAGGTTATTCCAAATTATATAGAAAGATGAGGGATCTTATCAGAGATTGGTGGACAACAAAGC
TTTGTTTTACAGGACAGTGGTACAGAGTTTCAGTGGGGTCCACACAGTCCAAGCAGGACCTTCTGATAGTGTTCCTCA
GTGTCTCTCAGTATGTGTTCCTGTACACTAATGTCATACATGGCCCTGTATTCCTCCACAATCTTCTTCAGGTCGACC
TCAGAGCGACCCACTATCACCCTGGTCAGCGTATCCTCACATGTGCCGTGACCCTTCATGGCCAGATGGAGCTTCTCA
GCAAAGAAAGCTGGTATGTTCCAGGCACATTTCACAATGTCAATGAGACAGTCTTCGATGTCTCCTTTCAGCTCCATG
TTCAGGGCTTT

>Sequ00138SNP1
GTTAAATTACAGTCAGTAATTCCAGTTATTATTATATGACCAATATCTCACACTCCCCTTAATAGCTTAATATATAAC
GGAGGTTTTGAATATCAAAAGTTGCAGCGTAATGTTTGAGAAGGGGGATCAGATAGATTTCTTACATTACAAGTCTAT
GTATGTGCAATATTTTAACCTCACATAATAAGGTTATACACGACAAGCTGGGGTTAGCTGACTGACCCTTAGCTCTAC
TCTTGGCTTCTATTGTATTCATGTATGCAAAGCCGGACTGAAACCAGAACTCTCCTGTTTGAGTAATTACACGAGTGT
GGGGTTACGGGGCCACGCTGATATATTAGGGGCCTATCAGTGTTTGGCCTGCTGTTGAAACTTCTTAATGCAAACCAT
TTATTCCTAATTAAACTAGGTCGAGGCACCAACAGGCCGTGTAATTGCATCCCCCTCAATGTGCATCAGTTAACACCC
GTTTGAAGGACTATTCGCACACAGTAGTCGTGGAGAGCATTTTCTTGGGTTTTGGTTTTAGTTTGCTTGAGTTGATCA
AATGTCTGTATATTAATATCTCTTTTGCAGTTCTTATTCCTTTTTGCTGAATTTTGTCTGTCAATTAAAATCTAATAA
ATGTGTCATTTA

>Sequ00166SNP1
TTTAAATTGTGGATGAATGCACTTCTTAAACTGCAGGGATGACGTGATTTTCCTCGGTAGGTCGTCCTGACAACACGA
GCACTTCCAGCTTTGTGAAACATTTCCATTAACAAGTCAGAGTAATTTAATTTGTCGGTGCAGTGTTTAATGGCGTGG
TTGTTTATTCTAAGGATAGTATGAAAGCTTTGATGCGGTCTTAGAAGCCGCAGTGTAGTCGTAGATCCTTTTCTCACA
TGCTTGTAGCTACCTGCTTATTATTTCAATGAACACCTGTGTTTGCTGTCAACGCGCTCTTCGTCAAGTCTTCACTCC
GTCTGACAGTGCGGATGTGTGTCTGTAGTCGTCTTTGAGCGCTGACTGTGTGATGTCGATGAGTGTGTGTCGCAGAGT
CTTGTCTCCAAGCCAGTTTGCTGTGATGTTTCCAGTTTTGTAGTTGATGTTCCACCTTAGCTGATAGACAGGATGCAG
CGGAATCAGCCAAGAGGTTCATTCTGGTTCAGGAAA

>Sequ00208SNP1
CAGTTAATATGCTTCATCAGGACTGTGTGGGTTGTGGGTTGATCAGATGTGACTGACAGTAACCAAACAACCCTGCTT
AGTACATAACATAACTTTCTCCTGTTGAGCCCCACCCACTTGAAACCATGACACATAGACACAAACACAGAAATCTCA
TCATGTGAAAAATAACCAAGATGGTGTAGATTTAAGCAGAACACTTTATCTTCCTGTAGGACAACATTACTCATAGTA
AGAAGGTTGTTAGGGCCTTTCAGTTTACGGTGGGTAGAGAAGCCGTATCACTGACATCAGTGCTGTCTCACTTGGGTG
TGTTTATTTGGACAGTCAGCAGATTTAATATGACGACGCATGGCAGGAGAACTGGCATTACAAGAACGACAACTCCAC
CCTGCTCTGAGGTTTTGTGACCTTTGGTTCTCTTTGAATACTCTGCAAACTCAGGTGCCACTCCCATCTCATCACAGT
CTGCCAGGAGGGCCAACAAAGGCTTTTTTTCTTTTAATGTCTTTGTTTCCAGGACAAACAGACCCACAAGTATCATGA
ATGCCATGCATGTTTACAATTAAGGTAACTGTACAGCCCAGACTTTATCTGTACCTGATGACCAGATCAGGAACAGTT
CAGCACCAGACCGAGAAACTCTAGACCAGACGTAGAGGAACACAAAAGAACAAACAGAAAGTTGATCCCTTCATTGAT
AATGAAAATACAAAGGTTAGAAAAA

>Sequ00274SNP1
TCTTACTTTTCTAGATCAGGGGAATGTGGCAATAATTCTCACCAACACTCTGTGCTGTTACCTGTGTGGGATATATGA
CAATCAGCCCTTGGCAGTGAGTTCTCTAAGGTGAATCTCATGTTGTATCTAGCTACGTTTGTATAATGAAGCACAAGT
AAACACAAGGTCCCAGTGCACTCCAAATTCATACCCCTCATTAGCAGAATAATCCTTCTCAACTCCGATTAATCCTCA
CTGTTCAACGTGTTCTGTGCCCAAATGAGGAAAACATAAAATAGAGCAAACTCTGCTGTGTTTACTGTGGGTGCAGAA
TTTCTTTCACACTTTCTTGATCCAAGAGCAAAAACAGGTTGAGCATGTTCAGTATGATATCCCACCATGTTCAGTTAT

CTATATAAGACAACTACCCCACATCCATTAACCAATAAGATAGAGTTTGCCAAATGTGAAATGGTAAT

>Sequ00361SNP1
ATGTTATCTTTTTTTATTGGAGTGACGGTCAGACTGCAGTCCAGAGACATTCAGCTAATAGACAGTGACACACATCTC
TGTACACGGCGTTTTTACACTGAAGAGCTCAAACACCTTCAGAACAGGAAGAAACAACTCAGAAGAAACAAGTTAAAG
GGGAAGCACACGACAAAATCAATGTTTACTTGTTTGTTAACTTGGGTTAGGTTTTTTTTTCTTCTTTCATCATCTGCA
TTAATGGGGGAGATGTTGTAATATTGACCTCATAGTTGATGTCACTGGCGTTACTACATGTCATAACCTTTTATCTCA
GTCTAACAAAACGACGGCGTGATCACACGAAACTTTGATTTTGGTGCCGACGGCCCCTTTAATAGTGGTCAAACAAAG
TATCTCCAACAAAGAGTGCATTCTAGATCATAAATAATCATCATTAATATTATTTTTTATTTTCAGTAGTACAGGACA
AACCAGACCCTGGACATCATCACACACAGGTTCCATTTACTTCAATAACATACTGAATTTTACAATCAACCAATACCA
CGGTAGTGTACATGGGCCGGAGCACGAAACACAGAAAATGCTTGTTTTATTATTTTTATCTACCACACTTGACTGAAG
AGACCGAAGCACTGAGAACAATAATACAACCTGACCTGACAGCACCCC

>Sequ00492SNP1
CTCTGCTTCAACTGCCCCAATAAGGATGAAGGATCCTACACTGCAAGGTACAAAGCTGCCAGGTCCGAGGCCTCAGCG
CCAGCATCCGGGAAAGACTATGTGTGAAACTAGAGATCAGTATATGGCTTCCACAAAGCACTATTTACCGGAACAGAT
GCACTGGTGCTACTGAGTGTTGATAATTCAGGATGTCGCCTGATCAAAAAGTTACTCCTCCTGAGTGTTGGAAGGAAA
TGGATAGCAGGACAGGAACCGCACGCAAAGAGGAAATCCAATTCAATATGTTAACACATTTGTTAGTATGTGTATGGC
TGTTAATATGACTGTTCGTAAATTCAAAGTCTTTTCTTATTAGTATACACACTCTATAACCTCAGTCTTATTTTGAAA
ATCCTCTCTTTTCTTGACATGTATATGTGGGTTGTTTTTATAAACCATGTCTATGAGATTCACGATGGGCTGGTTAAA
CTGTAAATAAATATTTGTTTA

>Sequ00561SNP1
AACACATCCTACTCCTGTTAGTAAAGCATCATCACAATCAACATTTTCTCTTGAACTATGTTTGACATTCACATAGAG
GCACTGTCCAGGAATTAGCCAGATCCTGCAACATTCTGATCTCTATGGATCTGAATGTAGTGGATGAAAGAAAGATCA
TCATAGCTGTAGCTTATGATGGTGGTAAGATCGATGTAGGGACCATCTCATACACCTGTGTGTACAGTGTGTTCAGGG
CTTTTGTCTGGGCAGGACTGGATATGGAGTATGCAGTACAGCACAAAGTCCCATCACATTGTCTGGAATAGGTTCACT
GTAACTGCATCAAGAGGGCTGACTGTAAGGAGATGAGGTGGTTTAATGATTCTGTCACTTAATGGAGAAATCTTCTGT
ATTTTAAAATTGTAATTTTAGTCACTGAGATAGTTGGCACTCACTTTCATCCTAATTCTGGTGCCATTTCTTGTTCAA
ATCTCCTGATCAAAAGGATTGGTGTAAAGATGTAAAACTTTGATTTAACATTTCAGCTTGGAGCGCGCGCGCAC

>Sequ00611SNP1
TACAATTGGCTTGGCCTTGCCTTGACAAAATGCCATAATATGGAGTTCCAGTTTAGGTCTAAGGCAAACTTGAGATGC
ACTCACTGTGAACTTCCAACAGAGAACAAAAAACTCTTAAAAAGAACTAATAGGTCTAAGTCTCATAATAAAAATAAC
AAAAATGAATAGCTGCCCTGAGGGAGAAGTACGAGTTAACCTTAACCGTTCTTGTTGCACAACGGTCGTTTGTAAGCA
CTTTGGATCTTCAGACAGGCTGGTGTCTCAAGTATTGCTGGGTTGAGACGACCATTCTCCTCCTCGTTCGACTCGACG
TTCGACCACAGCCGGCCGTAACACTGGAAGGCTTGGGCTGACTTCATCCACCTCAAGCAAATTCATGACATCACAAAC

>Sequ00662SNP1
CTCCCCTTGTGGATTAGCTGAGTGTCAATGGAGAAAGGGCTAATCATATCGTCTGTGGGTTTACCTCTGCAGCCCAGA
CAACAGTTGTTAATCTCTTCAAGGTAATAATTTGACATAACCTGGATCTTTACCCACAGAGTATTATCAGCGCAGGGG
CCTGTAATTATTTTACTGCGCATATCACGCTCAGATTCGCTTGTATAGATCCGTGTTACGCTGCAGCAGGAAAGATGG
CCTACATTCAGAAGCCTAAGCTTGTGTATCTTTGAAGATCCATCGAACTAATCTGTGAATCAGTTAATCTCGCGGAGA
GAAAACACTGATGACTAATAGTGTCAGGCTGCAGACGTCACCGCATCAGAACCGCATCCAGCTCCTCGTAC

>Sequ00695SNP1
TTTTATTTTTCTGGTTTTGATTGTGTCCTTTTCTTTAACAGAGGGTCGATGAAAACAATGAGCTCACAGTTGTGTCTG
ATATTAACCTTGACCATTTCATTATTGCTACATCTGAAAATCTGTTCTTGACGAAGCAACTCAATGTAATTCACAGAA
GCGAGCAAATTATCATCCTGTACAAACGCTAAGTAACATAACACCGGAGATGATAGAGAAAAGACGCGTCACAGTCGG
GTGAGAAGGAACATATTATGGACTGAACAACATGCCTTGATGCTAATAAAGCTGTGTTTGAACCTGGTAAATGTTGTT
TCATTTATACAGGTAAAAGGCTGAAGGTTCACATCTGGGTTAGCTTTTCTCAGTCCCCGCTCTCAGC

>Sequ00752SNP1
GGCGTTTTCACATGTTCCTGGACTGGCGCAATATATATATATTTGACTATTTTTAAAGGAAGAGCTATGAAGAATATTTG
ACATTACTGTAACATATGACCATTTGGGAAATGGTTGAGGAGACACCAGTGGAGAGGGGCTGTGTATGTTGTGGCATG
GAACAGTTGCCTCGTTGATCCCTCCAGGAAGTACAGCTACAATTGAGATTACATCGGTCTTCACATACACTTGGAATG
AACCCCTTCTAACCTACGAGCTTGTACATGATATTAATAATGGTTATACTTATCATTGTACAATTCCGACTTTGCTAA
CTGAGCTTTGCCACTATGTTTTTCTTTTGGATCTTACATTGTATATAACTGACTCATGAAA

>Sequ00790EST3
AGTGATCGTTAACCTGATGGAGAGCGGTAGCCGGTCGTGGAAATGTGTAAACGCTCACACTCAGAATGTCACATTACA
GATTGTGGGGAACAGAATAAAGAAAATTCACGTGGCAGCATCTCCAGTGTCCGGTATCTCATATTGCACCTGAACACA
ACGCAGAATTGTCGGGACTTCAAGGCTGTTCCGGCTACACAAGGGTGACGTAGTACGTAGAAGTGGCGTTGTCTCTAT
GGCTGCGGAGGACGCGGAGACTGATACCAGATTACGCACTGCCATTCCAAAACTGCGCTGTTAAGGTAAGATTGCACG
ATGTATCACAAAGAAAAGAGCATCCAGATCAAAAACAGCGCCTCGGCGCTATACAACAACCTCGGTGTCCTGCGCATC
GCCCCGCGGCGCCTCACCTACTTCACGGTGGTCCATGCTAACGTGGTCAACATGGTCAGCGCGTCCTGGGACGGCCTC
AACTACTCCCACCGTCAACTGCAGTCAAAAGAGCCCAATGTTGCTACAAGCACTTCGCTGATCATGCAGGCTGCATTT
TGTGCTCTGCCCTCTCGTGATCTGCTGGTCGTGACCTCTCAGAAAGGCATCCAGATGTATGAATCAGATGGCTCCATC
ATGGTATACTGGCATGCTCTGGATACTCCAGAAACACCTACAGCTCAGGCCGTGTTTGCTCGAGGGATATCAGCTGTG
TGGGAGAATTATATATGTGTGGGCGTTTCATCAGGTGCAATTTTCGTATTTGATATTCCCAGTAAAGGCAGTAATATA

ACCCTGTCCGAGGTCCTGGAGGAGCACAAGCAGTCTATCACTGACATGGCCTCTGAGTGCTCTGGTAGCCAGGAGTGC
ATAGCTGATCTGGTCAGTGCAGATGATGGGGGCAACCTTTGTGTCTGGAAGTCGGGGGAGGAATTTCAGCTGCTTAAC
AAGATCCCTGGCTTTGATATGAGCTGCTCATCTGTTAAGTTGTGGAAAGGTACAGTGGTTGCAGGCTATGGCACAGGC
CAGATCCGTCTCTATGAGGCAGTGACGGGAATTCTGCATGCTGAGGTCAACGCCCATGCTCGCTGGATATACTCATTA
GACATTGCTCCTTTTTCTGGACTGCTTCTGTCTGCTGCCGAGGATTCTCTAGTCAGGGTGTGGCATCTGACTTTGACC
CCAGAGACAAACAGTGTGGAGGTCGCCCATTTGCACAATGAGTGTGTGACAGACACACAAATCTGTGGCGCCAAGTTC
TGTGATGGTGATGGCTATGCCTTTGCAGTGACAGGT

>Sequ00889SNP1
AACTCATGAGAACATTATTTACAATACTGGGCTGGCAATGGGATCATTTGGTTTCCCTGCTGGGGCTGAATTACAGCA
CTAATCAGGTCTCAAACACCCCTGACATTTCAGAAGCTAGCTCTGGACTTTTCCTTCGACAGAGTTACACAAATTTTT
TCAACAGTTTTAAGTTCACTTAAATGAAATAATGGATCTGTTGAGGGAAAACAAATTACTGGAATAATGAAAGTGCTG
ACAACCTCAAACTACAGCTTTTATTTTGGTACGAGAGCAGGCGAAGCTCTGAACAGATTTCTTACCACAGAATCACTC
CTGTTCATTTCCCACTTTGTATAAATTCAGTTTACAAAAGCTTTTTTT

>Sequ00914SNP2
GGAAGTTCACTGACATATCGAAGCTCTGCGATGGCAGTTGGACTGGATTGGATTTGGCTATGCTGCAGTCCATTGCTT
TTGGAGGATTCATGGGATACAAGNGAAAAGCCAGCGTGATGTCCCTGATGGCTGGTTAGTTTTTGGTGGATTATCTGC
TTATGGTGCCTATAACATCTCTAATGACCCAAAGGACATAAAGGTCTCATTGTTTGCATCAGGAGTCCTCTCAGTAGT
GATGGGGAAGAGATACAAAAAAATCTGGAAAAATATTGCCTGCTGGCATTATGTCGGGGCTAAGTTTGTTGATGGTGT
TTCGACTGTTACTCCTGATCATGGTGTGATTGGAGGAACAACACTGACCAAACTTCAATGAACAAGCCTTATTTTAAA
GGAAACATCATTCCCAGTTAGCACAGTAAAACAAATGTTATTTTTGTATGGTAATATGAGGTTCATGTCACCAGAAAC
CTTGCAAAAGGTTTCACAGATTTAAGATAACTCTTATTGCAGTAAATCCTTTTGGTAATCTTGTATAACTTTTTTTGT
ACTAGCTTGAACATAAACCTTATGATTAATTTTTAATCTTGTGTTATTACCCAAGATTTATTCCCTCATAGTACGGGC
AGCACAACATATGTACATGAGCTTTGTGAGTGCTATTGTTTGTCATGGCAGTGTGTTTACTGTATACACTACAACATC
AGTTTTGCAGGCTCTTAAAATATCAAGGTTTATAAACTGAAATATTCAACTTCCTGAAAAAAGGACAAGATTGAAGGA
TAAAATTGGATTCTTGACTCCTNACATGCTACGGGAAAGGAATTAAAGTTAATATGTTTGCGTTTTTTGAATAACAGC
AAAACAAATAATTGATGTG

>Sequ00915SNP1
CTTGAACTGAAGTGCGGTTCTGATGCACAGGCCAGTCTCCAGCTCGCTATTTTAGAATCCCTCCCACGGTCGGTGTGC
AAGAAGCCAAATGAGCGACTGTTGGGAGACAGACGCGGAATGTGCTACAGTATGTCAGTTTTACTGCACCTCCTCATA
TGAGGAGAAATTGCACTGAACACAAGCCAGGATACCTACACGGTGCCACACAGTGGACGCACACTGCAACAGCAACTC
CACTCCACACCACACATGAATGTGATGACCAATGTGAATGGAATGTCTTTGTATGATTTCTATGTCGAATCTCTACAT
TTTGATTGATTGATTGATGTCGGCCAAGTGTTACTGTAAAAACTT

>Sequ01016SNP1
CCATATCAACTGTATTTATTTGTTTACAGTGAGTGCCACTTCATCAATGCTCAATCATATATATATCTTTATATTAGT
AAGTAAATACAATGTCAATGTCTCAGTCATAAGCTAGTGAACAGCATTATGGGTCAACAATGACATCAAGCAACAACA
GTGGAAAATGATGATTGTAGTCAGAGACAACATGAAGTGAAGCCTAAAACAGACAGTTAAAACACCTAATCCTTTTGC
ACAAGGAAGGTTTTTACATTTCACTTTCAAGCCATTTCAGACATGAAGTAATCACAGAAGAGATACAGGTTTTGATAT
CAATTCCAGACAGTAAGTGATCAGTTGTAGAAACTACCAAACATTTGGAAACCACACTTTTTCCAATGGTAATAACAG
CTGGGTGCAAACATGTACCTGTATGCAACCAGTGGTTCATCTGTGCTACACAGCAGTCACAGAAACTACTAAGTGTCG
TAGGATGTTAAGCGAGAAATGTCGCTGCTGAATCTGAAAACATGACTCGTGATTCAGACAGCTCAAGTAGTTTTATCT
GTTCATTTTAGGCTCAGTGTTGCTGCAGAGATGAGAAAGTGACAGACAGACAGACAGGTGGTCAGTGGACCTTGAAGT
GGACTGTGGATGGTTAGAGCACAGAGCGATCCCTTTTTTAAGTCTGCTTTATGGAAATGAGG

>Sequ01024SNP2
ATTATAAATCCATGCTGACCGTCAACCTGTGTGGAGTCATAGATGTGACACTCAGCGTCCTTCCTCTCATCAAGAAGG
CCAAAGGGAGAGTGGTGAACGTTGCCAGCGTGTTTGGGCGAATCAGCCCGTTCGGCGGACCCTACTGTGTGTCCAAGT
ACGGAGTGGAGTCCTTCAATGACAGTCTGCGTTTAAACATGGCACCGTTTGGAGTCAAGGTCGCGTGCATTGAGCCAG
GATTCTTCAAAACCAATGTGACTGATACAGTGATACTGAAGAATAACCTGCAGAGGCTGTGGGACAGATTACCTCAGG
ATGTGAAGGACGACTATGGAAAAGCTTTCTTGGACCAAGGTATGAAGGGGTTGGATGACAGGTTCAAGGTGTTCACGG
ACTCGGACCTGATGAAGGTGGTTGGCTGTATGGAGCACGCTGTCTCTGCCGTTCACCCTCGTACTCGCTACTCTCCTG
GATGGGACGCAAAGTTCCTCTGGTTGCCTCTTTCATACATGCCAACCTGCATCTCAGATAAACTTTTCCTTAAAAGTA
ACCCCAAACCCCAAATTTCTGTCCTGTAGTTTCTGTAAAAGTAGTAATTTTGTGGAACAGGTTTACACAAAGAGAACT
GACATTTCTTTAATTTTTTACACACAATAAATGTTTGCAAGTTAAGTATTCCACCATTGATAACATCATATAAACATA
TTCTGTATTATAAAACATGTTTGGTTCGTCATTATGTTTTTTGCTTGACTCTGGACATTAGCATGCCTGCTGTACAGA
TTATAACATCTCAGTTAATGGCCTCTGATCACAGACGATGGCATTTGCTTCTTGAGACATCTTGAACACAGTGTCACA
CCTCTGTCAACTTGAAAAGCTTTTGTTCTTGATGGACTTTAGCTCCGCTGCGTCTGTTTGAACTGAAAGTTTGATCAT
TAACGTTCATTTGTTCTCAATATCAAATACACAACAGCGTTTATCTGTAACCTTTGGCAGAATTGTTGTGTTTTCAGT
AGTTCAGTCAC

>Sequ01071SNP1
GTTTGTGTGTCCGCACAGTCTGTCAGTCCGTAGTTGTCTCATATTTCACTTGTCTTGGCTCAGATATCAGCTTTAACT
TATACTGTGTTAATCCCAATGGAGATGTAAAACTTGTACTTAACATTTCACTCATACTGACTGTGTAGTTACAGCCGC
TTGGGACCAAATTCAGTTGTAAGGCTAGAGCTTCTCTCAAGGGCAGTGGCATCAGGCACATTCCTAATACCTGGGAGT
CATGTGGAAAGCAGAGCAAACCAGAACACCTGGATGTTATTATTATGTCCCGACTCTGGAAAACTATCTTGTATTTGT
CCTTCCAGCCTACAATAAATATGCTTTTCAAT

>Sequ01113SNP1
GTTCGTTCAGAACATTTTAAAGTCCCGTGTTTAAACAGTATTATTGATGATCTCATTGTTTTAATTTAGGGTTTTTGC
CACTTTTGAAATGTCAATGTCACACACAGGAATGAATGTTAATATATGAAGCAGGATGTTTGAGAGAGGGAGTAGAGA
TGTACAGGTCCTCGCGTCAGCAGTGACGGGGCTGCAGGGAAAATAAATTATTGGCAGATTTTCGAAATCTAGTTTAAA
TAAAGGTGAGACGTCTACGTCTGAAATCAGCAACTAGAATGAAAGTTTTCTTCACTCGGTGTCTGAGTATCTGCTGAG
CCCTCACTGCCACAGTAACCACGACAACC

>Sequ01215SNP1
AAAGAGTGGCACTATTGCAGATAAATATGGGCCTCTCCTCTGTGTGGCCTACATATGTAGCATGCGGTTGCCATGTTA
GGATATTTCTTGCCTGGAAAAATGAATTATTCAGTGTCTTCTAGCAGTGGATGGACATGCATAACACTTAATGCGGTA
CTTGATATTGCTAGGGTTGAATCACGGGTAAAGCAGTCTGGCCTGGTATGTGTGTGTATAACTTTGCCGAACAGTTGG
ATTGGTTGCTGCTGAACTATGACCTTGAAGCAAAGCTCCACCTCCTTTCATTATCAATCACAAAGTTAGCTGTTACGA
TTAATGCCCAATAATTTGTCTGTTAAACAAGAGTCCCATCAATCATGCTGTTCAGACCACATACAAACGCAGTGTTTC
GCACAGACACTGAGCCTTATGATACAAATATGAAGTGTCAGTAATCAATTCAAAATTCAACCCTTGCTTTT

>Sequ01379SNP1
CTTCCCCAGTGCCTATTTATTGCTTTGTTTATTTCTGGTCCAACTTTTGCTGAGCGAATATTCCTTATCAAAAAGGTA
TTATATTAGATTTAAGTTTGACTAAATCAGGTCACTTTAATTCTGTTTGCATACATGTGTACGTCTGTATTTCTTTTG
CTTTCTTATCCTCGTGTTGCTTTGTTAATATGGAGCCTGAGTTCCCATTATCTAGTGGACTTTCGCCTTCCTCTCAAT
ACTCTGATGCGCAGGAGTATACTTTCATGTGATGAACATGCTGTACAGGACATGAATGTACAGTAAGCTTAATAAAAT
CTTGATACAAC

>Sequ01444SNP1
AAATACTCTTTATTTCATTCAATCCATGATCCCTTGAGCCTGAACAAGAGCTACTGATCAGACAGTTTGGTGAAGCTA
CACAAACCACAGATCAGCAGCTCTGTCAGTGGCCTGTTTGTATCTACTGCTCATCTAATGAATCCCTCTACAGTGTAT
CTGTAATACATACACATGGAAAGATTGTATGACCCACCAACCCGCATCAAAAATACACATGAAAAAAATTATCGTCAT
CTTCTCATCACTGTCTGTAAACAGTTTCTGAAAAATCACACAGTTCTGTTTTTTTAATAAATTACAAAGTAAAATACT
ATTACCCACCTGAAATGGTTAAAACGTATTTTCCTCCCAGAAAATATGAAGACAAAGATGACGGCATTAACTCTTTGA
TTTCATTCTTTTAATTAGGCATCTTCTTGTTTAAGTGTTTTACTTAATGATGTTAAATAAAACTCAAATAAAACACAC
TCTCACACAAACACCACACACATCTTCTTTTAGGTTGCATTGTTTTTAGAGTCAGTTTCTGTTTGGCAGTTGAAGTAG
TGACTCTGTTGCAGAGACAGAGCATGAATGACGAGCAGTGAGGAATAAATTACAC

>Sequ01484SNP1
TCTCTTTGTTCTTCTGTTACGCCTCAAAGGTTGTCTGTCCTACTCCATGTAATACATAGGGCTTTCAATATTCCTTCT
GCAAAAATGATACACCAATAGTTTGATGTCACCTTTCAATTTTAACCTCCAGCAACCTTGATGATGTCATTCAGAAGA
AAACAGCTGATGCAATTGCAGGGATGTGTAAATAGCAGTATACTGTGTGTAATTACCTTTGTACAGGCCACCATGACC
CAGTGGCATTGTAACTGTCGGGTTGGGGATGGTATAGAAGAGGCTGTGGCAATGGGGATTTCACTTTATGTATTGTGT
ACTGATAGCTTGTACAGTTCTTTAACACACTTTATTTACAACAGTATTTGTGTATATTTTATGAAGTAATAAAAATGA
ATAACGTT

>Sequ01546SNP1
CTTGTTTGTAGTTGTTGGTTCCTCGGTGACCCCGCATCATTTTGGACTTGAAATTCAATCGAGCCTAGAACCAGACTC
CATGATGTTTTGGGAATTGATAACTTATTTTCCTGCGTCATTGTCCCATAATTTGTCTCTATTGTGCCATTAGGTTAA
AAAACTCAGTAAAGATTTTGATTGGGTGGAACTGCAAAAATGTTTTTTTGTTCCCTGTTTCCTTATGAAAATGTGGCA
CAAGGTGTTTCAGCAACCTGTGTTCATCACTCTGTCCTCGACTGAAATACATGCAGGGGCTGTAAAACTCAATAAATT
G

>Sequ01628SNP2
CTTCAACATCAGACTGTGGAAAAATCAAAGCCTTAAAATGTGAAAAACAGCAAAACCNCTAAAAAGTCAGACACTGGA
GACCNGAAAATCCCACTGCAGACAGGCTCTCATCTACAAAAAAGTGTGCAAACCTACTGACAGTATTTCCATTGTCTG
TGCCTTCCAACTGAAACTCCTGTCTGAGTTTCATCAAACATTCACAAGCCTGAGATGCTCTGACAATCGCTCACACAC
CATTACTGGCTGCTAAGCTGGTGTGGCATCACACACTTTCCCTCGGCCAAGCCACTAAACCGGTGCCTAACCTTTGAC
CCTTGAATGTTCCTCAGTATTCAGTTAAACATAGTGGTAGATTTAGGTCAGGCTTCTGTGGCATCCAAATTTCCAATT
GTGTCTTGTACAGATGTGTGGACACAAAGTGAAATGTAAATCCAGTTAATAATGTAGATAGTTCAATCTTATTCACTA
ATATTCTTCATCAATATAATAGTTGAGTATAAAACAAGCCGTGTGCTTAATAAAGCTAGTAAATCAGAATGGGTGGCC
AGCTGTACTAAATGAATCTTCATAGGAATCACATTTGATTTCGACCTTTTGTACTATTTAATGCGAAAACATTGTGAC
TGTAAGTGGGCTTTATTTATTTGGGGTTGTGTTTCAGCACTTCAACTTGTCATGGTACCTGAAAGTCATGACTCTGAC
CACTATCTGCCTTTTGTATTGAGGACCATTGTTGAATAAAATAACTATGCAAAGAGGGTATAGTTATGCTTAAAGAAA
ATGTTTGATAAAGGTGAATAAAGTCTTTTTCAT

>Sequ01745SNP1
AAAACACTCGTATAATTACATAACAGCTTTTTACATTCTGAGTTCAGCAGAGGTCAGTAAATGATTATGTACAGTCAG
ACGCCCCCTCAGTGAACAGTAAGGCAGCAGCTCGGCCTGATGGGTAAAGGTCCGGTGAGTAAACAAATGAAAGAAGAA
GAGTTGGAATGAATTCTCCTGCATCAGCGCCACAACCTACAGTCTGACACCAAAACAACGTGGTCTCAGCATGGACTG
AACTTTATGGCGTTCACTCCCTGAACTCGTCCTCAGGTGGGTCATATAAGGCTGAACACGGTCCCTCAGT

>Sequ01822SNP1
TGGGAAGAGACAGAAAGAGAAAGAAATGCCACTCAAGAAAAACTGCAGCTTCTTAAAATGCAGGCTGAAATATCCCAA
CAGAGGGCAACTCTGGACAGAAGTAGCAGTAAAAAGGATATTTCACAACACAGAGATCAAACTGAGCGCACCAAACAA
AAGTGGACAAAAATCCAAAGAGATACTGAAATGGAGAGAACTGCAGTCTCAGAAGCGGAAAGGAGAGAAAGACAGAAA

AAAGGAACAGACACCTTTGAAAATGTGAAGGTAAAAGTCAGAAGAATCTTGGAAGAGATGAAGAAACTTTGGGATTTG
CTGGAAGACAGTAAACAACAACTGGAAATTACTCTTAGAGAGAAGCAAGAGCTAAACACAGAGAGCGGTCAAATCGAA
AACATGAAATCTGACTCTCAAAAGCAACAACAAGACATGAAGGCTGATGTGGAGAAGCAAAAACAGCAACGGCATAAA
CAGGAGCTGGACAACGAGTTACAAATTAC

>Sequ01844SNP1
CCGCTGTAACTCTAACGTAACATATCTGAACTGAGGGGAAGTGTGTGTGAGTGTTAATGTCACGTCAGCCAGCGAGTG
TAAACGATGGCATGAGGATCATACAACACATCAGACAGCGGCTGTGAAATTCTCTAAAACACATCGTTTACCTGCGCT
GTGCTGACCACGCGCTGCTGACGTGACATTTCCTTTTCCATTTCCCCGTCTTTCTCTCCCTCCTTCTCCTTGTCCGCC
GCTCTCTCGTCCTCCTCATCGATGCCGCTCTCTCGCTCCAGAACACGAAACTCCCAGTCCTCAAAG

>Sequ01869SNP1
ATCATGTTTTCTTCTTTAATTACCCTTCTCAAAAAAATATGTCATCTCAGAGAGAGGGCGGAGAGCAGGAGGCTTTGC
TCAATGTAAGATTTTTTAAAACTTCTTTCTCCCTCATCACCACCTTCTGTTGAACATCTCCTGAAGGCAGGTATGTTA
TATGTATGCTGCTTACATAAGTTCAGTGCAATACCCGCCTCACTTCTGGTTTTTAAAGGCACTTGAAAAGAAATGAGG
GACAGTCCAAACATAGTTTCTAAGATTTGAGCAGAGTCTTCTACTCTCACCCTCTCTAGGCAGTGTTTACGGGGGATG
GAAAGAGCAGGGAGAAGGATGAGTGAAGGGGGAGGAAGTTAAGATTTGAAAGAGGAAAAGATGATAAGAACAAAGCAT
ATAGAAGGACAGTGGAAGGGTTTAAATTGCTCTCTCCAAACAGGAGAAATAGGGGGAGCTGGGAAGAAGGGATGAGAG
GAGGAGGTGAAGAGAAGCA

>Sequ01888SNP1
GTTGGTACACTGATCATTGTAAGCCTTATCGACACGACACAGTAGTTGCTCCCTAAGAGCAAACTTTCAGGCGCTGAC
CAGGAAGGAGATTGTTCACCTTAGCGAGCATGTGTGAAAACATGAGGCCTCCATTCACCAAACAAAGGAAAGAAAAAC
AAACCTGTACAAATACTGAATAAATGAATAATACAAGAGTAGTTGTAAACATGTACATATTCACAAATCCATAATCAT
TTAACACTTTCCCCCAATTTTAGTCCAACAATATAAGTCATTTCCACACTTCAGACAGCGTGT

>Sequ01964SNP1
GTGTGTCGCAGGTTGTTTACAATGAACCCGAAGAAGACGTTTGTGTTTTTGTGTCGTATAATCTGATTTTAGGATATC
TGCTGAACAGCGTGAAGCCAAAACTCTCGTATACCTGTTCTGACTGTTACTGTATTCCCTTCATGCTAGTGTTAATGT
ATACAGATCATGTCTTTTTACTACTGAGTGAATGGTGGCAGCAGGTCGTGATTGCTGTTGACTGCACCAGCAGCAGTT
TGCTATCTTGTCATATGATGGTATTTTATCATGTGGTTGTTAATAAAAGCCAAGGGCACA

>Sequ01991SNP1
ACTTTTCAAAGTCATATTTATTGTTACATGTAAAGCACTTAAAGAGTAGACATGAAAATGAGACCAAATACAATTTGG
CCACTGTCATAGAATATCATGAACCTACCTGGAATGAAGTAACTGTTTATATTTGGTAATGTTGAATACAAAAGGGAA
AAAAAGGAAAACCTCAAAGCGCAAAGGACAAGTCATCTCTATGACAAGAAGAAGGCACAGCAGAAGGCAGGAGAGTCC
AGTCAGCCTGCTCCTCATCCAACGTAACAGACGAAAATAAAAAAGACCAAACACCAGACACCTCAATAACAGCATTTA
CCTCATGTGATAATCCCGAGAAACAAACAAATGACGAGTACAAGCATACAGACAGCACATCAGACTGGTCATTAAAAT
AATTTGACCACATGGCCTCTGTATGCAGATATGTAGAAGTTTATGGCGCCAGTTATTAAGACATGCCTGTACTTCATT

>Sequ02007SNP1
CTATCATAGAAATCCATCATACAGTCTCCAATGCAGCAGTCACTGCAACTTACTCATTCACAGTTTAACATTATGGAT
CTGAATTAGCATTGGCTTATTGCAACACAACACTTTCTTTCCACTTAACTGGTTGTTAGGAGAAGGAAAATCTACATT
TTACAAATAATCCCCCTCAGCCAGAAATATTGTACACGCAGTGCAAACGTGGGATTTCTGTAATGGAGACACCTTTGT
TGTTAAAGGTGGTAGTTTACAGATCCAAATTGTGCTTGTAGGCGGATGCTGTTTTTA

>Sequ02010SNP1
GCATCACACCAAGTAACTACTGTTAGTAAGTTCAAATATAGCACTTTGGCAAACAAAACCACAGAGTATAATTGTGCA
GGCGAGAAACGAGATATGTAAGTGTTGAATCTCTATAGTTGTAGTGCCACCAGGATGTGTCATTTCCTATCCTAAAAG
GCCTGCTCCCTGGAGTTTGTGCCTTGCTCTTCAGTCTGCACTTCCTGTGTGGCATATACTCAACTTCTCTACTACGGT
ACAGTCACCCACCCACATAACAATCCATCTATTTCCAGGGTACTCACGGGACATTTAC

>Sequ02141SNP1
CTCAAAAGCACTTCAGTATAATAAAGGCAAATGCGGATTTAAAACATGAATACAGCAGCTCTGTTTATTTTTATCCTG
GAGACTTCTGAGGCTGGTGGCAGTGTACCATCTGCAGCCAACCTGTCTGACATTATGTTCATCACAGCAGCTGAGAAA
ATATCCCTACTGCCACCATCCAGGCTGAATGTAAACATCATGATACTAAAAAATTGCATAAAAGATTCACTTTGGAAT
GCATCAAATTTTCAGCTCAGGGCAGATTCATCAGGACATCTCTGAGGATTAAATAACTCCAGTTCATACCATTTTCAA
TAAAACATTAGAATATTATCTGTAATATACTACAGCCCTCTTCCAGCATTGTATATAGAAAATCTATGGAATGAAAAA
AGGT

>Sequ02164SNP1
GGTGACACCTCAGGAGACTACCGTAAAACGCTGTTGTTGCTTTGTGGAGGGGAAGATTAAACTTGTCACATCCCTGCA
GCTTCGTTCACAGACTTCAGAGACAAACCACGAGACAAAGAGCGATCGCAAAGTGAAACTGGTGGTTAACGTCAGTCT
CTATCATCATTGTTCCTAACTGCCGCCACTCCTCAGCCAAACCAAGTGACCTGTGACCTCCCAGCCTACCAAGCCAGT
CTTCTGCTCTCACGTCTGATCGTTCTGCCCCGTCTGAACTGAATTCTTTTTT

>Sequ02181SNP1
GCATGTTGGAGCCCTGCAAACACATCAGTCTCATCTTCACACAGACAGCAGGTGGAGCTGTGACAGAGCAGTGGAGCT
GACTTCAGGTTTATAACATGATGTTCTTGTTAAAGGCCGTAATCATTAGAGAACATCGGGGGAATATGGTGTCATTTA
TATTTAATATGTTTGAATTCTGTGTCTGAGAGATGAGTACAGATCAATGATGTAAATCAAACTCCTCCCGTCCAATCA

GTGTCCCAGACTCCATAAAGCTGCCGCAGGTCAGTTTGGTCTTCATCACATGAATAATAGAAACACAGCGTTCCTGCT
GGTGTTTTACCTGCTCAGGTGAGAACCTGTTAGAGACATTTACTCTGAACCATCAGACTCAACTAAAAGCTCATAACT
TCATCAAACAGCTTCTTCTCTCCAGTGTCTAAAATAAACCTGTACATGTC

>Sequ02185SNP1
TCCTGTTGTTGTACTTGTTGTCGTACAAGTCTGTCCCTGCACTCGTCTTTCTCTCGGCATGTTTCACGCTGTTCGGCC
GCACTCTGGGTGTCCTGTTGGGAATAGATCATTTGTTAATCGTGTTTCGTTGGGCGAAACAAGCCTTTCAGAGCAATG
AACAGTATTTACGCGCCTGAACGTTAACCCTTTAATGCTCCCCCTGGCCGCCTGGGCTATATGTAGCTTTATCACATT
ATGTAGTAGTGAATTAAATGCTGAGGATAAGCCATGACCTCCAGTCATTTA

>Sequ02241SNP1
AACAGCAGTGCATAAAAAGTTTCAACAGAAACTAAACCAAATATAAAAATTTAAATACCCAGACTATAAATATTACAC
TTAAACTGTATTGTTAGAGGTGATTGTTAAACACACCTGGCAGGTGATGAGATGTTCAGTGCTACTTGTGCAACACAG
ACCTGAGATCGCTGGAGATCATCCCTCCAAACACCACGTGGCTTCATAAACCTGTTAAAGGCCTCTGAGGCAGAGATA
GTCTGCTGGGCCTGTTACCCCACAACTCTTCTATGGTACAGGACCACCC

>Sequ02307SNP1
CAGTCATATGAAATTCAACGGATGAATTCAGTTCTTTTGTAGACCAGGAAGTACTGATGACTTGAACTTAAAACGACT
AATCCTCCAATCAGAGGGGATAAAGATATCTTGCAGGGTATCTGAAAATATTTACAAGTTTGTTTAAAGATCAAGTAA
CTTTAAAGGGCGAATTGAAAGGTGCTCAGGTGAAACCAGCACCCCCTGCTGGTCAACCAGAGCAACACATAGAGTAAT
GAATCCAGTGGACTGTATGATAGAGGGATGGGGAAAAGGAATGGGG

>Sequ02376SNP1
GTTTAATTTAAAGTGTTGCTGGTGGAGCGTCACTGTCTTCTGGTCTCATGTAGGTTTCCACAGTGTAATATCTCCCGG
ACGATCGCTCGGTGTCAGTGACGCGTTCACACACTCATTGTTTACTATTTATTTCCCCTGATGTTGTTCTATGTGCGA
CAGCAAAGGGCCAAATAACAAACAATTTCCTTCTTGATTTTAATTATGAATGAACTGGATGATGCTGTGTTTATGGTC
CATGTTTGATAATGCTCTGTATATACCTTATGATGTTTTACCT

>Sequ02382SNP1
CTCCCACTTTTGTTGTAAACCGCACAGCAGGCAAATGTTAACGGTTTCCTCTTTGCACAGAAACGTTAAATGTCTAAG
AAGTTCCATTTTTACTGTCATCTTCTTCAACGATATGTACTCCGACCACTACTGGTATCCCTTGGAAAATTGTGATTA
TTTTTTTTCCCTGCAAAATGGTGTATTTTTCAAGTTTGTGTATATAATCTTGTTATACATATTTGTAAAGTTTTGTAG
TCGGATATGTTGGCGTATAGAATAAAATATTTGTGGTTTTTTG

>Sequ02391SNP1
GTGGGATTCAAGGCAGTGCTTCTGAAAAAAAGACTGTCAGCACTTGACTTCTATAATGGCTACCTGCATCAGACGGTG
AAGAAGACACCCCATGAGACAGCTGGAGGACTGTTTGTCAGTAGACAAGGAGGAACTGTAGCACATCAGAGGAGAGAA
AAATTAGTGCTGTGATGTTTCAACAATAATTCTATTCCCTGATGTGAACACTGCAGACATGACATCACCTAATGGAAA
TGACTTTAGTTAAGTTGTTACTCTCACTACTTACTGTCTGTTT

>Sequ02423SNP1
GCTGGGGTGTGTGAGTGACGATGTGGATGTACTTGAGTGAGCGTGTGACTGTATGTGCGTGTTTAAGGGACTATGAGA
ACTTGTGAGGTTTAGAAGCGGCTTGGAAGCCGTCGTGATAGATGGCTGGCGAAGCGTCTCTCTGTGCTGACAAACGAG
AGAGACCTGCCCTCCCCGGTGAGTCTCGGTCAAATTAGCCCTATTTATTTTAAATATATATAATATGTGTGCCTGTAC
AGTTCTGTGCAACTAGAGAAATGCTCCAAGGAGTCTGTTTTTTGAAGTTTATTCAGGTAGTGATGTTATTTTTAGTCT
GATTTTCTCATTCACCATGTCCATGGTTGGTAAGCTGTAACTGAAAACATCACCAGAAAATCTGCCAATTAATTTCAG
ATCACTTCGTAGAGGAGACAGGCCACTCTGTGACATGAGGGTTGTTG

>Sequ02443SNP1
GTATTTAAGATGATATTTATTTGAGGGCTGTGCACCACGTTACATACAGTCGTATACTTATGGTGTATTATGACAAAA
CATTTGAAAGTATTGCTGAGTTAATTCAGACACACATCCTCTGTTAGCTTTCCCGATTATTTCTTTCTTAGTCTCATC
TCCATATTGTTTTATAAAACAGCTGTTTGAAATATGGACTTCACAAGGTAGGTGCTTCCAAACTGGTGCCAATTGAGT
CCAAATGACAAACCATATCTGGTAAAGAAAATACATTTATGTAGTGTTCAAAACAACACTGAAGAAGGAAGGGAGAAA
ATGTGAACATCAGTGTCCAGCTGGGTGAGATCAACACGGCATGCAAGCATCACACGAAAACACATCATGAAGTCAGAA
CTTTTCCATGGAGGCCTTTTATTTGATCCAAGGTGGTGTCAGTGTTGTTGTGCTGAACACATTAAGAGTAGACTTCAA
ACACATGTGTTTACTTTTTAGGAATCTCCAAGTCCGGCAAACTGTTTCACACCGGCCTGCTGTTCACCGCCTGCTGCG
ATGCGGCTGAGGGGAATGAGCTTCATGGTGGTCTCTTTCAGCGAGTACCAGCGGTTGTGCCATGTGACCCAAATGATG
CCGTTGTCGAAGCCGTATTCACCTGCATCCTTTTCTGTGTACCTGCCACCCTGGTAGTATTTGCCGTTCAAATGCGCA
GCATGACACCTGTTCATCCACCAGCCAGAGCCGTCCTGCTGAGCACAGCTGCCATCATACTTGTCATTGTCCTTGTCA
ACGGTACTGAACTGCATGCCGTTGTGAGATGTGTAGAACTTGTCGCTGGGGTCATCTCCAAAGTCGAAGCCATCGAAA

>Sequ02608SNP1
CACCTGAGAGCAGGTCACGCTTGCACGGCACTGACATCACTATTCACGCAGAACAGACACAGTCCTCTCCGCGGTTTA
GGCCGGAGCCTGGGACCAGTGTGGATCCAGCTCCTCTGGGCTACATCTCATTCTGCCTCACACTGTTCACCGTCTACA
TCACTGAAGTTATTGCTTTGAGAGAGAAGTGCATATATTCAGAAAGAAATCCAGACCGGAGCTTTTCTCTAACCCTCC
ACTTCATAAGTGTAATATTTCCTGTCAGAAAAAAGTCTAGCTGCTTTTTGTTTTTTGATCTACGCTGTAAGAGCC

>Sequ02708SNP1
CGGGCACGAGAGCCCAGGCAGGCACTTTACAGCTGGGTGTCAAATCGTACGTGGGGCTGATGTCTTGAGGTAACAGAA
GCACGAGCTGCATTTTAACCACAGTTCACGTCACTGCACAGCGTTCAATCCCTCTCTCCTCACCCTGGGCGGCTTGTT

CTACTTTCTCTAAACTATTTCTATGATGTACCATTTTGAACTTGCACTGAGCTCAAATAAATTAAGCACAACCCAACT
GTCTTGTTGTCGCTAACAAAGACGCAAATCCAATGCTAAACATTTCGAGAATGACGATCATTTGACAGAGCCCACTGT
AGCGTCACGGCTCAAGGTTATTTATTTCGCAGGCGTAAGTCCTGGTGTGCCG

>Sequ02728SNP1
CAGTACATAAAACATGGAAAATCATGAAAATTATGATGAATTTTACATGTCTCCAACAATATATCAGTATTATATTAG
ACATGGAAGTTATTCACAAGGCCGGGCATCAGGTAATGGTTCACAACGATCCACAACAGCTCGGACTTCTCCAACCTG
GACACCTTTGTACGTCCCAGAGATGGATGTCCATCTGGTCTCTCCATTGCTGTAGGTGCGGCTGAGGCGTGCGGTGAA
AGGGATGTCTGCGCTGTACTTATATGCCA

>Sequ02738SNP1
ATTACAATATCCATGTTGATGTACATTTCTAGGGCTATATTTCCATAAGCCCCAACAGGAGAAAAGATGACTAATCAG
GCCTCGCCTCAGTAATTTAAAACAGGCTATTTCAGCAACAAACAGCAGGTGGACAGGCCAAGAGTCAAAGGTGAAGTC
AATGGTCGGCGTCTACTTGATCTTGATGCCGATGATCCTTGCATCACCGCTGACGTCAAAGAACTTGTACTTGATGTC
CCCCAGACGGTTGGGGAAATTCATCATGG

>Sequ02745SNP1
GCAGGGCTTTGCAAGAGGAGAGGAAGAGTCTTTATGAGAAGATGCAGGGAGCTGGAAGTCAACCAAGCGTCACCACTG
CCGAACCAACAGAGGAGAAGGTCCCCGAGGTGCAGCAGACCGCTAAAGACCCAGAAGCCGACCACCCTGTCCAGAATA
CCGAAGCCACTGATGCCCCCGCTGGGACCCCGACACTCAAAACTCCACTGACCAAGGAACTGGCTAAACTGAAAGCCG
AGCAGGCCCGTCTGAAGGAGATTGCCGGTTCTTTTACAATCTCTCATGTCATACCCACAGAAACAGTCGCTAGCCAAT
CACAAGGACACTCTGAAGGCGTCCAGGGGCCAGAGGAGAACCACATACAGGAGAGCAACGGCGAACACCTCCAGGAAG
CCAAGGAGGATGGATACCAAGAACAGAGAGATTTGGAAATGGAGTCAGTTGATTAATGCTGCAACGACTGTGAAAAGA
AACCTTG

>Sequ02760SNP1
CCTCCATACCTGAAATTACTTGAAGTGTGAGCCTGTGATGTTAATGATACACTTATAAAACCCTCTTTACAACGTTAG
GGAGTGTAATTCAAGCGCTGACATATTAACATTATTGCTGTTAAACGTGTCCTGTGTCACCTCTGGGTGGAGCCGCGT
GATACGAACGAACACTAGCACATAATAAAGCACAATGATGTCAGAACTCGGTAGTCCTGCTGCTCAAAGAGTCTGACT
GAAACCATCGGTTGGTCTAAAGCAGCTTAGACACTGTCCATGAGTTATATTTATTTTAATAACACAAATGTCAGCTTT
AGGTTTAATACCTGCTCACTCTCTGAATATGTGGCCTTTTTACAGTGGAGCAGTCGGGACCGCACCGTGATACAGTTT
GCGAAATAAAAGAACGGCACTCAGTAATTTCCAGCAATAATAAATATTGTT

>Sequ02777SNP1
TCTGTGTTATTTGTTTAATAAAAGTTAAAGAATTTACATATATCTGCAAAGATCACACTGATACTAAGGAGGCTCTGG
TGTGCCTTAGGGGCAATTGGAAGCTGTGATATCCAGTGACAAGTGTACATTCAGTGACAGCCCGGGCACTCTTGAATG
TGACCCATGCTAAAAAGATTTCTCTCAGGCTATAGTGAGCAGAGATATTTGGCACAACCTCACTAACACTTTGCAAGA
GTGAACCATGTCTTCACATGTAGTTCCACAGTACAATTTTCAACTGGTCATGCATCATTTTAACCCCTGGAGACTAGG
TCGTCATAGGCTTCTTCTTGATCATGATACAGAGTGGGTAAAAAACATTTTGTGCACATCATTTAGTAGTACAAGTAT
CTGAATAACAGAATACATGTTCAGAGATAAAAGGCTTTATCCACCTCCACCTGTCAACATGATTTAATCCTACTTGCT
CAACACATTTACTGTGTAACTACTGTATGTGTCTAAATGTGTCTGAAACTAATGCATAATGCACAATAAAGTAAAGTG
ATATGAATGT

>Sequ02797SNP1
CGACATTTGACTACCTCACAAACTGAATGGTGTGCAATGGTGGCAAAGTGAACTACCAATCAGAGCCTGAGAGCTGTG
CTGGTGCAGCTGCTTCCACATGTCATAAGACTTGATGTGAGGTGAACTTGCACCAAACATTTGCACAGGAGCTTCCTG
ACCAGCCACACCTCTGCTGCGGAGATCCTGTAGTGTCACTGCCTTCTGCTGGTAGGATAAGGAACAACAGTTGCTACC
CCATGATCAATTACAGTTTAACCCTTT

>Sequ02834SNP1
TTTTCTCTGTCTTGTCTGCCACAGGGGCCACCTCACTGTTTTTCTGTTGTTTGGTGGCTTTTAGACTTTCACTTTTGA
GCTTTTTCTCCTCTTTCTTTTCAACTTTATTTTCTTTAGTAATCTCACTTTTGGACTCTTCCTCCTGCCCACCAGCTT
CTTTCTTGGTTGTTTTAGAGTGTGGTTTGGGTGAAGATTTGAGACTCTCTTTGCTGTCAGTTCTTGATCTCATTTTAG
TCTGTTTGATGATGGGAGGTGGTGCACCAGATGATATGTCTTTTTGGGTAGCCACTGGGTATCGCAGAAAGTCCAGGT
GTTTCAGTTTCTCGAGGCCCTCGAAGATTTTATTCTGGGGTGCATTTCCTGGGAACAGCACCCTGACTATCTTTTCTG
ATGGACGGTGAGG

>Sequ02890SNP1
AAACAGGCCAGTGTGTTGAGGCACTGGTGCCATGTTCATGTCATATCACAATAAGCACAGGAGTGAAGACCCACTAAA
CAAAGTCTGTTAGCGAGAAAGCTAACCGTTAGCCTGCGAGGTTTGACATGGGCCACAGTTCTCTGACAGTGGGACTTC
ACCGACAGATTGATCAATTGATTAACATGTTTGGACGTCCACATCCAACACGGTGAGGTCATTTGTTGTCACTGTGAA
ATCAAATAATACCCACCAACAAA

>Sequ02929SNP1
CTCATAATTTAATTTATTCATGGACACAAACTCACAAAACCATTATGGTTGCACAACAAAGAATTAGTTTCTGCAGAC
AGACGGGAATGTTTAGCATACTTTTGTATCCTCTTTTTTATAATACAAATCACAACTAAATACAAATCAAACAATTTA
ACACAAATAATGTTAACATCCACTGTATTCTCTCAAGAGCAAGGTAGAGGAAGCAAGAGGGAAGAAAGAGTCAAAGTC
CTGGAGGCAATAGACAAACATGTGAACGCTGCCTGTGGCCAGAGTCCATCTGATTTAATTAACAGCAAACCTGACGCC
ACCAAATACAGCAGTTTTCTTTATGAAACAATAAACAGGAAGTGGACAAAGTAAGAGAAATCCCAGACTGTAAAATCA
ATGTCATGTACTGACAGGATTTTAGGGCATTTATTATTTTGTCAACATCCATATAGAAACATCTTTCTTCTGATGGAA

ACCTCCCTTTTCAGTGGGGGTGGATATTACATATCATTTATTGTAAAAAAAAA

>Sequ02941SNP1
TTGCTCAGCAGTAAAAAGTACAGTGTATACTACAGTACAAAAAATACATAATGTTGAACACTGCTCCACCGTGCCACT
GAACGTTTAGTAAAATATCCACTTTTTCAGCGTGCAAAACAACAAGCCCAGCTGTGAGCATCCAATGCAGGAAATATC
AATATGTTTAACCGTGTTATTCACCACCACCGCTTGAATCAGTGATTTCAGAACAACATCGCACAATGCAGAAAATCT
TTAGCGACAATAGAGGCAGGC

>Sequ03235SNP1
TTTTTTTTTGGTACAGCAGGTGTTTTCTACTCAGTCAAGACAAACAGTAACAGCATACACACGTAGAACATTTTTGAT
ATAAACATCTTATTAATCTCACAATTAAGAGTTTATCTTGCCATTGCTTTGATCAGAATATATCTTAAGTCTGACATT
TTGGTTTGGTGAAGGGACAGGCTTTGGGGCCTCCAGAATGACGATGAGTTGAATACATTTATCGATGCTCTGGGTGGG
AGAACCAGTAACGCAAATCAAC

>Sequ03290SNP1
TGATTTCCAGAATGTGTTAAATTTTGGTTGTGCGGTGATTCTTTATCCTATAAATAAAATTTAAAGCTCTAAAAGATT
AAACACGATTTCGTTAAAGATTTCTTGATATAACTTTTTGTTGTACTGGGTAATATATTGTGATGAGAGACAGGAAAT
GATGACCACAAATGTGCTTTCCACCCTGCTAAATGTCATCATTTGACCACAAGGGGGCACCCCAAACCTGCCTGTTGA
GACAGAAAGCACTGGACTGTGTATTGTTTTGTTTATGCATCTTAGATATTTTGGAAAAACTCTTGTATTTCCAGAGGC
CTTGCTCTGGTATGCAGCCTGTAGGAAATTATAATAAAATACAGTGGT

>Sequ03312SNP1
TAAAAAAAGCTTTACAATTTGAATTCTCCAGTTAGAATATTCACAGCATTACTATTCAGTTAGGTCTAAATTACACCT
TTAATCAAGTGAAACTACTATGACCATTTATTCACAGATTCACAGACAATTGAAGTATAATCACTGGTTGAAACTACA
AGGGTCAGTTTCCCCTCAATCACTGTTGTATCAGCAGTGACTCTCAGTAGCTTGTGTTTTTGATGGTGATCTTTTGAG
CCTCCTTTATCTTGAAATCATTGATACCCTGACTGAGTGCATCGCCGTTAGAGGAGAGGTGCCAGTATCTGCGGTCAC
TCTGTCTGGCCTGCTCTCCATTCACCACACTTAAGAAAGGTCCCCACAGGCTGGACTCCTTCTCAAACGTGAAGCCAA
CATTTGTCCCCTTGAGAAGTTCAAGGGCCTCCAACAGAGACCTGCCCTTTGGCACATCAACAGAG

>Sequ03369SNP1
TGTTTTACAGAGGAGCACATTGTTAACATCAGATAACGGCAGACAGCAAACTAAAGTAGTGACATTGGAACAGTATAT
ACATGTTAATATATCTAAAGCAAAATGAACCACTTTCTGTGACTTGTCACATTTGAAGCTGTTTGTTTTCGAGGCGGA
TCGTTTTTAATTGGCTTTAATGGTTTTCTCGATCCAGTCGACAAACTTGGAGACTCGAGCGTAGACGCCAGGTTTCAT
GGCGTTGGCGCATCCCAAACCCCAGGACGTGACGCCCTGCAGGATATACCTGTTCTGAGCATGGCACACCAGAGGACC
ACCGCTGTCACCCTGGCAGCTGTCTGTTCCTCCCTCGATGTTTCCA

>Sequ03370SNP1
CTTTTGGCAGCAGGATTTGATGTTTTGTGTTTTACCTGATACAAGCCACTTGAATGTTGAAGAACACGGTTATAACAT
ACAGCCAGAAAAACCGCACTGTCCTGTTTGTGTGCGGCTTCTCATGAAGATCAACCGCTCTGCAAATTAGAAACAAAA
GAAATCTTGTTTTAATATTGGCTTCCTCTCAAAGAAAACGCACTGTCATCAGAATTATAAAGCTTTAATCGGACTTAA
GTGGAGCGAACACCAGTGTGACTCTAATGTCTTTAATACTGTATTAGTCATTTATAACAGTAGAAACACTGAGTGACA
ACTTCATTTGATCAAAAGTCAAAATCTTGTTACCAACCTAATAAAACGTCTATTGTAACAGTGCTGTTTCATTATGTG
CACCAGGTACGTGTTGCAGTGTATCTGTATCGGCTCTGTACAGGTAAATGACAAAATGTTATT

>Sequ03384SNP1
CAACCTCAGTAACACATGCACTCAACACAGAGACGGCAGAATGACAGAGCTGAGAGGTCAGTGTGTCTGGACCATGAC
AGCTTTGTTTCCATACTGTCTGAGACAGACGGGGTGTAGTGTCCTTCAGGGTTGTACAATATATTTTAATGTGTTCAA
CTGGGTATTACTCCTCTCCTCAGCTGGGATTCGCATATACATGGGGATGGGGAGGACAAACCAGCATGGACACCTAAT
CCACTGCTGCCGAGAAACATCCTGTGATCAGTACCACCGCTGTGAAGCTTCTAATGAGACAGTCACAAAAACTAAACG
CTTCACACAGGTGGTATGTGTTGCAGGGTTGGAGTACTGGACTGTATCAGATCACACAGGTGAGCATGACAGCATCCA
CCTCTTCAACATGGTATAAGTGATACATTTTCTAAATGAAACATATTTACAGTGAGATCACAGAGAGAAGGGAGATC

>Sequ03443SNP1
CGGGATTTGAATGCATCTTGTCTATTGTTGTACATATACCTAACACTTGTTTGATGTGTGTATGTTTGTCTTTTCCTG
TTTGACAATTGTTAACACATGGATTTTCTATAAAGCATTTTCTTGTCTGATGTAGTTTCTAGGGTAATGTTTGTTAGC
TGATTTCTTGCACGGGATGGGCTGTGGAAATGGCTACTCTGTTGATGATCAACATTCGACTTTAAGCCTGAAAAACTC
CAATTTATTCCCAATAAGTTTGAGCTTGGTCAATAATGAATAAAACATACAGATTAACAGA

>Sequ03490SNP1
AAATAGAATTTTTATTAACCACACCTGAACACAACATTTGTTCCTGGCTGATTAGATGAGCTATAACTTAATAACATA
GGGAGGTGGTTTTTACTGTCGATGTCATATTCAGGAAAACAGAGGGTTCTTGTGACTTCACATTAATTCAGACACAAT
CTTCTTAATTACTGGCATTAATTCATTTAAAACATGCTTCTTTCATTATGTGCTTGAATGACATGAAAGAGCAGGAAG
TCTGACAGAGGAAGCTCTGCTGCCATGTTGAACACATGGACACAGAGAGATTAAGTTTCATACCTTTACTGATGAGCC
AGAGCATCATGACCACTCTCAGGTGATGCTGCTCATCTTCTAACTACGCAGCATGTTGAGCTTTGGAGACATATCAAC
ACGTGTTAAGACCTGAGCCACTGTGCGACAGTATGGTCAGACTACCGGGCTGCTCCGGGCCAGCAGAGGCCCGGGACA
GGGCTCTGAGATGCTTATGGAGGAATGTGACACAGCCACAGGTTGGTCAGAGCACCTTGCTGCTCATCATGTTCTGGC
TCATCAGTGAGGTACCTGTAGAAGCAGCTGTGAAGTGGTTTCTGCAGAAGCAGTGAGACATGAACAGCATTAACACCT
CAACATTTCCTTTAACACACACACACACACACTGTAACGCTGGACTCCCTTTAGAGTGTCATCTGTGTCAAACTCAAT
AAAATGCTGAATAATAAATATTTAATTGATC

>Sequ03558SNP1
CCGGTTTACTGCTACATATTTAACTGTATTTAACAAAAACATATAATGTTTTCTACATGTATTTATGTTTGTTCTGAG
ACGTAATGCTGATCTACGCTGATAAGATCTAGGCTTCATACAAACCTGACGGGTGGATTTAAAGGCCTCAGTGGAAAC
GCACATGAAATGGACCTTGCTTGAAAATACATTATGTATAGCTTATTAATTCTGCCATGTAACTAATAATTATTATTC
ACCTCAGTGTATATTAAAGAACTCTATTTTTGATAGTTTTCTTTCTTCATGTAGAAGAATTTAGTACATTTTCCTCAG
TGTTTATATAAACGTGTGTTTTTGATAAGCAATTTTTTTT

>Sequ03570SNP1
GAAAACTCCTGCTGGTGTTTCCGGGTGTCAAACTGGGATTTGAGAGAAACTTTCCCTTCAGCAGATGAACGTGAAAAC
AAACTCTGCTCATACATGATTGTGACTGATGAGGCTCTGTCAAATAACTGGAGGCTTTAACCTGAGTATTGTCAGCTA
AATGTTCCTCACAGTAAACATCCTCACTATGTACAACACCTACTTTACTTTGCTGTTTCATTTCCTGGTTAAATCTAT
AACTGTGCTTCATGTTCTATCAGCTGATGAAATGTCGTGTATGTTACATGCAAATGAATGAATGTACTCACTTGCTGA
TTCATTTATGAAAAATTATGTGGAATAGAATACTCTGTTAGAAACTCTTCTTTGTTGCTTTGACTTCTGAACTCATGT
AAACTCAGAAAATAAACATCTCCAGAATGAAATACACACAGATTAACAGATACACTGAGGTAGTAAGGAGGAAGTGAC
TAAGTAAAAAGGAAAAACTAGAATGAGATAATAAACAAATGTAAGTGAACAGAAAAATGTTCAAATGCAAACATTG

>Sequ03648SNP1
ACTTTATTCTTTGCTGTTATTCTTGTGGTAAGAAACAAGCAGCCACTTACAGATTAAACAGGTTCACTGTCCAACACA
ACAAGAGTCTACAAACACACACAGCCACTGACCAGTCACAGTGCCTTCAAAACAACAAAACCATCCAACACTAAAATA
ACGTTGTTGTAGTTAATCGTATAAAACAGCACCAGTTGTTGTGAACCTGTGGGAGAGATTTCACTCCTCCGTCTCCAT
GTGGTGCTCTTCATGCATGGGTGTTGGTGTGGCACGGGTGATTCACATTGGCACATTTATTCTTTCAACTAAAAAAAC
AAACAAACATCCTAAATATATAGTACACAGGCATGAAGACGTGATCATGTATAAATATAATACTGCTAGGCAGGAATG
AGACATCATCAGGAGGTGAGAGGATCAGTGGTTTTCTTCAGCCTGTGCAGCTCAATCTTCCTGCTCTGTGGAGACACA
CTACTCCTCTAACAATGACATTTTATTTTCCTACTAATAGGAAGTTTACTTTCAAAATGCAGTCCATGAGATGCTACT
GAAACTCAACAACATAAATTCATTTCTGTTGCTTACTTTCAAACCCGCT

>Sequ03661SNP1
CTCCCCTTCATAATTTCACGAAACTTTTGATACTTTCATTGCTGCTTGTATTTTGGTCTGTTATTACATTTCCACTGC
CAACATCTTCTATCAGTTCAGTGTAACAAAAAAATCTGCCCTGTGTAAAACAGACTTGACTCTTTCAAGCTTTTGATG
GTTTGATTACCATCCTGGGAGCAATTTTCTGGTTTTAATGCTGCTTTCCAACAGATTATATTAAAAAGTGCCTTAAAT
ATATGTGTAACAGAGTGTCTTTGGTTGTGTATCAATGTGAACATGTGTTTCTTAAGATGATTTTGTTCTAGATTTCTA
TCTGCTTTGTCTTCAGTCTGCAATAAA

>Sequ03720SNP1
TCTTCACTTTTCCTCGACTGTTTCTTCTCGGTGTGATCCTGCTGCTGTCTCGTCACCTCTTTACCAAACCACAGGTGA
CCTTTGGCCTCAGATTGAAGAGATCCGTAGCTGTCGACCCGGTGCTGAGCTTGACATGGCGCAGGAGGACGTGAACTG
TTAGTCGTAGTTGTAGTAAACATGGATGCTTAGAAACGCGATGAGCTGCTGTGTGATGTTGAGCTGATGATCAACATG
TCTGCTTGCTTCGTCTGCACTGTAGATTTCTACAGATTGTTTTTTGTATTCCCTTTTTAATAAACACTCCAGAACAAA
CAAAC

>Sequ03866SNP1
GGGAGAGGAGGAGGAGGTGGAAGAGAGGAGGGGCGAGGAGTCACAGGGAAGGAGGAGAGATCAGCAGAAGGCAGAGGT
GGAGGAGGACGAGAGGAGAGTCGTGGCCCAGAGCCAAGGAGACAACGTTTGTGCTCTGACCTGGGTCGTGAGACGGAC
GAGGCCGCCTTCGTCCCCGACTACAGCGAAGGTGAGGGCTCGGAGACGGAGAGAGGAAGAAGCGGCAGCCCAAGTCTG
TCCCCCAGCCAACAGTCCCACAGCCCCACCCCGAGCAACCAGAGCGACACGGCAACCACCATGGGAGACAAGAAGAAG
AAGAAACACAAGAAACATAAAAAACACAAAAA

>Sequ03932SNP1
GAAAGTAAAACAATGACAACTTAAGCCAAATATTCTTCAATAAATACCTAATGAGGATATTTTTGGCATAACTTAACT
GATGTAAGAAGAATGTAAGAACCACACTGTCAAAAGGCATAATACTGCTTCACTGGGTTGGAGTAACTGCCTGTAGTT
AATTTAAGCAGCACCATCTAGTGGTAACCGTTGAAATGTATGTCACAACCTCCCTGTGCATGACAGCGTCTCAGTAGA
GTTCAAACAACTTCATAGGCTTCATTATGACAGCGACAGCTGCCTCGTTCCTGCATTGCAGGAACCTTCACATTGACT
CCATGAACAACTCGACTAACCAAGTGACATAAAGGCTTCTTGTTTTCATTCATGGCAGAAGAGAAGAGCACGAGTGAG
TTCAAAGGTATTATATAAAACAGCATGAAAGGTAGAAAGAAAAAGTATAGAGTGAAGGAGGAGTTGGCCGTGAGCAGA
GCTACGGGAATAGAAAGTGCACAGGTGATGTGATGTCAAGGTGGATGTGGAGGAG

>Sequ04107SNP1
CTTTTTTTTTTCTTCTTCTTGTAGAATGTAATCACCAAACAAGTTTTGCTCTTTACAGATATACAGTATTATGTATAC
TATAACCTTCCTGTCGGTGACCCTCTGACAACAGTGTAAACAGAGAGAAAGAGCAGTTCCCGCTCTTCAGAAACACAT
GCAGGGAGTTGGGAGAAACATGTCAACTGTATGCCAAGGTTTAGTAACAGGAGACACAGTAATCTGTCAACATTTTGT
CAGATCAGATACTCTGGTACTGTTCTGCTCTCAGCTGAGGTCACCCCGGAGTTGCAGCGTCTCGTTTCCTCCACAAGA
GGGTGATAGTGACTCAGTGACAACAAGGTGAATGCCCTGGCGATACGAAGAAAAATCACTCTGGGTCAAACACAGGAC
TAAGATACTACCAGTGACTACTGTTTCTCCTCTAATGAGTGCAGTTAATATAACTTAGTCCTAGTAACAAAAACACAG
TTCAGAAAAATCACACCTACAGTTAGTTGCTGGTAGGGCATATCA

>Sequ04195SNP1
CAATAAAACAAGATGAGATAAGAAACACCTGAGATCATTTAAGGTTCTGTATCAAAGTTTCCTCTGCTGCTGAGGGTC
GACCAGGTGACGCAGCTTCAGCATCAGGTAATGACACGTCAGTGAGTTGCTATCAGAGAGCTAGCTGTTAGCATGCTA
ACTTCAGTAGCACTTATCATTGGTGTTCTTTCAGCTGTTGTCTCCAGAGGAATTAAGTTTTTTCTAGACTGGTAAGCA
AACAGCTGATAATACTAATGCTAATGCTAACGTTAGCTATGTAGCATTAGCTTAAACTTACATACAGCTCCTTTAAAA

CAAATATAACTTCATAACCTGAGACCCAACAAACAGAATTCATAAATATATCACACACACACCTGAGCAGAAACCCAA
CGAACAGAAATATGTTTACACGCAAAACAGCTGATTACAAAGCAATCCATCAATCAATCAATCAATATGTTGATTCAA
TAATTGACTTTCCACAATATTGAAATGATTGATAATAATCTACTTTACATTTGACAGGAGGGGGAGG

>Sequ04554SNP1
AAAGTCATTATCATGTTTATTTTTGCGTATTAGGCCATGTTCTTTCATAATGTATTGTGCTTTTCAACATAAAATATA
AGCTCATTTACTTTATTATCACATTCATTTCATATTTCTTGAACAGGAATCTCCATAAGACACACTGCGATGCACTGA
TATCAGGTTAAGGCACTTATTTGGGGCACAAGCAAGATGCCCCAGTGAGGCAATAATTTTACCAAATTGTAAAATTAA
GATTTTTTTTGTGAAATTAACATCCACCATCAATTTTTCCCTCAACATCTCATGAATAGACACAATCAGAAGTATCTA
TTAGAACAAACATGAGTCCCCA

>Sequ04627EST3
GGATGTTCATTCAGCATCTTGTCGGCGGATAGGTTAGAGCTGAGAGGCAGCCATGGCAGCTCCCAGCAGGAAGCAGGT
TTTCAAGTTTCTCAGCCAGTTTGGAGCGTTTGTTTTGACCCGGTTTGGATTTTGGAACTGCTTCTGTATGTTGATGCT
GTTTGCTGAGAGAGCGGACTCAAAAAGGAAGCCAGACATCCATGTACCGTACCTGTATGTGGATATGGGGGCCGCAGT
GCTCTGTGCCAGCTTCATGTCATTTGGGGTGAAGAGGAGGTGGTTTGCAATGGGTGCTGCCATACAACTGGCCATCAG
CACTTATGCATCGTATGTCGGAGAACAGGTGTACTACGGGGACTGGCTTAAGGTACGAATGTATTCCAGAGCGCTGGC
TATTATCGGCGGCTTTCTGGTTCTGGCCAGTGGGGCGGGGGAGGTGTACAGACAGAAACCTCGCAGCAGATCCCTGCA
GTCCACGGGACAGGTTTTCCTTGGAGTCTATCTCATTTGCACGGTGTACTCCCTCCAACACAGCAAAGAGGACAGACA
GGCCTATCTGAACCACATTGCTGGGGGAGAGATCACTCTGATGCTGCTGGAGGTGCTGTTTGGGGTGCTGGCTCTGGC
CTTCCTTTCTGGCTGCTACATCCGCCTGGCCGCTCAGATCCTGGCCACCGTCCTTCCTCTGGTGATCCTGCTCATTGA
CGGTAACTTGGGCTACTGGCACAACACTCGCAAGGTGGAGTTCTGGAACCAGATGAAGCTGATAGGACACAACGTGGG
CATCTTTGGCGCAGCGCTGATCCTAGCTACTGACGGCTGAACCCTCGGGCTCCCACAGACAACTTTTTTTGCACTGCG
CAGGATGAGATTGTCTGCCTTTACTGTTTGTCACTGCCCGACTTTGTTTTCAAAGCAGCTGTGGTTCAGTGACCTCTC
AGGATTCAGACTCTCCCAGCTGAGACAGTTGGATGAAAGCCAT

>Sequ04666SNP1
TACAAATTTTGCTCAACTTTCCTACATATTTAAAATATTTGATGTTTTGCCAGGCTTGGTTCTTTTAAACAGCGGATG
TAAATGTGCTTTATTTGCCTTGACTGACTCATTTTGTTACAAAAAGAATAAAAAACATGTTGAAGGCACTGCTGTTTG
TGCCAACCCAACCATCACCTCCTCTGCTCATTCAAATCATCTGATGTGAGCGACCCTGTCTGAGCATGTTACAAAATA
CATCAGAAATGCAGTTCAACCAATTGTATGTGCCACCTGGAATAAACTGTGAAGGATGAATATGAAAAAAAACTGATG
AAAACAACCATATCCTTATACAAGGCAACACTGGTGTCCAATAAAAGAAGGAAAAT

>Sequ04834SNP1
ACTTCTCAATGTTAAGTTCTTTTATGAAAAATGAGTTTCATAATGAGCTCTGGATCATTAAGAAAAACATTCGTCAAA
TTGGGAAACCAAACATTATTCCTTTATGAAAATTATTAGTGTACAAAAGAATAAGACAGTGTTAAGATGCAAAGGGCA
GGTATTTACATAGGTCTTAACAAACTTTATTAATACATGAACTTACTGTATTCTAGCATACTGTAGTAACATCCAGGG
GTGTACAACTGCTTTCTATAAACGTTCAAACGTCTGCTGATTTACTTGATGGCCAAAGCTTGCATCTCTTGATTTTAC
TGATGATCATGTTTTACCAAAGAGGCGAAAATAATAAAAGTGCCAGGCAGATTCCTCTGGATCGCCATCACGCACAGA
TAAAC

>Sequ04914SNP1
AAATCTCAGAATTCACTCAGTCATTAACAAATAATTTAATGCAGTTTGTACAAAGCCAGAGACTTGATTCCTCGTATG
GACAGGCACGCAGAGAGAAACTACAGGCAGCGTCAGGTGGAAACCTCGTTCCTCCGACAGACTCTCAACCATCAAAAC
ACTTTCATTTTTAAATAAGCAGCCAGAGACTGAGAGGAGGCGGCGCCCGACTCCGCCGCCACGGAAAACACTCAACGT
CCGGATGGAGACGAAGAACCGAAGAAAATCTAATGAAATGAGCAAAGTGAATCTTTCAAAACTAAACAACAAGAATAA
AACATGAAATATAAAAG

>Sequ05002SNP1
CGGAAATATTGTAGATAGTGTTTCATGGATACGGGAATAAACTGGGACAGAGCAGTTAGAAGCACAATGGAAGTCGCT
CCTTTCTTTGTGCTGGAGATCCCTTCTTTCATTCCTGTCGCATCAGTTTCCAAAAGAATAGTATCAACAGAATTTATC
AACACGTCGAATAAAAGAAAAAGACGATCCTGGCACTTAAAAGGAAAGAAGTGAAGCTCTGTCCACGTCAGAGGAGGG
AAGAGCCGCGGATGGAAAATGGGGCGGGTGGAGCCGGGGCTTCGTTCCTCCATCATTCATCCCTCACTGGGCTGAAAC
GAAGATTAACCTAGTGGTGAGATTCTTTATGTGAATCCAACTGGGAACGAAACTAATTTGTCCGTCACGGTGATCAAC
TTAAGTCGCATTCAACAGCAGCGGGCGTCATCTGCTTCCCCGCTACGAGATACAGGAGAAGTTGCATAGTAAAAAAAA
GAAATAATAATAATAATAAGAAGAAGAACTGTTGCTGTGTGTTTGTTTTGGCTCAATTCATTCAGTCATTTTATCCCA
TATTTCCTATAAAAATGAATATAAACTTCTATAAATCCAAAAAAAAAA

>Sequ05005SNP1
GCAACTTCAACACATGCATCAACTGTTGCAGGTAAAAATACATTCATTTGGTTCACTTTACACTTGACTCTGTCTGAA
GCTGAAGCGCGTTTTCAAAGCAGCTGACAGTGACCGCTGTCATTAACACAGGCATGCAAAACAGTTCTGAAATGACGA
GTAAGAAAACTGACCATTGTAAATCCTTGTTGCTGCTCACTCTGGGGGTGTCCTGACCTTTATTCTTCAACAGCAGCA
GTGTAACAAATTGCTCCCATACTCAAATAACCGGATAAACATCTCAACGTGCAACCGGGCAGAAATAAGTCAATCAAC
CGTCCATTTGAG

>Sequ05875SNP2
GTGACGTAGCAGATCGCGCACGGTGTATCTATCGGACAGAGTTCAGATAAGACTCCCCGGTTGACCTTGCTTTCGAGA
GCTGACCAGAAACAACACACAGTCCAGGGACAACATGACAACCTCGAGTTTATCTGCTATGCCGCAGCTACCAATGCC
TCCGCTGCCCACATTACCCAGCTCGGGATTTAGGTTATCTAAAGAGCATTTGGTCGTAGCGGTGCCAGTCGCAGTGGT
TGCAGCTGTTGGAGGCTTCCTAGTCAGCCATTACCTGAGCAGGCGGAGCTGTAAAAAGGGCCTGGTGAACACGTCCAT

CAGCAAAGACAGCCCCAAAGTGGTCCACAGCTTTGACATGGAGGACATCGGTACCAAGGCTGTGTACTGCCGCTGCTG
GAAGTCCAAGAAGTTCCCTTACTGTGACGGAGCCCACACCAAACACAACGAGGAGACCGGGGACAACGTTGGACCCCT
CATCATCAAGAAGAAGGATGCTTAAGCCAACCAATCACAGATCTCCACCCTCTCTTGACTTCTCATGCAGCCGCCCCC
AGGTTATACAATTGTTTACCATCATCATTAACAGTATCGCACTAATGGTTGTGGTACAACACTGCTACAATCAAACAG
TGGATATTCTTTGTAGCCCAGAATAGAGACCCAGAAAGTACTGAAAATATGCAAAATTTTGATTCTTGACCTTCCACA
CTGCATTAGACAACATGACTTTGAAATCAAAGGTCATTTGAGGAGAGATAAGGAGAGCACTTTGACAACCAGCACAGT
AAGACATGGAGGTATTGTGCCTGTGCAGTTGAATTTTGACATATTTAACCTGAAGCATTTGATCAATATTCTCATCAC
ACCTGAAATATTTCACTGTGTGGGCATTGTGTCGTCATGTCTGGTGTCCCCTTCCTTCTTGACTCCGCCAGGTGTGGA
GCAGGTGGTTGAAACATGTAATGAACTGGCAGCTCTTTGCCTCAGTCAGTATTTCATTGAAATTCAGCGCAGGCCTTA
TATTCAAATCTAGAGACCAGAGTCTGAACACACACAACCCACATGAATGTTACATGCTTTGATTTTTGCACAAAGAGC
TGCTTAGTCAGAGGAAACAGAAGTTTAGTTTGTCTTAAATTAAATAATAATCTCCGATCTTGAGAGCTGTTGAAACAC
TATCTGCTGTTTGTAATCACTTACTGTCTGCTTTCTGAGGAAACTGTTGTATTTAATCTGTAAAGTGTTCAAGGGTGA
AAACTCTGCTTGTAAAGGATATATTTTGCCTTGTCATCAGATTTATTTTCTACTGGCTGCGGTGTGTCTGACTTGATG
AATCATTGTTCTGTATACTGTGAATAAAACTTGTTGAGTGAATAAAAAAAAAAAAAAAAAAACCGAACCTCCCTAATAG
T

>Sequ05897EST2
TGATGAGTGGGAAACTCCAGAGAAGCTAGCCTTTCAAAGATTTTGGTAATATGCGTCACTGGATTGAGGACTCAGACT
GTCGCGACCAGTACAGTGTGATCTACGAAGCTGGAGAGAGGACCGCCATTTTTTCCAATGATGCTAAGGAACCAATCA
CAGTCGAAGAGAGAGCACGCTGGACAGAGACGTACGTGCGCTGGTCTCCCAAAGGCACCTATCTGGCCACATTCCACC
AACGTGGCATTGCATTGTGGGGCGGCGAGAAGTTCAAACAGATTCAGAGGTTCAGCCATCAGGGCGTGTCCCTCATCG
ACTTCTCACCATGTGAGAGGTATGTGGTGACCTTCAGTCCACTGATGGACACCAAGGAAGACCCGCAGGCCATCATCA
TCTGGGACATTCTGACCGGACAGAAGAAGAGAGGCTTCCACTGCGAGAGCTCTGCACACTGGCCCATATTTAAATGGA
GCCATGATGGAAAGTTCTTTGCCAGGATGACCCCTGATACGCTGAGCATCTATGAAACTCCATCTATGGGCTTGCTCG
ACAAGAAGAGTCTCAAGATTACTGGGATCAAGGACTTCTCATAGTCTCCTGGTGACAACATCATAGCGTTCTGGGTAC
CTGAGGACAAAGACATCCCAGCCAGAGTGACTCTGATGCAGTTGCCTTCCCGTCAGGAGATCAGGGTCCGCAACCTCT
TCAATGTTGTCGACTGCAAACTGCACTGGCAGAGGAATGGGGATTACCTGTGTGTGAAAGTGGACAGGACTCCTAAAG
GAACACAGGGCGTGGTTACCAACTTTGAAATCTTCCGCATGAGAGAGAAGCAGGTTCCTGTTGATGTGGTGGAGATGA
AGGAAAGCATCATAGCTTTTGCATGGGAGCCCAACGGCAGCAAGTTTGCTGTTCTCCACGGAGAGTCTCCCAGAATCA
ACGCCTCCTTCTATCATGTCAAAAACAACGGCAAGATTGAACTCATAAAGATGTTTGACAAGCAGCAGGCCAACAGCA
TCTTCTGGAGCCCCCAGGGACAGTTCATGGTCGCTGGCTGGACTCAGGAGTATGAACGGAGCCCTTGCCTTTGTGGAC
ACGTCAGACTGCACCATGATGTACATAGCAGAGCACTACATGGCCTCTGACGTGGAGTGGGACCCAACCGGTCGCTAC
GTCGTCACCTCCGTCTCCTGGTGGAGCCACAAGGTGGACAATGCGTACTGGCTGTGGACGTTCCAGGGCCGTCTTCTT
CAGAAGAACAACAAGGACCGCTTCTGCCAGCTGCTGTGGAGACCCAGACCACCCACCCTGCTCAGTGCAGACCAGATC
AAGTTGATCAAGAAGGATCTCAAGAAATACTCAAAGATCTTTGAGCAGAAGGATCGTCTGAGCCAGTCCAAGGCTTCG
AAGGAGCTGGTGGACAAGCGCCGGTCCATGATGGAGGACTACCGTCGCTACAGGGAGACAGCGCTGCAGACCTACCAG
GAACAGAAGAGCATCCGCCTCGAGCTCAGAGGAGGAGTGGACACCGATGAGCTGGACAGCAATGTGGACGACTGGGAG
GAGGAGACCATTGAGTTTTTTATCAACGAAGAAATCATTCCCCTTGGAGATCTGTAGTCCCCTACGCAGTCCATCCGT
TTTTGTGTGGAAGGAGGAGAGAGGAGCTGTGTCATTGATTGGAGAAGGACGACGACCACGCTGTGAATCTTGAGGTTC
ATCTCAAGCCTCAACCTTCAACATTAGCGACACCATCGAAGCACGCCTTTGCGGATCTGCTGTCACATCGAGGGACGA
GAGAGGATACTTTGTATTGAATATTTTCTCTTTGTCTCTCACCATCATCCGCCCCATTTTTTTTCTCTGTGGAACTTG
ATATTTAGTAATACTGCCTCCATTTTTCTATTCACCAATTGGAGTCGATCAGTCTAACCGCAGCCCCGTCTCTTGGAC
CTTGTTCTTCCTCTTGAGGTTGATTTCTGAACTAGCTTCACCATGCTAGTGTGTTATCACAGGGCAGCACAGAGTGCC
ATACCAGTGGGGGGTGGAGGTGTTGTGACTAAACAAGTAGCCTTGTTAAGAAATGCGTTGATGTGGCGCTATAGTGGC
TTGTATCCTCCTCCTTTCAGACTAGGCTTTCACACAGGGAGGGGTTGTGCAGTGGGAATAACTTTAAATAAACATTCA
AACTCAGTAATACCGAG

>Sequ05938EST2
TTAGCAGCCTGTCACTCACCTTCCATGGACAAGAGCTGCTAGCAGACACCAGCCTGGAGCTCAACTCAGGCAGACGCT
ATGGCCTCATCGGACTTAATGGCACNGGAAAATCCATGCTGCTGTCAGCCATCGGGCATCGTGAGATTCCCATTCCAG
AGCACATAGATATTTACCACTTGACCCGGGAGATGGCCCCCAGCGACAAGACAGCTCTGATGTGCGTTATGGAGGTTG
ATGAACAGAGGATTATGCTGGAGAAGGAGGCAGAGAGACTTGCCCATGAGGACTCTGAGTGTGAGAAGCTGATGGAAC
TGTATGAGCGTCTTGAGGAGCTGGATGCAGACAAGGCAGAGATGCGAGCCTCACGGATCCTCCACGGTTTGGGTTTCA
GCACCGCTATGCAGCAGAAGAAACTGAAGGACTTCAGTGGAGGATGGAGGATGCGTGTTGCTCTGGCCAGAGCCCTGT
TCATCAAGCCCTTTATGCTGTTGCTGGATGAGCCCACTAACCACTTGGACCTGGATGCTTGTGTGTGGTTGGAAGAGG
AGCTCAAGTCGTTCAAGCGAATCCTTGTGCTCATCTCACACTCTCAAGACTTCCTGAACGGTGTGTGCACCAACATCA
TCCACCTACATCAGAGAAAACTGAAATACTACACGGGTAACTATGACCAGTATGTGAAGACCAGGGAGGAGCTGGAAG
AGAACCAGATGAAGCGCTTCAACTGGGAACAGGACCAGATAGCACACATGAAAAATTACATAGCCAGGTTTGGTCACG
GCTCTGCCAAACTGGCACGACAGGCACAGAGCAAAGAGAAGACACTGCAGAAGATGGTGGCATCAGGCTTGACTGAAC
GAGTTGTGAATGACAAGACTCTGTCATTTTATTTTCCTCCCTGTGGGAAGATTCCTCCTCCTGTTATCATGGTTCAGA
ATGTGAGCTTCAAGTACAGTGACAACACACCACATATATACAAAGACCTGGAGTTTGGTATTGACTTGGATACACGAG
TGGCTCTGGTGGGACCCAATGGAGCGGGGAAGTCCACACTGCTGAAGCTGCTCATGGGAGAGCTCCTGCCCACCGACG
GCATGATCCGCAAACATTCTCACGTCAAGATTGGCAGATATCATCAGCATCTGACAGAGCAGCTGGAGCTGGACCTGT
CTCCTCTGGAGTACATGATGAAGTGTTTCCCTGAGATCAAAGAAAAGGAGGAGATGAGGAAGATCATTGGTCGCTACG
GCCTGACAGGAAAACAGCAGGTCAGTCCAATCAGGAACCTGTCAGATGGTCAGAAGTGCCGGGTGTGTTTTGCCTGGC
TGGCCTGGCAGAACCCTCACATGTTGTTCCTTGATGAGCCCACCAATCACCTGGATATCGAGACCATTGATGCATTAG
CAGAAGCAGTCAACGAGTTTGACGGCGGCATGATGCTAGTTAGCCACGACTTCAGGCTAATTCAACAGGTGGCTCAGG
AGATCTGGGTGTGTGAGAGTCAAACCATCACAAAGTGGAATAGGGACATCCTAGCGTATAAGGAGCACTTGAAATCAA
AGATCGAAAAGCAGGCGCATGACATCTAAAGCATCGTCACACTCTGAGCCACTCCTTTATCTTGCATCATGTATTTGA
CCTGCAAGCACCTGAAGGGAAGATCAGGGTTTGCATTGCAGGAAATGTCCCAACAAGAACTGAAACAATCTCTTATGC

ATCTTATTGGACACGTCTCATGTACATGTTGTACTGTATTTCCATCTTGGTTTGACAGTGCTACACTGTACTGAACCT
GACCCAACCCCTCCTCTCCCTTCACCTCAGGCTGCACTCTGATTGGCTGATTCACCTGAAGCATCTCAGTGCATTTTG
GAATGCATTCCATTGTGCTGTCTTCATAGAAACCATTTTATTTTTTCCAAACGTTCTGTTTTATCCCAACATGTATGC
AGCAGCTTTTAGGTCATATTTTATGATATTTAAAAGTTGACTTTTTTTGTCTGTGTAAAAGCAGTTCTGCCTTATTTA
ACAATGTAATGCAATAAAGGTTTGTCTGAATAAACAGAACAATAAAAAC

>Sequ05946SNP2
AATGCCAAAATCAAAGGAAGTGCTGTCTTCCACATCTGGAAGTGACTCCGACAGTGAAGTGGAGACCAAGGCAAAGAG
AAAGAAGTCAAGTGCACCAGAGAAAGCAGCCAAGAAACCAAAGAGTGGAGAGAGCTCCAAGCCAGGTGGCTCATCCAA
GGGCAGCAGTAATGCTGATGACAACATGTTCCAGATTGGAAAGATGAGATATGTCAGCGTCAGGGACTTCAAAGGTAA
AGTCCTGATTGACATCAGAGAGTACTGGATGAACCAGGATGGGGAGATGAAGCCGGGGAAGAAAGGTATCTCCCTGAA
TCCTGAACAGTGGAACCAACTGAAGGACCAGATTTCAGAAATCGATGATGCCATTAAGAGAATATAAGCATTCCTGTC
AGTATTATCTGTCGGCTCACCCGTTTTGTAAAAAGCCTGGGATTGTTTTCTACTTGTTTAATTTGTAGTTGTATAGCT
CCAGTGTCATCTGCATACTTCTGTGTTTGTGTGAAATTTGTGTTTGAGCTGTTGTAGTGAAGGTGTGTGTGGATGAGT
AGGTGATCGATCATCACAGCTGGTGGGAAGGGTTCGTCAATACAGTGATGTTTTTGTATCAAATAATAAAAATGAGGC
ACTAGTTTAATATTGTTGTTCAGTTAATGTTGAAGTTTGTGTTCAAAGTTTGACCCTCATGTAAATAGAAATGATAAG
AGTGCTTCAGCTGACCTTTGCTTTATATAGTACCCAATATTCATGTCTGAGAGACAAGCCCAGACTGTTGCACTGTAC
TTTTAGGTAAGTAGATTCAGATTTGGAAAATAAAAGATCCCATTTAACAGACTGGTATATTAAACGATTGTAATGATT
GAAAAGCACCTCTGTCAGTTGCAAAGAATGTGAGTTGGGGACCAGTGAATGAGGCTTGTGGTACTTTAGTGAGTAAAT
ACAAAAGCTGTAAATTTTCTCCAAACTTAGTGGATGTTTGTGAGAAGATAAAAGTAGATCAGGGGACACCATAAGTCG
TGTATGGGTCATAACAGTACTACAATTTTTTAACTGAATGATGGACATTTACTTTTCATTAATGTCATTTTCCCTCAA
ATTATTCACTCTTAACACTGAATTGACTGACATTATTGGAAATACCTGAAACAATTCTGCAGTTTATCAGATTATAAT
AGGATTATAATTTATTATAGCTATGAGAAATGTGATGCCCAGCAGTTAAGTGTAATGACAGTTTACCTTGTAAGTCCT
TTAGTGAGCCTACTACATGTTATGATTATTGTAAGAAAGTATTATGACTGTTTATGAGTGCTTGTAGTATAATTACTC
AATGCTATAAGCCAATTATAATGTATGATGAATGTTTGTAAAACATTAAAGACATGGGCTTCATGGAGTGTTAGAACA
ACAATGTTCAAACTGCTGCCTTGACAGTCCATATCTTAAACCCAAAAAAAAAAAAAAAAAAAACNGAAGACTCGACCGA
AGGCACCAGGAGGGTA

>Sequ05974SNP2
CCTGTTGTGATTTTTTTATTTATTTAAAGACACTGAAAAGAAAAATACCTTTACCCATTTTGTGTTCCTACAGTAATG
TACAGTGTGTTCAGCTATGTATTGGTAGATACCAATATACAAAAGAAAATATGCATTGCATTGATATGCATCTTGTCC
AGATATAGATCACATTTTAATACCAGGTGTAAACAGGGTGAGAGTTAACCATAAGCAGATAATTGATGTTATGTATTA
AAGCAACAGCAAAATCTTTGAAGGTGTTAATGACATAGAACAACAACAGTAACAAAGAAAAACACTTTTATTATTAGG
AGGCAGTGTATATGTTTACTGTGGGAGATCTATCATGCTACAAAGACTATGGCTCTGATAGTGAGGATGGACATTATT
TTACATTTTTCATGATGAGACATCACTTCATTCCAGTTGGGAAACCTCGTGGAAATATGCACCCAGCATCTTGATTCC
CTGAATATAGTTGGATCTGTTGAATTTCTCATTCTGGGAGTGAGCTCCATCATCAGAGGATCCAACTGGAAGCAGCAT
GACATTACGCCCTGTGGCTTCCTGGAAAGTCAGGGTGACGGGAATGCTTCCACCCTCACGTGTCAGGTCGGGCTCGAC
ACCAAAGACTGTTTTCATGGCCTTCCGACCAGCCATGTAGTGTGGGTGGTTGAAGTCAGAGACCCAGGCCCTGGCTCC
GTGGCCCATGTACACTTTCAGTTTGTTGGGGGTTCCCAGCTCAGCAAATTTCTTCTGCAGATGGCCGATAACCTGCTT
CTCCACATCTTTGGGGTCCATGTCAGGGACGAGGCGGATGGAGAACTTGCCGATGACCTTGCGGGGGATAACAGTCTT
GGCTCCTGCTTCAGCGAAAGCCCCCTCAATGCCATGAAGAGAAAGAGATGGGTACCTCCAGCGGTGCATTAGGATTTG
CTCCTTGGTGTCATGCAGCAGATGTTTAACTCCAACATCTTTGCAGTACTCATCCAAGTCAAACTCAATTTTCTCATA
CAGCTTCTTCTCCTCTTCTGTCAGAGGGGCCACGTCGTCGTACAACCCAGGGACCAAAATCTTCCCCTTCCTGTCTAC
CAGGGAGCCCATAAGTGCAATAAGGTCAGTCATGGCCTCATGAACAGAGCCACCAAACACCCCTGAGTGTAGGTCCTT
CTCACAGCCCTCCACCTCAATGAAGAAGTAGCAGATTCCTCTCAGCCCGTAGGTGATGCAGGGTTTGGTCTTGCCCAG
CCAGTAGTTGTCAGAGATGCAGACATAGTCCACGTCTTTTAAGAAGGTGTCTTTTCGAGAAACNCCAGTTCATCCAGG
CCCTCAGATCCAGACTCCTCCATCCCCTCGAAGCAGAACTTGATGTTGATGGGAAGCTCCTGCTGGATCTTCTGGTAG
GCCTCAATGCAGTTAAACCAGGCCAACACTGGACCCTTGTCGTCAGTTGAACCTCTTCCATAGAGCTTACCATCTTTC
TCCACCAGAGTGAAAGGCTCTGTATCCCAGCCGTCATCAATATTGGCTGGCTGGACATCAAGGTGGCCGTAGATGCAC
ACAGTCTTCTTACCCGGATCTGACCCCAGACGCCCCAGGACGATAGGAGGCAGGGGGATCTCCTCTCCGGAGGGAAGC
GTCTGGTTGCCAATGTCCACCATCTCTACTGTGCCCCCCAGCTTCTCAATGTCCTTGGCTGCCATCTCCATCATCTTC
TTGATCTCCCCACGCTTCTCTGGCCAAGCAGAAACGCTCTGGACTCCCACCCATTCAGCGAGACGCTCCACATACAGA
TCCTGGTGTTCGTCCACATACTTGAAAAGCGCTGTGAGATGAGCCATCTTTGCCAGTGACAGGAGGAGCTGACACACG
GCTCCTGTGTTTGTTACCGACGGACAGCGCTGTTTCTTAAACTTTGACGCAATCCTTTATTGAAGCCGTTATTGAGCC
GACAACTCACCTAGCCAGCGCACACACAGTG

>Sequ05983SNP2
CTATTTTTAGCGGCGGATTAATCCACATTTGCTGCTGTAGTGGGTATTTTGAGTAGCAGGCCGATGTGTGTGGGAGTG
AGTGAAAATAAACTTGTGCACAGTGTGTGTTCATGTAATGAAGGCTGTCACCCAGCGCAACAGTGTGGCTCAGTGATG
TGTTTTTAATGGTGTTTGGAAACAATGGAGCTCTATGGCTCAGAGGAGTCAGATGCATCAGACTGTCACACACACAAT
AGGAGATTATCTGTGTTGGTTTTGGTCTGCGGATGAGATTTAATGACTATAAGAGGAGGTGTGTCCTGAAGTCGTGGT
GTGCCTTTGCTCGTTTTATCTCCAGCAGCCGTACAGAGTTCATTGTGTTGCTTCTACTTGTCTGAATCTGTCTGTGAA
GTCATTCCAACAGTCTGTGGAGTTTAACAAGGAATCATTGAAAAACAACATGGTTGCGTTTGCTTGTTTTATTATTCA
ACTACTTTTTATTTAGTTTAGAATGAATTGAAATACAGAGTAAAGACCATATACTGGAAGCTGTTCAGTAGTCCAATG
TTGTGACTGTAACCAAACATTTACGCACATAATCACACCAAACAAACCACAGCAAAAAAGCTCATTAACTACAAAACA
TATTTCACATATAAATAATAATTATAATAAAATCCATTTCTCCAATCATTCAGTTACTTTTTAGACAGATTTTCTTGG
TTTCAGACTGTTCGTTTTCATTATTGACTAATCTGCATATTATTTTTCAGTTACTTCACTAATCATTAAGTCTTTAAA
ATTTGCTGCTTTATAGAGAGGGAAATACTTTTACCCAATTACATTTATCTGAAAGCTGTGATTACTTTACAGATTAAC
ATTTTATTTGCACTGAATGAGAAGATTTTGTAAATTAGGGACTTTAGTTGTAGTAGCATATGTTACATTATACTGCCA
CATTAGTGCTAGTGCTTTTACATGACATATACTCAGTTGTAGAAGAAGTACTCACATCATTTATATAAGACAATACAC

TACACTGGATTAGTTTTAGCTCGGTGTAGCTAATATATTGAGTGTACACTAAATATGCGCCTCGTGGATTGATCCTAA
AATCATTTTCCTAATCAAATAAACTTTTGGTTTCTTAAACCTATAAAAAATTATGACATTTCCTCTACAGTTTCTCTT
GTTTTGTCTGATCAATAGTCCAAAACTTAGCTCAATCATTATTTGATTTCTATTTAATTGGTTGAACTCCAATGTTTG
CTCAATGTGTATTGAACAAAAAAAGGGTTTTTTTACAGTACACATCAGAACATCTAAGATTGGAATTAACAAACCGAC
ACAAAGCAATATTTCTATTTTCAGCTACTGTCCAAAAC

>Sequ06020EST2
TGGTCTCCCGTACTGCTGAAGCGGTGCTGGCCGATGTGTCAAAATGGTCAGAGCTAACAACACTAGAGTAGCCTGCTG
GAGATGGGCTTTGACAGAAACAGAGCAGAGAAGGCGGTGGCCAACACGGGGAACCAGGGGATAGAACAAGCCATGGAC
TGGTTAATGGAACATGAGAACGACCCTGACATTGATGAGCCCTATGTGCCACCTGTGGGAAACGTCCTGGGAGGGGAA
GCAGACAGCCAGTCCAACGCAGAACAGCAACCCTAGCCGACACCGCTGAAGTGGGGATATCGGCTACAACGAGACTGA
TGAGAGTTCAAAAGCACCCAATGACAGAGGAGGAGAAACGTGAGCAAGTTAAGAGGCTAGAGGAACTGATGCGGGTGA
AGCAGGCGGAGAGAAGAGAGCGAGAGCGGGCAGAAGAGGTGGAGAGAGAGAAGGTTCGGAGGAGGCAGGGCCAAGAGT
TGCAGCAGATCCGCCAAAAACTGCAGGATGATGATATGAAGAAGCTTGCTGATCAGCGCAGGAAAGAGAAAATGGAGG
ACAAAATGGCAAGACAAAGGGTCAAAGAGAAGATAGCGCGAGACAGAGAGGAAAGAGCGCAAAAGTTTGGAGGCGGAG
GACCCCCGAGCACAGCTGCATCATTCCAACCTGCCCAGCCCAGCCCCTCATCACCCACCAGTCAGGGCCCTCCACCCA
CTAAGAAGGAGTATGATGAGTCCAGGATACAGGTACGTCTGCTGGACGGCTCCACCATCACGACGGTCTTCAAGGCCC
AGGAGCCGCTGGCGGCAGTGCGTGTCTACGTGCAAATGAACGGCAACACACCAGAGGGTCAGGACTTCATGCTGCTGT
CGCCCTACCCGCGACACGTCTACACCGAACTGGACATGGAGAAGCCCCTGAAAGAGCTGGGTTTGGTGCCTTCAGCTG
TGCTGGTTGTTGCCAAAAAGTGAGATCAGGAGGATCTGCTCTGTGAGCCTACCTCACCACTTCCACTACATCACCACC
AGAGACACCTGAAACGGATGGATCCAGGAACAGAGCCGCCACCTTTTTAGTATCACTCAATAAGCCAGCTAACTGAAC
TAAAGCAGAGATGGAGCTGGAGGCTGTGTTCAGACAATGTGTTATAGTTCTTAAAATGAAGATATGGAAAAGTTATAA
GGAAAAATGGTTTGTTTTCTTTCTAAACTAACTGAATGCAGCTCCTTGTCTTTCTGGGCTGATTTCACTGGCTGTAGA
TTGGATTGACAGTGACCTTTACCTATCAAGAACAAAGTGATGATAGCTGGCTTCTGACTGATACTGCCTCAAGAAGCA
CCGCTCATGGTTTCAGCAACACATTTACAAAAATGATACAGAACAAATTTGTTCAGTTTTAACCCGATCCTTATATAG
CAAAGCACCACACAAGACTTACTTGATTGGTTCTGAAAGGGCCTTAAATTTAATCCCCACTATCTTCCTGATTTTGTG
ACATTCTACTTTGCCAGTTTTTTTATAAGTTAAAATATATTGACAGTTATGTCTACATAATCACCAAGAATCTACTTA
CAATAAAGCTGACTTGGAGAATTGTGATGGTGGACTGGATTTTCTAAAAATCACTTGTGTAACCCTCCTGCTGATATT
CTGTATTTTCTTTTGTCAAACACTTTTTAGTTATTTTAATTATTAGGATTGATTTCATTTTATTATGACCATGGAAAG
CTGGAATAGGCCAAAAGTATCACTGCCCACTGGATTTTAAAGCACCTAGTGAGCTGGAACTCTGTTACATATAAAATA
AGATGAAAATGTTAAACATGAAAAAAGCAACGGCTAGTAAAATAATAAAAATCATTAGCTGGCTAGAAATTAACTAAA
AAAAAAAAAAAAACCGACCTACC

>Sequ06023SNP2
GGGGGTCATATCAGGTTCTGGATCTTGCTTTATACTGTGGGATTGTGTCTGTGTTTGATGCATAGCTCACACTTTAAC
ATCCTCTCTGCTCATGTTCATCTTTGATTTTATTGGGACCACATTGTCAAATCAAAACCTCACCCGCTCCATTACACA
GGTCACTTATAGTTCCAATATCCTGAACATAGCACCTGATTCCATGGTGGTCTGCTCACACCTTGATTGGCTGGGTAT
AAAACACTGGAGTGATCCAAGATCAGCATTTTTTATAACTCTGTTTTCCATTTGCTTTGAGATCATGTGTTGGCTAAG
AGCTTTGTTTAACCTGTTCTAGAGTCATCTCTCTGTCCTGTCTGAACACACTATATTGTAACATCAATGAGCGTTAAA
GGGGTAGTGACAAATAATTTTCCTTAAACATGTACATAGACCCTCCCCTGACCTTTCTAATGTCCTGCTCCTTTTGTG
TTTATCTCCACATGACGAGGACTTAAAAACAAACAGGTAGAGTGTCAGGCAGAGAACAGTTCCTGTCACACTCTGAGA
TGGGCAAATGCTTTTCAGCGTCTGTTAACTGTTTCTTTGGCGTTGTCCCAGATTGACTCTCAGGTAATGTCAGAATCT
ACCTCTGTAGGTGAGACTAACTGTGAAAGAAGAGGCACGAGTCACCATCGCTTCAGAGAGCTTTATTTTTACTAAAAG
AAGCCCACACCCCCTGATTGTACCTGTCTTTCTTTTCAATAAATATTTTGTAGAATTTGCAGTTTCTATTCACTATGG
ATTTCTTACTGGGTCTGGTGTGAGTGAAGTGGACAGGAGGTGTCGATTGCATGTGCAGCATTCCTCAATTTTATATAA
ACATATTTTTGAAAAGTTACAAAAGTTTTATTGTAATTTTGCATTACGTAACAGGAAGAGGAAAAGAGGTGTTCCCCT
CAAGTGCATTAATTTAGTCCCACACTTTCACTCCAGCCGAATACTGAAGACTTAATGAACAGGAGGAAAATACAACAG
GGAATGTTTCTTCAGAGGACAGCAGTGATATGTCGCTAAATGTGCAATCAGAACAAAAACGACAGGTGTGAAAGAAAG
CTTATGGACATGACCAGCATGGTTAGATTCACTGTCACAGTTCAGAGGGATGGAGGTCAGGTGTCTTCTGTCGTCCTT
CTCAGTGGTTTCCTGGTGACTCGAGTCAAATGGTAAAATACCACTTTATTTTCCTCACATGATCCCTTTGAAATGAAC
AGATGAACTGGTCTTAGTAGCTTTGTGTACTTTTAAGTTTTTCTGCTCCTTTGAAATTCTCCAAACACTGGAGAAACG
ACCTGAGCACATCTCAAATCTTGTAATGAGTGCGTACGTGCTGTTGGTACCTGGGCCACAGGGATGTCTGAGCAAGAT
AAGACTGAGTCCAACTGTCTGTTAGCAAGAGTGGAGTCCATGCTGTCTCTGACCCTCCACACTGTTCATCCATGTAGA
GGCAAATTCTTCATTTTGCACTCGAGTTAAGCCAGAAAGGGAGGTGAAGGTCTGAAGCTCAGGGGGTGGAGGAGGGGT
TGAACCTGGACAGGGTTTCATGCAGAGCTGGGTTAAGATCCCTGCGCCTCCAGCTCTGATGCGGGGGTGTTGTCTGGC
TCTTCGTCATTGTAGATGTTTTCCGCCATCTGCCAGACTTGCATGATGTTGTCCTC

>Sequ06038SNP2
CCGCACTACTACATTCTAATAATTTGATAATTTGTACATTTGATAAAACATTCAATCATGTTAATGTTTCTCCTCTTT
GCAACCTGTGCTCCTGGGCTGGCCCAAATATCTGTTTTCAGGTTCTGTGGGTTCATTAGTATTAATGCGGTAAACACA
GTCAGCTCAGGTCAACCATCTGCTTTCAGTGTGGCTAGTTTACAGAAATCACTGCCAGTGATCAGTGAGTTCTTCAGC
TGTTTTCTGCATCTTTGGGACCAGCACCTTTTGAGTTGCTACATTCGATGGCTGGCTGGATGTCTCAGAATGGTAGGA
AGTCTAGTTAAAGCGTAGTTAATTCACAATTTGGCCTGTGTTTGATTAAAACATCTCCCTGAAAAAGCTAAACCAATA
GCTGCTTGAGAAATAGTCTTGAGAATGTGTAAAATGATTCAGAAATAATTCCCTCTTGATAATTAGTTAGTATACAAA
GTCAGCCCTAGGAACAAATGTTCCTGTTTATAGCTATATTCACCTCTTATGTCTTATGGGTATATTTATTAAATTTCA
AGGTTCTCTGCAAAGCTAACAAGTTCGTCATTTCATAGGTTTCTTTCATAGGTTTCTTTCATACTTACAACAGGTTTA
TGGAACATAATGCAAAATAAAATTGGCACATCTGAGTATGTGTCATTTGAGACTCTCACAAAGTGATAAAACTTCATT
AGAAGGTTTAAGATGTGGTGGAAAGGGATGTTGTGAAGCACTTGTTTAAAAAATATACAGTGATGTTAGAAGACAACG
GTGTGAAACGACTCATGAGAAACTACTGGACTTCTTTAAATGTAATGTGTGGAAAATGTTATGCAGAAAAACTCCCAG
CATAATAAAATAAAGTTGTTAAGTTGGTT

>Sequ06067EST2
CGGCAGTAGCTTGGCAGCGGTGAGCTGAGGTGGACTTCTTCTCGTCGGTCGTTGTGAAAGGAGCGACACGGTCCAGGA
GCCGGAGCCCAGCATCAACAAGGAAACCTCAGAGACTCAGACAGCCTCACAGACACCACGATGATCCGGATAGCCGCC
TTCCTCGCTCTGCTGGTGGTCGCCTGCTCGGGAGAGAGCTGCACAGACCCAGTGATCACTCCGTCGGCCTACACCACG
TCTGACGCCGTCATCTCTTCCGAGTCTGTCTTCATCGTTGAACTCAGCCTGGCATGTGCCAACGGAGCACAGAGTGTA
ACTCTGTACGCTGATGTCAATGGGAGACAGTTCCCTGTGACCAGAGGCCAGGATGTTGGCAAGTACCAGGTGTCCTGG
AGTCTTCCTCACAAACAGGCCAGCTCTGGAACATATCAGGTCAAATTCTTTGATGAGGAGTCCTACAGCGCCCTGCGC
AAGGCCCAGAGAAATAATGAAGATGTAAACGCCATTCAGCCTCTCTTCTCCGTCAATATTGACCACAGGGGTGCGTGG
AATGGCCCATGGGTCTCTACTGAGGTGGTGGCTGCCCTCATCGGTATCCTGGTCTATTACATGGCTTTCAGCGCCAAG
AGCACCATCCAAGCATAAACGGATCCACATTCAGTTTTTCAGCAACTGGATCGTTAAAGACTGAATTCTGGTGGAACT
CGATCAACGATCCATCATCCAGCGTTGTGGCAAGCCAACAGGGTTTTGCCACTTGATCAGCTTTGGGATGAGGAGCAG
GAGTAGCAGTCTTTGTATTGTTTTATTCAGACTGTGTCTGTGTGCCACCTGTCGTATCTCTTTACAGACATGAATTTT
GTCACGAGTTTGTCCTGTCAGTGTGGAAGAAATTTTGTAATTGTTTTTGGTAAAATCAGACAAATATACCTGGACCAC
AAAAATAAACAAAACAAATATAGATGGTGTACTGTATTCAGTCTTACTTCAAAGATGATGCTACATCCAGACCAGACG
ATCCTCTGTCCTTCGGTGTGGATACATGTGGGACTTGTCCAGATGTGGTCAACACCAACAGAGAAAGGTCAAGCCTGC
ATATGAAATGTCAGTAATGTGACTGCACTGGGCTGGCCTGAACAGGTCTGGTGGATATGTCTCTTCCAGTGAAATGAA
TAAACGGCTTGTTTTTACCTAAAAAAAAAAAAAAAAAAACCGACCTACCCTA

>Sequ06078EST2
GACGTACTTGCTCTAAGTTTCGGCTCCGGGGAAAACAACATCTAAAGGACCCCAGCCTCAAGAATAAAACACCGAATA
TCCTGCGTCTGTACCAACTGATATCATCTTCAACGGATTCATCGTAACACGTAACTTCTTAACCACAAAATGCCCCAG
AACGAGCACATTGAGTTGCACCGCAAGCGGCACGGCTACCGCCTGGACCACCATGAAAAGAAAAGGAAGAAGGAGAGC
CGTGAGGCCCATGAGCGGTCGCACAAAGCCAGGAAGTTGATTGGTTTGAAGGCCAAACTGTACCACAAACAGAGACAT
GCAGAGAAGATCCAGATGAAGAAGACCATCAAAATGCATGAACAGAGGAAGACAAAACAAAAGAATGATGATAAGACG
CCAGAGGGAGCAGTGCCAGCCTACCTCTTGGACAGAGAGGGACAGTCCCGTGCTAAGGTCCTCTCCAACATGATCAAA
CAGAAGAGGAAAGAGAAGGCTGGTAAATGGGAGGTGCCCCTGCCAAAGGTGCGTGCCCAAGGAGAGACAGAAGTGCTT
AAAGTCATCAAAACTGGAAAGAGACAAAAGAAAGCCTGGAAGAGAATGGTCACCAAAGTTTGCTTTGTCGGTGATGGC
TTCACCCGTAAACCTCCAAAATATGAGCGTTTCATCAGGCCTATGGGTTTACGTTTTAAGAAAGCTCATGTCACACAT
CCAGAGCTGAAGGCCACATTCTGTCTTCCCATCCTTGGAGTGAAGAAGAACCCTTCCTCACCTCTTTACACCTCTCTG
GGAGTCATCACAAAAGGAACAGTCATAGAGGTCAATGTCAGCGAGCTGGGCCTGGTTACACAAGGAGGAAAGGTTGTC
TGGGGTAAATATGCCCAGGTGACAAATAACCCAGAAAATGATGGCTGCATTAATGCAGTCCTGCTGGTATAAGACTCT
CAACCTTTATACGGACTGTGCCTGGTAACTCTTAATTACCACAAAGAACACAAAGCTGCAACATGCAAGGGGCTTCAA
ATCATGGGATTTTCAGGAAGATCGCGGATGCAACACAACAGTGTTTGGACGTTCAGAGGAAAATAAAATGAGGTGATT
ACTGTGTTAAACTTGATATAAATAAACTTGATATGACAACCAAAAAAAAAAAA

>Sequ06081EST2
CAAGGCAGCACCATCCCCATCAACCAGGCCAGACCCAATCGTAACCTCACCTTCACCAAGAAGGAACCAATCGGAGTG
TGTGCCATCGTGATTCCCTGGAACTACCCTCTGATGATGCTGGCCTGGAAGACAGCAGCCTGCCTGGCAGCTGGAAAC
ACAGTGGTCCTCAAACCTGCTCAGGTGACTCCTCTGACGGCTCTGAAGTTTGCTGAGTTGGCAGCAAGAGCAGGACTG
CCCAAAGGTGTCGTCAATATTCTGCCTGGGTCAGGTGCTCTGGTGGGTCAGCGCCTGTCTGACCATCCTGACGTCCGT
AAACTGGGCTTCACGGGTTCCACTGAGATCGGTAAACACATCATGAAGAGCTGTGCAGTTAGTAACGTAAAGAAAGTC
TCTCTGGAGCTCGGAGGGAAATCCCCACTCATCATCTTCAGTGACTGCGACATGGACAAGGCTGTGCGCATGGGCATG
AGCTCCGTCTTCTTCAACAAGGGAGAGAACTGCATCGCGGCGGGCAGGCTGTTCGTGGAGGACACCATTCATGACCAG
TTTGTTAAGAGAGTGGTTGAGGAGGTGAAGAAGATGAAGACTGGTGATCCACTGGACCGCGCCACTGACCACGGTCCT
CAGAACCACAAAGCCCACTTGGACAAACTGGTGGAGTACTGTCAGACCGGGGTGAAGGAGGGAGCCACCCTGATCTGT
GGGGGCAAACAGATCCAGCGACCAGGTTTCTTCTTTGAACCCACAGTGTTCACTGACGTACAAGACCACATGTACATC
GCCATAGAGGAGTCGTTCGGCCCCGTCATGATCATTTCCAAGTTCAAGGGCGGTGAGGTGGACGATGTCCTGAGAAGG
GCCAATGCTACAGAGTTCGGCCTGGCGTCAGGCGTTTTCACTCGGGACATCAGCAAAGCTCTGTACGTCAGTGAAAGG
CTCAACGCCGGCACAGTTTTTGTCAACACCTACAACAAAACGGATGTGGCGGCGCCTTTCGGAGGCTTTAAACAGTCT
GGTTTTGGCAAAGACCTGGGACAAGAGGCTCTCAATGAATACCTGAAGACAAAGGCAGTGACCATCGAGTACTGAGCC
GCATCACAGCAGACAGGAAGCACTGAGGACATCAGCTTCCTGCATGCAGTGACTTCTGCATGGACACTGGCAGAAGAG
ACACGTGGAGTGATGTCATCAGCTCAGACCTGCAGCCATGCCCCAAGAGTAGTGACTACTGACTGTTGTACTTAGAGC
CTTAACATGCACTAACACCTTCATTCTGTCTAAGTCCTCTGAAAAGCACACGCCTGAGCTTTGTTGTAATAAATATTG
TCCTGAAATATGGAAATGATGTGTTTGTAGGTAGCTCAGGTAACGTCTGACACTGTTTTGTTATCAGTGTGTTTGAGG
TACAAGATAGAAAATGGAAACAGATCTAATAATTCATTTCTAATATGGCTTACATTAGGTCAGAAAAGTCTTAAGATA
TATAACATTTCTATTCATTTTTGGTTATCAATGAAAACTACTGAACACCCTGCCCCCCCCCCTGCTGTGGACCTCTTT
ATTGCTGTTGTTGTTGTTGTGTATCTTTTTCATGATGCATTTTTATAGGAATAGTTTTAACACTTTTGGAAAATGCAC
TTTCTTAACTCTCATACCTGTACGCCAAATACACAGCTGTTGCCAGGAGACAGTTAGCTTAGCATGAAGACTGGAAAC
AAGAGAAAACAGCCAACCTGGCTCTTCTAAAAGAATAAAAAAAAAAAATCCTTCTGCCAACAACTCTGAAGTTCATCAT
CACATTGTGTCGTTGTTTTA

>Sequ06105EST2
TGAAGTCATCTGACCTGCAGCTCACTGAAATACTCTACACCGTGTCTGTTGAGGGGGATGTCAAGCACAGCAATGAAG
AAAAAGGTGCTACTGATGGGGAAAAGTGGGTCTGGAAAGACCAGTATGAGATCAATCATCTTTGCCAATTACATAGCT
CGAGACACACGCCGCCTTGGAGCTACAATTGACGTGGAGCACTCCCATGTACGGTTTCTTGGCAATCTGGTTCTAAAC
CTGTGGGACTGTGGAGGACAGGACACGTTCATGGAGAACTACTTCACCAGCCAGAGGGACAACATTTTCAGAAATGTA
GAGGTGCTTATTTATGTATTTGATGTTGAGAGTCGCGAGCTGGAGAAAGACATGCATTACTACCAGTCGTGTCTGGAA
GCCATCCTGCAGAACTCCCCTGATGCCAAAGTGTTCTGCCTCGTGCACAAAATGGACCTGGTGCAGGAAGACCAGAGG
GACTTGATCTTTAAAGAGCGTGAAGAAGATCTGAAGAGACTGTCCAGACCTTTTGGCTTGCACGTGCTTCAGGACATC

GATCTGGGACGAAACCCTGTATAAGGCCTGGTCTAGCATAGTGTACCAGCTCATCCCGAACGTCCAGCAGCTGGAGAC
AAACCTGAGAAATTTTGCACAGATCATTGAGGCAGATGAAGTTCTTCTGTTTGAGAGAGCCACCTTCCTGGTGATCTC
TCACTATCAGTGCAAAGAGCAGCGCGATGCTCACCGGTTTGAGAAGATCAGTAACATTATTAAGCAGTTCAAACTCAG
CTGTAGTAAACTTGCAGCCTCTTTCCAAAGCATGGAAGTGAGAAACTCCAACTTTGCGGCCTTCATTGACGTCTTCAC
CTCCAACACATATGTCATGGTCATCATGTCAGACCCATCCATTCCATCTGCAGCCACTCTCATCAATATCCGTAATGC
TAGGAAACACTTTGAGAAGTTGGAGCGGGTGGATGGACCCAAGCACAGCCTGCACATGCGAATGCGCTAGCTGGCGCA
TTTGTCCCAGTGGATACTCTCAGCCAATCAGTGCACAGACCACAGAAGGCATGTTGCGGCCTTCCACCTTGCCGATAC
ATATGAGCAGCCCTGCACCGTTTCTCTATATTTTCACATTCAATCACAGTTATTACGAGAGAAATCATATTTTCTTTT
GGTCTAATTTCTACAGTATCTTGAACAGTGATCCTTTCTGTTGAAATATTTGTCCTAACTTGATGCATTAAAGAAAAA
ATTTATAGTTGTGCTTATTATGGAGGCGGCTACCTTTGCTGTCACACTTTGTCATACATGGTAGATACATTCACTTCA
TGCAATGCGTGAATCCCCGCAATCGGGGGATGTTATCCCCAAATTTGATTTTCAGATGCAGTTTGTAAGCTGTTACTC
TTTGACAGTACAAACAATATAGTTATATTCAAAAAAATATCATATGTGATGTGTTTTTTCTGCTTTTATATTTTGAAA
GTCCTCACCACTTGATCCACATTTACAAAAACATCATATTTATGGTGGTACATGTTATAAACAGATCTACATATTGGT
ACATATTTACTTCCTGTACAAACTATCCTGCTTTGATAAGGTTCATGTTATTTCCTCTATTCACTACAATGCCCGTGC
ACATAAGACCCTATTAACACTCTGCACCAGTTGTCCTGTACACATGGCACATGGTGGCAAAAGTGTGTAGGGGGTTGG
TTGGAGCTGCGTGGGAGGGGACATGATAATGGGGTGCACCTGTTAAGCAAGCTGAATGGAACTGACAACAGTGTATGT
GCAGATGGAGCAGCATGTTCATTTCAGTGAGTTTAACATGACAGCTGCATGTAGTGAAAAGTGAATGTCCCATGTTGG
TACTGTATATAAGTATTACTCTGCAACATTAAATATTCACAACTACATAAGC

>Sequ06106EST2
AGCTGATCTCTGCCAGAGAGAGGACGGAGAAAGATGTCGCTGACAAACTTGCGAAGGATGGAGGCTGTTTTATCCGTG
GCGTTTGTCCTATGCTTGCTTGCTTCAACCGGTGTACATGGGGACAGCTTAACAGTGCTGCAGGCTCCAGAGTTTGTG
TCCTTCCAGAAAGGAGACTGGCCTGTGTCCGGAGAGAAGATCCCTGACCTGGTGGCTTTGACCATGGGCTTCTCTGTT
CAGGAGGATCTGTCCTGGCCAGGCCTCCAGGCTGGTCCGTTATTCCAGCGTCCCCGGGCTAACGTACTTGTGGTGGTG
AGAGGAGTCGATAGCTTGGCCCTTCCTCAGAGTGTGGCTTCCTACCCGTTGGAGAATCCAGTACCCTTCACCTTGGAT
AGTGTTGCAGAGACGGTGCACTCTCTGTTTGCTGAGGACACTCCTGTAGTGCTGCAGCTGGCCCCTAGTGAGGAGAGG
CTGTATATGCTGGGCAAGGCCAATGCAGTGTTTGAAGACCTCCCAGTCACCTTGCAGCAGATCCGTGCCCGCCTGTCC
CAGGATGGCTCCGTGCTGGCCTCCCTGCCCCTCAACTCCCTCAGCAGAAATGCAGAGGCTGATTTGCTCTTCCTGTCT
GAGGTCCAAGTGCTGCATGATATCACAGCTCTTCTGCAGAGACACAGACATTTGGCAAAGGACCACTCCCCTGACCTG
TACTCCTTGGAGCTGTCTGGCCTGGAGGAGCTGAGCCGGCTCTACGGCCAAGACTCCCCACAGTATCGTGATGCAACT
GCCATTCTCGCCTCTGTCTTGCAGAAGTTTGGAGAGGATGTGTATGGTCTCTATGGCAACAGTGCTGTGGTGGAGGTT
GTGACGGTGAAGACTTTTGAGGCTCCTTTGACCAGGAAGTCCCGTTCGATCCTGCAATCCCCACAGATCAGTAACCCA
GGCAGCCCATACAACCTGGCCTACAAGTACAACTTCCATTATGCTGTGATCTTCAACATCGTGCTGTGGCTAATGATT
GCTCTGGCCCTCGCTGTCATTGCCATCGCTTACAACCTGTGGAACATGGACCCAGGATATGACAGCATCATCTACAGG
ATGACCAATCAGAAGATCAGGATGGATTAAATCCATCAACACTCCAGCTGTGCTCTAAGATCCCTGTTGTGAATGTTA
GTGTTTTGTGTGACGTCCTGTGTAGTGAAAGAACTGTGACAGCTTGATGGGAGAGAGCGTAACACTGAATCACCTTTT
TGTTTACAGCACTGATAACGGACAAAAAATAACAACATTACAGTAGTTGGCTTTTAATTTGCCTACATAAAGCAATTA
GATATCATCCTCCACTGGTGTGTACACTGAACAAATTCAGACTTCCTCCTTTCAAACACGCAGCTCACATCATTCCTT
ATGACTCTTCACTATGTTTAATTTCCATCCTGGTGATTGCAATTGTATTTTTTTATGTAAAAAAAAAAAAAAAAAAAT
TTTGTAATGTCATGAAAGAATGAGATTTGTCTGGTTTCTGTGTGAAGTTGTTGTGCTTGTCTTGATATGTGAAACTGT
TTTAAGTTAAATTAAATCTATGTGTATATATTGAAAATAAGAATACAACTGTATGAGAAACTGCTGTACTCTCATTGT
GAAAAGATTTGTGCAGTGTTTATTCCAGGTAGGTCCTGGTACCCCGCTTCTATTCCAAATGATAACTACATATTGGAT
TACGTAAATACTTGATCGTAATGTAGGTTGTTAGGATTGGATATGGATAAATGAAGCTCTGTAAATCGCAATAAAATG
CCTGACAATAAATTATTACATGGATCAAAAAAAAAAAAAAAAAAACAAAAAAAAAAAAAAACTAACGTAGACTACGT

>Sequ06109EST2
AGGGTTGCGATGTGCTGGCCAAGTCTCTGGAGAAAATGGGGTACAATGCGTGTACGCTGCACGGTGGCAAAGGCCAGG
AGCAGAGAGAGTTTGCGCTTTCCAATCTCAAAGCAGGGGCCAAAGATATTCTGGTGGCCACAGACGTTGCTGGTCGAG
GTATTGATATCCAGGACGTCTCGATGGTCATTAACTACGACATGGCTAAGAACATTGAAGACTACATCCATCGTATTG
GTCGTACAGGTCGTGCTGGTAAGAGTGGTGTGGCCATGACTTTCCTCACCAAAGAGGACTCAGCTGTGTTCTATGACC
TGAAGCAGGCCATCCTCGAGAGCCCGGTCTCCACCTGCCCTCCAGAGCTGGCCAACCACCCAGACGCTCAGCACAAAC
CGGGAACCATCCTGACCAAGAAGAGACGCGAGGAGACCATCTTTGCCTGATTTACTCCGACTGGTTCAGACTGTTTTG
TCATGTTGCATCTTCACACAATATCCGATTCTCTTTTTCTGCAGTCGAGATATGGTCCACAAGTTAATTCTGCCCATG
TCTTCAGTTGTTTCACACCTTCCCTCCGATTCTTGTCGGTCTGTTTTTGTCTGTTGGCCCAGTGTGCGAGTAGAAACA
GACACATTAACTGGTGCAGAGATAATCACAGCACTGGTTTTGAGGTGGCTCTAATGTGAACACTTCTTATCGATGTCT
GTGAATTTGAATCTGTTTACTCAGTTGGAAAATGTGTTAATGTAAGAGTCAGAGGTAGAAATTTGTTAAACTTCTGTG
AATAAGAGCGCAGGTCCAGGATCACTTAGCAGGATGCTGATGAGCTGGACTGTAAAGATGCTGCTCTTCAATAATTAT
CAAGTGTGATAAATACCAGCCAAGATGCAAGTGTAGATGAGTAGAGAGTACTGTGTTGTGCATTCTTATTAACCATTA
CCATTTGAGTTTCCTGACTGTTTAAGAGACCAATGCCAGAAGTAATGGCTACTGTTTTCACTTCAGACACATCGACTG
GCTAAATTGCAATTCGTTGTTTTTAAATATAAACATTTGTATGAAGTCAATACAGCAAATTTGAG

>Sequ06111SNP2

ACGCAATATTAAAGAAATCATGACTGTATGAGTATTTGCTCTTACCATTAAAATAAATATGATGATGATCTTTAACTT

CTGTTCCTTTCCTTTTCAGTGCCCATCATTGGAGCAGTATGCCATGAGGTCGTTTGCTGATGCTCTAGAGGTAATCCC

AATGGCACTGGCTGAGAACAGTGGGCTTAACCCTATCCAGAACATGACAGAGGTCAGAGCCAGACAGGTCAGAGA

>Sequ06152EST2
TTTATCATATGTCAAGCTAGCTCCCAGTGATTAGCCACGTCATGTAAACTTAGCAGTGGAGGAATTGTGATATCAACG

TTGTTGGGGGTCGCAGTTTTTCGGGGGATAGTGGTTTATATTCGACAAGGAGCTCTTGTTTCAGGGTTATAGCTGGGC
AGTAATCTTATGGAAAGATGTCAGATATCGGGGACTGGTTCAGAAGCATCCCTTTAATCACCCGGTCCTGGTTTGCTG
CCTCGATAGCTGTTCCCTTTATTGGGAAACTAGGATTGGTTGATTTCAGGCACCTCATGCTGGTCCCGGAGTTGGTGT
ATAACAAATTTCATCTCTGGAGACCAGTGACAGCCACCCTGTATTTTCCGATAACTCCTAACACTGGGTTTCTGTACC
TGGTCAACCTGTATTTCCTCTACCACTACTCCTCTCGGCTAGAGACAGGGGCGTTTGATGGCAGACCTGCAGACTATA
TCTTCATGCTCCTCTTCAACTGGATCTGCATTGTTATAACGGGGATGCTGATGAACATGCGGCTGCTGATGATCCCAC
TGATCATGTCAGTGTTGTACGTCTGGGCTCAGTTCAACAAAGACATGATTGTGTCCTTCTGGTTCGGCACACGATTCA
AGGCACATTATCTACCTTGGGTCATCCTGGTCTTCAACTTCATCATTGGAGGCTCTTTTGTCAATGAACTGACAGGGA
ACCTGGTGGGTCACCTCTACTTCTTCCTCATGTTCAAATACCCCATGGACCTGGGTGGACGCTCCTTCCTCTCTACAC
CAGAGTTCTTGTATCGGTATTTCCCTAACAAGAGGGGAGGGGTGTCGGGCTTTGGAGTCCCTCCCAGCAGGAGACCAG
CTGCCCAGGAGCAGGCGGGAGGAGGAGGAGGAGGCGGCGGTGGGGGAGGACGCCACAACTGGGGCCAAGGCTTCCGCC
TGGGGGGAGAATGAGAGGAGGCAAGAGGAGATGGCGACACCCTCCTCCTTAGCTCCCAATAACAAATGGCCGTGCAGC
TGATGGTGCAGGTTAAGATGCTTGCTTCTTCACATTCTTCTCTGTTTGAGGGGATGAAACCTTGAACCTCATGTTTAA
TTTAAAGGTTATAACAGGATGAAATCTGAGGAAGCACGCATTTTAACTAAGACAGCAGCACACAGCGGGACTCAGACT
CTGGCTATAGGCTCAGTTTACCAAGTAAAGCCAGCTCTACTATTAACTACTGCGGATGTGTCCATGTGAAGCAGATAG
AAGAAAATCTGAATAAATCATTTTTTTATGTGATGATTCTTGATCCCCTCTCCCCTTTTCTTCTTTCTTTCTGTACTC
TTCTTTCCATCCCTCCTCCACCTTTATTCCACACCCTGCCATTGGACTGTCAAGACAAGAGATGTTGCAGCTGCACAC
CAGCACGCTGTTGACTCTGACTCTTGGGTTCCTGTCCTCTCTGTCTCTGTGTTGCCATATCAGATTACATTGTAATTA
CCAACGCACTGGGAGTGTGCTCTCAGCCTCCCTTCACAGAACATGGGCAAAGCTATTTAGAGAGCGCTGTTTCCAGGT
CTGAATTTACATGGGTGTAAGATGTTTTTAAGCCCCTGTGGCACAGACCTGATCTTTCTCTGTCTAAAGATGTGATCT
TAACCAAAATGAGGTGAAAATGGGATTTTGCTCTTTTTTTTTTTGAAAATATAACAGTTTGTTTATTGAAAAGACAAG
TGAGTCTGAGTGTATGTTTATCCTGCTATTCATTTTTCTAAGCCATTCTAAGAGTCAGGACAGCTCAGCTAATCACGT
CTAAGTGTAGTACAATTGGGGCTGATACTAACGTTTATTTTCGTGATCGATTAATCTTCCAATGAATTTTGCAATTAT
TGTTCTATAAATTTCAGAAATTTGTGAAAAAAGTTTTGTTTGTTTGTTTGAACAACAGACCAAAACCCTGAGATATTC
ATTTCACTGCCATAAAACAAAGAAAAGCTGCAAATCAAGAACAATTTAAAAGTCTGTTTTTCCATTTGTGCTTGAAAA
TTGACTTGAGCA

>Sequ06181SNP2
GTGAGTGATCGGCAGAGTGATGCGGCAGAGCGGTGCAACAGAGCATGTGATTGGAAATATTAGAGAGCTTACATGGTT
TTGAATGTTTATTTTTTAAGTCAGTTTGTGCGAGCAGTTTCACTACATCTATCAAAAGAGCTCACATTGCTTGTCATC
CTGATTTATTCATCCCTTATTGGCGTGACATTATAAATGTGTTTCTTGTGTTGACCGTATTTCTGTGTTCCACGTTGC
ACAAAATAACTCTAGATGCATTATTGTTGTACAAAATGTAATGTCTGGTTTGTTCTTATCTCGTATTTTCGAATGGAG
TATTTTATTTATTTTGGATCAAAGGGAAAGTGTTGATTTTAGTGTTGTTTTTTTCTTAATTTGCTAACTAGATCAGAC
TGTTGTCTTTTTGAGTGTTCACAAATCACATAATTTGAAAACATAGGAGAGAGGATGATAGTAATAATGCATCATAAG
GCATGTTAATATTGTACAAGATCTGTAATAAAAGTGAATTATAATAGCCTGTTTCCTCAGACTAAATTTGTTAGTCTA
AATTACTGCTGCTGGTTGGAACAATGCTGTACTGATAAATGTTATGGATTTAGTGTAAAGTTTACCGAACAACCTTCT
AATATGCAAAAGCATGCTTTTCTATGTCAGCGCAACTGTATATATGTTTATGTAACAAGAAAAGTATATCATGGTATT
CGCGGTCAAATTGAAAGTGTTGTTGATACACCTGTAGATATCGGGTTCAGTGTGTTGGCAGACGTGTATAACTGACAG
CAGCTTGTGTGTGTGACGTGCCCTCTATTACGTTCAGTTTGGTGTGAAAATTGTTTTTGTGGCCTTATATTGCCAGTG
GTTAATTTCTCTTTGATAACAGGTTTGTATGTAGGAATGTCCAACATTCAAGTATCTCACCTATGATCTGTAACAACA
CTCGCAGTACTGTTGTGGCTAAACATCCCTCTTTCAGTGACTATTGGCAGAAAAGCAAGACTGCACAACATTCCAGCT
GTTAATGTGTTTTCAAAAAGGACAATGAATTTATTAAGTGGAGGAAATTAAAGCAACACAAACTGGTGCAAAAGTGTT
GAGTGATTTGATGATTTCTGGCACTACATCGATCGGT

>Sequ06191SNP2
GCGAATTGAAATCGAGGAGAACAAGAAGCAGGGTAAATATCCTGAGGTGCATATTGGATTAGAGAACCTTCAGCACTT
CCCAGCATTTGGCTCTCCACCTCACAACAGCAGCCCCCCGATCCAACCTGACTTCAGTTTTGCCCCTCCGTCACCTCT
CAGCAGCAGCCCATCCTCTGATGGGATGAGATTCCCGAGTCTGAATGGGCAAAGCCCTTCACCCGTTGTGGGTAGTGT
GGAGGACGACTCCCACTGCATGTCCTTTGCACAGATGCTAAGAGATGGAAAAGCCAGAGCTGATGCTGGGCCCAGGAT
CAACTCCAAAGAAAAGATAAGCTCCTAGCTCCCCCCAGCAGCAGACAGTGATGGAGAGAGTGATGGGTCGGACCGTGT
CCCGGTGCCCAGCTTCCAGAACTCCTTCAGCCAGGCCATTGAAAAGGCACTTCTGGAGCTGGACAATGGTCCAGCTTC
TCCTCCACAACCTGTGGTTGACCCAGATGAGAAAGGAGGCAAGAAGAAGAAGAAAAAGCAGAAACTTTTGTTCAGCAC
ATCCATGGTTCACACAAAGTAGACAACACTGAAGTTCATTTAATCATATCATTTCTTGAGTTGATCTTCCCACTGTTT
GTGTTTTTACTTCCATGCAGATCTTTCTTGGACTTACAAGCTATGGAAATAGGTTTTAGCATGTAGCCAAAGTTCATG
CTGCTTAAGTCATTGTTCCATTGGCTTGATTCATTGTTTGGGTGTCATGCCATTAGTTTAATTTTTTGTACAACTTGG
TCAAAGCTTTGCAGCAACCACCTGCCACACTGAGAGCCATATGGGGAACATTTAGTTCAGATGCTATGTAAAGCAAGG
AATAGAACAAGCTGCTGGCTCTCATACTATGTTTGCTTCAGGCTTTTCATGGATTCCAGATGCCTGGCTTGCATCACA
GTGGTTGCTACATGATTTCCAACTTTCCTCAAATTGACCTTGAGCATTGCATCTGGGACCCATGAGTAATAAGGATAA
GATAATTCGAGGGTGCTTTATATTACAGTAGCAGTCAGCCAGCTGCAGGTACGTTGCACCTTTGAATCTTTCCAGGAT
ACTCAACGCACAAACATTATAACTCTGGAACGTTATGTTACAGCAAAAAAACACTTAATTTTGTTTTCTCACAGGTAA
TAATTTAGGCCTATTCTTCATCAGCTTTCAAGAGGATAGATTTTGTGGATTGCTGCTTTGAGATCTATGCCCTAGTAT
TTTGTAACAACAACATGAATGCATAATGATCTAATACCATTGTGAGGAGGTGAAAACACAGTATTGTTTTAAAGAGAT
TAGTTCTGTTGTTTGAACAGAACTCCAGTATACTCTGCTTGGTTAAACCACAAATACACATTTTCTTCTCATTCATCT
TCCGTTGTAGAATGTTAACTAACTTTTAACATTACTCCCCCAGTTTAATTTCAGTGTTGGAAATGTGTGAAGTTAGTG
AATTGCTGACATTGAAAGGATGCTTGTGAAACCTGAAGAAAAGTAGAAAATTTAAATTTTTTGTTTTGAGGGTTCTAT
ATTCACCTTTATGTTTGAGCAAGTGTATAGACTAGTGGACATAACTAGTCTGTTGAAATGAATAAACAATTATGCACA
AATAAAAAAAAAAAAAAAAAAAAAAAAAACAAAAAAACCCCGAACCCTAACT

>Sequ06210EST2
GGTGCTGGTTCGTCAGTTGGAGCCCGTCTGGGACGCTGCTGGCTTCATNCGGGGGCGATAAGACCATCCGGATATGGG

GACGAGAGGGTGACTCTTGGATTTGTAAGAGTGTGCTTGAGGACGGACACCAGCGCACTGTGAGGAAGGTGGCATGGT
CTCCCTGTGGGAATTATCTGGCCTCTGCCAGCTTTGATGCAACTACATGCATCTGGAAAAAGAAGAACGATGGGTTTG
AGAGTTTGACTGTGCTGGAGGGACATGAAAACAAGGTCAAATGTGTGGCGTGGGCCCCTTCAGGGAACCTGTTAGCAA
CATGTAGCCGAGACAAGAGTGTCTGGGTCTGGGAAGTGGATGAAGAAGACGAGTATGAGTGTGTCACTGTTGTGAACT
CCCACACACAAGATGTCAAGCATGTTGTGTGGCACCCAACCCAGGAGCTCATGGCTTCAGCCAGCTACGACAACAACA
TTTGTATTTACAGGGAAGAGGATGATGACTGGGAGTGCCGGGCCACTTTGAAAGGACACACATCCACAGTCTGGAGTT
TGTCTTTTGATGCGACTGGACAGAGACTGGCCTCCTGCAGCGATGACCGCACCGTGAAGATTTGGAAGGAGTATCCAA
ATGAAGCTGGACAGGGAGACTTGTCATGGAAGTGTGTTTGCACTCTGTCTGGGTACCATGGACGAGCAGTGTATGACA
TCGCCTGGTGTCGGCTGACTGGGGCCTTGGTAACTGCCAGCGGTGATGACTCAGTGCGAGTGTTTAAGGAGGACGAGT
CGTCTGATCCCGACCAGCCGGTGTTCTCGCTGGCTGCTCAGGTGACCAAGGCTCACAACCAGGACGTGAACTGTGTCG
CCTGGAACCCAAAAGAGGCGGGACTGCTGGCCTCCTGCAGCGATAACGGAGAGATCGCCATCTGGAGATTCCAAGAAG
AAGACTGAATGCGTGGTCGCTCACTGAACTAACAGTTACTGTAAGTTACACTGGGAACTTAGTGGTCCAGAGGTCACC
ACAATATTGTTCATTCAACTTTCACAGCAAGATTCTGCTTAATATGGTGTTTAAACATGTCACCTGTCATCTGTCATG
TCTGTTTCTAAACTGATAATCATGCTTCATTGCATCTTGCGCTTTGAGTCTAAATATTTCTTTCTAAAAGCTCAAGTG
GGAATTTATTCATTCATGTTTATATAAATAAATAAATAAAAC

>Sequ06215SNP2
TTCCCAATGGAACACATAGGATGAAGATGTTTCCCCACTAGGCACAGATCTCCTCTAAATAATCAGAGTAATTTTTGT
TAGCCTGTTGCGTTACATGTACAAGGAATTTGTCAGCAACAGTTACCTAATGCCAATACAGCCATTGTAGAAGTCCCA
CTCTTCACACCCTTGCTGCATTGGTGCTGCACTCTTAGTTCTGCAATTTTGGGTCTTCTCCTTAAAAGGCATTGCTTT
GACAGTTGTGTAGTCTTAAGATGGTATAAATGGGCAGAGGGTCTTCTAAAATGGCTAACTTTCTTGGATTAAATGAGC
TGAGAAATTGACCTTAGATCTGAGTCTGTGGGGGGAACTCTCACTCCACCAATGTGCTGACCAATACCACATCATTGA
AACCAGCCAATCACAGCAACCCGTCAATCAGGCCAACTAACCAACCAATCACCTCACAGTGATGAGCCCAGGCTCCAG
CCTTCTACCTATGATCTTCACAGACCAAAAGAACCAATCAACCAATCATGAGGTGGACACCAGTTTCACATTATCACC
ACAAGCCAAAAAAAGAACACACAGACATTTTCTAAATTGCAAGAATCAGGTCGATGCGAGTGTATTACTGAGACTGTT
GCCAAAGTGCACTTGTACAGCGTGGACCAGAGAAAGTAAAACTAGCCATTTACTTGACAGCATTAACCAATTATGAGC
CTAAATGCTGAGGTTACCAGCAACTTTGACAGGCATTCCATTTGACCCTTACCCCCATATCCCCACCACAAATGTCCA
CCGAAATTAGAGAAGTAGTGATTGATCGGCCTGCAATCGAAATCTGGAAGTATATTCACTCTGGAGAACAAACTGGAC
ATTTTCAGTTTTATAGATGCCCTATAAGGAGAACAATAAGATGTAAAACATGACCCATTTCTGTGTAATGATGTCTGT
CATTACCTAAGTAGCTACTTGATGCATGTTGTTAATGTCATGACTCAGGATAGCTACCTACAGTTATAGCAGGGCCTA
AACCTACTTAGATTTTGCTACAGTTTCCTCCTAAGTAACTGGTTCTTACCTGTTATTCAAGAAAGAGCCTACCTCTTC
TCTGCTCTGCTCTTCTAGCAATGTTAACGGATTTTAGTGCAAACTCAGATTTGCGTGACATGGATTTATATAGTATCT
ACCACACACAAGCGCCAACTGGAATGAAAATGCAAGAGAAACTGTAATGAAATCTTAAGAAAGCTAACTACTGTGAAG
CAGCTGCAGGATGCTTTGTGTTTACATTAACATTAACCTTAACATCATAAGGAAGTAGCGTTCTTCATTACAAACTAT
AAAAAACATTGCAGAGAAGAGCAGTTCGGAATAGTGTGAGGCTCTTAACTGTAACATGAAGGTAAAAACATGGACAAG
TGCACAAGTGCAACAATGGCCAATTCTACATTCCATAGAAAAGTGATAAAACAAATACTGTATTTCAACCATGTGACG
GTTTATCAGTCTACAGAAAATGCAACACATTTAATTTCTACAAGCCAGGATGTATACAGATATGGATCCTGGCATATA
AGAAATCTAGAAATGATACATATCAGCACACACACAAAACACACACCACCCTTTTCCTTAATCACCCATTCCCTTATG
CTCAAGTGACATTTGTTGCAAAACACTAAAGTTCAGGTTTCCAGAAAAGAAAGAAATTTATCTTGCCAAGAATGAATG
ATCTCCTATGTTAATTTAATTAAGTTTAAAAGAGTTTTATTTTATTTTTTTCTCTTTTTTTTTCATGAGTAAATTGTT
CAAATTGTACAAATCTTTAGTCATTGAGTGGACTTAGGTATTAGGTGTGGTTGTCATAGTAAATAAAGATGTAGACTA
TCT

>Sequ06222EST2
CTCGGTTGCGTGGTGGTCCAAAATGTCGCTATTAGAGGGTCCGTTAAATGTGTTCGGCCAGAGAACATCTGGCGAGTC
TGTTCGCACTCAAAATGTCATGGCGGCATCATCCATCGCCAACATCGTCAAGAGCTCCCTCGGACCCGTTGGCCTCGA
CAAGATGTTGGTGGACGACATCGGGGACGTGACCATCACCAACGATGGAGCAACCATCCTGAAGCTACTGGAGGTGGA
GCACCCAGCTGCCAAGGTCCTGTGTGAACTGGCTGACCTGCAGGACAAGGAGGTCGGAGATGGGACCACGTCTGTGGT
AATTATTGCTGCAGAGCTGCTTAAGAGTGCAGATGAGCTGGTGAAGCAGAGGATTCATCCCACCTCAGTCATTAGTGG
ATACCGTCTGGCTTGCAAGGAGGCAGTGCGCTACATTAATGAAAATCTCACTATTGGAACAGATGACCTGGGCAGAGA
GTGCCTAATCAATGCAGCCAAGACATCCATGTCCTCCAAAATCATTGGAGTTGATGCAGAGTTCTTTGCTAACATGGT
GGTGGATGCTGCCATGGCTGTGAAGTTTGTGGACAGCAAAGGGGTGGCCAAATATCCCATCAACTCTGTCAACGTGCT
GAAAGCCCATGGCCGCAGCCAGAAAGAGAGCTTCTTGGTTAATGGATATGCACTGAACTGCACAGTTGGCTCACAAGG
CATGGTAAAGCGTGTTGTTAACGCCAAGATTGCTTGCCTGGACTTCAGCCTGCAGAAAACCAAAATGAAGATGGGAGT
CCAAGTCGTTATTAGCGACCCAGAGAAGCTTGACCAGATCAGACAAAGGGAATCTGATATCACCAAGGAGAGAATCCA
AAAGATTTTGGCAGCAGGTGCCAATGTGATCCTGACCACTGGTGGCATTGATGACATGTGTCTTAAGTACTTTGTGGA
TGTAGGAGCCATGGCAGTGAGAAGAGTTCTTAAGAGGGACCTCAAACGTATCGCCAAGGCAACAGGAGCCACTCTTTG
CCCATCTCTGACCAACCTGGAGGGAGAGGAGACATTTGAGGCCACTATGCTTGGCCAGGCTGAGGAGGTTGTGCAGGA
GCGCATCTGTGATGACGAGCTCATCCTTGTCAAGAATACCAAAGCACGCACATCAGCTTCCATTGTTTTGCGTGGGGC
CAACGACTTCATGTGTGACGAGATGGAGCGCTCGCTGCATGATGCTCTGTGTGTGGTCAAGAGGGTGCTGGAGTCAAA
GTCTGTAGTACCAGGAGGAGGCGCTGTGGAAGCAGCTCTGTCAATCTACCTGGAGAACTATGCTACCAGCATGGGTTC
CCGAGAGCAGTTGGCCATTGCCGAGTTCGCTCGGTCTCTCCTTGTCATCCCCAAGACGCTGGCTGTGAATGCTGCACA
GGACTCGACTGATCTGGTGGCCAAGCTTCGCGCCTTCCACAATGAGGCTCAAGTTAATCCTGAACGCAAGAATCTCAA
GTGGATTGGTCTCGACCTGGTTAACGGCAAGCCAAGAGACAACCGTCAAGCTGGCGTGTACGAGCCCACTATGGTTAA
GACAAAGAGCCTCAAGTTCGCCACTGAGGCTGCCATCACCATCCTTCGTATTGATGACCTCATCAAACTGTTCCCAGA
GCCGAAGGAGGGCGGGCAATCGTACAGGGATGCTGTGGAATCAGGATCTCTGGAGGGTTAAAAGGCTGCGACTGGTCC
CAATAGGTTCAACGTCTGTATTTATACATAAGGAGCTCTTCATGGCTGTTGCTCTAGTGTAGGCCGTGCACTTTGCCT
GTTTACTGTTCTCCCACTCCCTCTCCCTCGGGGTCTTGCTTCCAGAGGAACTGTAAGTGATTTGTTGGATTTTTTTGG
ACACTTAATAAAGCTCCAATCATTGTAAAAAAAAAAAAAAAAAAAAAAAAAAAAAAAANAAAAAAAAAAAAACCGAAACCT
ACCTAACTAAGT

>Sequ06243EST2
GCCTGATCCTTGCATTGAGCCTGTCTGCAGGCACAGGAGACTCTTTCTGGGTTATTGGTTCATTGCACAACAGTTACA
ACATTTAAATTAAAGCGTAGTTACTGGGAACAAAAATAAGACCCGAAGATGACCTCCAAGAAAGTGATCGAGTTGTTC
TACGTATGTGGTTTCTCCGTACTCCCTGGCTCGGCTTTGAGATCATGTGTCGCTACAGGAACGTGTGGAACATAGAGC
TCAAACTGTGCCCTGCATTTCTGGGTGGCATCATGCAAGGATCAGGCAACAAACCTCCAGGTCTGGTTCCAAACAAAT
TCATGTACATGACCAAGGATCTGAACCGCTTTGGCTCAGTATTTTTGATGTTCCCCTGCAGCCTCCGTCTGATCCCTT
CGAGGCCATGTTCAAAAAAGGCTCCTTGTCTGCAATGCGATTCGTGGCAGCAGTACAAGAGAGGGAAAAGGGCGGAGA
CAAACAGGTGGAGCAGGTGTCCCGGGAGCTGTGGAGAAGGATCTGGAGTGAAGACAAAGACATCACAGAACCCGCATC
ACTGTCTGAGGCAGCGAAGAAAGCGGGCCTGTCTGACGGTGAGATTAAAGAGGTGCTGCAGATGTCCACCTCAAAGAA
GATCAAAGACAAGCTGAAAAGCACAACACAGGATGCACTTGATATTGGGGCGTTTGGCTTCCCCTGGTGGTTTGTCAT
GTTGAGGAAAGCCAGAGATGTTTTTTGGATCTGACAGATTCGAGCTGATGGCCCACTGCATTGGAGAGAAGTGGCTGG
GGCCTCATCCTGGCAAATCAGCTGCCAAGCTTTGAGACGGAAGCTGCCAATTATCTCAATTTAATGATGTCTTTCATT
TTCTCTAATACTAATACTCTAATTTGTATGCTGTGCCTCATTAACAAATATAACATGTAAATAATCTAACATACCACT
GAACTGTTTGATATGTGCCAATTAAAAAAGAGTG

>Sequ06257SNP2
GCTGGACGGTGGCCCAGCTTCGATCGACGCGCTGTCTGAGATCGTGGACACGGTGCAGGGCAGGATCGAGATCTATCT
GGACGGAGGCATCAGGACAGGAAGTGATGTATTGAAAGCCCTGGCCTTGGGAGCCAAGTGCGTTTTCATTGGCCGTCC
AGCAGGGTGGGGCCTTGCATACAAGGGTGAGGAAGGAGTGAGGGAAGTACTGCAAATCTTAAATGACGAGTTCCGTCT
ATCCATGGCTCTATCAGGTTGCAGGAACGTGGCTGAAATCAACAGGAACTTGATCCAGTTCTCCAAACTCTAACTACT
CCAGCAGAGGGCAGCACTGAGGAGGACCCACTGAACAGGACTCACTACTGACTCAAACCAAAAGTGTTGAACAAGAGC
CTGCATCAGACTGCATCAAGTCAAAGTGAAAGATGTTCACCTCAGAACGCATGTCTTAATCACGTCAAAGGAAACATG
ATACATGCAAGCAGCTTCCATGATTTGGAAATGACATCCACTGTGTATTCATGCCAGAGTGTATGGGTGCGAGATTGT
CCTGGGGAAGCTGAGGGTCGGCAGCAGATTTAGCAAGTGAAAAATGAAAGTGTTTATGCTTTTGGATTAGAGGGGGAT
GGAGGCACTTAATGATCATGAAACCAACAAACCTCCTTTGGTTTTACTGTAACGAATGCAACAGATGATGACATACAC
GACGGTTTGAAATGCTGCCAGTGCTTGTGCTTTAATAAACACCTGCTGAGGGTGATGAAGACTTGTTGCTACCTTTCT
GTATCTAGTTAAAAGCAGGTAGAAAACAGGATGTAGATGTAGCAGACGTCTGTCTTTTGTTTTGTTTCATAAATTTAT
ACATACATTTAATCCAGGGAAGAAACATGACACAACATCTTTACAATACACGGGTTTAAGGGGATTATCTGAATGAAC
AGGGCAGGAAAACTAACCTGGTCAGTGCTTGAACCTCGCTACAGCTGGAGGCCAGATTCCCACCAGCTTCTAGAGAAC
AAGAAGAACTATCAAAAAAAAAAAAAGGTCAAAAGATGATTTTGTCTATTGTTTTTGCATTGGATTTTCAATCAGGAAA
AGACATGAAAATACTAATAAAACATGTAAGACTGAATGATAAACAGTTTTGAACAAAAACAAAAAAAAAAAAACCGAGA
ACT

>Sequ06259EST2
AATATCAAAGAGGAGGAGACGTGAGCGAAGCGTTTGTTCCGGGCAGCAGCGTGTTTGCAGTTTTGCTGCAAAAATTAA
AAGCCAGTAACGTGTGTTGTTGGCCTCGACATGGTGAAATTATCCGCGGAGCTGATTGAGCAGGCTGCTCAGTATACC
AACCCTGTGAGAGACAGGGAGCTGGATTTACGAGGTTATAAAATCCCAGTGCTTGAAAATCTTGGAGCTACACTGGAC
CAGTTTGACACTATAGATTTCTCTGATAATGAAGTCAGAAAACTGGATGGCTTCCCTCTGCTCAAGAGACTGAAAACA
CTTCTAATGAACAACAACAGGATTTGTCGCATAGGTGAGAATCTGGAGCAGTCTCTGCCAGGTCTGACAGAACTGGTC
CTCACAAACAACAACATTCAGGAGCTTGGTGACCTGGACCCACTGGCCTCGGTGAAGACATTGAACCTTCTCAGCCTG
TTAAGGAATCCAGTGACAAACAAGAAGCATTACAGGCTCTATGTCATCAGCAAAATCCCACAGATCCGTGTGCTTGAC
TTCCAGAAGGTGAAGCTCAAGGAACGCCAGGAGGCGGAGAAAATGTTCAAAGGCAAACGAGGTGCTCAACTTGCAAAG
GATATTGCCAGGCGAACAAAAACATTCACTCCTGGAGTGGCAGCACAGCTTGAGAAGAAGAGGACGGGGCCGTCACAA
GCAGACGTGGAAGCTGTCAAGAATGCCATCGCCAACGCTTCATCACTGGCAGAGGTGGAGAGGTTGAAAGGGATGCTG
CAGGCCGGTCAGATCCCAGGCAGAGATCTCAGACAAGGTCCAGCTGGGATGGTGGAGGAGGAGGAGGAAGAAGAGGAG
GAAGATGCTGAACAGATGGCAGCACACATGGGTGGAGATGCTGGAGAGGAGATGAACGAGGGAGATCAAGAGGAGGAG
GATGAAGATATGGAAGAAGAGCCACATGTTAATGGCTCCTGAGTACCCTGAAAGCATTTTCGTGTTGTTTCCATGTTG
TTTAATTCTTGTGTTGCATATAAAATTACCAGGGCTGTTTTTTATTTGTAAATTGACAC

>Sequ06310EST2
GGGAAGGCTTGGATGTGATCATCATCATCATCCGTTACTCTCGAGCGACAGGCCCAATCCGTCCAGGAGGCTGCGGTG
TGTTCCGGGACCAGGAAAGGGACCAAGCCAGGTTGGTGCTGTGGTTCTGTCCGGAGCGGAGTACACATTGATCCCTGC
TCCACCGACAACTTGACTCAGATCTGGGTCACACTTCACTGTCACCATGGGCTCCTCCACCACATCTGCCGCTCTGTT
GTGTCTGCTTGCGGGGGCCGTGGCAGCCATCCAGACCCCCCAGACAGTGTTTTTGGATAAGCAGCAAGCGAGCTTGGT
GATCTCCCGTCAGAGGAGGGCCGTCGGTGGCGAGTAACCCCTGCCTCCAGCCTGGAGCAGGCTTGCATGGAGAAGGTG
TGCAGCTACGAGGAGGCCAGAAGGTTCTTCCCTAGGACTCGTATCGTACGGATATCTTCTGGTCCGTCTACATTGACG
GAGACCAGTGTGCAGGGAGCCCTTGCAAGAATGGAGCCCTGTGTTCAGACAGCGTGGGAGGTTATGACTGTGTCTGCA
AGTCAGGTTTCACAGGGGTCCACTGTGAAAAAGACGAGACGCTGTGCACCCTGGAAAAGAACAAGGGCTGCTCCCAGT
TCTGTAAACCAGGCTACACTTCCTACCAGTGTTCCTGCGCCCGTGGTTGGAAGCTCAGCCAGACGAACAGGGATAGGT
GTGAGCCTGCAGTCAGATTCCCCTGTGGTAAGGTGAGCAGTCTGAGTCGGTGGGGTGACAGACAGTCAAGCAACACCC
GCAGCAACTTTGAGGGACTAAGCTGTACTTCTACAGAGTGTCCCTGGCAGGCTCTACTGAAGGGCTCTGAGTCGACTG
GATTCTGCAGCGGAGTCATTCTGAAGGAGAACCTGGTCTTGACTTCAGCTCATTGCGCCAACAAATACAGGTCATTCC
AGGTGGCGGTCGGAAAGCGCAGCACCGTCTATGAAGATGGAGAGCAGACACTGTACATGAAAATAATCCACATCCACC
CGCGCTATGTGGAGGGTCGTCCTGAAAATGACTTGGCCGTGGTTGAGCTCCGCGACCGCATCACCTTTAAGAGAGAAG
TGGTTGCTGCCTGTCTGCCAGACAGAGACTTTGCAGAGAGCATCCTGATGACAGGAGAGTTACCCGCAGTGGTCACTG
GCTGGAAAGACCCAAACAGGTATCAGCTTTCGAAGGCCCGCTCACCCTTAACCACCTGGTGTACAACCAGCTGCCTCA
GTGTCTGAAGTCTCACCCCAACCTGATTACCAATAAAATGGGCTGCACTGCTCCTCGGACCAATGCCGACTGCACCAT
GAGCTCCGGCAGCCCCTTGCTCACCCTGTACAGGGAGGTGTTCTACCTCACTGGGGTGGTGAGCCAGCCACAAGGGTC
TGACTGTAGCAAAGGCTACATCTTCCAAAAAGTGTCACGCCACCTCGGCTGGCTGCAGTCGCTCATGGGCTCCCGTTA

GAGGGAGTAGTCAGGGACAACATGAAAGCTACAAATTTAAAGTAAGGCAGGTATATAATATATATGTAAGAAATCACA
TCAGTGTTTTCATGTGTGCACTTCTAGCTCAGTCTTTAGTTTTGCCATTCTGTCTTTTAATAATACCCATCTACGAAT
GGAAAAACTATCCTAACAACATATAGTCTAAGTCCAGGTGATTGCTTTCCATTTCTCTCTATGGACTTCTTCATTTAA
CCACAAGGTGGCTCTCTGTTAACATAAACAGAGAAGCACTTACTGTATGTATCCACCAACCATATGGCCAAGAGATGA
AGTGAATTATTCAGCATGTGTTTTGTGATGTGGACAGACCTTTTAATAACCTTTGAGTGATTTTATTTTAGTGACTAC
GGCACATTGTAACATTCCAGGTAATCACTGTTCTATGAATGTAATAAACCCCAACATCAAAACGAAAAAAAAAAAAAA
CCCG

>Sequ06315EST2
GAAAGAGGCTGCTGATCCCCTGGCTTCAAAACTCAACAAGGTAACCCAGCTGACAGGCTTCTCAGACCCGGTGTATGC
TGAAGCCTATGTTCATGTCAACCAGTATGACATTGTGTTGGACGTGCTGGTGGTCAACCAAACCAGTGATACTCTCCA
GAACTGTACCCTTGAGCTGGCCACTTTAGGTGATCTCAAGTTGGTTGAGAAACCTTCACCTCTAACTCTGGCTCCTCA
TGATTTTGCTAACATCAAAGCCAATGTCAAGGTGGCCTCTACTGAGAATGGCATTATATTTGGCAACATAGTCTATGA
TGTGTCGGGAGCTGCTAGCGACAGAAACTGCGTCGTCCTCAGTGACATCCACATTGACATCATGGACTACATCCAGCC
AGCTTCCTGCACTGATGCAGAATTCAGACAGATGTGGGCAGAGTTTGAGTGGGAAAACAAGGTGACGGTGAACACCAA
TATCACCGATCTGAACGAATACCTCCAGCACATCCTCAAGTCCACCAACATGAAGTGTCTGACTCCAGAGAAGGCACT
CTCTGGCTTCTGTGGCTTCATGGCTGCCAACCTTTATGCTCGTTCTATCTTTGGAGAAGATGCCCTGGCTAATGTCAG
CATCGAGAAGCCCATCCACCTGGGGCCTGAGGCACCTGTCAACGGACACATACGCATTAGAGCCAAAAGTCAGGGGAT
GGCCTTGAGCCTCGGTGACAAGATCAACCTCTACCCAAAAAAGACAAATGTCTGAGACAACTCAGATCCAGCTGATCC
CTCTGACTTTCTAAAAGTCACCTGCTGTTCTCTTGTATCACCCATTTTACAGCCTTTTCATCACAGCAAATTAAGAAC
TTTATGTGGTCAGTTGCTTCCGTGCTGTTTCTTTCTTTTCTTTTCTTTTTTTTTTTTACATCACCTCTGTTTAAAAAC
TACATCTCATAAATGTCCCTGTCCTTCTTCTTTTCAATAAACATATGTATTAAAAGAAGGC

>Sequ06375SNP2
AGTGTCAGAGTGGAGCGTGGAAGCATCCCAGCTCCAGTACGAGATCAACCACTCAACCATCCAGAGCAACACCCGACA
TACAGACAGGCAGGACACACAGGAACACAATGACAACGATGATGATGATGCAGTCCAGCCAAAATGACATGACTAAAC
AGAGAATGTTTCAAGCCAAGGCTCTGGCTAAGGAAGCCAGAGGAGGGGGTCTGAACCTGGGGAAGAAGATCAGCGTTC
CAAAGGATGTGATGATGGAGGAGCTGAATCTTCCCTCTAATCGAGGCTCTCGCATGTTTCAGGAGAGACAGAAGAGAG
TGGAGAGGTACACGCTGGAGAACGCTGCCAACGGTGCTTACAACACCAATAATGTCCATCTCGAGGCCATTCCTCCCC
GCAAATCATCCCAGAGCCACAGGGAGGAAAGGAAAACCAGGCTTTCTCCATCCCTGGATATTCTGGCCCTTTGAAGGG
AGTTCCTCACGAGAAGTTCAACACAACAGTAATCCCCAAATCCTACTGCTCCCCATGGAGCGAAGCTCTGGGAAACAA
CGAAGAGCTCCTCAACATCCTCAATACCCAACTGCCCCGAGCTCCCACAAACATTACAGCCTGCCAGCTACAGGTGCT
TCAACAGATCTGCCATGCCATTTGGGGGCGCAATGGCCAGCAAGAGGGTGATACCAGTGATGAGCTTTGAGGCAGTGG
AATCCCAGAACTTGCCCAGCATCGCTCCTGACCGCATGTGCCAACGACCCAACTTCAACAGAGCACCCAGGGGATGGG
GAGCTGATTACAGCCCGGAATCAAATGAGCTATGAAAAAAGGGCTGACAACTTAAGACTAAAGCGCTGGCCTATGCTT
GCTCCTCTCCTCCTGCAAAGATGCCCCCCCTGACAGTCAATGGGCACTGCAGCAGGTGGGGGCACACTCCTGCATGAT
ATAGAAATTTGCTACTGCTGCGTGGTTTGACCAGCTGAGGCAAACTCTGGATGCTGGAATCAGTTAAATGCTGCAACA
GGATGATCAGTCCTAATTATTCCCCCTTCATCCAAACAAAGTGTCTCAATATGTGACTCCTCTTCTCACCACTTCAGT
TTCTTTTCATACTGTGGCTTTACAACTTTTTCTCACCTACCCAGACAACACACATACAACAGTAAATTGTTACGTAAA
TGAGTAATATGAACTCTGAGATGATCAATGACAAAAATGAGTCATTTTAATGACAAGTGAAAACAACATACAAAGAAA
GGCTTACATTAACGAAGTCTCTATGTCGTGGAATATTAAGATTGTGTCAAATAAAAAAAAAAATGTCAATGTAACTGAA
AATTAATACGTTTTAAAAATGCTCCTCAGAATTCAACTCCATGTGATACTTTGTACGATGTGACAATGACATGTTTTA
CCTAAAGTATACATTTATATATGTACAATGGCTCCTACCACAGGAGTCCTTTAAAGACAACCCTGATCACACTGGTCA
GTACTCTTATGTGTGGTGTCTTTTACTCTGTTAAGTTCCGCTGTTTAGATGCTGTTCTGGTACTTTTTGACTGCGGTT
AGTGCTAAAGTCATCTGGTAAGATGCAGGTTGTGTCTCTCCAGACACAGATGGTGCTCCTTCAGTTCATTCACTGCAC
TGCAGGTGGCATAATAAATCAGATGTTATACTGTAAGACATAATTGTCTTTTCTATTCAATGTATTTCTCCCATAAGT
AGTCTGCAGGTTTCAGGCCCACTGACAGCATCTGAAGTGAAAGCACCTGACCAGGTTTGACTCAAGGAAAAGAATGAC
ACCTAATCACAGTACAGTATTTCACTCTAGCAGCTAATCACCATGGCGTGAGCCATGTGCAGCATGGGCACCCATGCG
CTGAGGGTCCTGGGAGGGGTAGTGGATTTGACCAGGAGGTCTCATGCTCATTGGAGGGGGCATGGGAGGAGCCATGGG
ACCCATGGGGTGAAACATTGGAGGGCCGAAACCTGGAGGAGGTGGGTTAATGCCTGGAGGTGGGGGCAGAGCAATGTT
CATGACAGCAGGGAGAGGTGCTGGGGGTCCAGGTTGAAGTAGTTTGCAGGAGCCTCTTCATCCATAGCTGGTGGTGGA
GGAAGAGCTCCAGGCAGTCCAGGTACAGGATCAAGTCTGGTGCCAATCTCACTGACACCATCTTTTGATGCCCTCCTT
TCCTCTTGCTGCCTGAGACCTTCCCCACTTGACAGTGAGCCTGCGTCCATTAATGATCAGTTTGTTGAAGGACTTCTC
AGCAGCCATTTCTGCTGCCTGCCGTGTGGCAAACTGGATGAAGGCA

>Sequ06444EST2
GACGACGATTTGGTGGAGGAGGCCACCGTCGTGATGGAGGACGACTCCCCCGCCGAGCCGGCCGGCACACCGGGCACC
CAGAGGAACCTGTCGGCCTGGATCATTACCATCCCCTACATCGACATCTATGACGACGAGGTCAAGAGGGAGAGGATC
CCCGTCTTCTGCATCGATGTGGAACGCAACGATAGGAAGGAAGTAGTCGGTCATGAAACTGAGAAGTGGTCGGTCTAC
AGAAGATACATGGAGTTCTACGTACTAGAATCCAAACTCACTGAATTCCACGGCACATTCGCAGATGCACAGCTTCCA
TCCAAAAGAATCATCGGACCAAAGAATTATGAATTCCTCTCATCAAAAAGGGAAGAGTTTGAGGAATATTTGCAGAGG
CTCCTGCAGCATCCAGAGCTCAGTAACAGTCAGCTGCTGGCAGACTTCCTGTCTCCTCATAGCATGGAGTCTCAGTTC
CTGGACAGGATGCTGCCTGATGTCAACCTTGGGAAAATTTTCAAGTCTGTACCAGGGAAGCTGATAAAGGAGAGAGGA
CAAAACCTGGAGCCTTTCATCCAGTCGTTCTTCAACTCCTGCGAGTCGCCCAAACCCAAACCCAGCCGGCCAGAGCTG
ACCATCCTCAGCCCCACGGCAGAGAACAACAAGAAGCTCTTCAGCAGCCTGTTCAAGAACAACGCAAACCTGTCAGAG
AGTTCGGAGAGGAGAGCCAACCCCAACTACTTCATGGAGCTGCTCAATGTTCATGGCATGTATGACTACATGATGTAC
GTCGGTCGAGTGGTGTTCCACATGCCCGACTGGCTGCATCACATTCTGGCCGCCGGGAGAATCCTGTTCAAGAACACG
TTCGAGGCCTACATGGAACAGTACATGCAGTCCAAACTGCAGCAGATCCTCCAGGAGCACCGCATGGTGTCCCTCATC
ACACAGCTCAGAGACGCTGTGTTCTGTGAGGACGGCGAGGAGCGAACCACCGAGGACAAACAAGTCAGAGCCAAGCAG
ACGTTCGAAGAGATGATGAAGTATTTACCAGACTTTGTGGGAAAGTGCATCGGCGAGGAGGCGAAGTACGAAGGTATC

CGGCTGCTCTTTGACGGTTTCCAGCAGCCGCTCCTCAACAAGCAGATGACGTACGTCCTGCTGGACATCGCGGTGCAG
GAGCTGTTTCCTGAACTCAGCAAGGGAGTGAAGGAGGCGACCTTCATGTGAAGTCAACACTGAATCCACATCCTGGAC
GATCGACGTGCAATACACAACGATTTCCAACACCTCTGGAAACACCTCTACATTCCAATGTTTATTAATGCACAAATA
AAGATATATATTTATTGATGTCACCGC

>Sequ06448EST2
AACCTGACGACTGCACGGAGCACAAGGTCTTCATCAGCCTCAACTTCATTTTCTGCATCATTGTGTCCATCGTGGCCA
TTCTTCCCAAAGTTCAGGAGGCTCAGCCCAGCTCGGGCCTGCTCCAGGCCTCCCTCATCTCCCTCTACACCATGTACA
TCACCTGGTCAGCCATGACCAACAACCCCAACCGGCAGTGTAACCCGAGTCTGTTGAGTTTGGTCCAGCGCACCAGTC
CAACTCCACCACCGGGGCCTGCTCCTGCCCCTGGAAACGTCCAGTGGTGGGACGCGCAAGGCATCGTGGGATTGATGA
TTTCCTGTTCTGCACTCTTTATGCCAGTATCCGCTCCTCCAACAACACCCAGGTGAACAAGCTAATGCAGACTGAGGA
GGGCCAGGGTCTGACCGCTGACGAGGAGGCCGCCACAGGGGAGGACGGAGTCCGACGGGCTGTGGACAACGAAGAGGA
GGCCGTCACCTACAGCTACTCCTTCTTCCACTTCAGCCTCTTACTGGCCTCCCTCTACATCATGATGACCCTCACCAA
CTGGTACAAGCCCGAGAGCGACTACCAGGCCATGCAGACCACCATGCCGGCCGTCTGGGTGAAGATCGGCTCCAGCTG
GCTCGGCTTGGCCCTCTACCTGTGGACCCTGGTGGCTCCGCTGGTGCTCCCCGACAGAGACTTCAACTGAAAAACGGT
AGAGTCTCTCCACATTGGTCGATAGCCTCTTGCTGTTTGCACCTTAAGGGAAGATAACTTTAGCTTCAAATCATCCAG
CTTGAAATGTTTGATTTTACTCACAACTCCACATTTAAAGTTGCAAACTGAAATGTTAATGTGTCTTTTGTTTTGACC
CTAAGCCATTGCACAATTCACAGTTAACGTTAATGTGGGTTCTTGTCTCGATGAAATTTTCATTTTTGCAGAGGCTTA
GTTGAGCTTTAATCACACACTGTTTTTCGCCTCAGGTTATTTTTCTATTTTTCTTCCACAGTTACAAATATGAGAATT
GAGAGCACTACATGCCTTTATACAACAAGACCAAATGAATCTTAAATTATCTCTGTGGGAAATTCATGTTGTGACTCT
GTTCACTACATTTACATTGTATTTG

>Sequ06460SNP2
ACATCCAGACAGGTTGGAGAGCGGACACTGACGGGGACCGTGATCGACAGCGGAGACGGAGTCACACACGTTATCCCA
GTGGCCGAAGGCTACGTCATCGGTAGCTGCATTAAGCACATCCCCATCGCTGGTCGAGACATCACGTACTTCATCCAA
CAGCTGCTGAGGGAGCGAGAGGTGGGGATACCTCCGGAACAGTCTCTAGAGACGGCTAAAGCTGTCAAGGAGCGGTTC
AGCTACGTCTGCCCTGACCTTGTTAAGGAGTTCAGCAAGTACGACACAGACGGCTCCAAGTGGATCAAACAGTACACA
GGCGTGAACGCCATCAGCAAGAAGGAGTTCACCATCGATGTTGGCTACGAACGCTTCCTCGGGCCAGAGATCCTTTTC
CATCCTGAGTTTGCCAACCCAGATTTCACCCAGCCGATCTCGGAGGTGGTGGATGAGGTCATCCAGAACTGTCCCATC
GACGTCCGGCGTCCGCTGTACAAGAACATTGTGCTCTCCGGAGGCTCCACCATGTTCAGGGACTTTGGCAGACGCCTC
CAGAGGGACCTCAAGAGGACTGTGGACGCACGACTGAAAATAAGCGAAGAGCTGAGCGGCGGCAAGTTGAAGCCCAAA
CCCATTGACGTGCAAGTCATCACTCACCACATGCAGAGGTACGCCGTCTGGTTCGGAGGATCAATGCTGGCGTCTACT
CCTGAGTTTTACCAAGTGTGCCACACTAAGAGAGACTACGAGGAGATCGGCCCGAGCATCTGCCGCCACAACCCCGTA
TTCGGAGTCATGTCTTAGGCGCCGCGGGAGCACGTTCGGCGGAGGCCTCGAGGAAAAACGGAAAAGGAAGCGGCGCAA
GACCTCACATGCTGTGGAAGTCCCATGAGCCTTTGCACGGATGCTCCGACAGACCTGTTCATTCGGATAGAGCGACAG
TGGACCAAACAAACTGCCAAAAGAGTGGCAATATCCTCGCAAATTAGAGGAAAGAGGTGACTCAGCCCAAAGGATCCT
CAAATAAAAATGTTTACTGCAAGTAGGTTAATAATAGGTGATAAATCTTTAATTTCTGTGTGCCTCGTATATGTACTT
ATTCTATCCTCACAGGGTTGTAATATGTAATTAATCAACATGAGTCCTGTATAAAATGCACTTCTCTGTGTATAAGGG
TTGGGTTCTTGCTATATGTTAAATGCAAACTTAAGGAGTTGTCTCAAGAAGCAAGACGTGCTTAGCTAGATCACCTCC
ACCAACGTCATCGAGTCAAACACCTTTCTGATCGCTGAGTCATCAAACTGGTGCAAAAATGCAACACATTGTTACAAT
AAGAAAAAAACAACAAAAAAAAACAATCAGCAGACAAACTGACCATTCCTGATTTTCTTTCTTCTTCGTTGGTGTCTT
TTTTTTTTCTTTTTTTTTTAAATTGTCCCTCAGTACCTGGAAGAAATGTTTATTTGAAATTAGATGAAAATAAGTCAG
GCATTAAAAAACAAAAAGAAAAAACCTCTTTTAGGAATCTGTATCTGTTCAAATAATTTGTTCCAGGTTTGACTGAGA
CGCGTCCCGTCGTCTTTTTCCTCCTGCAGCACTCTGACTGATCGTCACCGCTTTCCTTTGACCTTTTTTATTTGTATT
AATCATCTCTTGAACAACCTGTCATGGAAGAAAAAAAAAAAGAAAAAAAAAAGTCG

>Sequ06472EST2
TCTCCAGGGCCCAAGCGACGTCCCTCTAGGTCCCCCAAGCGCAGAAGTTCTCCTGCTCAAAGGAGACGCACACCTCCA
TCCTCCTCATCACCACCACCAAGACATAGGAGGAGCCCCATGTTGCCTTCTGGCTGGCAAAGCAGGGACACACGATCC
CCTGCTGCAGGAGCCGGCCGCCTGTCCCCATCTCCTGTAAACCGCAGTCGCAATCTTAGGGGTTCCACCAGTCCCCAG
GGACGTTTTGAAAGCTCCTGCACATCTCCATCAAACCAGCGAAGACAACAGTCCCCTTCACACAGTGGCAAACCCTTC
CGCAGAGTGTCCCGCACCCCAGAGCCGCGCAGCAACCAGAGACCCTCTCCAAGCCCCCAGCCTATGAGGAGAGCATCT
TCCAGATCAAGATCTGTTTCTCCTCAACCAGCAGCTCAGAAACGTCCACCCCCTGCATCTGCCTCCCCCTCACCGTCT
CGCTCTGCTAGTGGGTCCCCACCACCAGCCAAAAAGGCCAGCAGCGGTTCCGGCAGTCAGTCCCCAAGCAAGAACTCA
GATGTTGACGGCAGTGGGAAGAAAAAGAAGAAGAAGAAGGAAAAGAAACACAAGAAAGAGAAGAAACATAAGAAGCAC
AAGAAGCACAAGAAGGAGAAGAGTGGTGCTGCTGGTAATGGAGATGGGCAAGAAAACCAGGTTGTGGAGGAGGATGGA
GATTCAAGAAAGGAATCAGATAGTGAAGTAGATGACAGCTTGGACGACCTAGAGAAGCACCTAAGAGAGAAGGCCCTG
CGCTCCATGAGGAAGGCCCAGATGTCTCCATCACAGATGTCCTGAAAACAAGGGGCTTTCTCACTTTTGAATTTTCCA
CTCACTCTTGATGTTCGTCATCAACCTGGTTCAGCTTTAGAATGTACAAATTGTTAATTCTTGAGTCTTTTGTTTTCT
TTTTACTGGTTTGGTACATTCTGAAGAGTGCGTTGCATTAAGAGCTTGATTTGTCAGTTAGTGTATTATCTAAACTGT
AAAGAAGCTTGTGTTGTCTATCATTTTAAATTGTTGAGCAGGGAAACGGTTTAAGCTCGGAGGTTAGAGTTGGGCAGG
TTTCAATTATTTTCCACAGCCTACACTAGATTCCATTGAGAAATTATTGCATGTGCAGTGATTGTCTCTGATGTGCAT
GAACCACTGACCTTATGGAGTGTAGAGAAAAACCAACATACAGTAACATACTGTTACTACTGCCTTTTTATTAGTGGA
ACCAGTGACACAGCTGTGTGCACTGTTAGAGGCCCTAACAGAATGTCTTGTCCCACATTGTAACTCAATAGAGGTGTT
TGGCATTGTTTAACCTGCTGTTTTTCTGACTTTAATGATCTTGTTTTATGACTAAAACAGTCTGATATGATACAGAGT
ATGCTTTAATTTTCCAAATGAATTGATATGCAATTTTATGTTGACACTCTTGCACCAAGCTAATCTCTGTGGGACTGC
TCTATAATTTGTATTTAGCAACCCTGGCTCTTGTCATCTGGTCATCCTTTCAGAATTAAAACCTTTTTATAGGATTTG
TGTATTTTCCTTTAATATAAGGGGCTGGAAGATTTGATCAGTTTTGTGTTGGTGGGGATCTATCAACTGACAATCGAT
TTGGATGGATGCAGATAAAAAAAATCTTTAAAAGCAGGTAAAATGCAGAATTGGTCATTACTGGGTTTTGGTTCTGTT
ACAGTTCACCTACATCTTCTGAAGCTGGGTGTATGATATCTGCCTGCACTGCATAATTATTCAAATGAGACAGGGCCC

AATCTTATGTATCTGCTAAGTCAAAACATCTAATGACAACATGCTGCAGGATATTCAGCAGCTGATCAGAAAGTATGT
GTTCTGTG

>Sequ06483EST2
ACGTCAGAGGGAACAGCAAAAGAACAAAATGGAGGAATATGCTAGAGAACCATGCCCCTGGAGGATTGTGGACGATTG
TGGGGGAGCTTTCACCATGGGAGCAATTGGAGGAGGAATATTCCAGGCAGTAAAAGGTTTCAGAAATGCACCCTCAGG
GATGAGCCACAGAATGAGAGGTAGCTTGACTGCCATCAAGACCAGAGCCCCACAGCTTGGAGGTAGCTTTGCAGTATG
GGGAGGCCTCTTCTCCATGATCGACTGTGGTTTAGTAAAAGTGCGAGGGAAGGAAGATCCCTGGAACTCAATAACAAG
TGGGGCCATGACAGGAGCTATCCTCGCTGCAAGAAATGGACCAGTAGCCATGGTAGGATCTGCAGCCATGGGAGGTAT
TTTGCTGGCATTGATAGAGGGCGCTGGAATCTTGCTCACTAGGTTTGCCTCTTCACAGTTCCCAACCGGGCCTCAGTT
TGCAGAGGAACCTGCCCCTGCTCCCATGCCCACCCCTTCCTTTGGAGACTACAGACAATATCAGTGAGAGGACTCCCC
ATGTCTTTATCCACTAGGCCTTTTTGAATTCCTTGCTGAAGTTACAAAGAAGAAATGGGGATGTAGAATACCACACAT
CAAAATAGAAGCTGCACCCGATTTTATGGACTGTGTCAGAAGTGAAGGACAAAATACAACTTAATTTTCCTACTCAAG
AGCCATGTACAGATCTGCCTTTTGCCATTCTCAGCCTTGTCGTGTGCATCTGTTTCACCCAACATTTGTTAAATACAG
AGATGTGGTGGGATTTATTGTTTGATGTTCATAGGCTTGTGGGGATGAGGTGTGTTTTTCTATTTTGTTCATATAAAG
TATATATACTGCATGTAGCCCAACAAGGGAATTCAGGACAGACCAGCTCTGGATAATGTTGAATCTTGCAAAAACACA
TGTCACACATGGAACGTAATAAATTATACTTATATATAAGTATAATACTTATGTGTATATACAGTAAAAACTTTCTGC
TTGTATTCAGTCATGTTAGTCTTTATTTGCCTTAAATATGTCACCTGCTTGTGCTGGCCACTGTCACTTGGCTGGCTC
TTTTATTTGGTTTTATTCAGAGTTATGAAGGCAAACATCTATCAAATACAATTCTATTCCTGGAATATTAGTTTGTTT
GAAAGTGATTTATTTATTTTAGATTGTAAAGAGTTGTGCAAGATTGTTGTATTCTATCAGACATTGCTTTTGCATTCA
ACACAGAGGTGTGGAAAAGGCTGCTAATGACAAAGGATGGCAAAGACAGTACAAATAAATGGAGAAACTCGAGCAAGG
GATTGACGCATATACTGTATTAAATCTGTATGACAGATTGTATTATGGTACAATAAAAAATGAGTTGTAAGATCTGTC
TTGTAGCCTTATGAATACAGTTATCCTATTAGTTCTGGTCTTGATTGTGAAAATAT

>Sequ06484SNP2
GCTCTGTCCAAAGGTAACAAAATCCACCTACAAGCACTTCTGAAACTCTCTAATTCTCATGTTTGTCTAAGCTGTACA
AAAACCAAACTGTAAAAAAGACAAGTGGTCAGTAGTTCTAGAGGATGCTTATGTGTAGGGTTATTGTTTTACACATTA
GTGTTTGTATGGATTAAACAAAAAACATAACCTCTTGATTTGTTGGCTTTATAGGTGCTATTAGGTGGATTTTGTTGC
CTCTGGTCAGCAGCTGGCCGGCTGTTTCCCCCTCTTTCCAGTCTTTATGCTAAGCTAAGATAACAGGCTCCTGGCTGT
AGTCTTTGCAAATATGCATATTTCTAGTGTATTCCTTTGTCTTTAAATAATAGTTTTTATTTTTATTTTTTTTATAAG
AAAAGGGATAACATACATGCTTTGTTAGACTTTCCGGTGGATGAAACTACCTGAGGTCAGTACATATCAAGATTACAT
TACCTTCTAAGAGGAAATATGGTCCAATTCCCTTTACCTTTTATCTCAGTTCCTGAGTTTGGTGTGTGTAACGACTTG
TAGTTGCATGTGTGCTGTAATCATCAACGTATAACTTTGCCTTTAGCTTTTCTCTACACTGTCACTCACACTGGAAAA
GTTAAGAGGCAGAGTGCTCCTCACCTGCAATGCTGCATTTGAACACACAGAGGTGCTCAAGTATCACAGTCCCCTGCT
CCACATACAGATGTGGTGATTGACCATGTTTTTGTCTGTACAGTTGGTAATGTATGAATGAGTTCTTACTGCTAACAC
AAAAAGAAAAGAAAAGAAATCAAAGAGGAGGGGTCGGTGTTGTATTACACAACTTAACAATAGAAAATGAACTCACAA
AGGTTTTATTAACTAAGTCAAAGTTGGTGTCATATTTGCCATTGCAGTGTAACCTTTTTACTTCACTTTTCACTGTAT
TGTGTTTTTAAACAGTTCTTTCCATTCCAGCTATCCTTGTGTTTAATTCTGGGTTTCCTGTCATGTGTAAACATTGTC
TGACACTTTTGTTCTCTCAGGGTTCACCCACATCTCAAAGAATCAGATGGTCTTTCCTAGTAGACCTAAATGGTACCT
TTAAAACTATTATTAGATCAACTAAAACCTTGGGCATTTAAGTTAAGATTTATGCTTTTGTGACTCTGTAATGTAGTG
ATATTGGGCCATATTACAACTGAAAAAGGTCCTTGATAGTTATATGCAGTTAATTGTTGGAATGGTAATTGACATACT
GTTTAAATATATTATCTTTTAATGCTGAGTTTGGATATGTCAGATGACTATGAAATTTTTTTGTGTAGTTGTTAATGG
CAAGCTAATACTGGTATCATGCTGCTGGTAAACTAAGCAGTTTTTTAAGTGTAATGGTAAAGTTCAGACACTATAATC
AAGAGTGTGTTATTTGATGATTGAGAATTCTGTTGAAATTATTTTTCATCTCTTATTCTCTGCACAGGAATAAATAAA
TGTTAAATAT

>Sequ06489EST2
CGCCGAGTGATTCATCCTGAACCACAGAGATGGCTTAGCTAGTTTGCTAGTTTACTCTATGATAAACATGACTCCGAC
TCGGATAAATGTGAACCTATCTATTTTACGCTCATAATACTCTTGTTTATATCATACAACCATCCAACCATGAAGCTG
ATCCATAAAGACATTGAAAAAGATAATGCCGGTCAGGTGACTCTGATGCCAGAGGAGGCAGAGGATATGTGGCACACC
TACAACCTGCTGCAAGTGAGGGACAGCCTGAGAGCCTCCACTATCAGGAAGGTGCAGACAGAGTCCACCACTGGAAGC
GTGGGCAGCTCCAGAGTTCGCACTACTCTTACTATATGTGTGGAGACGATCGACTTTGACTCCCAGGCCTGCCAGCTG
AGAGTAAAGGGCACTAACATAGAGGAGAACCAGTATGTCAAGATGGGGGCTTACCACACTATTGAGCTTGAGCTTAAC
AGGAAGTTCACTTTAGCTAAAAAAACTGGGACAGTGTTGTGCTGGATAGAATCGAGCAGGCATGTGATGCAACCCAGA
AGGCAGATGTGGCAGCTGTGGTAATGCAGGAGGGTCTGGCCAACCTGGTGCTGGTGACACCCGCCATGACTCTGCTCC
GTGCAAAAGTGGAGGTCACCATTCCTCGCAAGAGAAGAGGAAGCTGCACTCAGCACGAGAAGGCGCTGGAGAGGTTCT
ATGAGGCTGTGATGCAGGCGATTCTTCGGCACATCAATTTTGATGTGGTGAAGTGCTTCCTGATTGCCAGTCCAGGGT
TTGTGAGGGACCAGTTCATCACCTACCTCTTTAAAGAGGCAGTGCGGCAGGACAACAAGATCCTGCTGGAGAATCGCC
CCAAATTCATGCTGGTCCACTCGTCTTCGGGTCATAAGTACTCACTCAAAGAAATCCTCTCTGATCCCACAGTGACAA
GCAGGCTTTCTGACACAAAGGCAGCAGGAGAGGTGAAAGCCCTAGAGGATTTCTATAAGATGCTCCAGCATGAGCCTG
ACAGAGCTTTCTATGGAGTGGCTCATGTGGAGAAAGCTGCTGATGCCCTTGCCATCGACACTTTGCTGATAAGTGATA
AGCTGTTCAGACATCAGGACGTCCCGACGAGGAGTCGTTACGTTCGGTTGGTGGACAGCGTGAGAGACAATGGCGGCA
ATGTCAGAATATTCTCAAGCCTTCATGTGTCTGGTGAACAACTGACTCAGCTGAGCGGAGTGGCTGCCATCCTGCGGT
TTCCCATCGCCGACCTATCAGAGGGCGAGGATGACAGTAGCTCAGATGAAGACTGATCCCCAGAATTGGACCACAGAG
GAAAAATCATTTGGAGAAAAGAAGGTGGTCGTCCATTGTTGCAGATAACACAAGACTGCGTTGACTCAGTGTGTTATA
AAACAATAAAGCTGCTGTGTTATGGTCTATGTATTCCACCTCTGCAATATCCACACATCTCTCAGACCTAATTAACAC
ACCTGCTTTTCTAAAGTGGCCATAATATCACATAATTAGATGAAGTTTTTGTTTTGAGGTGAGTGTCTTTGAGATGGG
GCATGTTAAGCAAATGCCAGAGAACTCTTCATAAGAATGCCAAACAAAAGGAATGTAATTTGTTGCCAAGTTTCAAAT
GAAAACTGTTAGTTTTAAGAAACATTAAGCAATAGGAAAAAATGATTTTTCTTTCCAAGTGTATATACAGTTTATCTT
TCTTG

>Sequ06491EST2
GATCCATTATGAAAACAACACACCCTTCCTCACCATCAGCAGCATCACTCGCACTATTGAGGTGTCTCACTGGGGCAA
CATTGCTGTGGAAGAGACCATCGACCTGAGGCACACAGGAGCCGTCCTTAAGGGCCCCTTTTCACGTTACGATTACCA
GCGTCAGTCAGACAGCGGCATCTCATCCGTCAAATCCTTCAAGACTATCCTTCCTGCCTCAGCCCAGGATGTCTACTA
CAGAGATGAGATCGGGAACATCTCCACCTCCCACCTTCAGATCCTGGATGACTCAGTGGAGGTGGAAGTCAGGCCCCG
CTTCCCCTTGTTTGGAGGGTGGAAGACCCACTACATCATTGGCTACAATCTGCCCAGCTACGAGTACCTCTACACCCT
GGGTGACCAATATGCACTGAAGATGAGACTAGTTGACCATGTGTATGATGACCAGGTCATTGACTCCTTGACTGTGAA
AATTATCCTGCCAGAGGGGGCCAGACACATCCATGTGGACACACCGTACAAAATTGATCGTATGCCCAACCAGCTGCA
CTACACATATCTGGATACCTTTGGCCGACCTGTGCTGGTCGCCACCAAGAACAACCTGGTTGAGCAGCACATTCAGGA
TGTTGTGGTTCATTATAACTTCAATAAGATCCTGATGCTGCAGGAGCCTCTGTTGGTTGTAGGGGCCTTCTACATTCT
TTTCTTCACTGTCATCATCTATGTTCGTCTTGACTTTGCCATCACAAAGGACCCTGCTGCTGAGGTTCGTATGAAAGT
AGCCTCCATCACAGAACAGGTCCTGACTCTGGTTAACAAACGTCTGGGTCTGTACAGACACATGGACGAGGTGGTCAA
CCGCTACAAGCAGTCCCGGGACACTGGTGCGCTCAACAGTGGCCGGAAGACGCTTGAGGCTGACCACCGCACTCTCAC
CAATGAAATCAGCTCCCTTCAGGCCCGCCTCAAAGCTGAAGGCTCTGACTTGGCTGATAAGGTCGGGGAGGTGCAGAA
GCTGGATGGCCAGGTGAAGGAGCTGGTGTGTCGTTCTTGCCTGGAGGCGGAGCGTCTGGTGGCGGGTAAGGTCAAGAA
GGAGGCTTACATCGAGAGTGAGAAGACTCTGACCAGCAGGAGACAGGAGCTTGTCAGCCGTATCGACAGTCTGCTGGA
TGCCCTCTAAACTACTCCTGCCACACTAGCACACAAAAACACACACATTCAGCACCCCTGCTACAACATACAACCGAC
TGTCTGTATGTATGAGAATTAAAAGAAATGTGACACAATTCTTAAACAATTTTTTTTTTTACCATCATTGTTCCCAGA
CATGGACTATTAACATCTCATCTTACAGTAGTGAAGGGTTTATCCAAACACTTGTGCCTTTGTGCCTTGTGATAAACT
TGAGGTCACTGCATGTTCAGCTCTACAGCAGCTACATCACAACGTTTGGCTTTTTTGTTTCTGTTTTTGATTTTGATG
AGCTACACTTTGTCACACTTTGTTCAGACTTGACTCTTCATTTTCAAATCTGGACTCAATTTTTTTTTCATTCCTAAG
TATGTGCGGTTAAGGGATTTTTTTTATTTCTATGAATAGCTGAGGGGCCCAAGTGTTTGCAGTCTCATGTCTTTACTC
AGATATGCCATATTCTAAGATTGAATCATTTTTAGCTCTTAAGAATGAAGGTGATAGTCTTTTCCAATCTTAGCTCTC
TTGCACTTACTTTTTTTCTACTCGCCCCTGACTGATGTTTATATTGAAAAAAGTTGAAGCTCTGATTCTGTGTCATCC
CAAATGCTTGTTTTTGATGTCCCTCAGTCATATTTTCTATATTTTGTATCACTTTGTTTTGTAAGTGGTATTTTTTTT
TACCATGAGATTAGTGAATACATGAGATTAGGTTATATGTGAGAAAAGACATGAGAAATGGGACACAAATGTTTGTGA
AATGGGCAATGATATGGAAAGCTGTCAAATTTGAAATAAAATGTGGAGAAACTACAAAAAAAAAAAA

>Sequ06494EST2
GCGGAAACATGGAGGCTGTACTGAGTGATGTGGTAGCTCCTGAAGACCTTTTAAAATTTGAGAAGAAATACAACAGTG
AGCTGGTGAAGGGAGCAGTCTCCAAAGAAACAAAGTTTGAATATGCTTGGTGTCTGATCAGGAGTAAATACTCGGAGG
ATATCAAGAAGGGAATTGCACTCTTGGAGGAGCTTGTTCAAAAGTCATCAAAGGACGACTCCCGGGACTTCTTGTTTT
ACCTTGCAGTGGCCAACTACAGACTCAAAGAATATGAAAAAGCCCTGAAGTACATCCGGACCCTTCTTAAGAATGAGC
CAGGGAACAAGCAGGCTCTGGAGCTGGAGAAACTGATCGACAAGGCTTTAAAGAAAGACGGCTTGGTTGGCATGGCGA
TCGTTGGGGGAATCGGTCTCGGCGTGGCCGGCTTAGCGGGACTCATCGGCCTGGCTGTGTCAAAGGGAGCTGCCAAAT
CCTAACCTGGACATGAGAAGACGTGTCTTTGTTTTGCTGGACTGATACACTGACCTTTCGCCTGCAGAGAAAATTAAA
GCTTGTGTTAAAAGCTTGTTAAAGTTTGTTTGAAGGATGTAACATTTTGGTTTGAAGTTGAATATTTACTGAAGCTTA
ATAAAGGGTTTAGATAAAAGCCAGTGATTAACCTGTTTTCATATTATAACAGGGGTGAAAGTCACTATACATTTGACA
TTACTCTGTCCCACACTATAGTATTGCACTTTCATATTACTTATGTAAATAAATTAGAATAGATAAGGTGCAGCTCAA
CTCTATGTAATTTATTAGTTAGTGCGCACCTCAGCCTAAACTTCCTAAGTTGAAATTTACATTTTGTAAAAAAAAAAA
AACTATCTTTGTCAAAGTGCAGTTGTGGTTCTGTGACTGTGTCATTTTCATTAGTTTGGACATAAAGAATTGTAAAGG
AAAGTGTTACTGGTGCAGTGGGAAGTGTAGCCTGTAATGCCAAAAGGAACAGTGAGGTAACAGTGACTCTGCTACACT
GCTGTTAGGAAACTGTAAGTCCAGCTGCTGCTGCTGCTGTGAGGCCAGAGAACAGATGCATTGCGTTTATTTTGGACA
AGAAGTTTCTTTTCATTTGTTCTGAATACAACTTAAAGATAAGGGTGGCAGTGCATTTAAATTAATACATTTTGTCAT
GTAATTTGCATTTGCAAACATATATCTGTGCTGCATTTAATGACTTGTGTTTTCCATCTTAGTTAAATAAACAAT

>Sequ06517EST2, Sequ06517SNP2
GAGGGAAAGTCGTGACCCTCAGGCAGCGAAGAAGTGCCTGGCAGCCATTGAGGAGTGTGCCCAAACCAGAGAGGGCAA
CCTTTTAGCCCTGGCCGTCGAGGCAGCTCGAGCCAGATGCTCTGTTGGTGAAATCACTGATGCCATGAAGAAAGTGTT
TGGCGAACACAAGGCCAGCACCAGGATGGTGAGCGGGGCTTACCGCAGCGAGTTTGGGGAGCATGAAGAGATTGCCCT
GACCCACAACAGAGTTGCAGATTTTAAGAAGCACGAGGGCAGGAACCCTCGACTGCTTGTGGCAAAGATGGGGCAGGA
TGGCCACGACAGAGGAGCCAAAGTCATTGCCACAGGGTTTGCCGACCTGGGCTTTGATGTGGACATCGGACCACTTTT
TCAGACTCCCCTTGAGGTTGCCCAGCAGGCGGTCGATGCTGATGTTCACTGTGTCGGGGTCAGCACTCTTGCCGCAGG
ACACAAGACCCTGGTCCCAGAGCTCATCAAGGAACTACGGAAGCTACAACAGGCCAGATATTCTTGTCATCTGTGGAG
GTGTCATCCCACCACAGGACTATGAGTTCCTATACCAGAGTGGTGTGTGTGCAATCTTCGGTCCAGGAACCAGGATCC
CACAAGCTGCAGTGGAGGTTATTGACAACATTGAGAAGAGTCTGGAAAACATCCGGCAGGCCATGTGAACTCTTAAGA
AAAAACTACAACAAGAGCTGTCTATTCATGCTGTGCCAAGAACAGAGTCACAAGTGCGAGCCAACACTTTTGTACAGA
ATCTGTTTGACTGGTAGCATGAAGTCGTAAAAAACTGTTCACAGCATGTACAGAAGTTAGAATCACTACCAACCTGTG
GGGATGTTATGCTTGTATTAACACTCCATTCCCTGTATATCAGATTTGATTTGAACTAGTAATAGGATCCCCAGAGTT
TGCAGTAACTTCACAAAGAAACAAAATTAACATCACCAATAAAGAGTGTAGTGTTTTTTTTTTTTTCATTGAGGGAAT
CACTCAAAATCTAAAGAAATACTGTCACACATAAACGTTTCAGATCGGTAAATGGGGATTGAACACATGCTTGTGTGA
GAATTGCATTGGTTGCAGGGAGAGTAATATCACTGTAAATATCTTTGAAATTCTAAAATATAATTGGGAACTGGGACC
CTTGGTGAACACTGTAGACTGTAATAGGATATAATTCAATATTCACGACGGAAATCAGACTATATTAAGACCTTTACC
CACACTTACACAATGGAACATACATTACGTTTAATCAAATTGGAACAGCTTAACAGACACAGTATCATGTGACAGAAA
TTTGGCTTGAGAAGAGCAGAGCTTTTTTTGCCATCAGTATAAAACAAAGGACTATAAACCTTGGAATGTAATGCTTTT
TTCGTAAGATGTCTGTGTATAATTTGCAGTTACCTAGCAGGGTGTGAATAGTGCTAGTGTAGTGCGGATGGATAGACA
GAAATTCACATCAAAACGAACCTCTGTGGAACCCTTACCTCTGATTTTTATGTTTATGGATATTATCATATTGACAGT
GGCCTTTTCAATAGTTGTTTCGATCTTTCCTCCCTGCATAATGTTTGATTTGACCAGCTTCAGTCGGTGATCACAATT
TATGAAGATTTCCGAAATGGTATTTTTCTATGTGTGATTCTGAATCAGAGTTGGTGCTCTTCAGTCTAAATAAAGTAT

GTGAACTTTGGAAATGTGACTTGCATCAGAAGATTGTGTAACTTTCTACGCCTCCGCCCGGCGACAGCCAGGGGCTTG
GAGGCAATATGTTTCAGGTCATCCATCCATCCGTCCCATTCTCGTGAACAGATATCTCAGTAATCTAATCTA

>Sequ06529EST2
CAGACTTGTGCTGGCATCTCAGAGGGCAGCCCCATTTTCCAGTCCAGTCCTAGATGTCGAAGCTGATCAACCCCCTTT
GCTGCAGTTCACCTGACCTCTCAGTTAGAGCCTCCCCTGCTCTCCAGCCTTCCTGTGGACTAAACTGACCTCTCAGGG
CTGTCATTCACAACTGCGGACATCCACAGCTGATCTACACAAGCAAAGGACCAGTAACACTAAGCTGTGGACCAAGGG
GAGCAAAGAGAGTGCACCATACTTCATTTGAGGATACCCAACCTCGTCTAGTCCAAGTTGAACCCATCAACATCATCC
CCCACAGACTGAGGATCCAGTGGTGGAGCAAGACATTTCCATAGACTGTCACATTTCTTTGTGGGACTATACGTGGGC
CCTTTACAACAGACAGAGACTGATATAAAACCAAGACAAATCCATCTGATTCAAACAACATCTTTCACCATGGCAACC
ACAGGTATGCAGTTGCTGGGCCTAATAATGTCCATTGTAGGCTGGGTGGGTGGGGCGGTAGTCTGTGCCATCCCCCTA
TGGAGGGTCACTGCCTTCATCGGTAACAACATAGTGACGGCTCAGATCATTTGGGAAGGCCTTTGGATGAATTGCATT
GTCCAGAACACAGGTCAGATCCAGTGTAAGGTGTATGACAGCTTACTGGCTCTGCCCAGTGACATGCAGGCTGCCCGG
GGCCTCACAGTGTTCTCCATCCTGCTCTGTGGCCTGGCTCTGGCTCTGGGGGTCCTAGGAGTCAAGTGCACTAAGTGC
ATCGGTGTAAACAGCCTCAAGGCCCGTATTGCTCGCATCTCTGGAGCTCTTTTTGCCATTGCAGGGTTCCTCTACCTT
GTGCCCGTCTGCTGGACTGCCCACTCCATCATCAGGGATTTCTATGACCCACATGTAGCGGCTCCACACAAGCGTGAG
CTTGGCCCTGCCCTATACATTGGCTGGGCGGCTTCAGCTTTGCTCCTTATTGGAGGATCTCTGCTCTATGCTGGGTCA
AGTCCACCTGGCATCCCAGGCTCTCCCACCTTCAGCAGTGGAGAAAGTAGTCCTCGCAGGGCACCTACTACACAAGTC
AAAGGTTATGTCTAAGTTCCCAAAATGTCTGAATGCCTTCAAGTCTCACAAATCCACCCCAAACAATGACAAACCTGT
TTTTTCTCCTCTTGATTTTGCTCCTTGTAATTATTTTATATTTAATGTTATTCCAGTTTGCTTTTTTCTTTGAAAAGC
CGCAGGAGGCTGTTTAGGAAAAAATTGAACTCATTTTATTATTTCATACCTGATATCTTCAAATAACATTTTTTCTGT
TTGTCAGTGTAAAATACAATAAAACCTTTGGTTAGTACTAAAAAAAAAAAAAACCGACCTACCCT

>Sequ06599SNP2
GGACCTGGCTGATAGCAGTGGTGTGCCGGAGTTGGTTACACGGAGCTTTGCTGCAGCATTGGCAGGAACTGCAGAGGC
CATCCTGACGCCGTTTGAACGCGTGCAGACTCTCCTACAAGACCATCGGCACCACGCCCGCTTCAACAACACGGCCCA
CACCTTCCGGACACTTCTGACTGAGTATGGTGTCAGAGAGTGTTACCGTGGCCTTGTGCCTATACTACTCCGCAATGG
CCCTAGCAATGTGCTCTTTTTCGGGCTCCGGGGGCCCATTAAGGAGCAGCTCCCAGAAGCCACTAGCCGGGCAGGTCA
CTTGGTGAATGATTTTGTGTGTGGAGGGGTGTTGGCGCAGCCCTTGGCATTATGTTCTATCCATTAAATGTGGTAAAG
TCTCGTGCTCAGTCTCAGGTTGGCGGGGCCTTCCAGCCTTGTGGGAAGGTGCTGCTAACAGTTTGGAGAGAGAGGGGT
GGCAGCTTGGCTATGCTCTTCAGAGGGGCCCACCTCAACTACCACCGTTCACTCCTCTCCTGGGGGATCATCAATGCC
ACCTACGAGCTGCTGCTGAAGCTCATATGAAGAGGGGGTGACAAAAAGAAAGAAAGAGAGAGAAAATATTTAAAGGGA
AAAACCTGTAAGGAGTAATAGAGCCAGTTTGTAGAGAAGAAGGGTAATTGTAAAGTAAAGCCGATGCACTTGCTGTCT
GGCATTGTTTGGAGTTTCCGTAACTGAAAGACAGCAATGAAGTTGCACTTCTGACAGGGGCCAACAGTCATTGTGAGA
TAACCACCTGCCATTGTGACTATTTACTGTTGTCAGAGTATTAAAAACTAAACACACGCTTTAGCCTACACAAAACTT
GTGCCAGGGTCCAATTCAAATGCCCTTGTTTGCTCTGTGTACAAAAATTTTGTGGAGCGAACTCCAGCGCTTTTTTTC
ACAGTTTCAAGCAAAGCTTACTTGACTGGATAAACTGTTTTATAGGAGAAGCTGTTTCTTTCTATGTGCTGTGTGCTG
TGCTGTTTTGTAGACAATATCCATCTGTTTTGTTTTTTTGACCTAGAGCACTTTGTCTGTGAAGCTGAATTTCTGCCA
TTTAACTCAAACACTTTTGTGCTTTTGACCATTGAAATACCCCAACAATTACTGTCAAAAAGAATGTAATAAACTGTG
TGAGCGTTGGCCCTTTTATTTCTCGTTATACTCACTCTCCACCAATTCAGAGTACATGTCGAGGAGATCAGGAGGCTA
AGAAATTGCTAAAATGAGTATTGGAGATGTACATATTCTTGGACATACTGTCCAGCAGACACAGGCTATTTTAAAACC
ATTTCAACTTTCACACATAATTCATCTATGTGAACTGCAGAGTCAAGTATTGTTTGTCTTGATTTATTTTCTGAAGTG
CTAGTGGCAGGTTGTGTCTGTTATTTGAGTGTTGACAAGTGCCCATTGATGTATATGTCAGTAAAACATACAGTTAAC
AC

>Sequ06616EST2
ATGCCTCTATTGGAGACGTGGAGCTCTGCTGTACATGTACTGCCACACCCTTCATCAACGAAAACAGTGGATCAAGAA
AAACAAGGATACATTCCTTAAGTGTATTCAAGAAGGTGTGCGCTACCTGATGCGGATGCTGCAGGTGAGAAACTCTGT
GAAGCTCAATGATGGGGTGGTGCTCCATGACACTGCTACAGCAGGCTTCCTGTCTGAAGGCATCTTCTCAGACACACA
CCTGTTGACGATGATGTACATTGGAGAAATGTGTTTCTGGGCAGTCAAGTATGAAGACTGCAGTGCTGACACAATGGA
TCGGAAAGAAGATCGGCTCCAGTTTCGGGACATTGGCACTCAGATCCTCAACAAATATGTGCTTGCCTGCGAGGGCCC
TCTGCAGGGCCAGGGCTGGAACACGGAGAATGCCAAGGAAATCCTTAGTATTTTACAGTGAACCAAGATGCTCCTGTG
TTGAAGTTGCCCTCAGGCACAGATCTAAGTTCAGTATTCAAATGTGTGTCAACCCCAATAAAAATTGAACCCATCAGT
GGGATGTAATGTTGTGGAAGTTAAGGGGAGGCACAAGGAGTATGTGAAGAGGAGTCTTTGGATCAGTGTCTACAGGCA
ACCTAATCCGATTCCAATCGGCTCCTGGCCGAAGCAGGTGAATGAGAGGTTAGAAAGTATAAGATTGGTGGTGTAAAA
CTGGCAGGGGCTGCAGGGGAGGCTGTTGAATAATTTTTTGCCCTGTCCTGCTGAAAAAGCAGTTATCATCCTGATTAC
AGACTGTTGACTGGGGCACTTGTTTTGCTCTAAGAATGTGTTATAATAACATTAATATTAATATAAAAAGGTTATTAA
TAAAATTGACCTTTTTTTCCATTTTTTAAGATTTGTTACAAGTTTTAAAGATAATCCAGCTAGGAAACAGACATTGCA
CAGTAACATTTTTA

>Sequ06687SNP2
AAACGCTCACTGAGATTTCTCAGTCTCTCTGGATGTTATCAAGTCACTGATTTGGGCTTGAGGGCTCTGTCTCAGCGT
GGAGGGCTTCCTGTCCTGGAGCATCTCAACTTGTCCGGCTGCCTCTTCATCACTGAGGTGGGGCTGCAGGAGCTGGTG
TCAGCCTGTCCTTCCCTCAATGATGAACAGTTCTATTACTGCGACAACATCAACGGTCCTCACGCAGACACGGCCAGC
GGCTGCCAGAACCTGCAGTGTGGCTTCAGAGCCTGCTGTCGCTCTGGAGAGTGATCCTGCCAAGCAACGCTCTCCTGA
AAATGTCACGTCTCCTTTTTATTGCACTTTATCCTGGGAATACCATTTGCTGTACTTTGTGTCACTTAAAAATGTTTC
TGGATGAAACTGTAATTTTGCTCCTGTTTTTTCCATTTCCATCTATATTAAATGGACACGATCCTTCAGAGAAAGAAA
ACAATGAGGGGTGATGATGAATTTGAAGCCTCTGCAAATGTACATCCTGGCTCCCTTTTTTTTTGGGTTTTGAAAAGT
AAAACTAGAGAAACATGACACCAAGTACGTAGTGTTTTCTGTAGTTAAACTGTTTCTTTTCACTAAAACTATACTCAT
ATGTAAATCTGCTTCTCCCCTTGTTTAGTTGACCCATTTATAATCAACAAAGTGTTAACTGACTGACTGTACTGTCAA

AGTGTTGTTTTACTTTGTTTTATCTTAAACAAGCTTTGTCATTTAATGGAGAGAGTGATGTGGAAAATTAAACAGGAG
TGAACCGTCACTGCCTCCACTTAGACTGATGTGAACAGCTGTCATGTTGATCTGCATATACGTTGATGAGCCAAGTGA
CCACTCACAGATGTAGAGAATAACACTGAATGTGGCATAATTAAGACATTTAATAGGATGATATTTTACTGTATTAAG
TAAATAACAAGAGGTCTCTACAGCACGTTTTCACACAGCGTCACACTTTGATAAGATGTCTGGCATTAAATTATATGG
GACATCTCGGGATTGCTGCATAATCTCTAACAAAAAAATGTAAGTCAGGTCAATAAGTCAAACAGAAATGATTCCACA
ATAGTTTTTTTTCTTTCAATAACAGACTGGAATGTAAAGTCCCAGCATTGTTTATGTGTTGGTCATTTGAAGTCATG
AGCAAACCATGTGTCACGGGCTGCCTGACTCTTGTTACAGCGCCTCTTCACTTTTTTTCACATTTGTATGTTGCAGCC
TGATGTGAAAAATCATTAAAATAAATTAATTTTTTACCCCAAAAAAAAAAAAAAACCGACCTACCCTACTAG

>Sequ06696SNP2
TTATCTTGTGGTAATCGTTACAGTCATCCATACTCACAAACTTTATAAATGAGTGCAGTTGTGAATGGATGTGATAAA
TTTAATATTGGCTTGTTGTGAAAAATTGCATCACTGTCAGTGTCTTCCCAACCTGTGCTTTCCTCAGAATTTTTAAAA
GTTTGATATGTTTTGGTTTTCTTTCAGTAATACCAGCAGTGCAACCAGTGGTTCGCTTTGTGGAGTAAATGCAGCAGC
TAGAAAGGGCTAAACGACAGCTCATCATGGATAAAACAAAATCAGGATTGCTGGATTTTGTAGAGTACTAACACTGGA
TAACATAGGTCTAAAGCGTTGAAAAAATAACTCTGGAGGGGGTTTTTATACTTGATTCAAAGGGTCAGAGCAGGGAAA
ACAGCATAACCCTCCTTTTTACCAGCATGTCATCTTTACATTCAAAGCAGGTTCAGCTACTTCCAGCTAAAAAATGCC
CATGAAAAGACGGTGTCAGTTGCTGTGAGTAGCCACAAAGTATCACGTATTCTTTTTACTGTATGGCTTAAAAGTGTG
ATATTGACCTTATTGTAGCATTTTTTTCTGCCTTGTTTACTACAAAACTTCCCAGAATGGAAATAAAGTGTAAAACAC
ATACACACAACAACAACAACAACAACTTATGGCATGAATAGTGTCATGTATGTGTTTTCATTTTGTTTCTGTGCCTTA
TTTTATCACAGCCTTTCAGTGACGATCATCGATAAACAACCTCACACTTGTGTTAATCCTGTATTTCTGTGTGTGTGC
TTTTGTGGAGATGGATATTCATAGGTTCGCCTGATCTGGCAAGTATTTTTTTGTGAAAGAAAAAGAGATGACAGACAG
AGGATAAAAGGGACTGTGTTCTGTGATGGCAACCATGTTTTCAGGTGAAAGATATGAGAGAGCTAAAGTACGTCTTGA
CTGTTTGTTATGGAAGAAGGGAAATGGTCTTACCAGGCATATACTCACACTCCCTGTAATAGTTTTATCATTTGTTAC
AGTCCTGCACCAGATGAACTTATAATAAAGTTTAAATATATAATATACCTGTTTGT

>Sequ06704EST2
TGAGCCAGAAGAAGGTGGAGAAGAACGTATTCTCCTTCTACCTGAACAGGAACCCAGACACTGCGCCCGGCGGTGAGC
TGCTGCTGGGAGGAACCGACCCCAAATACTACACCGGTGACTTCAACTACGTCAACGTCACCCGCCAGGCCTACTGGC
AGGTCCACATGGACCGGATGGCAGTGGGCACCCAGTTGACCCTGTGTAAGGACGGCTGCGAGGCCATCGTGGACACCG
GGACGTCTCTGATCACCGGCCCCTCTGCCGAGGTCAAGGCCCTGCAGAAAGCCATCGGAGCCATTCCACTCATACAGG
GACAGTACATGGTGATGTGTGACAAGGTCCCGTCGCTGCCCGTCATCACCTTCACCGTGGGCGGACAGAGCTACAGTC
TGACCGGAGAGCAGTACGTCCTCAAGGAGAGTCAGGGTGGAAAGACCATTTGCCTGAGCGGCTTCATGGGTCTGGACA
TCCCCGCCCCCGCCGGGCCCCTGTGGATTCTGGGAGATGTATTCATCGGCCAGTACTACACCGTGTTTGACCGAGAGA
ACAACAGAGTGGGCTTTGCTAAGTCCAAGTAAGCACAAATGGCACTGAAACAATATGTTCAAGTGCAAAGCTAAAAGT
ACGAGTTTCAGGAGAGAAAAATTCAGAGCTGCACGTTTTCTGTTGCTTTGCAATCAAAAAGTATTTAGCAATAGATGT
TGTGTAGAGCATAAATGCACTGATTTCCAGCAGCGTGCGTGAACCAGACTGGAGAGTGGATAGGTTGTGTGATGGGTT
CATATGATCAGGGATGAGTTGATTGTATCGGTACTGTCCCTTTTCAAAC

>Sequ06738EST2
AGACGCGGAAGTGATTTGCAGCTGTCAAACAGATTTTTGTAAATCAGTTTCAGTTTTCTGCTGAAAGGCGACTAACAC
TTGACGTTACTGCAACATGGACTGTCCACGCTGGCTGCCTCTGGAGTCAAATCCAGAAGTCATGACAAAGTTTGTTAA
TTGTTTGGGTATGAGGCCTACCTGGCAATTTGGAGATGTATATGGATTGGATCCAGAGCTTCTCAGCATGGTGCCAAG
ACCTGTGTGCGCAGTGCTACTCCTCTTCCCAGTGACAGAGAAGTATGAGGCATTCAAGCAAGAAGAAGAAGAGAAACT
TAAGGATCAGCGACAGGACGTCTCGCCTGACGTCTACTTCATTAAGCAAACTATCGGAAACGCCTGTGGAACAATAGG
GTTAATTCACGCAGTGGCAAACAACCAGACACATCTGGACTTTGAGCCTGATTCTGCTCTTAAGAAGTTTCTTGAACA
AACCTCTAAAATGACCCCAGAGGAAAGGGCTGCCTTCCTGGAAAAAGATGAGAGTATACGTGTTACACATGAGTCGAG
TGCTCAAGAGGGACAGACTGAGGCTCCCAGCTTAGATGAGAAAGTGAATCTGCATTTTATAGCTTTCGTGAATGTCGG
AGGACAGTTATATGAACTGGATGGCCGGAAGCCTTTCCCTATTCTTCACAGAAAAAACCTCAGAAGATACTTTCCTCGA
GGATGCTGTAGAGGTTTGTAAGATCTTCATGGCTCGCGACCCTCAAGAAGTCCGTTTCACCATCATTGCCCTCTCCAA
AGATTCATACTGAGGAACCTTCCTTGTGACCTGAGAAAACAAAGAAAAGATTACTGCCAAACATTCTCAAGTGCTGAC
TTCAATGGAAAATTAGATTTCTTTTTCAAAATACCTGAGCAAGTGATGTGGGGAAAATCATAAAATGTCAGGGATGTC
ATTCTAAAGTAATGTTATACATCACTTGTTACGCGTTAGATGAAATTGCAAGGGAATGCTGCTGTGCAACTAAAAGTA
ATTCTTGCTAAGATTAAAATCTTCCAGTTCGTCACTGCTGAAACATCTCTAACTATAATACAGATATCAGCTGTACTG
CATTGACACTGCACTAAAGTGTTACATGGCGTGTTTGGCTTTCAATTGCGAAGGAAAATTTAACACATTTTTCATGTA
ACAGAGGGAACTCCTCACTTCTTTTTTTTTTTTTTTTTAATTTTTTTTTTTAATTCTGGGTTCAGAGATAGATACCGT
ATGTAGTGATTTGCTTTTGATCATATGTCTAACACATGGT

>Sequ06759SNP2
AAGGATTTGAACTCTATTTCTAAGATCATGTTTACATCCCTGTAGGATTTGTGGACCAAACGCTCCCTTTAGATATGT
TATAATGGTCTCCAGTGAACCAAACGTACTCAAAAGTTGGTGTACTCTTGAACAAATAAGTGGTTAAACATTTCAAGT
AGTTGGCTGCTTAATTCTAGCATCCAGTTAGGCAGGATGAACAGCGTCTTTCTTAACATCACATTTGGTGCCAATGTA
TCTGCAATGCGCTTCGGGAGGTGGTTGTTCACTGTTGTATTAGCACTTACCTGACATCTCCTACGTTTTCCGTAATTT
TACTGTATAACTTGAAAAGCTTCACCAATGGGTGTTCTGCATTGCTGACTGACTGGGGCAAAAAGACGCCAAAAACAT
TTATAAGGGAGCATTTTCGTAGGGGAGAGTAAACGATTGGACCGAAAAGATGTTGAATCTTCTTCCTTTCAACCACGT
AGCTTTTCCTTCCTTCCTCTGTTATTCTATGTAAGTTTCACTGATTGTACTGGGCATGAAGATGCAGTAATGTGACTA
TGCACTGAAACCCTGCCTCATTCTGAACAGACATAAAGGTCATAGCTGGTGTGTTTAAATTGACCGGACTCAGTCTTA
CTAGCCTTAGGGAACAATCACCACATGGGAAAAATGAAGGCTTCACTTATTTGTGCTTTTCTATGTATTTGCATCATA
CGTTTGATTAATCTCAGTTGAAATGTTTTTTGTATCGCTTTCATGTTCTTTTTTTTTTTAATTTTATTGAGAATAACA
TGGTTTACTTCTATTACTCGTAACATCTGTCAAACCCCCATGCTTTCATGATTGTGTTGTTACGGTTTGGGATCCAGA
GGAATGACAGCCTTCACACACAATGTTTGTGCACTGTTTTGTAGGTCGGTGGTTGTAGATGTTTGCCTGTTATTGATG

TATATTCACTATGAACCATGTTTTAGACATTCCACGTCTCTCTCTGAGCTCCTGCTTTGCTTTTGGACCAATTCCCCT
TGGCCAAAAAAAGAAGAAATTAGGGAATAAAATGCTGTTGTCTTCCAAGAAGATTGTGAAAAGTGATTTATGGGTTAA
AAAAATAAAATGGAGT

>Sequ06770SNP2
GAGTTAGCTTGCGGCTACAGCTGAACTGAAGACAGAAACATGGTGCTGCTTGAAAATGACTCGTTCCTCACAGAGCTC
ACACGGCTCTTCCAGAAGTGCAGGACATCTGGGAGTGTTGTCATCACACTAAAGAAATATGATGGGAGGACCAAGCCA
GTGCCTAGAAAGGGCCACTCAGAGACATTTGAACCAGCAGACAACAAATGTCTCCTCAGAGCGTCTGATGGCAAGAGG
AAAATTAGCACAGTGGTCAGCACCAAAGAAGTAATCAAGTTTCAAATGGCGTACTCCAACCTCCTTAGAGCTCACATG
GATGGACTTAAGAAGAAAGATAAGAAAAGCAAAAGCAAGAAAACCAAAGCCACCCAATGAGCAACAGACTCTTTAACA
TATCCATGGTTACCTTAACTGGCCTGTGCGATCAGTGGGGGAAATCGGGAAAGGCCTCCGCTTGGTTTGTCGCCCCAC
CCCACTTTCTCCCCCATTCATCTGAGGCCACAGAATCACTGTTCATCCATCCGGTGACTGCTACGATATTATGCCATT
GATTGCATGGTAACATCTCATCCCTGGCTGTTCATTGCCACCGGTGATCTGAAGGAGGATTTTTCAAAACTCATACAA
GATCAAGCAGGTCAGCTTTATCTCCATAGCTTATGCTTCAGAAACCAGGTCATCAACAGCTGCGTCGCCTCTGGAGCC
AAATCCAGACGCCCAATGATTAACTGCCTTTCATACTATTTAATTGTTAGAGTGAGTCCCTATTTATGTTATATTAAC
AGCTAACCTGTGAGCCCTGTTAGCACATTTCAATTGGACTATTTCTTTAGAAGGATGTTAAATTTTCCAGTTATTTGA
ATTTTCTTCTTTTAAATATAACATTTTTGCTTTGAAATTTAAGCATGGGTGCTCAAAAAATGCAGAAGTAAATATGAC
CAGGGTGTTTTATTTTTGTCAAATTTCACAGTATTTGACTTGGCTTGATATTGGAGATGAAATTGTATGCTATGTTTT
TATTTTACTTTTTGAAGTACAATGAATATTCTTGTTCCCCCATGTCAAGCTGTCAAACACCAATAAAATGTACAGTAT
AGCTTCAGTTAATGTGGTGAATTCAAATTGACATTTTCTGAATGATGTCATGATTAAGTTAAATAGCACAAAAAACGT
ATAAAGTTGTAGATTTACTTTAAATAATTTCTGAGTTATGAATACAGAGACAAAGTATTGGCTGTAATTCTAATCATT
TCTCTTTATCACAACTACAAGGCAGGTTTAGCCACAGCCAGTACACTCTGTCAGCCATTTTGTTGTCATGTCACGCTC
CCAAGTCCTGATCTCATGGAAAAGGACTGCAGCAGAATTTAAGTTCAGAGATAAAGTGCTTTGTTAAAT

>Sequ06777EST2
GGTTTGGGGATGTAGCTGCAGAAAGCTGTGTGCCACTTCAGCGGGTTGGAGGAGGTGGGGGTGACCTTCCTGTCCTGG
TCCCCTGATGGCAGCCACATCCTGGCTTCTACACCGTCTAACCTGTTCAGGGTTTGGGAGACCAGGATGTGGACCTGT
GAGCGTTGGCCATGTGTGAAGGGGCGCTGCCAGTCTGGCTGTTGGAGTCCAGATGGGAGTCGCCTGCTTTTCACTGTG
CAGGGAGAGACGGTCATCTACGCTCTGACCTTCACTGACACACCAGGCATTTCTACAAGCACATCAGCAGGGCCACAG
GCAGCAGCAGTGGTGGCTGACCTATCAGAGACGACCTTTAACACTCCAGATGGAGACATCATTGTTGGTGGAGAGATC
CAGTGTTTAGCCTGGGATCCCAGAGGAGAGAGGCTCGCAGTGCTTCTTAAAGGTGATCCACACGCAGCAGACCGGCCT
GCAATCATAGCAGTGTTCAAGACAAGAACCAACCCCATTTTTGAGCTTTTGCCATGTGGTTTTGTTCAAGGAGAACCT
GGTGCGGAGCCGAGACTGATGCAGTTCCACCCGAATTTCCAGCACGGAGCTCTGCTCACTTTGTGTTGGTCCAATGGA
AGAATTACCCATGTGCCTTTCTACTTTGTGAGCGCTGGCATACCCCATTTTGGCCTGAGTGGCAGTCCGTTACTGCCT
TGCCCACAGGAAAGGCCTTCTGCCCTTGCCAATCAGTCGCTCTTTACAGAGTTCATCTCTTGACCGACCATCACACAC
CCATGAGTCATTTTTGCTATCACAGATTGTCAATAAATGTGTAGAAAGTTCTTGTTATACTAAGACTTGTTTCCTTAT
TTGTCAAAACATTGAACCAAATAGAAATCATTGTGAGGTCAAGATGAAATGTAGAGTTCATGCTCCAGGGCTGAAGTA
TTTGCACTTAAAGTTAAAAGAGTACTCTGCTGATTTAACTTTTGTACTTTTACAACATTGTCGGACTCTCAATGGACA
GTTTTTTAAAAAGAATCAATCACTCACAATCACTGCATCAGAGCCTGAAATATTCGACATTTATGTTGTGTGCACACT
AGAAAAGTGACAAAATAAAGTATACTCAAAGCTATTTTTTTTTGATTGTGCTGCTAATGTTTGGAGGAAATGACAATA
AATCTGAGCAAAATGATGCGTTCTGCTCCTTTCCATGATAAG

>Sequ06799SNP2
TGTTGTTTGTTAAATACTGATGACACTTGATCTGATTGGTCCAGTGCTGATACTCAACCAGGAAGTGACAAAAGGTCA
AAGTTCAACAATGAGCCATAAACATCAACGTAACTCAGATCCAAACTGATCTGACTCACGACGCTGCGTTCAGCTCCA
GTCACTGTTGAATATGTTGAGTCGCAGCATCAGATCGTCTGATTCAAGGGACTG

>Sequ06811SNP2
ATAACTTTGTGGCCGTTTCTCTGCACCCAGAATTGGTGAGAGAATTTCGTGTATAAACATTTTTACTGTGGTGATATG
AATTACTGGGATTAAGTCAATGAAATTCTAGTTTGGGAGGACTGTATATGCTTCAAAATTCACTATAAGCAGGGCAAC
ATTAGAAAACGTATGAATCATGAGTTTTATTCATTGCTTGTGAAGCACTGACTCTCATCAAATATAAGGGTATTATTA
CAACACTGCTTCAT

>Sequ06823EST2
GAGAGGATGGATGAAGAGTTTACCAAGATCATGCAGAACACTGATCCCCACTCACAAGAATATGTTGACAATCTGAAG
GATGAGGGACGGGTTTGCGGCATCATTGACCGGCTGCTGGACTACTTGGAGAACAAGGGCAGCACAGAGGAGATTTGC
CGTATCTATCTGCGTAGGATCATGCACACCTACTACAAGTTTGACTACAAGGCCCACCGGCGCAGCCTGGGCATCCAG
GGAGAGACCAAGTCCGAGCAGGACCAGGAGGAGAGCGAGGGGGAGGACAGCGCTGTGATCATGGACCGTCTCTGCAAG
TTCATCTACGCCAAGGATCGCACCGACCGTATCCGTACCTGCGCTATCCTCTGCCACATTTACCACCACGCTCTGCAT
TCACGCTGGTACCAGGCCCGCGACCTGATGCTGATGAGCCACCTGCAGGACAATATCCAGCACGCTGACCCACCTGTA
CAGATCCTTTACAACAGAACCATGGTCCAACTGGGCATCTGCGCTTTTAGGCAGGGCATGATTAAAGATGCCCACAAT
GCCCTGCTGGATATCCAGTCCTCTGGCCGCGCCAAGGAGCTGCTGGGTCAGGGTCTGCTCATGAGGAACATGCAGGAG
AGGAACGCCGAGCAGGAAAAAATTGAAAAGAGAAGACAAGTGCCATTCCATATGCACATCAACCTGGAGCTGCTGGAG
TGTGTGTACCTGGTGTCAGCCATGCTGCTGGAAATCCCCTACATGGCCGCCCATGAGTTTGATGCCCGCCGCAGGATG
ATCAGCAAGCAGTTCCATCACCAGCTGAGGGTGGGAGAGAGACAGCCACTGCTGGGACCCCCAGAGAGCATGAGGGAG
CATGTGGTGGCCGCCAGCAAGGCCATGAAGATGGGAGACTGGCGTACCTGCCACTCATTCATCATCAATGAGAAGATG
AACAGTAAGGTGTGGGACCTGTTTCCTGAGACGCAGAGAGTACGCGAGATGCTTGTCAGGAAGATCCAAGAGGAGTCT
CTGAGGACTTATCTGTTCACGTACAGCAGTGTGTACGACTCCATCAGCATGGAGACACTATCTGAGATGTTTGAGTTG
GAGATACCCACAGTTCACAGCATCATCAGCAAAATGATCATCAACGAGGAGCTGATGGCATCACTTGACCAGCCCACA
CAGACTGTTGTGATGCACCGCACAGAGCCCACCTCCCTGCAGAACATGGCTCTGCAGCTCGCTGAGAAACTGGGCGGC

TTGGTGGAGAACAACGAGCGCGTCTTCGACCTCAAACAGGGTGTCTACGGAGGCTACTTCAACAGAGATCAAAAAGGT
GGCTACCAACAGAAACAGTCCTACCAGAGAGATCAGAAAGGTGGATACCAGCAGAAACAGGGGGGCTACCAGCGGGGC
GGCTACAGAAATCAAAACCAAAGCAACTACTGAGCTTGCAACGTGTGGATTTCAGTCTTGCCACCTGAATTAGTCTTC
ATTCCATAACAACCACCACCTGGGTTTCTGTGCTTGGCACTTGGCTGTGTGCTGTTCTCCCTCATTTGACTCAAAACT
GATCCAGAAATAGCTGAAAGTCATTGCAGCACAAGCCTTTCTCACTGCAACTGCACGGGGTGATATTCCAGTACATGA
AGAGTTTTAAGGTTATTGATAATAGAACATTGTACAAAAAGGGCATTGCAGTGTGGCTAGCTTGTGGCAGTGCAGTCA
GACGATAGACGCTTCAAGTATTGTTGACTTATACTTAACAAATAAACAATAA

>Sequ06852SNP2
ATCAACATAAAATAATAGCATTTAAATTCTGTCAATCATGAAAATCTGCAGATAAAAAATGTAACTGACCTTGTAATT
CCAAACTCAGTTTTTTACCAGTAGATGCATCTCTACCGTTTACAATATATTTCAGGGAAAACAATACTTGGAGCCAGT
TTGATAGGAAGTTTGACCTGATCTGTTTAATCATGAAACCATAGATGGGCTTACTGCCCTCCAGCACCAACATGTCTA
TAAGCAGTTTAACT

>Sequ06859EST2
ACCGGTGTCCCACGACTACGGCGACACCGTGGCCCTGGAGCTGCTCTATAGGAGTGACCAAAATCGCACAGGTCACCT
GACCTTTAACAGCCTGTGTCTTTCCAACGGAGGCATATTCCCAGAAACACACTACCCACGGCTGCTGCAGACGCTTCT
GAAGGACTCCAGTTTTCTGGCTCCGCTCCTGACTCGTCTCACCAACTATATGATCTTCCAAAAGGGGGATCGGGGAAG
TGTTCGGTCCATACACGCAGCCCACAGACGCTGAGTTCTGGGACATGTGGACGGGTTTGCGCTACAATGATGGCAACC
TAGTTTTGGACAGTATTCTCCAGTACATCAACCAGAGATTGAAACACAGAGAGCGGTGGGTGGGTGCGCTCACTTCCA
CCTTCGTCCCACTGCACATGATCTACGGACCCCTGGACCCGGTCAACCCCCATCCTCAGTTCATCCGCGTTTACCAGC
AGCTGGTCCAGAGGTCGACAGTCACCATTTTGGATGAACACATCAGTCACTACCCTCAGCTGGAGGATCCCACTGGCT
TCCTCAATGCATATTTCAATTTTATTCACTCTTTCTGACGGGAAATGCAACGGCCATGTTCAAGAATGATGCTAAAAC
CTGTTCAAGCGAGTGTACCAAAGTGATAAATGATCACATGTGGTGGATAACACTCTCCTGATGATTGTAACCAGGAGA
AACGCTGCTTTAAACTGAGATTTAATCACAACTGATGGGTTACTTTACCAGCATTAATCTCTGAGATGTCAGGTCCCA
TCACAGACATAAAATC

>Sequ06880SNP2
GCTGCAGACTCGGGGGAAGGAGGTGGGCGTGACCACAGGCAGGAAGAGACGATGTGGTTGGCTGGACCTGGTGCTCAT
CAAATATGCACACATGATCAACGGCTTCACCGCTTTAGCGCTCACCAAACTTGACATACTTGACGTGTTCCCAGAGAT
CAGAGTGGGCGTAGGCTACAAAGTCGACAACCAAACTATACCTCACTTCCCAGCCAATCAGGAGGTACTGCAGCGTGT
TGAGGTCCAGTATGAGACGCTGCCCGGCTGGAAAAGCGACACCTCGGCTGCTAGAAGCTTCGCGGAGCTGCCTGAGAA
CGCGCAGAAATATGTCCGCTTCATTGAGGAGCACGTGGGAGTGCCTGTCAAATGGATCGGAGTGGGCAAGTCTCGGGA
GTCCATGATCCAGCTGTTCTAGACGGAAGGGAGGAGAGACACAGGCAAAGAAGATGCACAGCAGCGAAATCAACGGCA
CCCTTCTCCCCCTCAGCCCCACACACAGCCTACCTACCTGTCTCAGCTCTCCGACAGAGACGCGACCATCATGTCTGT
TCTTAACGCTTTACACCAGTGTGTCATCAGACTGTCCCATGTTACAGACCAGACCGTTCTGGATCGGACTCCCATCAG
ACTGACCTCCCCGTGCGAGTGGTTCCCAACGTCTCCATCTGGATTTTTAACAGTTTTAAACTTTAGGATAACAGCTCC
ACAGTCAAGTCCCTCTTTAGTTTGTCCCTGTTTGTATCGTAGCCGTTTATTTCAGACATACAACTCACCAATCGGCAA
CACCAGCTGCCCTCAGGTCTGAGAGAGGACATGAGGGAATGGACTCGACACACGCAGACGCAGACAGGGGATCACTGC
CCGGAGGAACTGTCCACAGATGTTTGGTCGGTCAAACTTTGTTCAAGTGTATTTTGTTAATCTATGCTGTGTTTTAAA
TGCGCTGTGCCAGTCCGGTGTAAACCAGTTAATAAGTTAAAATAAGTTTTAACCTCGGCACTTAAGATGCTTTGGACT
ATTATCTGCTTTATGTTTTATTCTGCCAAGTCAACAACACATCCTGACTTGATAAAGGGATCCTTTAATATTTTAAAA
AGTGTCTTTTTGTGTTTGTTTTTCGTCATCAGACAAATGTGAGAGACTTCTTATATTGCCAAAAAATTAGCTTTGAAA
TTTCTTGTAGCTATAGCGATCGCTTCCAGGGAGCGCTGTTGATGAAATGTTGACGTACATCTTCCCGTACTGCGAAAA
GGAAAGGCTCCTTTTCCTCACTTTAACTTTAGCAGGTGAGTTTTTCATGAACCAGAAAAATAACTATCACTTTTTCAA
ATGAAAATCAAATCATCAGGCCAGCATCTGTGATTG

>Sequ06881EST2
GTTGTGCGTCCCATCAGCGTGTGTTCCAAGGAGATTTTACCTGCAGAGTTATTCGGAAAATGGCGAGCCTGTGTGCCA
GAGCAGTGAGACAGAGCTACTCACTAGTAGCTTCATCTGTACTTACACGAACATCTCCACGATTGTTATGTACGGCCA
CTCAGCAGAAGAATGGCCACAGGTCAGAGGAGGACGCTGAGAAGCCAGAGCAGAGTGCAGCAGAGAAAGTCCTGACGG
AGGAGAAGACCCAGCTGGAGGAGCAGCTCAAGGAGATGACGGAAAAGTACAAGCGAGCCTTGGCTGACACAGAGAACC
TCAGAACGCGGAGTCAGAAGATGATAGAAGATGCTAAATTATACGGGATCCAGGGCTTCTGTAAAGACCTGCTGGAAG
TGGCCGACATCCTGGAGAAAGCTACTGAGAGCGTGCCCAAGGAGGAGGTGACAACCAAGAACCCTCACCTGAAGAACC
TGTACGACGGCCTGGTGATGACTGAGGTCCAGATCCAGAAGGTGTTCACCAAGCACGGCCTGGTCAAGCTGAACCCCG
ACGGCCAGAAGTTCGACCCCTACGAGCACGAGGCCCTCTTCCACGCCCCTGTGGAAGGCAAGGAGCCCGGCACTGTCG
CCGTAGTAACCAAAGTGGGCTACAAGATTCACGGTCGCACCCTCAGGCCAGCGCTGGTGGGTGTGGCCAAAGCCCCCT
AGAAATGATGACAACTGTGACAAAATGGGATTTGTTTTGTTGCCTGGGTGGACGGGGGACCTCAGTTACAGTAGACTG
GTCCCAGCACCCTGCCACCCTCTTTTCCCATGGGTCACTCCCCCTCCTGCCTCGAAGAGACGTGAACCCAGCTGAAAT
GAACTTTGTATCCGGACAATATTCATCTGTTCCTAACAGATGCATAACACTCTGTCTGGCACAGGACAGTCTTTGCAG
TAAAAATTAATTTAGTCATTACATAAGTCATTTCAGGCAGTTATCGTCAGATGTTTAAAAAAGAAACAGCACCAGCTC
ACAGACGGAGGCTTTCATGTCTCCTCATGCTGCCATAAATTTAAACTGGAAGAGAAGCAGTGGAATATATTTCACTGA
CTTATATTAAAAAAAAGTTTACCATCCGATCATAACCCTTCTAATTTCAATACTTTTTGGATGAGAGCTGGTGTAAAA
TCCATCCACACGGTGAACAGTGACTCTGAACTGCTGATATCATTCCGGACTTTTGTTGAAAGCTCTAATAATGTTGAA
ATTGACTCTTCAGTT

>Sequ06882EST2
GAACCGGACCGAGAAATGCCGCCTACTACAGTCCTCAGGGCCACATACTGGTCCTGGCTGGCTTCGGGAACCTGCGGG
GTCAGATGGAGGTCTGGGACGTGAAGAAATACAAACAGGTGTCCAAACCTCAGGCTCCAGATGCCACACATTTCTGCT
GGTCTCCTGACGGTGAGCACGTCATCACGGCAACCTGTTCTCCCCGGCTGCGGGTCAGTAATGGTTATAAGATCTGGC

ACTACACTGGCTCGGTTCTGCACAAATGGGAAGTGGCGGCCGGTTCGGAGCTGTGGGAGGTTCACTGGCAGCCGTTCC
CCGACGGCAGCTTCCCCGAGCGTGCCATCAAGTACCAGGCAGCACCCAGCGAGCTGGGGAGCACGCAGGCCCCACCCA
CTCAGGCATACCGCCCACCTGCACTGAGACATCTGCCGGCCACGCCCAGCGCCAAACTGCATGAGGAGGAGCCCCCTC
AGAACATGCGTCCAGGTGTTTCGGGGGAGAAGAGTCTTTCTAAAGCAGCTCTGAAGAATCAGAGGAAACGAGAAGCTA
AGAAGGCAGCTAAACAGGAATCAAAGTCTGAACATGAGCCTCCGTCTGACCCCGCCCCCGTCACCAACAGCCAATCAG
AACCAACCAGCGGTGACCCGGAGATGGACAAGAAGATAAAGAATTTAAAGAAGAAACTTAAAGCCATTGAGGAGCTGA
AGGAGCAACAAGCATCTGGAAAAGTCCTGCAGAAGAACCAGCTGGAGAAGATCCAGAAGGAGGACCAGCTGCTAAAGG
AGCTGGAGGAGCTGCAGATCGGATAGTAGGGGGTGCTGCTGGAGCTACAGTCTGCAACTCCAAATCCTACAATCCATC
ACTCATGCACAAACACAGAGGAGGGGTCAGTGCGTGTGTTTCACTGAAGCTGCATCTGTTGCTTTAGTGCTGAAATGT
TTTCTGTTTCCTTCACCTGACACCCGAGGACAGGTGAGGAGGGGGCATGGAGTTCTGATACATTTCTATTTGGATCAG
CCTGTGATCAATTCATTCCTCTGGAGCTTCTGCAGAGGAACCTCGGAAATGTAGTTGAACAGTTTGTCACCAAATAAC
TGCTGTGTGTGTGTGTGTG

>Sequ06907SNP2
AAACCATGTGACGTTCTTTATNTCGGGTCACATTCTAAGTGGATAAGAGCGGTGTGATGTAACCTTACTGTGCCAACT
TCACATTTCCATGCATTAGGAAATGAAAATGTGCTGAACGGAAAGTGCACTGACTGACACAGGAGAGCAAACTCCACG
TGGTTTTGTTAAATAATGACTGACCGTAATGTTGGTTATTCAACAGTAGCTGTTGTCGACCTTTAGGACCAGGAAGCG
TTCTCATTTTTCTAAAATCTGCACCACTGACTCTTCAGTTATCCATCATCACTGTTAACTGTTGGTCCATTTTCAGTG
TTGAAATAATTGGCAACACTTTATTTTACAGGTTTGTAATTTCCTAATAATCTACAAGAAAGATTACAAGAAGTAGAG
TTTGTAATTTTGTACTATTTATAGGTAAATGAAGAGTTTAGAAGCAGTTTCCTTTAAAAGAATTGATCCATTTAAACC
AAAACATAGTGTATTTTCATTCTTGGAAACTGTATTCTACATTTTTGCATGTTCATCTGACCTTCTGAGCTCTTACAC
AGGATTATCAAGGTTATATTAGCACTTAATTGGAGGTGAAATGAACAATTATAAATTGTACAAAATGTTAGTCATTTC
TAGGAACCTTCCTATGAATATTATTTAATTTCTTGGATATTAAAGGAGAATATTTGACCCATACAGTAAGGTGTTTCC
AGATAATTCATGTAGCCTTTACAGTTGACTTAAATTAACTATGAACTGATTGTAACAGTCTTTGACAATTGATCCAAC
TACCTTTTAATAATGAGATTAAAAGATCTTATGATGTTTTTGTTGCCAATTATCTTGTGTACGTCTTGTGTGATCTGA
AGGTGTGATTGTTGTTTGTTGTTACTGATAAAGAAATGTTTGTTTTTTTGACTTGAATTAACTGTGGAATAAATAAAG
TTTAATTTG

>Sequ06943EST2
TAGGGTAGGTTCGGTTTTTTTTTTTTTTTAGAGTTGGTTATAACGTTTTATTTAAAATGAACATACATAAGCATACCAA
AGTGTTTTAACAGTAGGTTTGTTCTCCAGACATGAAAGTATACAATATACATTAGATATTGCTACAGGTAAATGGAAA
ATGAACAATATTTGGGTTGAAACTAGTTCTATGCGTGAGTTTGAGTACAGTATAGGGAAGGAAACAAGGAAACTGGAT
CCTGCAGTGTCACTTTCCCGCCTTTGCTGAATGACCAAAGTAAATAATTATACAAATTGAAGTTATCAGTATAACATA
GCTATGAAATCCAAAACCACATAACATGCTTTCTCAAACTCATACATTGAAAAATCACACTAATTTTACTCCAGAAAA
CAAAATCTAATCTAATTGCTTAAAATGTAAATATTAGGATAATCAGAACTTGTCAGAAAAACAATCCTACATCGAAAT
TTTCAAAACAACGGGTGAACCGAATAAAAAAATAATCAACTCTAAACGACAACCACACGCCATCGGCGTGGGAACATA
AAAGGGATCACTGTGTGTTTTATTCTTCATGTTAATGTGTCCTAGTTGAACGGCAACAATCGCTGCTCGTTATTATCA
GGTTTTCTGGGAAGTGACCTTCCTACCTGTTTGGGTTTTTGAGGTCTTCATTATGATTAGGGGTGTCTCTGTCCGTAT
CTCTGCCATGGTTGGGTTTCACGGCAGACGCCCCGTAACTGAATTTGAGTTTAGTCGTTCAAATCCTGGCTGGAGGTA
ATTTGTGGCAGGGGGATGGGTGGTTGACTTTCCATTGTACAGTCTCATCTAGTAGCCGCCGCCGTAGCCCCCCCTTGG
GTAGCCACCACCGCCGCCGCCTCTGGTGTATGGGGCAGCGCTCCTTTGACCGCCGAAGGGCCCTTTCATGGGGCCATA
ACCAGACGAATGCTGGCCATAGCCGCTGCCAAACTCGTTGTAACCGTTTCCACCACCATACCCACTTCCCTGGTCTCC
GTAGCCGCCCCCATATGGCCCGCCGTACCCTCCGTAGCCGCCACAGTTGTAGCCTCCGCCATTTCCGTAGTTGTAGTT
TCCGCCGTAGTCCCTGCCGCCGTAGCCATTTTGATTTCCCCTCATGCCTCTACCTGGTCTACCCCGAGGCGCCATAGC
GCTTCTGTTGGCCGCCTGCATCTCCTGCTTGGTCAGGGCTTTCTTAACCTCAACCTTGTGTCCATTGACAGTGTGAAA
TTTCACAACGACCGCCTTGTCAGCTGCGTCGTGGTCGGTGAAATACACAAAGCCGAACCCCCTTTTCTTTCCGGTCTC
CTTCTCCGAGATGACTTCGGCCTTCTCGATCTGACCGTACTGGGAGAAGTATTCGGTCAGATGTTCCTCTTCAATGTC
GTCTTTCAGGCCGCCGACAAATATTTTCTTAACCTTTGCGAGTGCCTCTGGCTTGTTAGCGTCTTCTCTCGCCACGGC
CCGCTTCACCTCGACAGCATTGCCGTCGACAGTGTGCGGCCTAGCCGCCATTGCTGCATCGGCCTCCTCTGGTGTGGA
GTAAGTTACAAAGCCGAAACAGCGGGACCGCTGCAACTGCTTGTTCACGACGACAACGCAGTCGGTAAGCGTACCGTA
CTGCTCGAAATGCTTGCGCAGGCCATCGTCGTCGGTGTCCACATTCAGTCCACCCACGAAAAGTTTGCAAAGCTGGTC
GGTCATTTTTGAACAACAGGGAGATGATAATTTCTCAACAAAGTCAGTCGTCTGTTAATCTCAAAATGGTC

>Sequ06953EST2
TGCCCTCCGTTGTTCATTCAACATGGCGGCGGGACCAATTTCAGAAAGAAACCAAGACGCCACTGTGTATGTCGGCGG
CTTGGATGAGAAAGTGTCAGAGCCGTTACTATGGGAGCTTTTCTTGCAGGCTGGTCCTGTAGTCAACACTCACATGCC
CAAAGACAGGGTCACTGGACAACATCAGGGCTATGGTTTTGTGGAGTTTCTCAGCGAAGAGGACGCTGACTATGCCAT
CAAAATCATGAATATGATAAAGCTCTATGGCAAACCAATTCGAGTTAATAAGGCATCAGCGCACAACAAAAACTTGGA
TGTGGGTGCAAACATCTTCATTGGTAACCTGGACCCGGAAATTGATGAGAAACTGCTCTATGACACATTCAGTGCCTT
TGGCGTGATCCTCCAGACGCCAAAGATCATGCGAGACCCAGACACTGGCAACTCCAAGGGTTATGCTTTCATCAATTT
CGCCAGCTTTGACGCGTCAGATGCCGCCATTGAGGCCATGAATGGCCAGTACCTGTGCAACAGGCCCATCACGGTGTC
GTATGCCTTCAAGAAGGATTCTAAAGGAGAACGACACGGCTCAGCTGCAGAGCGACTCCTGGCGGCACAAAACCCTCT
ACTCCCAGGCAGACAGACCTCATCAGCTGTTTGCAGACGCTCCCCCACCACCATCTGCTCCGACACCAGTCCTGACCA
CGCTGGGAACTGGGATGCCCATGCCAGGCATGCCCCCTCCTGGTGCTTTCCCTCCTGTTCCTCCTCCGGGATCGATGC
CTCCAACAATCGCCCCCTAGCATGGCCATGCCTCCAAATGCAGGGAATCCAGGCCCACAGGGTGGGGCGGAGGACCTC
CACCCGGACCACCACCCTTCCCTCCTGCCAACATGCATCCAGGTATGCCTCAGATGCCCATGCCCCCTCCTGCTCCTC
CTGGCATGGTACCTCCAACCTCCTGCTCCTCCAGGATCAAATCAACCACGGGCACCGCCACCGCCTGGCATGCCTCCA
CCCCCACCCATGGGCATGCCACCCAGAGCACCATATGGACCTCCCATGGGTCCACCTGTGCCTCCAGGTATGAGAGGG
CCACCTCCACCCATGCCTCCACCTGGCTACGGTGCTGGTCCTCCTCGTCCACCTCCTTTTGGCTTCCAGAGAGGACCC
CCAATGCCACCAAGGCCCCCTGGTGCCCCACCACGCGTCCCAATGAGAGCACCAATGCCACCGTAATCCATCTCAGAC

TGTAGAAACAGGCTCGCATTTAGATTTTTCTTTTTACTACTACAATATGTAATTGTGAGAATAATTTAACCTGTTTAT
TTGTTTGTTTTTTTTTGGTTCTATTGTACAGTTGAAGGACC

>Sequ06979EST2
GGATAAATTTTGGAGGTCATCAAGCCATTCTGTGCAGTCCTGCCAGAAATTCAGAAACCAGAAAGAAATTGTTTGGAA
TGATCATCACCATTGGACAGGCCATTGTATATGTAATGACTGGCATGTATGGAGACCCTTCAGAGATGGGTGCTGGGA
TATGCTTGCTCATCATCATCCAGCTCTTTGTTGCAGGTCTGATTGTCTTGCTGCTGGATGAGCTCCTCCAGAAGGGCT
ATGGTCTGGGCTCAGGTATCTCCCTCTTCATTGCAACCAACATCTGTGAGACGATTGTCTGGAAGGCTTTCAGCCCCA
CCACCGTCAACACTGGCAGAGGTACTGAGTTTGAGGGAGCCATCATTGCTCTCTTCCATCTCCTGGCTACCCGCACTG
ACAAGGTGCGTGCCCTGAGAGAAGCCTTCTACAGACAAAACCTGCCCAACCTCATGAACCTCATCGCCACCGTCTTTG
TGTTTGCAGTGGTCATATACTTCCAGGGCTTCAGAGTGGATCTGCCCATCAAATCAGCACGTTATCGTGGCCAATACA
ACACTTACCCCATCAAACTATTCTACACCTCCAACATCCCCATCATCCTGCAGTCTGCCCTGGTCTCCAATCTCTACG
TAATTTCACAGATGTTGTCGACACGTTTCAGTGGCAACTTCCTGGTCAACCTTCTGGGAACCTGGTCTGACACTTCGA
GTGGAGGACCAGCTCGGGCCTACCCAGTGGGCGGTCTGTGCTACTACCTTTCGCCTCCAGAGTCATTTGGTTCTGTTT
TAGATGACCCAGTTCACGCTGTCATTTATATTGTCTTCATGCTCGGCTCCTGTGCCTTCTTCTCCAAGACCTGGATTG
AGGTGTCTGGATCCTCTGCCAAAGATGTTGCAAAGCAGCTGAAGGAGCAGCAGATGGTGATGAGAGGGCACAGAGAGA
CCTCTATGGTGCATGAGCTTAACAGGTACATCCCCACAGCTGCTGCCTTTGGTGGTCTGTGTATAGGAGGGCTGTCTG
TCATGGCTGACTTCTTGGGTGCCATTGGCTCGGGCACAGGGATCCTCTTGGCTGTGACCATCATCTACCAGTACTTTG
AGATCTTTGTGAAGGAGCAGAGTGAAGTGGGCAGCATGGGAGCACTGCTCTTCTAGAAAATACCAACCTTCTGACGCA
AACCAGACACAGTTCATCCACCCCTCCCATCTTTTTTATTTGCATTTTTGCAGTTCGCACAATCAGTACATTTTCCAG
CTGGGATAGGTAATTTTCTCTGCCACCTTTGTTCCCCACCATATTTCTCTAGCATGTCAATGCTGAGAAAACTGTTTG
GGTTACTGCCAGGGTTGAGCATAGTATCAGTCCTTTTTATCCAAGAGCTACTACCCACTCTCTGCAGGCTGCCTAAAT
CCCCACTGACTGTCTCTTCAGCTGAAAAATGCCTATAAACACACAAGTGTGAGGTGTTGTCTCTGACTTACCTCAGGC
ATTGTTCATTGCAGCATGTACAAGGAACACTAGACACTTCAGACAGTGACAACATGTAAGTGCTTATTTCAAGCTGAA
GGCACTGTGCCTGGACTTTGCAGGGGGAGTTGAAAGAAAGCCATGTCATGTTCATCACATAACAGTCGTGCTTGAGGT
TTCTAGGGCACTATAGTAGGATGTGAAAAGTATAAATAAAGTATGCTTACACTCCTAATAATGTTTCTGGTAAACTCT
GACAAACTGTTGGTGGTTTTCATTTATCTTTATTTTGATATTTTAAATGATGGACTATGTCTATATTCACTCTGGTCT
TATGTAGGGCACAGTGTTTGTCCTCTGTCTCTGATTCCTCAGAGGAAAGTGTTTGTGTGTGTCGTTGTGCTCTTTTAT
TCCCAGACTGAATCGCTGCACAATACTGCCGAGGTGGTCGGGATGAGGGTGGGTTTGATAGGGATGAAAAAGGGGTTT
CATTTTGGTTTTACTTTATGTAACAGACATAATTGTGTTATTAAAAAAAACAGGATCTTTTTT

>Sequ06980EST2
GGGGCGTCGGAGCGGCGGACGGGTGAGTAATGCCTAGGAAATTGCCTTGATGTGGGGGATAACCATTGGAAACGATGG
CTAATACCGCATAATGCCTACGGGCCAAAGAGGGGGAAATTTTCATGTTCCTTCCCGGCGGGTGGTGTGCTGAGCCTG
AGAAGACTTGGTTTATTTAACCAGCGCGCCAAGAAAGATTTTCCTCAGGTTACCTCGTGGTTCAGATCAGCCACCTCT
AAAACATCAAGCAGCCCTGCCTTAAACTCCACCTGCCAGCTCTTTACTGTAACCAAGCAAACCTAAAGCCTTCAGAAT
GATGCAGATCAGCAGCGACTCCGCCAGCAACAAGCACTCCCTCATCAACGTCATGCACCGCTTCATAGCAGCTGCCAA
CAATATGGACGAGACCATCATGGTGCCCAGTCTGCTGCGAGACGTGCCGCTGGAGGAGCAGGCGGCCAGCCAGGTGGA
GGCCAACAATAACAATGAACCGCCGTGTCCCAACAAGCAGAGGGATATGTATGAGCACTACCTGCTCCTCAAATCCAT
AAAGAATGACATGGAGTGGGGTCTGCTGAAGAGGGAGATGAGCAGCGGCGCCAGCTTCCTGGAGATGGCGGTGAAGCA
GGAGGAACAGCAGCCGGTCACCGGGGATCTGCTCCCGGACGACAACTTGGACCTGGAGCATCAGTTTCATTATCACCT
CAGAGGACTGTTTGGAGTTCTGTCCAAGCTCACAATGCAAGCAGACCACCTCACCAACAGATACAAGAGAGAAATAGG
AGGAGGAAACTTCATGAGATAGAAGTCTCTACTGTCCCCCTCCCTCCCCACCCTGACTGCTTGACCTCCAACAATGGG
ATGTGTTGAAGGGGCCCCCCCATCTCTTCTTAATAGATCTCAATGGACAGTGAACTGACCAGGGCAACCACTTTGTGA
CTTCTCACTCCTGTGTAGCGACACTCCAACTTCCCTCTTCTTCCCCAAACAAAACATACCTGAAACCAGTGACGCCTG
CACATGTCTACCTGCGCCCGTTCGTCAGTGCAGTGGTTTGACATTTCGTGCTTGGTGGAGGTTAATTGATTCTGTTCC
CAAGTCCAAATGAAGCACAGCTCTGGTTTATGCAGAAACTTTCCCACCCTTTTTGTTTTCATTTCAAATTTGAACGTG
AATTTGTCATCAGATCGATGTCTGATTGTGAATAAAAGGTTTGACTGTAGGAGATATTGAATGCTGGTTATTCTGTCC
CTCCTTTTTAAAACATGAAATGATTTTAATGCTGAGCTGAAGGGCTCTTAACCCACAGAGGTTTCAAATCTTTCCACT
TGGTGTTACTGTAACATGGTGTGCCTTGACTTTTTGTATACCCTCTTGCCCTGTTCAGTTAACTCCCTCATTGTCTGT
CTGCTTAAATGGTTCATCACGGTATTTATGATGAAATGGCTCATTTGTTTGTCCTTGGATATTTCAAAACCAATTTTA
TATTGACGCAGAAATGCAGTAAAAGTGAAATAAATAATTGGAAACTTAATTTTTGGACTTTGTTTCTTTTGTCACTGT
CAACAGGAAGGATCTTAAAG

>Sequ06997EST2
ACTATAGGAGGTCGTTTTTTTTTTTTAGGGTTAATCACGAATTTATTCCAGAAAAGTTAACATATCCAGGACAAAAACA
TTTCCTATCCCTATGAAAAATACATTAATGTGAGAAATAATGTAAAACACATGGCTCATTTGGAGAGTTTGGACATTT
TAGTACTAAAACATAATTCAAAAGTACAAATAAAAAAAACAAAAAACAAAAAACACATGCAACAGTTTTCAAAGTTCA
AAAGTAACGCTCACTACCTGAACTTCAAAATACAGTGAGTATGAAAATGGAAAAAATTAGATGTGATTCAAAATCTGC
GTTGCTTATTAGGTCCATAGTCTCCAGGCCCAGGAAGGCCTCTGGGGAAGCCACCCTGACCTCCTCCGGTCATGTTGT
TGTTTCCCGGCCCCGGCATCATTGAAGCATTCTCAGCAGCCATGCCAGAAGCACCAGCAGGACCAGAATTACCACCCA
GAGAGTTCCTGTTCATGGGCATCCCATGGCCGGCCATATGCATCCTTATGTCTTGCTCCCTATTTTCAGAGAAGTTGC
CTCTGAAGCCTTCCTGCTGCCTCTTTATCATCTCTTCGTTGCGCATCCTCATCTCCTCTTCTCTCCTCCGGCGTTCCT
CCTCCTGTCGGAGCTCAGCCTGTTTCCTCTTCTGCACCTCCTGACTGTGGAGCTCCTCCATCCTCCGCAGCTCCTCCT
GCCGCCTCAGCAGGTCCGGCCTCATCAGCATCACCTGATGCTCGTGTCTGGCCGCCTCCATCTCGGCCTCCAGCTTCT
CCTGAGCCTCTTTCATGTTGCGGTCCACCATTTCGTACTGCTGCTTCTCCATCTCCATCAGGGCTTTCCAGCGCATGG
CATACTCATACTCAAAGGAGCCTGGCTGAGCAAATCGAGGTGGCTGCTCACGCTCTTTGTGATATTGCTGGTTTTTGT
TTATGATCTTTTCTGACAGTCCCTCCTCTTCATCCATCCGATCCATAGGTTCCACTGTAACAGGGCGAGGAAAAGCTG
TGAGAAGATAAGCACCATCACTGCACTTATCCAGAGCTTTTCTTGCAGCTGGCTTTGAGGTGTACTCCACTATTCCTT
TCCCTGTAGGCCTCCCTCTGTCATCCACTATGACCACAGCTCTTTCTATCTGACCAAACACAGCGAAGGCCTCCTCCA

GCAGCTCGTTGGACACAAACTCTGGCAAATTCTTCACAGATAAAGCAGCGCCGTGTGTTGCAAACCTCACCCGTATGG
GTCTGCCTCTGAACAGAGTATCATCCAACTCGGCTCTGGCAATCTCTGCTATGATCCTGGTCTCCAGCCGGATGAAGC
CGAAGCCTCTTTCCTTGTTGACAAAGATCTCACTGGCCTTCCCGTACTTGGAGAACAGCTTCTCGATTTCCTCCTCGG
TTACTCCAGTTGGGAGGTTTCCTACAAACAGCCTGCTTCGCTGTGTGAAGGTCTTCTCCCCGGGTTTCCTGAAGCTGT
GCAGGTCTAGGGTTAAGGCCTCATTGATGTTGGTATGCTCGCTCGGCTCAGGTTGCTGGCCGTTGGTGTTTGTTCCTC
CGGGGTTTTTTCTGGTCACCCGGGCGGTTGAGGCCGTGGTTCTGCTGGGGGCCTTTGTTTCCCTGCATTTTTGGCAAC
AGAATTCAAACCCGAGTCGATGAACCGCTCAATAAAAGACGCAAACTCTTAGCAGACAGTGGCGCACAAAGAGCTGCG
AAAATGGCGAGGCTGCGTGCTGCCGCGACGCTAT

>Sequ07002EST2
CAGTTTCAAAGATGGCTCTTGCTAGTGTGTTGAACAGTGATTGTGCGGATTTTTCTTTTGGTAATTGTCAGCCTTTTG
CGTCTGGTTCTGGACCCGGACATAGCGGTCTGGGTTTTGAGGCTACTCCCGGAGACAGCCTCAGTTTTTCCGTTAAAA
ACCCGCTGTCTGCCGGGGACGATGATGGCGTCGAGAGGAAAATAGAGTTTCTGCATGGGACCACCACCTTAGCATTCA
AATTCCAGCATGGTGTTATTGTTGCTGTGGACTCTCGAGCCACGGCGGGCTCTTACATCGCATCTCAAACAGTGAAGA
AGGTGATTGAGATCAACCCCTACCTGCTGGGTACCATGGCTGGAGGAGCTGCAGACTGTAGCTTCTGGGAACGTCTGC
TGGCCCGCCAGTGCCGCATCTACGAGCTGCGCAACAAGGAGCGTATCTCAGTGGCAGCAGCCTCCAAGCTGCTCGCTA
ACATGGTGTACCAGTACAAGGGCATGGGACTCAGCATGGGAACCATGGTGTGTGGCTGGGACAAGAGAGGCCCAGGGC
TGTACTACGTCGACTCAGAGGGAAACCGCGTGTGTGGCGACCTATTCGCCGTGGGCTCGGGCTCCATGTACGCTTATG
GTGTGATGGACAGTGGCCTGCGACAGGACCTGACCGTGGAGGAGGCCTGCGAGCTAGGCCGCCGCGCCATCTACCAGG
CCACATACCGCGACGCCTACAGCGGAGGGCAGGTCAACCTTTACCACGTCCACAGCGAGGGCTGGACCAGGATCTCCC
AGGATGATGTGCTCATGCTGCACCAGCAGTACAAAGATCAGGCATAGTCAGGAATAGGGAGGGTTAGGAGTTTATTCT
AGTATCCAATGTTGAAGATGTTCTGAAACGTCAGTAGGGTTGACATATGAATAAAAAAGGACTCATTTGCCGATTGGC
TTCACTTATATCACTTCTGTGGCCCGTTTTTGACTTGTTGCTAGCTTAAATCTTTATATACAAGTTCAGTGAGCTTCA
TTCACCCTGCACGGTATGGGAGTATGATGTGAGGGTCTGGCGGGGGGGGGCTGATGACTCGAACATGCTTTAATATA
GTGTGGGATCAGTGTGGTGTTGCCCTACATACATTTAATTTAGTTCATACCCTGGGAAACTACATATTTCTCAATAAA
ACCTGTCATGTTCTTTTTTCTTTTTCAAGACGGAAAAAAAAAAAAAACCGACCTACCCTATAGT

>Sequ07015EST2
CGGCTCCGGACCCAAATACCTGAAGGGCTGGAACGGTTAGACCCGGGTCAGTTGGTTGAGTTAGCCCGCCTGCTGCGG
TTCCGTGCTGAGATGTCCAGGGTTCTGATCGTCGGAGCCGGGCTGACAGGCAGCCTGTGCGCATGTCTGCTGAGGAGA
GAGCTGCAGAGCAAAGTTCAGATAGTGGTGTGGGACAAAGCGCGGGGCTCGGGAGGCAGGATGTCCACCAGTCGTTCC
CCTGACCCTTCGTCTCACTCTGCCGACCTGGGAGCTCAGTACATCACCGCCACACCGGCCTACGCTCAGTCACACCAC
AGTTTCTACTCGGAGCTGCTGTCTGCTGGCGTCCTGCAGCCTCTCCTCGCTCAGGTCGAAGGTCTGAAGCAGAAAGAC
GACAGTAAGAACTACACAACGCCACTGGGCATGTGCAGTGTGGTCAAACACTTCCTGTCTGAGTCAGAAGCAGATTTG
TTCTTTGAGTGTCATGTGACTGGCCTGTACCGCCGCGGTGCATCATGGGAGGTTCAGAGGAAAGAGGGAGACAGCGAG
ACGTTTGACGCTGTGGTCCTGACGATGCCAGTCCCACAGATCCTGCAGCTTCAGGGAGACGTGGGACACTTCTTGTCT
GTCCATCAGAAGCAGCAGTTAGAACGTGTCGTCTACTCGTCTCGTTTCGCCCTCGCTCTCTTCTTCCCTCCAGACACC
GTCTTCAGTTTCTCTTGGGCGGCTCGATACATCACTGACAACTCCTGCATCTGCTACATCGCTGTGGATGCTCGCAAA
CGCAACGCAGACGCTCCTGGTTTCGGTCCATCCCTCGTCATCCACACCAGTGTGCCGTTTGGCCTGGAGCACCTGGAG
CGAGACAAGGAGGACGTCCAGCCAATCATCTTACAAGAGCTCCACAAGCTCCTCCCCGGTCTGCCTCAGCCAATCAGC
ATCAAGTGTCAGAAGTGGAGGTACTCCCAGGTGCTGACCTCGGTGCAGGACTGTCCGGGTCACATGACCGTCCTCGAC
CGGCCATTGCTCGTCTGTGGCGGTGATGCATTCAGCCACTCCAACTTCGATGGCTGCGTGGAGTCCGCACTGAGCGTG
CTCAGTGCGTTGAAGGCCTCGCTGTGACACTGCCGCCACGACAGGAAGTCCTCAAGGCAATGGACCAGGTTCAGAGCC
CAACAGGAGGATCTGACCGATACTGACAGGATCAATGATCAATGGAGTCATCAGTTAGGGTCAATACATGAAGTTTGA
TGAAGTGGTTCATTAAGATTTTGTCTTTAAATAAAGCCGTGTCTACATTAAAAAAAAAAAAAAAACCGACCTACCCTAT

>Sequ07057SNP2
ACTATAGGTCAATGTGTCCTTCCACACGTGTGTTTCTGTGATCTTCCAGCAGCAGTAACGGGGACTCAGCCGGTCTGT
GTGCTGTCAAACTCATTTATTCTGTCGTTACACGCTTAAATATTTCTAACATGCTAAAGTCGGTGGTCCGCGCGGTGG
GAGCCGCTGTCCGCATGTCCTCAGCCACCACGTCCACAGCCCGGGCTCTGCCGCTCAGCTGCCCGACTCTGCGCTCTC
CGTGTTCGGCTACAACGCGGCCCTTCACCCGGTCTCTGTGGATGCTGAACAGCAAAGGAGCCTCGTCAGGATACAGGC
CAAAACTGTTCAGTTCAAAGGTGTTGCAACCCACGGTGTCGTGTGGATGTGGAGGACTGCACACAGAAGGTGACAAAG
CATTTGGTGATTTCCTGTCTGATGAACTCAAAGAAGAGAAGAAGATCCAGAAAAACAAAACTCTTCCTAAGCTGTCTG
GAGGATGGGAGCTGGAGATGAATGGCACAGAGGCTAAAACTCACAAGAAGTGTTTCTGGAGAGAAAAATCTCTGTCTC
ATTCAACGTCAACAACAGCATTCCTCCTAACTTTGAGGAAGAGGCAGAACAAGGTCAGCAGAAGTCAGCAGAGGAAGA
GCCTGAAATTGTGTCAACTCCCAATTTTGTTGTTGAAGTCACAAAACAGGCTTCAAAACATTCCCTGGTGTTTGACTG
CCATTTCCCTGAAGACGAGATGAGTCATGGCGAAGGAGAAGAGGAGAGCGACATCTTTGCCATCCGTGAGGTCAGTTT
CCAGCCTGAGGGAGATACAGACTGGAAGGAGACCAGCTACACACTCAACACAGACTCTCTGGACTGGGCCCTGTATGA
CCACTTGATGGACTTCCTGGCCGACCGTGGGATCGACAACACCTTTGCTGATGAACTGATGGAGCTGAGCACCGCCAT
TGAGCATCAAGAGTACATTAAGTTCCTGGAAGACCTCCAAGGCTTTGTCAAATGTAACTAATGTTAGATAGAAGCTAA
ATTTCACTTTTCTTCAGTACACATCCGGAGATAGGATGATACAGTAAGACTAAAGCAAATGAAAGCACTGAATGATTG
ATTTGTTCAGCGCACGAAGGAGACTGATGGTAGTTTCTGTGACAGTGAGCCTTGTTGCCAGCAACCGTCACTAAGACT
TATCTGCAAACATCTTTCCTTTGGTGAAATGTGACTTTATTTTTCTGTAACTTAAACAACATAGTTTAAATTAAATAT
TTAAAGGCCATAAATAATGTAGGTCATGATAATCGTGCAGAAACCGTCATTGTAAGAAATGTCTCTCAATTTTAGAGT
TCCTGTCTTAACTGCACGGCTTCAGGAGGGTCCAGTCAGTATTTATGAATGTTCACTTTCCAATAAAGGACAAATTTG
TCAAGGTGCAAATTCAAAACAAAAATATCAAAAAGTAAACAGTTTACAAAATCAACCCTCACTTCAAAATGTATGTGA
CAGCTTCTGGCAATCCTGCTCCGAGTCTGACTTCTAGCTGGGCTTGTTGAGGGAGTTCACGTTGACCAACATTGATTC
AACTCTGCCAGACCATAAGACATGAAGTCTACAGTTGAATGATATATATCCATGTATGTTAAGAGAATGGAAGTTCTA
TGTCCATTAAAACAGTTTGATAG

>Sequ07071EST2
AGAGGCACAAGCAGACCGGGAACATGGAGCTCGCCGAGGCTCTGTCCCGTTGGGTGTTCAAGGAGGCCGGAGTCCTCA
GAGTGGGAGCTGTTACCCATCATCCTGTGGGAGAGAACACTCCCCCAGCAGCATACACCATCACTGACCTTGTGGAAT
ACAGCATTGTCATCGAGATGTTGTCCGAGGGCCGCTGGGTCCCCTTCGATGGAGATGATATTCAGCTGGAGTTTGTGA
GGATCGATCCCTTCGTCAGGACTTACCTCAAGAAAAATGGAGGTAAATACAGCGTCCAGTTCAAACTGCCTGATGTGT
ACGGAGTCTTCCAGTTCAAGGTGGACTACAACCGACTGGGGTACACACACCTCTACTCCTCCACTCAGGTATCGGTGC
GTCCCCTGCAGCACACTCAGTACGAGCGCTTCATCCCCTCAGCCTTCCCATACTACGCCAGCGTCTTCTCCATGATGT
CGGGGCTCTTTGTCTTCAGCATTGTCTTCCTGCACATGAAAGAGAAGGAGAAGTCAGACTAATGCAGACAAACAGGGA
AGGGGGAAAAGGAGATTATAAAATTTGAACTTGTGGTGTGAAGAGTAGGAATTGAATGTCTTGAATCTTCACGCAGGG
ACTTGGACAGCAGTCCTCCATTAAAAATACAGTTTCTGTTCGGTTATTGGGTCTCTCAACTAATCGGGGTCTCGAGGA
CAAAAGGGGGTGCTGACTGTAATGCAATACAGTGGTTGCTTTTTTTT

>Sequ07074SNP2
GGACACATTTCCGGTGACCTAACGTGAACAAACTGTTTCACCGTTGTCTTCAGCTCCTGGATTAACATTGTAGGTGTG
AAGTCAGTGCATTTGATATGGATGTAGTTACATCTGTATGTTTATTTCATAACGTTTAGTTAAGTTTGATCCATACGC
TTTAGTTTGAGGTTGGGGGCCGTAACCTTGGCGTGCATGCCTGTAACATCAGCGCTATTGTAGAAGAAGTGGTGGACC
CAAAAGAAAACATGACAGGTGCCTTTCAAGGTTTGACATCCTTATCAACCAAGATCCTGACAGAAGCTACACTTTTAC
CATGCCCATGGTTTGTGTCCGCACGCAGCAAGTTTTCCAAAGCAAGAATCCCAAAAGAGATTTTTGTTGAGAGATCAA
AAGAACATGAGAAGTATGGAGGGGATCCGGACCAACCTCATAAACTGCATATAGTGACTCGAGTCAAAAGTGTAATGC
GAAGACCATACTGGGAGAAAGACATGGTGAAACACCTGGGGCTTGAAAAGGCACATGTCCCTGTAATTCACAAAAATA
CACCAGCGGTCAACAGCCAGCTGAAATTTGTCAAGCACCTGGTGAGGATACAGCCGCTGAAGACTCCCTATGGACTCC
CTGCTGAGCAGGACATGGCCGACACCTACATTAACAGCAACGGAGAGCTGATTGTCCGTCGTCTACTCCAACCTGTAG
ACCCCAAGGCTATCGAATCTTAGCCTCACTCTTAATAAATTAATAATTCAGTTGTAAATAAATATCATGGAAATGACC
CTGTGCTCCATATCTACCAGTTCTGATTCCACTACTTGAAGGTAGCTGTATGCAATGTGTTTTGAGTTCTTGGACTAA
TGATTAAAATGAGAAATGTTGTCATCACACTCTATAACAAGTCCCTCAACAAACTAGAGGTCTGTCTCATGAAGCAGG
AATGAAAATTACTTGTTTTTCTGAATAATTTTGCTGAGTAAAAAAACAGTTTCAGGTTTAAATGTGCTCTGGATTTAC
TCAGTAGTTATCCAGGCAAGTTTGTTTATTTTGTCTATCAGTAAATGCATTTAAATACCAACCTATCAACCATACAGC
TGTTACAACTGTAAACTGTAATCTGTTTTCCATCACAATTCAGAAACAGGTGATGCCTTAGATTCCTCATTAAATACG
ACATATACGTACCCAAAATAAAGACCTTTTAGAAAACGAAAAAAAAAAAAAACCGAACCTACCCTAAT

>Sequ07106EST2
ATGGGGCGTTGACTTCTGTCACGTGACCCACATGTCCTTGGTTTGGCTAACTCTGACATGTGAACAGCAGAAACATGT
CTTTTCCTAAAAACCTGGTGGTAAAGCGTTAGATGTTTTGCAGAACCTACCACGGATAACTTTAGCGAACTTGCGGCC
TGAACCAGGAGCCAAACAGCCGGAAAGACGGCGGGGCAGAGGACAGCATGGTGGCAACAGAAGCGGTAGAGGCCACAA
AGGAGAGCGACAAAGAGGCACCCGGCCTCGGCTCGGGTTTGAGGGAGGTCAGACCCCGTTTTATCTGGCTATTCCTAA
ATATGGCTTCAATGAAGGACACAGTCGCCGTCCTCAGTACCAGCCTCTGTCTTTGAAACGACTGCAGTACCTGATCGA
TTTGGGACGAGTCGACACAACCCAGCCCATAGACCTGACCCAGCTGGTCAATGCCAGAGGAGTCACAATCCAGCCTCT
GAAGAGGGACTATGGAGTCCAGCTTGTTGATGAGGGTGCCGATGTTTTTGCTGCAAAAATCAACATTGAAGTTCAGAG
AGCTTCTGAAGGAGCCATAGCTGCTATTGAGAGGAACGGAGGCATCATCACGACCAGTTTCTATGACCCTATAAGTCT
CGGGATTCTCATCAAGCCGGTCCCGTTTTTCTTACGTGGGCAGCCGATTCCAAAGCGAATGTTACCAGGGCAGGATAT
GGTCCCGTACTACACAGATGCTGCAAGCCGTGGTTACTTGGCAGACCCGGTGAAAATCCAGCAGGCCCGGCTAGCCCT
GGCACAAAAGTACGGATACATTTTGCCAGACGTTTCAAAAGATGAACTGTATCACATGCTCTCCATGAGGAAGGATGT
TCGACAGATCTTCTTTGGCCTTGCTCCAGGCTGGGTCGTTAACATGCCGGAGAAGAAGATACTGAAACCCACTGATGA
GAAACTGCTTAAATATTACAGTTCATAGGTGTGAGGTGTAAAATAAATGTTATGTACATTATCTCATGGCAGAAATCA
CGGAAATACTGAGATTTGCATCTTGGAAAAACCTGTAAAAAAAAAAAAAAAAAAAAAAAGTCTGGGTGAGGTTCTGATT
TTATATATAAAAAAATGTGGAAAAGAGATTCTGTGTGTGAGAACGAAGCAATCGTCATTAAACTCTG

>Sequ07110EST2
CACCTGGCAGCTGTTTCTGGCTCACAGTATCCCTCTGGAAACTATTATCCCCATACTGCAACACCTCAAATACAAAGA
ACATCCAGAGGCCTTATCCTGTCTGCTGCTGCAGCTGCGCCGAGAGAAGCCGAGTGAGGAGATGGTGAAGATGGTTCT
GAGTCGACCCTGTCACCCTGAAGACCAGTTCACCACCAGCATCCTGAGACACTGGGCATCCAAATACGACGACACGCT
GGGAGAACACATCAAGGCCCAGCTGATCAAGAACAACAACCAGCCCCGTAAGAGACAGAGTCTTCGTAGTTCCAGCAG
TAAACTGGCTCAGCTGACCCTGGAACAGATCTTGGAGCACATGGACAACCTGAGACTGAGTCTGAGCAACACCAAGAA
CAACTTTTTCACTCAGACTCCGATCCTCCAGGCTCTGCAGCACGTTCAGGCCAGCTGTGACGAAGCACACAAGATGAG
GTTCAGTGACCTGTTCGCCCTGGCGGAGGAATACGAAGACTCTCAGTCCAAGCCTCCAAAGTCTCGTCGTAAAGCTCC
AGCCTCTTCGCCTCGCTCAAGGAAAGGAGCAGCCCCGCCCACCAACAACGAGGAGGAGAGTGCCTCCAGCAGCGCCTC
GGAGAAGAAGACTCGAAGCCCAAAGCTCCCAAAAAGGAGAGGAAAGGCTCGTCAGCCGTTGGCTCTGACAGCGACTGA
TGGACTGATCCTCTGACCAATCACATGCCAGCTGCCACAGAGACCGCCAGCCAACCAGCCGCTCTGACCACTGGAAGA
GGCCCGCCCCTCCCGATAAGACATGCACTGACCGCCGCGGGTGGGGCAGGGCTCTGAGACGCCACGGACTTTAAATCC
ATGTGGTGAACTGACTTTCACATGATGAGACTGTCGCTGTTTCCATGGAGACGAGGACTGACCTGATCAAAGGTCATA
GTGTGGCTTAACTCCCATGATGCACTGGGAGGAGGAAATCACAAACTCTGAGAATGAAACGACGACGTTTTTAATACA
GGGAACATGATGATGATGATGATAGGAATAATGATGAAGATGGGTGCGGTTGTTTGGGCTTTTTTTTTCCCCAGAAGC
GGGGGATTTTGAACTCTTATGTGGAGGATCCTGACTTCCTGCTGTTTTAGCAGCTCTTCCTGTTTGTCTCGCTGCAGT
AAAAGCTCCTCGTCTGTTCTGAACATCATGTTGGATGAATTCTTCATATAAAAACTGATGCTTGTTTCAGGTTGTCAT
GTCTGCTGCTGGTGGGGGGTGAGGGGCTAAGGTTTTGATGCCGCACATGTGGCGCTGCAGTCAAAGAAAGAACCACTC
CACTAGTTTTTTTTTTTTTTTGTTGTTTTTTTTTACCAG

>Sequ07114SNP2
AACAAAATGTAATTTATAATTTGTTTTTGTTTTTTTTTTTTGGGGTTACAGGAAAACAAAGACAACCTGTAAGAAAATA
TGTGTATAAACTGAGGGGGTTAAAACAACCAGGACAGCAAAAGAATAAAGCGAGCAAAGTTTGTACAGTACAAAATAC

TAAATCACTGCTGCCTTCATCTAATTGTCACTGAAGTTGTGTGTCTGTCCATTCTTATGTCCGTGGTGATGGCTCTGC
AGTCCATGTGAACTCCTGGCCTACGCCCCCACATCTGTCCGACAGGCTGAGTATCTGTAGGTGTACAGCTTGTTATCT
GCTCCTCCACTCAACATCAGCCCGCTTTCTGTCCAGTCCAAACAAAACACTTTGTCCTCGTGGGCCGCCACGTCATAC
AGAGGGGCCTTACAGCTTCTGGTGTCCCACAGTTTGACAAGGTTGTCAAGGGAACCGGAGACCAGCTGGTGCTCATGT
GAAGGCGCCCACTTGACGGCGGTGACCCAGCCAGTATGCGAGGTTAGAGACAGCAGCACCAGTGACCCGTCTTTGGTG
CGAGGGTCCCACAGTCTGATGTGTCTGTCGGTGCTGCCTGAGGCCAGGCGTCGACACAGAGGTGAGTAGGAGATGCAG
TTGAACACTTTACTGCCCGTCAGCGTTGTCTTCATTCCTCCAGTCTCAACGTCCCACACACGGATGGTGTGGTCCCAG
GATGCACTGCAGACTTCCTCACTGTCGCACCAGAGGACAGAGGACACCGCCTCATTGTGCCCGGACAACGTCATCAGG
GGAGTCCTGGTCAGGCCCAGCTGTTGAGTCTTCTGTTTCTTCCTCGGTCTGTCGGCAGTTTCTTCCACCTCGTCTGCC
TCATCTGTGGGGACTGCGGACCAGATTTTCAACATTTTGTCCCAGGAGCCACTGCAGAACTTGGAGCCGGTGGGGTCT
GTGGCAACAGTGTCGACGCTCCCCGTGTGTCCCCGACAGCAGTGTCTGGCTTTCACCTTGTTCCTCTCAGAGTTCCAC
TCCCACATCAGGACGGTTTGGTCCAGAGAGGCCGTCAGAAGCACCGACGTCAGACCGTCTCTTTTAACCCAGGCTACA
TCTTTGACTACATCTGTGTGTCCCGCCACCGTCATCACCGCCTTGCCCTCCGTGGACCAGATCCTGGCCGTCTTGTCA
TAAGACCCTGTCAGGATCCATTCAGAATCTGCATCTACGGCGCTGATCCAGTCATCATGCATCATACACTCCTCAGGC
TCTGGAGCTGTGATTCGCTCCACGTACTCGATCTCCACCACATCTTCCGTTGATATGCCTTCTGTATCCATGTGAAAG
GACAGCGACGATCGCAGGAACTGACCCCTGACCAGGAAGTCGAACTCCACTTTACTGTGGGACGCATCTTTAGCCAAC
AGCAGCTTGTTGATAACATTGCTGAGGTCCTCCACTTCAGAGCCAGCTGGGATGGAGAAGGGAACATCATCTACAACA
TACCTTTTGTCTCAGTGAAGAACCTCGCCTGTAACTGAGACATTGTTCTGGCTCGTGTGTTTCTCTATCAGACCGCCG
GCTGTAGTAAAGTAAGGAACGACTTCACCCTGCTCCTTGTCGTGCAGTCACGCCATGTGGTACC

>Sequ07141EST2
CTAAAACCGGAACCGTTGAGCAGGGGAAAGAAAATGGCGAATCTCGTGGAGGCGACAACCCAACAGCAGTTTGAAGAC
TTCTTAGCCAAAGCTGGAAAATGCCTGACCGTGGTACATTTCCAGGCGGCATGGGCTCCTCAGTGCGGCCACATGAAC
GAGGTAATGGCCGAGCTGGCGAAGGAACACATAAGCACCACGTTTGTCAAGATGGAGGCGGAAGCGGTGCCGGAGGTG
TCGGAGAAGTATGAAATCTCCTCTGTCCCCACTTTCCTTTTCTTCAAAGGGGGGGAGAAAGTGGACCGCCTGGACGGG
GCCCATGCCCCGGAGCTGACCAAGAAGGTGCAGCGCTTGGCAGTGACCGGAAGTCCGCGTGGAAATGCGGAAAGTAGC
AGCACAGACCTGAACCAGCGGCTAAAGAAGCTAATCAACGCGGCACCCTGCATGCTGTTCATGAAGGGGTCATCACAG
GAGCCCCGCTGCGGCTTCAGTCGGCAGATAGTGGCCCTGCTGAAGGAGCACAACATCCAGTTCAGCAGCTTCGACATC
CTGTCCGACGAGGAGGTCCGACAGGAGCTCAAGACCTACTCCAACTGGCCCACCTACCCTCAGCTGTATGTGAATGGA
GAGCTGGTGGGAGGACTGGACATAGTGAAGGAGCTGGCAGAGTCTGGAGAGCTAGAGAACACCTGCCCCAAGGCTGTC
ACCTTGGAGCACCGGCTGAAGACCGTCATCAACCAGGGCCCAGTCATGCTGTTCATGAAGGGCAACAAGGAGGCTGCA
AGATGCGGCTTCAGCAAGCAGATACTGGAGATTTTGAATGGCACCGGGGTGATTATGACACCTTTGATATTCTGCAGG
ATGAAGAGGTGCGTCAGGCTCTGAAGACTTATTCTAACTGGCCAACCTACCCCAGCTCTACGTGAAGGGAGAGCTCAT
CGGAGGGTTGGACATAGTCAAGGAGCTGAAGGAGAGCGGAGACCTGGTGTCAGTGCTGAAGGGGAGTCCTGAAGGGGC
AGTCGTAGGTGGATTTGCCATGTCATCAGAGACAGGAAACAAGAAGCTGTCACATATGCCTAGATCGGCCCTCTGTCT
CCGATGCAAAACTACAGGAGTGCCAGGAAGTGAGAGAAACCATTTGTGAAGAGAAATTAGAGTTAATTATCAAATAAA
CCTCTTCTAACCTAACTGTTGTAGCATTTGGATAGGTTTATGTTCTGTTTAAAGTTAACAATGAAACTGCAGTTAACA
ATGAGGACCGACGTGGTTTTCATTCTACATTTTCCATATAGTGTTACAAGAAGTTTTGCTTCGAGTAATCCTTGAAAA
TACACCTTCAGGAATGTGTAAAGTGAAAGTTAAGTGTTCCCTGTGCTTGGCCTAATATCATAAATCCCTGCAATTAGT
GATTGGTAGAAACACCTCCTCCTTGCTATGTGACACTGGGTGGGAATAGTTGATGTTAGTTCATATTATCTGCTGTCT
AGGTTACATTTTCCCTCCAGTATTCACAGCCTTGCACTTAACAACAGGTTTATATTTAGTGATAGCTGACTCTTCCTG
CCGTCCTGTCCAGCTGCTGTCCATGCATTCTTCACCTGAAAGACACTAAAACTGTCTTTTACCATATCCAAGAATCAC
TCTGGTTTAAATACTTTAAAACAAATTCTATGTTGCACTTATTGCTTTGTTTCTTCATCAGTAACAT

>Sequ07142EST2
TAACCGATGTCCGTCTGACAGTTGGTCTAGTCTCTCGTCGTTACCGGCCAGATCATCATGTCTTCAGATCCGTGCTCC
TTCTCCTCCTTCACCAGGTGCGTCACCAAACACCTGAACCTTTCCCTCCACGTGGACTTCGACAGACACGTCATCAGG
GGAAAGGTGGAGCTCACGGTGGAGGCTCTGGAGGACCGCTTCTCTGTTCTGACCTTGGACACCAGAGACCTGACGATC
GTATCAGTGTCAGCCAATGGACAGGCGGCGCACTTCGGTCTGGGCCCCAAACACAGCTTCAAGGGGACACAACTGGAC
ATCACGCTGCCTTTGACCTCTCCAGAGGGCAGCATGTGATTGTGGAGGTGACCTATGAGACGTCTCCATCTGCGTCGG
CCCTGCAGTGGCTCACACCTCAACAGACCGCCGGGAAGAAGCAGCCTTATCTGTTCAGCCAGTGTCAGGCTCATCACT
GCAGGAGTATGGTCCCCTGTCAGGACAGTCCGTCTGTCAAACACACCTACTACGCTCAGGTGTCTGTACCTAAGGATC
TGGTGGCTGTAATGAGTGCAGTGAGAGACGGACAGGAAGTCGATCCTCAGGACAACAACTGCATCATCTACAGATTCA
GACAGCCGGTGCCCATGCCTTCCTACCTGATAGCCATCGTGGTCGGAGCTCTGGAGAGCAGGGAGATCGGTCCAAGGT
CCAGAGTTTGGCTGAGAAAGAGTTTGTGGATAAAGCAGCGTTTGAGTTCTCTGAGACGGAGACCATGTTGAAGAGCGC
TGAGGATCTGGCTGGACCGTACGTCTGGGGACAGTATGACATCCTGGTTCTTCCTCCATCTTTCCCCTATGGAGGCAT
GGAGAACCCCTGTCTGACCTTCGCCACGCCGACACTGCTGGCAGGAGATAAATCTCTGTCTAATGTGATCGCCCATGA
GATCTCCCACAGCTGGACTGGTAATCTGGTGACCAACAAGACCTGGGAACAGGTCACACTGTCTACCTGGAGAGGATG
ATTGGGAGGAGTATGGAGAGTGAACAGTTCAGACAGTTTAAAGCCATGGGGGGCTGGAAGGACCTACAGGACTCGGTG
AACACCTTCGGGGCCAACAACCCTTTGACGAACCTTATCCCCAGTCTGCAGGACGTCGACCCCGACGACGCCTTCTCC
TCTGTCCCCTATGAGAAAGGCTTTGCTCTGCTGTATCACCTGGAGGAGCTGATGGGAGGCCCAGAGGTGTTCATGGGG
TTTGTCAAGTCCTACATCCAGATGTTCGCCTACAGCAGTGTCACTTCAGAGGAGTGGAAAAACTACCTGTTCACCTAC
TTCAAAGACAAGGTGGATGTCCTGAACAAGGTGGACTGGAACGCCTGGATGTTCACTCCAGGGATGCCTCCTGTCAAA
CCTCAGTATGACACCACGCTGGCAGACGCCTGCATCGCTCTGAGCCAGAGATGGATCAAGGCCAAAGACCAGGATCTG
AGCAGCTTTAAAGAGTGTGACGTGAAGGCTCTGTCATCCCACCAGCTAATCGAGTTCCTGTCCCTCCTGCTTCAGGAG
GATCCTCTTCCTCTGACTCATGTGAAGAAGATGCAGGATGTTTATGGTTTCAACACCTGTATGAACTCAGAGATCCAC
TTCAGGTGGCTGAGGTTGTGTGTTCGGTCCAAGTGGGAAGAAGCTGTTCCCATGGCACTGAAGATGGCGACTGAACAG
GGACGGATGAAGTTCACAAGACCACTGTTCAGAGAGGTCTTTAACTTTGAAAAGTACCGTGAGGAAGCGGTGCGAGTG
TTCCTGGCTCACCGGGCAGCAATGCACCCGGTCACCTCCGGACTGGTCGCCAAAGACCTGAAAGTGGACGCCAGCCAA
ACCACCAGTCTGTAAAACTAGTCAGAAACCAGTATTAATACACCGGACAACAGTATCAGCAGCCAATCAAAGCTCTTC

ATTCTTCACGCTCACCTGTCAGCTGTTTCCTCTCAGACTGTTTGTGATGATTTTTAGTTCTCTGGATGAACATGTTTC
ATGTCAGAAGAAATTATTTTAANAAAGAAATAAAATCTAGCCT

>Sequ07148EST2
CCACCCTGAGCTTGCCCAGCTTGTTAAAGAATTCTTTGGGGTAGTGGACAAAAACAGTGCTGCTTTGAAGCTGGCCAT
GGTTCCTCGCTCAGCCAAACTTAACTATGGAACCGACCCTCAGACTGGACAACTGCTCCGCTATCCACTGGTCAGTGT
GCATAATGTGTACATCGTTCCTGGGATCCCGTCTCTGATGGAAAGGGCCTTCACTGGGCTGGAGCATCTCTTTGCTAG
TTCAGGGACCACTTTTCACACTCGTGAGGTGTTTGTGGATGCAGATGAAACAGAGATCGCCCCTGTGCTGACCAAACT
GCAGGCTGGTTGGGGTAAGAAGGTGGCTCTAGGCTCCTACCCCGACTGGCTGAGTAACTATCACCGAGTCAGGCTGGT
CCTGGACTCAGACAGCCAGGAAGAGGCGGACAAATCCCGGGCGCAGCTGTTAGATGAACTGCCCAAGGGCAGCGTGGT
GCCCTTAGTCACTGACCCGGTGTCCATCGCCACTGCTGAGGTGTACACACTGGCCAACAACGGTACGCCGTTAGGTGA
GAAAGTTATGTCTGCTCTGAAAACGATAGAGGCTGCACTGGATCAGTATTCAACAGGGGAAGTCTGTGTCGGCTTCAA
CGGAGGCAAAGACTGCACGGCGCTGCTCCATCTCTACTATGCTGCACTGAAACGACGGTATCCAGACGGTAAGGACAA
GGTGAAAGCTCTGTATATTCGTATCGTCTCTCCATTCCCAGAAATGGAGAGATTCCTCCAGGACACAATTAAAAGGTA
CGACCTGGATCTCTTCTCTGTGGAAGGCAGTATTCGGCAGGCGCTGAGCGAGGTGCAGGAGCGGAGGCCGGAGCTGAG
AGCCGTCCTGATGGGGACCAGGAGGAGTGACCCCTACTCACACACACTCACATCCATGTGTCCCACCGACCCCGGCTG
GCCAGACTACATGAGAGTCAACCCGTTACTAGACTGGACGTATCATGACATCTGGTCGTTCTTGAGGACGCTATATGT
GCCCTACTGCATTCTCTACGATAAAGGGTACACTTCACTGGGCAGCATGGACAACACCTGTCGAAACACGTCCCTCCA
GATGGTGGATGGGAGAGGGGTGACACGGTACAAGCCAGCCTACCTCCTGGAGAATGAAGAAGAGGAGCGAAACTCCCG
TGCCTGATGTAGCCTCACTTCTCCCTGTCACCTGTTGCTGATGGAGCCAGATTTTCAGATCACCACTCACACTATTGT
CCTTGTGAAGAAAAGACTCAACGCATCCGACTGCAGAATGAGATTGTGAAGGAAGTCTGGTTGTGTGTGTGTGTGTG

>Sequ07149EST2
CTGGACACTTGGTTCTCATCTGGCATTTTCCCCTTCTCTATCTTCGGATGGCCAAATGAGACACAGGACCTGAACGTG
TTCTACCCTGGCACCTTGCTGGAAACGGGCCATGACATCCTGTTCTTCTGGGTCGCTCGTATGGTGATGATGGGCCTC
AAACTGACCGGCAAGCTGCCATTCAAAGAGGTCTATCTGCATGCGGTCGTGAGGGATGCCCACGGAAGGAAGATGAGC
AAATCTCTGGGCAACGTCATTGATCCTCTGGACGTCATTACAGGGATCTCCCTAGAGGGTCTTCATGCCCAGTTGATG
GACAGCAACTTGGATCCTTTGGAGGTGGAGAAGGCAAAGCAGGGCCAGAAGTCAGACTACCCAACTGGCATTCCAGAG
TGCGGCACAGATGCTCTCCGGTTTGCCCTGTGTGCCTACACTAGCCAAGGTAGGGATATCAACCTGGATGTCAACCGC
ATCCTCGGTTACCGTCACTTCTGCAACAAACTGTGGAACGCTGTGAAGTTTGCCATGAAGACTCTGGGAGACAACTTT
GTACCATCAGAGAAAGCCCAGCTGTGTGGAGAGGAGAGTGTGTCAGACAGGTGGATTCTGTCTAGACTGAGTGCTGCT
GTTGCTCTCTGCGATGCTGCCTTTAAGGCCTACGACTTCCCAGCCATCACCACCGCCATCTACAACTTCTGGCTATAC
GAGCTCTGTGATGTCTACCTGGAAAGTGTGAAACCTGTGTTCAGTAAAGCAGAGGAAGACAGTGCCAGCCAGAGACAG
GCCCTGGTGTGCAGACAGACCCTTTACACCTGTCTAGAGGTCGGTCTCCGCCTCCTGTCTCCCTTGATGCCCTTCGTC
ACTGAAGAGCTTTACCAGAGGTTACCACGAAGACGACCTCAGAGTGACCCCCCAGCATCAGTGTCACATCCTATCCCA
ACACAGAGGAGTTCTGCTGGCACAGTGAGGAGGTCGACCGTGACATGGAGTTCGTAATGACTGTGGTCAAGACAATCC
GGTCACTCAGGGCCGACTACAACCTGACCAAGACCCGAGCTGACTGCTACCTCGAGTGCATAGACTCTGCAACTGTGT
CCCTGGTGCAGAAGTACAGTCTGCAGATCCAGACCTTGTCGTATTCTCAGGCTATCATCCCTCTGACAGCCAACCAGC
CTGTCCCAGAAGGCTGCGCTGTGGCTATCGCCTCTGACAGATGCACTGTCAACCTTATGCTCAAGGGTCTCATTGATG
TGGAGAAGGAGGTAGCCAAGCTGATGACAAAGAAAGGTGACTTGGAGAAACAGATGGAGAAATTGAGAGAGAAAATGG
CAAAGAATGACTACAAAGAGAAGGTGCCAGTGAAGGTGCAGGAGCAGGATGCCGAGAAGCTACGGCAGAGCCAAACTG
AACTTGAGAAAGTGAAAGAAGCCGTGGACAACTTCAGGAAAATGATGTAACTTTCCCTTTTAAAATTATGTAAATCAA
GATTTATTTCCACTCTTTTGATGTTAGTGTTAATTTTCATATTCATTTATCAAATTGATCATAGTATGGAGGGAATTC
AATAAAGCTCTGACAATTG

>Sequ07153SNP2
AACACTTTGAAATGAGGGTGTGAATTATACTTTATTATATAAGAAAGAGCAAGTTTTCCTCGCTGGATTTTGATTCAC
ATTTAAATTCATGTGTCAGTTTCTCTTAACTGACTCTATGATTGCAAGAGAGGTCATCGTTTCATCAGGCTTATTCTT
TAATAAGAGACATTAACTGTTACTGTTATAGACTTTATTACCGTTTCCAGACGGTTGCTGCCTCTGCGAACGGTGCGT
GTAACGTTTATGAACTCGGTTGTTTTTTTGCAACAGTGCTGGAATCAGGAAAGTGTTGACAATCAGTGTGTGAGACTG
TCCTGATGAGACCCCAAACATCTGCCCTGCTGATGTGCCCTCGAGCCGCTGCATCACCATACTCGTAGAGAGAAATCA
GAGCAAGTGCAAAGGTAGATATTCAATAAATAAAATATGCACAGGGCATTACATCCTTAAAATTTTCCCCAGATTCAA
CAACCAAACCAAGTTCTGTCTGAGTTTAGCATGTTCTTAAATATCGGTGTTTTGAATTTAATGTGCGTTCCAAGGCTT
TACATATAGCAGTGTTTAATTTAACATTGTGTCATTTCATTTAACGCTTATCCGTCCCATTTTAATGTCGCCTGTCTG
TTCAGTCATCGGTCCATTAACGGATTACAGGGACCATAACTCACTAAAGAGCTGTCAGGTATTAGAGATAACGTCTCT
ACAAGTTGTATATACAGGAAGCTGATTTTAAATTAAGTGCCTGTCATGTTTTTGTATAGGATACAATAATGTGTTCAG
CGATGTGATTTAAAACTACACAAACCTATTTGTAATTCTCGAGTTCTTCCCTTTTCTGTTTGCTTGTACAATGCGGAA
GAAAACTTTGTATTATTTTGTCACAGAGTGATTTCAAGTTTGTTATTGAAGGTTCCCTATTAAATATGAACTTTTCAT
AATACGAAAAAAAAAAAAAAAAACCGACCTACCCTAATA

>Sequ07190EST2
GAGACGAGGGGAAACAGATGACTCTGCTGCAGCTTTCAGGTTGAGGTTGACTGAAAACATCCGAGGGCTGTTTCAGAA
CATTTAAACCGCCAGAAGGAGTCAGTTTGAGAGATGCAGTGATGCAGTTTACCTGTACTGAGTGAGTCCACGTTCCCA
GCGTTAACCAGTGGGCGAGCGGGGGGCGTGCAGCCTTGCCCAGAGTGGGCGTGGCCGGCGATGGACAGCCGATGCAGC
AGCAGCGCCGATTCACGGCTGACCGGCAGAGGGTTGGGCTCTATCAACACCACGCCCATCTGCGCGAGGGTGGAGTCG
TACGAAGCCGTGGAGAAGAAGTGCATCTCTGACGTGAGGAGGACCTTCTGCCTCTTCGTCACCTTTGACCTCTTGTTC
ATCACCTTGCTCTGGATCATAGAGCTCAATGTGAACAGCGGCATTCAGCAGCAGCTGGATAAAGAAGTCCTGCACTAC
GACTACCGCGCCTCTTTCTTCGACATCTTTCTGCTGGCTGTCTTCAGGTTTGCCGCTCTCATCTTGGCGTACGCCGTC
TGTAAGCTTCGTCACTGGTGGGCCATCGCGATCACCACTGCGGTCAGCTGTGCTTTCCTCATCGTTAAAGTCATTTTA
TCAAAGCTGCTGTCTCAGGGGGCCTTTGGGTACCTGCTGCCCATCATCTCCTTTGTGTTGGCGTGGATAGAAACATGG

CTGCTAGACTTCAAGGTTCTGCCTCAGGGAGGCCGAGGACGAAAACNGGTATCAGTCATTCATGGACGCCAACAAAGC
GAGCTCCACTCATGT

>Sequ07210EST2
CAAGACGGATGTGCTCAAGTNCCGGGTTGCTCTNAACCCCAAAGAACTACCAGACGTTGCAGCTCAAAGTCACTCCNG
AAAACACGGCCCCTGGTCCCAGGAGGAGCTTCAGGTTCTGGAGAAGTTCTTTGAGACACGGGTTGCTGGTCCTCCCTT
CAAATACAACACTCTGAATGCCTTCACAAAGCTGCTGGGGGCACCCACTAACATCCTTAGGGACTGTGTGCGCATCAT
GAAGCTGGAACTGTTCCCTGACCAGGCTGGACAGCTGAAGTGGAACGTCCAGTTCTGTCTCACCATCCCTCCCAGTGC
TCCTCCCATCGCACCACCTGGGACCATCGCTGTGGTGCTCAAGTCCAAGATGCTCTTCTTTTTGCAGCTGACCCAGCG
TATCCCAGTGCCCCAGGACCCAGTAAGCATTATTGTACCTATTGTGTACGACATGGCCACAGGCCTCACTCAGCAGGC
TGACATCCCCAGACAGCACAGCTCATCTGGGGCTGCGGCACTCATGGTCTCCAATATCCTTAAGAGGTTCAATGAGCT
GCACCCCGCAAGACAGGGTGAGTGTACGATATTTACATCTGTTCACGAGCTGATGGCCAACCTCACTCTACCCCCCGG
CACTCGTCAGTAGAAACAACAAATTCAGAACCAGTACTGGAACTCGACTGGAGTCCCTTTGAGACCGAGGATAGGTCC
ATCATTGAGCTAAGAAGAAACAAGGGAGTAACAGCTGTGGTCCCCAGACATCAAAATCCAGCATGTGTCCTTTCTGCT
TCAACCACTGGACTTTTGACCTGATGTATTATCACTGTTATGAAAGGGAGTACAATATGTACAGTCGTGGACCCACAG
TCTTTATTCTTGATAGAAGCTTACAAACAAGATTTACTGTTATGGTAGATCTCGGACAAGGGGGGCATTTGATGCAAA
TGGATATAAATGTTATTTTTTTTCCTCGAGGTGCACTTGTAAACAAGATTATCTATTGAAATTATGGTTGTTTACGAA
AATAATGTACATTGTTGTATATTTGAGTGTTGAAAAGAATCAGGTGAGTTAGAATTTTGCTAGAGCCTTGTCTTCTAG
TTTTTTTTTTTTTTACAATGCCATACTGATATTATTGTAGTATCAGGAGATATATTCCTTAGCACACTATTTAAATGT
CTTCTTGCCTTTACGCTGTCAAGACCTCTGAAGTTTTATTTCAAACTTTCACTTTGGATACTGTAAAACTGTGACAAA
CTTTATAAGCCAGTTGTATTTTACTAAGACGCATTTTGAGTCTTGATTGAAAGATTTGAGACATCTTGTCTGACTTTG
CTATTTAATAAACCAAGCCCACAGCTGGTTTGTGCTTTGAGGATCCCCAGAAAAAAAAAAAAAAAAACCG

>Sequ07264SNP2
GTTCCCTCTTTTTAATATTCACTTGCACATGATCCATTAAGGTGTGATCTTAATGTACAACAAAGGCTGGCCATGGTA
ACAGGCATTACTCTACCCTCTACTGGCAGGACATGAAATCCTCTTCAGCTTCAAATGAAATTCATCTTCTCTGGTGAA
GGATGAAATATCAGTGTGGTACAAAGTAATTTTACTAAAACAAAATACCAAAAAGGTATGAATCATAGATTTTTACAC
TCTTCTTCCACAAAAACCCCAGTGAGGGAATAAGCTCAAAAATACAGACTGCCTTCGCTTTTAAACCTCCAACTACCA
AGATTACAGACTTAAGCATATACTACACAACTTCAAATATACAAAAAGATAGTAAACAGCTACAAGCACGTTCAGTAT
TTACAGCAAAGTCTCTTCTCTGTTGAAATCAAAGGAACATGGGTCACATTCAGAACACCCTGTCTTGTTTTTCATTTG
TAACTTCCAAAAAGGTTCTGGTGGTGTAAAATAAAGTAGGAACACAAGCCTCTGCACCCCTCTCAGGGCACATCAGTA
CTTGTTGAACCAGATGTCTTGATGTTTATTAGTATCTGTCACAGTGGTTCAGCATCCATTCCCGTAAGGTCCGAGCCA
AGCTCTACCATCTCCCTCTGTGGAAAGGTGGGCACCCGAACCCTGGCCTCCCATTTCGGAAATGCCTCCCTTCTCTGT
CATCCATGCCCTCTGGTCCCATAGACGGATGTCGATGGAAAGGAGGACCNCGAGGAGGGAAGGGCATTGGCATTCTGA
ACCTGTCCGGTGGAAACATGGGTCCTCCGGGCATCATTTGCGGAGGCATGTGTGGCATGTTTGGAGGTGAGAGGAATG
GAGGTAGATGAGGATTTGGCATGCCAGGAGGAGGCTGCAGAGGAGGCATACCCGGTCTGGGACCCTGTATGACTGCTG
GGCCTGGCAGAGTCATCACTGGTGCTGCAGGAGAGCCCAGCTGGTTGTCACTACCTGCATCTGAGAGTGTACCTTTTT
CTCTATTAGAATCCTTGGTGGAGTCCTCTGGAGGTTTTAGACTCCCTGGTGCCAACTTTGAAGGGTCAAACTCAGGGG
GGATGAACGGGGCTCCTCCTGGAGGCAAGGCTGTAGGGGACAGCATAATGGGGCCAGGGAAAGCAGGTGGACTTCTGA
TGGGTGCTGTCTGTTGAACCGCTGGCACCTGAATGTGAGCAGGGATCGTTCCATCTGCTGGTACACTTTCCAATGCTC
CATTTAAGTGCCTGCTTATTGAGGTCCCTGGCATTATTCCATTCTGGATTTAGTGTCTCTGTATCCAGCATCG

>Sequ07286SNP2
TAGGGTAGGTCGGTTTTTTTTTTTTTGGTAGTTTGTGGAGTTTAATAAGCACTTGTGAAGGATTCAGTTAGAGAATGC
TGAATAAACAATATCCCATAAAAAATAAACATTACCCTCATTTTTACAGCTGACCCACAAATAGCCCCAAAACAAGAA
GTAGATCCACATGTACAGTCCCACCAAAAATCTTTTTTCTTCCCCCCCATTGTTTTCACAATGTATTACTGTACGAAC
ATATTCACTTTTCATTTATTTCTTTACGTGTATCAATTAGCGTACACCCACTACCACCAGTGGCTGAAGCGAAAACGC
CAACACACCTTAAATCACACATCTAAAGATTGTTGTAAGAGTTGAACCGATAGTTTGAGTACGATGGAGCATTTGTGG
CATCGGTGGAGGCCACAGGAAGCAGCAGGAGCCCTGTGCTGATGTCTCTCTTACACCGATCCATCGCCTGCTTGTATG
TTATCTTCTCTTTGGTGATGGGGTCTACCAGCTCTTTTGTGTAGGAAGCCTCGTCTTGCAGATGACTGTGTGTTTCAC
TGTCAATTAGTTTGGCGGCGAGAGCTTCTTCGACAGTGAGGCGACCTGCTTTAGCGGGATTCACCAACCCGCCGGTCA
GGTACTGCGCCTCCATGTACCTTCTGGCATTTTCTCTGGGGATATATCCTTTCTGCGCTGCCAGACCTACTGCGAGAC
GGTCTTTGGTCACAGGATCCTCGACTCCAGTGAAGGCTTTCTGGGCATTCAGGAGTTTATACATGTGAGCTGTATCAA
TGAGACCATATTCAGCTGCCTTATGGACAGAAAATTTGTCCTTTTTGGCGATATCAACAATTCCACCGGAGGCTGCCT
GTGCCTCAAGCAACCTAAAAGCTGTATCGGTATCAATGAGTTTGCGGGTGACAGCGCTTCTGACGGACATGCGGCTTT
CAGTGGTGTTGTCGTAAATACCAGAGATTGGGAAATGCTCATCGCCTGAAGCATTGAGGCTGTTTGAACTGCCGCTGA
GGTGAGTGTTGAGGTTGGTGTAGGAGGACCTCAGAGAGGTTGACATGGAGTTCAAGGGAGAGGTCGGGGTGGGTTTGG
TGGGTGATCTTGGGGTTGTTAAT

>Sequ07309EST2
TTGCGTCAATTAATTTATTCATTTCCTCCCCATCATTAAAAGAAATGCGGTTTACCAAAGCTACTACATACATACAAA
TGGCCATACAACAGCAGTTAACCATCTTGTCGTCAATAAATCCCTGAACCAAGTTTTGTTTTTTTCAGTTTCCTCTTG
TTGTGGGGTTCTTCTACTGTGGAAAGAAGTGCAGGCGGGCAGGACACCACAGCTTAGGAACAAAACCAAACTAAGCAA
CATTTACACGAGGGTCATCTATATTTTATACAACAAATGTTTCGTTTCTTTTTTTTTTTTTAAATGTAAAAGCCACAAA
ACTAACTTGAGCTAAATCTGCTTGTAACTAATCCAGAACTAGCACACAGCTTGTGTCAGGAGGGACAAAAATTATTTT
TTTTTTTTTGCATAGTTTCTCTTAAGGCACATTGGCTCTTGATAAACACGTAAAGTGAGTCGTCTCTGGTCCTGGAAG
AACCACCTTCATTATTTCATCAGACAGATTATAACCAGCGTTGAAAGGCAGAGCAGGTCTCACTCATTTCATTTGATG
TTAACTGGATCGACACTAGCAGCACACATCCATTCTGTTGGTTTGATAAGTGACACTAACATCCTGGCGTTAAAACTA
GAAAATGTGCATATTCAGACATTCAGTTGTCATGCTGTAACCCTCCACGTGTATCAAATGTCTACACACAACAAATTG
TGCAACAGGTTGGGGTTAAGCAAGTGAAAAGGCAATGTGAATCAGAATCAAAAGAAGACTTTGCATAAAAGAGGCGTC

GTGGCAAGTGACGGTTTTCCGTAACAAGGAAGAGGACTTGGATGCACAAACGTCCACTCATCAGTTGTAAGTTTTAAA
ATAATTTTCTTTAAATTATCTTTTTACGTGTATTTAAATTCAAAATGAAATGATTTTTTTTCTACTTCACATATTTCG
ACACCATGGCAGCAGAGAAAGTCCAGCGAGTGTTCAAGTTGTGTTAATGGTGAAGTCAAGTTCATTGTCTCTTTCAGA
TAGAAGGAAAGTAGAGAGGGGTGTTTGGGGCAATAGACAAGTCTGACCCACCTCTCTAACCCAACTGGTCCTCATATT
GCTTACGGAAGCAGTCTCCAGTAGGGAAGAAGTCCCAGCATCTCTCCTGGTACATCTTCCACACATATGACTCCTGAC
CCGTGTGTTCAGTCTCGTCCTTGTTAATCAGGTACATCAAGAAGAACAGGTAGTTGGCTAGGTTGTGCTCTTGTAAGG
TGTGGGTTTCAAACCCGTGAGGCGTCCGATCAAAGTAGTCGTTGCCAATGCCACAGATGAAGCATTTGGTCTCCATGT
CCTCCTTCACCTGCTCCTGCTGGTCTCTCAGCTCACCAAAGGCATCAATGATCAAACCCTGAATGATGGCCAGCAGGA
TGACGATGACAAAGAAGAAGAAGGTGATGTCAAACAGGATGCGGTAGAGCTCATAAGGGTCTCCAGCTGGGTCCTCCA
GCTCATCTCCAATACCTCCTCCAGCTCTCACACCGACATACATGTGGAACAAATAGCACGTCATCATGTCGTCACACT
TCAT

>Sequ07328SNP2
ATATAAACTTCAGTCTTTGTTTCTCTTCTGTGAAAGTATGAGCTAATGAGTTAAAGTTGTTTTCCTTTGACCAGTGAT
TGTTTTTTATTTATGAAGAGTAAATTACAAACAGGCCTGTATGTATCAGCACTCACTCAGGCTCTGAGTCTGCACTGT
GGTGTCACAACAGAAATACAGAAAGAAAATAAGATCAAACTTTAAGGCTTAGTCCAAACATATTAAAACTTGTTCTCT
CTCCTTTCAGAGTAAAACTAATGGTCGATAGAACAAAACAGCTGCTGATGAACAGTTAGGTCAACCAGTTTACTCGTG
GTCACAGTGGAAAAATTGGCCTAAATTATCTGTTTACAGCTTCATCCTGAGTAGGAGCAGAGTGTTGCCTTTGGTACA
GAAAAGAGTAAGACAACGTTGGAGAGATTGAGGTAAAGAAAAGGTAGTGAAATAGAAGAAGAAACGGAATCATAGAGT
TCACTGAGGCTCGGCAGGTTCAGCTCTAATCCAGACTCATGTCTTCGTCGTCCTCTTTCTTCTCTTTGGAGTCTCCTT
TGGTGCCCTGCATGGCTCTGGCAAACGCCTCCACATCTCCTCTGTTGGCGGCGTCCACAGCCTCCGCCGGCAGACCAA
ACTGACTCATGAGAGGGCCGAGCTGCCCGGAGGCCAAGGCACTGCTGAACATACTCATCGACTGCTGGAACTGAGGTG
AATTGAGTGTGTTCTGGATCTCTTCGGCACTCTGTGGTAAACTCTCTCCGCTGGGGAGGAAGGGGAGGAGCCTCTGCT
GGACCTCAGCATTAGTCAGGATGGGAGCCATCATCTCTGGGGTGCAAACACTCGCCAGGTCCACTGCTGATCCCTGAG
CAGCCGTTGCTGGGACGTTCATGGTGGCGAGGATGCTCTGAAGGTCACTCAGCTGGATGGGCTGGTGGGGGGCAGGGC
TTCCAACAGAGGCTGGGGCCAGAGGAGTCTGAGCTGAGGCTGGTGTGGAGGGAGCAACACCAGTAGGTGACACGGCTG
AGGCAGCAGGAGTCACAGGGGTGGTGGGAGCCTGAGAGGAACTCAGTCTGGGGGCGCCGCCCGATGAAGGAGTGGCTG
CTGCTGATTGGCTACGAGAGCTGGATGAGGAGCTACTTGTGGCTGGTCCTCCACTGCCCAGCAAGTTGGCAAGTCCTG
GCCCTGCTAGAGCTCCCAGGCCCCAGTCCACCCAGTCCCGTTGGTCCAATCAGCTGCATCAGCTGGTTGTGGCTCATG
TTTCCCAGGAGGTTTTGCAGGCCCCCTTCTCCTCCGAGCGCAGAGAGCTCATGGCCGCTGCCGCTTCCGCTGCCTGGT
GCTCCGGGGATCGGTGGGTTGTTC

>Sequ07340EST2
GATCGGGGGTGAGGTGCAGATATTTGACCTGGGCCGCAGGTCCCTGAGGCCAGTCGCCACCATAGAGCAGGGGGGCTG
CGGGACAAGCTGCTACTTGTTTGGCCTTCAACTGTCAGAGCACCCATCTGTTGGCGGTGGGAAACACAGACGGGACGG
TTGGTGTTTGGCAGCTGAGTGCAAATCTGACCGAGCAAAGCCCCAGAGAGAGCAGCCAGCTGGAGCAGATAGCCAATC
AGGTGGCAGAATGAGACACATGACCTGTTTCATCCTCTGTTTATATCAACTGTGTTTTGTAAAAAACAAATGTAAAAA
ATGTTTTTGTAATTTTAAAAAAGACTCAGTGTGGAAGTGAAAAAAAAGGAGTGAGCAGAGGTTTCCAGTTCTAACCAG
TAGCGTTTATTAAAGCACACTGCAGTTGTTCAGGTGTTGGTACAACATTATTGTTACTATTAGGTTCTTAAATTCATC
GAAAGCACAAGCTAAAACAGCTAAAACAATCACATTCAAATGCAGACAGATCCACTATGCTATCGTGAGAAGCACAAA
GTTAAGGTAGAGCGAGATAAATAGAACAGACAGTTGTTATTAGTGAGTTTTCTGGTGCCACAGTCATGCAGTTTCAGC
CGTGTGTTCGTGTTGAAGTGTGTGAGGGAAAACGCGTCCCGATTGGTCACTGGGGGGCGGGGGATCAGTTGACGAAGA
GCGAGCGGGTGAATTCGACAAAGTCGAAGGCTGACGGCAGCTCGCGGCCCTTGCTGTCGAGGTAGGGCTTCATGTGTG
AGAGACAGTAGTCCGCCTGCTCCTTGGTCAGGTTCTGGTAGAGCTCTTCTTTAGTCACGTAAGGTTTGTTTTCGGTGC
TCAGAGCTCGGAACGCGCTCTCTATCTCCTCACTGGACTTGACGTTCTCTGTCTCACGGCTGATCATGAACGCCATGT
ACTCCTGCAGCGACACGTTGCCATCCCTGTTGGGATCCACCGTGTCGAGTATGGCCTCGAACTCAGGATCAGGTTCTC
CCTCCTCCACCATGGGCAGGTCGTAGCCTAACGAGCGCAGACACGACTTGAACTCCTGGTGGTTCAGACGACCAGACT
TCTCCTTGTCGAAGTGCTTGAACATCATGCTGAACTCCTTCAGAGCCTCCTCCGTCACTCCAGTGGTGTTCCTGGCCT
GTATCTGCTGCTCCAGGTTATGCTGCATCCTCATTCCCAGTTGGTCCAGTTGGTCCCACTGCTGGGCCAGGCCCACTG
TGCTGTGCTCTGTGTACTTGTTGTCTAAGATCAATGCCTCCTCCATAGCTGCTCCCAGGTCTTCAATCTTCTTCAGCT
GGCTGCGCATGGCCCGGATCTCCTGGTGCTTACGCTTAGTGGCCTCCAGCTGGGACTCCAGGGTACCGGACTCCTCCA
CCATACAGGACCCATCCAGAAGATACGTCCTGGTCTCCTGCAGCCACTGGTGGAAGGCGTTGGCGTGCTGCGCAAACT
CCTGACGCAGTTTGTCGTTTTCCTCCTGTCTCCTCTGCTCCTTCTGCAGCTCCAGCTCTCGCTCCTTGATGATCTTCT
GCAGGTTCCTCCAGGTCTCCTCCAGCGCCTCCATGGTGAACCAGGTGTAGGGGTTGGAAACCACCTGGTAGCTCTTGA
TCTGCTGGTCCAGCTCGGCCAGCTGGTTGAAGTCGGCCTGAGCCGAGCTCAGCGAGGAGCGGAAGGCCTCGTGGGCCT
CTCTCAGTGCCCGGATCTCCTCCAGAGAGTTGCACCTGACCGGGTCTGTCAGGTCCTCCTCTGCGTTCTCGAACCAGC
TGTTGAAGGCTGAAGCTTTCTGGCGAATGTCAGGAACAAGTCTTCAACCTTTCTGAAGTGCTCCTGAGCCTCCAGAAG
CTTCTTCTTGCGAGCGGCTGAGTTAGACAGCAGCTGGTTCCAGCGCTTCATCAAGGCAGCGTGGCGAGCTTCGA

>Sequ07341SNP2
AGGAGTGAAAGTACTCGAACTCCTCCGCTTGGACTCATTCTATGTTGGCTTTTATTTAAAATTACTAATTCCTTTTTT
TTTTTTTCCTTCTCCCAAACATGTGATGGGACTGCTGTGTTGCCTGAGTGTGAAGTGCAGCTCAGGTCAGACTGTCGT
TGGTTGCAGACACGAGTGAAACTCGGACAGTGGAAATTTGAAATGAGTCATGTGAGCAGCTGACACTTTGGCAGGACT
CCTTTGGAAATTAAAGGGTCAGTTCACCCAAATGACAAGCCATATTTTCTCACTTAATACAAGTGGTGCAGATAGTTT
GTTTTTGATTTCTGCTTCCACCCTGACACTACAGATAAATGTCATTGAAAATGTGCCCTTTCATTTTTTAAAATCTAA
TTTGGGATAATTTCTTCAGTAGACAATGAAAACTTTTCTCTTGCTATAATATTTAAATGCCATTACTAAATCAGAAGA
GATTAACTGGGGGAGAAATAGCTTGATAAATAAAGCTCTGGATCTGCATTAAAGGCAGGAGAGAGGGGGCTTATTATC
TCTGAACTGACTCTTTAATGTTGCTTTACATGATACGAAAGAGGTTGTAAATCATTGGGAGCATAATGTGACTATCAG
TGTCATTCATTTCCTGTGGCAAATCTTGGTGTGTTTTGCTGTTTTCTGCCCCTCAGGTGAGAAACATGAGAACCTGTG
GTATGTGACGCAACGCCGAACACTTGGAATATTGAACCTGCACATGTAAAACTGTGCCTGCTTTACAGATGTCCTCCC

ACCATCTGCAAACCACAGCTTCCCCAGCACATTCACCGTTCAGTCTCCGCTCTGCCAGAAGAGACGTCTCAAGCGTGA
ACTGTGTTGTACAGAAGTTGCTTCTGACTTAAAGAGTTAAAATGGTACCAGTTCTGTGAATAAAGGTATTT

>Sequ07345EST2
GACGCACTATATACAGACACTATACGAAGTATGCTGCCAGGGCAGCCATTTTGTCTTAGGTTTTTTAGGATTGCCAAG
CCGTCCCCTGCCTCGTCCCCGTCCTCCTCGTCTTGCACCCCCACCCCGATGCATGAAGTGCCTCATGGTGGCATGTTT
AAGCTCCACCAGATCCTGGAAAACCGGATCACAGAAAACACAGCCGGTGTGTTGGGTCTCGCTGGCAGGAAGTGGAGT
GCGTGTCTTGTTGAAGTCGACTGCGACCAGAGAGGCGTCGCAGGCATCGCAGGTCTCCTGAGCAACCTGCACTCTGTG
TGCATGTTCAAAGAAATGTACCACCACGTTGCATTTATTACAGGCCATTCTCCATTTTGGTCCAGAGGTGGGATCCAG
CACTAAGACTCCACCATCACACTCCACACACTGTCCAATGCCCAAAGAGTTGAGAGAGTGCTGACAGGACGGATGAGT
ACATTCATTGCATCCCATACCTTTTTTCATGTCCCTAAAAGGCGGGTTGCTAAAGCAGTAAGGGCACAGGGGGGTAGC
TCTTGCCCCGGGCCCCAGAGGTCCACAGCACAAGCTCAAACTCATCCAGTGGACAGCGCAGCTCCTTGTACAGCTTGA
TGGCTCCATTCTGTGGAAGACTGTAGGTCTCGTCACAGTGGGAGCAATGGAGCCTGCTGGGCTTGGCCTGAATGTACT
TCATGAAGCGGTGGCACTTGCCACAGCGAGACAGGGGCTTTCCTGAGGCAGCAATGGGAGAAAAGGAGACCTCCATCA
ACTCATCCATGCTGGTGATGGAATCCACAAAGAAGTGAAACTTCCTCTTGAAGATATCCAGTGTATGCCCC

>Sequ07358SNP2
GCAGTGTCGGCAAAAGTATGTCAGTGTCAACCTTGCTTTTGACCAGTACATGCTTGTGCACCATGTATTGTCTTGCAG
TGCAACCTCAAAGGTTTTCTCTAAGGAAACATTTCAGTGTGATATGTTTAAGCAGGTGGACACTGAACTGACTGATTT
ATCATGAATCTGTGTCATAATGGCACTTGAAGTATTCTTCATATCACCAGAGAGTTGAATTAAGTTTTATACGTGTAA
AATGTAAAATATACTGGGCTGTGTCATTAATGGTTTCTTTTACTGTGCAAGTGATGGAGTGGTAGTCTCTTCAGCATT
ACACTTATTCTATTAGACCAACTGGTGAAACTTGGAGAGAACATTAGTGTTGGTTATTTATTTGTTGTGTATTTTTTC
TGCGTGGTTGGTCCCTGTATGAAACAACATGGTGCCTTTTCAGATGTTGTGCCTGACTGTTCTGACCTATTTCTGTGC
AAAAAGACAAGAATATTCCCTGAAGATGAACATGAATCCGAACAATCCTGATTTTAATTTTTAAAAGAAACTGCCCGA
GGTGCTCTAATGTTATTTTTCCACTTAGTTTTTGTCCTTGTGCCAATGTGAGTTATAGTACCTCTTTCTCTTATTTA
ATGCTGTCTAACCACTGTTGCCTGTTTTGTCACATTAAATTTGTTAAATAACTATTTAACTGCACGTCAGTAAAAAGA
AGACTGTGTTGCTAACATGGGCTCAACTTGATGGATGTGAACTGTTACACAATAAGCAGATGTTCTGTGCCCTGTGTT
CTAATGATGTGTATATTCTCATTTCTTTCTTTGCTTTTAAATATTCACCCATATGTTCACTTTTTTTAAGTTTCTTGC
TTGCATTACCTATGCACATCTCAAAATAAAATCTTTATTATGCATTTTGTGCATTTTTTAACATTAATACATGATCTT
GTATTGCACATACCTGGCAAATACAAATCACAGCTGCTTCATTGTTTAGTAGCAGGAATTCAGTCCAGTGTCAAAATG
CCCAGGCCAGAATATTTTTTCTTAACTGTTTTCACTGGTCACAGAGGAGGCTGCTCTCAGGGCTCTTGGCGTCCACGT
CTCCCCAGTAAGGCAGAATGAAGGGCAGGTTGGCCACTCGGCTCTCCAGCAGCCCCCACAGGTGCTGGGCCACAAACT
GGTTTCCCTTCTCTGAGAGATGTAATCCGTCAGACAAGTAGACTGTGTAGTCCTGTCCATCTTTCTGCATGAGTGTCC
AGAGGTCCAGGACATCTGTGCCACACTGACTGGCAGCCTGAACACAAGCCTGGGCATACTGACCCGCTACAGAGTTGT
GGCGATTGAGAGAAAATCCTTTCAAAATGCACTGCTTCTCCCAGGCTGGTTCATGGATAGGTGGGGGGTGATGAAGAT
AACTTTGTCTGCTGACACTCCAGCTGAAGCCAGAAAGCGGGTGATCTCCTTCAGGTTCTCTGAATACTCCTGCAGAGG
GATGTGCTGCTGGGGGTTTTTATCTTCCAGTGAAGAGTCGTTGGCTCCAAAGAAGACAGTGACAGCAGCTATGTTGTT
GTCTGCTGAGCTCTGGCTGTTGATGAGGCGAGGAAGAACAATCTTACCCCATTTGGAGTTGTAGCCAGACAGCCCTCT
GTTTACAACATCACACTTTCTTGCTAATTTGTCCGCAATTTCTGCACCCCATCCATTGGGTTGAAATGAAAACTGTGT
GATGGAGTCGCCAAATAAAATCACTTTAGGCCAAGTTACGGTTTTGAACTTAGACATCATGAGGCTATAAGTAGGAAC
TTACGAACACACCCTTCCTGTT

>Sequ07368EST2
CACAGGCTTTTATTTTGCAAAAAGATAAACACAATTCAAAAGACAACAGTGATTCAAATTGTCTGTGTGCGTAAATGA
GTTCATGGCTGTAGCTCGTAGCCCAGTGGGTCGAGTCCTCGGTCCAACAGAGAGAGCGACGACTCCCTCAACTTCTGC
AGGAACCTCCTCAACTCCTCTTTGGAGAAAACCTGGTAGAGCCGGTGTTGGTCAGATGAGATGATGTCAGCGAGTCGC
AGCGCCTCCTGGTGTCTGGAGGAGTTCTGCAGCACACTGAGCAGCAAGAAGCTGAGGCGAGGCAGACACAGAGAACGC
AACGCTGCCATCTGGTGGCTGCGCTCTGAATCAGGCTCTGAATCCTGTCTGTTGTCGACCATCCATCCTCCATCGACA
AACAGCAGCACGTTGTAGATTCTCTCCTTCACATCTTCAGTCAGAACGTCCAGACGGCACGACCAGGCGGACAGAGAA
GTCTGGTACTCCTTTTCTCTCATCTCATTGGCTACCCTCTCTGTGAATTTAGCTTCAGGGGCAGGTGCTGGCTTCTGA
GGGGCGGAGCTACTATGGCTGAACCAGTCAGTGAAAGCCTCATGAGCTTCCAGGTACGCTCTGATACACAGGTGTTCT
CTGATGGCGTTCTCGTCCTCAGCAGGTAAGGTGGTCTGACCGATCCCCGCCCACTGGCAGCAAATCTCCTTCATTGAA
TCCTCTGGAACTTTAGAGAACACCGCCTTAGCTGCGTCGTGTTTCTGCAAGGCCAGAAACTTCCTCATGATGGCGTTG
GACTGTTTCAGGGCCTCGGCTCGATGGGCGGGGTCAAATAGCAGCCAGTCGATAACATCAATCTTCCTTTGGTCCTCC
TTTGTAGTTGCCGTCTCCAGCGTCTGGTTGTGGTGTGTAAACTCAGTGTCATCTCTCTCCCTCACAGTCTCCACCACC
AGCTTAGTTATCGCAGCAACATCCAGACCGGCTTCACTGGCGAGCTGCAGGCAGCGGGGGCGAGCTCTGCCTGGGGTG
ACGGTCTCCAAGAAAGCAGCGTACTGAGCTGTGGCGAGCTCGGCCGGGAGCCCCG

>Sequ07377EST2
ATTACTTTTATTTCGTTACAGCATACCTTTCTCCTTTTAGCAAATTTAAATGCAAAACAATGCAAAAATACATTTGCC
CACCTGAATTTATGACAGGTGCATCAGAGGACAACTTGAAAAAAAAGCGACAAAATCCTGCACACTGTTATGTGCTGC
CGGTATATTTCATATATCATTTCAACAGAATCTTTTCACTTTTATTTAAGTCCAATCTTTAGCTGCAAATACTGTGTG
TATACATATTCTTTTGCTTTCTGCCCTGTGTAAAAAGTCTGTGGTGTGGGTTCAGTTACGGAATCTAAAAAACACCCT
TGGTGCTGAGTTCTGGGACTTCAGTAGGATGGATAACTAACACAGCTCTTAAAGTCAGATCAGTAGTCTCTGTGCCAA
CCTGCTGCCAATGGCCGAGTACTTTCACTGTTTTAATTCAGCCACTGTGACAGCGAACAGAATACAAGAGTTTACATG
TGAATTACAAATGAGGATACATGTTTTCAGTTCTGGCTATTTACACAATGACCAAACTCTTGCAAAAGACACAAACAA
TACCTGCTCAATAGCTGTGGTAGACTGGAGATGATTGGCCCATATCCAGGAGCGTTGTCATACAGTTCAAACAGTGGT
GCAGCCACCAGTTTATAGTTCTTGGGGACAGCAAACAGCGCTTTTTCCTGCAGCTGGACCAGGAACAGCTTCTTATGC
TCCTTAGGCTTGGTGATGTGAGCTGGAATATAGGGGTACTGTGGAGGCTCAAAGTTGGGGCGCCACCAGTTGCCGATA
CAGTCGTCAATCACCCAGTCCTGCTTCACCCCGTCCTGCCGCCCGAGGATCTCTGTCATCAGGCGTTTCAGACCCTCC

ACCTCATCTTCTCCGGGACTCAGCTCTCCACCGGGCAGTTTAAAGAAAGTGGTACCCAGCTGCAGGAGTAACACGTGA
GGCAGCCTGTGTTCATGGACAATGAGGACACCTTCCACAGTCCTCCGCATTCCCATTTTATCAAACTCCTCACGCATC
CGCTGGAACCGGGCTGCCACTGAACTGTCCTTCTCGTACAAAGGCTCCTTGGTGCCCGAATGTGTAGTTG

>Sequ07395EST2
ACTATAGGGTAGGTCGGTTTTTTTTTTTTTTTTAGGTTTGTCTAACATTTTTATTTCATGAATATTAAATGTACTTACA
GTTTGTATAAAAAAATATAGCAAAAGCTAAAGGCAAACAGCTGGCAAGAGGACACCAATACTTAATCAGTCTGGGGGT
GTAATGACAATGTCTCCCTATAACAGAGAGAAAGAAAGAGCTAAGTTTCAACAGGGAAGACAACACCTGGTCAGTGAC
TGGGGAAAAGAAAAGACATTTATGCTTTTCCATGCATGCATAAATAGGAATGCAGGAAGCATAAATATGTACAGATGG
GACTAAAATAGAAACCGTGTAATTTGGCGCTGTCAAGTCTCTTTCCAGTCAGATAACATATCAAATTGCATACACCAT
TTGCCTTCTGTTAATAAAAACTATAGAGGTATTGGTAAGCTCTTAAAGAACATCAGTTATGTTTTTATAGAAAGTGTA
AATTAGTTGCTACATGAAAGTCAAAGGGGCTTTGGGAGGATGTGTGTCCATTTGAAGCACTGAGGGATCAGTAACTGT
ATGTCTATGCGAGAGTTTGAATGGCAAACTTTCACAGTGTCACTAAAGGCATTGTTTGTTGAAAAATAATAATATACG
TCTGTAAATGAGTGTAAGTTACACCACTCAATGTCATTGTAAATGAAGGCATCCCTTCTTAGTGCTCAACAGAAGCTG
TAACGTCTCTTTTAGAGGGACATACAGGATGTTATGTTGTGTCACACACTTTGTGCGAGCTTCAGCCTCTCCCACATC
AGTTTTGTCGTAAACCCCGGCAGCCTCTGTCCTCTAAGCATTCTGCATCTCTTTGCCGATCTTGTAGCTGAGGATCTT
GTTCCAGTCGATCTTGGTGCGGGTGACCGGGAGCTGTCTACGTAAGGCTTTGAAGGTGGTGTCTGACATGGTCTGATA
GTTCTCACTGATGGCCACCTGGTATTCATTCTCTGCATCCTCGATAATTTTCACCAACTCCTTTGCAGTCTGGCTCTC
ATTAGACACAGTCAGTGCTTCCTGTACATCTTTGTGGCTGACCAGTTGCACATTTCCATCCTCATAATAATGTACCTG
TATTTTCAAGACTCCAACCACCTGTGCTGTCGATTGCGAGACACTGAACTTCCATTCAGACCGGCAGCGACCGTTCCA
GAAGTTTTTAGGCTCAAATTGATGACCCTCGATACAAGCGATAATGGTCTGCTGGCCATCAATCGTTTTCCCATAAAC
GGTGCAGACTCCAGTGGGGTAGTGCTCCTTGACATAGGCCCTCAGCGCAGAATCACAGGCGTCTCTCCATGACCTGAG
GGCTGACTCGCCCTCATGGGGCTGGGGGTCGCTGGCCTCTTTCCTCAGGTGGTCAAACTTGAAGGAGATTTTATTGTG
TGGGTCAAAGAAGCGGCCATTACCCAGATCTCCATGCTCTGTTATCAGGACCTGATCTTCATATCCATCAATTTTGGC
AAGAGTGAACTGATCCATGTTGTACTGGGCAAAGGCGTGAGCCGCGCCCTCTCTGAGAAGATTATCATTATTAAGCAG
CAGACGCACATCATTGAAGACTTCATTGAACTCCCCTGGAGGAGCGTGCATCACAAAATTAGCTGCAATACGGATCTT
CTCCTCATCGCTGAGCTCTTCGAAATCTGCCATCTTGGTTCAATGAAGTGTTAACAA

>Sequ07400EST2, Sequ07400SNP2
CAGTCTCGGTTTTTTTTTTTTTTTCTAGTTCTTTACATTTATTAAGAATGTAAAAAGAATAGCAGGGTATATATCCTTC
CAAAACGTAATGAAAAATCCAAACAGAGGCAGAATCTAGGCATGGGTGAGGGAAGAACTTGCTTCTCAGAACAGGAGT
GCTGATCTAGAATCAGGTTGGCAATCCACAAAAACAAGGATGTGTGAATAATGAAAATATGAAGGGAGTAATTGTGGA
AAGCAGGAGAAGAGCATACATTTGAAATAAATGTAGACAGTCAACTGATATCAAAAGGGAAAATCAGCTCCCCAGTAA
AAGCCTCACATAAGAGGCCACCTGAACAACCTCTGGATCAAAACTCCTGTTTTAAGAAACTTGATGTAAAAGGGCCAA
ACCAACAGTGTGAATGTGGAAAGAACAGACCAAAGAAGAAAGCAGATGGAAAGATCATTTTTACTCCTCAGTCTTTCC
TCAAAGGTACAGTCTGCTCATTAATGCCCTCCTTAGTGATGACGCAGACCCGAAGGGCATCCCCGGTGTAGACATCTC
TCTCTGCAGCTGAGATGAAGACATCTTTGACCAGCTGAACCGCCTTGTCCTGAGTCAGAGGGACATGCTGCACACCCT
CCATGTTCTTAAAACCAATCTGGTTGTCAAGCAGTGGCTGCAGCATGGCACTTGCCGATCCTCCAGCCTTGTAGGTGT
CTCTCTGGTAAGAGCCCACTGGGTCAAAACTGTACACTGCTCCCTTGCCCTCTTCATCCAGTCCTCCAATGATGTTGT
AGACGTAGTAGGGGAAGAACCTCCTGCTGTATAGGATGGTAGACAACATGGCTGCGATGGCTCCGCTGGTCATTGTCT
TGTTGTTTGAATGTTTGTACATCTTTAATCTGGCATCAATGATTTTGGTCAGAGTCAGGCAGTCGCCATGGAAACCGC
TGCATCCAATGACGGTTGTGTCTGTCAGCTTGTAGCATTTTGGTGAGTCCCGGCTGTGGATTGAGTAGCCTTCACTCA
GCCTGGTGTCTGAGGCTACGATGGCGAAGTCTTCCCCGGCAACCGCTAGTACAGTTCCTCCGTTGAAAGCGTACGGTG
AGAACCGGTGCTCTACCGGACCGGTATAATGATATTCTTTCATCTTCCCAGAGTCTCCAAAAGTCTGCGCAGAGAGCA
TCTTTGTTACGGATAGACAACAGTGTTCAGCTTTCTGCCAGGTAAAGTCGCTGTATCACTGCAATATGGCAGCTTCTT
CTTTTACTTCTCC

>Sequ07451EST2
GTCCCAGATCCGTGAGAAGATTGTTCAGCAAGAAGTGGTAAAGATGGAAGAAGACCCTCTCCTCAGGTCAGAGTGTGA
CACCTTCTCACAAAACATCAGCACTGAGCAGAAACACAAAGAGAGCCTGAAAACAGAGCTCTTCCAACTGCAAAGACA
GAAGGCTGACCTGGATCTGCAACTAGAGGAACTAGAGCGTGAGCGCAAGGCAAGGCGTGATGCAGAATTGGAGATCCA
AAGGCTTCGAATCAGACTTAATGAACTGGAGATCCGTGACAAAGAGAACCGTGAGAAGGTGACAGTCAAACAGAAGGT
AGTGCTGCAGCAGGATCCCCAGCAGGAGAAGGAACACTCCATCCTTAGACTACAGCTCGATGAAGAGAGGCACAAACG
CACCCTGCTTGAGAAGGAGTTGAATGCCCTGATCCAGCAGCAACTCACCCTGGAGAAGATGGATGTGAAGGAAAGAGT
TGTCCGCACTGAGAAAGTTCAGGTTGAAAGGGACCCAGAAGCTGAGATTGAGATTGAGAACCTAAGGAGGACTCTGGA
AGAGGAGAAAAGGAGAAGACGAGAACTTGACCAGGAGCTGTCCATCCTCACTTCCAGGCTCTCAGATATGGAGTTCTC
TAACACCAAGTCTTCTAAAGAACTGGACTACATCCGTGATGAAAGTAGCCGTCTGCAGCAAGAAAACCAAAGGATGCA
GAACGAGATCCGCAAACTGCGTTCTGAGATCGATATCACCAGCAAAGAGACTCGGATTATTACTGAGTCAGCACCAAG
GGAAGATGGAAAGAACCTGGAGTTGAGGCTTGATTCATTACAGAGGGAACTCGCAGAACTCAGAGGCATAACTATCCA
AAAAGATGAGGAAATCGATAAGCTTCAAAAGAACCTGTCGGCAGTGAGAATAAAGAGGGAGCAGAGGGAGAGCCATCT
CCGTCGATCTATCGTCGTCATCGATCCAGATACAGGCAAGGAGATGCGACCAGAGGAGGCGTACAAGCTAGGGCTGAT
CGACTGGAAGATGTTCGTGAACCTGCAGAGCCAGGAGTGTGACTGGGAGGAGATCACAGTGAAGGGTCCCAAAGGAGA
ATCCTCGGTTCTTCACGACAGAAAATCAGGGAAAAAGTTCTCCATTGATGATGCGTTGCGTCTTGGGCACATCACAAA
TCGCCAGCTTCAGCAGTACATAAACAAAGAAATCTCCATCCAGGAGTTTGGTGTAATGGTGTCGGGCAAAAAATAAGT
AAATGTAAGAAAATGCATTTTTGGTTTGACTGTAAAAGCTTTAATCTGTTTCATCTGTGTTGTTTTAAACTCTCCTTG
CTTTTTATTGTACTTCTGCTCTGACTTCAATTCTTTGGTGATTTGGCATTATTTTGTACTTTATCCACGATTCGTAAA
TGCACTATGTATTTATTTGCTTATTCATATTTAGTTTTGTGGGACTGGCATGGATATCTGGATTAATATTACTATCGT
TACAGCCAACTACATAACACTTCAGCATAACACTGTGGTAGGAAAAGTTTGCTCAGCTGAGCCTTAATTTCTTAGACT
GGGTACGAGGGTGGAGAATATATCTTTGATTTGATATTGTCATTGAAATTCACAAAGGTACTAAATGTTTCATTTATT
TGTGGGGTTTTCCCAGGTTTATCTGATGTGTGCTCTCCAAGCTTCAATAAAAGTCAAATCAAAACCAAAAAAAAAAAA

AAAAAAAACCGAACCTACCCTAACTAAGT

>Sequ07494SNP2
TGCTGCTTTGGTTTAGGAGCTGATCAACCAAATAGAGAAGGAAATAGGAAGCTAATGAGTTTCATTGCTTTTGTGTAT
GTACTGTATGTTCCTGTGGTGCTTTGTCGAGGACCCGGGGGATTTTAAATGAGAGTTGTGTATGTTTTGCAATAGAAT
ATGCGAGAAGAAAATGAAGGCTGGGGTTTATCTTATTTAAAACAATCACTGTATGTTGTAGTATGTTTGCTCAGTCTG
CAGATTTATATTCATTCAGCAGTAGCAGCAGCAGGAGGGCGGTTGGGATGACGTGTTTGCCTGTAAGCTCCTGTAGCC
TTCATGTTTTCATGTATGTTTCAGTGTAGAAAACTCTTCCACACTCTTCCACTTCATTATATTCAGGGCCTGTTGTGC
TTTAGAGCCAAATATGAGTTCAACTTTTGTACGCATGGGGGCACTTTGTGTGTATGGGTCTATATTTTTTGTACCATT
TTGAAGTGCACATCTTTTCAGCATTCAAAATATTCAGTTGCAGGATTTTGTTGTTATAGAAATAATCAACCCCTAAATT
CATTTATTAGCCGGTCAGATTACCTCGTGATTGTGAGATGGAGGAAAATATAGAACGATTTATAACTCCATGTCTTAC
ACTGAGAAGAGCACGCTTCTGATGTGCTGTTTAAATGTTTCTACATTGTTCTTTTAGCCATGAATACATGGGTTTTAA
TTAGAAAGAACAAAGCGGTTTTTAGCTGAAATTGTTTTCCTTTTGAGTTTGCAAATGAACACAGCCCACTGGTTATTC
ATTGTTTTCATGCAAAACAGAAAAATCCTCAAGCACTCAACTAAACACTGCAGTTCTAATAAATGTCCCATGATCTCA
TATTCTCATCCAATTGGCATGATATCAATGGTACGTGCAATAAAACTAGTATTCGTATAGATCAGTGTAATGTATTGT
AGAAGCCATTTTGTTTTGTCAGTTTTAGTGCGTTTAATGAAAATCAAATAACATTGCTGAATTTGAAGAAATGCATTT
GTGTGGCCAAATATGGTCAAAACTACAAAGCTCAGATTAAATAAAAAACTTTTACAACAAAAAAAAAAAA

>Sequ07802SNP2
GTATCGTGGCGTTTGCTGATGTGTTTAGATTTCTCTTGGACTTCCCACAGTTAAGCTGTTACCTGATAACTGTCATTT
CTGCTTTACTTGATCTCATTTTTTGCCCGTCAAATTACTAAAACCACCGGGCAGTATTTCCCTTAGCGGGTCAAGATG
AGGCGCCTCGGTTTGGGTGAAGGAGGCTCTGGGAATTATGTGGCAAGTGGATACAGTATGCATGAAGAGGAAAATGAA
CATCTACAGGAGGGACTGAGAGCCAAAGTCAACGCTTTGAAAAGTCTGTCTATTGACATTGGAACGGAAGTGAAATAC
CAGAATAAAATGCTGGATGATATGGACACAGACTTTGACTCAACAGGCGGCCTGCTTGGTGCCACCATTGGCAGAGTG
AAGCAGCTATCCAGAGGCAGTCAAACCAAACTTTTGTGCTACATGCTCCTCTTCTGCTTTTTCGTCTTCTTCATCCTG
TACTGGTTCATCAAGCTGAGGTGATAAGAGGATCCTCCTGGATTTGGCCCTTTTAACTTCACATCTTCCTCCCCAGTC
TGTGCCATGCTGATGTATCTCGAAACTAGACCAACATAACGATGATGAGGAAAGGCAGGATTTTTGTGTCTCAGCTTT
ACTGGTTCCTGTGAGCACTTCATACAATGACCCCAAAATCAAAATTGAGTCATGACAGGAATCTTGCTGATGCTATTT
GATGGAGTCCTAACACATTACAACTTGTGCAAAAGGTCCGAGCTGACTAATGATCACGTGGAGGTATCATATATACCA
TAAGTAGACAGTGCCAGTATTTGTATGTACATGATATCAGCCCAATTCACCTGAATTGAGAAGATGCATCATAACATT
AAACATAACTGGAGAACATACTACTGTGTCAAGAATCTTAAAGCAGTAGTGTGCATATTGAATGGTAAACAGCAACAA
ATCTGTGGATTCCTTTATCAAACCACTCCCGTGTCAAAAGTGATTAAGTTATAGAATGCTAGCTGAAACAAATAATTG
TATTTGCTTTTAAGAATGAATGGAAATATTACAGCATTTCTCATTCATACAAATGATTAATAGCAGTGTACGTCATTA
CCGTTTAAATTTTGTTGTAAAAATGTTTTTTAATGTAAATAAAGACATTTTAAGCTACTAG

>Sequ07879SNP2
TAGCTGTGCTGCAACTAATATGGAAGTAAACCAAAATATCTCCTGCCTAACGGATGACATAGCTGACATGTCTTGGGG
GCTTGGAGACCAAGCAGCAGCTGTTGAAGCCAAATGCAAACAGAACATGCAGGATTTGATTTCACTTTGGGTATATTA
ACAGCCTAATATCAACAGTTGGTTTTCTGGGTTCCTCACATGCATCGTGAAACTGACCAGTATAATTTACAAGAATGT
TGATTCTCTTCAACCTTGAATTTTGTTGGCATAAGCCAAATGACAAAACCTGTAGAAACATGAATTTTTTCCTAATGA
AAACCTTTATTAAAGCCCTGACATTGGAGGCAGTTATGGAAACGCTGTTGAAATCGCCTGAATCCTGCACATAGCTTT
TGCATGCTCAAATGTCTTAGTGATAGAACATTCATTGCTTTGACTCTCATCTACACAGCACACCTGTAGCTGATATTT
GGCAGAGGTGTATGATGTTTTGTTAGTATAGATTCAAGGTGTGTGTCTGAGGAACCCAGGCTGCAGCAGTAACTACCA
GCATGACGTGGACTGTTTGTAAGTTTACGAGTCTTGAGCCTTTTTTTTTCCCTCATCTGGTCGGATGTGATCAGTGGT
TCAAGGAAGGCATCAATGCAGAGTGACATCAGTCCAGAACCTGTCATCGTCTCCTCCATCTATAAGTTTTTCTTTATT
TTCCCATTATATACCCCCTTCCCACATGTCTACCAGCCCTCTAGCCTACCCTTATGTTTTTGGTTTTACATGTAAAGT
TATGGACTTTGGGGGAAAAAAAAAAACTTATTTACAGTGTAAAGATGGGTTGATATCCACTGACAAAATGTGAACGCT
TTGATTTATAAAAGCAAACTGATCAGTGGAAGGATTGAATGAATAAATGTAAAATAAAAAACGATCATTCAGGAAAGA
TCAATATGTTTGCACATAAAGGGACTGGCAGGCAAGCAGTTTTTGTTTCCATTGTCCTTTTTTCTTTTGGACGGTGTA
TGTTGCTAAAGCTGTGTTTTGTACAGTTTCAGAAAAGCAAACTGGTTATATACATATGTTTTTTTCTTTTTTTACAGC
AGGGGATGCAGACTTGATTTCAAAACATTTTAGGGTTGTCAATTTGTTACACTGGTTTTTAGTCTGCACGTGTTCATG
TCCAATCCAATTTTTATTCAATAATAAATGAGTTATG

>Sequ07939EST2
GGGCGAGCGGGGAGCTTCTACCAGAATAACCATGAAAGCTTTTCCTTCGACAGAGATCCTCAGCTGTACTTCGATGAT
ACCTGTGTGGTCCCTGAAAGATTAGAGGGCAAAGTGAAACAGGAGCCCTCTGTGTATCGTGACGGACCTCCTTACCAG
CGCCGCGGCTCCCTGCAGCTCTGGCAGTTCCTGGTCACTTTACTGGACGACCCAGCCAACGGCCACTTCATAGCCTGG
ACCGGTCGTGGCATGGAGTTCAAACTCATCGAGCCCGAGGAGGTGGCTCGTCGCTGGGGAATCCAGAAGAACAGGCCG
GCGATGAACTACGACAAGCTGAGCCGCTCGCTGCGTTACTACTACGAGAAGGGCATCATGCAGAAGGTGGCCGGTGAG
AGGTACGTGTACAAGTTCGTGTGCGACCCCGAGGCTCTCTTCTCCATGGCGTTCCCCGACAACCAGAGGCCCAACCTG
AAGGCCGACCCGGACAGTCTGCCGGGGCTGGACGAAGACACCGTGCCCCTCACCCATTACGACGAAAGCGCTCCCTAC
CTGCTGGACGGCGGGGAGCAGTGCGTGGCGGGCTTGCCGTTTCCAGACGGATACGGCTACTAAACGAGCGCGCAGGGG
CGTTTCCTGACGCCAACAAACACACATGTACACGCAAAAAAAAAAAAAAACAAAAAAAAAAAAAAAAACTAAGAGACTAG
CGAACGACGACACAGGGAGTAG

>Sequ07985EST2
ATCTGGCACAACAACGAGAAGACCTTCCTGATCTGGGTGAACGAGGAGGACCACACCAGAGTCATCTCCATGGAGAAG
GGAGGCAACATGAAGAGGGTGTTTGAGAGGTTCTGCAGAGGACTCAAGCAGGTGGAGCATCTGATCCAGGAGAGAGGC
TGGGAGTTCATGTGGAACGAGCGTCTGGGCTACGTCCTCACATGTCCCTCCAACTTGGGCACCGGCCTCAGGGCGGGT
GTGCACGTACGCCTGCCAAAGCTCAGCAAGGACTCACGATTCTCCAAGATCCTGGACAACCTGCGGCTGCAGAAGAGA

GGCACAGGAGGAGTGGACACCGCCGCCACCGGAGACACCTTCGACATTTCCAACAACGACCGTCTGGGCAAGTCTGAG
GTGGAGCTGGTGCAGCTGCTGGTCGACGGAGTCAACTACCTGATTGAGTGTGAGAAGAGGCTGGAGAAGGGCCAGGAC
ATCAAAGTCCCGGCTCCCATCACTCAGTTCAGGAAGTAAAGAGCTGGACTCATGATGCCGTCAGCGTTGGCAGGAGGT
CAGCTCTCTGCTCGCTCGCTCCCTCCCAAACCGAGCCCGCGGAGAAAACCTCAGAACGACGTCGGTCCGCCGCCACAG
AGGAGGCTGCTTCTTTACACCTCCCTCACTAAGATTTCAAATCTTTAGTGTAGCCTAAACTTAGACATGGTCTGCTGT
TAGGGGAAAATTCTACCTCAAGTTAAGGTCTTCGCTGTAACCCTGACTCCAATAAAGCCGACAGTAT

>Sequ08061SNP2
GCTGTCTGCAGGAGCCCAAAGACACAATGTTGTTCCACGTCTTCCTCCTGTTGGCGGCGACTAACGCCGCCTTCAGCT
TTGACGACAGGATCGTCGGGGGTTACGAGTGTAGACCTCACTCCGTCCCCTGGCAGGTGTCGCTCAACAACGGCTGGC
ACTTCTGCGGAGGAACCCTGATCAACAGCCGCTGGGTGCTCAGTGCTGCTCACTGCTATAAGGATGAGAACATTGAGC
TGCGTTTGGGTGAGCACCACATTCGCTACAAGGACGGCCCCGAGCAGTTCATCGCCGCTGCTGCCGTCATCCGCCACC
CCGAGTACGACCGTTACACCATCAACAACGACATCATGATGATCAAACTGGCCGAGCCGGCGGAGCTTAATGAGTCCG
TGCAGCCGGTGTCCCTGCCCAGCAGATGTGCCCCTGCAGGGACCCAGTGTCTGGTGTCAGGGTGGGGGCCACTCAGAG
TCCCTTCGGATGTCTGAAGTGTCTTCACTGCCTGGACCTGCCCGTCCTGTCCCAGCAGGACTGTGAGCGCTCCTACCC
CGACAGGATCACCGCCGCCATGTTCTGTGCCGGCTTCCTGGAGGGAGGAAAGGACTCCTGCCAGGGAGACTCTGGGGG
TCCTCTGGTGTGTAACGGCGAGCTGCAGGGGGTGGTGTCCTGGGGATGGGGCTGTGCTGAGGTGAATCGTCCCGGAGT
CTACGCCAAGGTCTGCCACTTCTCTGACTGGATCCAGTCCACCATGTCCTCTCACTGACGTCCTCCTGCAGAGGACAG
ATCCACTCTGTCTGCAGCTGCTGTCCATTTCATGTCATCACACTGACTCTTTATAAGAGGAACATATTTATGACTTTG
TTAAGCAGCTGTTGACTGTTTGACTGTTTGTGGATCATGTTTTTAACAATGACAGTAAAATATCACCATGAACAACCT
AACAAAAAAAAAAAAA

>Sequ08137EST2
TCAACAACCTGAAGGTCCAGGAGCGTCAGAACAGAGTCTGCACGACCGTGGCCATCGCCATCGTCGCCGAGACCTGTT
CGCCGTTCACGGTGCTGCCGGCGCTCATGAACGAATACCGCGTGCCCGAGCTCAACGTGCAGAACGGCGTGCTCAAGT
CCCTCTCCTTCCTGTTCGAGTAATCGGAGAGATGGGCAAAGACTACATCTACGCTGTCACGCCGCTGCTGGAGGACGC
GCTCATGGACAGAGACCTGGTCCACAGACAGACCGCCAGCGCCGTCGTCCAGCACATGTCTCTGGGCGTCTACGGTTT
CGGCTGCGAAGACTCGCTCAACCACCTGCTGAACTACGTGTGGCCCAATGTATTCGAGACGTCGCCTCACGTGATCCA
GGCGGTCATGGGCGCCCTGGAGGGGCTGAGGGTGGCCATCGGACCCTGCCGCATGTTGCAGTACTGCTTACAGGGTTT
GTTCCATCCGGCCAGGAAGGTGAGAGACGTCTACTGGAAGATCTACAACTCCATCTACATCGGCTCACAAGACGCCCT
CATCGCCCATTACCCACAAGTCAACAACGACGAGAAAAACATTTACGTCCGCTACGAGCTCGAGTACGTCTTGTAAAT
ACAAATTTGTGTGGTCTTTGTTTTTCTCCTCCCTCGACCATCACCCTCCTCCGGCATCCATCTCTCTCTCTCTCTCTC
TCTCTTTCTCGGTTTTAGGACTGAGTCAGTTTTATTTAGCGGCTGTATGTCGGTCTGAACAGTCCGTGTCCTGAATGC
CACTTATTGATCCTTGATTGAAAGTGAGCGTGATATTAGGGGGAAATAAAAGAAGAACATTGTGGCGTCTGTATGTAT
TTTTTTAATTTTCTTTGTTTGAGTTCCCTGTGAGGAATGGTTTAAGGTCGGATGAGCCTTGGTAGAGGAATGCAGCGC
AGTCATGACAATGTTTTTAAAGATTTGGAATTTTTTTTTGTTTTTTTGTTCTTTGTGTATTGATAGGCTCCTGTATCA
GCTGTACACACCCTCTCCATGTTTCACACCTCCTCTGTTCTTATTAGATTGTTGCTTTATTTAACCAAATAAAATGTT
TGTGTATAAAAATCAACTGCGTGTCAAGTTTTGTTTAGTCAAAGTAATTTAAGGGTCGGACAACCAGCTGAAGCCCTC
GCCTGCTGCACTGCAACGTTCA

>Sequ08208EST2
GTCATCCAGAAGCTGTGGAAAGACTCTGGTGTGCAGGCCGGCTTTGATAGAGCTGCTGAGTACCAACTGAACGACTCT
GCTGGCTACTACCTCAATGAAATGGACAGAATCTGCAAGCCTGATTACCTCCCCACTGAGCAGGACGTGCTGCGATCT
CGAGTCAAAACAACTGGTATCATTGAAGAACAGTTCTCCTGCAAAGAGCTGCACTTCAGGATGTTTGACGTGGGTGGC
CAGAGGTCAGAGAGAAAGAAGTGGATTCACTGTTTCGAAGGTGTGACCTGTATCATCTTCATTGCTGCTTTGAGCGCC
TACGACATGGTGCTGGTGGAGGATGATGAAGTGAATCGAATGCACGAGAGTCTGCACTTGTTCAACAGTATCTGCAAC
CACCGCTACTTCGCCACCACCTCCATCGTACTCTTCCTCAACAAAAAAGACGTGTTCGTTGAGAAGATCAAGAAAGCT
CATCTCAGCATGTGCTTCCCTGAATACGATGGACCCAACACCTACGAGGATGCTGGAAACTACATCAAAATGCAGTTT
TTGGACCTGAACTTGCGCCGAGACGTCAAAGAAATCTACTCTCACATGACCTGTGCCACAGACACAGAAAACGTCAAG
TTTGTGTTCGATGCCGTAACCGACATCATCATCAAAGAAAACCTGAAAGATTGTGGTCTCTTCTAAGAGCCACGCTCA
GGGACCCAATGACGGGTGCTGTTGTCGCCCCGTCAGACTCCACAAATCCCTCCTCAATTGAAGCTGAGGACCCAAGAT
GAGAAACTGTCCCCGAAAATATCAATTCTCAAACAGGCGAACGCAAAGAAATAAAAAATCAATTTGTTGTGTTCAAAA
AAAGTTGTAGCTTTGACCAAAGACTTCAGGTCAACTCAGACCAGAAGACAGCTCAGATTAAGTCAGAAACTTCTGTGC
AGATTTCAGATGAGTTGAACTTTAGTCAAGCTAACTTTTCTCACGAGTCTCCTGAACTTGTCTTCGGAGGGGTACGCA
ATCTGTAGTTAACACATCAAAAGCTTCTGTCAGCATAAAGTGATTTCTTCTCACATGTATTAATGTGAATAGATGTCA
GGCTAACGCATTCGCTCTATGCTCCAGTAACTGGTCAGTATATACCGCCAAAAAAAATAAAATAAATAAAAATAAAGC
AAACAAGATGGCCTCACCACCTGCAAACGCACTTCGACCTCTGTTAGAGATTTAAAAAAACTTGTCACTAGAAACACC
ACAGAGCAGTTTGAAACACAACTCAGGCAGGTTACTCACTGAATTTTTAAGTAAGAAAATTTAAACTTTTTTTTTTTA

>Sequ08248EST2
CCAGAGACGGGCAGCACATGAATCCAAGATGATCGAGAAACACGGCGGGTACAAGTTCACGGCGCCAGTGGTGCCATC
TACTTTCAACTTTTGGAGGCACTGCTCCAGGAATGAATTGAGTCATCTGTTCTACTTTTTATTACTCTGTGACTGATT
GTAGTGAAGTGAAAAAAAGCTTGTGTTGTTCCCTTAAGTAGTGGTTCCAGAACTGGTTCTTCTCAGTCATTCTTCAAT
CTGTCAAATGGGAAATAGCACCAGAAGATGTGGGAAAGCAACCAAAATGCAACCAATGGCTGGGGAAAACAAACCAAG
AACTTGAGCACGATGCAAGAGAAGAAGCTCTGAACAACGTTGTCTTCTGGAGCCCAGTGTGGAGGGGATACCATGACG
GCAACTCTTTTGTTTTTTTCCCACTTAAACAAAATTGGGGGATTAACAGTTAGAAATTGATAATGGGACTAAATATTT
CATTATCACTGTTGTGGCCAAATGGCCGTAATAAGATTATACCTCTGGTAGTGGTGATATCTACGTTATTTTCTGCAT
AAACACTCATGTTTATGTACATACAAGCCAAGGTTATTGATTTTTCAAGTAGCCTTGAAAAACAACCAGAGGCATTTG
TAAGGTGTCAACAGCTGTATTCATATGTAGCATATTGGATACTTCATAGCACTATGTGATGCCTGATCTCTGTAAATT
AATGACTAGCACTTTCATATTGTTCAGGTCTCCTAGCCTTCTGTATCAGACTGCAAATTTTTTAAATTGAAGACTATT

TAAAATGAAAACAGCTGTAACTTTGTTACTTAGCAAGCGACTTCTGTAATGACTGGTTGTCACGCATCTGGTTACAGA
GTGGATCAGTGGGCAACTCGGAAACATTGATTTCAGGAAAAAGAGAATGCTCTTGGTGTTCAGTATGGCTACAGGACA
TGTGTTTGTGAGCACTGAAAGTACAATTGGATACATCATTGAAGCATGTACAATTGGACTTATCGTGGAGGTCTCAAG
CTTTTGGTTGGTGTATGGATGAACCATCTATATATACAACAACATAATTAAAAAAAAAAAG

>Sequ08425SNP2
TCATATTGTGATAGTTTGGACTCTGATCTGTCCTGACTCTGACTCGTCTCGGCCTGGTAGCTGTCTGGACTCGGACTC
GTCTGAGTGTTGTGGGGTTTTTAATTCGGGCTTGTAAAGAGACAGACTTGTTTTGGTCTCATACCCGTCTGGACTCTT
GAGTCTTGTGGGCGTTTTGACCTGGATTTGTTCTGACTCGGACTCGTCTGGATCTTGTGAATGTTGACGCTGAGACTT
GTAACGAGTCTCTCACGTGTGTCCTGACTCAGTCCGGTCGTGGTGTAACGGATGTTTGGACTTGTTTCTGTCTCGGCC
ACGAGTCTGACCTGTTACAGGCTGCTGTCAGTCAGATGTAAAGTGGTATCAACTGCATTTATTCAGCATATAAGTAGA
TTAGAAACTACTACATAAATCTTAGCTTGTTGCACTTTTTAAATAGTTTTAACTATTTTAAATAATAAAAAAAAAAAA
TTGAATTATCTGTTACATGTTTTAAGGATGCCAGACACCCTCCAGTCATTCCCAGGGTCAGTCATGTGATCACGAGGG
GTTCATGTAGAAGGTAAATCTCTTGCACATGGTCAGTACTTGGAAAGGAAACTTAATGCTGACAGGTGATTTATTTTC
AACTGCTGAATTTTCCTGTAACAGTCACCGTCTTTGTTCTTCAGCTGTCTGAGTGTTTCTGTTACGCTCTGCTTCTCA
AACACATCCATTAGTGCTGTCTCCGCGTTCTCATCGTTCCTCAGGAAGACCTGAGCTTCAATCGTAGCTATGAATTTG
TATTTTTACTTATACTAATTCCCAGCAAT

>Sequ08460EST2
GACTGACGGTGCTTTTCGTTCCATTGTTTGAGACGTACCGACGCACAGCTGGCTTCATATAGCGCTGTAGGGAAAGCC
AAGATGGTGAAGGATCCAAAGAAGCCGAGGGGCAAAATGTCCTCATATGCTTACTTTGTGCAAACATGCCGAGAGGAG
CACAAGAAGAAACATCCTGATGCCAGTGTCAACTTTGCAGAGTTCTCCAAGAAATGCTCTGAGCGATGGAAGACAATG
TCACCGAAAGAGAAAGGCAAGTTTGAAGATATGGCCAAACAAGACAAGGTGCGTTATGAGAGGGAAATGAAGAATTAC
ATTCCCCCCAAGGGCCAAAAGAAGAAGCGATTCAAGGACCCCAATGCCCCCAAGAGACCACCGTCTGCATTCTTCCTA
TTCTGTGCAGACTTTCGCCCCAAGGTAAAAGGTGAGACTCCTGGACTCTCCATCGGAGACACAGCAAAGAAGTTGGGA
GAGATGTGGAATAGCTCATCTGCAGAGGACAAGCAGCCGTATGAGAAGAAGGCTGCCAAGTTGAAGGAGAAATACGAC
AAGGATATTGTTGCTTACCGCACGAAGGGCAAAGTGGATTCTGCTGCTGCTGCGGCAGACGACGATGATGAGGAAGAC
GAAGAAGATGAGGGAGAGGAGGATGACGATGACGATGAGGACGACGATGACGAGTAGATTGCATCGGAATGGGACCTG
AAGTTTTGTTTATGCCATATAACCCTAATATACTCAATTCACCATCTTGAAACAAGTCAGATTGAACAAGAACATGTG
TATATTTAATGTTTTTAACATGTACAGTGTTATGCTCCTTTTTTTGTAAAGTTAACACTACATATCTTAAGTTGAGAT
TTTCTAGTGGTAGTTCTTTATCCCTGCCATATTCTGTAACAATTTAGGAGGACTACAAATCAGTATGAAAGTAATTAG
TCGATAGGTGTTACAACTCAAAAATGAGATTTCTGAGTTGGTAGTGTATTTTTCTTTATTTTGAAAAATATTTTTTTA
TGTGCTGTCCCAATGTCTTTTTATTGTTCTTTGAATACCACTCTAATATCAAATGCAGCTGTCGACATTCAAGAATGT
CTTCAATAAAGGTCGTTTTTTTTTAAAGAACGAAAAAAAAAAACCGAAACCTCCCTAATAGT

>Sequ08562EST2
TCTCACTCGTCAGGGGCGACGTCAACCTGCTGATGCTGGGAGATCCTGGTACAGCCAAGTCTCAGCTGCTCAAGTTCG
TGGAGAGATGCTCACCTATCGGGGTTTACACCTCGGGTAAAGGCAGCAGTGCAGCCGGTCTGACCGCCTCCGTGCTGA
GGGACCCCAACACTCGTGGATTCATCATGGGAGGGGGGGGCCATGGTGCTGGCTGACGGCGGAGTCGTATGCATCGAT
GAGTTTGACAAGATGAGAGAGGACGACAGAGTGGCAATCCACGAGGCCATGGAGCAGCAGACCATCTCCATCGCTAAG
GCTGGCATCACCACCACGCTGAACTCCCGCTGCTCGGTCCTCGCCGCCGCTAACTCCGTGTTCGGCCGCTGGGACGAC
ACAAAGGGGGAGGACAACATCGACTTCATGCCCACCATCTTGTCCCGTTTCGACATGATCTTCATCATCAAAGACCAG
CACGACCAGCAGAGAGACATGACTCTGGCTCGTCACGTGATGAACGTCCACCTCAGCGCCCGGACGCAGACCGAGGGT
GTTGAGGGCGAGATCCCGCTGGCCACCTTTAAGAAATACATCGCCTACTCCAGAGCTAAGTGCGGCCCTCGGCTCTCC
GCGGCGGCTGCTGAGAAGCTGAAGAACAGATACGTGGTGATGAGGAGCGGAGCGAGGGAGCACGAAAGAGAGAGCGAC
AAGAGACCCTCCATCCCCATCACCGTCAGGCAGCTGGAGGCGGTCGTGCGTATCGCAGAGTCTCTGGCTAAGATGAAG
CTGCAGGCCGTGGCTGGAGAGGAAGAGGTGGACGAGGCTCTCAGACTCTTCCAGGTTTCTACACTGGACGCTGCGCTG
TCGGGAAGCCTGTCAGGAGTGGAGGGCTTCACCTCGCAGGAGGACCAGGAAATGGTCTCGCGCGTCGAGAAGCAGCTG
AAGAGACGCTTCGCTATCGGCTCCCAGGTGTCCGAGCACAGCATCGTCCAGGACTTCACCAAACAGAAGTATCCAGAG
CACGCCATCTACAAAGTGCTGCACCTGATGCTGAGGCGGGGGGAGCTGCAGCACCGCATGCAGAGGAAAGTGCTCTAC
AGAGTCAAGTAGAGCTAACCGCTGCTCCGATACTTCCGTACTGTTGTTGATGTAAATAGTTTTTTTTCTCTGCATATT
TGTGCTGCTCTGTATTTACCAATAATACAATAATTAATTTCAAACCTGGGATGGATTGAGTTGGATCTGGATTCTTCT
CTCGTCTGCTCACATGCTCCAG

>Sequ08564EST2
AAACACAAATCTTCATTCACCAAGATGGCGACATTGACAGTGAGCCGGCCACCGATCGCGGGTTTCAGTTTCGAAAAC
TGCAAGAGAAATGCAGTTCTGGAAGGCGAAGTTAACAAAGTGGGATGCAGTTTACCTGCTGCTCGTAAAACAGGAACT
ACCATCTGCGGTGTTGTCTTCAAGGATGGCATTGTCCTGGGAGCTGACACCAGAGCCACTGAGGGCATGGTGGTGGCA
GACAAGAACTGCTCCAAGATCCACTACATCTCCCCCAACATTTACTGTTGTGGAGCAGGAACAGCTGCAGACACAGAG
ATGACCACACAGATCATCTCCTCCAACCTGGAGCTGCACTCGCTCTCCACCGGCAGGCTGCCACGTGTGGCCACCGCT
AACCGCATGCTCAAACAGATGCTGTTCAGGTATCAGGGCTACATCGGAGCTGCTCTGGTCCTGGGTGGAGTGGACTGT
AACGGACCTCACCTTTACAGTATCTACCCTCACGGCTCCACTGACAAGCTGCCCTACGTCACCATGGGCTCCGGGTCT
CTGGCTGCTATGGCCGTGTTTGAGGACCGCTACAAGCCTGACATGGAGGAGGAGGACGCCAAGCGGCTGGTGCGTGAC
GCCATCGCTGCAGGTATCTTCAATGACCTGGGCTCTGGCAGCAACATCGACCTGTGTGTCATCTCCAAGGGCAAAGTG
GACTACATCCGGCCCCACGATGAGGCCAACAAGAAGGGGGTCAGAACTGGTGACTACAAGTACAAACGAGGAACCACC
AGTGTGTTGACGAAGAAGGTGACCCCCCTGGACCTGGAGGTGGTGGATGAATCTGTACAGACTATGGATACGTCCTAA
AATACTTTGACCATGTTTTCTTGATGTAACTCACCACCCGTCAGAATTCCTAATGCAAATGGCTCCTTTTTCCATGAT
TTACAATAAAAGTGTTTGAGTTAGCAAAAAAAAAAAAAAAACCGACCT

>Sequ08572EST2

GTGATGGTGTGACCGCCATGCATAAAGTTTTTGACTGGGCCGGCACAAGTCGACGAGGGCTGCTGCTATTTGTTGATG
AAGCTGATGCCTTCCTTCGCAAGAGATCCACTGAGAAGATCAGTGAAGACCTCAGAGCCACTTTGAATGCGTTCTTGT
ATCGCACTGGAGAACAGAGCAACAAATTCATGCTGGTGTTGGCCAGTAACCAACCAGAGCAGTTTGACTGGGCCATAA
ACGACCGTATAGATGAAATAGTGAATTTTGCTCTGCCGGGTCCTGAGGAGAGGGAGAGGCTGGTGCGGTTGTACTTTG
ACAGATATGTGCTGGAGCCGCCACTGGAGGGAGGCAGAGGATGAAGCTGGCACAGTTTGACTACGGTAAAAAGTGCTC
TGAGATTGCGAAGAGGACAGAGGGCATGTCAGGAAGAGAGATCTCCAAGCTGGGTGTGGCCTGGCAGGCAGCAGCATA
TTCCTCTGAAGATGGGGTCCTGACAGAGGCTATGATTGACGCTCGGGTTGACGACGCTGTCAAGCAACACGTTCAGAA
GATGGACTGGCTGCGTGGAGATGAGGAGGCTCAGGTCAAGACCCTTACCCTCCTGCAGCTGGGGTGGCAGGCAGTGGA
GGCAAAATGGGCTTCAATCTGCCTCTCAGTGAAGCACCCGAGGCTCAGGAGGTGATCGCGCCAGTCCTTGAGATAAAT
GCAAAGCAAGAAGGTGACAATATACCACCTCCTTCTGATATCGACCAATCGGCAGAGGGCAAAAATGCCGTTGGGCAG
GACTGTGAAGACGCTGTCAAAGCAGAAGCTACAACAGCAGCGGAGAGTTTAGCCCCGCCTGCTACCTCCACTGACAGT
GAGAGCAAGGTGGAAAAGGAAGACAAGACTGGATCCTCTCCTCCAAAGGATGGAACTCCAGTTTGAATCCAAGATTTT
TTTCCAGCGGGGTGCCGGATTTAAAAGATTAGTGTCCTGCCCCTTTAACTATTCTGTCAATGTCCATAAACATGTTGG
ACTGTCTTTTATGACAGAAGATGAGTTCACATGGTTGGGGAGATAATCCATTAACAAAACAATGCTGGAGACTTTCAT
TCTAATATATAATGTATACATTGTAAAAGTGTACTTTACATGGGAATCAGATTCTGTGTCTGTAATAACAGTATATAA
ATGTTGTGCACAGTGGAGTGTCTCTGTTGAAATGATTAAATGGGTCTGTGTTTTATAAACACTAAAAAAAAAA

>Sequ08578SNP2
CACTGTGACTGACTTTGGTCGTTGTCCATGTGCACAACATAGAAACTTGACATATTTTGGAAACCATTTGCGCAGGTT
CAAGAACGTGGCAGAAGAGTCAAAGACACTATTTCCAGGTTAAGAGTCTTTGAAAATAATATTGTGTATTTCATGCTG
CTGGTCTACATAATATTAATGCGTATCTTTTTTTGACTTTTTAAATGCAGCATAATATAAACAGGATTTATATTATAT
GGATATCCTGTACTTAAGATGTACAGGCAGGCCTACTCGATGTCAGATTGATATCGAAAACTGATATTGGTCATAAAC
TCAAAGCCAAATCATGTTTTTGAAATCTCTGAAAAATGATATATAGACAGTACCAATGTTGGCATCAATGTGGTTATG
TTTAATGTTGAAACAATGACATCTTTGATTATAGCTGTTGATCATGTGATCACACTCTTAAAGTTGTTGCAGAGGGTG
TTGCATGTTTGGGCAGATGATCTCAGATGTTAATTGTCACATTAATTCTCAGTCAGCTTGTAGCAGCTGCTGTTTACA
TATGGCTCTTTACATTTTGCTCCATATGCTGCATAGAAGACAGTTATAACAGCTTGCAGTACTAATCTGAGTCCACAT
AAATGTAAATCCACTTATATTCTTATTAAAATTATAGGAACACACTTTTGGTGCATAAGACCCTCTGCAGCTCACACC
GAGCTCAGGCTCCAGTAACTGTAGTGTAACAGCTGGAGTTTTGTGGTGATGTGACTGTTGAAGTTGTGGGTAAAGACA
CTGTCCTGTCTTTGAATATTTGTAGAAGTTATTGGATTTTAGGAAAGAAGATGTGTAACTAAAAACATTTTGATTGAT
GGCGGCAACAGATTATTTGTCTCAGTCGTGTTAACTGAATTATATCAGAAGACTCACTTTTACCAAGTGAATACGTGC
AATACATAAATATCTCTGCTTTGATGAAGCACTGTATGTTTGTCTGACAGTCCTAGAATGAAATGTGCATGATGTGTG
TTTTTATTTTATGCTCTCAAATGTTTATATTATAATTTTCCCCAACCTTGAGAATGGCAACACTAAATACTTGAACTC
ATGAATCCAATAAATGACATTTTGTCTCTGTGTAGTGTTGCTAACATCATATAATTACAGTTACATAACTGTAATTAA
AGTAACCTCATTGTAAATAGTTAAATGAATGAAAAAATAGACTAGAGATTTGCTGAAAATGATGTTTTCAATCATTAC
TACTGAAAGTGCTCTTTAGCTTCAAATCACATTATGTTTCATTTGTCAGTGACACCTTCATTTTGTCTGTGAATAGAA
GCTATTTAACATTAAAACATAGCAATGCAATGAACAAAAAAAAAAAAAAACTACG

>Sequ08595SNP2
GAAGGGGGAGTCCTGTAAAAACCTTTATGGCTGCCGTCTGGTTTCATCTCACCTCGTACAGGACTGCAAAAATAAACA
CAATAAACCTTCAGAGTTAAGAGACCCAAAGAAGCTGTAGGAGTTATAAACACACCCCACATTTCTATCCACTGCAGA
CTATGTGGAATTAAAGTGTAAAACATAAAATTCCTGCTTAATTTCAGAAAATTTTTCTCTAAAATTCCAGTTGAGTTG
TTTTAAAACATTCACATCCATTTTGACTCTGGCTTTCCTGACTGCTACTTCTAATTTGCAATTCTGGAGACATACACC
AGTTCATGAAAATGTTTGTTTTGTGAATGTTTGCTGCTGTAAAGTGTCCTCCATCGCTTCCCCAAATCAAATCTCTAA
TTTCACACAGGAGCCATAAACCAGACTGCTGCTGCTGCTGTTGTTGATTGAAAGCCTTTTATCTATATCATGTAATCT
TTTCTTGATCATGTGAAATTCACGGTAAGGTAACATGATTTGTGGGGGATTGAATTTTTTTTTTCCACAAAAACGCAA
CAAGATACTCAAAGCAACAAAGACCCAGGACTGAACATTTCACATGGACTCCAAACACCTTTACTGTATGTTTGATCT
TTGAACTCATAGGACGATGTCATATATCCCAAAAAACCTGCTACTGTTACGTGCAAACTGTCAGACTGTGTATATTGT
TTTTGTATATAAAGAATAATGACGAAAAAAAA

>Sequ08700SNP2
TCTTGCAAAACATAACAGATGAATACATTTTGGCAGGTATATCGTCCACTAAGTAGAAGTTTTGGGGTTTTTTGTTTT
TTGTTGCTTTTAATCATTGATCTTGAATTTAACAGTGAACCTGATTCGAAAAAATGGTTGATCAGTGACAAAAGGCCA
CATTTACGTCCTCAGATTTTGCAGTAGGGTTTGATGAAACATACTTTCCCATGCACAGAGAGAAATGGAGAACGGGGC
ATTTAACAGAGCTTTAAAGCCATCCTCACATGAATCAAATCTCTGCCGCTGGAGAATATCCTAAATCAGTAAAGCTGC
TGATAATGCGGTTACGTGTGGCAGAATCAGGATGCCTCATGGCTTTGAAAGGCCCTGGTGTGCATCATTATGTGTCAG
TGAATTCCCCTTCACAGTCTGAAGCCTGCTGTTGGGGGAATTTTTAATATGACAAAGAAAAGGTACGCTGACTCACAC
GAACTCATGCACAATGTGAACTGCTGAAGATTATTTGTCTGTAATGCTTTATTTGATCACTGAAGTGTTTTTGATTGA
CTGATTTTAAGTATAATTCATATCAGTGTTTTTTATTTATATTCCTCTCCAGCCAAACTGGAGTATTCCACTGCTTGT
GTCTCTACATTCGGCTTGATGTGAAATAAAAAAGAATACTCTGGTTGAGTGCAAGATTTTTGGTCATTCTTCTGGATG
TTTGGGAGTAATCTTTTGGGTGCTGCAAATTTTATTCCCTTTGTGAGTCACCAGTACTGTTTATTGGCACTAGAGGGC
GACAGAGGACACAATATGTCGGCCGTCTGCTTCCTCTCTGCCGAGAGAAATCCTGCTTTATCATCAAATGAATCCT

>Sequ08730EST2
AAAACAGCCAGGAACTTCAATCAGCCCATGTGCAAGGCAGCCAAAACCACTATAGTTGAGGTGGAGGAGGTGGTGGAC
GTTGGGGACCTTTGCTGAAGAGGACATCCACATACCCAGCATCTATGTCCACAAGGTTGTCAAGGGAGCCAGCTACGA
GAAAAGGATTGAGAAACGCACAGTAAGGAAAAGCCAGGAACAGAAGCCCAAACCAAAGAAGGACTCGGATATTGTTCG
GGAAAGGATCATTCGCCGGGCTGCTCTGGAGTTTCAGGATGGGATGTATGCAAACCTTGGTATAGGTATACCCCATGT
TGGCGAGCAACTTCATAAAACCAGACATCACTGTACATCTTCAAAGTGAAAATGGAATTCTGGGACTGGGCCCGTACC
CCACTGAGGATGCGGTGGATGCGGACCTGATTAACGCTGGAAAGGAGACCGTCACAGTGCTCCCTGGGGCCGCTTATT
TCTCCAGTGACGAGTCATTTGCCATGATCCGTGGGGGTCACATCAACCTGACAATGCTGGGAGCCATGCAGGTGTCAA

AACACGGAGACCTAGCCAACTGGATGATCCCTGGTAAGATGGTGAAGGGAATGGGAGGAGCAATGGATTTGGTGGCCA
GCGCTGGAACCAAGGTGGTGGTCACCATGGAGCACTCAGCTAAGGGAGGAAAACACAAGATATTGGACAAATGCAGTC
TGCCTTTGACAGGGAAACAGTGTGTGGATCGTATCATCACAGAAAAGGCTGTGTTTGATGTGGATAACACAAAAGGCC
TGACTCTGATAGAAGTATGGGAAGGGGGCTCACTCCTGAGGATATCAAGGCATGCACTGGCACAGATTTTGAGGTGTC
TCCCAACCTGAGGGCCATGCAGCAAATCTAGAGGAAGCTGGCAAAGGATCTGAGATGTGTTTTCGGTTTGTGTCACTG
TGTTCTCTGTGGTAAAAAGTTGGTGATTTAGCAACCTTTAGATGGGCCAAATGAACTTCTAAACCACAAGTGCTTCTG
CTCAGTTCCCTGGTATTTAGCAGGCAAATATCAAAAGGATTTAGTCCTCACTAGTATTAACTGAATGGGAGCCAACAT
GCATGGTTGTTCTTTCTGATCTGATATGACAAATGATTAGGGGACATTGCATTGTTGCCTTTTACACCATTAAGTTAG
TAATTCAAGTTTGTGCAGCTATTGCCAAATAAGCCATCTGATATTACATAAAAACAGAAATTGTTGTTTATATGTTAT
CTACGAAAAATGCAACGTTACTACTAAGTACTGTGAACAAAAATTATGCAATAAGCTCATACATATTTCATCACTAAT
CATTTTTCCTGTAGGATCAATAAGCTTCTGTGACGCGATACACAGAACTGCAGTAAAAAACGTGTTCTGTAGCTCAGG
TGATTTCTCATCAACAGAGGAGAGTACGTATGTATCTTTAAACTTGTGCTGCACCAAGTAGCAAATGTAAGATATCTT
TTCTCCATTTAGAAAATGAAATATGCAGTATTGAAGTGTGATACTAAGCTCTCCCACAAAAACATACTGTTTGATGAA
CTATGTCAACCTGTGTCGTTTTGTTTTATTTTCTCTCAGCCACATATATTTCAGCTGTTGAGTTGATGGGTATGTCTT
GCATTTCAGCCAGAAAACATGAAGCCTCAACATTTTGTACTCAGACATTTGCTGTTTTCCTGCCTTTGTATATTAAAT
AAAACACAACCTTGTGTGCTGAGTAGG

>Sequ08950SNP2
AATAGTTAATAGTAAAAAAAAAAAAAAAGAAAGAAGAAAAAAAGATTATGTTTCATCATATTTGGATTGATGGAGTTTT
TAGCTTTTGTGAAATCTTTTGTCCACTTTGCTTTTTTATCCTGTCAGTCCTATTTTCTGTGCTCCGATTATCTTATTT
AATGGTCATGTAATATTAATATAAATGCATTTTTTTTGGGTGGGAAGGGAGCAGTTTTGCAATTCTATCATTGTTTTA
TGTTTGTAAAAGCAGCCTAGGGTCAGAAACTGTCAGAGGGAGAATCTCTAATAGGAAAATGTTGTGTGTTTTATTGGT
GAAGAAAAGCCCACATTTAAACGATTAGCATGATATTCTAGGATAACTCTTGTCTTTGGTACATGCTGCAATTTCTTT
ATTGTTTGAAACACACTTATGCTATTAGAAATTTCCAGAACTCTGTCTTCCAGGTCAACTTAAGCTGTCACAATAAGT
AATGTAAGGAGTCCTGAAAATACTTATGTATATTTAGATATAGCTTTCCTGACTGGTAAGGGTGTGCAAAGCAACGCT
CAACCAGCCACTGGCAGGCTGGAGTTTTTGCTATCAAAATTATGTTATTTGCCTCTTCCCCTTGATGTATTTTATTGT
CTGAATTCTGTTCATTTTGATGTTTGTTTTATAATAAAACAACACATGATAGCGAAAAAAAAAAAAAAACCGAC

>Sequ09034SNP2
ATACATTGGAAGTTATTGTCTAAATGTTGAAAAAATGGAGTTGCTGTTTTCTGCAATAATATTTTGATCACAGAAGCT
AAAGCACATATCTTACAGAATTATACGAAATGATGATGACAATGAAGATACTGACGATTATATTAAAAATAGAAACTA
TGTCTAGTAAAACAATATTGTTTTGTATATTTTTAACTGTCTCACTGCCTAATGTAAAACATAATATAAACATGATAT
GTATCTAAATGGTG

>Sequ09113EST2
GAAGAAGCAGAAGAAGAAGAGAGCGGCTCGCTGCTATCATCACGTTTCTGTGTAAAAGTGAAAGTTTGGTGTTGTGAG
TGAAAATGGACCCGGTGGAGGCGGCTGTGGCGGCGGCCAAGGAGATCGACGGCAGCGTGATGGAGGGGGGTGGACAGA
TCCTGAGAGTCTCCGCGGCGCTCAGCTGCATCACCGGTACCGCAATCAAAATCTTCAAAATCCGAGCCGGCAGGAGCA
CACCGGGGCTCAGACCGCAGCACCTGAGCGGCCTACAACTGGTCTCAGATCTGTGCTCTGGCAGTCTGCAGGGAGCCA
GCATCGGCTCCACCGACATCAGTCTGACTCCAGGAAAGATCCAATCTGCAAACCACACGGCCGACACACAGACAGCAG
GGAGTGTGTGTCTGCTGCTGCAGGTCGCTCTGCCCTGTGCTCTGTTTGCTGATGCCTCCTCACAGCTCTGTCTGAAGG
GAGGAACCAACGCTGAGATGGCTCCTCAGATCGACTACACTGTCAAGGTGTTTAAACCCATTGTGGAGAAGTTTGGAG
TCCATTTTGACTGTGACATCAGAATGAGGGGTTACTACCCTAAAGGTGGAGGTGAGGTGATGGTGACAGTGAACCCGG
TGAAAGAGCTGCTGCCGGTCACCATGACGGAGAGAGGAAACATCACCAAGATCCACGGCAGAGCGTTCGTCGCTGGAG
TACTGCCCTTCAAATTGGCTAAAGACATGTCGACTGCTGCTGTCAGAACCATCAGGAAGGAAATCAAAGAACTTTACA
TCAACATCCAGGCGCTGCAGGAAAAAGAAAAGGCCTATGGTAACGGCAACGGCATCATAATCATCGCTGAGTCGTCAA
CAGGTTGTCTATTTGCAGGTTCAGCTCTGGGGAAGAAAGGTGTGTATGCAGATAAAGTTGGTATTGAAGCTGCTGAGA
TGTTGTTGAGAAACATCAGACACAATGGCTGTGTGGATGAGTTCCTCCAGGACCAGCTCATCATCTTCATGGCGTTGG
CAAAGGGAACGTCTCGGATTCGAACAGGAGCCGTGACGCTGCACACACAGACGGCCATCCACATCGCAGAGCAGCTCA
CTCAGGCAAAGTTCTCAATAACAAAGTGTGAGGACGAGCTGAGCAGTAATGTCAACCACATCATCGAATGTCAAGGAT
CAGGAGCCACCAACCTCAACCTGTAGAGGGAGACACACACACACACACA

>Sequ09151SNP2
TGGCATTGACTTCTTTCACACCACACGCCGCTGTAGCTTGTGGCCTGCCGGAGGCTACAGGGCCACAAGCATGGTATA
GGAGTGGAGACAAAAAGCCAAGAGTCTCTATTTTTTTGTGGTCTTATCACTATCCTAGACTAATCTTGTGTGAGCACTA
AACCACTATAAAATGGCTGACTCAGATGGAGCAGAGGATGGCCAAGACCTGGAGGCTCCAGAGGAAGAGCTGGATGAC
CAACAGCAAGCCCTACTGCACTTCAGTAAATACAGTGAAGCCCGGCGAGCGCTACCTTGGGAGCAGTGGACCTTCCGA
GAAAAGGCAAACTATTACATGGACCGCATGTTCCTCGGGTTGTTGGTTATTTTCTTCTTCATGCTGGTGGGAGAGTTT
GCCTATAAAATGTGGTATGTGACGAACGTGGAGAAGATCACAGAGTTTGTGTCAGACTCGGTGGTCTTTGTGTCCCAC
TGGCTCTTCACACAGGAGAGACAGGAGCAGCTGGCTGAATTGTAGTTTGATATTAGAGCAACTGTTAATCTATGGAGT
GTACCCAGTATTGCTGTGTGGGATGAACACTTGATTGTGTTTTATACAATGTTTTAGGCGCATGAGGCATTACAAATG
CCCAGTAAAGAATTCATTTATAGCTACATCTTGACTAGTGATACATCAACATATGAATGATTTCTGAGCTTTTATTTT
TGATGGTGACTTAATGGAAATAAATGATGTGTGCAATTAACTGAATCATTGTTCTGTCCTACTACACACAAGATTTGA
GTATCTCCACCTTTTGGTCGTCATCGTTCATTTGTGCATGCCTGTTTTATTCTCAATAAAATGTCATTTAA

>Sequ09198SNP2
TACTCTTTGGTTGTTTACTTGTCAGCTGTCTATACAACCGAACACGTTATAAAAGTGTTTTAAAAATAAAACTCATCA
TAAAACTGCTGCTGACGTCACTACCGAAGTATCGACGAAACAAAGGAAGAAACCGACAAACACCGGAACTCACCTGTT
TGTCGATTTCAGCAGATTTAAGTGAGTTATTGTGAAGTTAGCTGAGTTAGCTACGTCAAAGCTAAGTGACGCTGAGTG
TCCACCAGACTGCACTTTGTTCCAGGGAAAGTTCAGGAGCAGCAGAGGTCACCTGGACAGTTTGACAGCCCCCCATCC

CGCCCTTCACCCCCCCAATCCTCCTGGACCATGGCGAACCCTCTGAGAGCTGAAGTGGTTCGACTCTATAAAAATCTC
CTCTACCTCGGTCGAGAGTACCCCAAAGGAGGCGAGTACTTCAGAGACCGACTGAGAGCGGCGTTCACCAAGAACAAG
TCAGTCCAGGACCCGGAGCAGATCAAGGAGATGATCGCTCGTGGGGAGTACGTGGCCCGAGAGCTGGAGGCGCTCTAC
TACCTGAGGAAGTACAGAGCGCTGAAGAAACGCTACTATGAGGAATAAACAGCTGCCGCAGAGACTCAGGACAGACTC
CACCCACTGAAACCACCTGTGTTAATCACCATCAGCTGCCAGAAAAAACAAGACACGTACAAAATATAAAGAGTCTTT
GTTTATTTCAGGCTCATTAAAAACTGGAATGACAGAACAACAGAAATGAGCAGCAGCTTCGAGGTTTTAATGTTTTAT
TCCTGTCGAGTTCATTCAGCTCTGAGAGAAAACACTTAAATAAAGTCATAAAAAAAGA

>Sequ09223EST2
CACCGTGGCCGAGCTCCGGGAAGACGAGGACAGGCCTACTTCAGTTCCTACGGCCATTATAGCATCCACGAGGAGATG
CTGAAGGNTAAAGTGCGCACAGAGAGTTACCGTGACTTCATGTACCGCAACCCTGAGGTGTTCAAAGACAAGGTGGTG
CTCGATGTGGGCTGTGGGACTGGCATACTGTCCATGTTTGCCGCCAGAGCTGGGGCCAAGAAAGTGATAGCAGTTGAC
CAATCAGAAATCATCTATCAAGCCATGGACATAGTCAGGTCCAACCAGCTGGAGCTGGAGGACAAGATCACTCTGATC
AAAGGCCGCATAGAAGACATCAACCTCCCAGTGGAGAAGGTGGACATCATCATCTCAGAGTGGATGGGTTACTTCCTG
CTGTTTGAGTCCATGTTGGACTCAGTCCTGTACGCCAGAGACCTCTACTTGGCTGACGGTGGCTCAGTCTATCCAGAC
CTTTGTAATATCAGCCTGGCAGCACTGGGTGACACACAGAAGCACCAAGACCGCATTGCATTCTGGGACGACGTGTAT
GGCTTCAACATGGCATGCATGAAGAAGGCTGTGGTGCCTGAGGCTATGGTCGAAGTGGTGAAAGCAGATACCCTCATC
TCTGAGCCGACAGTCATACAGACGATTGACTGTAACAGAGTGTGTCTGTCTGAGCTGGAGTTCGCATCAGATTTCT

>Sequ09293SNP2
TCCAGGGTCGTCACTATGAGTGCAGTAATGACTGTCCTGAGATGCAAAACTACTTCAGCCGCTGTAACTCGATAAGAG
TGGAGAGCGGTTGTTGGGTGGCCTACGAGAAGCCCAATTATGGCGGCTACCAGTATATGCTGCACAAGGGCGAGTACC
CCGACTACCAACGCTGGGCAGGCTTCAATGACTGCATCCGCTCCTGCCGTATGGTGCCACCTTATAATGGGAACTACA
GGATGAAGATCTTTGAGCGGTCTGACTTTGGGGGCCAGAATCTGGAGCTAATGGACGACTGCCCAGATCTGCACGAGC
GTTTCCACACCCGTGACATCTCCTCCGTCAACGTCATGGAGGGCTACTGGATGCTGCATGAACACCCTAATTACAGGG
GACGCCAGTACTTCTTGCGTCCCGGAGAGTACAGGAGGCACAGTGAGTGGGGAAGCACCAGCCCTACCATTGGCTCTC
TGAGACGTGTCACTGAGATCAACTGATTCCCAGTTGTTTTGTTTTTTACCCTTACTGAAGCCCACTGTTGAGCTTTCC
ACCCTTTTGTCATCCTAATGCCAACAGTGTTTCAATGTCACAACAATGTGGTGTCCTAACCTGAAATTATACATTTTA
ATTGATGGGAGTTTTGGTGCGTCTGATGTTGGGTTCTGGTTTTGTTGGGTTGCTCTGCATTGTTGGGTAAAGTTCCTC
CCCAACTCAGTGCTCAAGCAATGCTCACAGTAAATAGTGATGATCTTTTTTGCTTTGGTCGTCCCATTGCAAAATGGT
TTTGCCTCACTAGTCCCCAACCTCCTGTCCTACTGGAATCACAATAAATGAAAACTTGATTAAAGTACAAAAAAAAAA
AAAAACGAAACCTACCC

>Sequ09322SNP2
AAGAGCTGATGGAACAGGACGCACCTGCTGCACGCTTTATGAAGACCATAACATCTAAATGCTGGGCCTACTCCAAAG
AGGCTATGAAGACAAAGAGGGACGTGTACTTCAGACCTGGCTTCGTCTCTTATGACGCCTATGCAATGGCGGCCTGCA
TCGACGGCAGCGTGGTGACGGAGAGCATCGAGTGTCCTGTCCGTGTGGAGCTGCAGGGGTCGATGTGTCGCGGCATGA
TGGCTCTCGATCGCACTAATAAGCTGAAGAAGAGTCACAGTGTGTTTGTTATGTCTAAATGTGACGTGGCCAAGTTTA
GTCAGCTACTAATGGAGTCTCTAAGACAACCGTGTAATAAATAATCTTTGGTGTAATTCGAGGAAAATAGATATCTGG
CTCATACAGGCGTGAGAAATAACTTAACTTAAATAATTTAAACCGAATGGTTTCATGAGTATGACCATGATGTGAATC
AGATGCCGTGACCTTCAGTTAGAAATGAAGAAGACGTTAGACAGCGCTCTCCACCACCATCATTCACGCAACAAACAA
GGGAATATCTTTCAGAAGAATGGTGTTCATCTCTCCAGTAGAGATTCCACAGACACAGAAGATGTTTTTCTTCTTTTC
TTTCTCTAATCTGTCACCGATGTGTAAGTGTGTAGATGCAAATGGAATGAAATCACAAAAAACAGATGTTTTGCAGAA
ATCCTTTATTTAAATTTGCTGACAAAAAAAAAAG

>Sequ09323EST2, Sequ09323SNP2
GCATATACAAACACTCAGTCAAGCATCCACGCTCACCCCACAAACTGCTCTAGTGGCCAGCAGAGAACTGACTGATTC
CTTCATACAGGCGGCATGGCTAACAAAGGTCCATCCTACGGCATGAGCCGGCAGGTTCAGGATAAAATTGACAGCAAG
TATGACCCTGAACTGGAGCAGATCCTGGTGGAGTGGATTGCCCGTCAGTGTGGCTCCAGTGTGGGGAATCCAGAACCC
GGCAAAATGGGCTTCCAGGCCTGGCTTAAAGACGGATGTGTCCTTATCACGCTGATTAACAGTCTGTCTACTGGAGAC
GGTCCCTTGAAGAAGGCCCACACCTCACCCATGGCCTTCAAACAGATGGAGCAGATCTCCCAGTTCCTCAGTGCTGCT
GAGAAGTATGGCGTCATCAAGACTGACATGTTCCAGACCGTGGACCTCTGGGAAGGGAAGGACCTGGCGGCGGTGCAG
AGGACCCTGTCGGCTCTGGGCAGCTTGGCTGTTACCAAGGATGAAGGCACATACAAAGGAGACCCTAGCTGGTTCTTC
AAGAAAGCACAGGAGAACAAGCGAGATTTCAGCGATGAGCAGATGAAGGCGGGCAAAAATGTTATTGGCCTACAGATG
GGCTCCAATAAGGGAGCCAGTCAGGAGGGTATGAGCTACGGAAGACCCCGGCAGATCATGTAAAACCACTGAGCCACA
AGATACCAAAACCTCTGCTGCCCCTGACACCCTCTAAGAGCCTGTAAGCGTCTAATGCTTCCCCTTTCATCGTAAAAC
CTTCTCTGCTTTAGAAATGATTTACTTTCCAACAAGTTCCCTCTCCTCTCTAGTCTTGACACTTGCCCCGAGAACCCT
TATGTAGATGCTTACACTGGTGACCGTTGCAGAGCTCACCATGCTTTGTCCTTCCCACTGCAGTTAGGACCAGTAAAA
CTGATAAAAAAATAACCATAAAAATGACCAACTCATTGCCTTGGAGTTAAAAACACGAAGACGACCATGTTCCAAAAG
TCATGTTGTTCCTTCCTAATATGAAACAGCATTGATATTACAGATGACTGAGCACTGTCTCATCTCTGTGTACCAAAC
ATCTGAATCATGGCACCGGTTTCCTAAATTTTACAAATAGGCTATTGCTTCTGGATTCTGACTCAGAGATCGATTTTT
GTATGTAATTTGCAGAAGGGACATTCTTTACAATTATAGTGGTGACACAACCTGTGGCATACCAGTAACTACTCCTTA
ATTTTACTGTTACAATATTGTTCCACCACTTCCCACAATGCTCTGCCTGCCATATCAGTGCCCATATTTTTGTACATT
GTCTTATTAAATAATGACTTTGAAATACATCTCTGAATTGACTTTGTGATTCTGGTATTGAGTGTTGTTTGAAGACAG
ACTTAATAAATGTGCTTCCA

>Sequ09495EST2
AGGGAGCTCACTCAGGACAGGGACTTGGAAATGCCTTCCTCTCCCACATCAGTGCTTGCGACGGCATCTTCCACATGA
CCCGTGCTTTTGATGATGAGGATATTATCCACGTGGAGGGTAACGTGGACCCAGTGAGGGACATCGAAATCATCCACG
AGGAGCTGCGACTAAAAGATGAGGAAATGATGGCTCCAATTATTGACAAACTGGAGAAAACCGCCGTGAGAGGAGGGG

ACAAAAAAACTCAAACCTGAATATGATATCATATTGAAGGTAAAGAACTGGGTGGTGGACGAGAAGAAACACGTCAGA
TTCTACCATGACTGGAATGACAAAGAGATTGAGGTGTTGAACAATACCTGTTTCTTACATCCAAGCCCATGATCTACC
TGGTGAATCTCTCGGAGAAAGATTACATACAGAAAAAAGAACAAGTAGGTTGGTAAAAATCAAGGAGTGGATAGACGC
TCATGATCCTGGTGCTCTGGTCATTCCCCCTGAGTGGAGCTCTGGAGTCCAAGCTGCTGGACATGGAAGATGAAGAGG
AGAGGAATAAGTACTGCGAGGAAAATGAAGACACAGAGTGTTCTGACCAAAATAATAAAGACCGGCTATTCAGCACTG
CAGCTGGAATACTTCTTCACAGCAGGACCAGATGAGGTGCGAGCGTGGACCGTCAGGAAAGGTTCCAAGGCTCCTCAA
GCTGCAGGAAAGATCCACACTGACTTTGAGAAAGGCTTTATCATGGCCGAGGTGATGAAATACAGTGACTTCAAAGAG
GAGGGCAGTGAAAATGCAGTCAAGGCTGCTGGGAAATACAGGCAACAGGGCAGGAACTACATCGTGGAGGATGGGGAC
ATTATCTTTTTCAAATTCAACACACCAAACGCCCCTAAAAAGAAATGAGACCATACAATCACCAGAGGGAGAACTCAG
AATACTGTTCAGTCCGGCCCCTGAGAGCTACACTGTCTGCTGGCTTTCATACTTCTCTCTTGATTTCAGAGGATCAAC
ATGCAGCCTGGTCGGCCAGAGATATTTACTCACCTGGCCTGGTCTGGAGCAGAATCTATCAAAATGATTCTGTATAAT
ACGAATTCTATACAAACACACCATACATTCCTATGTATAAGAATATGAACAAATTTTGAAATGGTACCAACTGCACTG
CCAGTCAAACATTGTTGAACACACTCACAGTCAAGAAGAGATGTGACTGTAAT

>Sequ09509EST2
ATTAGGTCCCAGTCCCAGTCCACACCTGACAGCAAAATGTCTGACAAAGGAACCTTCGATACCAACGTGCTGACCCTC
ACCAGGTTTGTTCTGGAGAGGGCAGGAAGGCACAAGGGACAGGTGAGCTGACTAACCTGCTCAACTCCATCTGCACTG
CTGTCAAAGCCATTTCCACTGCTGTCAGGAAGGCTGGGATCGCTAACCTATATGGCATCGCTGGAAGCACCAACGTGA
CAGGGGACCAGGTGAAGAAGCTGGATGTCCTGTCCAACGACCTGGTCATCAACATGCTCAAGTCTCCTTCTCCTCCTG
CATGCTTGTGTCAGAGGAAGACGAGAGGGCCCTCATTGTGGAGCCAGACAAGAGAGGAAAATACATTGTGTGCTTTGA
TCCTCTGGATGGTTCCTCAAACATTGACTGTCTGGTCTCTATTGGAACAATTTTTGCCATCTACAAAAAGACCACAGA
TGACGAGCCATCTGAGAAGGATGCTTTGCAGCCTGGAAGAAACATTGTTGCTGCTGGTTATGCTCTGTATGGAAGTGC
CACCATGATGGTCCTCTCCACTGGTCAGGGCGTCAACTGCTTCATGCTTGACCCTGCGATCGGTGAGTTCATCTTGGT
AGATCGAGATGTGAAGATTAAGAAAAGGGGAAAAATCTACAGTTTGAATGAAGGATATGCGCAGCACTTTTACCCAGA
TGTGACAGAGTACCTGCAAAAGAAGAAATACCCGGAGGATGGTTCTGCTCCATATGGCAGTCGCTATGTTGGTTCAAT
GGTAGCTGATGTTCACCGTACTTTGGTGTATGGAGGAATCTTTTTATATCCTGCTAATGTCAAGAGTCCTAAGGGCAA
GCTGAGGCTGCTGTATGAGTGCAACCCCATGGCCTTCATCATGGAGCAGGCAGGAGGCATGGCCACGACAGGATCCAT
GAATGTTCTGGACATCCAGCCCACCAATATCCACCAGCGAGTCCCTGTGGTCCTTGGATCCCCTGATGATGTGCAAGA
ATATCTTTCAATCTACAAGAAGCATAACAAATGAGGTACTCAGATCTGCTAAAACATACTCAAGACAGTTTAAAGTAA
TCATCGACTGCCAAACTCTCTGGGAAAATAAATTAGCCCCCCCCCCACCCCCCAGTCTCTTTGAATACAGCAGGTCAA
GTCTGGTGTTGAGCAGAACTAACACTAAGAGACTCTATCGGGACATTTTCACTCATTCCCTCAGCTGCACTGATGAAG
GAACCATGAGCTTCACTGGACCCACGAACTCCACCTCACACTGTGCCACTTCCAGCTCATTGCTGTGTGTGTTATTTG
AAGGGACTGTAACCCTGTA

>Sequ09514EST2
ACGACTATGCCGCCAAGAAGATGGGCATCGACCACAAAGGCCAGGTGTGTGTGATGATCCACAGTGGCAGCAGAGGCC
TAGGACACCAGGTGGCCACAGATGCTCTTGTTGCCATGGAGAAGGCGATGAAGAGGGATAAGATCATAGTGAACGACC
GTCAGCTGGCGTGCGCCCGCATCACCTCTCAGGAGGGTCAGGACTACCTGAAGGGGATGGCTGCCGCCGGAAACTATG
CCTGGGTCAACCGCTCGTCCATGACCTTCCTCACCAGACAGGCGTTCTCTGAGGTCTTCACCACCCACGCCGGACGACC
TGGACATGCACATCATCTACGACGTCTCGCACAACATCGCCAAGGTTGAAGAACACATGGTGGACGGGAAACAGAAGA
CTCTGCTGATTCACCGCAAAGGATCCACCCGAGCTTTCCCCCCACACCACCCCCCTCATACCTGTAGACTACCAGCTC
ACAGGTCAGCCGGTGTTGATCGGAGGGACAATGGGAACCTGCAGTTACGTCCTAACGGGGACGGAGCAGGGGATGACG
GAGACGTTCGGTACCACCTGTCACGGAGCGGGTCGCGCTCTCTCTCGAGCGAAGTCCCGCAGGAACCTGGACTTCCAG
GACGTTCTGGACAAACTGGCCGACCAGGGCATCGCCATCCGAGTGGCTTCACCCAAACTGGTCATGGAGGAGGCTCCT
GAATCGTACAAAAACGTGACGGACGTCGTCAACACGTGTCACGACGCTGGGATCAGTAAGAAGGCGATCAAACTGAGA
CCCATCGCTGTGATTAAAGGCTGAAGCTGCACAGATCTTTATTTAAAGACAATTTACAACAAACAAAAGGACATGAAG
ACAAAAAAAACTCAAGACGCAAAATCCACAGGAATCTTTAAAGACAGAAGATATTTGCTCAAATATTTTTTTTTTTGG
AATTTATGTTGAAGAGCCTGAAATACACTAATAAGAAAATAATTGATTGAACATTTTATTTTCCTGTTTATGCCAAAT
CTGTGGTCCAAATAAAGGTGATCACTAGAAAAAACCGAAAAAAAAAAAAAACCGACCT

>Sequ09539EST2, Sequ09539SNP2
AGCTGCAGAAACAACTGGAGAAGCAGAGGAAGCTGAAACAAAAGCAGCTTCTAAAAGATTCTGGGGAGAAAGTGGCAG
AACAGATTAAAGTGCTTGGTAATGGTGAGAATGACAATGATCCTGATAGGAGGAACAACATTGTTTTCAACGCCACCT
CAGAGTTTTGCAGAACTCTGGGTGATATTCCAACTTATGGACTGTCAGGCAACAGAGAGGACCAAGAAGACATTATGG
ACTTTGAACAGGAGGAAGAGAAAGATGATGCTGGAGATTCAGACTCTGAAAATGGATGAGAACGTTGGATGGAGCACA
GTCAACCTGGATGAAGAGCAAAAACAACCTGACTTCTCCACAGCCTCAGCCACCATTTTAGATGAAGAGCCCATCGTC
AACTCTGGCCTTGCTGCTGCCTTGCTTTTGTGCAAAAACAAAGGTCTGTTGGACACTCAAATGCAGAAGGTAGCCCGT
GTCAAAGCCACAAAGGGCGCCCTGCCCAACGACAACTACTGCATTGAGGACAAGATGGGCTTTGATGACAAGTACAGT
CGCAGAGAAGAATACAGAGGCTTCACTCAAGACTTCAAGGAGAAGGATGGGTACAAGCCTGACGTTAAGATTGAATAT
GTGGATGAGTCTGGGCGGAAACTCACTCCAAAAGAAGCTTTCAGGCAGCTTTCACATCGATTCCATGGGAAAGGATCT
GGAAAGATGAAGACAGAGAGAAGGATGAAAAAGCTGGAGGAAGAGGCACTGCTGAAGAAGATGAGCAGCAGTGATACT
CCTCTGGGGACTGTTGCCTTGCTTCAAGAGAAGCAGAAGTCTCAGAAAACACCATATATTGTGCTTAGTGGGAGTGGA
AAAAGTATGAATGCAAACACCATCACTAAATGAGTGAATGGAGAAGAGTGGATTATCTTTAATACAAACGTACACATT
CATACACAGGATGTTTTAAGTAAAGTAACTGCTATAATGTCATGCCCCAGTACATACAAGTGTCACTGCTGTAGTAAG
AATTGTTGAATAAAAAT

>Sequ09683EST2
TGACAAAGTTGAGCTGGAAGCCACTGATAATGCTATTGATACCCTGGGCTTCACTGGTGAGGAGAAGATGAGCATCTA
CAAGATGACTGGTGCTGTGCTCCACCATGGTAACATGAAGTTCAAGCAGAAGCAGCGTGAGGAGCAGGCTGAGCCCGA
TGGCACAGAGGATGCTGACAAGGTTGCTTACTTGTTGGGTCTGAACTCCGCTGACATGCTGAAGGCTCTGTGCTATCC

CAGAGTGAAGGTCGGAAATGAGTTCGTCACCAAGGGACAGACTGTACCTCAGGTCCTGAACTCAGTCACTGCCCTGGC
CAAGTCTATCTATGAGAGGATGTTCTTGTGGATGGTCATCCGTATCAACCAGATGTTGGACACTAAGCAGCAAAGGAA
CTTCTTCATTGGTGTCCTGGATATTGCTGGCTTTGAAATCTTTGATTACAACAGCATGGAGCAGCTGTGCATCAACTT
CACCAATGAGAAACTGCAACAGTTCTTCAACCACCATATGTTTGTCCTGGAGCAAGAGGAGTACAAGAAGGAGGGTAT
TATCTGGGAGTTCATTGACTTCGGTATGGACTTGGCTGCCTGCATTGAGCTGATTGAAAAGCCCATGGGAATCTTCTC
CATCCTTGAGGAGGAGTGCATGTTCCCCAAGGCCTCTGACACAATCCTTCAAGAATAAGCTGTATGACCAGCATCTTG
GCAAAAACAGAGCATTTGAGAAGCCAAAGCCCGCCAAGGGCAAGGCTGAGGCCCACTTCTCCCTGGTGCATTATGCTG
GCACTGTGGACTACAATATCACTGGCTGGCTGGACAAGAACAAGGACCCACTGAATGAGTCTGTCGTGCAGCTGTACC
AGAAGTCCCCAGTTAAACTGTTGGCATTCCTGTATCCACCTGTTGTTGAGGAGACCGGTGGTGGAAAGAAGGGAGGCA
AGAAGAAGGGTGGCTCTATGCAGACTGTGTCTTCACAGTTCAGGGAGAACTTGGGCAAGCCGATGACTAACTTGAGGA
GCACCCATCCTCACTTTGTGCGCTGCCTGATTCCCAATGAGTCAAAGACTCCAGGTCTGATGGAGAACTTCCTGGTCA
TCCACCAGCTCAGGTGTAACGGTGTGCTGGAGGGTATCAGAATCTGCAGAAAAGGTTTCCCCAGCAGAATCCTCTATG
GTGACTTCAAACAGAGGTACAAGGTACTGAATGCCAGTGTCATCCCTGAGGGCCAGTTCATTGACAACAAGAAGGCTG
CAGAGAAGCTACTTGGATCCATTGATGTTGATCATGAACAGTACAAATTCGGACACACCAAGGTGTTCTTCAAGGCCG
GTTTGCTGGGTACCCTTGAGGAAATGAGAGATGAAAAACTGGCAGCTCTGGTTACCATGACTCAGGCTCTCTGCCGTG
GATACCTCATGAGAAAGGAGTTTGTGAAGATGATGGAAAGGAGGGAAGCCATCTATACCATCCAGTACAACGTCCGCT
CATTCATGAATGTCAAGCACTGGCCATGGATGAAGGTGTACTACAAGATCAAGCCACTGCTGAAGAGTGCTGAAACTG
AGAAGGAGCTGGCTCAGATGAAGGAAAACTATGAGAAGATGAAAACTGACTTGGCTACTGCACTGGCCAAGAAGAAGG
AACTGGAAGAGAAGATGGTGTCTCTTCTGCAGGAGAAAAACGATCTGCAGCTCCAAGTAGCATCCGAATCAGAGAATC
TGTCAGATGCTGAGGAGAGATGTGAGGGACTCATCAAGAGCAAGATTCAGCTGGAGGC

>Sequ09813EST2
TGGAGATTGAGGTCAGCGTCCTGATGGGGGCCAACATCGCTAGCGAGGTGGCAGATGAGAAGTTCTGCGAAACCACCA
TTGGAGCCAAAAATGAGGGAAACGGCCAAATCTTCAAAGAGCTGCTTCAGACTTCCAACTTTCGCATCAACGTCGTAC
ATGAGAGCGACACAGTGGAGATGTGCGGAGCCTTAAAGAATATTGTGGCAGTAGGTGCTGGATTCTGCGACGGCCTCG
GTTTCGGCGACAACACCAAAGCGGCGGTGATCAGGCTGGGTCTGATGGAAATGGTCGCCTTCTCCAAACTGTTCTGCA
AAACCCAAGTGAGCTCCTCCACCTTCCTGGAAAGCTGCGGCGTGGCCGACCTCATCACCACCTGCTACGGCGGACGGA
ACCGTAAAGTTGCCGAGGCCTTCGTCAAAACAGGCAAGTCAATCGCTGAACTGGAGGCAGAAATGCTCAACGGCCAGA
AGCTTCAGGGTCCCCAGACTTCGGCTGAGGTCTACAAGATCCTGCAAAAGAACGACATAGTCAACCAGTTTCCCTTGT
TTGCAGCCGTCTACCAGATCTGCTTTGAGGGCAAAGAGGTGAAAGAGTTCATCACCTGTCTGCAGAACCATCCAGAAC
ACATGTAATGTGGCCAGTGTGCAGGCAGGTCCACCAGCTGCTCCTTCAGTAACAATTTACCATCACTAGAGGGTGAAA
GAGGTTCATAAGGTAGAAACGCACAATACAACAAAGCAAACTCAACCACTTTTTACTTAAGATCATTTTTTATCGGTG
ACATTTCATTTAAAAAAACTAACAAACTATATTTGTTCTTAGCTTCAAAAGCAATTTATTAATTGACTTTATTCTTAT
TTGCGAAAGGTTCTTTAATATTTTTTGGGCTTTTGTGCAGTGAAGTAAAAATATCAATTAAATGATACAAAGCTGTAT
TTTTTAACTGTGGTTAGTATAAAACTCTCTCTAAGGCAGACCACTCCACTTGTATTCCTACTATAATTGCTGTAAAAA
GTAATTTTGTATTGTGTCACATAAATGCAACAATGTCGGTCTTGATTTCACTACATGCACGTGTGTTTACAATCTCAA
AATTATTATTTTTTATCCGTATGTGGAGTTGTTTTCTCAATTAAATCAATTCAT

>Sequ09916EST2
AGCGACTCAACTTTCATCTCTACAGTGCCCTAAAGAAGGCCTTGTTCAAACCAGGGGCATGGTTTAAAGGTATTCTGA
TTCCTCTGTGTGAATCGGGGACTTGTACTCTCAGGGAAGCCATCATCATCGGGAGCATACTCACAAAGTGCTCAATCC
CTGTGCTCCACTCCAGCGCTGCGATGCTTAAGTTGGCAGAGATGGAGTATAACGGCGCCAACAGCATTTTCCTGCGTC
TCTTGCTTGACAAGAAATACGCCCTGCCTTTCCGTGTCCTAGATGCCTTGGTGGCCCACTTCCTGTCCTTCCGCAATG
AAAAACGTGTGCTTCCTGTGCTGTGGCATCAGAGTTTACTCACCCTGGCTCAGCGCTACAAAGCTGACCTGGCCTCCG
AACAGAAGACGGCACTGCTGGAGCTGCTAAAGATACAAACACACCCTCAGATATCTGCAGAGATTCGCAGAGAACTGC
AAAACTCAGAATCAAGGGATATTGAAATTGGGCTCCCTGTTACAGTGGAAATGGATTGAGGATTCAGTCTGGTCTGTT
CTTCTGCTTGGATGAGTTCAGTAGTGATGTAGATGTTGTAATTTCATATCATGAATCTCTGAGCTTTTTTCCTGCCAT
TGCAGTGCAGTGATTGCTTAGAAATACAAAGACTGGTATTTGTGTACATTTGATTACATTTTAATGCAAAAAATTGGA
TTAAAAACAAACTG

>Sequ10028SNP2
ATCTTTTCTTGAGTGTGTACTGTACTTCACTTCCAGCACTAAGGCCGGTGGTTGTTTGAATGTATGCTTTTACTTGCT
CTCCTTCTCCTCGCTCTCTTCTTTAAACCATGCTGTTATTTTCTATGAAAAGGGGTGTCGACTGTGGTCCAACTATTG
CTAGCTTCTGTTTTCAAGTAGCCCATTGCAGCACTTACCGATTAGGGCTTGGTCAATAAGTTGTCTTTAGGTGGCACT
TTACAACGTAGAAGTTGGTTTAGATCAGTGTGAAGATGCTTTGGCATTTGAGCACACGGTTTGACGGGTGTTTGTCAA
CAGGTTTGGTGACTGCATGTGTGCTTTTCTATGTCTAAATGGAAACTGATGCATGCAACAAGCTTGCTTCTGGAGTTG
TTTCAGTTTTTATTTTTAAATCAATGGTGATAGATTCAAGTAATTTATCATGCAAATAGTGTTTTGTCAGCTTAAGCC
CGTCAACATTTAGATTTCGTCTCTGAAGCATTTGTTTTCCTTCATGGCTGAGGCATTGTGTGACACCATAGCTAAAAA
TGTGCCCTCGCCACTTAGAAATTCAGTTATTGCCTCTGACCAATCTTATCTGTTAAATGTTTGAGGATCTCTCTGCTG
GTTTGCACTACTGTACACTTTTACATCCTGTTGGAAATGTCCTGTGGCCTCTGGCTTCTTCACACCAACAGGAGGAAG
ATAGATGATTGGAAATGGCGTCATTAAAACTGAAGTCAGTGATCAAGTCTTGCCTACTCTATGCTATGACTACCTTCT
GTTAGGGTCTGTAGATACTGCGCGATACAAGTGTGGAACTGCCTGGCATGCTTGTAACCGTCTTGTACACTGTATGCT
GGACACTGGTCTGTTTCTGCTACGCTATTTACCCCCTTTTTTCACCTCCTGAAATAAAAATTACACTATCAGACAACC
AAAAAAAAAAAAAACCGACTA

>Sequ10037EST2
GCCAGAGAAGGAGAGAATTNAGGCTCAAACAAACCCTTTGATGCCAAGAGTGCCTGCTATGTCGTTGATGCCAAGGAG
CTCTACTTGAAGGCAACAATCCTCAAGAAAGACGGTGGCAAAGTCACAGTCAAAGTCCTGGACACTCAGGAGGAGAAG
ACAGTTAAAGAAGATGATGTCACTCCAATGAACCCTCCCAAGTTCGACAAAATTGAGGATATGGCCATGATGACCCAT
CTCAATGAAGCCTCTGTCCTGTATAATCTCAAAGAGCGTTATGCAGCATGGATGATCTACACCTACTCTGGGTTGTTC

TGTGCCACCGTGAACCCCTACAAGTGGCTCCCAGTGTACGATGCTGAAGTTGTAAACGCTTATAGAGGCAAGAAGCGT
ATGGAGGCTCCACCCCACATCTTCTCTGTCTCTGACAACGCTTATCAGTTCATGCTTACTGATAGGGAGAACCAGTCT
GTCTTGATCACTGGAGAATCTGGTGCTGGAAAGACTGTGAACACCAAGCGTGTCATCCAGTACTTTGCCACAATCTCA
GTTGGGGGAGACAAGAAGAAGGAACCAAGCAAAATGCAGGGGTCACTGGAGGATCAGATTATTGCAGCCAATCCCCTG
CTGGAGGCCTATGGTAATGCCAAAACTGTGAGGAATGACAACTCTTCTCGTTTCGGTAAATTCATCAGGATCCATTTC
GGCACAACTGGCAAACTGTCTAGTGCTGATATTGAGACATATCTGCTGGAGAAGTCAAGAG

>Sequ10077EST2
CGGAGCTATTGCACTTCAGGAGATTTTGACAGCATCCAGCCGAAAATGTTTGGTATGGTGTTGGAGAAAATAGTTGTT
CCAGAGGTTCAGAAGGTGTCTGGAGCAGTTGAGAAGAAGATCTGTGCTGTTGGCATTACAAAAGTCCTTACTGAGTGT
CCTGCAATGATGGACACGGAGTACACTAAAATCTGGACCCCACTGCTCCAGGCCCTCATTGGTCTTTTTGAGTTACCA
GAAGATGACAGCATCCCAGATGATGAGCACTTCATCGACATTGAGGACACACCAGGCTACCAGACAGCATTCTCACAG
CTGGCCTTTGCTGGGAAGAACGAGCATGACCCGATCGGAGACGCCGTTGGCAACCCCAAGATTCTGCTGGCACAATCA
CTCCACAAGCTTTCTACTGCCTTTCCAGGAAGGGTTCCTTCGATGCTGAGCACCAGTCTGAATGCAGAAGCCCTCCAG
TTCTTGCAGGGTTACTTACAGGCAGCCAGTGTGCAGTTGGTTTGAAACGGATGTGATGCTCTTCACATAATGAGACCG
CAATACACTGCAGGAGTAGCCACAGCGGCCTCGGGGTCACATGACAGACCTGCTTCAGCTGAACTTGAGTCTTTACAG
ATTTGGTGTGACTTGACAGTTATGTGATGGATTTTGACAGAGGAAATAATCATTTTGCCTGAATTTAGGGTGTGTTCT
CTGATGGAAGTTTTTTAAGGGTTTTTAAAGACATGACCAAAAGTTGACATAACTGAATTACTGTTTTGTGTTTGGGCC
TTTTGTGTGCTGTGTAGCAAAGGCTAACAAGGATTCTCAAGTTCTGATACAATCAGTTTATTTCAACAACACCACCAA
CAACAACAACAGCAGCAGCAGAGATATATTTCAACAGTATTGGGGAACGCTGAACCTTTGATTCATATGGGAAAGATT
TCTTTTGCGTCTTTTTTCCTGGTATGTTTGAATTTCCTTTTTACGGTATGGAGACTGTAGTCTGTGGCTCAAACATAA
TGCTTAATATTTTTCTGTTGCATGTTTCCTGTAGATTTGTTTATGTCTAGTCTCTGTTTCT

>Sequ10100SNP2
GCAAGTAAACAGAACAAAAAGGCCTTCAATCAGAAAGGTGTTCATATTCAAACTACGTGAATCCAGTTTCGAATCTGG
CCTGGTGCCAGCTATATTTATATCTATTTATGTTGTTAATAAGCATAATCATTAAATTATCCACAGGCCAGGTATTTT
TAAGTATTTATCTAAATATTGCTAAATTGGGTTGATCCACTAACTGTCACAACATTCCTTTCTGCATATTTGGAGGCA
CTTGAAGGGCTTGTTTATCTCTCTTTCCTCAGAAGCTTAGTGATCAATACCTCTGTAGATGACCAGTCTCAGCTGTAA
GAGAATAGACGGGAACGAACAAACTACAGCAGAAGTTGTGTAACCAGGCACATATATTTTTGTCAGCACTTGGTACAT
GGAGGTCATCTGTATTTGAGCTGAATGTTTATTGATGGTGGAGACTGGGCCTAATGACGATTCTCCATGAAGCTTGAA
TGGGAGCTGAAAAGGGCCCCAGTGCTCCACATCAACCCTTGACTCCTCCTCAATCATGCACCAAGGATGAAGGTTTTC
TTCTTTATTTCCTGTGTGATGGCTGAAGTGGCATAATGACAGAGAATTGCCAAAGCAAGTCAGTTTCAGAGCTTAAAA
AAATCCTGCTGTATGATGGTATGTTCAGGTAATTGTATGTCTCAAGTTTCCATGGGTGTGTGACAGTGGGAGGATGTG
GGGGTTTTGATTTCGACTCTGATGTTGAGGAGTTATTAAAGAAATTATAAGCTTTGGTTGTTTTGATTTCCTTTGTAT
GTTGCACTCAACAACATTAATACACTGATTATTACTATCTCTATGATTAACTTGGCTCAAGTAATATAATGTCTGACA
ACACTGATGATCTGTTCAATGATAAGTGTGATGCTGTTCACTCTATAGACTGATTACATGTAAAATAAAAC

>Sequ10260EST2
GCAAATGTTGCTGCCTCCGACTTACAAGGTCCAGCTTCGGCTGGGCCGAGTCCGGACAGCCCGACGGTCCAGTACAGT
ATGATCCTGGACCACCTTATCGGGGACAAGAGGCCCGTAAAGGACCTGAACCCGGCCGTTATGGGGGGGCTGCCTGTG
CCTGCGAAAAGCGACGAGCAGAAGATGATCGAGAGGGGAATGGAGAGCTGCGCCTTCAAGTCTGTCCTGGCCTGTGTG
GGAGGCTTCGTTCTCGGAGGAGCCTTCGGTGTCTTCACAGCTGGCATCGATACCAATGTTGGCTTCGACCCCAAAGAC
CCCCTGAGAACTCCAACAGCACGAGAGGTCCTCAAAGACATGGGCCAGAGGGGGATGTCCTACGCCAAGAACTTTGCC
ATCGTGGGCGCCATGTTCTCCTGTACAGAGTGCATCATAGAATCACACCGAGGCGTATCTGACTGGAAGAACGCAGTG
TACAGCGGCTGTGTAACCGGAGGAGCAATTGGATTTCGTGCTGGTCTGAAGGCGGGGGTCCTGGGGTGTGGAGGCTTT
GCTGCATTCTCCGCTGCCATTGAATATTATTTGCGGTGAGCCACGAGAGAGTGGCTCTGTGGACAAGAACGTAATCGA
TGCACATCTGGAAGAGCCGCGAAGACTCACGCTGTGAGAAATCTGACTTTGTGAATATTTACCTGCCCATGGAGGATT
GCTGCAAAAGCAGAACAGATGTATTTGGAGGAAGTGACAGGTTCCTGTTCGTGAACTGAAACAGACCTGTCTGTATTT
TCTTCCTTTGTCTCATTACTTTTGTGTTGTGGACAGTTTGAACAAAGCAAACCGACTTCATGTGGAGACTGAAGAAAC
TCAGCAGAGTGAGTCCTCTTCTGTGTAACGATGAGATGAGAATCACTGTCGCTCTGAGTCAGGACCACCTCGCATCAG
ACTCAGATTAACACTGTTCTTGTTGTAGAACATTGGTATGATGTGTCAGTAACTAATCCACGTCAGGATTATTTATAT
TAAAGATGTTTTTTTTGTAACTTGAACATTCAGAACCTTTTTCTGATGTAAGTTTACATGTGTCCATTGACCACAACA
TTGTTATCGCTTATTAATTACAGTACTGATTCCAGGGCTGCACGATACGAC

>Sequ10270EST2
AAGACAGAAATCACAGATAAGCTGCGTGCCGAGATCAACAAGGTTGTGAACCGCTACATTAACCAAGGCGTAGCTGAG
CTCGTACCTGGTGTGCTATTTGTGGACGAGGTGCACATGCTGGACATAGAATGCTTCACCTACCTTCACCGGGCACTC
GAGAGCACCATCGCCCCCATTGTTGTGTTTGCTTCTAACAGGGGAAACTGTTTAATCAGGGGGACAGAAGACATCAGC
TCCCCACATGGGATTCCTCTGGATTTACTGGACAGAGTAATGATAATCCGCACCATGTTGTACACGCCACAGGAGATG
AAGCAGATCATCAAGATCCGTGCACAGACTGAGGGGATCAATATCAGCGAGGAGGCACTCACACACCTGGCAGAGATT
GGCACAAAGACAACACTCAGGTACGCTGTGCAGCTGCTGACGCCAGCCAGTCTGCTGGGCCGTGTTCAGGGAAAAGAG
ACCGTGGAGAGGGAGCAGGTGGAGGAAATCAACGAGCTGTTCTACGACGCCAAGTCTTCCGCCAAAATCCTCCAAGAC
CAACATCACAAGTTTATGAAATAAAACTGGCTTTCTATTGTTTCTTTTACATGTTATCAACCAGTTGTTACATGGTTC
ACTTTATGTCTTCATTCTTGTTTCATGTTTTGTTTCTTTGCATTTTTTTTCCCAAAATGTCATTCTCCTCCACAGCTC
ATTCTAGTGTCCTTGCTTCTGCCACATTTACTGTAGTAGAACTAATAAATTCTAAGTTTTATTCATTCCGTTTGTTGA
TGAAAAAATTTTTTGAATAAAAAGAAAACAACAAAAAAAAAAAAAAACTAGAGACT

>Sequ10296EST2
GACTGTGCTCATGCCGACAGCATCAGCGCCCAGTTTGTGCAGCATACGGCATTCAGCAATGGTCTCAAACGAGGGCCC
GCCCAGCACGCAGTAGACGCCCTCCTTCAGGAAGTCTCCAAAGCCGAGCTCCTGTCCCACATCCATGGCCATCTGCTG

CAGATCCCTGTCGTATGCGTCGGACATGCAGGGGAAACGTACGCCGAACCTCTCATCGTTGGGTCCGGACAGAGGATT
GTTGCCAGCGAAGCCGGGCATGTTGAGGTGGTCTTTAATGATCATGATGTCTCCCACTTTAAAGTCCTGATTGAGGCC
TCCAGCTGCGTTGGTCAGCATCACGGTCTCCACACCGAGCAGCTTGAAGATTCGCATGGGCAGTGTAATCTTCTGGAT
TGGGTAGCCTTCATACAGGTGGAAGCGCCCCTGCATGCAAACACACGGCCTCCCCTTCAGTGTGCCAAACACTAGCCG
TCCTGCATGTCCATGCACAGTGCTCTGTGGAAAGCTGGGGATGTCCTTGTAGTTGAAGGCCACCTGGTCCTTTAACAT
GTCAGCCAGCCCTCCGAGCCCCGACCCGCACACGATGCCCACTGCGGGTCGGACGTCTGTCTGGGCCAGCAGCCAATC
TGCAATGGCCTTGCAGTCCTCGTAGCTGTAACCTTTGTTTGTCTCTGGAAACATTGCGGAAGATTAAATAAGAGTCTG
ATGAGGCAAAGAAAAAGTCAAATGTGTGACAACACCCTTTTTAAGCGAAACCAAAAAAATAAACTAGTTACCTACG

>Sequ10379EST2
AGTGGTTTGCGTTTATTTAGTTTTATTTACTTACATATTCGCCTTACAAGCTCAAACACTTTAATCATGATTATGAAA
TCATTAGTTATGATCCTTACAAAACAATTTAAAAGCTAATTTTTTTTCATGGTTAGTCAATGTGATGTGACATTGACT
TGTCAAGCTTATTAATGCTGATATGTTGATCACGGCAGCCACGTTTTCTGGATTCGTCTCTGTGCCTTCACAGCTTCG
CTTGCTGTTTCCTGCGTACTGTCCCTGTGATGTTCCCTGAGGGGTTATCCAGGGGAAGGAGCACTTCAGCTTTGTTGG
TGAGGCCCATTTTTGTCATGTCTATGGTGAAGTAATGCTGGTTGGGCATGACGATCTCTATTTCATCCACCTCAGGAA
CTCTGTCCAGGACCAGAAGCTGTGTGTCATAAAGGGTCTTCTGCACAGAAGGTGAGAACTCTCCACGACCGTAGGGAC
CAGCAAACTTCTCAATAATTGTCTCCTTCACGCACTTCCATGCAGCGTCAAAGTTGACATCCTGAACCTTGTTGTAGC
GCCACCTAGCATAGATAGAGGTGCAGAAACACCTGTCCTTGGTCTCTTGCAGAGTAGTGAAGCGGTCTCGGAGGAAAC
CCTCAAAACCAGACTGGGTGGTCTTCAGCACCGTCATGTTCTTCACCCCAGTATGAACCACCGGGATGCCATGGAGAT
TCTGTTCAACATCGCAGAAGCGACAAGCCTCTGGGTTGAAGATGAAAGCATGAGCGTGTTCTACCCCATTCTTCTCCA
GCCTTTTCCACGGAGCCTCCTCCATGTAAACCTTGGCCCTCAGCACATGGTTGAAAGAGGTCAGGAAGTGACTACAGA
TATCCAGGGAAAACTGCTCGATGGTCTTTACCCCCCTGAGCTTGGCCAAGGCATGGACTGTGTTCTTGATGGTGTCGG
TGGGGATGATGTCTGAGTTGTCTCCCGTTAGATAATCCTTGCGTGATTTGAGTGTAAGCTCCACGTCGGCTTTGAGCT
CGATGATGGAGTGGTGGCTTCCCTGTCTCCTGATGACCAGCACTTTCACCGTGTTCTTGCCGTAGCCTGTTCGCACAA
ACTCCACATTCTTTTCTGAAGCAGTTGCCATAGTGTCTTCTGTTCATACACCTGCTGTCTGACTGAACTCAGCCCCTG
TGCCGTTTCCAGCAAAAGGCC

>Sequ10451EST2, Sequ10451SNP2
GATCAATAGAAGTAAAGCTTAAGGCTCCTCTTTCAGTCTCTAGGCCATGCCCTCTGTGTGACATCACTCCTTCGGGCC
GTTGCTGTGATGACGTCACAGCTTGGCTTGGCGGCGGTCTCTGATGGCATACAGGATGGCGTGAGCTTGGTCGAAGGC
GTCACCAACGGCCGATTGGACTCTGCTCCTCCAGCTCTGCAGCTGAGGACGATCCAGCAGGATGTCCCTGCCACTGCC
CACCGGCTGCATGAGCTCGCAGACAGCGAGCAGGTCGGCCACAGTGATGTCATCGCCGCAGAGGAAAGGCTGTCTGCG
CAGGAACATGGACTCCAGTTTCCCCAGAGTGTCGTCGAGCTGAGAGAGCGCACGAACCAAACGCACCTCCTCCACCGG
AGAACCCGACTGAGCCGGGAGGAGCACCTCCAGTATAAAGACTTTAGCAGCGTGTGGTCGTGTGTTACTGTGATGCCA
GGCTGTGTATTCGTCCACTCGGGCTCGTCTCTCCTGTTGCCGTGGATACCAGTGCTCTGGGACGTAGTACTTGGTGGC
CAGGTACTTCAAAATGGCATCGCTCTCAGTGAGGACGAAGCCGTCGTCCACCATGACAGGGACCTTCTGCATGGGGTT
CAGCCGGGTGAAGTCTGGAGTCCTGTTCTCTCCTTTCCTCAGAGCCACGGTGTGGACTCTATGCGGGATTTTGGCGCA
GTTCAGTAGGATGTGCACCGCCCGGCAGGGCTGGGACAGGAGGTCCAGATACACCTCCACCAGCCGGCCCGTTGCCAT
GGAGACTGAGCTGGTCTTTAACGGTTAAAACGGTAAATAATTGGAGGATATCACCAACAGCTGTTGTTCCTCTGACAG
TCTGTGCGCCCCCCCCCATCGTACTCT

>Sequ10504EST2
TTTCAGCTGAGCCTCCAGTCTCTCAAGACTCAGTATCTTCTTGCTCTCAGCATCGAGCTGCTCCTGGATGTGGCTGTG
GAAGAGGCGGAGAAAGCGGAGGGGCTGTGGGGGCCTTCACCTTCCAGTAGAGCTTCTCCATGATGATCTTCTCCATCC
TCATCATATCAGACACCGTGAAGCGATTCTGACTAATGCGGATCAGGTCATTGGCTAGAGGCACGTTCTTCTCCTCTT
CTGACGACTTCACAGCAATGTAGAAGCAGCACAGGCCCACGCAGGACAGGTGCTTAGGTTGAATCTTCATTACTGAGA
GGAAGCGATCCAGCAAGCTAATGGCCAGTGAGAAGGTCTCTGAGCTGAAACCAAAGAACCTGGTCAGAGAGAGCAGGT
CCTTCACCTCCAGCTCCCTCAGTCTGGCAGTCATCCTGAGGCCATTGTCCTGGGCGCTTTCGATGAGCCTCAAACCGC
TCAGCTTTGGTTGGTATCGGCCCTCCAGATCTGTTAAGGCCTTCAGCTGAACTGCAAAGGGCTGTGCTCCAGGTCCTG
CGACTGTATCAATCATCTTGAATCAAGTTACGCTGCCTTTATGAAAAATCGGCAGTGGGGAAAAGCCTCCACGCGCTT
TAAATAAGCGAGTGAAAAGAGGTTTAAAACACAAAAAAGTGTAAGTATGAACTTTCGCTAAAGAAAGTTTTAAGCATA
GGTTAATTTCTGCCTATGCACACCCCCCTACCCTTGGCGTAAGGCGATGGGGAAAAG

>Sequ10559EST2
AATTGTTTTAATTATTTCACAGAGCTTGGTTTTAATAATAAATATAACTCACAACACAGTAGTCAAAAGGAAGCAATG
AGGATCGAACAAGCAACCATTTCATCTTCATACCTTCAAACCAGCAGCTTCTGGTGTTAGATAAATACTGTACTTTAG
AAAAACCCAAGAATCTTAGCTGTATGTCCACAATCAACGATATATAGTGACTTGATAGTTGTTTCAACCCAGCTGGAC
GTGCACTGTGTTGCAGGGATGTACTGTTGGAGTTGGTGAGGTGCTAGGAGCAGTCAAGGACCAGGACTGGGAGTTGGC
GTGGAAGCGATGGCATGAACACAACAAACTGAGGATCTTCTGTGCTCCATCAGTAAACTTCCAGGTTAATGCCAATAT
GTGAGGGGTTAAGGTAGGAAAGGAAGTTCTCTTTTTATTCTGGAGCTGCTTAGAAAAGACCGCAATCCTTAAGGTTTT
CTTTGATGATGATGTCTGTCACAGCGTTGAACACAATCTCGACGTTCTTTGTGTCTGTGGCACAGGTCAAGTGGGAGT
AGATTTCTTTGACACCCTTCTTCATGTTCAGTTCCAAGAACTGTGTCTTGATGTAGTTGCTGGCGTCGTCATACGTGT
TGGGGCCATCATAGTCTGGGAAGCAGATACTCAGATGGACTTTCTTGATCTTCTCTTCAAAGAGATCCTTCTTGTTGA
GGAAAAGCACGATGGAGGTCAGTGCAAAGAATCTGTGGTTGCAGATACTGTTGAATAGATGGAGGGACTCGTGCATGC
GGTTCACTTCGTCGTCCTCTACGAGCACCATGTCGTATGCACTGAGAGCTCCGCAGAAGATGATGCAGGTCACACCCT
CAAAACAGTGGATCCACTTCTTT

>Sequ10660EST2, Sequ10660SNP2
GACAGCACGCCAAATAATACTCTAGTCATTTCTCCAAACAGCTCTGTGTGAAGGCCCGGTCTGAGCTCTCTGCAAAAG
GGGTTTTTTAAGAAGCGAATAAAGTTAGATTGAGGTTTGGCTGTAGGACTTCAAACAAATGAGAATTTGTGTTGTCAG

AGCAGGGTTTTGTTTCTCTTTGACGCTTCACACCTTGAGACAAAAAGAAACTGAACCTGGCTGTGCCATGTTGGTAAC
CTTTGTTTAACAGACAAGTTTGAGGTGATGAAATAAACAGCAAAAGGACAGGCTTGTTCAGATCAACTGCCAAAAGAA
AAGTGCCACACAGAAAAGACAGAACCTAGTCGCCTATTCAGTCCTGTACGGCACAAACTCTGAACAAGCCTCAACAGA
ACAACGCAGATCTAGAGTTGAAGCCCTTGATAAAACAGGGGAACATATAAAAAACAAAACAGACATTATCACCACTTT
TCTTTCCCCTGATGGTAGGTAGCTACTGAATATTTGTAGACCTACTCAGTTATGAGTGAGAGACCTTCCTCTCGTCTT
TTTACATCTCTACGCCTGGCCTTAGATGTGGTTTCATTACGTGTGAATGTACATATAGATTTTTTTCCAATTATTTAT
TAAAATTCATCTCTAATTAAACCCAAAAGGAAGGTCAGAAAACAGAGTGAGGGAGAGAGAGTATGAAAAACGAAGACA
AAAAAGAAAAAAAAAGAGGGAGAATAGCATTGACGAGGCAATATCCCCTCCTTTTCCCCAGGTCCAGTGCATCTCGTT
TTGCCCCTGCAGTGGCTTATTTACCGAAGGCTTTGATGCCAATTGCAGCTTCTTCATCCATTGCATTTAGAACGGTGA
TCAGGATCTCCTCTCCGGCATCGTGCTTGCTCTCTATCTCTTTTCCCAGGTCTCCCTCAGGAATCTTGAGGTCCTCTC
TCACCTCCCCGCTGTCCTGGAGCAGGGACAGGTAGCCATCTTGGATCCCAATGAGCTGGTAATCCATTCTCTTGATGT
TGGGGACGTCCATGTTGTGGGTGGAGGGACAAATATCTTCATACTTCTTACCAGAGAAGATGTCGATACCAACCAAGT
GCACCCTAGCGTGGCCGTGCTTGCCGGTCTTGGAGGTGGACATCTCAACGATCTTGCAGGGTCGACCTTTCAGCACAC
AAAGCCATTTTTGCGCAGGG

>Sequ10762EST2
TGTTATCTCATCTTCATTTATTTAAGAATATCAAGAATCCATCAAGGTTGGAGATAAATAAATGGCTTTCACCTCAAC
TGACCTTCTGTTTTTTGATTTATTTGCACTTCAGGCCATCTTGGCTTGATAAGAGGTCCTCATTAAATACTTAAAAAG
ATTACAATGGTAACAGAAAGCTGCAGGTTTACTATACAAGATATTCTAGACTGATGAAATAAACCTTTAAAGCTGCGT
CAGGGTCTGATGGCCGACTTCAGCTTCGGGAAGAGTCGGAGAGCGTTCTGCGTCGTCACCTCCATCACCTTCTCCAGC
GACACTCCTTTAATCTTACTGATGTACTCAGCAGAGATACAGATGTTTTTTGGCTCATTTCTCACCTGCTTTTCTGGA
CCCAGAGCAGGTGAATCTGTTTCCAGGCAGATGTTCTCTAATGGTAACTGTTTCACAAGTTTCTGCTGTTCGCTCCGT
ATTATGGATGGTGGGATAGAGAAAGAAATATCCAGCCTTCACTCCCTCCATGGCAACAGACGGTTTCCCATCGAAAGC
ATGAAGCAGGACTTTTTCGACACCTTGCTTCTTTGAGGAGGTTGATCGTGGGTCTTCCTGCAGACCGTAAATGGACAT
TTAGAGGAAGATCCAGCTCCTTGGCTATCTGTGCTTGACGAATGAGGACCTGCCTCTGGCTCTCTGTATCAGTGTCAT
TGCTGACATATCTGGGTGTAAAATCCAAACCGACCTCCCCAATAGCAACTAGGTGGTCTTTGTATTTCTCTATGAGGG
GTAAAGCAGCATCGAGATCCTGGAGAGAAGCACCTCTCTGCTGCTCTGGGGAAACTTCTTGAACAGGGTGGACTCCTA
AGCAGGGGAAAATAAAACCTGGAAACCTTTCTGACAACGCAATAATCTTGTCAAATTCCCCAGCATGCTCTGCGACGG
CTAACAGCGCCAACAATCCAGCCTTTTTTGAATTCTCAACCACCTCCTCTATGTCCTTGTCAAAATCCCCCGCAGAGA
TATGACAGTGACAGTCAACGTAGCCATGCATGTGGACAAT

>Sequ10772EST2
CTGCCAGAGACTGGCTCCTGAAGACGGCCAATGACAAATCTGCTGTCGCCAAACATTTCGTGGCTCTGTCCACCAACG
CAGCTAAAGTCAAGGATTTTGGCATCGACACAGCGAACATGTTCGAGTTCTGGGATTGGGTTGGAGGTCGTTACTCAC
TGTGGTCAGCCATCGGTCTGTCCATCGCTCTGCACGTAGGCTTTGAGAACTTCGAGCAGCTGCTGGCGGGAGCTCACT
GGATGGACAACCATTTCCGCAGCGCCCCCCTGGAGAAGAACGTCCCCGTCCTATTGGCTCTTCTCGGAGTCTGGTACA
TCAACTTCTTCCAGGCTGAGACTCACGCCATGCTGCCCTACGACCAGTACATGCACCGCTTCGCCGCCTACTTCCAAC
AGGGCGACATGGAGTCAAACGGGAAGTACATCACCAAAGACGGCGCCCGCGTCAACTACCACACCGGCCCCATCGTGT
GGGGGGAGCCGGGGACCAACGGACAGCACGCCTTCTACCAGCTGATCCACCAAGGTACTCGTATGATTCCTGCTGACT
TCCTCATTCCTGCCCAGTCTCAGCATCCAATCAGAGACAACCTCCACCACAAGATCCTGGTGGCGAACTTCCTGGCTC
AGACAGAAGCTCTGATGAAGGGAAAGACTTCGGACGAGGCTCGTAAGGAGCTGGAGGCCGGCGGCCTGAAGAGGGACG
CTCTGGAGAAGCTGCTGCCTCACAAGGTCTTCCAGGGAAACAAGCCGAGCAACTCCATCGTTTTTAAGAAGCTGACTC
CGTTCATACTGGGAGCTCTGGTCGCCATGTACGAACACAAGATCTTTGTTCAGGGCATCATGTGGGACATCAACAGCT
ACGACCAGTGGGGGTGGAGCTGGGGAAGCAGCTGGCGAAGAAGATCGAGCCGGAGCTGCAGGACGCCTCGGAGGTCAC
CTCCCACGACTCGTCCACCAACGGCCTCATCAGCTTCCTGAAGAAGAACTTCGCCTGAGTCCTGACTCCTCCTCCTGC
TCCTCTGCTGGACCCACCC

>Sequ10812SNP2
TGCTGCCGATGGAGCTCACAGCCAACCTTAAAATAGTTCTTAGAGACAACAGGTAGTGATACATCACATTTGTAAAAG
CAAGACTGAAAAAAAAAAAGGTCTCACGGTTCCGACACAAGCTGAGAAGACTGTCCGAGTTTGAAGTAAAACACAAAAA
GCATGTAAGCCAACCACAGGAACCATGGGTGAGTGTCAGGGCTGGCACATCAACACATTCAGAGAACAGAGTTTAAGA
CAAACTGGCAGAAAGAGCTGATATACAACAATGAGCTTCAGCCATGATTCCTGCAAAGTGCATTAGCATGGAAGCAAT
AATAATCACTGCAATACACATGGGAACAAAGAAGCTTTTACCAATGCTGCCATCAGGTACAACAATTCAGAGAGGCTT
TATAAAGTAGCAAGGTACATACATTTGCTACCAAAAGCTGTCCAATTCTTTCTGAATATCCTTTGTAGTTTAACACTG
AACTGTTGTCTACAAAAAGATGACTAAAGGCTGCAGCAGAGTAAAAGTCGTACAGTTCTCTACCTGCTTTGGTTCAGT
CAGCGAGCAGCTGAACACAGTGTAGCATTTAGCAGCTGAAGAGCCAAAACCAGAGCTGACAGGAGAGTGAGTATTGGA
CTTACATTCATCATTTGGACACATCAAAATGAATGAAACATAAAAACACTTAATGCAGCTTTTATTTCTGTGTTTCTC
AGAGTGAAGGCAGGAGCTAAAGGTCACTGTTCAGGCGGTTCTCCGGAGCCCGTCTAACTCCTACAAAGTCAAGGAACC
GACGCTTTGCACGACCGGGGTTAATTCATTGAATGAAATTTGAAGCAGAGAAGCAAGAAGTGTGGAATGTGGTGAGTT
TTGTAATCTTCTTAACAAGAACTTTGTGAGAGTTCACCACATTGTGGTCACAACATTAAACCTGGAGAGAAACAGCAG
AGGTACAGTCCGGCTTTTGGGACGTAGACTTGTCTGCAGTCATACCCGGAGTCAAGAGGACACAGATCGGTTTCATCT
GTGTGTGTCGAGGACACAGATATGTCCAGGATATGGTTAGTCTCTCAAACTATTATTAGATAGCTAGCTACTTACAAA
TATATCCAAGTGTGTTGTCTTTACAT

>Sequ10833SNP2
AATGCACTGGAAACTTCAGGAGGAATCTCCATTGATAGATGATAGATGATAGATTGATAGTATTTTTTTTTTTTTCAA
CCTCTTAACGTTTAATATGCCATATACATTGTAGGGAAATTTGTTGAAAAAGTGGAAGCAAATCTCATGTACAGTTTA
TTTAAAACTGTATTGCGACTAGTCAGTTGGTAAGAATCTTTAATCTGGTGACTCCTGACTATAAATCTGTCCTCACAG
TTATTGCTGGGGCAGCATGATACATTAATGGAAGTTGTACTATAATTAGTGACTGGATAACTCAAAATCATTGTGTCA
GATCAGATTAAATTCTACTAAAAACAAGTCAGTCACAAGAAGGATAGGATAATATACATGACCAGAAAATGCTGAAGG

TGGATCTACTTGGTTGATAACACAACTACTGAAGATGTGTCCTTCAGTGATGTCACTTACGTTTCTATTTTTTTTTAG
TTTTAGTTTGCTCTCTTGATGGAAGTTGTTTTCTGGTGAACGTCCAATATTACACAGTCCCAGAAGTTCATACTCCCC
ACCAGCATTTAGAGTTTTCTTTTACTTCCTGACTGAACAGAATCATGCTTACCTCAAAAAGAGCACATGCTTGGGTCC
ATAATGTAGCCATTAGTGTCTGTGAATGACTGCTCAGGTTCTCCCGTGCAGACTGAGAACTAGTTATAAAACTGAATT
GCCCAATAGCTATACATATATGCTAAACATCACAGAAGAGGTGGTGTGTTAGCATTGTCACCGTTCTTGACCTCAACA
TGCCCTGTGGTGCCGATTCTTGCTGAGATGTTGGCTCCATTTTTTTTCCCCCATCAGAACAGGAAGGGGTTATGGACT
CTGTATTAGAAGGGTGGGTGAGTTTTGTGCTACCGCTGCAGCACTCTGGATTATTTGAAGGTGGAACGGAAAAATACA
AACAAAGCCAAGAAAGGAAGACACAAAAAAATGTAGTTTCTAAAACTGTTAATAAAAAATCCTTTTTTTTTAGACTAA
AAAAAAAAAAAAACCGACCTACCCTAATAGT

>Sequ10925EST2
CCGACATTATTTAGGAGTGTGTGACATGTTCTGCGATGAATATTTAATAACAATTGCAAAGACTTTTTTTTTCACTAT
TTCTTCCCTCTTCCCTTTCCTCTCCCTCTTGTGCTTTTCCCTCTGTGGCCTCGTCGGCTGCCTTCAAAGACAGATCGG
GCTGTAACCATAGCAACTTCACAGCCACCATCGCTGTCCTCGCCGGAGCTGCTGCTGCCCCCGACCCTTTGAGGT
AGACTCTTAGTAGACTGAGGGACGACCTTCACTGAGAGGGACTCCTGGCTGGTGCTGCTCACATCCTTGAGAGGTGTA
GGCGGAGATTCACCAATCACCTCCGACCCCAGAAACCCTCCTCCTGCCACAGGATTCTCCTCCTCTTTCTCCCCTTCT
CCTTTCTTTGCATTCTTGTTTGCCGCCTTCCCTCTCTTGGGTTTGACTGGGGAAGGCTTTTCCTCCTCACTGTCCTCC
TCCCCTCCCTCTCTCTCTTTTCTCTTCAGGCAGGTGACTGCTCGGCGGAGTCGCTGGCTGCGGATCGCCTGCTTTTCC
TGTTGCTCCATGCGGAAGAATGAGTCGATCCGCAGCTGAGTCTGCTGGGCGTTGAGCTGCTTGATCACAGGCTGAAGA
GTCTCCTCTGTTTTCCGACTGCTCCAGCCGAAACGACTGAGACAGAACTCTTTGATCATGTCGAGTTGTGGGCGTCCC
CAGCAGAAGGAGCTGTCTGACTGGTCCACAGCCGGTTCCAGGTAAGCCTGAGCCACCACAGGGTTGGGGAAACCAGGC
TGCAGTTTTAGGTCCCTCAGTTTTTTCTTCACCTTCGTGTCCCGAGGATCACCCATCAGACGCTTTTTCTCCTGAGCC
TCTGACCACCATTTACTAAACTGAGTTAGTGGCTCCAACCCTGGCCCTGGGAACTCATTCAGAATCTCCATGCCAGTC
ACATATCCGACCCCTGGCACACCCTCTGTGTAGTCACTTCCAAGCAGGTAAGCCAGATTTATCAGTTTCAGTCCTGTC
CAGACCCAGTTGGTTCTGCAGGTCACTGTACTGGTAGTGTTCGACATATTTGTTCTGGCTGAAGAAATTTTTATACAC
ATGTCGCCCTCCGAACAGCCACACATCTGAGTCGTCTGTGATGGTCCCATGAGTTTGGTCGGTGCGGTCGAGAGCTGC
ACACTGAGACTCGGCCTCCGTCGGCGCCACCAGGAACGGCACGCCAAACAGACGCAGGAGCTCCTGGCTCTCCAGGTA
CATCTGTCCGGTGACTGTGTTCGCCATCCTCTCCTGCTGCTGTTTCTGCTCCCTCAGGTTGC

>Sequ11009EST3
TGGAGGTGATCCATTGTTGATGAGAGCTCAGATCTTTCACCTCATCTGATCTGCTCTGCTCATCAGCACTCCAGTCCA
GTAGGTGGGGTAAAACCAAGTTTGCCAACCGCCGCGAATGCAAAGAAGAAGAAGAAGACGAACAGGAAGTGAGGAAGT
GAGGAAGTAAGGAAGCGTAGACCGCACAGCTGAACTGCAGATATCTGCTGCCCTGAAAAATCCTGTGTTTCTCTGAAC
AACCACTGCTGCGTCCAAACATGTCCAAAAACACGGTGTCCGACCGCTTCAGGAAAGTGGACGTGGATGAATACGACG
AGAACAAGTTCGTGGACGAGGACGACGGAGGAGAGAACCAGGGAGGTCCGGACGAAGCAGAAGTCGACGGTCTGCTCA
GACAGGGGAACATAAACGGTGCTCTCCACGCTGTGCTGAAGAACCCTCCAATCAACACCAAGAACCAGAATGCCAAGG
AGCGAGCAGAGCAGCTGGTGGTCAAAGTGCTGAGCAGCTTTAAGAGCAGTGACATAGAGAAAGCTGTGGGCTCTCTGG
ACAAAGCGGGAGTGGACCTGCTGATGAAATACATCTACAGAGGCTTCGAGAAACCATCGGACAACAGCAGCGCTGTGC
TGCTGCAGTGGCACGAAAAGGCCCTGGCTGTGGGAGGGGTGGGCTCCATAGTAAGAGTCCTGACCGCCAGGAAGACCG
TCTGATGGTCCAGGACCCAACAGATTCCATCTGTGAATGGACATTAGACAAGAATATGGCCGAACACAAAGTCACCGG
AGCCGACGACAGAGACGCGTCTCTTCTTCTTCTGCTGCTGCTTTTGGACATGATCCCTGTTTGTTTTTGTACTGGAAC
TGAGTGTCTTCTTGATGCAACTCCTTCCTCTTTTTTCTGCATTCACTATTTTCAGACACATGTCTGGGATTTAGACGT
GCTGGCAGTGAGACAGGGAGGTGCTGGCACCTCACACTATGTTTCCAGCAGTCCACACAGAAGAAACTTAGGATGCTT
CACTCAGACACTTTCTGTCCACAGCGGCTCTTCGCTCCTTTCAAACTGCTTCACAGCTGCTCTGATGCCTCTGCTGGC
TCGCTCTAACATCTCTCCTCCTCCTCTTCTTCCTCTTGTCCTCTTCCCCCTCCTTCCCCTCTGAAGCCTCTGAGTCAT
CCAGGTTGAGGAAATGGAAAAGATTGTTCTGGGGTGGGCTGTCAGACTGGTGACCTGTCCAGGGTGTTTCCCCGCCTT
TGGCCCAGTGCATGCTGGGTTCTTTTTCCCTAATGCTCTAACTTCTGAACATCTGTTCTATTTTTTTTCATTTTAG

>Sequ11010EST3
GTAGCACACGTTTATGTGTAGACGTTCAGCTGCGACGCTCAGTTGATCCTCTCGTTAGCGGACAGTGAAGGTTACCTG
CTTCAACAGTGCTTGAACGGCAACCTTTCTAACTGTCTTCAAACACCACAGGACGGGACTAATAGCTCTGATCACTCA
GATTTCAATCGACAACATCAGATCAAATTTTAATAACAGTAAAACTTCAGCTAGCTCCGTTATCCTAAACAAATTTAG
CTTGACAGCTAGCTAACTGTTTGTTTTGCTAACAACGCAAGCCACAGCTTTTCGACCTCCTCACAGGAAAGAAGACCT
GCCACTGATATTTGAACCATGAGTTCCCAAGTGAGACAGAACTTCCACCAGGACTGCGAGGCTGCAATCAACAGGCAG
ATCAACCTGGAGCTGTACGCCTCCTACGTCTACCTGTCTATGGCATACTACTTTGACCGTGATGACCAGGCATTGCAC
AACTTTGCTAAGTTCTTCCGTAATCAGTCACATGAGGAGCGTGAGCACGCTGAAAAGCTCATGAAACTCCAGAACCAG
AGGGGGGGAAGGATCTTCCTACAGGATGTCAGGAAGCCAGAGAGGGATGAGTGGGGCAGTGGTGTTGAGGCTCTTGAA
TGTGCCCTGCAGCTTGAGAAGAGCGTGAACCAGTCACTGCTGGACCTGCACAAGCTCTGCTCTGATCACAATGACCCA
CATTTGTGTGATTTCATCGAGACGCACTACCTGGACGAGCAGGTGAAGTCCATCAAAGAGCTTGCAGACTGGGTGACC
AACCTGCGCCGCATGGGAGCTCCTCAGAATGGCTTGGCCGAATACTTGTTTGACAAACACACCCTGGGCAAAGAAAGC
AGCTAAATCCCTCCATATCACATTTACAAAAGCTTCTATATGTGTATATTTGTATCTTGCTTCCAAATATTTTCAAAG
CCATCTGCATGGTAGTGGCTAGTCATTAAAACCTCTTATATTACTCATGTTGTTAATGCCATTTTCTCCATATTACAA
AAGTATTTGCCCCTGAGACATTCAAATCCCACTCTTGTATCTCGCACTTCTCTCTCTGCTATCAGCTATGTCTTTGAC
ATTACTATATTTTTGCTGTTTTATGCCAGGAGCTGTTTGTCAAACCAATAAATGGAACCCTCTGGATTCTGATCAGCC
TCTTTCGCAGT

>Sequ11024EST3
GGTCTACTGGCCAATCAGCGGGCGGTAAATATTTCCGTGGAGGCCTTCAACCAGGAGCAGCGGTAGTGTTGTGTGTGT
GTGGCGAGTTAACAACGTTAGTTTTAGGCGTCTTTAGCTTCAATAATGCCGTATGCTAACCAGCCGACGGTGAAAATC
ACAGAACTGACAGACGAAAATGTCAAGTTCGTCATTGAAAACACTGATTTGGCGGTTGCAAATTCTATACGCCGTGTC

TTCATGTCAGAAGTTCCCACTATAGCGATTGATTGGATCCAGATCGATGCCAATTCCTCAGTACTGCATGATGAGTTC
ATTGCGCACAGAGTCGGTCTCATACCTCTCACGAGTGATGACATTGTGGACAAAATGCAGTATTCTAGGGACTGTACC
TGTGATGATTTCTGTCCAGAGTGTTCTGTTGAGTTGACCCTGGATGTTCGATGTACTGAAGATCAGACGCGTCATGTC
ACCTCACGTGACCTTTTATCCAACAACCCCAGAGTCATCCCGGTGACTTCCAGGAGTCGAGACAATGATCCCAATGAC
TACGTTGAACAAGACGACATTTTGCTGGTGAAGCTTCGTAAAGGTCAGGAGCTGCGACTCAGGGCGTACGCAAAGAAA
GGCTTTGGTAAGGAGCACGCCAAGTGGAACCCAACAGCAGGGGTGTCCTTCGAGTATGATCCAGACAACGCACTCAGG
CACACAGTCTACCCACGACCTGAGGAGTGGCCGAAGAGTGAATACTCAGAGATCGAGGAGGATGAGGTTCAGGCTCCC
TACGACCCCAATGGAAAACCAGAAAGGTTCTTTTACAACGTGGAGTCATGTGGCTCCCTGCGACCGCAGACCATTGTC
ATGTCAGCGCTGGCCGTCCTCAAGAAGAAGCTGAGTGACCTGCAGACCCAGCTGAGCCATGAGATCCAGAGCGACGTG
CTCACCATCAACTGAGGACATCTCACTTACAGACCAGGACTTGCCCTCACTCAGCTTTGTAATGATCAGAGGGGCTGA
CTTTAGGATCACCGTTGAGGTCACATGGCACACGTCCCAGTATTGTTTATGGAGTTGATGGATTTTTCCAGCAGCAGC
GATTGTGACATAAAGCGGTTGTGCTAAGGAGGGCAATGCAGGTTCAAGTGTGTGAGAGGGAGCTTGTCTCAGAAATTT
ATTTGTTGCTGTCAGTGTAAATGTTGCCTT

>Sequ11076EST3
GGGCCAGGAGCTGACGGCCGAAAAGCATGGACATTCGTCAGGATGAGCTGGGAGATCTGGTCGACAAAGAAATGGCTG
CAACATCGGCAGCTATTGAGGAGGCAGTCCGCAGGATTGATGAAATGATGAATCAGGCTCGAAAGGACACATCAGGAG
TTAAACTGGAGGTCAATGAGAGAATCCTTTACAGCTGCACAGACCTGATGAAGGCCATCCGCATGCTGATTATAGCAT
CTACAGATTTGCAAAAGGAGATCGTTGAGGGTGGAGGGGTGCAGCCACCATTAAAGAGTTCTACGCCAGAAACTCCCG
CTGGACTGAGGGACTCATCTCTGCTGCCAAGGCTGTGGGCTGGGGAGCCACAGAGATGGTAGAGTCTGCTGATAAGGT
GGTGCTGCACACAGGCAAATATGAGGAGTTGATCGTCTGCTCTCATGAGATTGCTGCTAGCACAGCACAGCTGGTCGC
CGCTTCAAAGGTTAAAGCAGACCGCAACAGTAAGAGGCTGACAGTTCTCCAACAGGCCTCTCGCCGTGTGAATGAAAT
GGCAGCTAATGTAGTGGCCTCAACAAAGACAGGCCAGGAGAACCTGGAGGAAAAAGATACCATGGACTTCTCTGGGAT
GTCCCTCATCAAGTTAAGAAAAGAAGAAATGGAGTCACAGGTGAAAGTGCTGGAATTAGAAAGTCAATTGGAGAACGA
GCGACTGCGTTTGGGCGAGCTGAGGAAGAAGCACTATGACTTGGCTGGAGTTCCTCTAGAGCAGGTTTCTGAGGGGAA
CGGCGAAGCCTCCTCGCTGGTCCATCCTGTAACTATGTCACCAAAACCCAGCAAACCTGCTCTCATGAAGAAACCTGC
GTTGGCCCAGAAACCCCACATTCCACCCAAATTTAACTAAATGCCTGAGCAGAGGATGATAATGCTCCACTACTGGAT
GTTTGGACTGACCAGAGAAAGATCGCCTTTTTGTGGACATACAGTTCCTTTAAGTTGCATGCTGACAATCCTCTGCCT
CATCCTGCATCTCGCTGTTCATCCCTCCTTTGTTTTTCTTTTCTTTATGCCAAAATGGACCAGAAGCCTGTTTTTTTT
TTTTTTAAACCTGCGATGCCTGGAAGGGATATGCTGCCTTTACTCCTCTTTTTAAAAGATACATATGCATGCCATCCA
GGCAGAACCCTTTTTATTTAGAACTTGGCATCAAGAACAAAAAGGTTAATATTATAATTTTAGGCTGTTTGAAAGTTG
TTGTTTTTCTTCTGCATCACACAGATTTTAGACTATTAAGCACTACTGCTTTTAGAATACAAACCTTGATGATGACCA
CAAATCGACAGCATATCTTGTATGTGAATAGGTAGTTTAGTTTTGATCATGCTTTGGACAACCTTTTTGCACCATGTT
TTGGACATTCATTACAGAGCACATTACATTTTTCTTAAGGTACACTGTATGCTATTTATGTTGTGGTGGGCGGTAAAG
GAAACTGGGGAAATGTGTTGTGTGCGTCCATTTTGCAGCAGAAGTTTTACATACCTCTGGCTTTGCCTTCATGATGGA
CAATGTTCCTCGTATGTGCAAAATAGCTTTCTTTCTCAGCAGTACAAGATTGTCTCAGAGGCCTTGAAACGTACTGTA
CTAAAGCATTAAATAATACCTGTAAAGACATACAGTCTGTATAGGAATGTGCAATTCACACCTAGTAAATATCTACAC
TCATCTCTTGAATGTCCCTTTAGAGGAGAACCTGGATATTATGTTAAAGCATATAGACAGACTTAATTCTTGCACACA
AATATCAGATGTAGGTGTCCTTAATTATCAAATACTTCTTCTAGGCTGAATATGAAGATGTAGGGTTAGTTAATGTAC
AATATTAAGGTGTTCACGTCCACTTTGCCTCTGGGAGCTTTTCTTACAGGTTCTTAAAATGCATCTTTTTATGTTCTG
TCCTTTCCTCTGTACGAAATGTCTTTATACACTCAGATCTATTAATATATTTAGTAAAGATATTGGTGGTTTCTCCT
TGTAAGAAGTATAAATGTAAAACAAATGAACAATAAATGTTTGATTTGATGTCCAGT

>Sequ11081EST3
TCCTGCGTGTCTGACCTTCATAGGAGTAACCATGTTCGCCAGGACCAGCGCGTTGGTGTTCTCCCCACAATGTGGGCA
GGTCAGGAACATGGCTACCTTGAAGGACATCACCATTCGGTTGAAGTCCATCAAGAACATCCAGAAAATCACAAAGTC
CATGAAAATGGTGGCCGCTGCCAAGTACGCTCGTGCTGAGAGGCAGCTGAAGCCAGCCCGTGTTTATGGCACCGGTGC
TCTGGCTCTGTACGAGAAGGCTGAAATCAAAGCTCCCGAGGACAAGGCCGCCAAGCATTTGATCGTTGGTGTGACCTC
TGACCGTGGCCTCTGTGGTGCCATCCACTCTGGTGTGGCTAAGGCCATTAAGAGCGAGATCGCCAACCTGACTGGCGC
TGGCAAGGAGGTGATGGTGATCAATGTGGGAGACAAGCTGAGAGGCCTGCTGCACAGAACTCATGGAAAGCACATCAT
GCTGAACTGCAAGGAAATCGGCCGCAAGCCCCCCAGCTTCGGTGACGCCTCCATCATCGCCACCGAGCTGCTCAACTC
TGGATACGAGTTCGACCAGGGCTCCATCATCTTCAACAGATTCAGGTCTGTTATCTCATACAAGACGGACAACAAGCC
TGTGTTCTCCACAGACATCGTTGCCAACTCAGAGAGCATGGGCGTCTATGATGACATCGATGCCGACGTGCTGAGGAA
CTACCAGGAGTTTGCTATGGTCAACATCATCTACCTGGCCCTGAGGGAGTCCTCCACAGCAGAGCAGAGCGCCAGGAT
GACTGCCATGGACAGCGCCAGCAAGAACGCTTCCGAGATGATTGACAAGCTGACCCTCACCTTCAACCGTACCAGACA
GGCCGTCATCACCAAGGAGCTCATTGAGATCATCTCCGGAGCTGCTGCTCTATAAACGGGCCAGGTCATCGTTTCCAT
TGCCTAAAGCTCTTCAAAAAAAGTACTTCGGACAAGTGTTGTCGAGAGTCTAAATGAACATTTGTGCTTCTATTTGTA
AAATATATGGAGAAAATAAACCTCTTACACAGAACCTTCATCCTTTCCTCAGTCCCTGTATCTCATTCCTCACAATAA
AACCTGACGACTGAGAAAAATGAATGTTGATGCTCTTCTCTTCTGAAGGTGTCGCTACTTTTAAATCATCAGTTTGTG
TTTTTGAATTTTCTTCTAGAAAGGAATTTAGTCTCAGACGCTTAAGAGTTGTTCGACGGTGAATTATTCATGTGGATT
GTAAATCTAATTATTCCTCAGTTGTGTTATTAAACAGAGGATGGACAAAATAGCTGAAACACGTTATGTAATCCAAAC
AATAAATAAATAAATAAATGCCACCTCTGGGCTCTACACTGG

>Sequ11087EST3
CACACACACACACACACGAAGGAAGGACAAATAAATACACTTCTTTACCAGCTGTGTTAATACAAAAACTTATAGTGT
TGTAGTCTGAATTTGTTTAAAAGTCATGCTTGATTCCTATGAATGAGTCGGTCCATGTGTTATGTGTTTCATGATGCA
GTAAAATCACATGGTCTCTGCTCCCACACTGAGAGGAGTCGTTAGTGAACTACAGGCTGATGCTCTGGGTTTTTACCT
GCCGTCTGAGATATTATTAAAACAGGACAAAGACGTAGGAGACGATCAGAGTGACACAAAACGAATAAGGCAGTGAGG
GTCTCGAGTCTCTCCCCTCGGACTAATGCTGTCCCCCGTCTAGCACAGATCACATAACAGATACTGACGACACGTGCC
GACGCTCACTTCACAGGGGACCCAGTGCAATCAGGGTAAACAAGGTCACAGGTGGCGTCCAGTGATGCTCCTGGACTC

TGTTATTGTTGTGTTTTCTCTCTGTCACAGGTTCGGCTTGTCGTCTAGTAAACAGGGATGTCCCCCCCCCGACCCCCC
CCCCGGTCGTTTTTAGGAATGACTTCATCTCCTGGAGTCGTGGGCTCGCTCCTCCTCCCCGCTGCAGTGTTAGTTCCA
GTCGCTGTGTAGACGTCTCTGTGTGCGTGAGGCATGTGTTTTCAGTGTGTTTGTGTCTGTGTTGAGGCGTCAGAGGCG
ACATCACAGCTTGGCCTTCAGGTTGTCTGCCAGCTTGTCCAGGAACTCAAAGGTGTTCAAGTAGTCGGCACGAGTCAC
ATTTGGCATTCCTTTGATGCAGATCGCCAAATCCTTGGTCATGAAGCCGGCCTCGATGGTCTCGACGCAAACGGCCTC
CAGCGCCTCAGAGAACACTCGCAGCTCTGCGTTGTTGTCCAGCTTCGCCCGGTGGAGCAGACCCCGCGTCCACGCAAA
GATGGAGGCTATGGGGTTGGTGGAAGTCTCCTTCCCCTGCTGGTGCTGTCTGTAGTGGCGGGTCACTGTGCCGTGGGC
GGCCTCCGACTCCACCGTGCGTCCATCGGGACAGATCAACACGCTGGTCATCATGCCCAGGGAGCCGTAGCCTTGTGC
CACGGAGTCAGACTGGACGTCTCCGTCGTAGTTCTTGCAGGCCCAGATGAAGCCTCCCTCGGACTTCATGGCCTGGGC
CACCATGTCGTCGATCAGACGGTGCTCGTACCAGATGCCTTTGGCCTCAAACTGAGCGCGGTACTCCTTCTCGTAGAT
CTCCTGGAAGATGTCCTTGAAGCGGCCGTCGTACTTCTTCAGGATGGTGTTCTTGGTGCTGAGGTAAAGCGGCCAGGC
TTTAGTCAGAGCCATCTGGAAGGAGCTGTGGGCGAAATCCCTGATGGACTTGTCTGTATTGTACATCCCCAAAGCCAC
ACCCCCTGTGCCCTCGAACTCGTGCACGACGAATTTAACTGGCTCTCCGCTGGTGGGCGTGTAGGTCATCTCCACTTT
CCCGGGCCCGGGCACCACAAAGTCTGTGGCTTTGTACTGGTCTCCATGTGCATGTCTGCCGATGATGATGGGTTTGAG
CCAGCCGGGCACCAGGCGGGGGATGTTCTTACAGATGATGGCCTCTCTGAACACGGTGCCACCCAGGATGTTACGGAT
GGTCCCGTTGGGCGAGCGCCACATCTGCTTCAGCTTGAACTCCTCCACTCGCTTCTCATCTGGAGTGATGGTGGCGCA
CTTGATGCCCACGTTGTAGCGGCGGACCGCCTCCGCCGCATCAACCGTCACCCGGTCGTCCGTGGCGTCTCGGTTCTC
CATGCCCAGGTCGTAGCTGTGCAGGTCCAGCTCCAGGTAGGGGAAGATCAGCTTCTCCTTAATGAGCTCCCAGATGAC
CCGAGTCATTTCATCTCCCTGCATCTCCACCACAGAGCCTGCCTTGATCTTCTGAGACATGTCGGAGCCTTGTTTTAT
TCAAACGTCCACAACAGAAACGGCGAAACGAGATGAGGAGGCGGACCGACGTCGTGAACCGTAGCACAGGTTGACTGG
GCTGGGACTGCCGGAGGAGCAGCGGCAGGAG

>Sequ11088EST3
TTGGAACGCACCTTTTCTACAGGAAGTGACGTACAGCGTTGTATCGGGTCTTCATTTTTGTCGTGTGGTAGCAGCTTC
GAGAACCATCCAAGATGACTACAGCTGCAAGACCAACATTTGAGCCGGCAAGAGGAGGGAGAGGTAAAGGAGAAGGTG
ATCTGAGTGCTCTCTCCAAGCAATATTCCAGCCGAGATCTTCCGGGTCACACTAAGATCAAGTACAGGCAACCTACCC
AGGATGCCCCTGAGGAGGTGCGTGCCCGTGACTTCCGCAGGGAGCTGGAAGAGAGGGAGCGTGTAGCTGCACGTGATA
AGACCAGAGAGAGGGGACCAAGAGAGCACACCACATCATCATCATCTTCGTCATCATCTTCAAAGAGGCCCAGACTGG
ATCAGATCCCAGCAGCCAACCTTGATGCAGATGACCCTCTCACTGATGATGATGAGGACGAGGACTCTGAGGAGGACA
GTGATGACGATGACACTGCAGCTCTTCTGGCAGAACTGGAGAAGATCAAGAAGGAGCGGGCTGAAGAGCAAGAGCGCA
AAGAGCGAGAGCAAAAGGCAGAGGAGGAGAGGATTCGCATGGAGAATATCTTGAGTGGCAATCCATTGATTAATTTGG
CGGGGCAACAACAGCAACAACAACAACAACAACAACAGCAGCAACAGATGAACCAGAGTCAGAATACATTTAGGGTCA
AGAGAAGGTGGGATGATGATGTTGTGTTCAAAAACTGTGCTAAAGGAGTGGACGAGGCACGGAAAGAGAAACGCTTCG
TCAATGATACACTGCGCTCGGAGTTCCACAAGAAATTTATGGAGAAATATGTAAAGTAAAGTACTTGATGTGTGTTGA
TTTGACTGTTTCTTTTTTTTTTAATCTAATTTCTTTGATCTCCTACTTCCATTTGTTTTTTAGCATTTAAAATTAAAT
GCATCAGTCTCTGTACAGTCAACAGGTAAAATGTTGTATATAAATGAGTTGATGAGTTACATCCTTTGAACAAAGCTC
AAAACAAGGTGGAAT

>Sequ11139EST3
GCACGTTGAGCGCCGGTGGCAACCTGAAACCGCCCACAACAGGAGGACCCGTGACGTCATATCTCAGCGACGACACGT
CTTCAGATTGCACCATGGCGGAGGCTGAGTCAAAAAAGGTGATCAAGTTAGTTCCTGAGTATCTACTGAAGAAGAGGA
AAGCTTACCAGGCCATTAAAGCCACACAAGCCAAGCTTGCACTTCTTGAGAAGAGAAAGGTATCGAAAGGCAAACCAT
TGAAATTCAAACGTTTGGAAGACTTCTTGAAAGCCAGCCACAAGAAGCATCGTGATGAAACTCGCATCCGCAGAACAC
AGCACAGGCCGCCTGCCCCTCTGCCTCCTGCTAAGAATAAGCTAGCCTTTGTTGTCCGCATCAGAGAGATTAAAGGTG
TCAGCCCCAAAGTGATGAAGGTGATCCAGATGTTGAGGCTGAGGAAGATCTTCAGCGGGGCCTTCGTCAAAATCAACA
AGACCTCCGTAGCGATGATGAAGGTGGTGGAGCCTTATGTGGCCTGGGGGTTTCCCAACTTGAAGTCTGTTCGTGAGC
TCATTCTGAAGAGAGGACAGACCAGGATAGGCAGGAGGAGAGTCCCACTCACAGACAACGCCTTCATTGAGCAGCACA
TGGGTAAACACGGCATCATCTGTCTGGAGGACCTGATCCATGAGATCTACTCAGTCGGTAAGGGCTTCAGGGCAACCA
ACAACTTCCTGCTGCCTTTCAAGCTGTCAGTGGCTCGTCACGCCGCCAGGGATAAAGCTGGGCTCCTGAAGGACCTGG
GAAACCCTGGATTTCGTGGTACGGACATTAACACTATCATCAGACAGCTGAACTGAGGAGAAACGCTTGCAAGATGGA
CACCCAGCGGCATCTTACAGGATGCTGTACTTCCTGAGGAGCAAACGCCACTGACAACAAAATATTTAAAATTTGTTT
CATTGGCAGAGAAGCTTGTGGGCTTCACACTGTACACTCATTCTCTGGTTACATTCTCTGGACACTGGGAGATATCAG
AAAAGTGGAGTCTTTTCCGTAATCATGTTGTTTTTGTTTTGTGCTCATGTTTTATAAAAATATATATATAACGTGGTG
CCCCTGACAGTGGACAGATGGAAGCAGCTCTATGGTGAAAATGCTGTATGTTTAATTAGTACATGTAATATTCCTCAG
GTTGTCAGAGGAACGGATCAATACTTTCTACATATACAAGGACAGGAAATTGATTAATTAGGATGAGTTGTCCTACGT
GCAGGAGCCACCAGCCCATCTACAGCAAATTCATTTACTGCAGTCAATAGGTTTCAATGTGAAAAGGGGTAAAAAAAT
CTGTTTAACACTGATTCACTGATTGATTGATTGACTGTTGTACACTTGTTTTTACAATAAACAATAATATTCAGTACT
TTTGCAGTGATAGCTACAATGTTTGATACATAATGAGAGG

>Sequ11146EST3
ATGCTGGCGAGCATGATCTTCAGGAAGGAGCCCTTCTTCCATGGCCATGACAACTATGACCAGTTGGTGAGAATAGCC
AAGGTTCTGGGAACTGAAGACCTGTACGACTACATTGACAAGTACAACATTGAGCTGGAACCTCGCTTCAATGACATT
CTGGGAAGGCATTCACGTAAGCGGTGGGAGAGGTTCGTCCACAGTGAGAACCAGCACTTGGTCAGCCCCGAGGCTCTG
GATTTCTTGGACAAGCTGCTGCGCTATGACCACCAAGCCCGGCTGACCGCCCATGAGGCCATGGATCACCCCTTACTTC
TTCCCCATTGTGAAAGATCAGTCTCGTGTGGCCGGATCAGCCAACTTGCCCAGTGGGAATACAGCTGTTAGCACAGCC
AGCATGATCACTGGTATCTCTGCCTTGCCAGCCTCCACTGCCCTGGGCCCTCTCATTGGCACGCCGGTCCTGTCTGCT
GCCACCAATGCCCTGAGCACCCCGGTGCCCGCTGCTGCCGGTGCCCCACAGTGACACCCCATCTAAATCCTCATCTCT
CCCATCTCCACCACCACCACCACCCAGCTCGGGTGAAGAACTAGACAGGTGCCATTCTGCCAGCCCTCACCACACCCT
GTCTAACTCAGTGTAACCCTGGCTGGAGGACACTGTCGTCAAAGAAAAAAAACGTTTCAGCATTCCTTTCCTTCCTTG
AATGAACGTGTTAACCTGACATCTGAAATGAATGTTCTGTGTAACTGAAATATACAGATAATCCCTCCATTTTCTCAC

AAGCTGTAGTGATTACATGGGTGTGTGTTTGTGTGGTGAGTCTGAGAACGAGTGTTGTCCATGGCGGATTTGGGTCTC
ATGACCCGCCCAGCCCCACCCTATGTTTTAGTTTGGACAGTGAAGGCACTGGTCAATAAGTACATCAACAGAAGGCAA
AAGAAAAAAGATGTTCAAGAGGAGCACATCCTGTGTAGTTCACTACTGCAGCTTTGTCATAAACAACATCTGATGAAA
ACACTCAGTTTACCTCCCGAGACCAGTCTTTACACCCTCCATGTTGCAGCATTTTGGAAGTGTTTGGTGCTGTCCTGT
GCTCCAGCTGTATGTGAACGCAATGACACTTGAACACTTTCTCTGCCTGAGTAGTCCTGTCTTGCCTTTCTTACCACA
CCAGATCACCAGTCTTCTTTTGATAATACGACTCCCCGTCATGTCATCCATTATTAAACTGTTTTGTATTGCAATGTT
GTGGACTGCTGGATAAGGTTCATTACCATCAGGTGGTTTGATCAACTCTAGTGTTGTACTCTGATCTCTTAAGAAACC
AGGTAAAGTTCATTCAGGTTCTCAAAGAAAGTTTCTCAAACGTGCTGCTTTCCAGATCCTTGAACTGCTTCAAGTGGC
TTTTCTTGAATTACTCCAAGACATAACCAAAGTTTACCCAGATGGTTGGAAGAAGAGACTCAGACCTAGTTTATTTGT
GGAAGTTACTTATGGAGACGCCTTGACACAGGTTGAAAGTGTGGGTTCTAGTGAATAAGATCACGGCATGATACAATA
ATCATCTGCAGGTGACTGAAGTGCTATTAGTGTTGTGACACGGGTGAGCTTAGTGATGAGTAATACAGTTAATTGGCC
TTCACCACCATGTTGAGGGAAGTATTGAAGTTGAATTGTTGAATAGCACTTGAAGGGTAGCGTGTTGCGCAGTGTTTT
AGTGTCAGAGTTTGAAAGTTTAAGTTTTAGACCCTGCAGTACATACGCATACTTTTATTTTCCCATTACAAAACCTTA
TAATGCAAATAAATCAGAAAATGTATACGTTGACCTGTGAACAGAGCAGTTCGAGTCCCTACATCACTGTATATATTT
TTATGTTCTTATGTAGAAGACTAGTATTTGAAAATATTTGTGTAAATAAACACGTGTTTAATTATCAGGTACATCTGA
CTGATTTATATGTCATGTTCATACAGACCACTCTTCTCACACACACTTTCCTCACAGCATCAAGGTTACAGAGTGCAT
ATGATCA

>Sequ11147EST3
GCACGGCGACTTAACGCGGGAGCACCGCTACAGAGAGCCAGCACCAGCCAGAGGCTTCTAGAAACAACGTGCACCGGG
ACAGTCACCGGCAAACACACTTGCTCTCCGCACGAAACGTTACACAGTATAACTTTTTTTATTATCATTTAGCTGGAT
AAATGGCGAAGTGGGGAGAAGGGGACCCTCGCTGGATTGTAGAGGAGAGAGCCGATGCGACTAATGTCAACAATTGGC
ACTGGACTGAACGAGATGCAACAAACTGGTCCTCAGACAAATTAAAATCATTGCTCCTCGGGTTGAGCGTGGAGAACG
AAGACGGGACGTGTGAGGTGACCGAAGTCAGCAAGCTGGAGGGAGAGGCCTCGATTAACAACCGCAAAGGGAAACTTA
TTTCTCTATGAATGGAACCTGAAAGCTGCTTGGACTGGAAAGTCAAAATCAGGAGTGAATTATAAAGGAACACTTGAA
GTTCCGAACCTGTCTGATGAAAACGACATGGAGGATCTTGATATTTCTGTTTCGTTGAACAAAGACGAACCCGAAACG
CCGCTGACCAACCTGATGAAAACGAAAGGAGCTGAGAAGGTGCGTGAAGCCCTGGGAAGCTACGTCGAGTTCTTAAAA
ACAGAGTTCACGCAGGGAATGATCCTGCCCACAGCCAACGGTGTGGCCAAGCCGCAGTCCACATCACAGTCCAAAGCC
AAGCTGGATAAAACTCAGATTTCCTCCTCAGGCAGCACCACTGCTCCAGTCAACACCGGCGTCAAGATCCCCACCTGT
AAATTCAGCATTAGAGAAACGTTCCTCACCTCACCAGCTGATCTCTACAGGGTGTTCCTCAACCAGGAGATGGTCCAG
GCGTTCACACATGCTCCAGCCACAGTGGACGGAGAGAGGGGCGGAAAGTTTCGTCTGTTAGAAGGAAATGTTTTCGGT
GAATTTACGGAGCTGATACCTGATGAGAAAATAATTATGAAGTGGAGGTATAACAACTGGCCCTGCGAGCATTACGCA
ACAATCACCATGACCTTCTTGGACCGGAGCAGCGAGACGGAGCTGAAGGTGGAGTGTCGAGGCGTCCCGGAGAGCGAG
GAGGAGCGGACGAAAGAGGGCTGGAAGAGATACTACTTCGAAGCTATTAAACAGACTTTTGGCTACGGAGCGCGACTC
TTCTGAAGACGGCGTGCTGTAGTACTGTTTCTCTTTTTCTGTTTTTTGGATTTTTAAAACTTCTTGTCCTTCGATCTC
TCTGGAACGAGAAGTCCCATTTGTAAACTCATAGCACGAGTTAACGAAAGAGAAGAAAATAGGTGAGGCCAGCCTGTC
ACCTGTCTGTGTACGTGTAAATGTTTTAATTTCTCAACATATTTAAATCTAGTTTCTCCCCGTCAGTATTTGGGTCAG
TTTTTAAACGGAGCGTAAATGGACTGTGCCTCTGGGACTCTGGATGAAAAATACATTGAATAAAGCAAAGGAGAATTT
ATTGAACCACTAATGCAAATTGGCCTTTACTTCTATAAATATGTAATTATTGCAAGAGTTAAAAATAAATGTTTGTTT
TCTTTTATTTGAAAAAATATTCTGTTGCTTTGAAAATTGAATGTTTATTTTTACTTTATTTACTCTTTTCAAGTGTAA
CTGTAGTGACGATGCTTTCAGCCGAGATGCATGTTCCATTTCAAACTTTCAGAGGGAGGAATCACCACTGGACTTGAA
CCTTACACGTGTTAAATCTAAGCAGTACTGTACGTTCAGTTGTTTCAATGAGACTTGATTTCATGCACATTATGAGCC
CAGCGTCCGTCTGGGGGATCAGCTCAGCTCGGACCTTCAGCGGGAGCGTTAAGGCAAATGTTTCAGTGTTGGAAACAT
GAAAACAGTTTCAATCTCTTATGCAGGTAGCTAGCGTAGCAACTAAAGAGAGATGTTCTGCTATATTTGGCTTCAAAC
TGTAAATTTCA

>Sequ11170EST3
AAAGCTGAGGAAGAGGAGGGAGTGGCAGAAACTGCTCAGCCAGAGGAGGAAAAGGCAGGACCTTCACAGGGAGCTGTG
GAGGAGAGTGACAGCGACTCAGATGACAGCAGCGACGATGAGAAGGAGATCACCAGGATGAAGCAGGCCAGGGGGGCT
GGTGGGACGTCGGGAGGAGCCGATGCTGACGACTTCCAGGTTGTTCCTGTAGAAAGCACCAGTAAGAAAGCCAGGATC
CTAGATGCAGAAGGCCTGGCCCTCGGCTGTCAGATCGCTACGTCTAAGAAGAGAGCCAGGGACCTGGTGGACAGCTCC
TTTCATAGGTTTGCAAACTCCGACGAGGCGTAGGAGGTTCCTGAATGGTTCCTGGACGATGAACGCAAACACCGGAAG
AAGCCAGTGCCAGTCACCAAAGAGATGGTGGAGGAGTACAAACAGAAATGGAAGGAGATCGATTCCCGACCTATCAAA
CGAGTTGCTGAGGCCAAGGCCAGGAAGAAGAGGAGGATGCTGAAAAAGATGGAGCAGGCTAAGAAGAAAGCGGAGGCA
GTCGTCAATACAGTGGACATCTCTGAAAGGGAGAAGATGGCTCAGCTAAAGAGTATCTACAAGAAAGCAGGGGTTGGA
AAGGAGAAGAGAGAAGTAACATACGTTGTGAGCAAAAAGGGAGCCGGCAAGAAGGTGAGACGGCCACCTGGTGTCAAG
GGAGTCTTCAAAGTGGTGGACAGTCGCATGAAGAAAGACATGCGGGAAATGCAGAGGAAAGACCAACATGCTAAAGGA
GGCAAAGGAAAAGGTGGCAAAGGCAAAGGCAAAGGAAGGCCGTCAAAGGGTGGCAAAGGAGGAATGAAGGGTGGTAAA
GGGCGAAAAGGAAATAAATTTGAACCCACAACATTCTTGCAATCCTGCCTTTCTATCTAACGATTAAACATTGCCTGG
ACTCATGTGTGCAGGTAAGCATTTATGGAGCTTTGTGAGCTGAGATGGGTCTCACACTTTGGGCTCCTGTATCATGGA
CACAGTGAACTTTGGTCATTGACATGGATGCCCTCTTTTTAGCAACCTTCGCTGAGGTTCCTTAAACCCCTGGACACT
AGAGATATGATCTACAGGTATTACAGTGCTGGTGTTCTGGTTACATCTTTCTGAAGATGTACATTATCTGCCTTGAGC
TCCTCGAATGGAGCTGTTTCACCGAAG

>Sequ11176SNP3
CTGTTTCTGCTGTGGCACCGGTAAGTGAACCGGATGTTGTCTGAATGTCAAAGATGGCGACGGACATGTCACGCTGAA
CTCGTAGCAAATTGTTCCACAGTATGATTTTAAGGATATAAAGCCTTACCAGATGCTAGCACACTGACCGACTCTGTG
TTTTAGTTTGTGAGGTGAACACAGCTGAGGAATAAATGGCTCTGAGTGATTTAAATCGCTGTGTGCGCCAGTTGTTAG
GTCAAATTCAACGCTGTCGCCCGGTTGCAGCTACAAACCAGACAGCTCGGTGTCTCAGCCAAGCAGCCTCTCAGCCGG
AGCCCAGCGCGAACGAAAATGAAGCCGTGAACCCGACCTTTGTGAACAGAAACCCCCGGAACCTGGAGCAGATGGCGC

TGGCTGTGAAGGACCGGGGCTGGAAAACGACCTGGCCCCACCGGGAGTTCTACCACAGGTTGGTGTTTTCTCGCACCC
AACATTATGTGACAGCACAAGTGTTCTCCAGCAGTTCTCCTGTCCCAGTGTTGACCTGCTCAACCACAGAGTGGGCAT
TGAAAAAGGAATTGGCTTCTACAAGGTGTGTGGCAGCATGTCAGGCTGTGGGTGAGGTGCTGGCACGTCGATGTCAAC
AGGCTGGCATCACCAGGATGGTGTACAGGGCTATTCCCTGGGCCTATCGCTCTGACGCTGTTCAGGCTTTCAGGAAAG
GAATGAAAGAAGGAGGAATCACGCTCAGTGAACCCAGAAGAAAATACATTGGGACCTAAATTTTTGTGTCATCTATCA
CATCTGCTGCTTTATTAAAATATCCTCTGTAACCCTTATTATATCTGCTATGTTGTGAATTTATCACTGTACTGATAT
TAAGATGACAAGCACTACAGTGAAATACAATACAGAGAATCAAAATGAGCGCTGAAAGTAATGTTGCTTGTCCCTCTG
CTTTGTTTCATGTGGATAAAGTGTTTCTGAATATAACCAGTCTGCTCTCTCTGCTCTGGTGTGTTATTTAATCAAGGA
ACAAAGCAGTTGGTTTGAGGATAATCATTTATTTTTCACTCATAGTGCTTGTTGTAAACAAGAGTGAGGTGCAGACAG
TGGAGGCTAAACATAAGAAGGCATATAAATGTT

>Sequ11252EST3
AGAGGCTACTCTGCTAAAGCGGGAGAGGGATTAAAACAAACTGCGAAGAAGACTGCATTTCTTACCAGCCCACCAAAC
TTTGTAGCAGACACTCTGTGCTTTCGGTCGAGTTAAACAGGAGGAGAAACACACTAAAAAGAGTTTCTACCCAGCATG
GACAGAGGGCCAACAGAGGGTGTAGGGGTGGTGGAGGTCCCTGCCAAAGACTTCCCCTCCATCCAGGCCGTTCACAGC
CAGGATGCCATGGTTGCTGACCTCCTCCAGCAAGCTGCAGAGGCCGGGGGTGTGGTAGAGGGACAAGCTGGAGTCGTC
CTTGAACAAGGTCATGGAGAAGGAATGGTATCAGCCACTCAGGCCCAGCAGCAGCTGATGGGAGCTGGGGTCGGGGTT
GGTGTAGAAGGCGGAGCAGGGGTTGAAATGATGGTTATGGACTCACTGGATCCCACCCTGTTGCAGATGAAGACTGAG
GTGATAGATGCTGCAGTGGGGGGATCATCAGCAGCAGTGGGTGTTGTTGGAGGGGTCGCTGGTGCTGCTCACCAAGCA
ACTGTAACAACAGTGGACCAGACCCAAATTATCACACTACAGGTGGTAAACATGGAGGAGCAGGCAGCCCTGGGCCTC
GGGGAGCTCCAGCTGGTCCAGGTGCCTGTTTCTGCCACCACCGTGGAGGCTCTGCAGCAAGGCACTTTTGTAGACACC
ACTGCAATGCCAAAGGATGGAGACCCAGTCATCTGCCACACCCTGCCGTTGCCCGAGGGCTTTCAGGTAGTCAAGGTT
GGTGCTAATGGGGAGGTGGAGACAGTGGAACAGGAAGAGGAGGGAGGAGAGGCCCACCAAGAGGAAGAAGATGAGGAG
GTGGGGAACCAGCTGCTGGAAGAAGGGGAAGATGAGCCCATTCAGCCTCCGAATGATGACCCAAACTGGGCTAAAGAC
CCAGACTATCAACCTCCATCCGGAGCAGTCAAGAAGACCAAAAAGGGTAAAAAGAGTCGTCTGCGTTACGCTGATGGA
GATAAGGACATGGACGTCAGTGTGTATGACTTTGAAGAAGAACAGCAGGAGGGCCTTCTGTCTGAAGTCAATGCTGAG
AAAGTGGTGGGCAATATGAAACCACCCAAACCCACAAAGATCAAGAAGAAAGGAGTGAAGAAGACGTTTCAGTGTGAA
CTGTGCAGCTACACCTGCCCGCGACGCTCCAACCTGGACAGACACATGAAGAGCCACACTGACGAGAGACCTCACAAG
TGTCACCTGTGTGGAAGAGCCTTTAGGACGGTCACCTTGCTAAGAAACCATCTCAACACACACACCGGAACTCGTCCA
CACAAGTGCACAGACTGTGATATGGCTTTTGTGACTAGCGGGGAGCTGGTTCGTCATCGTCGCTACAAACACACACAT
GAAAAACCCTTTAAATGCTCGATGTGCGACTATGCCAGTGTGGAGGTGAGTAAACTAAAGCGTCACATTCGTTCCCAC
ACCGGTGAGCGTCCATTCCAGTGCAGTCTCTGCAGCTATGCCAGCAGAGACACGTACAAGCTGAAGAGACACATGAGG
ACACACTCAGGAGAGAAACCTTATGAGTGCTACATCTGCCATGCCCGGTTTACCCAGAGTGGAACCATGAAGATGCAC
ATCCTGCAGAAACACACAGAGAATGTGGCCAAATTCCACTGTCCACACTGTGACACTGTTATTGCACGCAAGAGTGAC
TTGGGTGTCCATCTTCGTAAGCAGCACTCATTTATTGAGACTGGAAAGAAGTGTCGTTACTGCGACGCTGTTTTCCAC
GAGCGCTATGCTCTGATCCAGCATCAGAAGTCCCATAAGAATGAGAAAAGGTTCAAATGTGACATGTGCGACTACTGC
TGCCGCCAGGAGCGCCACATGGTGATGCACCGTCGAACCCACACCGGAGAGAAACCATATGCCTGCAGCCAGTGTGAG
AAGACCTTCAGACAGAAGCAGCTGCTGGACATGCACTTCAAACGCTATCACGACCCCAACTTTGTGCCCACCGCCTTC
GTCTGCCCCAAGTGTAGCAAGACCTTCACTCGCAGGAACACTATGGCGCGCCATGCTGAGAACTGCAGTGGTGAGGTG
GAGGATGCCGAGAATGGAGCTCCAACCCCAAAGAGAGGGAGAAGAGGAAGAAAGAGGAAGATGAGGAGCAGGAGAGAT
GAGGATGACAGCGAGGAAGATCACGTTGAACCTGACGAGGAGGAGGAGGGTGAGGGTGAGGGTGAGGAAGAATCATCA
CTGCTACAGGAAGAGGAGGAACCAGAGGGTATGGAACTGGACCAGGCCCCTGCTGCCATTCCTGTACCGGCCCCGACG
AGCCACCAGTCAAGAGGAAACGAGGCAGACCCCCGAAGAATGCCCCTAAGCCTCCCGCAGCCAGCAAGCCAGTCAGGG
TGGCTGCCAAGACAACAGCTTCTGCTGCTGCCATCATTCAGGTGGAGGATGAGAGCACCGGAGCAGTGGAGAACATCA
TAGTGAAGAAAGAGGAGGGCGACGCCTCTGCAGCGACACCCCTGGAGCAGGGAGTGGCCCTGACAGTGGAGGGGTGGG
CTAGATGGGGACGGGTGGAGACTGTTGAGCTGGCTGTGAATGAGGAAACGGCAGCCGCTGCTGCTAATGGAGACCTGA
CGCCAGAAATGATCCTCAGCATGATGGACCGGTGAACACATCACAGACGCACACACGCAGTCGGACTCTGTCGAAAAT
TGGCGAATACAAATTAACGTTTCTGCATCCTCAGCCTTCACCTCATGTTCCATCTGTGTGTGCGCTTAAGTGGCAGCC
ATTTTGCCTTCATTGGAGTGGCTGCAAAAATGGTGACAGCAAAAACAACCATGCACAAAGTAAGGAAGCCACATGTAA
ATACTTACACAGCAACTTCACATCTACGTATGTCAACCTGCAGACTGACGTTTGTGGGTCTCATACACAAAATCCCCC
TGCACATACCTGTGTCCTCTCCTACATCATATAAGGATATTGTCCAAACTAATGACTGACTGCTCTTTGCTCTTAAAT
GCTTTTTTCAGTCTGAATGTCTTCTGAATGAATCATTGTTTATTTTGCTACTTGTGTCTACCCAAAGCCACATCCTGT
GTCAGTCCTCACCCACACCTTCTGCAGTTAATTTCTCTATGTTGCTCTGCCACAAGGGGTCATTCTTTCATCCACTTT
CTCTTTTTACTTTGTTTTTCTGTCCTGCTTGAGGTTCCTGCAGCTTTGCTGATTTAGGAGATTCCAAGCCTTTATTTG
TGTACGTATAGATCTAAATGTAGTTTTTTCCTAATGAAAATGTAGTGTATGGTACTTCAGTTTGCCTCTGAGATATCA
CCTGCTCTCAGTCATGATCTGTATATTTTTCTTCCTTGGCCTGTGTAAACACATGGTCTGTTATGTTATCAGTCAGCT
GTTTGTACATTGACAGAAGGATTCTTTATAAATGAGCTAATGAGGCAGGTGTATTCAAATGAACATAAGTCAGTGATA
ACTGTTTCTCAGCTTCCTAGTTCATCATCCGCATTGCCAGCACCCAACCCTGTTCTTATGGAGACTTGCCCTCAGTCA
GCTCATGTACATTAAATACTTGAGGTAGTTTCCCAGAACCAACAGAGTTTGAGATGAAGCTATTTTTCGACTGTTTTC
TATTTTCTGCGACCTGTCACTGACAAAAGATACTTCTCATTCGTAGCTAGTGTTCCTCCTGTTCGAAAGAGAACATAG
ATAGTTTGCCATCAGATAAACTGAATGATCCCTGCTGGATGGAGAAGATGCAGATGATCCTGATTGTTACATATTGAT
CATTGAACATTCCAGATACTTGAGTTAATATGGAATTATGCCTGTGCGTTAGAATCATCTCATCTGAAGAACTCGGCA
GTAACAAACTCAGTATTCACACCCCAGTTCACACTGTGTTTGGAACAGTTATCAAAGAGGTTGTCAATCACCCCCCCC
GCCCTCCCCCACAATAATCAATGATCACCAACTGTGGCAGGTGATTTGCATGTAAATACATATTTTAAACCCTTTTGC
TCTTTGATTTCTGGTGAACCTTTTTAATGTTTTTAAATAGCTGGTTTGGAGATGTTTTGGTTAAGAAGGACATCATGG
TTTCACTTGTGGTCAACATGTCAGTACTGTGCCTGGTACTTGACGTCTTCAGCCTGTAAAGGTGCTTTTCCTTCCACA
GATGAACCCATCTTAACCAACCGTGCTGACTTGAACTAATCTGAATCACTATGGTTTTCTGTCTGTATTCTGTTGCTC
ATAGCCATTTTCTATCTGCTTTGATACTGTTTTTAGTTTTCATTTCTAGCCATTCTTTTGTAGTGGTCACTGGGTTTC
CCTGCAGGCTGGTTGGTTCAGTTCAGTCCTATGACAGAAGCTTTGTCTCACTCTGTTCTCCTTTAACCTTTGGCCCAA
AGGCATGAGGCTTTCATCCATGAAGGATCGAGAGGTTTTTGAGTGTCTGCTGAAAGACAAAACTGTTTCACTGAATGA

AGTTCAGCTAGTAAATTGTAATTAATGACAACAAAACAGCCTTCATGGTCTTTTTGACAGGTTTTAAAACTGTTTTTA
GAAGGATGACAGTTTGTGGAAGCTGTGTATTTTTGCTGTAATATGATTAACTATGTAATCAAGAGGGCTAATCCACAC
TGTTCTCAGCCTTGGACTAAGGCCCTGTTAGCCCAGGTTTTGCAGCTCCACTAGTATTTCAGTCACTGTCCATTTGCC
ACTGATTCTAAGGTATGTCCTAACCTCAGCAGAGGCCAATCACTCCTGATCAGAGCTGTCCCTGATATTTTTTTTATA
AAACACAGAACTAAAATCACTCACAGGAATTTTCACAGTCCGGATCTCAACAAACGTGAATATGTTCATCCTCAGACT
CGGGCTAAAAAGTCGGGGTTTGACCACAGGTTATGATGGTCGTTATGTGGCCTTCATTTTCTTTCACCTGTTTTTGTT
ATATATAACATCTCCTCTGTAACATGACACTGGTTAGATGCCCAATATTAAATGTAAAAATGAGGATCTAAAGAACCA
TGGTCACAAGATATCTGAGGGAATCAATGTTCATGCATCAAACTGACACTGTATGTTCTGTGACTGTACTGCTGTGGT
CTGTTATCTGTCAATCATGTTATTCTGCAGACGGACAGATGATGCTTCGCTGTTCTCATAGCAACAGTATTGTTTGTT
CTTTGCACATTGCTTGTTTGTTTTGGC

>Sequ11253SNP2
CAGTCTCGTTTTTTTTTTTGTTCAGCTTCCTGGAGAAGACATTCCTGTTTCGTTTATTCGAGCCCAACATGGATGTAC
AGATTGAATTTGTTCATATGAGTATGTGAATGTACATGGGAAGTAGAGAAGTTCTACATTATTATCATGTGTTCGCAG
ATGCTGTTGAATTACCGTGAATAATTAAAAAGGAGCAGAAATAATTCATACAAACAGAGTATGTTCAGAAATTAAAAA
TGGAGCTGTATAGCAAGTAGGAAGCTTGGCTACAGAGCAGCAGTTGCACAAAAGTCAAACAACACAAGACAAGACCAA
GGATTCAGAAGATATAATCCAAGATGGAAAATGCGGTCCTTCAGAGGAGGGTTAAAGCTGGAGATCTTGGCTTGGTCT
CACAACTCAAAGCAGAAGTATTCAAAATTCCAAAAATAGAAACAAAAACAAAGTCTGACACATTTTCTTACATCACTT
ACATGGAGTTAATTTCAGTTCATAGAGTGGCTTTCACTCTCTATCAGCTGATGAAGTCACTGTCATCAGGGTCGTCCT
GTGTGTGGACGATGAGCAGAGGGTTTCTGCGAGGGCTCAGCTCTAAGAAAGTCCCACCGCTGCTCTTCCCAGATGTTG
CTCCAGTCTTTGCCTCGGCGGTGCTCAGCTGTGAACGGTCTCTCCCTGCTTTGTAGCTGGAGTCTTGAGAGATTTTCA
GCTCCTCCACCACCCGCTCCCCAAGCTCCTGGATCAGCTGTCTGGTGTCTTCTGAAGCTGAGGGGAGGGCGTCCACTT
TCCTCTCCGCGGAGGAGACTGTGCTTGTTGGCTCCTGGGGGATGGGAGAGGCAGCGGAGGTGGGTTGGGGCTGGAGAG
GGTTGTCCTTAGACGTCTCTCCTCCTCCTCCTCCCTGGTTGGATTCGGTGCAGACTTGCCCTTCAGCCTCAGATTCCT
CACTCTCATCAGACGAGCCGCTG

>Sequ11258SNP2
TAGTAGGAGGTTTCGGGTTTTTTTTGTAATTTGCATTTATTCTAAAGGGAAAATATATAATTCAATAAATATTGTGGC
AACAACATCGGCAAAGACATTTTGAAGGTGACATCAACATTCAAGAAAACAAGATAATGTACATGGGGAGGAGACAAC
AGATTAGAAAATGAAGATAAGAGTCGGGGGCCGGGGGCTCTGCCAAGTGCGACAGCGCCGGCATTTCAACAAGCTAAT
GGGGGAGTTTGAAAGTTACAATAAAAGTGCATCAATGCCTGATATTCAAACAGCTTCTTAATTGCACTGTTAGAGGAA
AACCACAAATTCGAACACACTGTTTTTCCAGACAAGTTGACCATTTGTTGTTTACATTTTGTCATGTTTCAAAAACTG
AACCGACCGTGTAGGAAAAATAAAATCCTCCAATTTTGAGTTCACACCAGAAAGCGTCTTGAAATATGTGGTGAGGGC
CAATTAGTAAACTACTGCAGCTGGCGAGCTAACCGCTAACACACTATCCAGCCTGACTAACGCTAGTCAGATACAATA
GCCCCGTGAGCTAATGTCTATTTTCTCTCTTTGTACTGATTATTCCCTCCTGCCATTAATATTCTCAGGATTTTACAT
CATTTCATTCAATAAAAAGCTACTGAAGGGAGGAAAACACTCCATGCTAACAGGTGTGCTAAGTGTTAGCTAGCAGGT
TTTGTTGCTAACAGTCAACAAGTTCACACAGTTTAATCAAAAGTCATATCTGTACCAACGAATCCTGTCTCAAAACCA
CCTTTTCTATTTGTGGAGCAGTTAATTAGGTTAAATAAAAGTGAAACACAGCTAATTAGCTACGCTAGCTTCCTCTAG
CTCCGTGTTAGCACTGTCAAAAACTGTTACACTATCG

>Sequ11268EST3
ACTAGGTTGTGTGTAACGACCGGCGATGACTAGCCATTAGCTTGTTTTCTCAAGAAAAACTTACACAGTGTTCATCGT
CTCCGACTGCCTTGTTAACCCTGTCCGCTCGAGGCAGTCCCGGTGCAGCTGAATCTCGCTTAAACTGTGACCCAGTGT
GGAGGTGCACCATTAGGATGTTCTATCAATACCACCACCTCCTCTCCCTCTTTCCCCTCCTCCTGCTGCTGGGTGTGG
GAGTCTCTGCGGTGGAAGTGCAACGACCTCGCGGTGTACCTTTATCAAAACGGCAGTTCTATGAAGAGGGCAAACCAT
TTACTTGTCTGGATAACTCCGGCACTATTCCCTTTGACAGAGTGAATGATGACTACTGTGATTGCCAGGATGGCTCTG
ATGAGCCAGGCACTGCTGCTTGTCCCAACGGCAGCTTCCACTGCACCAATGCAGGTTTCAGAGAAGCCTTCATCCCCT
CCTCCCGCATTAATGATGGAATCTGTGACTGCTGTGACACAACAGATGAGTACAACAGTGGTGCCGCCTGTCAGAACA
CCTGCAGGGAAATGGGGCGTAAAGAGAGGGAGAGCCTGCAGAAGATGGCAGAGATCGCAAAGGAGGGCTTTCTGCTTA
AACAACAACTCATACAGGAGGCCAAGAGGGGCCTAGAGGATAAGAAGGCCAAACTTGTAGATGTTCAGGTCAGTAAGA
AGGATCTGGAAGACAAGGTGGAGGCACTGAGAACTGTAAAGGAGACTGCAGAGCAGCCAGAGAAAGAAGCTAAAGAGC
GCCATCTGCAGGCCTGGGAAGATCAAAAAGCTCTTGTTCGTATGGAGAAGGACAAGACCAGAATGGCTGAGGTTTTTC
TTGAGCTTGATGATGATGCAGATGGCTTCGTCTCAGTGGCTGAACTTCTGTCCCATTCTGAGCTCGATCCAGATTCAG
ATGGTTCATTCACCGAAGCAGAGGCTCAGGCATCGTTGGGAGGAATGGACAAAGTGGACACAGTAGCATTTGAGGCTG
TTTGGAATAACATCAAAGAAAAATACACATCAGAGGCCGCCGCAGACACCCCAGCACCAGTGGAGACTCCACAGGAGG
AGCCAATCTCCGACAATGACTCTGAGCAGTACCCTGAAGATGACATCCCAGAGGATGAAGAGGAGGAGGACGAAGAGG
ATGAAGATGAAGAACCAGATGATGGAGATTATAAGACCCCTCTCCGACGCCAACTCAGGAAAAGAAAGACGACGATGA
GGGGACTATGCCGCCCTATGACCAAGAAACGCAGAGCCTCATTGATGCTGCACAGAAAGCCAGGGATGAGTTTGACAC
GGCTGAGAGAGCTCTCCGGGAAGTGGATGATCAGATCAGGAACCTTGAGAAGGAAATTTCCTTTGACTTTGGACCCAA
TGCTGAGTTTGCTCATCTCTACAGCCAATGTTATGAGTTAACTACTAGCGAGTACATCTACAGGCTGTGTCCGTTCAA
CAGAGTATCCCAGAAACCCAAGTACGGTGGATCTGAAACTAATCTAGGAACATGGGGAAAATGGGCAGGTCCTGAGGA
TAACATCTATTCGGTGATGAAGTATGAACATGGAACAGGATGCTGGCAAGGCCCGAACAGGTCCACCATGGTTAAGTT
AACATGTGGAAAGGAGACGGTTGTGACATCTACCTCAGAGCCCAGTCGCTGCGAGTACCTGATGGAGTTCACCAGCCC
TGCTGTCTGCCAGGAGCCCTCCAGCCTGGATTCTTTGCTTCATGAGCACGAAGAGCTCTAGGTCCGCTTGATTTGTTG
TTCCTGAGGGTACCACACAGTCACTGCAGCAACACGACGTTGCCATGCTGTGAGCAGCACCAACTTGTTGTCGGATAA
CAATCGAGAATAGATGATGGAAATTCCAACGAGTTATGTCGTCTACACTTTAAACTTCCACTGCTACCTGTTGTGTTG
ACTTTCTGATTAATCTCAAATGTAGTGTCAT

>Sequ11296EST3
CCCTTTCCTGTCTACGGCCTTTTGGAAGATGGCGGTGCAAATCTCCAAGAAGAGGAAGTTCGTCTCAGACGGTATCTT

CAAGGCCGAGCTGAACGAGTTCCTGACTCGCGAGCTTGCCGAGGATGGCTACTCCGGTGTGGAGGTGCGTGTGACTCC
AACCAGGACTGAGATCATCATCCTGGCTACAAGGACCCAGAATGTTCTGGGAGAGAAGGGTCGTCGGATCAGAGAGCT
GACCGCTGTGGTCCAGAAGAGGTTTGGTTTCCCCGAGGGCAGCGTGGAGCTGTATGCTGAGAAAGTTGCCACTCGTGG
TCTGTGTGCCATCGCTCAGGCAGAGTCTCTGCGCTACAAGCTGCTGGGAGGCCTGGCTGTTCGTAGGGCATGCTACGG
TGTTCTGAGGTTCATCATGGAGAGCGGTGCCAAGGGCTGCGAGGTTGTTGTGTCTGGCAAGCTGAGGGGTCAGAGGGC
TAAGTCCATGAAGTTTGTGGACGGCCTGATGATCCACAGCGGAGACCCCGTCAACTATTACGTCGACACAGCTGTCCG
CCACGTCCTGCTAAGGCAGGGTGTGCTGGGCATCAAGGTCAAGATCATGCTGCCCTGGGACCCCAGTGGTAAGATTGG
ACCCAAGAAGCCCCTGCCCGACCACGTCAGCATTGTGGAGCCCAAGGAGGAGACCCTGCCCACCACACCCATGTCTGA
GCAGAAGGGGGCCAAGCCAGAGGTGCCCGTCATGCCCCAGGGAGCACCTGTCCCCACAGCATAAAAGGGTTTTCAACT
GTTCCACCACATGGAAGCAAGACCCTGTGTTTTTTGTGTACAAAAACATAAAATCTGGAAT

>Sequ11320EST3
ACACGCCTATCGGACAACTAAACCCGTTACCCCTAATACTTCCGGCGCTTGTTTGGCTGGCGGTTTGAAATGAGATTG
CTGCTGTAAAAAAAACACACTTTTCGTGCTTAAATGGCAGGTCTATTAAGAGAGAGTATACGCTGCGGAGCAAGGAGA
GTATTATAGAGCGTAATACGGGTTCAACTTCACTCTCGCTTTACTGTACTATGTTCAAAAAGATGACTGAATTTGGTC
CAGATTCCGGGGGGCGAGTTAAGGGAGTGACTATTGTGAAGCCTATAGTGTTTGGGAACGTTGCCCGCTACTTCGGGA
AGAAGAGAGAGGAGGATGGACACACACATCAGTGGTCTGTCTATGTGAAGCCTTACAGAAATGAGGATATGTCAGCTT
ATGTGAAGAAGATCCAGTTCAAGTTACATGAGAGTTATGGTAACCCACTGAGAGTGGTGACAAAGCCTCCATATGAGA
TTACAGAGACGGGCTGGGGCGAGTTTGAGATCATCATTAAGATCTTCTTCATTGACCCCAATGAGAGACCTGTGACTC
TGTACCATCTATTGAAGCTGTTCCAGTCAGACTCCAGTGCCATGCCTAAGAAGACAGTTGTCTCTGAATTCTATGATG
AAATGATCTTTCAGGATCCTACAGCCATGATGCAGCAGCTACTGACCACATCAAGACAACTCACCCTTGGTGCATACA
AGCATGAGACAGAGTTCAGTGAGTTGGAGCAAAGGACCAAGGAGAAAATGGAAGCGGCAAAGAAGAGAACCAGCCAGG
AGATCGCAGAGCTGAAAGACAATTAAAAGCCAGCAGAGAAAACATCAACCACCTGAAGTCAGAGATCAGGAAACTGGA
GGAGGACGGAGACCACAAGGAGCACTGAGAAATGGACAAACACAGCACACATGTGAACGTGATCATTTGGTATGGCTC
CTGACCCCATCCCTGATTTGAAAGAAACAAATGCATTTTTAGTGATCTACTGTGTGTTCAAAGTAATGGTAAAAACTT
AATTCAATTCAATTTTATTTGTTGTTGTTTTTTAGTTTGTTTTTAACTGATCTTGTCATTTTGTATCTAAAGT

>Sequ11351EST3
GGTATGAGTATGACAAGTGCACTATATGATGCAACGAAGACACTTGAACAAATATTAACATGCAGGTTTAAAACGACA
GGAAGCGGTCACAGATAACAGACTTTGAACTACCTCAGCTACAGCCAAGCAAAGAAGCTAACTACCCTACTTGCTTTT
AAAAGCACCCGTTACGTTTTCAACCGGTTGAGGAACTTTACGACGCTCCCTAATCACCACTTCCAACACAACCTTTGC
CCTTCGTCGCGTAGCTCGCTGCAGTTTTGTAGCATGCAGTGGGTAATAACAGGAGGACTGTGCACACTACGACAGAGC
CTCATAATCAGAGACGTGCTAACACATTTGCCAAGGTTGGTGACTCGTCGGAACATGTCGTCTCAAGCGAAACGGCCG
GTTTCCTTACTGGGAACTATGGCGTTCGGTGGCCGAGTTGACGCCGAGCAGAGCCTGGAGATGGTGCAAGCTTTCCTG
AGCAGGGGACACAAGCAGCTGGACACAGCCCTCATGTACGTGGATGGGAAGTCAGAGACCGTCATAGGGGGCATGAAT
CTCCCCAAAACAGTAAGCATTGCTACCAAGGCCAACCCCTGGGATGGGAAGACGCTGAAGCCAGACAGTGTGCGCTCC
CAGCTGGAAACCTCCCTTCAGAGGCTGCAGACCGATTCTGTGGACCTTTTCTACCTCCATGCCCCTGACCACCAAAAC
CCCATCCAGGATACCCTTAGGGCCTGCAATGAACTCCACAAAGAGGGAAAATTCAAGGAGTTTGGCCTGTCAAACTAT
GCATCATGGGAAGTGGCTGAAATTGCCACCATCTGCAGACACAACAACTGGATCGCTCCCACTGTTTATCAGGGGATG
TACAATGCCACTACAAGACAGGTTGAGACAGAGTTGCTGCCATGTTTGAGATACTACGGAATGAGATTCTACGCATAC
AATCCTCTAGCAGGTGGTCTTCTGACAGGGAAGTATCATTATCAAGACAAAGATGGTTCCCAGCCTGCTGGTCGGTTC
TTTGGTAACAGCTGGGCCACAGCATATCGGGACAGATACTGGAAGGAAAGTCATTTCCAGGCCATAGAGGTGGTTCTG
AAGGCCTTGGAGACGGTGTACGGCTCAGAAAAACCCACCCTGACTTCTGCTGCTATGCGCTGGATGTACCACCACTCC
CAACTTAAGGGCGATCTTGGAGATGGAGTTATCATTGGCATGTCCAGCATGGAGCAACTTCAGCAGAACTTGGCTGCT
TCAGAGGAAGGTCCTCTGGATGAGCGAGTGGTTGCAGCCTTCAATGAAGCCTGGAATCTCGTAGCCCACGAGTGTCCA
AACTACTTCAGATGAGGACCCTCTTTTTAATGTAGAGCCAAAATCTCCATTACAGTCCAATCAAATATATTTCAACTT
TTTACAGAGATCTGCTAAAGCCCCATTCCTTTCAGTTACTTAATAGTTTTCCTGAAATCACACTTAAAATTTGCATTT
TTTTGCATCAATTGGAAACCATTATGTAATATGCAAGAGCATTTCAGTGTTGATGTATAAATGTAAAAATGACAAGAT
ATTTCTTCAGGGAGCAATAACTTTTTCTGGAGCCCGCCTTAATTAAACTGTTACATTTAAAAAGATAACAAAAATTTC
TGATGTCAACAAAATGTTTTTCTTACACATAAAATGCATCAAATGGGTAGTTGTAACACATGAAACGCTATGTGCTTA
TTTGTTTTGGCTGCATTGCAAAGTACTTGCATGGAATACAATTCTTTTTGTCATTACATTGACAAATATTGACAAAGC
AAGTGGCATCAAGGTGATTACATGTACAAGATTGTTGAGTCTTGACTCCAGTCACCAGTGTCTCCTGAACAACAATTC
AGTCCAAAGCTAAATATCAGGGGGGATGACAGATGTTCATGTTTTGGGGTGAAAGCAAGACAGATGAGAGAGATTAAC
AAAGAGTCCAGGGACAGAACAGGCAGTCGGGATGAATGGTTTCAACAACAATGTCCCTCTGGTGTTGAGGTTGGCAAA
AAGACTTTTAAAAACAGACGTGTAACAAGCCCCTGCCCATGCCGATGTTTTATTCTGCATCGTCATCTTCTTCATCAT
CTTCATTCTCCTGCATAGACTCTTCCTCCCCAGCAAAGTTGTCAAACTCACCTGTTTCTGAGTTCCACATACATTTGA
TTTGCACCTTGAATTCTGCCATCTCGATGCCAAAGAGTTTCAGAATACGAGCCTGCTCAGGAGACAGCATATCTCCCT
CCTTACAGACCTCATGGTCCTTCAGCAGTGTTACCACTCCTTTCTTGAGAGCGGTAGGAAGTCCCAATTGTCTCAACT
GGGGCTCCATTGAGTGAGGAAACTGTTCCAGGGGTCCCTCATCCAGTGTTATATCCATCTGTGCCTGGTTGCCAGCTC
GCGCATAATCCACCTCTTTGAAATGATTGAAATACTCTTGTACCTCATCCTTTGTTTTATTTGTGAATAGTACACCCA
CTTCTCCTCGTAGATATTTGCAAACCTTGTGCAAATTATCTTTGTATTCATCTGTTTCTCCTTTACCCAAGGCGATCA
TCATGACCTTATTTTTGCCGAAGAAGAATCTGCTGTGTTTCCATGCTGTCCTGATGTCTTTCAGCTTGTTATTCCTCA
TATTGGCCACAGAGAATATGAACAAGTTTCTGTAGGTGTCCACACATTTCCGTAACTCCTCGATTAATTTCTGTTTCA
ACTCCAGTCCCTTCTTGGCTGTCTTCGTTAATGAAATTTTCTTGTCCCTCTTTGACTTCGGCATGGTTTCCTCTCACG
ATGGCAGCGCAGCAGACGCACA

>Sequ11369EST3
AGGGCGTGGCAATGCGCGCGAGTCGCGGTGGTGTGGTCTGTGGTGGTGGCGGGTGAACATCACGTGAACACTCGGCTC
ATGTGACTCGGGAGACTCAAACCAGGAACAAAATGGCTGCAACTGGAGACGACGCCAGCGCGATGGAGAGCATCTCCA
GGGCGCCAGCGGCTCATACGGCGGCCCCGATCCCGGCTCCGAGCTCCAGCAGCCGCACAGAAGTCCCGCAGGATGCGG

AGGACAGCCCGGCGGCCGCTCCGAAGAAACCCCACACCAACAGTGATGGAGGTGTGGAGACAGCAGAGGAGGCTGTGG
AGCCTGCAGAGCCTGACATCAACGAGTTGTGCACTGATATGTTTGAAAAGATGGCCGTCTTCCTGCAAGGAGAACTCA
CAGCCACCTGCGAGGATTACCGCCTGCTGGAGAACATGAACAAGCTAACCAGCCTGAAGTACATGGAAATGAAAGACA
TCAGCATTAATATCAGCCGTAACCTGCAGGATCTCAACAACAAGTATGCCAGCCTACAGCCTTACTTGGACCAAATAA
ATCAGATTGAGGAGCAAGTGTCTTCACTTGAACAAGCTGCTTACAAACTGGACGCATACTCCAAGAAGCTGGAGGCCA
GATTCAAAAAACTGGAGAAGCGATGAGGAGGAAGAAGAGGCAGACTGAGAGTTACGGTGCCTTCTCGCCTTCTTTCAC
CCTGCCTTGCCTCAGTCGTTCCCTGCCAGCGGAGCCAGGAGCTGCGGGGCAGGATGTGAACTGAGACTGAAACAACAA
ACAGTCCGAGCCACATTGACACTTAGAATCGACCGAGGAGGAAGCAGCTGAAGGCACATGAATTAGTACTGCAGTGCT
TGGCTTAGATTTAACTCCAGTATCACAGTTTTTTGGCGGCAGTCGTGCAGCCAAGTTCACGGTGTACAGCCGCGTTCT
TTTTGCAGACACACTTTACGTTGAATAAATCTGCCTGATGACCTGCATATCCTAACTGAGGGCTGGAATCAGAGTGTA
CTAATCTAATACTAAACATTAAGACACACAGCAGCTACTTAAACCAGTGGAGTATATAATGACACTTTGGTCATGTAT
AGATCATTTACATGACGAAGCATGCTCATTGTTTTAATAATGACCCTGAGAGTTAATAGTAGCCTAAAATCTACATTG
GGAGATTGAATTATTCTCATTACAGATTTGTTTTCCTGGCTGGTTGTGAACAGCACCACAGTCTGTTAACGTTATTGA
GAGTTTCTTATGTTAAAGGTTACTGTGTAGAGCACAGCTGAAAATCACTCTAGCTTTGTACAGTGAGTTTTAAGTTTG
TAATTTAAAAAGATGAATATTTGACATGGATGAACTATTGTATGATGTGTATTTTGAGATATTGACACTGAAAACTGG
AGGCTT

>Sequ11401EST3
TTTCTAGAGGAGTGGAGCATGGAGAACCTGGAGGAGATCTCACCTGCCGCCATCGCAGATGCCACTAAGATCTTTGTG
AACGGCTGCTGGGTCGGAATCCACAAAGATCCCGAACAGCTGATGAACACACTGAGGAAACTGCGGCGACAGATGGAC
ATCATCGTGTCTGAGGTGTCGATGATCAGAGACATCAGAGAACGTGAGATCAGGATTTACACAGATGCTGGGCGAATC
TGTCGACCGCTGCTGATCGTCGAGAAACAAAACTACTGCTGAAGAGACGACACATCGACCAGCTGAAGGAGAGAGAGT
ACAACAACTACAGCTGGCAGGACTTGGTGGCGAGCGGCGTGGTTGAGTACATCGACACTCTGGAGGAGGAGACGGTGA
TGCTCGCCATGACCCCTGATGACCTCCAGGAGAAGGGCGTGGCATACTGCTCCACCTACACCCACTGTGAGATTCACC
CGTCCATGATCCTCGGAGTTTGTGCCTCCATCATCCCCTTCCCTGACCACAACCAGTCTCCCAGGAACACATACCAGT
CTGCCATGGGTAAACAGGCCATGGGCGTCTACATCACCAACTTCCACGTACGAATGGACACACTGGCTCACGTTTTGT
ACTACCCTCAGAAACCGCTGGTCACCACCCGATCTATGGAGTACCTGCGCTTCAGAGAGCTGCCTGCAGGTATCAATT
CCATCGTGGCGATTGCCTCGTACACAGGATACAACCAGGAAGACTCTGTGATCATGAACAGGTCGGCTGTGGATCGAG
GCTTCTTCAGGTCTGTTTTCTATCGGTCCTACAAAGAGCAAGAGTCAAAGAAAGGTTTTGACCAGGAGGAGATTTTTG
AGAAGCCGACACGTGAAACCTGTCAGGGTATGAGACACGCCATCTATGACAAGCTGGATGACGATGGCCTGATCGCGC
CCGGAGTCCGTGTCTCAGGTGAAGACGTGATCATTGGGAAAACGGTGACGCTGCCAGAGAATGACGACGAGCTTGACA
GCACCAACCGCCGCTACACCAAAAGGGACTGCAGCACCTTCCTGAGAACGAGTGAGACCGGCATTGTGGACCAGGTGA
TGGTGACCCTGAACCAGGAAGGCTACAAGTTCTGCAAGATCAGGGTGCGTTCAGTCCGGATTCCTCAGATCGGAGACA
AATTTGCCAGCCGACATGGACAGAAAGGAACCTGTGGGATCCAGTACAGACAGGAGGACATGCCGTTCACCTGTGAGG
GAATCACACCTGACATCATCATCAATCCTCACGCCATCCCGTCCAGAATGACAGTCGGTCATTTGATCGAGTGTCTGC
AGGGGAAGGTGTCTGCAAACAAAGGTGAGATCGGTGACGCGACACCATTTAACGACGCCGTCAACGTACAGAAAGTAT
CGAACCTGCTGTCAGAGTACGGATACCACCTGAGAGGAAATGAGGTTCTGTATAACGGCTTCACGGGAAGGAAGCTGA
CCTCTCAGATCTTCATCGGCCCAACGTATTATCAGCGTCTGAAACACATGGTGGATGACAAGATCCACTCGAGGGCCC
GGGGCCCGTCCAGATACTCAACAGGCAGCCGATGGAGGGACGATCACGTGATGGCGGTCTGCGGTTCGGGGAGATGGA
GCGTGACTGTCAGATCGCTCATGGAGCGGCTCAATTCCTCCGAGAGCGTCTTTTCGAGGCGTCTGATCCTTATCAAGT
TCACGTCTGTAACTTGTGTGGACTAATGGCCATCGCCAACACACGGACGCACACGTACGAGTGTCGAGGCTGCCGGAA
CAAGACACAGATCTCTTTGGTCCGGATGCCGTATGCTTGTAAACTTCTCTTCCAGGAGTTGATGTCGATGAGCATCGC
CCCTCGTATGATGACATCATAGAGTGGCTCGGCCTATCAAACACCTCCCTGCAGAGTTTAAGTGTGAGATCATCAGCA
GGAAGTGAAAGTTTAAGTTAGTTTAGCTTATGTTTTTACAGTTTCATGTTGTCGTCTCACTTTGTTTTTGTAAAAGTA
GAATAAATCGGATTTTCTTGGCAAAAAAAAAGACTAATAATTGATATATCACAAACACTAATGAATAACAATACCAAA
ATATAAACCTACAAAATCCATTCCTCTCTGGAATCATTCATGAGTCC

>Sequ11505SNP3
CTGGGTAGTTTCAGCCCCAGCACAGAGTCCAGGACAGAGGACCAACACCATCATCATCACGTCAAGGCCCCAAGTAAA
GACGAGTTGAATCGCCTCTCTGACAGCCAGGTGCAGGAGTTGAGCAATCAAGTCCGGGTTTATTGCATCATCATGGTA
CAGCCAAAGATCCTTGTTTACTGGGCTACAGCTATGGACACCTGGAGCAAACACTGTGACAAGGCTGTGTTTTACACC
TCAGAGTCCTCTAAAGCGCTCGATGCCATAGACCTGAAAGAAAAAGATGACTGGGCAAGGTTACGTAAAGCTCTGAAG
CATGCTTATGAGAATGCCGGCGACCTGCGCTGGTTCTTTGTGGCACAGCCGACTACTTTTGCCATCATTGAGAACCTC
AAATACCTGGTGCTTGCAAAGGATCCCAGTGAGCCCTTCTATCTGGGCAATGCTATGAAGTCAGGGGAGCTTGAGTAT
GTGGCGTATGATAGTGGCATTGTTCTAAGCTATGAAGCTCTGAAAAGGCTGGTTCACGTGTTTCAGGACGAAGACAAA
TGTCCAGAGAGAGGACGAGCCCTGTGGAAGCTGAGTGATGACAAGCAGCTGGCCGTGTGTCTCAAATATACAGGTGTG
TTTGCAGAGAACGGAGAAGATGCACACGGAAAGGGCCTGTTCAACAGCAAGAGTGTCGACAGCCTGATAACAGACAGC
ATGAAGGACAACCCCACCAATGTGGTGGAGGGCTGCTGCTCCGACTTGGCAGTAACATTCAGCGGGATGTCACCAAAT
CAGATGCAGGTCATGATGTTTGGAGTTTACAGACTCCGACCTTACGGGCACGATTTTCATGACTCGTTAACATTTTAC
CCTCCTGAAGGATCAGACAATGACTAGAGACTTATTCCTTCAAGCTGGATCTTGCATTTTAATTATTTTGCGTCTGTG
ATGGCAAAACTCGGTATTGGGAGATGGTTTTCTTATGCTGTTTGTAAAACTTGATCCTGGGTGGGAACAACACTTTTA
TGTATATTTGTAACTTTTGCACTTGGCACAATCAGCACCACATTTCTTTGTGGTGTAGGTACACAGCTGATGGGAGTG
CCGTCCTTTTGAATGAAGATATGACTGGGAAATACATCAAGCCTGTGACATAAGATTACCTGGAAGTTTTGGTTATTG
TAATATTGTTATGTATCTTAAATGTTTGTCTTTTTGTCGTCTTAAAGCTGCATTAGTTATTGAGTGATACTGTTTTGA
AATCATTTAACTGTTTAACAAATTGCTCGGCCCTCTTCACATTACGAATGACTTTTTCTTTCCTCTGAGAATTGAATC
AAACATGCTCTAACCCAGTTCTAAATTAAGTGCATTTCTTGTCATGTAATGCTTGTTTATCTCTACATATTACGAATT
GGCCATCACTGCTTAATTAATGAATGAGTAAAGTATATTTCTTCTGTCACACATATAAAAAAAAAAAACGGACAGAGA
TGATATTTTAAAAACACCTGGCTTCAGACTCACCATATGATGCCTTTTTAAAAGAAAAGAAAAGAAAAAACACTCGGG
TAATAAAAGTAAATAAAATGAATCACTGACTGACTATTACAGGGTGGGAATGAGGATGAACGAATGCTAGTAGCTT

>Sequ11639EST2, Sequ11639SNP2
GAAGGCAGCAAAGGAGTTGAGCCAGCGTTCTCCTCCCATTCCTCTGGCCAAGGTGGACGCCACTGTGGAGAATGAGAT
CGCCACACGCTTTGAAGTCACAGGCTATCCCACCCTCAAGATCTTCAGGAAGGGCAAGGCGTTTGACTACGAGCGGAC
CCAGAGAGAAGTATGGTATTGTTGACTACATGGGTGAGCAAGCAGGGCCTCCCTCCAAACAGGTCCAGGCAGCAAAAC
AGGTGCAGGAGCTTATCAAGGATGGGGATGATGCTGTCATAGTCGGTGTGTTCTCCGGCGAGCAGGATGCAGCCTATG
AGATCTACATCGAGGCTTGTAATGTACTGAGGAAGACTTCACCTTCGTCACTCCTTCAGCTCTGAAGTGAGCAAACTG
CTCAAAGCGTCAACTGGTCAGGTTGTCATTGTTCAGCCTGAAAAGTTTCGCTCCAAGTTTGAGCCAGCGTCACACACA
CTTTCAGTCAAGGACTCTACGTCAGTGTCAGAAGTACAGGAGTTCTTCAAAAAGCATGTGATTCCTCTGGTGGGCCAC
AGGAAACCAAGCAATGATGCTAAGCGCTACACAAAACGACCCCTGGTGGTTGTGTATTATGGAGTAGACTTCAGCTTT
GACTACAGGAAAGCTACACAGTTCTGGAGGTCCAAGGTGCTGGAGGTGGCCAAAGAGTTCCCAGAATACACGTTTGCC
ATTGCTGATGAGGAAGACTACGCAGAGGAGCTGAAGAGCCTGGGTCTGAGTGAGAGCGGAGAAGAAGTTAATGTGGGA
ATTCTGGCAGATGGAGGCAAAAAGTTTGCCATGGAGCCCGAGGAGTTTGACGCTGAGGTGCTGCAAGACTTTGTTATG
GCTTTTAAGAAAGGAAAGCTCAAACCCATTGTCAAGTCCCAGCCAGTGCCAAAGAACAACAAAGGACCAGTTAAAGTT
GTGGTAGGAAAAACTTTTGATGAGATTGTCATGGATACCCAGAAGGACGTCCTGATTGAGTTTTATGCTCCCTGGTGT
GGCCACTGTAAGAAACTGGAGCCTGATTATTTGGCTCTGGGCAAAAAGTACAAAGGGGAGAAGAACCTGGTGATCGCC
AAGATGGACACCACAGCCAACGACGTGCCAAACGAGAGCTACAAAGTGGAAGGCTTTCCCACGATATACTTTGCCCCA
AGCAACAGCAAGCAGAGCCCAGTCAAATTTGAAGGCGGAGACAGAACAATAGAAGGATTCAGTAAGTTCATAGAAAAA
CACGCCACAAAACTACAGAAGAGAGATGAACTTTGAAACTTTCTCTCCTGACCAGTAGCTAAGAACTCTTACCGCTCT
CAGGCGGCGCCTTGGAGGAAGCTGAGAGGGGCCTCTGGGAAGGTGAAGGGAACACAGTGGAGTTAAACGGTGTTACTG
AGTTGAATGATGATTATTATGTTCAATCATCCTTTTTAGTTTCTGTTCATTTGAAGAGATTCAGCTTTCTTCTGTTTC
TAAAAGCATGTAAGCACTGTTGAAGATTTAAGTCTTTTTAAATAAAACTCAAAATGACATCAGCTTTGTGTGCCAACA
CTAC

>Sequ11670SNP3
GTGTCCAATGAGCCAGGAAATCGCTACAACATCCAGCTGATCAATGCTCTAGTGTTGTATGTAGGCACACAGGCTATT
GCTCACATCCACAACAAGGGCAGCAGCCCCTCCATGAGCACGATCACTCACTCTGCACACATGGACATCTTCCAGAAC
CTGGCTGTGGATCTGGACACTGAAGGACGTTACCTGTTCTTGAATGCAATCGCCAATCAGCTGCGATACCCCAACAGC
CACACTCACTACTTCAGCTGCACTATGCTCTATCTGTTTGCTGAGGCAAACACAGAGGCCATACAGGAGCAGATCACC
AGGGTTCTGTTGGAGCGGCTGATAGTGAACAGGCCTCACCCATGGGGTCTCCTTATCACCTTCATCGAGCTGATCAAG
AATCCTGCCTTCAAGTTCTGGAGCCACGACTTTGTGCACTGTGCCCCTGAGATTGAAAAGCTGTTCCAGTCAGTAGCC
CAGTGCTGCATGGGACAGAAGCAGGCCCAGCAGGTGATGGAAGGCACCGGTGCCAGCTAGCCTGTGCACCAGCCAGCT
CGTAGCCCTTGGGAGAGATGCCGCGGCCTTGACCCACTCCCCCACCACACACATGTTGGCACTGGATAAACTGGCAAC
CACAGTCATTTGAAATGTGGCTGAAAGGACTACTGCTCACGGATCTAAAGATTAGATTTTGTTATCCCTTTGGGGCTG
AATTGAGAAGGAGAGAATGGGTTGGGACATGCTGTTTAATATACTTTTTTGATGCTTTGATGTAAATATTGAAAAAAG
GGAGAACGGGAGAGTAAATAAGTGGAGAACAATTCACATGTCAAATAAAATGTATCCTGACATGTAAAGACATCTTAA
CATATTTTCACTCACTACCAAAGTCCTTGGTTTGCTTCTCTCCACACAGATCCTTTTTTTCAGACCGTTTTTGTTTTG
ATTTCAGAGGCTGAATGGTAGATGATTGAAGAAAAAAGCTGCCTGCTATTTTCATTTGATTCCTTTTTATCCCTGAGT
GGGACAGGAAGATGTAGTTTAGTCTGCCAGGTGGGTGGGGGTCTGCTCCTGGTGTGTTGGTGGAGACAGTTTGCTGAT
GGTTTGTCCAAACTGGCTCTGGACGTCTCTCCCTGGCACTTGTAAAATTGTGCAAAGTGATGGTGACATCGTATCTTG
ACAATTTGTACAAAGATCCAAATTGAAGAACAACAATAGAAGGACCAATACAGCCCATTTTGTAACATTTTGAATGTT
TTGTAAATGTATTGGTCCATGTGTTGTATTTTGTAGTCCTTTCTAGTTTGCCTTTAGTTCTTGCTACTTAACTGCAGT
ATGCATACATACAGGAAATAAAGCATTCAAACTGCAATGCATCTGTGTTCTTTTAGGACTACAAAATTAAGATGATGT
TGCTAATATATCAGGTGTTTCGATTACAAGGTATCTTCTAGTATCTAGAAAACCAACATTCATAGTAAATACTCAGAT
TTCCAGAGTCGGGTGCAGGTCATACTTAAATATTTGAATTGAGTGAAGGTTTATATCTTGTTTATTGGAAATGTGAAA
TGTTCTGATTCATGTACATCCAGAGGACTGGATTACCTGCCAGTCCTATGGTTACAAACCAGAAATGTTAGCTCAAAT
GCCAAT

>Sequ11980SNP3
CCAAATCTGTCACTTCCACTTTTTCGGAGCTTCTCAACAAGCTCTACTGCCAGCTGGCAAAAACCACCCCTGTGGAAG
TGCTGGTGAGCAAGGAGCTTCCTCAGGGTGCTGTCCTCAGGGCCACAGCAGTTTATAAGAAGACGGAGCATGTGGCTG
ATGTGGTTCGCAGATGTCCACATCACCAGAATGAAGATACTGCAGAGCATCGCAGCCATCTGATCAGAGTGGAAGGCA
GCCAGAGGGCTCAGTATTTTGAAGATCCACACACAAAGAGGCAGAGTGTGACGGTCCCTTATGAGCCCCCCCAGTTGG
GTTCAGAGATGACAACCATCCTGCTGAGCTTCATGTGCAACAGCTCCTGCATGGGCGGCATGAACCGCAGGCCTATCC
TCACCATCCTGACCCTCGAGTCTCCAGAGGGACTGGTGCTGGGCCGGAGATGCTTCGAGGTGCGTGTCTGCGCATGTC
CAGGCAGGGACCGCAAAACAGAGGAGGACAACAGCACCAAGGCGCAGAACGGCACCAAGCAAACCAAAAAACGAAAGA
GCACTCCTGCCCCTGACACGACTTCTGTGAAGAAGTCCAAGTCTGCCTCCAGTGCAGAGGAGGAAGACAAGGATGTCT
ATGTTCTGCGCGTTCATGGTCGTGAGCGTTATGAAATGCTAAAGAAAATCAACGATGGTCTGGAGCTGCTTGACAAAG
AAAGCAAAACCAAGTCTAAGGTCTCTGTGAAACATGAGGTCCCTCTGCCCTCCAGCGGAAAGAGACTCCTGCAGAGAG
GAGAGCGGAGCGACAGTGACTGAGAAGCTTGTAGCCGTGATCATGAGGGAACAAACGTCATCTCTTAATCCTTCCAAG
CCATCACGTTTACTTCAGGTTTTTAACCGTATTGGAACCCCAAGTCACCTCCTGCCTCACCTCATCACTTGAGCTGAT
AACTTAACTGCCTGACCATTTACTGTACTGTACTGTACTGTACAGTGTCTTGCTGTTTATGACATATGCAAATGAATA
AAGGAGAGCAATTCATATTGTAGGCAGGCCTTGCCTCCTCCTCACTGCTCTCATTGCATTAGTAGGTCCTGTGAAATA
GCTGTGAGACCTTAATCTTTCTGTGATTGCAAGAAACAGGATGTCGGGGTGGGACTTAAATTTGGGAAGGACTGGATT
TAATCAGTGTTAATTTTGGCTGGGATTTTAATTTGAGTTTCATTGACTCCGTTTTTAAAATTTGTGTCAGTCTTGTCA
TTTATGATGCATTACATTTAGTCTATTTAGTTAGTAGATGAAAACCAAAGAGATTGTTTCTTGGTTTTTCAGTGACAT
TTATTTTTTTATGTTTGTCAGAATATTGATTATTATCTGAATTGAGCAGTTTTGTCAGTTGAGTCCCGACTGAGGGAG
ACGTGTGACTGCAAAGGATCATAATTGTAACAAGTCACTGCCATTTATTAGAAAAAGTGCAAAATCACCCTTGTGAAA
CTTGAATCGGTGTTTACCTCCAATAATGTTGCTGTATTACTGCACCACAGCCCAACACACATTTACTGCAACAGATCC
CAACACGCAATTACTGCAGCACAGCACATACAATTTTACACATTTTTATGTACCTGTATGAGGAAATTCAGTCCATGT
GTTGCTGTCCAGCTGTGATTAAATGTGCTGTGCAAGCTTACAAGACATTTATAGATAA

>Sequ12149SNP3
GTCATTGTATTTTTTCAGTTTTCTGATGTATTATAAACTGATGATTGACTGAGAAAATAAAAGATGCCTGCTTCAGTG
CAGCTTTTGCCTGACGCACATCTGTGCAGGTTGTTTCCAAAGGAATTGAACTGGGAGTCATTGACACGATGTGCAGAT
CTTTATTCTTTTTCCTCCCTTTTCTTGCTTCCAGACATTATTTCAAACGAGCAGGACTGGTTTCTGCATGGTGTAAAA
TCTAAAGGGAAATCCATCCTTAAAATCTTCAGTCTGCTGTTGCTTGGAGTTCTCTATGTTGTTGTTTTCATCTATTTT
GTTGCTTGGTGATGTTTTTTTTGTTTTTTTTTTCTTACCCCAGAGGAGAACCAGCCAAACTTAGACGGCATGACATCA
TTGAGTCCAGATATTTCCAGGTCCACATTCTCTCTGGGGTCATGGTTGGAAACGTACAGTAGGAAATCACTCACTCCA
GCAGAAGTTGATAATGATTAAGGGGAAGGACACTAAAAAGTCAATAACAACACGTCAGCAGAAACAACATGCTCTGAA
CATCACAGAATAATGATTTAAGGGTGGACTATTCCTTTAATTTTTATCCCAGACGCTAGATTTTTGATTATGTTTGAC
ATAAACAGCCTTTTACATTCAGTGATTATTACGTTTCTTTTTTTTTTTCACCACCATGACAATGACAATGTGGCACCT
CTGTCCAGACCAGCAGACACCCGTCCCCGTCCCAGGGCTCCATGGCTCTGTAACATGCTGGCACTCTGGCCACCGGGC
ACTATAGGTCTGTATAGCCTTGTTATTTACTACTGACTCCTCAACTCTGTACAGAAGAGGAGAATATGATCGCATGGA
ACCTTGTTACTGTAATAATAACCTATACAGTATATAATATTTAATTTTTGATAACTTTAATCAATTAAATCTACTTAC
CAACAACAGACAGACTTGCGAAAAACCATTTTTTTATGTTTCTTTTTGAGCACATGTTTTTTTTTGTAGAATAGAAAG
GTTGTTTCTTCATTGTTCCCGTTTGGTTTTTACAGTTACTGGGGTTTTTTGTGTCTTTTACTGTAGGGAAAAGAATAT
GACGTTATCACTTTAAAGATAGACAAGAGTCATGCTTACGCTAATTAAATGATGATGACCCTGATCATGCCAGTGATA
GTTATCCAGTGTGAGAATGTTCAAGAATTTGTGAATGAACTGTGTCTGGTTTACACTGAGCGATTAGCAGTGATGAAT
CCTGGCTTATGAAGTTTTATGACTGTATATTGAAGGGCTTGGTGAGGATGCAGGCTCTAGAGACCCGTGACAGTCCAG
CGCGGTTCTCAAAGTCATCTGCCACCATAAAAACCAACCTCCCTTCCTAACAGTGAAGGAGTGATCGGTTTCATATCA
ATAACATTTGACCAGTGGTTTCCTTTATTCATTGTCTGTATAGACAAGTGAACAGTCTCTAGAGCCTGAGTTCCACCT
CTGAGAAATTAAAGGATTCTCTGTATTGCCTTGTTAATATTAATTGTGGACACATAATTTTATAAATGACAAAGACGT
GGAT

>Sequ12305SNP3
TCCCCCTCCAACAGCGCCTGCCCTCCCCTGGAGTCTGCAGACCGGGGTGGACTGCACAACTGGTGGAGTCCTAGCATT
GACTGCTCTGCTCTTCAAAATGGAGGAGGCCAACATCGCCAGCAGAGCCAAAGCACAAGAGTTCATCCAAGCAACCAG
CCAGATTCTCTCACAAGCCAATCAGAGCCAGTCTCAGCAGCACGCTCCCCCTTCCTCTGCCTCCTCCTCCTCCTCCTC
ACAGATCCCTCCCCCTCCTTCTATCCCTCCGCCTCCTTGTCTGAGCCCAGCCCAGTTTATCCTCCACAGCTCCCTCCC
ATTGGTTGGATGCACCAAAACCCCACCCTCTCACCTGCACCCTTCCATGAGTGGCGGTTGTGCACAGACCCCACCCCC
CATCATGCCAGTGGGACTGTCTGGAGTGACTGGGAGTTCTGGTGACACTGGATGGGACAGTGAGAACAAAGACCCTGA
CAAGTACCTGAAGAAGCTGCACACCCAGGAGCGAGCAGTGGAGGAGGTGAAGCTTGCCATCAAACCTTACTATCAACG
CAAAGACATCAACAAGGATGAATACAAAGACATCCTCAGGAAAGCTGTGCACAAGATCTGCCACAGCCGCACTGGAGA
GATCAACCCGGTCAAAGTCAGCAACCTTGTGAAGCTCTACGTGCAGCGCTACAAATACTTCCGGAAACATGGACGTAA
AATGGATGAGGAGGAGAGGGATGACAGGGAGCCAGGAGTGCTCCACTCCTCTGCCTGAGAGGAGACCACTCCGCCTTG
TGCTCAGACCTGGGCCACCCTCTGATTGTTTGTCTGATATTTTTATTGCTTTCATAGCCACCTGTCTGTTTTTATGTG
ATCTCCCTTCTCTCTGTGATCTCACTGTAAATATGTTTCATTTAGAAAATGTATCACTTATACCATGTTGAAGAGGTG
GATGCTGTCATGCTCACCTGTGTGATCTGATACAGTCCAGTACTCCAACCCTGCAACACATACCACCTGTGTGAAGCG
TTTAGTTTTGTTGACTGTCTCATTAGAAGCTTCACAGCGGTGGTACTGATCACAGGATGTTTCTCGGCAGCAGTGGAT
TAGGTCTCCATGCTGGTTTGTCCTCCCCATCCCCATGTATATGCGAATCCCAGCTGAGGAGAGGAGTAATACCCAGTT
GAACACATTAAAATATATTGTACAACCCTGAAGGACACTACACCCCGTCTGTCTCAGACAGTATGGAAACAAAGCTGT
CATGGTCCAGACACACTGACCTCTCAGCTCTGTCATTCTGCCGTCTCTGTGTTGAGTGCATGTGTTACTGAGGTTGAG
AAAGTTGCTAAATCTCTTTATAAAATGACTTGACCAACAAAATATAAATGTTGCTTGAAAACCAGTT

>Sequ12349SNP3
GAAGAAAAAGAGCCGTCATCGCTTCCGCAACAACCGTTAGTGCAACAACATGGATGTTACAGCTGAAATTAGTCAGGA
GGATGGATTTGCTGGTGAAAGCGGTGCCGCCAAGATAAAAGAGCGACTTGAGAAACGGAATCAGGCCAGAATAGAGGA
TGCCGAGCGGCGTAAAGAAGAGAAAGAGAGCCAGTCTGTCGCCGAGGAGAAGGACGAGTTTTTCTCCAAGACTTTCAG
CAGGGAGCGGGTGTGTATCGAGGAGCTGCTGTCCAGCTGCTCCGGAGCTGACCGAGCCGTGGTGACTCAGAGGCTGGA
GGAGGCGACGGTCAAAACCGTGCAGCTCCAAAAGTTTCTGAATGACAGCGTGTTGTTTCTGACGCAGTACGAGCTGAG
ACAATCCCAGGCAGCTCTCCAGAAACTCCAGACATCCCTTGCGGAGACCAGAGAGACGGCTCTGCCCAAGAAGAAGTT
TGCCTTTCGAGCTCGCACTAAAGCTGCAGATAAAGTCTCTGCACCAGTGTCAGACACAGCTCAAGCTGATGTTGCCAC
ATCTGCACCTGCATCTGGTGAAGTGGATGGAGATGCAGCGTCAGAGCAGTGTGGCTTCTCCAACATGGACAATGAGTT
TCTGACCAAAACATCTGAGGAGATCCAGAAAAGAGACATGCTCTTGACTCACCTGTCCAACTGCAAGGTCCGTCTGCT
CGGTTCCCCCAGCACATTGCACCTGAAGCACATCGACAGCTGTGAGATCCTCTGCGGGCCGGTGTCTAGTTCTGTATT
TGTTGATCATTGCAGAAACAGTACGCTGAGCTTCCCCTGTCAGCAGCTGCGGACCCACAACACCAAAGACACACAGGT
GTATCTGCATGTCACCAGCCGAGCCATCATAGAGGACTGTCATGGAGTGAGTTTCGCCCCGTTTTCCTGGTCTTATCC
CACTCTGGAAGAGGACTTTAGTGTGTCTGGCCTAGACCAGGACCGGAACAACTGGAATCAGGTGGATGATTTCAACTG
GCTCGCTGCAGGAACACCTTCCCCCAACTGGACTGTCATCCCAGAACCAGACAGGAAAACCAACTGGGATCCTTAACT
TTAAAGCCTTTATTTGTCAAATTAACACCTGTCAAACCAAATGACATGTTGACAGCCTGAATACACATACGTAGTCTT
GTCCATAATCTTGAATTTAAGGTGGTTTAATGAATTTTCTTTGAAGTGGCTGCTCTTTTTTTTATTGATAAACTATAA
CAAACAAAATGTAACATACATAGCCCACACACACAAATACACACACTCAAAAAGGCAGAGAACAAAACGAATAAATGC
ATCATATTCTCTATCTCAGCGCTACAAAAATGACATTTTTGATCCTTAACCGTGTCTGAAGCATGTGTCTAAACACAA
GTTTAATTGAAAATACTCTGAACTGGTTGTCATATGCTGCTACTTGTCCATATGGCTACAAAAAAAGGGCACTTTGTA
TCATAAGGTTTAAGCTACATTAATAAATTTAAATAAAAATCCACCTGTCTTATCAGCCTGAATAACTGTGGGGCTTTA
GTACTTAGGAGCATCACATCTCCCCTGAGGCAGAGCATCCACACTCTTGCCGAAATTAAACTCCAAGCTTTTATTCTT
CTGCTCATGATTACACTAGTTCACACAGAGAAAGCTGCTCTCCAACATTAGATTTTATTCCCATTATTCTGTGATATT
CAAAAAATTCAAATCAGCCATATTGGAATAGTTAACTAGTGTGAATTTACAGGCTTTCTGGTAGAGCCAAGTTGATAT
TACTTTATTCCAGAGATATATGTATAATGTATTGTGTTAGAAACCATTTATTAAAACTGT

>Sequ12494SNP3
TGTCCTCAACAACTGTGATGAAGGGCAGCCATCTTGTGCCATTTTGCAGTACATGTATAATTATTAGAATATGCATTT
TAGCTTATCTGTTCTCCAACAATACGATACAATACACTTGGAAAGTAATTTTCATATATAAAATGAACTGTCTGAGAA
GACGTGTGTTTCATTGTGCGGCAGGTTGTATTTTCCTTTTGTAACTGTGTATTGATGCAGGGTAGTAACAATGTGTCA
AATCTTGTTCTTTG

>Sequ12653SNP3
TAGCAGGTTTATTGGCTGGATAACGTCGTTTCTGTAAAAAAAAAAAACTACGGTACCCATCATGCCTCTCGGGTGCGT
GATGACGCCACAGAACATCAACAACTCACATGTGGCGTACGTGAGCCGTTGCTGTGCCGTGCTAGCAACAACTGGGTA
ATGTGGAGCTGACGTCCCAGCTGAAATACCACGTTAAAATAAATATCACACAGCTACCTACACGCGTTTTGTACACAG
CTTAACCTGAGCCAACATGCCTCCGAAGAAACCCGCCCAGCCTGCGGGAAATAAAAAACTCAAGAGAAGAAAAAGGAA
AAGATAATTGAGGACAAGACATTTGGCCTCAAGAACAAGAAAGGGGCCAAACAGCAGAAGTTCATCAAGAACGTTACT
CAGCAAGTGAAATATGGACAACAAAGTGCAAGACAGGCTGAGGCAGACAAGACTGCTAAGAAAACTGACAAGAAGAAA
GAGTTGGACGAACTCAACGAGCTGTTCAAACCTGTAGTTGCTGCCCAGAAAGTCAGCAAAGGTGTCGACCCAAAGTCC
GTGCTGTGTGCATTCTTTAAGCAGGGCCAGTGCACCAAAGGTGACAAGTGCAAGTTCAGCCATGATTTGTCAATGGAG
AGGAAATGTGAGAAGAGAAGTGTCTACGTGGATGAAAGAGACGAAGACCTGGAGAAAGACACAATGGAGAACTGGGAT
GAGAAGAAGCTGGAGGAGGTGGTCAATAAAAACACGGAGAAGCTGAGAAGAAAAAAGCCAAAACACAAATTGTGTGCA
AGTACTTCTTGGAGGCCATAGAGAATAATAAGTACGGCTGGTTCTGGGTGTGTCCAGGGGGGGGCGATACTTGCATGT
ACCGGCACGCTCTGCCGCCCGGCTTCGTACTCAAGAAAGACAAGAAGAAGGAGGAGAAAGAGGAGGAAATTTCGTTGG
AGGAACTGATAGAAAACGAGCGTGCAGCTCTGGGTGTCAACGTGACTCGGATCACCCTGGAGACTTTCCTGGCTTGGA
AGAAGAGGAAAAGGCAGGAGAAGGTGGACAAAGCGAGGGAGGAGCTGGAGAAGAAGAAGGCCGACTTCAAGGCTGGAA
AGTCGCTAGTAGTGAGCGGCCGCGAGGTGTTCGAGTTCCGTCCAGAGCTGGTCGACGACGACGACGCTGAAGCCGACG
ACACTCGATACGAGAGCGAAGAAGAGGAAGACGACGAAGTGATTGATACCACAGAGGTCCAGGACATAGACTTATCTC
GTTTTGTTCCACAAGAGGTCGACAACACAGGCATTACCGTAGCATCCGTGGACCGTTTTACCTCGAGGAATAAGAACC
GTGAGAACAGTGAACCGACAGAAGAAGACAACGAGGAACAGCTGAACGGAGCTTGCGGCGGCGCTGAGGCCAATGGGC
TTTCAGGAGCAGAGGGGGGAGGGGAGGACGGAGGAGGGGAGGATGAAGAGGATGAGGAGGAGGTGGAGGAAGAAGAGG
TTCCGGTGGACGAGAACCTGTTCACGGGTGAAGATCTGGAGGAACTGGACGAGGAGCTCAACACACTGACGCTGGAAG
AATGAGAGGGGGCGCGCGAGGTGGAGGTTAGGACGGACTAAGTGACAAAGGGATGGACCGATGGATGCATGGAGCTTA
ACAACAAAGAGACCGGATGTTTGAATGATTTTAAATTTCAGAGACTCCAGTAATAAAGCCTGATTATATCATTACATC
ACTGCCTGTCTCTGCCCCCTCTGTTTTCTGTATCGCCCCTCTCACATCCCAGACGTTCTGTCCATCGGGTGTCCAAAT
TTCCACAGCAGGGCCAATAAGGGTCAACCAGGGCCAACTCCACATGAATGTCGCTTGTCACAATTCCTAATACGAGAG
AATTCGGCTGCTGCTGTTGTGCACTTTTTGTTGCTCAACGCTGGCTTACGTTGAAACTGAAATTACTTTAGATAAAAA
AATTAGGCTTCGAGTGCTTCTCTACCTGATCACTATTAAATATCGAACAGGGTGATTTGCTGAGTGAGACATTACAAG
GGCGTAAAAATCAAAGCACTGCTGGAAACGGCTTCAGTAAACTTAATAACATTATCGATGGATGTTTAAAAAAAGCTT
TTGTAAACTGAATTTATACAAAGTGGGAAATGAACAGGAGTGATTCTGTGGTAAGAAATCTGTTCAGAGCTTCGCCTG
CTCTCGTACCAAAATAAAAGCTGTAGTTTGAGGTTGTCAGCACTTTCATTATTCCAGTAATTTGTTTTCCCTCAACAG
ATCCATTATTTCATTTAAGTGAACTTAAAACTGTTGAAAAAATTTGTGTAACTCTGTCGAAGGAAAAGTCCAGACCTA
GCTTCTGAAATGTCAGGGGTGTTTGAGACCTGATTAGTGCTGTAATTCAGCCCCAGCAGGGAAACCAAATGATCCCAT
TGCCAGCCCAGTATTGTAAATAATGTTCTCATGAGTTGCCTCCT

>Sequ13256SNP3
ATTGAGGGAATCACTCAAAATCTAAAGAAATACTGTCACACATAAACGTTTCAGATCGGTAAATGGGGATTGAACACA
TGCTTGTGTGAGAATTGCATTGGTTGCAGGGAGAGTAATATCACTGTAAATATCTTTGAAATTCTAAAATATAATTGG
GAACTGGGACCCTTGGTGAACACTGTAGACTGTAATAGGATATAATTCAATATTCACGACGGAAATCAGACTATATTA
AGACCTTTACCCACACTTACACAATGGAACATACATTACGTTTAATCAAATTGGAACAGCTTAACAGACACAGTATCA
TGTGACAGAAATTTGGCTTGAGAAGAGCAGAGCTTTTTTTGCCATCAGTATAAAACAAAGGACTATAAACCTTGGAAT
GTAATGCTTTTTTCGTAAGATGTCTGTGTATAATTTGCAGTTACCTAGCAGGGTGTGAATAGTGCTAGTGTAGTGCGG
ATGGATAGACAGAAATTCACATCAAAACGAACCTCTGTGGAACCCTTACCTCTGATTTTTATGTTTATGGATATTATC
ATATTGACAGTGGCCTTTTCAATAGTTGTTTCGATCTTTCCTCCCTGCATAATGTTTGATTTGACCAGCTTCAGTCGG
TGATCACAATTTATGAAGATTTCCGAAATGGTATTTTTCTATGTGTGATTCTGAATCAGAGTTGGTGCTCTTCAGTCT
AAATAAAGTATGTGAACTTTGGAAATGTGACTTGCATCAGAAGATTGTGTAACTTTCTACGCCTCCGCCCGGCGACAG
CCAGGGCTTGGAGGCATTATGTTTTCAGGTCATCCATCCATCCGTCCCATTCTCGTGAACAAGATATCTCAGTAATCT
AATCTATCTCAAGGAATTTCTACAAATTTGGCATAAACATTCACTTGGACTCAAGGATAAACTGATTTGATTTTGATG
ATAAAAGGTCAGGCTATACTTGAATTTGCTTGGCCTTCAGATGAAAAAATGACCCCAGTTAGCACATGTAAGGATCTG
CACTGTCCTACTGCAAAAACTGACCTGACGTCACACTGGCT

>Sequ13415EST2
CACACTCGTCACAGGATTTTCAAGAATATTTGGTTACCCTGTCGGAATCATTGGCAACAACGGAGTCTTGTTTTCAGA
ATCTGCAAAAAAGGGGCGCATTTCATCGAGTTATGTTGCCAGCGAAACATTCCACTTATTTTTCTACAAAACATAACA
GGCTTCATGGTGGGCAGAGAATATGAAGCAGGAGGAATTGCCAAAGATGGAGCCAAGATGGTAACTGCAGTCGCCTGT
GCCAATGTACCCAAGATTACTGTTATCATTGGAGGCTCGTACGGCGCAGGAAACTACGGCATGTGTGGCAGAGCCTAC
AGCCCACGATTCCTGTACATGTGGCCAAATTCCCGTATCTCAGTGATGGGTGGTGAGCAGGCAGCCACTGTCCTGGCC
ACCATCACTAAGGATCAGAGGGCGCGAGAAGGAAAGGAGTTCACAGCAGAGCAAGAGGCTGCCATGAAGGAACCAATA
GTGCGGCGGTTTGAAGAGGAAGGCAGCCCATACTATTCTAGTGCCAGACTGTGGGATGACGGGATTATTGATCCTGCT
GATACTCGTCTGGTTTTGGGACTGAGTATCAGTGCAGCACTGAATGCACCAACAAAGAAGACACGGTTTGGAGTGTTC
AGGATGTAATGTTCATTCGGCCTGTACAGGTGTAGACCTCAGACTTTCCAGAGATGTACTGTAGTTATTCTAGGTTTT
GTAGTTTTATTTCTGATATTATTAAGAATAACAAACAGAGAAAAAACACTACGGAAAATTAAAATCACAGGAGTGGAA
TCACACAATCTTGCATATTAGTTATTGTGAGACCTTTCCACTGTCACGTTTGTGCCTTAACTGTTACTACACCAGTGT
AAATGCAACTGTAGTTAAAAAACTGTTTCCTTTGGTGGTTTTTTTTTTTTTTAT

>Sequ13465SNP3
GCTAAAGAGGAAACACTTTCATAACATGGCGTTGGGACAAAGCACATATTGTACTTAATTAATGGATGAGTGACATCA
ACATTGTTTATCTACAGGAAGCGCCGGCAGGGGAGACAAACTGTAGTACGACCTGGTTTCAGTGGTAGTTTGTACATT
TTCATTAACCGGAGTCGAAGATGGCCTCTGCTCTGGCTAACAGAGCAATGGGAGCCATTGTTGGATCAGCTGTTGCAG
ATGCAGCAGCGCAGCCCCTCCACTGGGTGTACGACCTCCAGAAGCTGCAGGTGATTCTGGCTCAGGATCCAAACCCCG
AGTTCCGCTCTGAGTCCGCCAACCCGTTCTACAGGAGGCAGACGGGCCAGCAGAGCTGCTATGGAGACCAGGCGTATG
TTCTGCTGGAGTCCCTGTCTGAATGTGGAGGTCTAAATGTTGAGGATCTGAAGCAGCGCACGGTGAAATTCTTCGGTC
CTGGATCAGAGTATGACACGCCTATCAACGATCCTTACAGAGAGAGAGGAGGGCCAAGACCGCAGCTGCCCATCGAGG
GACCATGGAGACATGCGAGTTTAAAGGGCTTCCTGAAGAATGTGGACGCAGGCAAAGAGGAGACAGGATGTGAGAACG
ACTGTCAGATCGATGGAATAACCAAACTGGCTCCTGTAGTGGCTTTTTATGCAGGAAAGCCTGACATGCTGGAGAAGG
TTGAGCAGGCCACACGTGTCACCCAGAACAACGACGCATGTGTGGCAGAGACTCTGGCAGCAGCAAGGTTCCTGGAGC
ATTTCATCCTGAACGGTCCTGACCCTAAAGCCTTGGACGCAGTGCTCGATCAGCTCAGTGACAACAACAGAAAGCAGC
CGCAGGACCTGGACAAAGCAGTCGCCGGGCACTTTCACCAAGTGAAGGAGAATCTCTCCAGGACTCCTCGGGAGCTAA
TCCCCACTGAGTTTCCAAACACCTGAGGTTTGCCAGGTGCGTTCCAAGCAGCGCTGCATGGAGTCCTGACAGCAAAGC
AGTATGAGCAGGCTGTCAGAGATACCATGAGCTGCGGGGGATGCACCTGCAGTCGAGGATCCTTCATTGGGGCATGTC
TTGGGGCACAGACTGGACTGGAGGGAATTCCATCTTCCTGGACGACCAAAACCCTGCGATACGACTCAGTGTTGGCAC
ATGCCAAGAAGATAACCAAACACCACCAATAGTTTCCTG

>Sequ13476SNP3
ACAACCAGCTATTCTTAAAGGAGCCACGCCACTCGTGTAACATTATCTTCGTCAAAGAGACATAGTGCCAAATCGCCG
GCACGCATGAACAATACATTAGCATAACTTAATTTCAGGCTGGATCATGTGTTCAGGGGAGGCGGAGTTCACACCCTG
CACAAAATGTATTTGTCATCTTACTGTCATCACTCAGGTTGTCCATGAGAGAGAGAGCGAGAGGACGAGAGACCAAGA
CCCTCTCTTTTCTTTTCTCTTTTTCTTTCTTGCTCCCACATAGAGTAACGAGTGACGACTGAGAAAGACGAGACACCA
ACAAACACTCAGCACTCCACCGGGTGAAGACAGGACGGTCTGACGGTGGTTAAGATCGTAGCGTCTTCACTATCAACC
GTCCACTCACAAAGAGCTTTTCTAGTCATACAGACCAGTCACAGTACATTCACCTATTCCCTCACACACACGTCATCA
CACCCACACACTGATGGAACAGCCCGAGGACACTTCAACACGTGGACCGGAGGAGCCGGGGATCGAACCACCGACCTT
GTGATTAGCAATACAACAACACCAAGAAAATAATCTTTAACTAGGTGAAAAACATTTGAACTGTCAGAGTGTAAACAC
CAACGATGACTTTATAAAGACTCATGACATATAAAACTATCTGCTTTAACTTTTCATTTTTCAGCAGCTGCAGTTCAA
ACCTCCACTGACATTAAATCTGAGCTGCAGACGTGTGTTTGTGTATTTACACACTTTTTCCAGGACTCTCAGCCTCTC
TAACAAAGAGGCCTGTGTTAAAGGCCAAATGAAACATCGTATGAAACCCTCATTCATTTTCCTGCACAGAATTGACAT
TAAGTCTGGATGTGTGGGCGAGGCTGGGCGACGCCTCTGAAGTGTGTCCTGTTCGTCCACAGAGACACGTCATTGAAC
TACAACGCAGTCCAGGACAATGTCCAGAGGTTTACTGGACGGATCAGTTCCTGTCTGTGGACTGAGACTCCACACAGG
CCTCTGATGTCCCGAATGCTGAGCATCAGAAAACTCATGATCTTGTGTTTTTCATGTCAGAGCTGTGGTTTTCCTGGA
GCACCGCCTGGTGGACGATAGAGGAACTGCAGCAGTGGAGGCTGGTCGTCTGATCGCTTTGAATATCTGACATTGTGT
TAAATCTTTATGTTTCAGCAGCTCATCAAGAACTGAGAGCTGAAGCTAACGGAGGAAATACTGAGAATAACCACCTGA
ACAGGTGAACAGGAACCACCACCCGCAT

>Sequ13632SNP3
GCCACTGATGTTGCTGCGCTGAAGCTGACACGATAAAGGTGAAATCCCAAGATTCCTGAAGATCTTGTCAGTCAACTC
CCACATGGCAGTGAACCTCTCAACTGAAGATTAAACCACCTTCAGCTCCAAAGCATTGCCCACTTAGATGCCGCTCGG
TCTTCACCTTTTCAAATGGCTTGCTGTCTGAAGGACGAGCTCCTCTGTTCCATCTGCCTCAGCATCTACCAGGACCCG
GTCAGCTTTGGCTGCGAGCATTACTTCTGCAGAAAGTGCATTACCGAGCACTGGAGCAGACAGGAGCCTCACGGAACA
CGAGACTGCCCAGAGTGCCGGAGGACCTTCACCGATCCTCTCCTCTCCCCGAGTCTCAAGTTGTCCAACATTGTGGAG
CGCTACTCCGCCTTCCCGCTGGACGCCATCCTTAACGCTCAGAGGAGCTCCTATCCCTGCAAGGACCACGAGAAGGTC
AAGCTCTTCTGCCTCACTGATAAAAGTCTGGTGTGCTTCTTCTGCGACGAGCCAGCACTACACGAGCAGCACCAAGTG
ACCACTATTGACGAGGCTTACGAGGAGATACAGAGGGAGATGAAGGAGCAGCTGGCCACTCTGCAGGACAGTGAGAAG
GGACACACTGAGGCCCTGCAGCTCCTGCAGAGACAACTCACTGAGACCAAGTCCTCGGCCAAAAGTCTGCGCGCAACC
ATCGGAGATGCATTCGAGCGCCTTCACCGTTTCCTGCGCGAGCGTCAGAAGAGCATGCTGGAAGAGTTAGAGATGGAT
ACGGCCCGCAAGCTCGCCGACATCGAGCACAAGATCCAGCGCTACAGTCAGCAGCTGCGCGACGTCAAGGAGGGCATC
CAGATCCTGCAGGAGCGCCTTAATGAGACGGGACGACACGACTTCCTGGAGGGAGTAGCCGTCACGTCTGAGAGGATT
AAAGGGAAGATACATGAGACCAATCTGACGTACGAAGACTTCCCAACATCCAAGTACATGGGACCCTTACAGTACACG
ATATGGAAGTCGCTCTTCCAGGATATTTCACCAGTGCCAGCAGCGTTGACCCTCGACCCCATCACAGCCCACCAAAGA
CTGATCCTTTCAGACGACTGTACCATCGTGGCCTACGGGAATCTCCATCCGCAGCCGCTGCAGGACTCCCCGCGCCGC
TTTGATGTGGAGGTGTCTGTTCTGGGCGCAGAGGGCTTCGCCTCAGGTGTCCATTACTGGGAAGTGATGGTGTCGGAG
AAGACCCAGTGGATGATCGGTGTGGCCCACGAGACAGTCAGTCGCAAGGGCAGCATCCAGATCCAGCCCAGTCGCGGC
TTCTACTGCATCGTCATGCACGACGGGAACCAGTACAGTGCCTGCACCGAGCCCTGGACCCGTCTCAACGTCAAGAGC
AAGCTGGAGAAGGTGGGGGTGTATCTGGACTACCCGAAAGGCCTGCTCATCTTCTACAACGCGGATGACATGTCCTGG
CTCTACACCTACCGAGAGAAATTCCCCGCCAAGGTCTTCCCCTATTTCAGCCCCGGCCAGAGCCATGCTAACGGCAAG
AACGTGCAGCCATTGCGAATCAACACTGTACGCCTTTAAGAGCTACACACATCTGTGGTTTTGATTGTCCTGACAGAT
GAATCCATCGTAGGTGAAATCTCAGGACTTAAAGAAAAATAATTAAATAAATCTGTTTAAAAAAAAACGTGTTTAATA
GGGTGTTTGAAATGTTTATTCCTTCAGTTCGGTGTTAAGAATTGTGCTTGATGTTGCTCTAAAAACCTGTGCTTGAAA
AGATGACACTAAATGGAGACAGCGGGACCCCTGGGCTCCCCTGGGGTCTGTGGCAGCGTGTTTGAAAAAGAAAAAAGC
GATACCTGCGCTCAAAGTGACTCCCAGGCCCACCTTGCACACTCCCAGGGGACACCACTCCCATTATTAAGCTCCAGG
CTTGTCAGACCTGACGTCCCCTCTCTGTCCAGGCAAGCAGGACACTGAAGACTGGTCACGTGTCTTCTCTGTGCTGTA
CAGTTGGCTTAGCTGTGTTTGTGGAACGTGATTGACAGCAGTCTGCTCTTTATTTTGAACTTGTCGAGTCCGTTGGCA
CTTTTTACTCTGTCTGAGACCATGAACTTGGTTCCTCCTGTAAGACGTATCCACATCTGTCTGCTCAGTCTTCTTCAT
AACAAGGAAAGCTCCTAGTCCATGTGCACCAATGATTCTGCACGTTACCTAAGACAAATTAAATCAATGAAAAAGTAG
TTCATAGGCTGCCCATCCCCATCCACTGCCCAGTCCTACCCATGCGTGATAGGAAGCTCTGTTTGGAGGTGGAGATTG
TCAAGAAATATGCTGCTATTGACGGAGTAAGGACTTTCATATTTGTATTTAGCATATTAAGGGTTAAAAGGTTTGAGT
CTTTCATCATGCTGGCTCATGTTTTGCCTCCTTTGGTTTTGCTAGCCTTCAGCATACACTACACATGTGGGCTTTTTG

TCCACTTTAAGTGGATTGGATTGGATTCAAGCACATTTCTGCTTCCTGTCATGATGTTTTATCATTTTGTCTTGGAAT
CAAAAGAATATGAAGTCATGCTGTCTAACTAGATATCCGCATGAGGCAGAGACCTCTGGTTGTTCTCAGATATGAAAA
TCTGAAAAGTTAAAGATGAAAATTGCTTTAAAAGAAAAAGGCTACAGAGACCAACTGTACTCCATTTATTTTAAATCG
TATCTTTTATGTAAATGAAGTCAGTCTGTATGTTTTTAGAAAGTGGCAATTATCAAGGAAAATGAAGAAGGATGGATT
GACTTGAGCACAACTTATGTGATCCTGGCATCGCTTTGGCTGCTGCTGTGTTACATAATGATGAAATCACTGATAATT
CCTCTGAGGCTCAAGTCATCTTTTATTCTTTGCCCCACGTGCCTCAAGTGCTCTTGTTTATCATTAGTTGCACACAAG
TCCTGCAGAATCTGCTCCAAGAGGCTCTTGTCCCTCCGCCAAGTCCGGCACACTGAGAGATGTGTGTGTCTTTGTTTG
AGCATGTTCACGGGTATGTTTGAGTGGCGTGCTTCAGCTGTAATTTTAAATCCAGCTTTGGATGTGCTTGATAAAGCC
CATCTGGCCCTCAAAGCAAAACCATGCAGTGGCCAAAAGTGTTTATTATGAAATTCTTTAGAATCAGAGAGAACTTGA
CAGATCTTTCTTTGGGAAATCTAAAAAAAAGGACAAACAAAAGGATCACCTCACCCTTCCTTTGCCACTGTACATCTTC
ATCCGTGCATTAGTGTATGTGTAGTCTGTGTTGTCCATTTAGAAGAAAATTGGGGCAGTGCCCTTGTTTTTGTAGGAT
GTGATTCCGGTATGTGCAGTATGATGCAAACTAGGGTCAAATTGTGAGCATCTCCAAACTAGTCTTGGTTGTTTGTTT
TAACTTGCACAGATCTGCAGTGATCTGATTAAAACCAGGCACAGCTCATTGTCTGTCAGTTTTCTGGGATTTACTCAT
TAAATTTATCCAGATAACTTGATGAGCTTGCTTGCTGAAAAAGATTTTATTATTAATGTGTGGTTTCGAGTCCCGCAT
CGGACAAGCCTTTACCTGTATGTCATTCCCCTTCTCTCTGCATCCCCATTTCCTGTCTCTCTCTACTGCACTATCAAT
AAAGGCACAAAAAGCCTGAAAAATTAATTTAAAAAGTTAATTAATGTGTGGTTTAGAGAATCAGGACTGTATTCAGTT
ATTTTTCACTTTTATCCTCTTGATCTGTCTATCACTATATAAAATGTCCTTCCAAAGTAAATCCCAACCTGCTGGGCT
CATGCAGGTTAAAACAAATGTTAAAATTTGACCCCAGTTTGCTGTGTTGCAGACCTCAGTGCAGTCCGCCTCATTAAG
ACTTTCTGTTTGAATAGTAAGAAGGAGATTATCGTGAGGTGGTGTTGGGTTACTGGTGATGCTGATGATGCTAATGGT
TAAAATGATTTTGATGATGATGATGATGATGATGATGATGATAATATAACTATGACACTTTTAATGATTATGTCTTTT
GATGATGGCCGCTGTGCACCCATCACTGTATGAACACACTCTAGTGTGCTTTTGCTGCTGGAGGTTTGTGGGAAATGT
TTTAATGTACAATAAATTACTGCG

>Sequ13768SNP3
AAGTAGGAAGTTTTCTGTTTAATTCAGTTTTCATCGCACAGGCAGCGACGCGCGACCACAAAATGGACACAGAGACGC
CCACCAAGCTGGAGTTTGCAGTGCAGATGACATGCGAGAGCTGTGCAGAGAAAGTCAGTGCTGCTCTGGAGGGGAAAC
CAGGAGTGAAGTCTGTCAGCATTGATCTCAGTAGGGAAGAGGTGTTGGTGGAATCGGCTCTGACCAGTGCCGAAGTGC
AGGCTCTGATAGAAAGCACCGGACGCAGGGCGGTGCTGAAAGGCATCGGAGGATCGGTGCAAGATCTGGGTGCAGCGG
TGGCCATGCTGGATGGTGTCGGAAAGATCCAAGGGGTGGTGCGTTTCCTGCAGTTATCGGAGGAGCGCTGCTTGATAG
ATGGGGCCGTCGATGGGTTGGAGCCTGGACTCCACGGCCTCCACATTCACACCCTGGGGGACCTCACGCAGGACTGCA
GCAGTTGTGGAGAGCACTATAACCCGTTTGGAAGACAGCACGGTGGACCAGGGGACTCTGAAAGGCATGTAGGTGATC
TGGGAAATATTGTTGCCGGACCAGATGGCAGAGCCTCATTTAGACTAGAAGACAGTCAGCTAAAGGTATGGGATGTGA
TTGGCCGATCGCTGGTGGTGGATGCAGGGGAGGACGACTTGGGCCGAGGCAGTCACCCTCTCTCCAAACAGACCGGGA
ACTCGTGGGAAAGGCTGGCATGTGGGATCATCGCCCGATCTGCGGGCCTTTTCCAAAATCCCAAACAGATATGTGCGT
GTGACGGGGTGACGCTGTGGGAGGAGAGAGACAGGCCGATAGCTGGGAAAGGCCGAAACAAGACCAACGCAGCGACAC
CGGCAGCGCACCTGTGAACCTCGGGCACAAACACTGAGCCATCTCCAAGAGAAATGCAGAGGATGTTTGTCGACAGAG
GGTCGGCGAGGCGACGAGCACTGAGGAATGTGTTCACAGACAGCTGGAGGGAGAACCAAAGAAAAATGAATTCCTGAT
TTCCTTGAAATTTTTCTGTGATAAAATCAAACGCTCCTACAGTCGCAGTTAGTGTCCTCTGAGGGTTTTAAATGCACT
AAAGCAGATTAAAACAGACCAGGACTTTGTAAAAGTCCCCTCTGATTAGCTGTTATATATATTTGTATAACTAGTCGC
CTCGGACACAATCGTCTCATCACCATCTAAATTGATGTACATGTGAGATCTTAGGTATCCATTTTGACTGTGTGCTGG
TAGGGATAAATCCCCTTCAATTTGCTATAAAAAAAGGGTCTAAGCCTCCTAAAACAGTGGTAAGTAATAAAAAAAAAA
AATATAACACTGCTCTCACATCAGCTCACTCTCCCACCCAGGAACAATCATACACCAGGGAAACATTGTGGAGTGGCT
CAGCTGATTTAGAAAAAAAAAACGGTTAGTGACTTGAATTAAAATCTATCCACACGTTTCAGTTTAGGAGAGAATATA
ACAAATGTGTGATGCTGAAATCTGTCATCTTACACTTTTGGTCAGTTGTAGCTATAAGTGTTGTGACTGAAAAGACAT
ACAGGTTAGCTAGTTAACAGAGAAAAGTTGAAAGAAATAGTTATAACAGACAAATGATTGAAACATCTGCGACTGTCA
GAAGGATGAATACAAGCTTGACCAAACTCAGATGTGGACTCACCACAGCGCGTTATCTTGAATAATAAAGGGGAACAA
TGAGAGAGCACTTAATAAAAAAGAGGAAAAAAAA

>Sequ13860SNP3
CTTACGCTGCGCTCGCACGCGGCACGCTGTGGCGCGAAAACCCTTCAAGTTGTGCGGTGAGACGGCAGCGGGGAACAC
GGAGCCACAAACTCTGAGCAACATCTGAAGCGTTTTTCAAGAAGAATAAAAAAAAAATCATACAACATGGCAACCGAC
GTCGTCGACGACCAGGAGATGAGGGAGGCTCAGCGGGATTACCTGGACTTCCTGGACGACGATCAAGACCAGGGGGTT
TATCAGAGCAAAGTCCGCGACATGATCAGCGAGAATAAAGCCCGGCTCACCGTCAACATCAACGACCTGAGGAGACGC
AACGAGGCCCGGGCTGCAAAACTGATGAGCAATGCCTTTGAGGAGCTGCTTGCGTTCCAGCGGGCGTTGAAGGACATG
GTCGCCTCTGTGGACGCCACCTACGCCAAGCAGTACGAGGAGTTCTTCGTCGCCTTGGAGGGCAGCTTCGGCTCCAAG
CACGTCACCCCTCGTACCCTGACCTCCCGACTGCTGGGCAGCATGGTCTGCGTGGAGGGCATCATCACCAAGTGTTCT
CTGGTGCGTCCCAAAGTGGTGCGCAGCGTCCACTACTGCCCGGCCACCAAGAAGACGATGGAGAGGAAGTACACGGAC
ATGACCTCCCTGGACGCTTTCCCTTCCAGTGCCATCTACCCCACCAAGGATGAGGAGAACAACCCTCTGGAGACAGAG
TTCGGTCTGTCCGTCTACAAGGACCACCAGACCATCACGGTGCAGGAGATGCCGGAGAAGGCGCCCGCCGGGCAGCTC
CCCCGGTCTGTGGACATCATCCTGGACAACGACCTGGTGGACGTGGTCAAACCGGGAGACAGGGTCCAGGTCGTTGGG
ACGTACCGCTGTCTGCCCGGAAAGAAGGGAGGGTTCACCTCCGGCACGTTCAGGACCATCATGATCGCCTGCCACGTC
AAACAAATGAGCAAGGAGGTGTCCCCCTCCTTCTCTGCGGATGATGTGGCCAAAATCAGAAACTTCAGCCGCACCCGC
TCCAAGGATGTGTTCGAGCAGCTGGCTCGCTCCCTGGCACCCAGCATCCACGGACACGAGTACATCAAGAAGGCCATC
CTCTGCATGCTGCTGGGCGGCGTGGAGAAGGTGCTGGAGAACGGCTCGCGCATCAGAGGAGACATCAACATCCTGCTC
ATCGGTGATCCCTCAGTGGCCAAGTCCCAGCTGCTGCGTTATGTGCTACACACGGCGCCCAGAGCCATCCCCACCACG
GGACGAGGCTCCTCTGGTGTGGGTCTGACTGCCGCCGTCACCACTGACCAGGAGACCGGTGAGCGCCGGCTGGAGGCG
GGCGCCATGGTGCTGGCTGACCGCGGCGTGGTGTGCATCGACGAGTTCGACAAGATGTCCGACATGGACCGCACGGCC
ATCCACGAGGTGATGGAGCAGGGTCGTGTCACCATCGCCAAGGCTGGGATCCACGCCCGCCTCAACGCTCGCTGCTCC
GTGCTGGCAGCCGCCAACCCTGTCTACGGCAGATACGATCAGTATAAAACCCCCATGGAGAACATCGGCCTCCAGGAC
TCCCTGCTGTCGCGTTTCGACCTCCTCTTCATCGTGCTGGATCAGATGGACCCCGAGCAGGACCGGGAGATCTCCGAC

CACGTGCTGAGGATGCACCGCTACCGTGACCCCCGCGAGCAGGAGGGAACAGCCATGGCTCTGGGCGGGACAGTCGAC
GTCCTGGCTACTGAGGACCCAGACGCAGTAGCAGAGGAGCACGAGGAATTGCAGATCTACGAGAAACACAACAACCTG
CTGCACGGAAGCAAGAGGAAGAAGGATAAGATTGTGAGTAAGGAGTTCATGAGGAAGTACATCCACATCGCCAAGATT
GTGACACCTGTGCTGACGGAGGAGGCGGCCAATCACATCGCAGAGGAGTACTCGAGGCTGAGGAGCCAGGAGCAGCTG
GCTGCAGATATCGCCAGGACCTCTCCAGTGACGGCCCGAACTCTGGAGACCATGATCCGTCTGTCCACGGCACACGCC
AAGGCCCGCATGAGCAAAACCGTGGAGCTGGAGGACTCGGAGGTGGCCGTGGAGCTCGTCCAGTTCGCCTACTTCAAA
AAGGTTCTGGAGAAGGAGAAGAAACGTTCGAGACAGGAGCGGGACTCTGGCTCGGAGGAGGAGGAGGAGGAGGATTCG
TCAACTCAGCGTTCACAGAAAACTCAGAGGAAGAGGGGGCGCCGTGGCTCTCAGGGCAGCGAGCCCTACAGCCCATAT
GACTTCAGTGAGGAGCAGAACGTCCCCGAGATTCAGGCCGGCACGCCGAAACCAGCCAAGCCGCAGCGAGAGGAGGAG
GAGCCCATGGACGCCGCCTCACAGGCTGAAGACACTGAGCTCTCTGCAGACAGACTGAAAGAATCCAAGTCGTCCCTG
TTCGCCGTGTTCCAGTCCGCTCACGCTCAGTCCGTGAAGATGAAGACGCTGATGGACAACGTCAACAAGGAGCGCCAG
AATCAGTTCACGGAGCCAGAGGTTCGCGCCGCTCTGGCCCGCATGCAGGACGACAACCAGGTCATGGTCGCTGATGAC
ATCATCTTCCTCATCTGAGGAATGACGAGGAGGATGACTGTTTCTGAAATTTAATAACATGGACTTTGAAAGAAACAG
ATTTATATCAAAACTGTACGTTTATTTCATCTGATATTGGACACTTCTTTTATTTTTCACGCTGTTTTTCGTCTCTCA
TTTTCTGGAAAGACACCTGACCTCTGATGCGCATGAGGCACCAAGAAAGACTTGAACCGGTTTATTCCTGTGGTGGGT
TTTGATCCACATAAGGTGCAGGTTGATCTATTAGCAAGACCAGTTTTATTCATATATGTATGCACTTGTTTCCTTTCA
GGTAATATCACTTCAAATGGTTTTATTACCTCGTCTCAACAGATTAATGTTGTGTGTAATAATGAGCATTTCAGTTCA
TATAAAGGAGGCAGGTGTTGCAGCCCAGTGCAGGTACAGTCTTCATTTAGCAATGAAACATACTGTGCATTACTGCAT
CAGAATCAGGACATTGTGAAGTTAGTGTTACCAACACTTCTGTTGTCTGGGTTTGTATATAGTTGAAGAGGAGTTTTG
AAATTGTTAATTAACTGTAGTTGTGTATAAAAA

>Sequ13900SNP2
AACCGATGTGCAATAACAGTTCTGTGACGAGTTTAGTTTGACTAAGCAAGAAGTGGTCGGTTAGCTAATATCGTTTAC
TGCGGCGGCCTAGCTAACATTAGCTAGCGCTGTGAAGTTGGGACAGTCCTTTCATTTCATGAGCTCACTGTGAGGGCC
TCTTTTAACCTGCTGTGCTTTCCATGATACTACAGCTCCACCTGGAATTATTTCATTATCACTGTGGCTATATTGATC
AAAGCTTATCGTACCTTTAGTGGAAGCCAGATCCTCGGAAAGCCAGAGTGAGTACGGCTTCTTGAGAACAAATCAAGG
GGAAGTGGTTATCCATGTCCACGAGCTACTGCGGATTAATAATCTTTAATAACATCAGCAAACATGTCTTTAGTCGCC
TATGGCAGCAGTGATGATAGTGACTCCGAGGAAACCTCCACTTCTGCCGCAGCGGAGAGCAAAGTCAGCGCAGGAGGG
CTCTTCTCTCTCCTGCCTGCTCCTAAAAAGCCAGGATCTGCAGGTGGAAACGATGGACCAAGAAAGGAGACAAAAGCA
AACCCTTCAGCTGGGGACAGCACGTCAAACGATGACCTACATCCTCCAGCATCTAAAGGAGGCTTCCTTTCTAGTCTG
CCTAAGCCGAGGAAGCGAACAGAGCCTGTCAAGATCACTGTGCCAGAGATCCAGAGACACGATTCAGACTCTGATGAT
GATGAACCAG

>Sequ14234SNP3
GGCAGTCCGGTCTCATTCACACCACACGGCCGTCCTCTCCTGCAGGAGTTATATCATCCCGTGTTGCACACCTTGTAG
CAGCTACAAGCCTTTTACACCACAAAGATGTTTCGTCTCCCAACAGTCATGAAGCAGGTGAGGCCTGTGTGCCGGGCG
CTGGCTCCTCACCTGACACGAGCCTACGCTAAAGACGTGAAGTTTGGAGCCGACGCTCGCGCCCTCATGCTGCAGGGA
GTCGACCTGCTGGCGGACGCTGTGGCCGTCACCATGGGCCCAAAGGGTCGTACCGTCATCATTGAGCAGAGCTGGGGC
AGCCCTAAAGTCACCAAGGACGGCGTGACCGTGGCCAAGAGCATCGACCTGAAGGACAAGTACAAGAACATCGGCGCC
AAGCTGGTGCAGGACGTGGCCAACAACACCAACGAGGAGGCCGGCGACGGCACCACCACCGCCACGGTGCTTGCTCGT
GCCATCGCCAAGGAGGGCTTCGACACCATCAGCAAAGGCGCCAACCCCGTGGAGATCCGCCGTGGAGTCATGATGGCC
GTGGAATACGTCATCAGCGAGCTGAAGAAGCTCTCCAAGCCCGTCACAACCCCCGAGGAGATCGCACAGGTTGCTACA
ATCTCAGCCAATGGAGACGTGGAGATCGGTAACATCATCTCTAACGCCATGAAGAAAGTTGGCCGCAAGGGAGTCATC
ACTGTCAAGGACGGGAAGACGCTGCACGACGAGCTGGAGATCATCGAGGGCATGAAGTTTGACCGTGGTTACATCTCC
CCGTACTTCATCAACACCGCCAAAGGTCAGAAGTGTGAGTTCCAGGACGCCTACCTGCTGCTGAGTGAGAAGAAGATC
TCCAGTGTCCAGAGCATCGTTCCAGCCCTGGAGATCGCCAACCAGCACCGCAAGCCGCTGGTCATCGTGGCGGAGGAC
GTGGACGGAGAGGCCCTGAGCACCTTGGTTCTCAACAGGCTGAAGGTGGGGCTTCAGGTGGTCGCTGTCAAGGCCCCG
GGCTTCGGAGACAACAGGAAGAACCAGCTGAAGGATATGGCCATCGCCACCGGGGGCACCGTGTTTGGAGACGAGGCT
GTGGGCGTGGCCCTGGAAGACATCCAGGCTCACGACTTCGGTAAGGTCGGCGAGGTGCAGATCACCAAAGACGACACC
CTGCTGCTGAGGGGAGGCGGCAGCCCAGCCGAGGTGGACAAACGCGCAGCGGAGATCGTGGAGCAGCTGGAGAACACC
ACCAGCGACTACGAGAAGGAGAAGCTCAACGAGAGGCTCGCCAAGCTGTCGGACGGCGTGGCCGTGCTCAAGATCGGA
GGAACGAGTGACGTGGAGGTGAACGAGAAGAAGGACCGTGTGACGGACGCTCTGAACGCCACCCGGGCAGCTGTGGAG
GAGGGGATCGTTCCAGGTGGAGGCTGCGCTCTGCTGCGCTGCATCCCATCGCTCGACAGCCTCAAAACCGCCAACGCC
GACCAGAAGATCGGTGTGGACATCATCAGGCGAGCGCTGCGTATCCCCTCGATGACCATCGCTAAGAACGCCGGTGTG
GAGGGCTCACTGGTGGTGGAGAAGATCCTGCAGGAGTCAGCTGATATCGGCTACGACGCCATGCAGGGGGAGTACGTC
AACATGGTGGAGAAGGGCATCATTGACCCCACCAAGGTGGTGAGGACGGCGCTGCTGGACGCCGCAGGAGTCGCCTCC
CTGCTCTCCACCGCCGAGGCCGTCGTCACGGAGATCCCCAAGGAGGAGAAGGAGATGCCGGGAGGTGGCATGGGCGGC
ATGGGAGGCATGGGCGGCATGGGAGGAGGCATGGGTTTCTAAGCCGGTCCTCCTGACTTGTTGCTGTACAGACTAAAA
GGCAGAGCGGTGCTGGGGGTGGCTGTGGAGCACACGCAGAAGAAGAAAAAAACAAACAAACAAAACCCTCCAAGAATC
TGCAACCACCTCCAGCGAGCTCTGCCCACACTGACTGGCCATGAGTATGTTGGAGATGCCCACGCTGTTTAATTCTGT
CTCCTGCCTCCGGAGGACCCGGTCCGGAGCAGGAGACCCCATCGAGCCCAGTTACTTACTACACCAGAACCCTCCGAC
GGCTCTGCTCATCGAACCAATCCACATGTTCAAACTCATAGTTCTTCATGTAAAACGCTCGTACAGGTGATGCCACTG
TGATCTCTGTAAGAGGTAGGAAAGTAATTCAAGTCGTCTAAGAAGAAACTTCAGAAACTAACATAGTCATGCGTATTG
ATGCTCTGTATTACTGCAAAAGAGAGCAGGGCAGGTACGAGAGAAGAAGCCTAGCCAGGAATGTGTCTGTCCTTTATT
TTTGTCCTTGTAGTATTTTGTTAATTCGTTTTGTTTTTTTATATATATATATATCTGTTATGGTTTTTTTCCCCCCCA
GCTGATAACGACGTGGGTTAAAATCTCTCATTTGATTTTAATTAAACCAGTTTTCTTTCAGAGCCTGAGCATTGTCTG
TGTCCCTGAAGTGACGTTACATTTTGTTTGTCTGGTGATGATGGAGGAAAGATCACCTGTTCCCTCGTACCTG

>Sequ14380SNP3
ATCACAGTGGCTAAGATAGGCAGTTGCTCCTGATGGCATGGAAACAAGGGAAGAAAATCAAGGTCAAAATTTCTACAC

GTGACTAAGTAACTATTGGATATCGAAGTTACATAATCACAAGTGAACAAAAGATGCCATGGAGCAGCTAGAAGAGGA
ACTTACCTGCCCAATCTGCTGCGGTCTCTTCGAGGACCCACGGGTCTTGCTGTGCTCACACAGCTTTTGCAAGAAATG
CTTGGAGGGACTCTTGGAAGGAAACCGAGGTCCAGCTTTCAGAACACCTTTCAAATGCCCCACATGCCGCAAAGAGAC
CCCTCACAACGGCGCAAACAGCCTGCAGATCAACTACTCTCTGCGCGGAATAGTGGAGAAGTACAGCAAAATAAGGGT
TCTGCCTAAGATGTCTGCTTGTAAACAACACTGCGACCAGCCTCTTAACATATTTTGCGCCACGGACTTGAAACTAAT
TTGTGGGTTTTGCGCAACTACAGATGACCACAAAGGGCATAAATTCTGCTCCTTGGAGGAGGCATATGACCATGAGAA
GGAGGCGTTTGAAGAGCTGCTTCACGGGGTGGAGAGCTGGCAGAGCGCAGATATCCTGTCCTGCCTGGAGACACTACA
AACCAGTAAGAAAAAGGCTCTTCAGTCCGTGACCAAGGACGCAGAAAAGGTAACAGACTATTTTGACAAGCTGATCAG
TGCACTTGAATGCAAAAAGAATGAGATCCTCTCCGATTTTGAAACACTGAAGCTGGTGGTGATGCAAGCATATGACCC
GGAGATCACCAAGCTGAGCGCAGCACTGGAAGAGCAGAGACTGGCGCTCAGCATCGCGGAGTCTTTCAGGAGCGTCTC
CGACCCCTTGTGCTTTCTGCAGCAGATGCAGGAGTTTCGGGAGAGATTACAGGTCCTTAAGGAGACTCCCCTGCCCTC
CCGGAAAGAGATGGACGTCGGCCCCCTTGTGCGCAATTTCGATGTAAAAAAGTGGGATTCACTAAGGCTCAGAGACGT
GGACAAGATCTCAGTCCCCCATGAGAATGGCTCGTACAGAGTGGGGGGCTCACGGATGGCAGCGCCTCGGTGGATCAC
CTTATTTATGAGTTTGTTGCTGTCAACTCTGCTCCTGCTGCAGCCGCACAACCTTACAGCCTACGTAACCTCTCAGAT
TGAACCATTTCTTACTACAGTTTCCTCGCACCTCAGCCACACTGCTGTGTACCTGCAGGAAATGACTGACATGTGCAC
AAGTTTAATGAGCGCAGGCCAGGAATGTGTCGTGCACTTAATTGACTCTACTGTCAGTTTTATTGGCAGTTGTAAGTT
GTTTTAATTACTAGCCTGTGGCATCTCACGCACTCACAGCTACAAATTGTTTCCCAAGTGACCCGTGTGATGCACAAA
AGTAGTGACAAATATGCACCTGAGCATATGTAAACATTTGTTTATCTAATTGTTTATTATGTCTTTTGCTCTTTTTTT
GCTCAACCTCTCTGAAGCCACAGAATCGATCATTCATTCAGAGATTCAAGCCTTCATTTACATAGGACAGACACTAAA
TCACACCTCTTTCCACAAATGTGCGATCTATTCTTAATACGATGACACTTTTATACTTCTCCAAGATGATCTTGATAA
ATAAAACTGAGAAATGTAAGACACATCCATGTTTGA

>Sequ14504SNP3
GGAGCCTCTTAAAGAAGAAGTCACAGAAGCTGGAGGAAAGGGTGCCAAGGCAGGTGGAGGAGGAGGAGATGACGATGA
GCTGGCACCCTCCAGATTGGACATCAGAGTGGGCAAGGTCATCAGTGTGGAGAAGCATCCAGACGCTGATTCACTGTA
CCTGGAGAAGATCGACGTGGGCGAGGCGGAGCCCAGGACGGTAGTCAGCGGGCTGGTGGCCTATGTTTCACAGGAGGA
CCTGCAGGACAGAATGGTGTTGTTGTTGTGCAATCTCAAACCCCAGAAGATGCGAGGGATCGAGTCGCAAGCCATGCT
GCTGTGCGCCTCTGTTGAAGGGGAGCCCAGGAGGGTGGAGCCTCTAGACCCTCCAGAGGGTTCGTCACCAGGGGAACG
GGTCTTTGTGGAAGGATATGAGACAGGCAAAGCAGACGACAGACTCAACCCAAAGAAGAAGGTGTGGGAGAAACTACA
GGTCGACCTGAAGGTCTCAGACGAGTGTGTAGCTCAGTGGAAAGACAAGCAGCTGATGACCAAACTGGGACAGATCAC
ATGTAAAACACTGAAAGGAGGCAACATCAGTTAAATACACTCAAGCTACATTCACCACTGAAGCTGCTGCTGCCCACC
ACCTCAAAACAACAATACGCTCATCACCAAATTGCCATGGCGACAACGGTCAATCTTCATCTGGATGTTTTCTCAGAA
AAGGACATCGATGCCAAAAGTCTCAGATGCTTTCAGACTGGCTTTGACAAATTCTGTTACCTTGCTTTGACTTCATTA
GGAAAGATGTATGTAATTCCTCTAAACATAAGCAACTTACAAGAAAAATTATGAAACTAATCAACCTTTGATTCATTC
GTTTATCTTTTTGTTTACTGGGCATTGATGGTTTTACTAATGCACTTAAATGTACACATACAACAAATGTTCTGCAAA
TCGGAGATAGCGGGGCCTGATCAGATAAAGAAGGACTGATTGCTTGAATGTTGACGTACCAGTCGCTCATTCCTGGTA
GTAGCTGATAAGGATTCTCAGGTGTTTAAAAAGGGACTTAATGTATCAGTACTTTTCCTGGGTAAAATTGCACTTGTG
TTGTTTGCCTTCATGGTAAATGTCATGCCAACCTGCCAAACCTAATTAATAAGAATGGTTTTATGGGGGTTTCAGTCA
TCAGATGTGTGGAAAGATGTATTTGCCTTGTGCTATAGGACGGTTGCATTTGGGGGTT

>Sequ14769SNP2
TACAGGTGTTGGTCAGAAAAACGCTAAAGCAAGTTTTAGATAATGCGTGATTGCGTGCAGAGCCAACGGGAAGATGCA
CGACTGTCCAAGGCTTTACAGCTCTACTTCATGAACGTAGAGAGAGAGACTGAAGGAAACTGATGTTCTGATATTTAA
AACTGACAACAAACTCAAGCAAACAAACTTCCACAGCAACTTTGCTTCGTATGTTGCGAGCTAGCTCAAGTTACAACT
GAGGTCTGTACAGTTGGCACATTCGGCTGTTTGTTTCTGTCTGACAACTCCGTATCATCATGATTTATCACAGTGAAA
TCAGTTCATTTCAGAGGAATCACCACAGTCGTTGAACTCACGCATACAAGAGATACAGATACCAGTGTGTTCTCTTGA
ATTAACCGTGGTTTCTTATTGTCCCGGGGGTAGCAACCAAGCCGGTTAGCTTTTTGAACGCTAGCAAGCAAGCTATTT
TGGTTGGAGCCTGTTACCAGATAGCTAAACTAGCCGCAACAAAAAGATAGCTACGGCGCGACTCCACGGCTAGCCGCT
ACATCGCTAAGCTTCCAGTAGCACCTTTTTTCGCAAGGACTATGCGGTGTAGCCAGGCGCTAACACCATTGTCACACT
TCGTCTTCTTTTCTCTTTTGACTTCCAGTCCATCGATTCAGTTGATTCTTTTATATCAAAATGTAATATATAATATCA
GTGTAAAGAGAATCAGAAGAAGATGTTAGCATACGTGTACTAAAGACCTCAGAGTTGGTATCGATGGCTGAGACGAGC
TTCACAGAAACACTGGGAGTGTGATCGGACAGATTTCTATCGACGTTTTCTCCCGCCTCTGTGCCATTACACTGGAAT
ACTTATGAATTTGACTGTCTTAGATTAACCTATCATTACGTGTGACGTATAATTGTTATAAATAAACGTTAAAGAAG

>Sequ14956EST2
TCAGGAGGTGACACTTCCTGGGGTTGGGGGCAGGTGGTCGTACAGCGGGTCTGACTGGCGTTTTGGGGGGTGGACCTC
GTGCCGGCTCCTGAGTCGACGGCTGTGATCTGAGAACCACTAGGGACGCTTGTATAGTGGGCGAGGAATCCATCCGAT
GTCACACTGAGGTCAGACACGAACTGGACCAGCAGCACATTGCCACTGGTAATAATGGGTTGCGGGGCCTGATCGCCA
CAGTATTTTCCAATCAGCCGTGAATCATCTTTCTCTCCACCGTTAAAGAACGCCACGTAGTCAAAAGCGACAGTAGGT
GTCAGACTCCAAGAGGAACTTATCAAACTTCACCCTGGATTACCATTTCAGGCTCCACCGTGATAAGCCAGGAGCAGC
TGGTTCCTGGTGGGTATTTCTTGTCAGGCCAGTTGGGCGTCATGATCTCTCCCTGGGCTTTGGTTATCTTCCCCCCGC
AGAACTGCTGGTCATCCACATATGGTTTGGCTCCACTGAAATAGGCCACAAATCCTCTGCCCTGCGTCTCTGCATCAG
ACGCCATCTCTAACATCATGGTGTTCGTGGTAGAAATAAGAGCGCCGGGCCGGAAAGTTCCACAAAATCGACCCAGTT
TTTGCACCAAGTTGGAATGGCCATTGTAAACATCCAGATAGTCGTAGCGACACTGTGAGTCAGCCTCCAGGTCAAAAA
TGCGGAAGGAGAGCATGACCACGTTTCCCTCAGGGACAGTGATACGCCAGGTGCATTTGCTGTTAGGTTTGTAGAAGC
TTGGGAACCCTTNACTGCCAACAAACCCTGAGTCCGCCACCAGGTCGCCTCCGCAGTAAAACACAGGCCTGGTGTAAT
TCGTCTGCTGAGCCTTCGTCCATCCTAAACTCAGAGACAGGATCAAGGACAGACCCCAGATGCAGCCCACACACACCA
TGATGCCGCGTGCACAC

>Sequ15243SNP3

TCTTCCCCCCGAGTCCCCTCCTCGGAAGTAGACGCATCTGTCTATATTCCGTTCTTTTATTTTAATACATTTTTTACA
GCAACCCCTTTTCCCCAGCACATAGCACCATGGAAGCCAAGATGGACGACTAAATTAAACAACGGGTACCCGATTTCA
CCCCCTAATTATCCTTGAATTGAGTGGACCGGATGAAATCCAACTGTGGTTTTCTCCGCTTCGCAGGGGAGGTAGCTG
TATCCAGTGGGGAGGTAGGCTCAACCAGGGGAGGAAAGAATGAAGTTCACTCAACCCTGGGGACGCCAGAGGCTGTCT
TTTCTTCTGGAAAAGGCTGCCTCTAGGGAAGCCAAGATGTGGAAGGTCTACGTGCCAAAGAAGCCCTCCTCACAGGAT
ACAGACATCTCCCCGGCCCAGCGGGACGAGGCCGTGCGCTGGTTGACGGAGCTCCACAGCAGGCTGCAGCTGTACCCA
GAGACCCTGGTGTTAGCTGTTAGCATTCTGGACCGCTTCCTCGCTCCCATCAAGGCCCGTCCAAAGTACCTGCGCTGC
ATCGCCATCGCCTGCTTCTTCCTGGCTGCCAAGACATGTGAGGAGGACGAGTGTGTGCCCTCTCTGAGGGAGCTGGCT
GCCTCCAGCAGCTGTGGCTGTTCTCCATCAGAGATCCTGAGGATGGAGAGGATCATCCTGGACAAATTGAACTGGGAC
CTGCACACCGCCACAGCACTGGACTTCCTGCACATTTTCCATGCGATGGTGTTGTCGTGTCGTTCTGGGTTTTTGGAC
TCCATGTTGGGATTGAACCGCTCTCAGCACCTCGCCCTGCTCACACAGCGACTGTACCACTGTCTGGCTGACCACACT
CTCATACAGCTCAGAGGATCCATGCTGGCCTTGGCCCTCATCACCCTGGAACTGGAGACCTGCTGTCCTGACTGGCTG
GCTCTCACTATTGACCTGCTGAAGAGGGCACAGATCAACAGTTCTGAGTTGATCAGGAGTCGAGAGCTGGTGGCTCGT
AGACTGTCCACACTGAGAGCTTCCCTGCCTCCAAACACTGTCTACATCTACCAACCCCTGCAGAGCCAAACCGCTCTG
CGGCACCCGGCCCAGAGGACCCCCCACCCCCCTGCTGCACACTGGGGACCGTCACCTCCACCACCGAGTCTTCGAGGA
ACCACGCCCTCGTCAACACCTCCCCTGCCCAGCTACAGACACCTGCTGGAGGGGAGGAGGGGAGTCCGCAGCCTTGGA
GCTCCATCTCCTCCTCCACCGACAGCAGCATGCCGACTCTGCTCTCTCCACCCAAAAACCTCTACCATCGCAACCGCC
TGCAGAAGGTCACGCTGCGCTGCAAAGCCTCCGCCAAGCGGAAGGTGGAGGAGATGGAGGTGGACGACTTCTTCGACG
GCATCAAACGCCTGTACAACGAAGACGTCACCACCACCATCACCGTCCAGGAGGGGGCGACACCTGCGACGGGGGCGA
TAACATCAGGTGGAGGAGGAGGAGGAGGTCTGAACACCTGCAGTGTCCTGTTGTCTCGACAGGAAGGCAGTTCTTCCC
CCTGCCCGCCTCTGCAGCCAGTCAGTGCCTCCTAGTGACGGAAGCCGTTTACCAAAACCACCTCAACACCGACATCAT
CTTCCTGTTTCACTCTCAGACTAAAATAAAGTATTTAAACTACGGTCAGTTAAATTTAGGCAGCAACCGTGATGCGTG
ACTCCCTCAGCAATAGTCAGTGTAGGGGGAGAAAGAGAGAGATTTTGTTTAATTTGCATTAAAAAGCAATACAGAGGC
AGCTCCTGGGGGACGGTGCTCATCAGAGTTGTCAGTGAAAATCGTCCTTCATGTTGCTTCTGCTGATGAAACATTCAC
TGAAGAGTTAAATATTTGTTACAACACAAACAAAGCAGTGAAACAGATGATGTCAGAGGTTAAGGTGGTTTTGGTGCA
AATGACCCCAGCGTGACCCGGAGCTGTTCAGCTGCCCCGAGGTCAGTTCCCATCATCCCCTGCTGCAGGACAACAGAG
CCCAAAAAATTTAACATTCAAAAAATAAAAACTGCTGCTACTATCTGATTAATGATATTTGTTGAAAATGAATACAGT
ATTACAAAAGGTTTAAAAAATAAATAAAGAGAAAAAGTTATCTTGAAAAAAACCTAGAATTGACTTCTCTCCCCGTGT
CTCGCTCTCAGTCCTCTCGTGAAAGATTTTGTTTGTCAACTTATATTTGTTGTTCACTTTTTAAACTCTTCTTTCATT
TCACATATTTGTGTTTTTATTGTCTTCTTCCCTTCCCTTACTCACTCTGCATGGTTTCATCTCTATTTATAACTTCCT
CTTAAAGAGTCTAAATCATACGTATGAATGTTACTGTATAGACGATTATCTGTCGTACTCGCCCAGATAGAAAAAAGT
TTAGGTTTTTTTTGTTTGTTTGATCGTTTTTGTTTTTGTTTTTTAGTGAAGTGCAATTTGATTGTTAATGAGATGCTG
ATGTAAACATCTTACCTATGAATCACTTTGTGTTGTTTCCCTTCCCTCTCAGCTTGTTGTGAAAGCTTGACTGAATGA
AAGTTTCTCAGGTTGTTTTATTCACAAGTTCCTCCTGCAGATGTCACAGCTGCCAAATGCATTCAGTTTGTTATGTAG
CCCAACTCATCTTGAGATAAATTATTAGTTACTACAGCAGCTCTCAGTGGCTGGAGTCGACCACAGTCTACCCATCTG
GTTCTTGTGCAAGTTTAAAACAAATGCTGTGAAAAACTCTGGAGGACCTGCGGTTCTTTAAACCTGTTTTAACTAATT
CAGGTTATAATGATCCTGCTTCTTCTAATTATTCTTTTTAACACAGAGACACCAAGAGATCTAGATAAATTCTCATTT
AAAACCATGAAAGTCGTGGTTATGAGGACGTGTTGCTATGACAACAGCTCCAGTTATATTCAGAAAAATCCAGGCTTT
ACAGTCAACCTAACTGAAGAGGGCCCTGGTAAAAAAAATAAGATGGCATGTGGTTTCTGTGTTGAAGCAACAAGAGGA
AAACTTTCATATGTAATAAAACAACCTTGAGTTATTGAAAGCATTTTAATAGTAAGCTAAGAGACAAACATTAGGTTT
TTGAGTTGGTGTCTGAGCTGAGCTGACTCAGAAAAGGAACACGAGTGACAATCTTCACTTTGATGTTAAAAAAAAAAA
AAAACGCTAAAGCAACATGCTCTGTTTTTAATCTCTTGAAGTTCTGTTTAATATATTTTTATTCCTGCAAAAAGCTCT
GTTCTTCCTGCTGTAGTGGCTGCCGGTGGCTGCTCCGGGTCCCTGAACACACCGCGGCTCCAATAAAGATATAAGAGT
ATGAAAACTGACCATGAGCTCTTTGTCTTCATTTTCTTCTCATTCAGCTAGGTTAGGATTCATTCGTCATTAGAAGGA
AATGAGACGAGGAGCAGATAAGAGAAAGAAGGAAGGGCAAAAAGGATGTGGAGAATCAAACCCACTCATGATATAACG
TTCACCAGCTAAAGAGCCAGATGTTCTCAGGTACTGGAGACCAGACCGGTGCTAACAGGAGAGTCAGTACAGGGATGA

>Sequ15285SNP3
GAAAAAACTAATCTGCACTAAGACAGAGCTGAGCTGAAAATCACTGTTTTAACTCAACAACATAAATAACTTTTCCTA
CTCTTTCTACTTCTGGTCAGTCTGTTTTTATTTGAATAAAAACAACGTGGCCTCAACACGTCAAATTATTGAGTGATT
CTCCGAGCTCCCAGTCTCAGTGTTAAGCTAACCACAAACACAGGGCTGTAGATTCATCTGAGGCCTTGTCCTTAATAT
TTTCTTCTGAGGATCTGATGGTTTTAGGGATCTTGTGTAATTTTTATATTCTGTGATTTAAAACATTCCACTGGTTTA
GTTACATTTGTCACATTTGTGACTTTTAGGGCAATAGAAGTAAATAATTACATAAAGGATTGATATAAAATATAAAAC
ATACAAAAGAATAATGCTATTTTTATAGGTCATTTATCTGAGATTTCCTTGGAAGTTTCCTGGAAGTGACTGTAATCA
GGCACAAGTTATAAAAAGGACTGGGACAGTTGTGAGACATTTAAACAGATTTTATGTGGGTGTGTGGAGCAACTTGTA
AAGTTGAAGAAAATGTGTTAATTCCTGAAACTTACATGGACCTATCACTGTACTGGACCTCTGCCTTCTCATGTGACT
CTGGGAATCACAAAGAACAAAAGGACTTAAAAGGTAAAGGTTGGACTGTAACTCTCAGTGTGTATCAGGTTTAATAGA
AACAACAGAGGCCCCAGGATACTTCCCTGAGGGACCCCATGTGTTTATTAAAGACAGGGAACAGGATTCCTCCATCTT
TACCAGCAGACTTGTCTCTGTATGTCGCTGAACAGAACATTCAGCAGTGAATCTCTTGGGTTTGAAGTAGCCTTTGTG
TCTCATTATAACTGATTGCACTAATCTGTTGATGCTGAAAATTATGATCTTCATTTATTGATTAGTTTTGGTTGAAGT
TGTGTAGAGACGATGCAGAAGACATGTGTTGTCCCTGTTTCTTTTTGTTCCTGTGAGATTAATAAGTTTGATTTTTGC
TGTGACTACTTTTGATTTTCTTTTGTGTGGAATGATGGCTGGTACTTAAAGCCTTTCTGACTTTGGGATCACACATTT
CATTTGTAATGTTTTCCTTTTGATCATGATAACTGAGCTGTGGTACATTGATTAATTCATTACAATGCACTTTGATTT
ACAGTAGGGGTGAATAACGTCGTAATTGCAGCACTGTGAGAGTTTTATATGTTCAAACTTGTCTAAGACAGAAACACA
CCATCCAGGTAGCTGTTAGCATCGTTAGCTATCCCCATTGAGAACACAAGTGGAAGCTAACGGACAAGCATCCAGCTG
TGTTCCCAAGTGTTTAGGCTTACTAGCAGCTTACTTGCAGATCTTACACATTAGATTTTAGATGCCTGAGTTGTGATT
TCCACCTCCAAACATCTCACATTATGTCATTTGAATAAATGTGTGAGGATCAAAGAGGTGAAACAATCCAATGTTTCA
CTAAAAGAGATTCAAGTTTGTGCAGCGGGTTTTAGTTTTTCCTTTGATCCATCAGATTCAACTTGTGACCCACCAAAG
GGGTCCTGACCTTTTAATCAGAAACACTAGCCCTGTTTCGCTGAAAGCTGCTGTAACTCCTCAGTTTCACCACAGTTC
AGACACTTTGTGAGGGAT

>Sequ15340SNP3
ACAAAGTGTGGTGACCCAGATTGAGTGCGTGCTAATGCGCATGCGCTGATGTGACTGCAATTTCCTTTTCCCTGAATC
TGGCGGAACTAGTGTCCGGTGGTATTAGTTTTTATTTCACGGACTGTTTTTGCTCATAAAGAGGATTTCGAGAGTTTT
TTATGAGTAATCAAAGGCAGCAGAAGCCTACGCTAACAGGCCAGCGTTTTAAAACTCGCAAAAGAGATGAAAAGGAGA
GGTTCGACCCTTCCCAGTTTCAGGAAAGTATCATACAAGGTCTGAACCAAACTGGCACTGATTTGGAAGCTGTTGCAA
AGTTCCTTGATGCCTCTGGTGCCAAACTTGACTACCGACGCTATGCTGAGACTCTGTTCGACATCCTGGTGGCTGGTG
GAATGCTGGCCCCAGGGGGTACCTTGTCGGACGACGTGACATGCACAGACTTCTGTCTCTTCAAAGCACAAGAGGACA
TGGAGACCATGCAGGCATATGCACAGGTCTTTAACAAGCTCATCAGGCGTTACAAATACTTGGAGAAAGGATTTGAGG
AGGAGATTAAAAAGCTGCTGCTGTTTCTCAAGGGCTTCACAGAGTCTGAGCGCAACAAGCTGGCCATGCTAACAGGAA
TTCTGCTTGCCAACGGCAATATCTCTGCATCCATTCTTAGCAGCCTCTTCAATGAGAACCTGGTCAAAGAAGGTGTTT
CTGCATGCTTTGCTGTAAAACTCTTCAAATCCTGGCTTTCTGAAAAGGACATCAACTCTGTTGCTGCCAGTCTCCGAA
AGGTTGGCATGGACAACAGGCTCACGGAGCTGTTCCCTGCAAACAAACGCAGTTGTGAGCACTTCTCCAAGTACTTCA
CAGACGCTGGGCTCAAGGAGCTTTCGGACTTTGCAAGAAACCAGCAGTCCATAGGTGCTCGCAAGGAGCTGCAGAAAG
AGCTTCAGGAGCAGATGTCGCGTGGGGACCCCCTCAGAGATATCATCACCTACCTCCGAGAGGAAATCAAGAAGAACA
ACATCTCCGAGCAGACGATGATTGGATTAATTTGGTCCAGTGTAATGAGCTCCGTGGAGTGGAACAAGAAGGAGGAGC
TGGTCACAGAACAAGCCATCAAACATTTAAAGCAATACAGCCCACTGTTGAAGGCCTTTACCTCCCAGGGCCTCTCTG
AACTTACTCTCCTGCTGAAGATCCAGGAGTACTGTTATGACAACATCCATTTCATGAAGGCCTTCCAGAAAATAGTTG
TGCTGCTCTACAAAGCGGATGTCTTGAGTGAGGAGGCGATTCTGAAATGGTACAATGAAGCCCACCTAGCCAAAGGAA
AGAGCGTTTTCCTTGAACAGATGAAAAAGTTTGTTGAATGGCTCAAGAATGCAGAGGAAGAGTCCGAATCTGAGGAAG
AGGACGCAGACTGAGCTCTACTTGCTATAACTACCATCTTGAGTTGTATTTTTACATAAACGCAGCACCAGATGCAGT
AGAGGGATTTGTTACTGAAGTCTGTCTATATTTCTTTTTTCCTTATCTCTTCTCTACCTCCTCACATCTAGTTCAACT
GTAATGGAAGGGCTTTAATACTGTTTTTATTTTCTACATGTTTTGGGCCTGAATCAATACTTGTACTCTCCTTTATAC
TCTCCAGGAGCCGGTCACAGTGCCACAGTTAGCTAAATAGATTGAAACCCCTTTCATGTAACAAAGGGAGGGAGGGGT
GGGGTTTTGGAAAGATAGTCCTTTAAAATTAGCCAGTTTGAAGGCATATTTCACAGTTATTGTATTTAGTCATGGAAC
TCTTACTTGCCAGAGGTCTATCTGTGTAGCCATGATATCTTGGTGCTCTGAGAGGCAAACTGTTTTTGTAATCTCATT
TTATGTCTCTGCCAATTATGTGACAACTCTGAATTTATTGCCAGTTTTTAGATACTGTCTCCATTATTGGTGATGGAG
CTGTGTTGTTTCAAAGGTGATGGTGGCAATGCCCCATAAAATTGAATTTAATGCTTCACACATCTTAAGTAACAGAAC
CATGCTAGCTT

>Sequ15646EST2
AGTCTTTGCGGTCTACGAGTCGGGGGGTGTTGACTCGACCTTGGAGTTTGACGTGCAGCAGCATGGCGAACAAGGAGC
CCAATCGCTTCGTCCAGCACCTGAGGGATCTGGCGGGCCGGATGTCTTCGTCTGGTGGCAGAGGGGCAGGATTAGGAC
TGAAGCTGCTGTTGGGAGCTGGTGCTTTGGCATATGGTGTTAAAGAAGCCACCTACACAGTGGAAGGTGGTCACAGAG
CCATCATCTTCAACAGGATTGGAGGGATGCAGATGGACACTGTTCTGGCTGAGGGGCTGCATTTCAGGATCCCCTGGT
TCCAGTATCCCATCATCTATGATATCAGAGCCAAACCCAGGAAGATCTCCTCTCTGACTGGCAGCAAAGATCTGCAGA
TGGTGAACATAGCGGTGCGAGTGTTGTCCAGACCCATGGCGTCCAACCTCCCGGTCATGTACCAGCGGCTGGGGAAGG
ACTACGACGAGAGAGTCCTGCCCTCCATCGTCAACGAGGTCCTGAAGTCTGTGGTGGCGAAGTTTAACGCCTCGCAGC
TCATCACACAGAGAGCTCAGGTGTCCCTGCTGGTGCGCAGGGAGCTGTTTGAGAGAGCCCAAGACTTTAACATCATTC
TGGACGACGTGTCCATCACCGAGCTGAGCTTCAGCAGCCAGTACACCGCTGCAGTGGAGGCCAAGCAAGTCGCCCAGC
AAGAGGCCCAGAGAGCCCAGTTCTATGTGGAGAAAGCCAAACAGGATCAGAGACAGAAGATCATCCAGGCTGAAGGAG
AGGCTGAAGCTGCCAAAATGTTGGGTCAAGCAGTGACTAAGAACCCAGGTTACCTGAAGCTGAGGCGAATCAGAGCGG
CACAGACCATCGCTAAGACGGTGGCAACATCTCAGAACAAGGTTTACCTTAACGCTGAAAGTTTGGTCCTGAACTTAC
AAGACCAGGTGTCTTTTAACAATTTGTCTCTGGGTCCAAAGAAGTAATGAAGAGGACGTGGAGAGAAGCAGCAGAAGT
TTCCTGTCGTCGGCTCCGTCTGAACACTCAGTTTCACCTGTTGCATTAGAGGATCAACATACATGACTCTAGACACGT
CTGAATGTGGACAACATCAAAGAAACAATTACATTTTTTTATGTTTATTTTTTTTGTTCTTCTGACATAATATATAAG
ACTTTAGGGAGTGGCCTGTATTATGCTAAAGTTTTGTGAATGAATTATTGACAACGTGATCTCATTTCAACTTGACAG
TGAACAAATCAAAGACGTGTCAAACAGAAAGTACTTCCTGCACAGGATCAGTGTGTAAAAGGACTTTCATGAAGAAGA
ATTAGTGCAACAATACTTGAGAATATTTAGGAGATCAGTGTCTTCTGTCAGGAGGAGCTGCCAGGTCTCATGTCCAAA
ATATCAAACAAGCTATCATGTATTAAATGACGTATGTTTCACTGCTGTGTTAATCTCCCAGAGTTGGTCCTGATCTGG
CTGCCAGTCCTTCCTCTGCTCTACACACATCTCATCCTCACAGGTTTGACACTGCAGTGTTTGTCAGGGGTGTTACAC
ACGTTAGGAGGTGTGTGTGGATTCATGCAGAAAACCAGATAACACAGCCAGTGTTGGCAGACCAACTTAATACTAATT
TATCTGTTAATAAACTTTTGTTTTAAGGACTAAAGAAAAAAAAAAAAACCGAACCTACCCTAACTA

>Sequ15682EST2
GCTGGCCTGCGTGTCTGACCTTCATAGGAGTAACCATGTTCGCCAGGACCAGCGCGTTGGTGTTCTCCCCACAATGTG
GGCAGGTCAGGAACATGGCTACCTTGAAGGACATCACCATTCGGTTGAAGTCCATCAAGAACATCCAGAAAATCACAA
AGTCCATGAAAATGGTGGCCGCTGCCAAGTACGCTCGTGCTGAGAGGCAGCTGAAGCCAGCCCGTGTTTATGGCACCG
GTGCTCTGGCTCTGTACGAGAAGGCTGAAATCAAAGCTCCCGAGGACAAGGCCGCCAAGCATTTGATCGTTGGTGTGA
CCTCTGACCGTGGCCTCTGTGGTGCCATCCACTCCGGTGTGGCTAAGGCCATTAAGAGCGAGATCGCCAACCTGACTG
GCGCTGGCAAGGAGGTGATGGTGATCAATGTGGGAGACAAGCTGAGAGGCCTGCTGCACAGAACTCATGGAAAGCACA
TCATGCTGAACTGCAAGGAAATCGGCCGCAAGCCCCCCAGCTTCGGTGACGCCTCCATCATCGCCACCGAGCTGCTCA
ACTCTGGATACGAGTTCGACCAGGGCTCCATCATCTTCAACAGATTCAGGTCTGTTATCTCATACAAGACGGACAACA
AGCCTGTGTTCTCCACAGACATCGTTGCCAACTCAGAGAGCATGGGCGTCTATGATGACATCGATGCCGACGTGCTGA
GGAACTACCAGGAGTTTGCTATGGTCAACATCATCTACCTGGCCCTGAGGGAGTCCTCCACAGCAGAGCAGAGCGCCA
GGATGACTGCCATGGACAGCGCCAGCAAGAACGCTTCCGAGATGATTGACAAGCTGACCCTCACCTTCAACCGTACCA
GACAGGCCGTCATCACCAAGGAGCTCATTGAGATCATCTCCGGAGCTGCTGCTCTATAAACGGGCCAGGTCATCGTTT
CCATTGCCTAAAGCTCTTCAAAAAAAGTACTTCGGACAAGTGTTGTCGAGAGTCTAAATGAACATTTGTGCTTCTATT
TGTAAAATATATGGAGAAAATAAACCTCTTACACAGAACCTTCATCCTTTCCTCAGTCCCTGTATCTCATTCCTCACA
ATAAAACCTGACGACTGAGAAAAATGAATGTTGATGCTCTTCTCTTCTGAAGGTGTCGCTACTTTTAATCATCAGTT

TGTGTTTTTGAATTTTCTTCTAGAAAGGAATTTAGTCTCAGACGCTTAAGAGTTGTTCGACGGTGAATTATTCATGTG
GATTGTAAATCTAATTATTCCTCAGTTGTGTTATTAAACAGAGGATGGACAAAATAGCTGAAACACGTTATGTAATCC
AAACAATAAATAAATAAATAAAT

>Sequ15684EST2
AGCAAGATTCAGCTGGAGGCCAAACTCAAAGAGACATCTGAGAGACTGGAGGATGAAGAGGAAATCAATTCTGAGCTT
ACCGCTAAGAAGAGGAAGCTTGAGGATGAATGCTCTGAGCTCAAGAAGGATATTGATGACCTGGAGCTTACCTTGGCC
AAAGTGGAAAAAGAGAAACATGCCACTGAGAACAAGGTGAAGAACCTGACAGAGGAGATGGCTTCTCAGGATGAGAGC
ATTGCTAAGCTGACCAAGGAGAAGAAAGCCCTTCAGGAGGCTCATCAGCAGACTCTTGATGACCTGCAGGCAGAGGAA
GACAAAGTCAACACTCTGACCAAGGCCAAGACCAAGCTTGAACAGCAAGTTGATGATCTTGAGGGATCTCTGGAGCAA
GAGAAGAAGCTGCGTATGGACCTTGAGAGAGCCAAGAGAAAGCTTGAGGGTGATCTGAAACTGGCCCAGGAATCTATC
ATGGATCTTGAGAATGACAAGCAGCAGTCTGAGGAGAAACTGAAAAAGAAGGACTTTGAAATCAGCCAGCTCCTTAGC
AAGGTTGAGGATGAGCAGTCAATGGGCGCTCAGCTTCAGAAGAAGATCAAGGAGCTTCAGGCCCGTATTGAGGAACTG
GAGGAAGAGATTGAGGCTGAGCGTGCTGCTCGTGCCAAGGTTGAGAAGCAGAGGGCTGACCTCTCCAGGGAACTGGAG
GAGATCAGTGAGAGGCTGGAGGAGGCCGGTGGTGCCACTGCTGCTCAGATTGAGATGAACAAGAAGCGTGAGGCTGAG
TTCCAGAAGCTCCGTCGTGACCTTGAGGAGTCCACTCTGCAGCATGAAGCCACCGCTGCTGCTCTTCGCAAGAAGCAG
GCTGACAGCGTTGCTGAGCTGGGAGAGCAGATCGACAACCTCCAGCGTGTCAAGCAGAAGCTTGAGAAGGAAAAGAGT
GAATACAAGATGGAGATCGATGACCTCTCCAGCAACATGGAAGCTGTTGCCAAAGCAAAGGGAAATCTTGAAAAGATG
AGCCGTACTCTTGAGGACCAACTTAGCGAACTGAAGACCAAGAATGATGAAAATATCCGTCAAAACAATGACTTGAGT
GCACAGAAAGCACGTCTCCTGACAGAGAATGGTGAGTTCGGCCGCCAAATTGAAGAGAAAGAAGCTCTTGTCTCCCAG
CTGACCAGAGGCAAACAGGCCTTCACACAGCAGATTGAGGAGCTGAAGAGACAGATTGAAGAGGAAGTTAAGGCCAAG
AATGCTCTTGCCCATGGACTGCAATCCGCCCGCCATGACTGCGATCTGCTGAGGGAGCAGTTTGAGGAGGAGCAGGAG
GCCAAGGCTGAGCTGCAGCGTGGAATGTCCAAGGCCAACAGTGAGGTGGCTCAGTGGAGAACTAAATATGAAACTGAT
GCTATCCAGCGCACTGAGGAGCTTGAGGAGGCCAAGAAAAAGCTGGCTCAGCGCCTTCAGGAGGCTGAGGAGCAGATT
GAGGCTGTGAATTCCAAGTGTGCCTCTCTGGAGAAAACCAAACAGAGGCTCCAGAGTGAGGTGGAGGACCTCATGATT
GATGTGGAGAGGGCTAATGGGCTGGCTGCTAACCTGGACAAGAAGCAGAGGAACTTTGACAAGGTGTTGGCAGAATGG
AAGCAAAAGTATGAGGAGGGTCAGGCAGAGCTTGAAGGAGCTCAGAAGGAGGCTCGTTCTCTTAGCACTGAGCTGTTC
AAGATGAAGAACTCATATGAGGAAGCTCTGGATCAGCTGGAGACCATGAAGCGTGAAAACAAGAACCTGCAGCAGGAG
ATCTCAGATCTGACTGAACAGATTGGTGAGACTGGCAAGAGCATCCATGAGCTGGAGAAGGCCAAGAAGCAGGTGGAA
ACAGAGAAGTCTGAGATCCAGACAGCTCTTGAGGAGGCTGAGGGAACTCTGGAACACGAAGAGTCTAAGATCCTGCGT
GTCCAGCTGGAGCTCAACCAGATTAAGGGTGAGGTGGACAGGAAGCTCGCAGAAAAAGATGAGGAGATGGAGCAGATC
AAGAGGAACAGCCAGAGGGTGACTGACTCCATGCAGAGCACTCTGGATTCTGAGGTCAGGAGCAGGAATGATGCCTTG
AGAATCAAGAAGAAGATGGAGGGAGACCTGAATGAGATGGAGATTCAGCTGAGCCATGCCAATCGCCAGGCTTCTGAG
TCCCAGAAGCAGCTGAGGAATGTGCAGGCACAACTGAAGGATGCTCAACTGCACCTTGATGATGCTGTCAGAGCCCAG
GAAGACCTCAAGGAACAGGCTGCTATGGTGGACCGCAGAAACGGTCTCATGGTGGCTGAAATTGAGGAACTTAGAGCT
GCTCTGGAACAGACAGAGAGAGGTCGCAAGATCGCTGAGCAGGAGCTGGTGGATGCCAGTGAGCGTGTTGGACTTCTG
CACTCTCAGAACACAAGCCTTATGAACACCAAGAAGAAGCTTGAGACTGACCTGGTCCAGATCCAGAGTGAAGTTGAT
GACACTGTTCAGGAAGCAAGGAATGCAGAGGAGAAGGCAAAGAAGGCCATCACTGATGCTGCAATGATGGCTGAGGAG
CTGAAGAAGGAGCAGGATACTAGCGCTCACCTGGAGAGGATGAAGAAGAACCTGGAGGTCGCTGTTAAGGACCTGCAG
CACCGCCTGGATGAGGCTGAGAACCTGGCCATGAAGGGTGGCAAGAAGCAGCTCCAGAAACTTGAGTCTAGGGTGCGT
GAGCTGGAGTCAGAGGTTGAGGGTGAGCAGAGACGTGGAGCAGATGCTGTTAAGGGTGTCCGCAAATACGAGAGGAGG
GTGAAGGAGCTCACCTATCAGACTGAGGAGGACAAGAAGAACGTTACCAGGCTGCAGGATCTGGTTGACAAGCTGCAG
CTCAAGGTTAAGGCCTACAAGAGGCAGGCTGAGGAAGCGGAGGAGCAGGCCAATGTTCATCTGTCCAAGTGCAGGAAG
GTCCAGCATGAGCTGGAGGAGGCTGAGGAGCGTGCTGACATCGCTGAGTCCCAGGTCAACAAGCTGAGAGCCAAGAGC
CGTGACTCTGGCAAGGGA

>Sequ15694EST2
CGACAATCCAGACGACGGGGAGTTTCTGGGCATGAAGGGGATAAAGGGTCAGCTGGGCCGACAGGTGGCCGACGAGGT
GTGGCAGGCGGGGAAGCGTCAGGCGTCTAAAGCCTTTAACCTTTATGCCAACATCGACATCCTGAGGCCGTACTTTGA
CGTGGAGCCGGTTCAGGTCCGCAGCAGGCTGATCGAGTCCATGATACCTGTCCGCATGATCAACTTCCCCCAGAAGAT
CGCAGGTGAGCTGTACGGTCCTCTGATGCTGGTCTTTACCCTGGTGGCCATCCTGCTGCACGGGATGAAGACCTCAGG
AACTGTCATAAGGGAGGGGACTCTGATGGGCACAGCTATAGGAACGTGTTTCGGGTACTGGCTCGGCGTTTCCTCCTT
CATCTACTTCCTGGCGTACCTGGTCAACGCTCAGATCACCATGCTGCAAATGCTGTCCCTGCTGGGTTACGGTTTGTT
TGGTCACTGCGCCGTCCTCCTCATCACCTACAACATCCACTTCCACTTCCTGTTCTACGGTCCTGTGGCTGCTGGTTG
GAGGACTGTCACCCTGCGTATGGTGCGGCTCTGCTGTCTCGTACAGTCGGACAGACGCCTCGTCTCCTCCTCTGTGGG
ACTCTGTCTCTGCTGCACATGCTCTTCCTGCTCTACCTGCACTTCGCCTACCACAAGATCGTAGAAGGGCTGCTAGAC
ACTCTGGAAGGACCCAACCTGGCTCCCATGCAGCGTGTGGCCAGAGACGTGCCTGAACTGACACTCAACGCCACATTG
AGGAACCTGGGGGCCTCACTGAGGGTCCACTGAGGCCCCCACTCTCTCATAGACGCTCTCAGTCATCTTTTAAAAGAA
GAAAGTTTCTGTTTCACTATAAAAGTTCATTGGTGGGAAACCTAAAATAACAGAATGATCACAACTCAAAGGTCTGAG
TGGAATCACATCAGCATGTGACAACTGAGCAGATGAAGACCATGACGGCTCCTGAAAAAGCTAATAAGTGGCCTTTGA
GTAGAGATGATTCTGTGTTAATGGCCTCTGAAGATGAAGCTTCATGTTCATGAAGTCAAACGTTTTCTCTCTCAGCTC
TTGTACATTTTTTATTCATTCTGTAACATTTTGTTTTGGCTTTTGTGTTGTTCTCTTCATGTTCAGATGTATTTATAG
TTTCTGTACAGAAACCCCGAGTAAACTTTATGGACTTTGGTTGGAGTCACTGAGATAATCCACACTTTATTTAGTAAA
ACGACATCATTTAAATTTACAAACAGATTCTTTCTCGACATTTTGGTATTTCATCTATCAGCAGAGTTTCAAGATGAA
TGGTTGCCGTCCTCAAAACATTATAATGATCAAGATTATTTAATTGTAAGCAGAAAAATCTGAGGTCAAACAGCAGCT
TAATTCAGACTTAATGCAGAGTTCACGCTTCATTTGTAAATATAACTCCACCATTAAACTAATTCACAAGACAAAAGT
ATCATCAGAATATAGTTAAAGACCAAAAGTAAAAGTACCCAGTATGCAGAGTCATATATAATGCTGTATTATAAATAC
TGATGCATTAATGTGTTCATCACTTTAATGGTTTATTTGAATTATTTTATATTCTGCTGGGTAGTTTGTGAATTTCCC
CTGTGGATGAATAAAGTTTTATCT

>Sequ15704SNP2
TCGATTGCCTTTGTTCCACTTCGATTGTAAGGCATTTATGGATGAACCATCTGATTTCAAGGAAGGCATAACTCTTTC
TAGTCAGTCTTCAAACGGAACCCTAAAATTGAACGAGGGGGGACAAAATTGCTCACGTGAAATTAAAAATGTTGTTTT
TAAATCAGCACATTCTCAGGCAATGTAAACGCACTGGCCAATCAAAATCTTTGGGATGCGTTTGCAGAAAAACAATTA
ATCCAACATCTTAC

>Sequ15758SNP2
CGGATGTGTCTGACTAGTGTTAGCTAGAGGACACAGTCCTGTCTCAGTGCGGCGCTGACCTCCGGCTGTCGATGAGAA
AAGTCAAATGGCGGGGAGCAGACTAGAGAAGTTTGGAACCGTATTCACCCGGGTTCGAGACCTGATGCGCTCCGGAGT
CATAAAGCCGTCAGAGAAACCCGTCTGGTATGATGTGTACCAGGCTTTCCCACCGAAGAGGGACCCGCTCCACGTGAA
GCCACACTTCAGACCCAGCACCAAGAAACAGGAGACGGTGCCTGAGATCTTCTACAGAGAGGATGAAGTGAGAGCGAA
GTTCTATGAGCGCTACGGGGCGGGGCGGCCTCTCGATCTCGCCAAATCAAACTTTGTCTCTACGTGTCAGAGATTTGT
GGACAAGTACACGGAGTTAAAGAGCCGCAGCGAGCTGGATGACTCCGCCCTGTTTGAGGAGACTGGGAAGGCTTTACT
CGCCGAGGGCATCGTGCTGAGGAGGAGAGGAGCTCCTCCTGTGGCAGCAGAGTCCAGGGATCCGGTGCTGGAGCTGAA
GCTGACAGACATGCTGGCAGAGCAGCAGTCAGCCGGTGTGGACAGCAAAGAGACAGTGGACCACAGCGCACACACACA
CACATCATAGACTTCAACTGGAGAGCGAGTGGACGAGCTGCAGATTCTGCACTCGAGACGTGTGAGCGACATTAGACG
CTGTGGTCGTGTTGCTGCTCCTCCAGTCCGTCATGGATCTGCTCTCTGGACTGTGTGAAGACCAGCTTCATGTTTCCT
GACGTCACTTAGATATAAATTCACTGCGAGCCACAAACGCTGAGTCTACATCACAGCAGAGGATGGGCTGTAAAATAC
CTGGACATGGTCTATGCACATGTTTTATATATATAACACATGATTTATGTACAGACAGGTGCAAATTAAAGGAAGAAC
CAACATACAGTGTGTTAGTCAGGTGTTGTTCATCCCGTGTCTCCAGAGCAGCGTCACTGCTCCAGGTCTCGTATTCCC
TCATGTGATGACGGAGGTGCAGAGCGCCGTCTGACACGTCGCTCCAACATAGCCCATGGGTNTTCACCTGGACTGATG
GGAGGGGTCTGTTCCAGGACGACAGTGTCTCCCACCCACAGGACACCAGGGCTCACTGA

>Sequ15767EST2
GGACTTTCTCCTCCAGCTGAAGCAGAAAGACAGCCTTCTTCACCCTCATCACCAGACATCATGACGTCCATGATGATG
CGAAGCTCCAGGTCCATGGGTGGCGGCGCCTACAGGGCCAGCGCCGGCTCCATGCACGGAGGTGCTGGTGGCAGCAGT
GTCCGTATCTCCTCTGGCGGCTCCTCCATGTCCATGTATGGTGGTGGCGGCGGTGGAAGTGGTGGAGCGTCCTACAGC
TTCAGCTACGGCGGTGGAGGTGGTGGCGGCGGCGGGTTTGGCGGGTTCGGTGGCGCCGGCGGCGGCGGCGGCGGCGAC
AGCATGGACATCTCGGCCAACGAGAAGGCCACCATGCAGAACCTGAACGACCGTCTGGCCACGTACCTGGAGAAGGTC
CGCAAGCTGGAGGCGGCCAACGCCGAGCTGGAGCTGAAGATCAGGCAGTTCCTGGAGAGCAAGACCGCCCCCTCCTCC
AGAGACTACAGCGCCTTCTACGTCACCATCGCAGAGCTGCAGGGAAAGATCCAGGACGCCACCAAAGTCAACGGTGGC
ATCTACCTCGCCATTGACAACGCCAAGCTGGCCGCCGACGACTTCAGGCTCAAGTTCGAGAACGAGCTGGCCATGCGT
CAGTCGGTGGAGGCCGACATCGCCGGGCTGAGGAGGGTTCTGGACGAGCTGACTCTGGCCAGGACCGACCTGGAGATG
CAGATCGAGGGTCTGAAGGAGGAGCTGATCTTCCTCAAGAAGAACCACGAGGAGGAGCTGCTGGCCATGCGAGCTCAG
ATGAGCGGACAGGTGAACGTGGAGGTGGACGCCGCTCCTCAGGAGGACCTCAACAAAATCCTGGAGGAGATGAGAGAG
TACTACGAGTCCGTCACCGCCAAGAGCCGCAAAGACCTGGAGGCCTGGTTCCAGGCCAAGTCAGAAACACTAAACAAA
GAAGTCGCCGCCAGCACAGAAACCATCCAGACGTCCAAGTCGGAGCTCACAGAGATCAAACGGACGCTGCAGGGCCTG
GAGATCGAGCTGCAGTCACAGCTCAGCATGAAAGCCGGTCTGGAGGGAACCCTGGCTGAGACTCAGAGCCGGTACGCC
ATGCAGCTGGCCGGTTACCAGAACCAGGTAGTCATGCTGGAGGAGCAGCTGGTGCAGCTGAGGGCCGACCTGGAGCGT
CAGGGACAGGAGTACCAGATGCTGCTGGACATCAAGACCAGACTGGAGATGGAGATCGCCGAGTACAGGAGGCTGCTG
GACGGAGAGGGCGGCGGCAGCATCTCCACCTCCTCCTCCTTCTCCTCCTCCGCCACCTCCAACTCCAACTCCACC
TCCAACTCCACCTCCACCTCCTCCACCACCACCAAACAGAAAGTCATCACAGTCATAGAGGAGATCGTGGATGGAAAG
GTGGTGAGCTCCACAGAGACCGTCAGCTCCAGCGAGTGAGCACCAACTGCTCTCACTGCACTCTCACAAAAAATAAAA
GTCTGCTGAGGTGCTTCAAAAAAAAAAAA

>Sequ15789EST2
ATTTTCCACAGTAAAATTTATTGCTTCGGCTTTGCTCAGAGCCAGATTCCAAACTGAACAACGCTCAGACGCCTCGTT
CAGACAGCAACCACGTCTAACCACAAGTTGATTGAGCTTTTTTTTTCTTTTATATCACTGACATTGATCGCACTTTTC
ATGGAGACAAAAACATACTGACCTTGTGACATCTTTGAAGAAACACTGCGACACCATCATAGCTGCAGTGTAAAAAAT
CTAGTTGAAAACATACGCTGAAACAAGATCACCACTTTGCATAGGTCGACTCTTTCATCTCCAACAATGTACCATGGC
ACAGACACAACACTAGTACACAGAAACAAAACATTTTCTCCAGGTTTACTCTTTTGTTAATTTGTTAATATCAATAAT
AAAGTTTTACACGTTTGGCACACCGTACGAAACAATATCAAACGCACAATACTATGTGTCATTATAAAGGGATAAATG
ATTTATTAAATCAAGTCTTTGTTTCATCTGTACAACAAAAACAGTACATTGTAGTCTGGAGTTCCTGACCTCCGGGAC
ACTGTACACAGTCAAGCACATTTCAGCACGTTGGGTTGGAAAGGTAGAAAATATCACATCAGCACAACAAATCTTCAC
TACCTCATAACTCGAGAGAAATCATTTTCCATTTGTCGTCATGTTTTCATGTGGTTGTAATTTGTGGTGTATCCAGTA
ATCTTGTAGGAGAGATTTCCACAAAGAGGAAGTCGGAGCAGTACAGGTTAGTAACACATTACTTTTTAAAAAACGGTT
GAACTATTTCTGTAGCGAACGCCACTTTTTAGTGCTTAAAAGAGCAAGAAGGGCACACCAAAAAGTGAAGAGAATTAG
GCAAATACACTTTAAAATTATGCAAGAATTTTTGCAATCCTAACGCCACGTGGCTCTGTGGAAATGATCTGCTTCACT
GATCCGTTCATTAAATTTTCTTAAAGGTATTTCAGAGGAGGAGAAGCTTCTTCTTAGTGTCCGGACACAGTGAGGTGT
GTCATCTTCTTGGCAGCCTCTTCAGACACCACCTTGGATGTTTTCTCGGGGCTGGGAGCTGGACTGGGATTCGGAGTG
TTTGCTGCGCTGTTCGGATACTCCCTGTTGCCGAATATGATGCTGCCCACTCGCACGTTGGTGGCGCCAACCTCGATC
GCATGTTCAAAGTCTGTGGACATACCCATGCTGAGCTCCACCTCCTCCAGAGGCAGCTTCAGACTGTCACACACCTCC
TGCCTCCGACTCAGCAGCATTTGAAAGTCCGGGTTGGGACCCAGGGTGAGGTTGTAGCCATAGCGCCCGATGGTCATG
AGTCCTGAGAAGTGAAGGGCAGAACACTGGGACACGATGTGTTTCACGGTGTTCACCGTCTCCTCCGGGGGCAGGCCA
TGTTTACTCTGTTCTCCGCTGGTGTTGATCTGCACCATGACCTTTTAACCTCTGCATGACTGGCTCCCCTGAGCCGCT
GCCATGAGCTGTTGACCTTGTCGGCCAGTTTTCGCCGAGTCAACCGTCTCAACGAGGAACAGGTTTGGCACGCCCAGA
AGTTTGTTGACATTATTCTTCTGTAGGTGGCCGATGAAGTGCCACTTGATTTCGGGACATGATTCTAAAATCAGAGGA
TCTGAAGCTTTGTCCACAAGTTCATTAACGTAATTTTCTCCAAAGTTGCGCTGCCCTTGTCTGTAGGCCTCCACAACC
ATCTCTGGTGGTTTAGTCTTGCTGACAGCTACGAGGCGGGGCGGCACAGCCGGCAGTGTCTTGGGCCGCCGTGCCGCC
GCCTGTTTCACCCGATCCACTACAGACTGTAGCGCCTTCCCAACCTCCTCCGACATTGCTACTTTC

>Sequ15794EST2
GACTTCACGTGGAGGAACAGCACGAGCGTTTGATCCTCTGTCAGGACGAAGAATAAAGACACAAAATGACACAGTTGG
ACGGTATAGTAAAACCCAAGTCGAAGCGCTCCAAGCGGTTCTTGGAGAGCAGGGCGCCGAAGCTGACGGAGGATGTGA
AGACTGCCATGATCATGAAAGGAGGGAACACCAGTCAGACCATCACCCAGGCCCTCAAGGACCTATATTCCCTGAAGA
AACCCAATGCTGTGCTGTACAAGAAGAAGAACATAACTCGGCCATTTGAGGACTCAACATCGCTGGAGTTTTTCTCCA
AGAAGACGGACTGTTCTCTGTTTCTGTTTGGCTCTCACAACAAGAAACGGCCCAACAACCTCATATTTGGTCGTCTGT
TTGACTTCCACGTGCTCGATATGATTGAACTTGGGATCGAGAAGTTTGTCTCTCTGAGTGACATCAAGACCAGTAAGT
GTCCCGAGGGGACGAAGCCCATGCTTGTGTTTGCAGGAGAGGCTTTTGATATAGACAACGAGCACAAGCGTCTGAAGA
GTCTTCTCACAGACTTCTTCAGGGGTCCCACTGTGTCTGCAGTGCGTCTGGCAGGTTTAGAGCATGTGCTGCACTTCA
CTGCCCTGGACGGGAAAATATTCCTGCGCAGCTACAGGTCTCTGTTGAAGAAGTCCGGCTGCCGGACACCGCGGATAG
AGCTGGAGGAGATCGGGCCGTCGTTTGACTTTGTCCTAAGACGAACCCACCTGGCTTCAGATGACTTGTACAAGTTGG
CCCACAGACAGCCCAAGGCCCTGAAGGCCAAGAAGAAGAAGAACATTTCCCACGATGCCTTTGGTACCAAGTTAGGTC
GGGTGCACATGCAGAAGCAGGATCTGTCCAAGCTGCAGACACGCAAGATGAAAGGCCTGAGGAAGAGGAAGGGGGAGG
TGGTCGCCGAAGAGCAGGATGGACAGACATCCAAAGTGGCCAAAGTGGACAGCTGATGTCTGAGAGAGACAGCTGGAC
TCATTCACCACCTGTGTGTGTCCATCCCTTTGTTTCTGAGACTGACCATGTGCTGATCATTACCGGCTTTTTACTGTT
GGAGCGACCCGGCGTGATAGTGGCCACCAGGTCAGGATCCAGATCTATTACTTCTTTTCTTTCGTTTCAGCCGTGTTA
CATTCAGGTGCCTCTTTGTCTCGAGCGTTGTAAACGTGTCAGCTCTCGGCTTGTCTGGTGAACACAGTCAGTGTTGTA
TATGTACTGAATTATAAACAGATTTCTTCGTTAATTTGAAGTGTGTAAGAAAACAAAGAGATATTTTACTGGCAGCAC
TCGCTTTAAAGGAAATAAAACCTTCTACTCTTCCTGTTTTATCATTTGAAGTTTTTATTAATTTATTAAAATACACAC
AAACGGACATTATCATCTTTGTTTATGAGATATTCAAAAGTCTGTGATAATCTTTTTTTTTTCTTTTCCCAATGTTTA
TTTCCGTCCAGCATAAATAATTGGTCAGTAAACGCATCATGTGGAGCTTAGAAGGAGTAAAATAAAACCTGTGTATTA
TCTTATACTGAATACATTTCAATACATTTCCCCCTCAGCCTCCTTTTAATATATTTCTCAGTATTGGACATTATTTTT
AACCATTCCTCGTCTCCAAGATCACACATCACAGATTACAGATGTCTTTTTGTAAAACCACCAAGGTGCACCTTGGTG
GTGACATTTTCTTTATATTGATCCTTGGTCCCTTCTTGTCCCACAGGAAGTCCTTGATAATTCTATTAAATTGC

>Sequ15800SNP2
CTTAACTGAGTTAGAAATCATCAAACGTCCTTTTTCCTTTCCTCCATCCTCCTCTCCTCTTTTCTTCTCCTTTTGGGT
CAGAAAAGATTTAGAAAACAAACAAACAAAACTCAGAGTTTCTCGCTCCAGCCATTTGACAGACGACCACTGACACTC
AGCCTGCAGTAAACGAGCCGCTCGTATATTTATTTGGAAGTATGATTACCGGCTTGCCAAAGTAACTTTCATCTCTGT
GGAAATAGCTTGAATATAAAAGATCCTTTCATGCAGTTGAGTGAAAGAAGGGGGGGCAGATGATTTGAGAAATTAATT
TTCATTGCATGCTTGTGTACAGAGGAAAAGAAAAGTGATGCACTTTTGATTGATTTTTAGCCTCCTGCAAGTACTGCA
CAGCTCAGACAAAAAGCTTTTACAAAGTTGGCTCTTGATAAACTCTGAAACTTTGATCCTGCCTCTGCTGTTTGTGTT
TCATCTCACCCACAGCCCTGTTCATTTAGAAACAACCCCCAAAGCACTTTTTCTTTCCTGCTTGCTGAGGGCTGCTTTA
ACTTTTGGCTTCCTGTACCACTTAAGCTCAGCCTCTGGAAGAAAAAGTGAAAAGTCAGCCCAGCTCTTAAATGGTTTA
GGTACAGTTTACACAGTATGTCCTCTTGTTGGCTGAAGCCCTATGATCTGATCTCTGAGCAGTGGCTCCGTCCATCTG
TTTGAGTGAAAGCCTGAGTTCTCTCCTGCGATTGGTTGTATACGTGGTAGTTGTATCATATTCTTGCTTTTTAAATAG
CAAGCCATTATCTGACATTTGTAATAACAGGTTTAATTTAGTAAATGAATAACTCATTTATTGTATAAAATTATCATG
AACGTGTTCAATAATCAGTCTGTGTCACTTCATTTCCCTGACAGTCAATATTGATTCAGTGCAGAAATGTTGAAAATG
TCTGTTATCATTGTAGCTGTTCCATGTCAGAGACATGTCACCTTATGGGAATAGAATAAATAAATCTGGTAAATGACA
AAAAAAAAAAAA

>Sequ15827EST2
TAAAGTTAAGTTTTCAAAAGAAGGTGCACTAGCATCCAGGAGCTACCTCTGACAGCACCCTCTGACTTCACAAGCTCT
CCCTAAGTAAGAAGTTAAAAAAAAAAAAATGGGGGACCCATGGCAGAAGGCGTATGACTTTGCTGTTGCAGTGGCAAGA
AAAGCTGGAGCGGAAATTAGGAAAGCTGGGGAGAGTGAAATAAGGGTCATGACAAAAAGCTCCACTGTAGACCTTGTC
ACAAAGACTGATGAGAGGGTGGAGAAAATCATCATCGGGTCTCTTAAAGAGGAATTCGGAGAAGGCACACACTGCTTC
ATTGGGGAGGAGTCGGTGGCGAAGGGGGGAGCCGTGTGTCTTAACCGACAAACCCACGTGGATCATTGACCCGGTGGAC
GGCACCCACAAACTTTGTACACGGATTCCCATTTGTGGCTGTGTCAATTGCCTTTGCGGTCAATAAGGAGTTGGAGTTC
GGTGTGGTGTACAGCTGCTTGGAAGACAAGATGTATAAAGCAAGGAAGGGGAAAGGAGCTTTCTGCGACGACGAAGCA
ATTCAAGTGTCCGATGTAGCAGAAATCAAGAAGTCCATTATCATTTCTGAGCATGGAACCGACAGGAGCCCAGAAAAA
GTAACCAAGATCTTCTCTACCATGCAGAAGATCCTCTGCATCCCCGTGCACGGGCTCCGTGGATCAGGGACAGCTGCC
ACCAACATGTGTCTGGTGGCGTCGGGGGCAGTGGAGGCCTTCTTTGAGATCGGCATCCACTGCTGGGACATCGCTGCT
GGTGCGGTGATAGTCAAAGAAGCTGGAGGAATATTACTGGATGTTGATGGCGGACCATTCGATTTGATGTCCCGAAGG
ATGGTTTCAGCAAACAACGATGTTATTGCTAAGCGCATCATCAAAGAAATTGAGGCGTTCCCAGTGGTGAGGGACGAT
GCTCCTGTGCAGAAGAAATGAGAATATTCTCAAACGCTGTTTACACTGTGCACAAGTGCAGTCTGCCGCTATTTGTGT
CTGAATAAAAGTTACTGTT

>Sequ16441SNP3
GTTTAAACATGGATTTGCTGTATGGTCCATTGAGCTTGGGTGTAGTAGCAGGAGTGGGCTGTGGGCTGTTCCTCGGTT
GGCACCTTCGGGGACGATTCCGTCCAGCATCCCAAAGCATCATGGCGGCGATGGGGAACGGCAACAGTGAGGCAAGTG
TGATGGGAGAAGGAGGCGAATTCAAGATGATTCTGGTGGTCCGTAATGACCTGAAGATGGGCAAAGGAAAGGTTGCTG
CCCAATGCGCCCATGCTGCTGTATCAGCTTACAAGCAGGTCCAGCGCAGGAACCCAGAGCTTCTCAAACAGTGGGAGT
ACTGCGGCCAGCCCAAGGTGGTGGTGAAGGCCCCCGATGAGAACACCCTGATCGATCTGTTGGGTCACGCCAAAGAAG
TAGGGCTTCCTGTCAGCCTGATCCAGGATGCAGGAAGGACTCAAATTGCACCTGGATCTCGCACTGTGCTGGGTATTG
GTCCAGGCCCAGCGGATCTGATTGATAGTGTTAGTGGAGAGTTGAAGCTCTACTAGGTTTCCAGCTTGCACCTGTTGG
ACTTGCATACAATTTCTTTGTTAAAACCATTTTGCAAAGTAAACTAACTTGAGCTACCATGAGTTTATATTTTTATAT
AAGAACAACCGACTCAATTACATGTCATCTACTTGAATTTATTGTGCAGTTCTGGGGGTTTTATAGCAGTAATAACAG
TTTAATGTGTATTTCTTCTTCTTTTCTGAAAGCTATGACCTGGCATAAACACAGTCATGCTGCTGTCCTCCACGCCAA
CCAGTCTGAGACACGATGCTCACAGGAAACGGATGATAAACATGAATTGTGTCAAATATAAACAGCAAAGCAGTAATT

GAATATTATTGTTGTTGGTCTCAGAATCACAGCACTGCAAAATGATACCGAGCTATGATCCCAAAATATGTTCTCTCC
ATGATAAAAATCACAATCTAACT

>Sequ16779SNP3
ACCAACACCATGGCAACAGGACTACGTACCCCCCGCACCCCTGCTGCCCAGGACAGGATACTACAGGAAGCCCAGAAC
CTGATGGCTCTGACCAACATCGACACTCCCCTGAAGGGAGGCCTCAACACTCCGCTGCACGAGAGCGACTTCAGTGGA
GTGACGCCTCAGCGCCAGCAGATACAGACACCCAACACTGTTCTCAGCACACCATTCAGAACTCCCGGACCAGGTCAA
GGGTCAGAGAGTATGACCCCTCAAGCCGGAGGAGTGATGACACCGCGTGGGGCCGTCACCCCAGGTTTGACCCCTGGC
CGTACACCCCTGAGAGACAAACTGAATATTAACAGTGAGGAGCAACTGACTGACCCTGCGTACGCTAAACACATGCAA
AGGGAAAGCCTGCAGCAGCTGAGGCAGGGACTGATGTCACTTCCTGTTCCTAAGAACGATTTTGAGATTGTTCTTCCA
GAAAACGCAGAGAAAGAACTCGAGGAAACAGAAACGGACACCGGGTTCGTGGAGGACTCGGCCGACATAGAGGCGCGC
AAGCAGGCTGTACGGGAGGCAGAAAGAGAGAAGGAGCTGAAGCTGCGACACACTGCTGTTCAGAGGAGTCTCCCCAGA
CCTACTGAGGTAAACGAGTCCGTCCTCCGTCCCGCCTCCATAGAGGCGCTCCCTGACCTCCAGCTGGCCGAGGAGCTG
ATCAAACAGGAGATGATCACCATGCTGCACCACGACTGCCTGCACCACCCGTCGGCCAACGCCGCCAACCAGCTGCAG
CGCGGCAAAAGCAGAGGCCCCACTCCACATCTAACAACGCGTCGCACATATCTTACCTGGAAACACACCCCTACAAGC
CGATCAGCACAGAGGAGATGGAGCAGGCTAAAGCAATGCTGGCAGCAGAAATGGAGGTAGTGAAGACAGGAATGGGCC
ACGGTGACCTCAGCATGGAGGCCTACAGCCAGGTGTGGGAGGAATGCTACGGACAGGTGTTATATCTACCTGGTCAGA
ACAGATACACCCGAGCTAACCTGGCATCAAAGAAAGATCGTATCGAAAGCCTGGAGAAAAAACTAGATGTGAACCGTG
GTCACATGACAGCGGAGGCCAGGAGAGCAGCCAAGCTGGAGAAGAAGCTCAAAATCCTGCTGGGAGGGTTTCAGTCCA
GAGCGCTGGGGCTCCTGAAGCAGCACAATGAACTCTGGGAACAGGTGGAGCAGGCAGCCACAGAGCTCCAGACCTTCA
CTCAGCTGAAGAAACAGGAAGATACGGCCATTCCCAGGAGACAAGAGGGTCTGCGGGAGGACGTGGAGAGGCAGATGG
AGAGAGAGCGAGAGCTCCAGCAGAGATACGGAGAGTTGCTGATGGAGAGGGAGTCGCTGATCAACAGCGCTCAGAAAT
ACTGAGCAACACACACGCTAATCACACACACACACACGCGCGCTACACACACTCAAATCCTCCTCATGGAGACACGGA
TATCAAGCGAACAGCAGCTCTGAGGAACACAAAGGTTTCCGAGTCGACGTACTAAACCCTTCTATCATTTTTCAATTA
TTCAATTGTTGCGGCATTTCCAGAGATAAGCCCCGCTCCATGTGCTCATGTTGCTATGATGTCACACGCTGAGTTTTT
GAGCATGCTCAGTAGCCCCTTTT

>Sequ16785SNP3
GTGTTATTTCCGGTGCAAAGGTCGTAGTTACTATAGGTCAATGTGTCCTTCCACACGTGTGTTTCTGTGATCTTCCAG
CAGCAGTAACGGGGACTCAGCCGGTCTGTGTGCTGTCAAACTCATTTATTCTGTCGTTACACGCTTAAATATTTCTAA
CATGCTAAAGTCGGTGGTCCGCGCGGTGGGAGCCGCTGTCCGCATGTCCTCAGCCACCACGTCCACAGCCCGGGCTCT
GCCGCTCAGCTGCCCGACTCTGTGCTCTCCGTGTTCGGCTACAACGCGGCCCTTCACCCGGTCTCTGTGGATGCTGAA
CAGCAAAGGAGCCTCGTCAGGATACAGGCCAAAACTGTTCAGTTCAAAGGTGTTGCAACCCACGGTGTCGTGTGGATG
TGGAGGACTGCACACAGAAGGTGACAAAGCATTTGGTGATTTCCTGTCTGATGAACTCAAAGAAGAGAAGAAGATCCA
GAAAAACAAAACTCTTCCTAAGCTGTCTGGAGGATGGGAGCTGGAGATGAATGGCACAGAAGCTAAA-CTCACAAGAA
GTGTTTCTGGAGAGAAAA-TCTCTGTCTCATTCAACGTCAACAACAGCATTCCTCCTAACTTTGAGGAAGAGGCAGAA
CAAGGTCAGCAGAAGTCAGCAGAGGAAGAGCCTGAAATTGTGTCAACTCCCAATTTTGTTGTTGAAGTCACAAAACAG
GCTTCAAAACATTCCCTGGTGTTTGACTGCCATTTCCCTGAAGACGAGATGAGTCATGGCGAAGGAGAAGAGGAGAGC
GACATCTTTGCCATCCGTGAGGTCAGTTTCCAGCCTGAGGGAGATACAGACTGGAAGGAGACCAGCTACACACTCAAC
ACAGACTCTCTGGACTGGGCCCTGTATGACCACTTGATGGACTTCCTGGCCGACCGTGGGATCGACAACACCTTTGCT
GATGAACTGATGGAGCTGAGCACCGCCATTGAGCATCAAGAGTACATTAAGTTCCTGGAAGACCTCCAAGGCTTTGTC
AAATGTAACTAATGTTAGATAGAAGCTAAATTTCACTTTTCTTCAGTACACATCCGGAGATAGGATGATACAGTAAGA
CTAAAGCAAATGAAAGCACTGAATGATTGATTTGTTCAGCGCACGAAGGAGACTGATGGTTGTGTCTGTGACAGTGAG
CCTTGTTGCCAGCAACTGTCACTAAGACTTATCTGCAAACATCTTTCCTTTGGTGAAATGTGACTTTATTTTTCTGTA
ACTTAAACAACATAGTTTAAATTAAATATTTAAAGGCCATAAATAATGTAGGTCATGATAATCGTGCAGAAACCGTCA
TTGTAAGAAATGTCTCTCAATTTTAGAGTTCCTGTCTTAACTGCACGGCTTCAGGAGGGTCCAGTCAGTATTTATGAA
TGTTCACTTTCCAATAAAGGACAAATTTGTCAAGGTGCAAATTCAAA

>Sequ16798SNP3
CTTATCATCGACACCGGGGCGTCACGAGCGCCTTCTCTGCCTGCCCGCACGAGAGAGACACAGCACCGTCCTTGCACA
CAGTTAGGCCGGAAAGATCCAGAACCATGTCCATCCTGAAGATCCACGCCCGTGAGATTTTTGACTCCCGTGGCAACC
CCACTGTGGAGGTTGATCTCTACACCAAGAAAGGTCTTTTCAGAGCTGCAGTACCCAGTGGTGCTTCCACAGGCATCT
ATGAGGCTCTGGAGCTCCGCGACAATGACAAAACACGCTACATGGGCAAAGGTGTCTCAAAAGCTGTTGAGCATATCA
ATAAAACAATTGCACCTGCACTGGTTAGCAAGGATGTGAGTGTTACGGAGCAGGAGAAGATTGACAAGCTGATGCTGG
ACATGGATGGCACAGAAAACAAGTCTAAGTTTGGTGCTAATGCCATCCTGGGCGTCTCCCTGGCTGTGTGCAAGGCTG
GTGCAGCAGAGAAGGGTGTCCCCCTCTACCGCCACATTGCTGACCTGGCTGGCAACCCTGAAGTCATCCTCCCAGTCC
CTGCTTTCAACGTCATCAATGGCGGCTCTCATGCAGGCAACAAGCTGGCCATGCAGGAGTTCATGATCCTGCCTGTGG
GAGCAAGCAGCTTCAAGGAAGCCATGCGTATTGGTGCTGAGGTCTACCACAACCTGAAGAATGTCATCAAGGAGAAAT
ATGGCAAGGACGCCACCAATGTAGGGGACGAAGGAGGCTTCGCCCCCAACATCCTGGAGAACAAGGAAGCTCTGGAGC
TGCTGAAGAACGCCATCGCTAAGGCCGGCTACACTGATAAGATTGTTATTGGCATGGATGTGGCCGCCTCAGAATTCT
ACAAAGGTGGAAAGTACGACCTGGACTTCAAGTCTCCTGACGACCCTAGCCGCTACATTTCCTCTGACAAGCTGGCTG
ACCTCTACAGGAGCTTTGTCAAAGATTACCCTGTTGTGTCCATTGAGGACCCCTTTGACCAGGATGACTGGGAGGGAT
GGTCCAAATTCACTGCCAGCACCAGTATCCAGGTGGTGGGTGATGACCTCACTGTCACCAACCCCAAACGCATCGCCA
AGGGCGTGACTGAAAAGGCCTGCAACTGCCTGCTACTCAAAGTCAACCAGATTGGCTCTGTCACAGAGTCCCTGCAGG
CCTGCAAGATGGCCCAGAGTAGCGGCTGGGGTGTGATGGTTAGCCATCGCTCTGGAGAGACAGAGGATACCTTCATTG
CTGACCTGGTGGTCGGTCTCTGCACTGGACAGATCAAGACAGGTGCACCCTGCCGATCCGAGCGCTTGGCCAAGTACA
ACCAGTTGCTCAGGATTGAAGAGGAGCTCGGCGACAAGGCCCGTTTTGCTGGCCAGAACTTCAGGCACCCCATCTGAG
CAGGTCTTGTTCCACGGGCCACTGTGTGTTCATTGCTCATAAATAACCCCCACACATATACGCCACGCTAGGTCGCCT
GTCCTGTCTGGAGAGAAGATGGAGAAAAACACCGCTGAAATCCAAGGTCCAAGTCTGTGGTCTGTGGCGTGGAGAAGC
TGCAGTACGTCTTAGTACAACCACAGTTCAGTTCATCCTAAAACTATGCTTTAGCTCCTGATTAGTGTCGGTCCCTAT

GTTTGTGTCCGTATAACTGTGTGAGAGATTTTTCTTAGCTTCTTGTGAGTTGTTGTAATTTTTGGTCCTCTACTGAGT
ATTTGAGCTACTGTAACTTTAGTATGGCTCTGCCTCGAGCAGTGGTAAGAGTACTGTATGTTCAATCTGTCGCATCTG
ACAAGGTCTCGTGTTGCTGTAAGCGTTTGGCAGT

>Sequ17206EST2
GGGGTTTTGAGCCCCGTCAGCCACCGTATCAGGCTGAGCATGAGACTGAGCTAAGCCAGTTATCGAAGGTTAACACAA
ACCTTTGGCTGCAACTTTAAGAAGCTGCAACCTGCAACTAAACTGTTGCTTTGTACATCGGTCCTGTGTTTCTGCTTC
TGTACATTTGCTGGCCACCGGCGAACTTTACAGACAGTAGGTAAGACACTTGTTTAAGAGCAGTTAGCATCAGTTAGC
TCTAGGCTAATGTTAATTAGTAGGGCAGCAGGCTAAGTGAATCAGTTCTCCCTGTTTGCTGCAGTTTTATTGATCAGT
AAAGACTCTGGACTCTGAGATCTGCGAACCTGCGAACCTGCGATGTTGTCAGTCATTGTCAGGTCAACTCTCACCAAA
TACAGGGGCTTGTGGGTGCCCAGTCAGTCTGTGGTCAGAGCCATGTCGTCTTCAGCTGCTCCTACCTCTCCTTACACC
ACCCTGGCCATCAGCCACCCGGCAGAGTCTGTCACACATGTGGAGCTTCACCGGCCTGAGAAACGCAATGCCATGAAC
AAAGCCTTCTGGAGTGAGATGGTGGATTGCTTTAATGAAATAGCCGGAGACCCGGACTGCAGAGTGGTGGTGGTTTCT
GGAGCAGGGAAGATCTTCACAGCTGGTATTGACCTGATGGACATGGCCAGCGACGTACTCCAGCCACAGGGCGACGAC
ACGGCCAGAGTTTCCTGGAACCTCAGACAAAAGATCGCCAAGTATCAAGAGACATTCTCCGTCATAGAGAAGTGTCCA
AAGCCTGTTGTGGTGGCCGTCCACGGAGCCTGTGTCGGAGGAGGTGTTGACTTGATCACAGCCTGTGACATCCGCCTG
TGTACCCAGGACGCCTGGTTCCAGGTCAAGGAAGTTGATATCGGGCTTGCAGCAGACGTTGGGACTCTCCAGAGACTT
CCTAAAGTCATTGGTAGCAGCAGCCTGGTGAACGACTTGGCTTTGACCGCCAGGAAGATGTATGCAGATGAAGCCAAG
AGCAGCGGCCTGGTCAGCCGAGTGTTTGCAGATAAGGAGGCCATGATGGCCGGAGCTCTGGAGATGGCCGATGAGATT
GCTGCTCGCAGTCCTATAGCTGTGCAGGGCACCAAAATCAACCTCATCTACGCCAGAGACCACAGTGTGGCAGAGGGA
CTGGACTACATGGCCACGTGGAACATGAGCATGCTTCAGACTCAAGATGTGATGAAATCCGCTCAGGCTGCCATGGAG
AAGAAGAGTCCAAAGACAATAGCCTTCTCCAAACTCTGAACCTCACTGAGGCACAGTTCAGATATAACACAATTGTGG
CCTTTCTGTGGGAAGGGGATCCAGTCTGTTCTACTCTGAGCTTTTAACACCCCCCGTTTTGTACATCTGTAAATGACT
TCATGAATGTTTTACCTTATTAAAAAGAAAACTCATTTGAAAAGACTAAAAAAAAAAAA

>Sequ17703SNP3
AGCCACATATCGCCGTCACTGCAGCGTACGTTTATACATAACCGAGCAGGTGGTGGATGACCTTCCTAGTTCGATGTG
CAACGTGTGAATCTTAATTTGTAGTGTTCAGCATGTTGTCAATGGATGACTTTGACAGCCACAGTTTAATAGCTATGA
CAATTTTTGTAATTTTTCGACACATCAACGTATTTTATTTTAAATCACGTGGTTTGTGTTTGCTCTTTTTTAAGCCAA
TGGGGTGTCACGCCGCCGCTAAACAAACCAATGAAAAATCGTGATGTCAGAAAACAGGGAGTGAAAGCTAGGTCGTCC
GACTACAGCACTGAGCTGTAAAATGTCGCCGTAAACAAACGGATAATACGTTAAAAGACGCGGTTTATCCAGCGGAGA
CAGGAGAGTACTTCTCTTCAATCCGTGTGATTGGTTTTCCGTTTCGGTCAGGCGATGGTGCGGGCTGTTTGGGCGGTG
CTTCGGAGAGTGTTCTGCGACAGGGCCGCCGTGCTGAAACCAAACGTACCTGCAACTGGGAGAGCCGCAGCACTGTGG
AGCAGACCCGTCCGCACGCCTATCATTACCTGCTGCAGAGCTTTAGCCAGTGAGGTGAGACACCTGCAGTCTACAGTC
AGAATGCCTGAGATACCCACAGATCACCTGGACGAGAAGCAGGTGCAGCTACTGGCTGAAATGTGCATCCTCATCGAC
GAGAACGACAGCAGGACCGGAGCTGATACCAAGAAAAACTGCCACCTCAACTCCAACATCGACAAAGGTTTATTGCAC
AGAGCCTTCAGCGTCTTCATTTTCAACAGTGAAGAGAAGCTGCTCTTACAACAGCGGTCTGATGCCAAAATCACTTTT
CCAGGCTGTTTCACAAACACATGCTGCAGTCATCCTCTACACACAGACAGTGAGCTGGAGGAGAAGGATGCTATAGGA
GTGAGGAGAGCTGCTCAGAGGAGACTCAAAGCTGAACTGGGTATCCCCGATGGAACAGGTGACACCAGATGAAATGAC
ATATCTAACCAGGATCCACTACAAGGCCCAGTCAGACGGTGTTTGGGGAGAACATGAGATCGACTACATCCTCTTCAT
GCAGAAGGACGTGGAGTTGAGTCCCGACCCCAACGAGATCAAAAGCCACTGTTATGTGAGCAAGGAGGAGCTGAAGGA
GATGTTAGAGAAGGCCAAGCGCAAGGAACTGGAGATCACACCTTGGTTCAGCCTCATCGCAGAGACCTTCCTCTTCAA
GTGGTGGGACAACCTGCAGAACCTCAAACAGTTCATGGATCACAACAACATCCACCGCATGTAACTACAACCAGGAAT
CAGCCTCTACCTGTTGTCCTCTCAAAACTCTGCTTGTTACAGTACTTGCCTGACATTCAAGCTAAGCTAGCCAAGCTG
CTGGAGACGCCTGACCTTCAGATCACTGGCCATTTATGTCAAGGTTGTTGTAAGTGTAGGATTTCTGCACTAAATAAG
GAATGTTTTAGCCTTGCTAAAAGTAAGAGGTGACATGGTTTACAGTAGGTTTGTAGCTCTGAGGTGCATTCAACAACA
CACAAGAGGTGATTCTATTGATTCTAGAGTCTTTAATAAAGAAAAATGTCATGTAGACAATTCTAATGGATTACAGAC
TGAATAGAAGATGTCGAATGTCTTAATTTATTAGGCAGAAAATGAAGAGAATAGCATATTTTTATATAAAAATGTGAA
GTGCCTGAGTACAGGTCTTTAAGCTGAGGTAAATAGAGTCTCCAAGTCACAACAACCACTGTCACTCTGTATTTGTTT
ACAATGATATCAGTGAACAAACTGAATCTTTGACCC

>Sequ18816SNP3
GCAGTGGGCGGAGCATACGTAGGTCCTGACGGGAAGGTCTTTTGTTTGGGCCGCTGAGAGATTCGACACTTGGTTTGA
TTCCCCTGGTTAACAACCGTTTTTTATCTGCATTAATTTCGGGTGTCTTTTATGAATAATCAAAAGCAGCAAAAGCCA
ACGCTAACCGGCCAGCGTTTCAAAACGAGGAAAAGAGATGAAAAGGAGAGATTTGACCCTACTCAGTTTCAAGAAAGT
ATCGTACAAGGCTTGAATCAAACTGGCACTGATTTGGAAGCGGTCGCGAAGTTCCTTGATGCCTCTGGCGCCAAGCTT
GACTACCGCCGGTATGCAGAGACACTCTTTGACATCCTGGTGGCCGGTGGGATGCTGGCCCCAGGCGGGACTCTATCT
GACGACATGACCCGCACCGAGTTCTGCCTCTTCACGGCACAAGAAGACCTGGAGACAATGCAAGCATATGCTCAGGTT
TTTAACAAGCTGATCAGGCGTTACAAGTACCTGGAGAAGGGTTTCGAGGAGGAGATCAAGAAGTTGCTGCTGTTTCTA
AAAGGGTTCACCGAGTCTGAGCGCAACAAGCTGGCCATGCTGACCGGTATCCTGCTGGCCAACGGCAACATATCGGCC
TCCATCCTGAGCAGCCTCTTCAACGAGAACCTCGTCAAAGAGGGAGTATCTGCAGCCTTCGCTGTCAAGCTGTTCAAG
TCATGGATCAATGAGAAGGACATCAACTCTGTCGCCGCCAGTCTCCGCAAAGTCGGCATGGACAACAGGCTGATGGAA
CTCTTTCCTGCCAACAAACGGAGCTGCGAGCATTTTTCAAGTACTTCACTGACGCCGGGCTGAAGGAGCTATCCGACT
TCGCCCGAAACCAGCAATCCATCGGTGCCCGCAAGGAGCTGCAGAAGGAGCTACAGGAGATGATGGCCCGCGGTGACC
CTCAGAAGGAGATTATCGCCTTCACCAAAGAGGAAATGAAAAAGGCCAGCCTCTCTGAGCAGGCCATGATCAGCATCA
TCTGGACCAGTGTGATGAGCTCCGTGGAGTGGAACAAAAAGGAAGAGCTGGTGACCGAACAAGCCATCAAACACTTGA
AGCAATACAGCCCTCTGCTGAAAGCCTTCACCTCCCAGGGTCTGTCTGAGCTCAGCCTGCTACTGAAGATTCAGGAGT
ACTGCTACGACAACATCCACTTCATGAAGGCCTTTCAGAAGATCGTGGTGCTCCTCTACAAAGCGGATGTATTGAGCG
AAGAGGCCATACTGAAGTGGTACACCGAAGCCCACCTTGCCAAGGGGAAGAGTGTTTTCCTGGAGCAGATGAAAAAAT
TTGTCGAGTGGCTGAAGAATGCAGAGGAAGAGTCCGAGTCGGACGAGGAGGAAGCAGACTAAAACCTCCATCGCCAGA

TGAGCTTTTCTTTTTTTCTTTTTTTTTTTTTTTTTTTTAACAAGCAGGAGCAGTAGAATGTGTTTTCCCTTCTTTCTC
TTCTACCTCCTGTTACTACTTTATCCCCAAACACACACACCTCCACTTAAACATTTAAACGTTAATTAAGCAAAATAA
AAAAGGGCTCAGTCCTTATTCGTAATGTTTGATGTTGCGGCTGACAGTGGAAGCGGAAAACTATGGATCCTCCTCTCT
TTATTTTTTGGGTATTATTGTTGTAATGCCTTCTGTAATTTGGATACAAAACATTCCATGTGATCTCTATAGCCGCTG
CTTGTATGAATCTGAACCAGAATTTGGTAAAAATTCAAGTTTGCCTTTTTGCCATGCGACTACGAGGCTGACAAAAAT
GTTAATGTAATTCAGCCAAAGTTTCTTATTCTTCTTTTCTCCCTCTCCTTTT

>Sequ19173SNP3
GGGGGGGTTAACAAGGCCAGTGGGTGGGAAGGGAGACATAGATGGAGGGGGCGAGAGGGAGAGGATGATGGTCGGGTG
GGGAGAACTGAAACAGAGAGCTGTTAGTGGGCAGAGGGAAGCCCAGGTAGAGTTATTAGACCTCTGTGTTTAACTCTC
AGAGACGGCCGGGATACGTCAGAGAAGACATGACCAACATCCTGAGGTGACAAATAAAGGTTTTACTTGGAGGAAAAT
CACAACCATCTCGTAGTCGTATTGAAATGTGGGACGCTGACTCTGCCTTTCTGCAGAAATGGGTAACTATATCAAGGG
AAGACATAGAGCTGGTTTGACTGGTTTGACTGGTATACGAATAGATTTATGATTACAGTGGAGAAATGTTTATCATGA
AAAGATTTATAACATTACAGTAGGTTTCTCGCTTAGATTTTTAGAGCCCCGGTGTTGAAATTTGATATATGTTGTTTG
AAGTTTTACTTAATTTGATGAGGAAATTATCTTCAGCTGCGGTTATTCATTTTCTGGAAAAGCTATAGACACATTCAG
TTCAGCTTTGCAGCGTTTGAGAATAAGCTCCTGTGGTCACAACAGAACTCGTGTGGAGCGTTGAATAACAGAGGTCAC
TGTGTAAACACTCCATGATACATTTTCCAGCACAATGGAGTCATTTTCCAGGTATAATGTTCGTGCCTACTTCTACCC
AGAGAACTTACAGCTCTGTTTGTGCCTACTCACACCAATCTATCATCTGTATTTGATCATGTGGCCATATCAATGAAT
TAAAGCATTTGCAACTGGTACACCTTCATGGATTCTGCTTTTTTTACTTTTTTGGTTAGTTGACATCT

>Sequ20292EST2
GCCCCGGTCAAAACCGGCCAGTGTTTACGTAACTTTGGCGTAGACTACATAGGCGACCATGACGTTAGCATGGGAGGC
CACACCTGTCTGCAGTGGGCGTCGCCGCAGGCCACGGCCCTCAGCCGGGACAAGGAGTTCATCCCAGAAGTCAGCCTG
CAGGGGAACAAGTGTCGTAACCCTGACAACGACCCCGAGGGCCCCTGGTGCTACGTGGAGGTACCTGGAAATGTGACC
GTGGACTACTGTGACCTGCATCTCTGTGAGGACCAGTTGCTTGCTGACTTGTTGACAACAGAGACTGGAGGGACGGAG
CGCTCCGTCCTGGGTCCCACCAGGAAAACCTTTTTCAATCCTCGTACCTTTGGACAAGGAGAGAGTGAGTGTGGACTG
CGCCCCCTGTTTGAAAAGAAGGGTAAAAAGGACACGAAGGAGGAAGAGCTGTTGGAGTCGTACAGAGACAAACGCATC
GTTGGGGGTGACGACGCCGATGTGGCCTCAGCACCATGGCAGGTGATGCTGTACAAGCGCAGCCCCCAGGAGCTGCTG
TGTGGAGCCAGTCTGATCAGTGATCAATGGATCCTCACCGCCGCTCACTGCATCCTCTACCCGCCCTGGAACAAGAAC
TTCACCAGCAAGGACATACTGGTCCGCCTGGGGAAACACAACAGAGCCAAGTTTGAGCGCGGCACCGAGAAGATCGTG
GCAATTGATGACATCATCGTCCATCCCAAGTACAACTGGAAGGAAAACCTGAACCGTGACATCGCTTTGCTGCACTTG
AGACGGCCAGTCACATTCACAGATGAGATCTTCCCCGTGTGCCTGCCCAGCAGGAAGGTCGCCCAGACCCTGATGACA
GAGGGCTATAAGGGCCGAGTGACCGGTTGGGGGAACCTGAAGGAAACCTGGAACCCATCAGCAAGAAATTTACCAACG
GTCCTCCAGCAAATCCACCTGCCAATCCAGGACCAGGACACCTGCCGCAGCTCCACATCAGTCAGGATCACAGACAAC
ATGTTCTGTGCTGGTTATAGACCGGAGGACGCCAAGCATGGTGACGCCTGTGAGGGAGACAGTGGTGGACCATTCGTG
ATGAAGTACCCGGCAGAAAACCGCTGGTATCAGATGGGCATCGTGTCGTGGGGCGAGGGCTGCGACCGGGACGGAAAG
TACGGCTTCTACACTCACCTGTTCAGGATGAGCAGGTGGATGAGGAAAGTTATTGAAAAGGCAGGAAACGACGACTAA
ACACAAGCATTCTTCACTTCCTGTTGTTGATGCTAATAAAGTCTGTTCCTTAACCACCAAAAAAAAAAAAAAAAACCGA
CCTACCCCTA

>Sequ20303EST2
GCGCTAACTGTGCAACTTCATCAGGTACTGGGGCGCGTCTGCGGAGGGAAGCGGGTTTCAATTTCAAACCCAAACAGT
CCGAGGGACGAGCCAGTGCATTGTGGGATAACGTGTAAACCAGATAGTTACAGCATTTAAGTTGAATTTGTAACGTTA
TTTAAACACACACCAGAACGGTGTCGCTACTACCGACCGTGGTTACAGTCCACAGGATCCGCGAAAAAAAGTCGCCTG
TCGCGTCTTCGCTCCAGTTTTCCGCGCCTGGTTTATTTGGATGACAAACCGACGCCTGGCTTGTTTGTTGCAGGTGCT
GACCAATCCGGATTTTGTTTCACATTAAAACTACCACTGTGTTTTAATTACACATATCTGACATAGCAAGCCATTTCT
TCAAGAAACATGCCGAGGTCCAACAGGCAAAAGGAATACAAACCTGGAGATCTTGTGTTTGCTAAAATGAAGGGGTAC
CCACACTGGCCTGCGAGGATTGATGAATTACCTGAAGGAGCAGTGAAGTCGCCCTCAAACAAATACCAGGTGTTCTTT
TTTGGAACACATGAGACGGCATTCCTGGGGGCAAAGGATTTGTTCCCGTATGATGAATGTAAGGAGAAGTTTGGAAAA
GCAAACAAAAGGAAGGGCTTTGCTGAGGGACTCTGGGAGATCGAAAACAACCCCACCGTCACACATGAAGGCTACGAG
TCATCAAAGAAAGACAATGCATCAGAAGGAGCCGGGGATACAGGTAGTTCGGAGAAAGCAGATGCTGAGGGCAGTAGT
GATGAGGATGAAGGGGCCCTGGTCATCGACGAGAAGAACGAGAGGGGAGGAACCAAACGAAAAGCAGAGGAGTCCACA
GAGGCATCTCCCAAGCGGCCGAAGGATACAGGAGTGGAAGGGGACTCTAAAGTAGAGAGCAACAAGTCTAACACAGAG
GCCAAGCTCAACGATGTGGCTGGACCCAAGGCAACTGCTCCCTCCTCGCAGAGTGAGTCAAAGCCAGAGGCCCAGGAA
AATGCTCCAGCAGGAGGCCAATTAACAGCAGATAAGCCTGTGACAGATAGTGCTTAACATCAACTCAGAAGAAAACCA
AGAACCTTTGACGCTTCTCTCTGGAATTTAGGCATCAACATGGTTACCTGCAGGATGCCAGATTTCAGCTGTTTCATT
AATAGGAAGAAAACTAAAAAAAAAAAAAACCGACTACCCT

>Sequ20309EST2
TTCCCTGCCTATGCCCTCGTGAGGTGGGCAGTGTAAAAATTTCCTGTCTCTTTTCTCCACCAAGCGAATCCAAAACCA
TCCGTCAAAATGGTGAACTTTACCGTAGACCAGATCCGTGCCATCATGGACAAAAAGGCCAACATCCGTAACATGTCT
GTGATTGCGCACGTCGACCATGGAAAGTCAACTCTGACAGACTCGCTGGTGTCGAAGGCTGGCATCATTGCCTCAGCT
CGTGCTGGAGAGACCCGATTCACAGACACGCGCAAAGATGAACAGGAACGCTGCATTACCATCAAGTCCACTGCCATC
TCCTTGTACTATGAGCTGGCTGAAAATGACTTGGCCTTCATTAAGCAGACCAAGGATGGAGCCGGCTTCTTGATCAAC
CTGATTGACTCACCAGGGCACGTTGACTTCTCCTCTGAAGTGACTGCTGCTCTCCGTGTGACTGATGGAGCCCTGGTT
GTAGTGGACTGCGTGTCTGGTGTTTGTGTGCAAACTGAGACAGTGCTCCGTCAGGCCATTTGCGAGCGTATCAAGCCA
GTCCTGATGATGAACAAGATGGACCGTGCCTTGTTGGAGTTGCAGCTTGAACCTGAAGACCTTTACCAGACTTTCCAG
CGCATTGTTGAGACGGTCAATGTCATCATCTCCACTTATGGAGAAGATGAGCATGGACCTATGGGCAACATCATGGTT
GATCCAGTCATTGGTACTGTCGGCTTTGGCTCTGGACTCCATGGCTGGGCTTTCACCCTGAAGCAGTTTGCTGAGATG
TATGCTGCCAAGTTCGCAGCTAAGGGTAACACCCAGATGACTCCAGCTGAGCGCTGCAAGAAGGTGGAAGATATGATG

AGGAAACTGTGGGGTGACAGGTACTTTGATGCGGAAACTGGAAAGTTCAGCAAGAGTGCTAATGGACCCGATGGCAAA
AAGTACCCCCGTACCTTTGTTGCTCTTATCCTGGACCCCATCTTCAAGGTGTTTGATGCCATCATGAACTTCAAGAAG
GAGGAAACTGCCAAACTGATCCAGAAACTGGAAATCAAGCTGGATACTGAGGACAAGGACAAGGAGGGTAAGCCTCTC
CTGAAGGCTGTCATGCGTCGCTGGCTGCCCGCTGGTGAAGCCCTTCTGCAAATGATCACCATCCACCTGCCTTCCCCC
GTCACTGCCCAGAAGTACCGCTGCGAGCTGCTCTATGAAGGACCTGGAGATGATGAGGCTGCCATGGGTATCAAGAAC
TGTGACTCCAAGGCTCCCTTGATGATGTACATCTCAAAGATGGTACCTACCAGTGACAAGGGTCGCTTCTACGCCTTT
GGCCGTGTGTTCTCTGGATCTGTCTCCACCGGACTGAAAGTACGCATCATGGGACCAAACTTTGTCCCTGGAAAGAAG
GACGACCTCTACCTGAAGCCAATTCAGAGGACATTTTGATGATGGGCCGTTACGTTGAGCCCATTGAAGATGTGCCAT
GCGGTAACATCGTGGGTCTGGTTGGTGTGGACCAGTACCTTGTCAAGACCGGAACAATCACCACCTTTGAGCAGGCAC
ACAACATGAAAGTCATGAAGTTCAGTGTCAGCCCTGTCGTGAGAGTTGCTGTTGAGGCCAAAAACCCAGCTGACCTGC
CCAAGCTGGTGGAGGGATTGAAGCGTCTGTCCAAGTCCGATCCTATGGTGCAGTGTATCATTGAGGAGTCTGGAGAAC
ATATCATTGCAGGAGCTGGAGAGCTGCATCTGGAGATCTGTCTGAAGGATCTGGAGGAGGACCATGCTTGCATTCCAC
TCAAGAAATCTGAACCAGTGGTGTCCTACAGAGAGACGGTCAGTGAAGAATCAAATACCATGTGTCTGTCAAAGTCAC
CCAACAAGCACAACCGTCTGTTCATGAGGGCCCGTCCCTTCGAAGATGGCCTGGCAGAGGACATTGAGAAGGGTGATG
TTAGCGCTCGTCAGGAGCTCAAGGCCCGTGCCCGTTACCTTGCTGACAAGTATGAGTGGGATGTCACTGAGGCCAGAA
AGATCTGGTGCTTTGGCCCTGATGGAAATGGCCCCAACATGCTGGTGGACGTTACCAAGGGAGTGCAGTACCTTAATG
AGATCAAGGATAGCGTTGTGGCAGGCTTCCAGTGGGCAGTCAAGGAGGGTGTCCTCTGTGAAGAGAACATGCGTGCCA
TTCGCTTCGACATCCATGATGTGACCCTGCACACAGATGCTATTCACCGTGGTGGTGGTCAGATTATCCCCACTGCCC
GCAGAGCTCTGTACGCCTGTGAGCTGACAGCTGAGCCCAGACTCATGGAGCCTGTCTATCTGGTGGAGATCCAGTGCC
CTGAAGTTGCAATGGGTGGAATCTATGGTGTGTTGACCAAGAGGCGTGGTCACGTGTTTGAGGAGTCCAGTGTGATGG
GAACACCCATGCGTGTCATCAAGGCCTACCTGCCTGTCATGGAGTCATTTGGTTTCACAGCTGACCTTCGCTCCAACA
CTGGTGGCCAGGCCTTCCCACAGTGTGTGTTTGACCACTGGCAGATCCTTCCAGGAAACCCAATGGACACCACAAGCA
AGCCTGGTGTAGTTGTCATGGAGACACGCAAACGTAAGGGTCTCAAGGAAGGTATCCCAGCCTTGGACAACTACCTGG
ACAAATTGTAAAAGCCTGAACCCATCTGGCTCATGCCTCATTCCTCGTTTCCCATCAAATTTAAGTCTACATCATGGG
ACCGGACTGTACAGAACATAGGGAGCACTAATGTTTGCCAAAAGCATTGACAATAATGAATAAACATCTACAAAAATT
GTAAACGATAATAAA

>Sequ20310EST2
TCGAGAAAGAAGCTGCAGACGGCGGATATGACGTCGACATCAGCAACGTGACGGAGGACATAGGCGTGCTCGGCATCG
CGGGACCCAACTCGCGCAAAGTTCTTCAGAAGCTGACGGAGGAGGATATGAGCGACGCCGGTTTCAAGTTTCTCCACT
GCAAGTCCATCAAGCTGGCCGGCATCCCCGTCCGCGCCATCAGGATCTCCTACACCGGCGAGCTCGGCTGGGAGCTGT
ACCTCGACCAGAAGAACATGGCGGCCGTGTACCAGGCCATGATGGAGGCAGGAAAAGACGAAGGCATCGACAACTTCG
GCACCTACGCCATGTCCTCCCTCAGACTGGAGAAAGGCTTTAGAGGCTGGGGAGCTGAGATGAACTGTGACACGAATC
CTCTGGAGGCTGGTTTGGATTATTTCATCAAACTGAACAAGCCGGCTGACTTCATTGGCAAAGCCGCCCTTCAGCAGA
TCAAAGCCAAAGGCCTGAAGAGGAAGCTGTCCTACATCACGCTGGATACGGACGATATCGACCCCGAGGGCAACGAGA
CCGTCTGGCACAACGGCAAGGTGGTCGGCAACACGACGTCCGGAGCCTACAGCTACAGCAGCCAGCAGAGCCTGGCGT
TCGCCTACCTGCCCATGGAGCTGTGCTCCGTGGGCCAGAAGGTGGAGGTGGAGCTGCTGGGGAGGAAATACGCCGCCA
TGGTCATCCAGGAGCCTTTAGTCCTCACCGAGCCAACGCGGACCCGGCTGCAGAAGAAAGCAAAGGGCAAAGCATAAG
CACGGACTTCTCCTCCATCAGTTTATCAGCAGCTAATGAAGAACAATCCCACGGAATTATGAATGAGAGGTAAAGCTG
TTATGGAAAGAAATCATGTGCAGGCCATGTGCCGAGGTGTTTGTGTCTGCGGCCACTTTAGGAAAAAAGAGGCTCGTT
CACATCAGTCACAACTTCCTCTTCTCGTCCTGAAAACAGACCCGGCATGTGAGAACGCTCAGTGTCTTACGACTGTGT
TTTTGAGAAATAATAACCGTCATGTGGTGTATTTGCACCACAGTGTCGTAAAGTGTTGAAAGAGTAATTTACATTTTT
TGTGTAATTCCATGAATGGATGTTAACGGTTTATGGGTCAATACAATACTTGAACATTTTCAAACAAAAGGGTCAAAT
GAAAAATACAGCTTTTTGTGTTTATTTGAGAGTCAAGAGATGATGTCGCTGCATTTTACACAGTCCTCATCAGCTGTT
ATAAACACCAACAACCGAAAGAAACAGAATGTTTGCAAAACTTGTACAAAGATTGTTTTTTAAAATCTGTTTTTTTTT
TTTACTAATATGTAATTAAATTAGGTGTACATTGTATACCTTGTATAATTAAAGCAATAGC

>Sequ20323EST2
TGCCATCGCTCTGACCTGTGGCAACGTCTGCCTCTGGAAAGGAGCTCCAACCACACCTCTCACGAGTGTTGCAGTTAC
CAAGATTGTGGCTGAAGTGCTGGAGCAGAACAACCTGCCCGGCGCAATCTGCGCCATGACCTGCGGAGGCGCTGATAT
CGGCACCGCCATGGCGAAGGATGAGCGCGTGGATCTGCTGTCGTTCACTGGGCAGCACCCATGTTGGCAAGATGGTGG
CCATGATGGTGCAGGAAAGGTTCGGTCGCAAACTGCTGGAGCTCGGCGGAAACAATGCTATCATTGTGTTTGAGGACG
CTGACCTAAATCTTGTGGTGCCCTCTGCTGTCTTCGCATCCGTGGGAACCGCTGGCCAGCGCTGCACCACAACCAGGA
GGCTGATGCTGCATGAGAGCGTTCACGACACAGTGGTCGAGAGGATCACCAAAGCCTACAAACAAGTCCGCATCGGAG
ACCCCTGGGATCCCAGCACCCTGTATGGGCCTCTGCACACCAAACAAGCGGTGGATCAGTACCTGGCAGCTATTGAGC
AGGCCAAGAAACAGGGCGGCACTCTGGTCTGTGGAGGAAAGGTGATGGACCGTCCTGGAAACTACGTGGAGCCCACCA
TCATCACAGGGCTGGCTCACGACGCTCCCATCGTCCATACAGAAACCTTCGTCCCCATCCTCTATGTCCTCAAGTTCA
AAACAGAAGAGGACGCATTTGCGTGGAACAACGAGGTCAAGCAGGGTCTGTCCAGCAGCATCTTCACCAAAGATATGG
GCCGGGTTTTCCGCTGGCTGGGACCCAAAGGATCCGACTGCGGCATCGTGAATGTCAACATTCCTACCAGCGGAGCTG
AGATCAGAGGAGCCTTTGGTGGGGAGAAACACACTGGAGGTGGAAGAGAGTCTGGCAGTGACTCGTGGAAGCAGTATA
TGAGGCGTTCAACGTGCACAATAAACTACAGCAAGGATCTTCCTCTTGCCCAGGGAATCAAGTTCGAGTGAAGCCTTG
AAGTAGTTTAATTAAGCTTCGATGAGCCCAAAAGGACAACACTACATGTTTTCAAGTGTTGCTTAATAATGCAAGACA
TTTGTTTTGCATTTTTGAATTCAGAAAGGTCAGTTTAATTTGAACAGAATTTGAAATACTTTTGGATGGGTCTCATTA
GTCATGCATCAAATCTATGTATGTGGTTGTGGCTTTAGATACTAATCCTGGATTTGAAGTTTGCAAATAGTTTTATGT
TTAAGGTCAAATACTGTTTCCTCAAAGGTTGGGGTGCACTTTTCTACAAGCACTTAGAAACTGGGTTTGAGAGCTAGA
TGACTGTAGCAGATACTTTATACGGTATAAAAAATAAAAAAAGTGATGTTGACATTATCAACATGTCAAATTAGAATA
TGACGGTCAATAAAGGTGCAGATTTGCATTGTTTTGATGGTTGATTTTATATAAACAGAAAGACACATGAGAATGCGA
GATACAGTATTTCTGACATGGTAAATAGCATTTCATCACAGACTGCTTGAAAAGGAGTTAAGTGTTAAAACACTGTTG
TTTGCATCTCTACAGCATTAATATACTGTGCTTTGCATCTAAAGCGCCCTTTAAGAATGGTCACAATGTGCTTTTCA
GACTAACCACAAATAAAAGCTTCTTGTTGATACGTACTAAAAAAAAAACGAAAACCTCCCTACTA

>Sequ20339EST2
GAGGATGCTAAGAAAGAGCGCGGCGTATCCTCGAGGCCTGCGGAGGCCACAGTATAGGGGCCTACAGTGCCAGCCAGG
GGATCGAGTGCGTCCGTCAGGATGTGGCGTGCTACATAGAGAGGAGAGATGGTGGAATCCCCTCCAGCCCTGACAACA
TCTACCTCTCCACTGGGGCCAGTGATGCCATTGTGACCATGCTGAAGCTGCTGATGTGCGGTGAAGGTCGAGACCGTA
CAGGAGTCATGATCTCCATACCTCAGTACCCCCTGTACTCGGCCGCATTGACCGAACTGGGTGCAGTGCAGATCAACT
ACTACCTGGACGAGGACAAGTGTTGGAGTCTGGACGTCACAGAGCTCAGGAGAGCCCTCAATGAAGCCAGGCAGCACT
GCAACCCTCGAGCCCTCTGCATCATCAACCCTGGAAACCCCACCGGTCAGGTCCAGAGCAGACAGTGCATTGAAGATG
TAATCCGATTTGCGAAAAAGGAGCACCTCTTCCTCATGGCTGATGAAGTCTACCAGGATAATGTGTATGCAGAGGGCA
GCAAATTCCACTCCTTCAAGAAGGTGCTGTTTGAGATGGGACCAGAGTACTCCAGCACAGTGGAGATGGCCTCCTTCC
ACTCCCACCTCCAAATGCTACATGGGAGAGTGTGGATTCCGTGGAGGTTACATGGAAGTGATTAACATGGACCCTGAG
GTGAAGGTCCAGCTCACCAAACTGGTGTCAGTGCGTCTGTGTCCCCCAGTTCCCGGACAGGCTCTCCTGGACCTGGTG
GTCAACCCCCCGCAGCCAGACGAGCCCTCCTACACCACATTTATGAAGGAGCGGACAGCGGTGTTAACAGCTTTGGCA
GAGAAGGCCAGGCTGACGGAGGAGATCTTCAACACAGTACCTGGTATCACTTGTAACCCAGTCCAGGGGGCCATGTAC
ACCTTCCCCCGCATCACTCTACCTCAAAAGGCCATTGACAAGGCCAAGGAAGAAGGCCAGGTCCCAGACATGTTCTAC
TGTATGAAGCTGCTGGAGGAGGAGGGGATCTGTCTGGTGCCAGGTAGTGGCTTTGGGCAGAGAGAGGGAACCTTCCAC
TTCAGGATGACCATCTTGCCACCCGCTGAGAAGCTAAAGTTTGTGCTGCAGAAGATCCGTGATTTCCACCTGCGGTTC
ACACAACAGTTTTCCTAAACAATCCTCCACCCTGAACTACATCTCAAGAACCAACCCATTCAGTGCCAGTATCTTCAA
GTGCCTTAATGTGTGAATGAGGCTGCAACTTCTGCAATACCCATATCTCACCTCAAAGCTGTGGTCACACCAGAGCAG
ATTTTTGTAAAGATATTTTCTGTGTTTTGCAGCAGTGATGAAAGTTTCCTTTGGATAACTAGTGAAAACATGTGCACA
GAATGATGCACTGTTCATATGTACATACAGCATGTGAAGACGTAATCATCTTCACTGCATGATGCACTTTCCCAAAGT
ATAACCCGGCCAAGTTTGTGATTTTAGATGTTATTATCTATAGAGTTATGAGGTATTTTAGATGTCCTTGTCACAGAA
ACTGCTACAGGTGCATCTCGCTCCTGCCAAAACTTTTTAACAGCAAAATTAGTTCAAAATGATGAACATGTTAGGTGA
TTACTGGATGAACATGACTGGCTGTAGAAGATGCACCATAATGAGGATGACTGAGTACTGCCAGGGCAATTCTTGCCT
GTCTCAGAATTTCTCTCCGTTTGTATTACTAATGTTTAAATTGTTCATAAATGTGATTTAAAAAACACTTGA

>Sequ20356EST2
GACAGATCACACCGAGAGGAGAGGGCGCATCCACATCTCTGCCCAGATCACTGAGGACACTCTCACCGTCATCATCAA
AGAGGCTAAGAACCTGGTGCCCATGGACCCCAATGGCCTGTCAGACCCCTACGTCAAGCTCAAACTGATCCCAGATCC
CAAGAGTGAGAGCAAACAGAAGACCAAGACCATCAAATGTTGCCTCAACCCCACCTGGAATGAGACCTTCAATTTCCG
CCTAAAAGAAAGTGACAAGGACCGCCGTCTCTCGGTGGAGGTCTGGGACTGGGATTTGACCAGCAGGAACGACTTCAT
GGGATCCCTGTCCTTTGGGATCTCAGAGCTACAGAAACTAGGGATAGACGGATGGTTTAAGCTGCTTTCTCAGGAGGA
AGGAGAATACTTTAATGTTCCTGTCCAGCCAGATGGAGAGGACGGTAACGAGGAGCTACGACAAAAGTTTGAGAGGGC
GAGGGTGGGTCCAGGGAAGCCCACGGACTCTTCTTCCTCCTCCTCGGTGTGCAAGTATGACAGCAATGGCAATCAGGA
CCGTGTCAAGCTATCGGACTTCAACTTCGTCATGGTTCTGGGCAAAGGCAGCTTTGGCAAGGTGATGTTGGCTGAGAG
GAAGGGGACGGACGAGTTGTACGCCATCAAAATCCTGAAGAAGGACGTGGTGATCCAGGACGATGACGTGGAGTGCAC
CATGGTGGAGAAGAGAGTCCTCGCTCTGTCTGGGAAACCACCTTTCCTCACTCAGCTGCACTCCTGTTTCCAAACCAT
GGACCGGTTGTATTTTGTGATGGAGTATATAAATGGAGGAGACCTTATGTACCACAACCAAAGGTTAGAAAGTGCAAG
GAGCCTGCATGCTAGGTTTTATGCTGCAGAGATTGCCATCGGACTCTTCTTCCTCCACTCCAAAGGCATTGTTTATCG
GGATCTGAAACTGGACAATGTGATGCTGGACTCTGAAGGCCACATAAAGATCGCCGACTTTGGAATGTGCAAAGAGAA
CATGTATGACGGCATCACCACAAAGACCTTCTGTGGAACACCAGACTACATCGCTCCAGAAATCATAGCCTATCAGCC
TTATGGGAAGTCTGTGGACTGGTGGGCCTTTGGAGTACTGCTGTACGAAATGCTAGCTGGACAGCCGCCTTTTGATGG
GGAGGATGAAGACGAGCTGTTCCAGTCGATCATGGAGCATCACGTCTCTTACCCTAAATCCATGTCCAAAGAAGCTGT
TGCCATCTGCAAGGGGCTGATGACCAAACATCCATGTAAGCGTCTGGGCTGTGGTCCAGAGGGGGAGAGAGACATCAA
AGAGCACGCCTTCTTCCGCTATATAGACTGGGAAAACTGGAGAATAAAGAGGTGCAGCCACCTTTCAAACCTAAAGCT
TGTGGGCGTGATGCGGAGAACTTTGATCGCTTTTTCACGCGCCACCCCCCAGAGCTGACCCCCCCAGATCAGGAGCTT
ATAGCCAACCTGGACCAGGAAGAATTCCAAGGCTTCTCATTTGTTAACCCTGAGTACACTCACTCAATCCACTGACCA
TGCCACATATCACTGGGCTGCACTAG

>Sequ20385EST2
GGTACTGCTCCCTTTTCTTTTCCGCTCAGGTCTCATCAGTGGTGGGAGCGTGGATTTCATCATCTAGGCTCACAAAAT
CACAAAAAAATGAGCGTCCCAGCATTTATCGACATTACGGAAGAAGACCAGGCCTCAGAGCTGAGAGCCTACATCAAG
TCCAAAGGAGCTGATATCTCAGAGGAGAACTCTGAAGGTGGACTTCATGTAGATCTGGCTCAGATCATTGAGGCATGT
GATGTCTGTCTCAAGGACGATGATAAAGATGTAGAGAGCGTGATGAACAGCATCGTGTCACTGCTGTTGATCCTGGAG
ACAGAGAAGCAGGAGGCTCTCATTGAAAGTCTTTGTGAGAAACTGGTGAAGTTCCGTGAAGGAGAGAGACCCTCCCTC
CGGATGCAGCTACTCAGCAACCTGTTCCATGGGATGGATGAGAACACTCCAGTGAGGTATACAGTCTTCTGCAGCCTC
ATCAAGGTGGCAGCGACTTGTAATGCCATCGCCTTCATCCCCACTGACCTTGATCAGGTGCGCAAGTGGATTGTTGAC
TGGAACCTGAACACAGAGAAGAAGCACACACTGTTGAGGCTGGTGTATGAAGCATTGGTTGACTGCAAAAAAAGTGAG
CCTGCAGCAAAAGTGATGGTTGAGCTGCTGGGAAGTTACACAGAAGACAATGCTTCACAAGCACGGGTTGATGCCCAC
AGGTGTATTGTCCGTGCTCTAAAAGATCCCAACACTTTCCTGTTTGACCACCTGCTCACCCTGAAACCTGTTCGCTTC
CTGGAGGGAGAACTCATCCATGACCTGTTAACCATCTTCGTGAGCGCAAAACTAGCAGCATACGTAAAATTTTACCAG
AGTAACAAAGACTTCATTGATTCTCTCGGCCTCTCTCACGAGCAAAACATGGCCAAGATGCGTTTGCTGACATTCATG
GGCATGGCGGTGGAATTCAAGGAGATCTCCTTTGACACCATGCAACAGGAGCTGCAGATCGGAGCCGATGACGTTGAG
GCTTTTGTCATTGACGCTGTTCGGACCAAGATGGTCTACTGCAAAATCGACCAGACACAGCGAAAAGTTGTTGTGAGC
CACAGCACACACCGCACCTTTGGCAAGCAGCAGTGGCAGCAGCTGTATGACAGCCTCAGCTCCTGGAAGGCCAATCTA
GCAACCGTCAAGACCAGTCTGCAAGCTCTATCACCTTCTGCTTAATTCCACCTTAACCCAACTGTCTAGACTGCCCAA
CCTTTCCAGATTCCCAGACTGAAATCATCAGTTTTTATATTCATTCAATAAAAGAACAGTGGAGATCCCAAAAAAAAA
AAAAAAACTACGAGACTAGCAACGGACGACACAGGGA

>Sequ20386EST2

CTGCTGCCACCGTACGCCACTATCCGCCGCCCGGAGGCTGGAGCTGGAGAATCACCCTCAGTGTGCTTGTGAGCGCGG
TGCTGTCGTCGTACTTCCTGACGGAGCGGCTGAACCTGCATGGGAAGCTCGGCTGCATGCTCAGCATCCTGGGGCTCC
ACCACAATGGTGATTCACGCACCGCAAGAGGAAGAGATCAGCAGCCTCGACCACATGGCCAGGAAGCTGGTCGACCCA
GGGTTTTTCATCTTTGCCACTCTCGTCATCATCGTGGCCATCATCTTCATATTTGTTGTGGGTCCCCGTCACGGTCAG
ACCAACATCCTCGTGTACATCACCATCTGCTCGGTAATCGGGGCGCTGTCGGTGTCCTGCGTCAAAGGACTTGGCATC
GCCATAAAGGAAGCGATCTCTGGGAAGAATGTTGTGAGGAACCCGCTGGCATGGTTCCTGCTTCTGGGTCTGGTGGCC
TGTGTGAGCACACAGATCAACTACTTGAACAAGGCTCTGGACATATTCAACACCTCCCTGGTGACTCCCATCTATTAC
GTGTTCTTCACCACGTCTGTGCTCACCTGCTCCGCCATCCTCTTCAAGGAGTGGGAGCACATGGGCACCGACGACGTG
ATCGGCACCCTCAGTGGCTTCCTCACAATCATCGTGGGCATCTTCCTGCTCCACGCCTTCAAAGACATCAGCGTGAGC
TTGGCTACTCTCGCCGTGTCCATGAGGAAGGAAGAACGGGCGTTCCCTGCAGCCAATGGCATGACTTCCCACAGCACG
TACGAACTGCTGCACAACGAGTCCACTGGGGACGTGGAGGACAGAGAAATGGGTTTGCCTTTTGACAGCATCTCCAGA
AGGAACGGGGCAATGACTTCCTCATTGGATCATTAAGGAAGTTTTTTTCTTTTTTTCCCCAGCCCCCGACTGACTGAC
CAATCGTGGCATTGTTGTGTAAGAACAAGACAATCAGCTGTATGGGGAGAAGTGACTTGGACAGACCAGTGGATTGTA
TTTAATGCTTTAATTTGCCTTACTTTTACTCAGAATATGACCTGCTCATATTCAGTTACAGTTAAAACTTAATTTCAT
TCCTCTGTAGCGCTGTAAATATGAGTTTTTAAAAGAAATGTTTTGTTATATGTACATGCATATTCATGTAATGAATT
ACTTTCTATAAATCAGAAGGTTTTAATATATCTTCTATATGAGGTCCTGCTGACGATGACTGCGGTTAAGAGGACGTT
CACTATGATTTGAGTTTTCGTGATACTTGAAGGTAACAACAGTTTTTGTTGGGAGTCATTCTGAACTTTTTTTTGTAG
TGTTTACATGAAAAGCTCTCGAGACGAACCAACAGATTTGTTACATATTTATTGAATTAAAATGATTAAAACATTTGA
AACACAAGGCACAATGAATAAATGCTTAACATGAGTATGAGTTACTTTTCAATTTCCTTTTTAAACTGTTTAGCATGC
ATGTTCAACAGTATAGAAAATACAAATTTACTTTCACCCCCACTTAAAATAGGTAACTGCTCACCCTTAATAATCCAC
CACTGCAAACCCTTAAATATTTACCTGATAAAACTAATTTCCCTCTATAGCTTTCTGCAAGTATAAAACAAAGGATAG
TTCATCCATAAAAGGGGAAATATCTATCATTTCTGATAAGGCACAAGTGAATACAAAAAAAAAAGTGGCCCAACTTCC
TTACATTTAACGGTCCTTCGCTACTGAAGACCTAAAGTAAATAAGGAGGAATATTCTAGCTGGTCTGGTAATGGTGGC
AGTGTTTGGTACTTCATGCTGGTGCAGGGTTTCAGCTGCTGGCCAGCGACTGGTGTATCGGTGGTTGGAAGCATCGGA
CGTGTTCCACCGGCATGTTCTCTCCGTCTCCGGACTTCATGTACTTGTTCAGGATGGCAAAGATCTCGTTGTTGAGGA
CTTGGAACTTGCGGATTCTGTCAACCATTTTCTTCAGTGGCACACTCTTGATGACCTCGTCTTTGCCGTCGTGTTTCT
GCACCTTCAGGAGGTGGTAGCTGAAGTCGAGGATGTCGAAGCGTCTCTGCTGTCCCAGCAGGGCGATGATCATGCAGC
CCGCCCAGTGGAGGCCGTCTCCGAAGCACTGCTCTACTGTGAACTCGTGGGCTCCCACAGGGATGCAGTACACAAACT
GCATGGCACTCCAGAGACGGTGGAACTCCACACACTCGTCCACGTGCATCACACCGTTGCTGGGCAGCGGCCCGCGCC
AGATGGGGTCATCTAGGAACCCTCGCACGCGCGTGAGGATGACTTCGAACATGGACAAACCACAGCACAGCCTCTCTT
TGGTCAGAAGGTCGCCCTCGCGAGCGATGGCTATTTGCTGTGGGGTGCCGAGTCGCTCTATCAGTGGAACCATATGCA
GTGCTGTGTATTTGGCTTCTAAGCGCTTCATCTTGGCGTCCAGACGTTCCCCCTCTTTGACATGGACTCTGGGAAGAA
TGTTTTGGAAAGGTGCAG

>Sequ20393EST2
GAGAGTTCTGGATGTCCTTCAGTGACTTCCTGCGTCACTACTCCCGGATCGAGGTGTGCACTCTGACGCCCGACACCA
TCGGTGACGACTCCGTCAAACACTGGAGCGTCAGCAAGTTTGACGGCACCTGGAGGAAGGGCTCGACGGCTGGGGGCT
GCAGGAACCACCCCTACACGTTCTGGATGAACCCTCAGTTCGTGATCCGGCTGGAGGAGGAGGACGACGACCCCGATG
ACGGCGAGGTGGGCTGCAGCTTCGTTGTCGGTCTGATCCAGAAGAACCGCAGGAAGCTGCGAAAACAAGGCGAGGACA
TGCACACCATTGGGTTCGCCATCTACGAGGTTCCACAACAGTTCCACGGGCAGCGGGAAGTGCACCTGGACAAGAACT
ACTTCCTGACACACGCTCAGACGGCGAAGTCAGAAACCTTCATCAACCTGCGCGAGGTCAGTTCCCGCTTCAAGCTCC
CTCCTGGAGAATACCTGATCGTCCCCTCCACCTTCGAGCCGCACATGAACGGGGACTTCTGTATCCGGGTGTTCTCCG
AGAAGCAGACAGAGACCCAACCCTGTGATGACCCGGTCCACGCTGACCTGGAGGACGAGACGGTGTCTGACGAGGAGG
TGGACGCAGGGTTCCGAGGCCTCTTCACCAAACTGGCCGGAGACGACATGGAGATCTCTGCGGCGGAACTCAGGACCA
TCATGAACAAGATCGTGGCCAAACGAACTGACATCAAGACGGACGGCTTCAGCATGGACACCTGCAGGATCATGGTCA
ACCTGATGGACGACAGCGGGAACGGGAAGCTGGGCCTCGGAGAGTTCGCCACGCTGTGGAAGAAGGTTCAGAGATACC
TGTCCATCTACAAGAAGAATGACTCTGATGACTCGGGCACCATGAGCACGCCAGAGATGAGAGTGGCCTTCAAAGACG
CAGGTTTCACCCTCAACAACACCATCTACCAGCAGCTGGTGACTCGATACTCCGACCCCGACATGACCGTCGACTTCG
ACAACTTCGTGGGTTGTCTGATGAGACTGGAGATGATGTTCAAGATCTTTAAGAAGCTCGACGCTCACGAAAGCGGCT
CCATTGAGCTCAACTTCAATCAGTGGTTAAACTTCGCCATGATCTGAGCCGTGACTCCTTGTTCGCCTGGAGCCTCAG
TGTTAAAGCAGCAAGCCATATTCCTGTTTACAGCCGCCGCTTCTTTTAGACTCCTAATGTTTTATATGTGAGGCTTGT
GTTTTATTATCTATACACTACCTGACTTACAGTCGATGTAAAGACAATAAATTTATAACCAAAAAAAAAAAAAAAAAA
AACTAAACGTAGAACG

>Sequ20398EST2
CCTCTCTGCCACTATCCGTCGGCCAGGCTGCTGCAGCGCTCAACCTGCCCGGAATACAACACCAGAAATCAGCCATGT
CTGACAAACTCCCCTTCAAAGTTGCTGACATCACCCTGGCCGAGTGGGGGCGTAAGGCCATTGATATCGCAGAGAATG
AGATGCCCGGTCTGATGAAGATGAGGGAGATGTACAGTCAGTCCAAGCCTCTGAAGGGCGCCCGCATCGCCGGCTGCC
TCCACATGACCCTGCAGACCGCCGTGCTCATCGAGACCCTCATCGCCCTCGGAGCTGAGGTTCAGTGGTCCAGCTGTA
ACATCTTCTCCACTCAGGATCACGCTGCTGCCGCCATCGCCAAGGCTGGTATTCCAGTGTACGCGTGGAAAGGAGAGA
CAGATGAGGAGTACGTGTGGTGCATCGAACAGACTGTGTACTTCAAAGACGGTCAGCCCCTCAACATGATCCTGGACG
ACGGAGGAGACCTCACCAACCTGGTCCACAAGAAGTACCCCAAACTGCTGGCAGGTATCCGTGGAGTGTCTGAGGAAA
CGACCACAGGTGTCCACAACCTGTACAAGATGATGAAGAAGGGCGATCTGAAGATCCCCGCCATCAATGTCAACGACT
CTGTCACCAAGAGTAAGTTTGACAACCTGTACGGCTGCAGGGAGAGTCTGATCGACGGCATCAAGCGCGCCACCGACG
TCATGATCGCCGGGAAAGTCGCCGTGGTGGCGGGTTACGGTGACGTGGGCAAAGGCTGCGTCCAGGCTCTGCGCGGGT
TCGGAGCTCGCGTCATCGTCACCGAGATCGACCCCATCAACGCCCTGCAGGCCGCCATGGAGGGTTATGAGGTCACCA
CCATGGACGAGGCCTGTAAAGAGGGAAACATCTTCGTCACCACCACCGGCTGCGAGGACATCATCCTGGACAACACT
TTGAGAACATGAAGGACGACGCCATCGTCTGTAACATCGGACACTTCGACTGCGAGATCGACATGAGCTGGCTCACCA
AGAATGCTGCGGAGAAGGTCAACATCAAGCCTCAGGTTGATCGTTACCGTCTGAAGAGCGGGCGTCACATCATCGTCC
TGGCTGAGGGCAGACTGGTGAACCTGGGCTGTGCCATGGGACACCCGTCCTTCGTCATGAGCAACTCCTTCACCAATC

AGGTGCTGGCTCAGATCGAGTTGTGGGTGAACACCTCCAAATACCCCGTGGGAGTTTACTTCCTGCCCAAGAAGCTGG
ACGAGCAGGTGGCCGCCGCCCACCTGGACAAACTGGGGGTGAAGCTGACCAAGCTGACAGACAAGCAGGCCAAGTACC
TGGGTCTGCCCAACGAGGGGCCCTTCAAACCGGACCACTACCGCTACTGAGCCCCCCCCCCCCCCCGAGCCCTGCTGG
GGAGAGGAGAAGAAGGAGGAGGAGTGGCTGGACAGGTGTTGGTGTGTTGGTGGATGATGGGAGCTCTGGGACAGTTTC
TCCAGGTTTCACTCTGGGAGCCAAATATCAAGTTTTCAGGGACAGAAACAGTTTGATTTTCTCTCTTAAAAAACAAAA
AACTTTTCAAACGCCGTGTTCTGCTTTTACTGATCCTCCATAGAGCTGCTGAGTAGAAACAGGAAGTAGACACAAAGC
TTCATCAGTAGTGTGATGTGGGAGTGTTAAGTGTCATAAAGTCGAATCAACAATCAGCCGAAAGTTTAGTTTGTCTCA
TCATGTTATTTCCCTCCGACACCTTCATCCCTCGTCCGTCTCTCCTCTTCCTCATCAGCTCTCCTGAAACACCGTCAG
TGGCCGTCAGAGACTCTTCCTCTTGTTCATGTCATTCCCTCCTTTAATGGAAAAATAAACCTCTTCTATCCGACCTCC
ATCTCGCAGCGTCTCCAGGAGAGAAGGTTTCGAGGCAGACGGTTTAGTGTACAGTGGCCTTATTCCATAACACTGCCC
TGTGTTAGCACTTAACTCAAACTGTACGTGAACTGAAGACGAGCGCCGCTGCCAAAGGAACCTCCAACGCTCCTCCTG
TTTCTGCCTGAGTCTTATTCTGTTGCCTCATTTTCCTCCTTTATTGTGATTTTTATTTAATTTGACTTAAAGGGCCAA
AACATGTGCAACAGCCCCC

>Sequ20407EST2
AGTCATCGTTGACTAGCTGATAGTAGCGAACGACAGACTCCGTCTACACAAGAAAACCCTCAGTTTGCGTGTTTGTTT
GTATACTTTTGAGTGAAAATGCCGCTGGAGAATTTGGAGGAGGAGGGTCTGCCCAAGAACCCCGACCTGAGGATAGCA
CAGTTGAAGTTCCTGCTGACGATGGACGGTCACCGACAGGATGCTAAAGTGAAGACTGAGCTCATGGACGCTATCAAA
GCTAACAATATGGCGCCCTATTATGAGGGTCTGTGTAAAGACCTGAAGTGGCAGCTTGACGGTGACCTGCTGAGTAAA
ATGAAGAAGGCCAACGAGGAAGAGCTGAAGCGTCTGGACGATGTGTTGGAGGATGCGGAGAAGAACCTGGGAGAGAGT
GAGATACGAGACGCCATGATGGCCAAAGCTGAATACCTGATCAGAATTGGAGACAAGGAGGGCGCCCTAACAGCCTTC
AGGAAGACCTATGACAAGACGGTCGCTCTGGGTCACAGGCTAGACATTGTCTTCTACCTGCTGAGGATCGGCCTCTTC
TACATGGATAGCGACCTCATCACACGCAACTCGGAGAAAGCCAAGAGCCTTATTGAGGAGGGGGAGACTGGGACAGG
AGGAATCGCCTGAAGGTCTACCAGGGCCTGTACTGTGTTGCCATCAGAGATTTCAAGCAAGCTGCTGAGCTCTTCCTT
GACACAGTCTCCACCTTCACCTCCTATGAGCTCATGGACTACAAGACCTTTGTTACCTACACTGTCTACGTCTGCATG
ATCGCCCTCAAAAGGCCTGACCTCCGTGAAAAGGTAATAAAGGGTGCAGAGATCTTGGAGGTGCTGCACAGTCTGCCT
GCTGTTCGCCAGTATCTTTTCTCACTCTACGAGTGCCGCTACTCTGTCTTCTTCCAGTCTTTGGCCATGGTGGAGCAG
GAGATGAAGAAAGACTGGCTCTTTGCGCCACACTACCGCTATTATGTGAGGGAGATGAGGATCCAGGCCTACAGCCAG
CTGCTAGAGTCCTACCGTTCCCTCACCCTGGGCTACATGGCTGAGGCCTTTGGTGTCAGCACAGAGTTCATTGACCAG
GAACTGTCCCGATTCATAGCTGCTGGCCGTCTCCACTGCAAAATTGATAAAGTGAATGAGATTGTGGAAACCAATAGA
CCTGATAGCAAAAACTGGCAGTACCAGGAAACCATCAAGAAGGGCGACCTGCTGCTCAACAGAGTCCAGAAGTTGTCG
AGAGTTATCAATATGTAACAGCGGCCATATTCTCTCATTACAGCAGGGGGGGATTAATCTTGGTATACCCACACCTGT
AGTTTGTCTGGCAAAGTGACTTCTTATATTGTGTATATAATAAAATTTGTTTAACTAGTAACGAAAAAAAAAAAAACC
GAACTACCCTAA

>Sequ20451SNP2
TTCTATACTGAATTTAAATGAAACAATAAAAAAACACATGTTGAAAAACAGCTCAAGAGAGACATTCAAAAGGAAGAA
CATTAACAATATTGGTGAGATAGGAAAAAGTGAGAGATTTGTGTCTCGTTTTGCTTTTTGTTTGGTTTCAGTTATTTT
TGCTGGTTTTTCAGGAGGCTATTCTGAAACTGTAACAGCAGAGCAGGGTTTTGTGTTTTTACCAAAAGACACAGGACA
CCAACCAAGGCTTT

>Sequ20473EST2
TCTGTGCGCTTCTGAGCCAAGAGGAGATCTACACTTTTCCACCAGCCTTCTGAGGACCAAGACCAGCCAAGATGATGC
AGTGTTTTCACTTCATGGTGGAGCCGGCCAAAAACTTCACGGCCAAGATAAAGGAATCACACAAGAGAGCAAAGAAGG
ACAAGAGGGAGCCTCTGGATGAGGATCAGCTGTTTGTGGTGGATCTGGCTAGGGACCTCAGCCGAGTGTGTCAGAGGT
CAGCAGTCCTGGAGCACATCTGGAACCAGGATGACACCTGGCCAACTCCTCTCTGCAAGGCTTTCATCCTACAGTGGG
CCTCTATGCTGGAGAGCAAGAGGAGGCCTCTGCAGACTGATGGCTGGCCAGAGATGGATGAGGGCAAACTGCCAGATC
TGATTAATGAGCAGGACCTGATGCAGGCCAAAAATGTGATTCTCAACTGGATCAAGGATGTGAGAGCTCAGCCTGAGC
AAAGTGTATGGCCTGGGGAACCTGTGGCAAAGGTTCTGGAGGACCTGCAGTCAGCCTGGCGTTGGGGCCGTGCACCCA
ATCTGCTGACTGCAATGGAGCTGGTCATAGTGGACTTTAATGCTGCAGCGCCCCAGATAAGGACACCCATCCCGGCAG
CAGTGGCTCATGTGGAAGCAGAGGACTCAGAATATTGGTGCCATATCCTACGTTCCTCAACCAGTGTGGGACTGGATC
TCAGATGCCGCAGTTGAGGTGACTCTGGATCTGGACACAGCCAACCCAGATCTGCTCATCTCCAGTGACGAGAAGAAG
ATGCGCTGTGGCTTTGAGAGGAAGGATGTTCCCAACTACCACCAACGCTTCGACGGCTGGTGGTGTGCTGTCGGGGTG
GAGGGCTTCGGCTCTGGCCGCCACTACTGGGAGGTGGAGGTCGGTGAGCGGGACTGGCGGCTGGGCGTGGCTAAAGAG
TCGGCCCTGAGGAAAGGCTTCAAGTCGCTGAACACCAATACAGGGTACCTGACCCTGCGGCTGGAGAGGGGCACTGAG
CTGAAGGCGCTGACTGTGCCCTTCACTGCCCTGCCGCCTGGTCTCATCCCCCGCAAAGTGGGCATCTATCTCGACTAC
GACCACGGCCAGCTGTCCTTCTATGACGTCGACAAACACTTGCACATTTACACCTACAATGAGAGCTTCACTGAGAAG
CTGTTCCCCTTGTTTGGTACAGTGGAGATTATCAAGGATCTGGTGATCAAGTCCCCAGCAGCTAAGACCCAATGTCTC
TGCTCCACATCCTGCCTCTGGGGTTGAGCTTCCTTCTGTCCCTCTTCCTTCCAAATCCAGTAGACATCATGTTAAAGT
ATAATAAACCAGGTTATGTAATACTGTAATCAGAATCAGAGATCCTGATGTAAAAGACCATCAATAGCTCCAGTATGT
GACCACGCTCTATGCTGCTACTGCTTCACTGTGATTGTTGCTGCCACTTTCTCTCTATAGAGTTATCATGTTGCCCGC
GTAGAAGGAGTCTGTTGCGCTACAGTCCGTCCACTAAAACAGGTCGGACCTCTCAGAATAAGACAATATACTACACAG
TTCAATGGGCCTCTTGTCAACAACTGAACCCATGTTACCATGTTATCATGTTTCCCTTTGCTCACATACATAATGTAT
TTTCATACATTTAAAACAAAGCCTATAGCATTAATGTACTGTATTGTGCAATATTAATCCTGCAGTAAGAATGCACTC
TGTGAATAATAAATAAAGCAAATGTGTAGGAACAAAAAAAAAAAACCGAACTCCCTA

>Sequ20479EST2
GTACAATTCAGTCGCAGAATAGGCTCGGTGATTTTTCGGGTCAGGTCGGAGGGTTCTTGGAGGACAGTTAATCATGGC
CCTAAGGGTGATCCGGCTGGTCATCCCGTCGGGAGCAAATGCTTTCAAATCGGTGAATGCTGTGCAGAAGGCAGGTGT
TCACACAAGCTCAGTGAGAAGCCTGCGGTATGGCTGGTGGGCCTATGCACTGGGCGAAAGGACGACACCACGGTTAAC

GCAGAACAGCAAAATCATCTCTGTGGATGGAAACTTGGCCTCAGGGAAAGGAGCACTGGCCCAGAAGCTGGCTGACAA
GCTGGGGATGCTCTACATGCCTGAGCCTGACACCTTCTACTTGGACAAGATGTGCGGAGAGAAGCAGCCGCTCTCTGT
CGACTTCAATGGGATGTGCAGCCTGGAGAAGTTCTACGTCGACCCCAAAGCTGCTGATGGAAACAGCTACAGGCTGCA
GCTGTGGATGTACACCATGAGGCTGCTTCAGTACGCTGACGCCATCGAACACCTGCTCACCACAGGCCAGGGAGTGAT
CCTGGAGCGTTCCCCATTCAGTGACATGGTCTTCCTGGAGGCCATGTCCAAAGAAGGCTACATCAGGAAAGATTGTGT
GCAGCACTACTATGAGGTGAAGAACATTAGCGTCTGTGAGTTTCTGCCTCCACACCTTGTCATCTATGTAGACCTGCC
AGCCGAGGAGGTGCAGAAGAAGCTGAAACAGAGCAGCAAGTCCTATCTTCAGAATGTGCCCCTGACATATTTGAAGAG
CATCGAGGAGGGATACAAGAAGTCTTTTCTGCCCAAAATCAGTGAAGTGTCAGAGGTGCTCGCTTACGATGCAACCCA
ACCCCAAGACGTTGAAAGGGTGGCTGAAGACATTGAGTATTTGAAGTTTGAAAAGGGGCCGTGGTTGGAGCAGGATGA
CGTCACCTACCACCACATGAGGATGCTCGTGGAAGACAAACAGCAGGTGGCAACCCTGACCCACATACCTAGATTCCT
GCCGGAGATCACCATCGGGGCTCACGACTATGATGAAAAATACTACGCCTATAAATCACTGCCTGGGAAGAAGTACGC
CCCCGGTTATAATGCAGATGTTGGAGACAAATACATCTGGCTGAAGTGAGCGATTTCATCGGCCCCTCAACAAATTCA
ACCCTCGTCACTGGCTGATCGGCTCCCTGGGTGAATCTGGACCACTCGTCCATTATTGATCCGGTTGTCTTCTGTTGT
ATATCCCAAATAATATGTATGTAATAAAATAATAAATTTTGAATCTATTAACGAAAAAAAAAAAAAAAACCGACCTACC
T

>Sequ20506EST2
CCGCTCTCTTGCCCCAGACGAAGTGCACAGTGGTGTATTGTCGGTACCGTTTTTAATTCTCACAAACACCTTCCCGAA
AAAACCCGCAAACATGACCAGGAAATTCTTCGTCGGTGGAAACTGGAAGATGAACGGCGACAAGAAAAGCCTTGGGGA
GCTCATCCATACCATGAACGGAGCCAAGGTGGACCCCAATGTCGAGGTGGTGTGCGGCGCTCCAGCAATCTACCTGGA
CTTTGTCAGGTCCAAGCTGGATGCCAAGTTCGGTGTGGCTGCTCAGAACTGCTACAAAGTTGCCAAGGGTGCCTTCAC
TGGGGAGATCAGCCCTGCGATGATCAAAGACTGTGGTGTGCACTGGGTGATCCTGGGCCACTCTGAGAGGCGCCATGT
CTTCGGAGAGAGCGATGAGCTCATTGGCCAGAAGACAGCCCACGCTCTGGAGAATGGTCTCGGTGTGATCGCCTGCAT
CGGCGAGAAGCTGGACGAGAGAGAGGGCGGCATCACAGAGAAGGTCGTCTTTGCTCAGACCAAGGTCATCGCAGACAA
TGTAAAGGACTGGAGCAAGGTCGTGCTTGCTTATGAGCCTGTGTGGGCCATTGGCACCGGCAAGACTGCCTCCCCACA
GCAGGCTCAGGAGGTTCATGATAAACTGAGGGGATGGCTGAAGACCCATGTATCTGAGGCTGTTGCCAGTTCTGTGAG
GATCATCTACGGAGGTTCTGTGACCGGTGGTACCTGCAAAGAACTTGCCTCCCAGAAGGACGTTGACGGTTTCCTCGT
TGGTGGAGCCTCCCTCAAGCCAGAGATTATCGAGATCATCAACGCCAAGGCA

>Sequ20524SNP2
ACTGGACCTACAGCAGAACAGACAAGTGAGTACTCTGCTGTAGTCTGAGCTGAAGGAGTGGAAGCTTGCAAGAAAAAT
CATGGCTGCAAGGTATGCATTTTTATTTTTGACCCTTACATTAATCTGAAAATGAATTTACTCCCTGTACACAAGTTC
AATTTAGCCACATTTTTGCAGTTTGTAAAACTGTCTGTTGT

>Sequ20554SNP2
GGGAGTAAGTATGGCCCAAGAGAAGAACATGGCGGTCCCACAGTTACTCAGTAACGCCGAGTTAGTAAAACAAGGAGC
TGAAGCTCGGGTTTACCGGACAGAGTTTCTGGGAAAACCGACTATAGTGAAGGAACGGTTTCCGAAACGGTACAGACA
CCCAGTGCTGGATCAAAAGTTGACTCACCGGAGGACGGTGCAGGAGGTGCGATCCATACTGCGCTGTCGGAGGGCAGG
CATATCTACCCCTGTAGTCTACTTTGTGGACTACATCTCCCACTGTATTTTCTTGGAGGAAGTGGTGGGTTCCTTGAC
TGTGTGTGACCACATCGCCTCCATGCAGCAGTCTGAATCCTGTCAGGACCAGGAGCTGGAGTGGCTGGCTCAGAGGGT
GGGCCAGATCCTGGCCAAAATGCACGATGAGGACGTGATCCACGGAGACCTGACCACCTCCAACATGCTGTTGAGACG
TGGCCCGGAGGACCCAGAGTCCAACCTGGTCCTCATTGACTTTGGCTTGAGTTACATCTCTGCTCTGCCAGAGGATAA
GGGGGTTGACTTGTATGTGTTGGAGAAGGCATTCCTCAGTACTCACCCCAACACAGAGGCACTGTTTGAGAAGCTGCT
GAAGAGCTACACAGCGTCATCCAAGAAGTCGTCAGCAGTCATTCAAAAGCTGGATGAGGTTCGGCTGAGAGGTAGAAA
GAGGTCCATGGTGGGATGAAGATCCGAGCTGTGCTCAGGGACGAGGCTCTGTGGAGAAAACATCCAAAAACATGTTGG
ATTTCTAACAAGACTGTTGTAAGGGAAGTAAATAAAAAGTGTCTTGTAGTGAAATGGACAGAAGTTTTCTCTTCATTG
TAATGATGTTGGAACATCTCAGAGCAGGTTATTTGCTGACAGGTGATGAAATGTCACAAGACAGACTTACAGATAATG
TGTTAATGTACAAAGATCTATGGTTACACGTACTCATGCCTGTGGCCTAGATATCTGTGCCAACTTCAGTTCTCAAGT
AAGAGCTAACTGACTGACAAGTGTTCCCTTCAAACCCTTGTAATGTAAGAGACAGCACAGATCAGACTACAGGCAGGA
GTTCACTGACCATCAGCTGTTACACACTGACTTTAATGGGATGTCCTCTCACTGCACAGTCATCTGGATGTATTACTT
TTCTTAATAATAAAATGATATT

>Sequ20598SNP2
GGAAGAAGGACACACATCAGTGGATTGCCCTGAATACACTAAATGAACCGATCGAGGATGTGAAGTATGCAGTCAAAG
ATATCACTGAGGGAACAGACTACGAGTTCAGGGTTTTCAGCAATCAATGAGTCTGGATCTGGAGATCCGAGCCCTCCA
TCTGCAATGGTGTGTGCAAAGAATCCCAACATGAGACCTCATTTTAAAGACCCAGAGGACTTCATTGTTGTCAGAGCA
GGAAATTCTGCTCGCGTCAAAATTTGCTATGAGGCTGAACCTCCACCTCAGATCACCTGGCTGAAAGACGATGAGCCA
ATATCTCCGTGGTTTAATATCGTCAATACAGAGGGAATGTCTCAGCTTGTTATTCCCTCATCAAAGCGGTCAGATTCA
GGCATCTACACTATCAAAGCCAAAAAACTCTGTGGGTGAGGCTTCATTTGACATTGAGGTTAGAGTCACAGACGAACC
AAGACTCCAGGGCCAGTGGAGTTGGAGCAAACAGTCTATGGCAAAGTGGTGGTATCATGGGCTCCCTCTCCAGACGAG
GAGCTCGATGACCGCCTGTACTATATGGTGTCTCAACGCGACTCCAACACCAGAGTGTGGAAGACTGTAGCAGACCGC
CTCTTCACTCACACATACACAGTCAACAACATCCTTCCTGGGATAGAGTATCATTTCCGGATCTACGCCAAGAACGAC
ATGGGCCCCTCAGATCCATCTCAGTCACCTACATGGGGCGCCAATAGCAACAGAGTTCAAATAGCTTCAAATGGAGCT
AATTCGGCGGATGTCTGCTTTGAGAGGCCTCCATCCATCTTGGTCCCTCTGAAAGTCCACACACCACCTAAAGGTTAC
CAACTTTTCATGACATGCGCTGTCCGAGGATGCCCTACGCCCAGTGTTTCCTGGTACCTGAACGACGTCTGCATCAAC
TCAGACAACAACTACTACATCACCAACTCATTTGGTGTGTGCTCCATGTATATCCTCAGAGTCCGACCAATCGACGGC
GGTGAATATAAGGTAGTCGCAGTCAACTCTTTCGGCAAAGCTGAGTGTTCCACTAAACTTACTGTTAGAGATTAAAAA
GGCCTGAAGGTTTCATGCAGCTTTTCCTCTTACTGAGATATCGTGGAAAGTAAGGAAGTAGAATGTTTTAGTTGACAG
CAAAGACTGCATTGAAATGGTGCTATATTGTTATGCAATAGTGAAGATAAAATCATATAAAAATAGTCTAAATACTAG
CTTATTTAATTTATTTGAAATGCTACTCATTTATGATTGTTACCCTGTCATCATAAATAACTGTTTGACAAAGATGTG

TCAAAATGTTTAAGCTCTTAACTGCAATAAATCAACAAAAGACAAAAAAAAAAAAAAAACCGACCTACCCTAT

>Sequ20655SNP2
GATCGCTGCAACACAAATGCAATAACCATGAATACCTTTACAGTGAAAGGCATGAAGTCATTGGCCATGTACTGGGTG
AGAGTAATTGCTACTAATGATGGAGGAGAGGGAGAGCCGCAGGAGCTGGATAATTACATCCTCGCTATGCCTCCTCCT
GTGAGGCCACGGTTCACAGATGCAAAAATCAAGAGTTTCATGGTGGTGAGAGCAGGAAATTCTGCCCGATTGAACATT
AACTTTGAGGCCTCCCCTTGGCCTGAGGTCATCTGGCTGAAAGATGGAGCACCAGTGTCCAAAAAAGTGACCGTCAGC
AATGCAGAGGGTACATCCCAGCTTCTGATTCCCTCCTCTGAGCGCTCAGATACTGGAATCTACACAATCATTGTTAAG
AACATTGTTGGCCAAGAAACATTCAGCATTGAAATTAGAGTCACAGATGAACCTAAGCCACCAGGTCCTGTGGAGCTT
GATGAAAACGTGCCTGGTACAGTGACTATGTCATGGTCCGCATCTCCAGATGAGAAACGTGACGACAGGCTGCACTAC
ATTATCACCAAGCGTGATTCTGTTAAGCGTACATGGCAAACCGTGGCAGACCATCTCTTCAACAACAAGTTCACGGCC
ATCAACATCATGCCGGGAAGGCAGTACAAGTTCCGGGTCTATGCCAAGAATGACATGGGGTCTTCCAAACCTTCTGAG
TCAGCGACCTGGGAAGTGAAGAGAAAGAAAGAGACATTTTCTTTGAACCTTCCTGCCTCTAAGGACTGCAACTTTGAG
ACGCCTCCATCATTCTCCGTTCCACTAAAAACCCACAACAGCCCAGAGAGTTACGAGTGCTACATGAGCTGCGCAGTG
ACAGGAAACCCCAGACCCTATGTTACCTGGTACAGGAATAACATCAGCCTCAACACCAACACTAACTACTACATCACC
AATACATGTGGGGTCTGCTCCATGGTGATACTCAAAGTTGGGCCCAAGGACAGCGGAGATTACACAGTCATAGCAGAA
AACCCTCTGGGCAGGGTGGAGTGTTCAACTAAACTCGCTGTTAAAGATTAGGATGAAACTGTGCTCCTGAACTCCTGA
CTAAGCCTGCTCCATTTAAATGCATGGAAGGTGCCAAATTGAAACCTGTTGTTATCTGTTTGTTGTTTTGTTCTTGTT
TTTTTTGTTGTGAATTATTGTATTAATGTGTTTAAATACACTGAGAGAAAGAAAGGAAGAACTCTTGATAACAATCCA
CAAATGCAAAAAGCACTGATAACAAATGTCAAAGATTCAGACTTTAGAATTTATCTACCGCATGCAAAGAACACTTTG
AGGAGGGACATTTGAGATTCAACATTAGAGATATGCTAATCCTGCACCCAAACATGTTTATTTATTGACCTATTTTTC
CTGGAGCTGCGACTACACTAAGTTTCCTCTTCTGTTTTCTGAATAAGACAGAACATTTACTGATTCTTCTGAAGTTAC
CATCTCATGGATAATTGACATGAAATTGAAATTTATATGATAATATGGCTACAACTGAGTATGTTTTAGTGTGTGTAT
GTTTAGTGTTTGAATTATTAATCATTGTGCAAAAATGTAAATAAATCCATTCAGTTCAAACGTAAAAAAAAAACCGAAA
CCT

>Sequ20662SNP2
GGTTCTGCTCCTAATGGTTGTGATACCTTTTTCGCTCCACATTGAATGATGCTGGGCCAAGTCTTCCTAATTCTGACG
TGTATCATATTCGAGCATGCACCTGTTGCTTTTTACCGTCATGGCCGTCTCCTGTGTGCTCACGGTTGGACTCTTTAT
GAAGGATCTGGCAGCTCTTTCTGGACGGGTGGAGATTGTGGGTGTTGGGTGGGAAGCGGGTGGGAGGTTGGCAGGGTG
TGTGTGAGTGGAGGGATCTTAGAAGATGTGAATGAAGCATGCCATATTATTGCCAAACCGTCATAAGTAACGTTGGTG
ACCTGCATTTACTTGCATCTTTTCCCTCTTTGTGTGATTTCTCTGTCTTCATCTTTGGTTAGAATTGCGTCTCTAATT
GCAAAATGCCTTAACATTGTCTGTTTTTGGGGTGGGGGGGGGGGGGTTTTGTTTCACATATCGCGTATTGTAAAACGC
ACCGGCTTTCCAGAATCAAATCCAGACACAATGCTCGTGTTTCCACCTCTGAGCTCCGAGAAGCTCAAATGACCACGC
TTGCTGTCGTAGCTTTGAATCTGCTCCACTATATCACTGAAGACTGGAGTTAAAACAGTGGAGCCATATTCTCCTCCC
TGGCTTTTACATAAAGCAAAACACACACAGGAGTCTCGTATATAAGGCTGCCATTTCACCTTGATATCCTTTCTGTCA
TATTAGCAGCGCTTACAGGCTAGTATTAAGCCAACTCTGTGCCTTCTGCACTGTCGAGCTGCATCCTCTTCCCTGTAC
ATACAGTAGATAGGACCATAGGTTTAATGTGGCTAGGATGTCATTCAAATCCTTTTCCCTCTCCAGTGGAACTGCTGT
ACATGAATTCAAGTTTAAATAAACTGTCTCTAAAGATAACAAAAAAAAAAAA

>Sequ20663EST2
CAGCAAACAGCCGGGTCCTTCTCTGCGCAGCAACCCCGTGGCTCTGGAAGGTTCTAACCGGGACCGGGTGGAAGCAAC
AGAATCTGAGACCATCCGAAGCCGAACCGCTGCGTACCAGCGCCGGGACTGATCCGTCCCCTTCTGAAGACTCGTCTT
AACATTTCCCGTCGATTATAAAAGTTGCGATGCCCGAAGCAATGGCGGCACCGAAAGCCTTAACCGGAGGCAAAAACA
AAGGAGGGGCGTATGTGGACCGCGACAAGCCGGCCCAGATTCGGTTCAGTAACATCTCTGCTGCTAAAGCTGTTGCAG
ATGCCATCAGAACAAGCTTGGGACCCAAAGGCATGGACAAGATGATCCAGGACGAGAAAGGTGACGTGACCATTACCA
ACGACGGGGCCACCATCCTCAAGCAGATGCAGGTGCTCCACCCTGCCGCCAAAATGCTGGTGGAACTGTCCAAAGCCC
AGGACATCGAGGCAGGCGACGGCACCACCTCTGTGGTGGTGATCGCCGGAGCGCTGCTGGACTCCTGCTCCAAACTGC
TGCAGAGAGGCATCCACCCCACCATCATCTCCGAGTCGTTCCAGAAGGCCGTGGAGAAAGGCGTGGAGGTGCTGACGG
GCATGAGCCGGCCGGTGCAGCTGAGCGACCGCGAGACGCTGCTGAACAGCGCCACCACGTCGCTGTGCTCCAAGGTGG
TGTCGCAGTACTCCAGCCTGCTGGCGCCCATGAGCGTGGACGCCGTCATGAGAGTCATCGACCCGGCCACCGCCACCA
GCGTCGACCTGCAGGACATCAAAATCATCAAGAAGCTCGGTGGGACCATCGATGACTGCGAGCTGGTGGACGGCCTGG
TGCTGACCCAGAGGGTGGCCAACACCGGCGTGTCCCGCGTTGAGAAGGCCAAGATCGGCCTCATCCAGTTCTGCCTGT
CCCCTCCCAAAACCGACATGGACAACCAGATCGTGGTCTCAGACTACGCCCAGATGGACCGCGTCCTTCGGGAGGAAC
GCGCTTACATCCTGAACCTGGTGAAACAGATTAAGAAGGCCGGCTGCAACGTGCTGCTCATCCAGAAGTCCATCCTCA
GAGACGCTCTGAGCGACCTCGCCCTGCACTTCCTCAACAAAATGAAGATCATGGTGGTGAAAGAAATCGAGAGAGAGG
ACATCGAGTTCATCTGCAAGACGATCGGCACCAAGCCCATCGCCCACATCGACCACTTCACTCCAGAGATGCTCGGCA
CAGCAGAGCTGGCAGAGGAGGTCAGCCTGGACGGCTCCGGCAAGCTGGTCAAGATCACAGGCTGCACCAGCCCCGGGA
AGACGGTGAGCATCGTGGTCCGCGGCTCCAACAAGCTGGTGATCGAGGAGGCCGAGCGCTCCATCCACGACGCGCTGT
GTGTCATCCGCTGCCTGGTCAAGAAGAGGGCCTTGATAGCCGGCGGTGGCGCTCCAGAGATCGAGCTGGCTGTGCGTC
TGGCCGAGTACTCCCGTACCCTGGCCGGCATGGAGGCCTACTGCGTGCGGGCGTACGGCGACGCCCTGGAGGTGGTCC
CCTCCACGCTGGCCGAGAACGCCGGCCTGAACCCCATCTCCACTGTGACGGAGCTCCGCAACAGGCACGCCCAGGGTG
ACAAGATGGCCGGCATCAACGTCCGCAAGGGTGGGATCTCCAACATCCTCGACGAGCTGGTGGTGCAGCCTCTTCTGG
TTTCCATCAGTGCACTGACCCTGGCCACAGAGACGGTCCGCAGCATCCTCAAGATTGATGACGTGGTGAACACTCGAT
AAGAGACTTGAGTCTGTCCAGTGAGTTCTGTCACTAAGCCTGTTCGTGCTGTGAGGGATCAGAGGACTCTGTGTCGAC
ACCAGTCTGTTCAGATTTCCACTGTAGTTGTTACGTTGCATAATTTAATAAAAAACAAGGCTTGTTTTTTTGTTAGAC
ACTACGAAAAAAAAAAAAAAAACC

>Sequ20671SNP2
CTGTTGGTGCTGCTGTATATCAGCATATTGTCGCTGAATGCTACCAGCAACACAGATGATTACACAATTTACAATGAG

TCTGTAACTATGAAGAAGGGAGCTGTAATCTAAATTGTTACCTGGTAAAAGATGAAAAACAGTATGATGTGTTTGAGT
AACACTGGACACACAGATGTAGTTTAAGCCCTAGTCAGTCAGAACCAAAACGAGTCCAAACCAAGAGGCTTCTTCAGT
TTTTGATTTACAGAGTACTCACCAGTCAATACACTTAACACACAAAGCATCTGAATTAAAATAAAATCAGATTTCCCA
GGGTTCATCGCTCTCACGCTAATTCTCTGCTGCATAATTTCTGGGATTGTTCTGTCTGTCTGGGGACATCTGTGTGTG
CTAAACTTACACTAAATGGCTTATGAATATAAAATGAAGAAATCTGGCTTCTGTTTCTTTCTACGGTGCTCTAAGGGA
GTGAAATTTAAAAATAAAAAAATCTTTTAAAAAAGCTTGAAAAGTGAGGAAGCTCTGCTGTATTCTGCCATTTTCCGG
CTCTTTGTGAAACTTTTAACTGTAGTGTAGTTGTAGATCAGTGACATGCATTTGAGTCTCTTAGATTTAGAGCACTGA
TTGCACTCTTATTTCATTAAGCACAACTCAAAAGCACATGCATACAAGAAGGGCAGTGGATATTTACAGTAAAAACGA
CAATAACATCACAGGGAGGCAAAGTAGTGAGTGACAGTTGTTCAGGACTTGGACTAATTTGGTTTCGCAGTATGAGTT
TGGGGCTTCTCTGGCTAACCAGTAGTCTCTGTTTGCTTTGGCATGTGTCACTGTGTGCTCTGATTGTCAGTTTATGGT
TTTCAAGGGAATTAGAGTAAACAGGAAGATGAAATATTTGCTCGAGTTGTTGAGTCATACTGACTTGATTGAAATGTC
TAAAATATTTGCACTGTAACTTGGGGAGTCGTAACACGGGGTTTGTACTTGCTGTAACTGTGTGTCGCTGTATGTCTT
TGAGGCTGGGTTGATTGTATCTATTTGGCATGGTCAGTAAAACAGCTTAGAAACTAAAAAAAAAAAAAAACCGACCTA
CCCTATA

>Sequ20674EST2
TACGTCAGCACCGCGACACATGCGGCAGCCTCTCTCCCCGTGTTAGCCGTTAGCCGCGCAGTGCAGACGGTTGAAGTC
TTTCTCCGGACTCGGTGAGTTCAGGTCGAATAAAACGCTCAGAGGACGATGGCTGCCACCGGGACGCAGGGCAGGAAG
AGGCTCCTGAAGGAGGAGGACATGACCAAGGTAGAGTTCGAGACTAGCGAGGAGGTGGATGTGACCCCCACCTTCGAC
ACCATGGGACTCCGAGAGGACCTGCTCCGCGGCATCTACGCTTACGGTTTTGAGAAACCGTCTGCGATCCAGCAGAGA
GCCATCAAACAGATCATCAAAGGCAGAGACGTCATCGCACAGTCTCAGTCTGGAACAGGAAAGACGGCCACCTTCTGT
GTGTCAGTGCTGCAGTGCCTGGACATCCAGGTGAGGGAGACCCAGGCTCTGATCCTCGCTCCCACCAGAGAGCTGGCA
GGACAGATTCAGAAGGTGCTGCTTGCTCTGGGAGACTACATGAACGTTCAGTGTCACGCCTGCATCGGAGGGACCAAC
GTGGGTGAGGACATCAGGAAGCTGGACTACGGTCAGCACGTGGTGGCGGGGACACCTGGACGAGTGTTCGATATGATT
CGTCGCAGGAGTCTGAGGACAAGAGCCATCAAGATGCTGGTTCTGGACGAGGCTGATGAGATGCTCAACAAAGGTTTT
AAGGAGCAGATCTACGACGTGTACCGTTACCTGCCCCCGGCCACACAGGTGGTTCTGATCAGCGCCACGCTGCCACAC
GAGATCCTGGAGATGACCAACAAGTTCATGACGGACCCGATCCGGATCCTGGTCAAGCGTGACGAGCTGACCCTGGAG
GGGATCAAGCAGTTCTTTGTGGCCGTGGAGAGAGAGGAGTGGAAGTTCGACACTCTGTGTGACCTGTACGACACTCTG
ACCATCACACAGGCCGTGATCTTCTGTAACACCAAGAGGAAGGTGGACTGGCTGACGGAGAAGATGAGGGAGGCCAAC
TTCACGGTGTCGTCGATGCACGGAGACATGCCTCAGAAAGAGAGGGAGTCCATCATGAAGGAGTTCAGATCAGGAGCC
AGTCGCGTGTTGATCTCCACTGATGTCTGGGCTCGAGGTTTGGACGTTCCTCAAGTTTCTCTGATCATCAACTACGAC
CTGCCCAACAACAGAGAGCTCTACATCCACAGGATTGGTCGATCTGGTCGTTATGGTCGTAAAGGCGTGGCCATTAAC
TTTGTGAAGAACGACGACATCAGGATCCTGAGAGACATCGAGCAGTACTACTCCACCCAGATCGACGAGATGCCCATG
AACGTGGCCGACCTGATCTGATCTTACCTGGATGACATCACACCAGAGGCCCACCCCTCTGACCTGTTCTTTTCTGTC
TTTTTTATTTTTTCTTAATAGTTTTAGAGTGTTTTCTTAATGCGACTGTTGGGGTGAAAACGACTGTAATACTCCAGA
TTTGTTTTTGTAAATAAAGTTGTTTTTGGTAACTAAAGAAAAAAAAAAAAAACGAAAAACAACGTAGTACGGT

>Sequ20679SNP2
GCAGCCACGGGAGATATTGTTCAGGTGTACAAGGACGTGTGGAGCACTCCAGTGACTAAACCCTACATGGCTCACAGA
GCTCAGGGAACAGTGTGGGGGCTGCACTTCTGTCCCTTTGAGGACGTCCTCGGGGTCGGACACGGAGACGGTTTCACC
AGCATGCTCGTACCAGGTGCGGGTGAACCTAACTTTGACGGTCTGGATGCAAATCCATACCGCAGTGCAAAGCAGAGG
CAGGAGTGGGAGGTTAAAGCCCTGCTGGAGAAGATCCAGCCGGAGCTCATCACCCTGGACCCCAATGAACTGGGACAA
GTTGACCACGCCACCTTTCAACAAAGGCACCAAGACAGGGTCCAAGCTCTGGGCTTTGACCCACTTGCCAAAGAAAAT
TTATTCCCAAGTTTAAGAAAAAAGGTCGTAGTTCTGCTGGCAGTGTTGAAAGGCGCAAGAAGCAAGTGGCTCATGAGG
ACCAGAGGGATATAATCAGGAAACTGTGGAGGACAAAATGAAGATGGAAAAGAAAGAGAGGAGAGGGAGAAAAGAAGG
CAGTATTATCTAGCTCGAGATCCGCTCTGGACAGATTCAAAAAATAGAAAAGGAGGAGCCTTTGTGCCGCCCCAAACC
CCACTGACATTCTGTGGGAGTGACTTTTGAACTTCATACAAAGGACACATTTCTTGGAGACATTACTCAACTGTGGGA
ACAAAACACAAAACTGCTGAAATTCCCTATATGGTCTGCTAATGGTTAGCAGTTCATCTGTAATCAATTTTGAGTGCA
GTAGTGGGCTGTTCTATTTAAAGCCTGTAGCATGAAGAACTTTCTGTCTATGTGGGCAAATAATGTGAGGTGCAAGGC
TGGCATAAGTGTCACCTAGAATAAAGGTTTAGTTGTGTTTTTTCTCTAATGATAAACAAATTTTCACTCACTTTTGAC
CTTTTCAAATGATATGCTTTGGTGGCGGAGCTGACTGAACATGTGTCAAATGTTTTATATTAATATGTTATAAAAAAT
AAACAAC

>Sequ20681SNP2
GCCAAGCTGTCGGATGGCGTGGCCGTGCTCAAGATCGGAGGAACAAGTGACGTGGAAGGTGAACGAGAAGAAGGACCG
TGTGACGGACGCTCTGAACGCCACCCGGGCAGCTGTGGAGGAGGGGATCGTTCCAGGTGGAGGCTGCGCTCTGCTGCG
CTGCATCCCATCGCTCGACAGCCTCAAAACCGCCAACGCCGACCAGAAGATCGGTGTGGACATCATCAGGCGAGCGCT
GCGTATCCCCTCGATGACCATGCTAAGAACGCCGGTGTGGAGGGCTCACTGGTGGTGGAGAAGATCCTGCAGGAGTCA
GCTGATATCGGCTACGACGCCATGCAGGGGGAGTACGTCAACATGGTGGAGAAGGGCATCATTGACCCCACCAAGGTG
GTGAGGACGGCGCTGCTGGACGCCGCAGGAGTCGCCTCCCTGCTCTCCACCGCCGAGGCCGTCGTCACGGAGATCCCC
AAGGAGGAGAAGGAGATGCCGGGAGGTGGCATGGGCGGCATGGGAGGCATGGGCGGCATGGGAGGAGGCATGGGTTTC
TAAGCCGGTCCTCCTGACTTGTTGCTGTACAGACTAAAAGGCAGAGCGGTGCTGGGGGTGGCTGTGGAGCACACGCAG
AAGAAGAAAAAAACAAAACAAAACAAAACCCTCCAAGAATCTGCAACCACCTCCAGCGAGCTCTGCCCACACTGACTG
GCCATGAGTATGTTGGAGATGCCCACGCTGTTTAATTCTGTCTCCTGCCTCCGGAGGACCCGGTCCGGAGCAGGAGAC
CCCATCGAGCCCAGTTACTTACTACACCAGAACCCTCCGACGGCTCTGCTCATCGAACCAATCCACATGTTCAAACTC
ATAGTTCTTCATGTAAAACGCTCGTACAGGTGATGCCACTGTGATCTCTGTAAGAGGTAGGAAAGTGATTCAAGTCGT
CTAAGAAGAAACTTCAGAAACTAACATAGTCATGCGTATTGATGCTCTGTATTACTGCAAAAGAGAGCAGGGCAGGTA
CGAGAGAAGATGCCTAGCCAGGAATGTGTCTGTCCTTTATTTTGTCCTTGTAGTATTTTGTTAATTCGTTTTGTTTTT
TTATATATATATATATATATATCTGTTATGGTTTTCTTCCCCCCCAGCTGATAACGACGTGGGTTAAAATCTCTCATT
TGATTTTAATTAAACCAGTTTTCTTTCAGAGCCTGAGCATTGTCTGTGTCCCTGAAGTGACGTTACATTTTGTTTGTC

TGGTGATGATGGAGGAAAGATCACCTGTTCCCTCGTACCTGACCGCGGCTGGACAAAGAGTGCCTACGAGTCAAAACA
CCAGAGTCTGAGTATCACCAGGGGCCTGTTGAAAACAACGTAAAAAAAAAAGTAAAGTTAAATGTTTACCGAGATGTC
TGGCCTTTTTCTTTTTAAGCCTTTGTGAGTTGTTTTCACGAATGCAGCGTTTTTCTAAACATCAGTTTTGACGTCGCA
GAAGATTAATTTCACAACAAACGGCTGATTGTATGAGAGCGCGGAGGAGTTCAGACAAAATGTGAAAACTTGTGGCGT
CCAGGTGCTGCCTCTGTTCTGACTACGAGTCACAGAAAACTACCGACCATTTATCACTTACATAAAAAATCTGAGAGC
GTGGTGTTAGTTTTACACATAATTACATTATTCCTTTGCAAAATGGCAAATAGCTGTGATCATTCGACTTCTGTTTTG
TACAATGTAAA

>Sequ20704EST2
GCTATCCACTCTGGTCTGCCCCCTCTGCCTGCCTGCTCGCCTTGCCAACCGAGCCCTTCGGTCTGACCTTCCCAGACT
CAATTCATCCAAAAGAAGAAAAGATCCACTCCAGATTTCAGAATGTCTTCAGCATATAGGACCACCTCTTACAGTGTG
AAGAGTTCCAACGCCCCACGGAGCTTCAGCAGCAGCTCCTATGCTGGACCAGGCGGTGTCACCTCCCGCAAGAGCTAC
AGTGTCAGGAGTTCCTTTGGAGGAAGCAACAGGGGCTATGGAGGAGCTGGCATCACTAGCTCCTCTTCCTATGGCCTA
AGCTCAAGCGCAGGCATGGGCATGGGCATGGGTGGTGGCTATGGTGGTGGCTTTGGTGGTGGCTTTGGTGGTGGCATG
GTTGCTCAGGCCCCCATCACCGCCGTCACAGTGAACAAGAGCCTTCTGGCCCCCCTGAACCTCGAGATCGACCCCACC
ATCCAAGCCGTCCGCACCCAGGAGAAGGAGCAGATCAAGAGCCTCAACAACCGCTTTGCTTCCTTCATTGACAAGGTC
CGCTTCCTGGAGCAACAGAACAAAATGCTGGAGACCAAGTGGAACCTGCTGCAGGGACAGACCACCACTCGCTCCAAC
ATCGACGCCATGTTCGAGGCCTACATCGGCAACCTGCGCAGACAGCTCGACAGCCTGGGCAACGACAAGATGAAGCTG
GAGGCTGACCTGCACAACATGCAGGGCCTGGTGGAGGACTTCAAGAATAAGTATGAAGATGAGATCAACAAGCGCACA
GAATGTGAAAATGACTTCGTCCTCATCAAGAAGGATGTCGATGAGGCCTACATGAATAAGGTTGAGCTGGAGGCCAAG
CTCGAGAGTCTGACAGATGAGATCAACTTCCTGAGGTCGATCTACGAGGAGGAACTGCGTGAGCTCCAGAGCCAGATC
AAGGACACTTCAGTCATTGTGGAGATGGACAACAGCCGCAACCTGGACATGGACGCTATTGTGGCTGAAGTGAAGGCT
CAGTATGAGGACATCGCCAACCGCACCCGTGCCGAAGCAGAGACATGGTACAAGACCAAGTATGAGGAGATGCAGACA
TCCGCCAACAGATATGGGGATGACCTGAGATCCACCAGGACAGAGATCGCAGACCTCAACCGCATGATCCAGAGACTG
ACATCAGAGATCGATGCTGTCAAGGGACAGCGTGCCAACCTGGAGGCCCAGATCGCAGAGGCTGAGGAGCGCGGTGAG
CTGGCTGTGAAGGACGCCAAGCTCCGCATCAAGGACCTGGAAGACGCCCTGCAGAGAGCCAAACAGGACATGGCCCGC
CAGATCAGAGAATACCAGGACCTGATGAACGTCAAGCTGGCTCTAGACATTGAGATCGCCACATACAGGAAACTGCTG
GAGGGAGAGGAAGACAGGCTGGTGAATGGCATCAAGGCTATCAACATCTCCCAACAGAGCACAAGCTACAGCGGTTTC
CCCATGGACACCATGAAGAGTAGCTACTCCAGCGGGTACTCCAGCGGTTTTAGCGGTGGATACGGCAGCGGTAGTGCA
TACGGCAGCGGCGGTGGATACAGCAGCGGCGGTGGATACAGCAGTGGCAGTGGATACGGCAGCGGCATTGGCGGCTTC
AGCGGCGGCAGTGCTGGCGGATACAGCACCACCCAGAGCAAGAAGAACGTCGTTATCAAAATGATCGAGACCAAGGAC
GGTAGAGTGGTGTCCGAGTCCTCTGAGGTCATTGAGGATTGAGCTGTCTAGTTTAGAGGTGTAGCTACCTGTCTGCTA
TGATCTCCTCTGCCTCTCCGGTAGTTTTATACTCACAGTTCACCCCCTCGCCCAAAACTTTCATAATAAACAAGTGTG
AAGTAAAATGCTTGCCAGCCACTCTCCAATACAGCCTAAACATGTTTTTTCATGAAACAGTAAATGAACACTACCAAA
GCCCTGAAAGGGACCAAAAGAATTTATGATCTGATGTCTAACTGTCTGTACTAAAAGAAA

>Sequ20712EST2
TGGGGGCTTGTTTAAGTCTTCCCTCATTCACGCAACCATCTGCTATCGAAATTAAGCTAAGAAATAACATTAAATTGA
ATCAAAATGCCGTGTGGCAAAAACTTTAGAAGGAGAAGGGATTCGTCAGATGTAGAGGAAGAAGAGACAACTGAAGAA
GTCAGATCAAAAGTAGAAGAGGCTAAAGAGCTTCAGAGTTTGCGGAAACGACAGAGCGGAGTCAGTGTTACCGCCCTG
TTGGTTGGAGAGAAACTGCCACCAGAGGCTGAAATTGATAATGACCCATTCAAACTAAAGACTGGAGGAGTTGTAGAC
ATGAAGAAAGTCAAAGACAGGAACAGGGACATGACAGAAGACGAGACAGACCTCAACCTGGGCACCTCTTTCTCTGCT
GAAACTAACAGAAGAGACGAGGATGCAGACATGATGAAATATATTGAGACTGAGCTGAAAAAGAAGAAGGGCTTGGTG
GAGGCTGAGGAACAGAAAGTTAAGGTGAAAAATGCAGAGGACCACCTGTATGAGCTGCCTGAGAACATCCGAGTCAAC
TCTGCCAAGAAGACTGAGAGATGTTATTCCAATCAGATGTTGAGTGGGATCCCTGAAGTTGATCTTGGCATTGATGCA
AAGATAAAGAACATTATCCAAACAGAAGACGCAAAAGCCAAGCTTCTGGCAGAACAAAGGAACAGAAAAAAGACCATG
GCACATCATTTGTACCAACCAACATCGCTGTCAACTATGTCCAACATAACCGCTTCTATCATGAGGATGTGAATGCAC
CACAGCGGCATCACAGACACAGAGAAGAGCCCAAGGCAAGACCGCTGCGTGTGGGAGACACCGAGAAACCCGGTCCAG
AAACATCATCGCCACCTAACTACCGCAAACGTCCAAACAACAAAAAGGCCACAGATGACTACCACTATGAGAAATTCA
AGAAGATGAATCGACGATATTGAGGAGCTACACGTTCTTTTGTTTTTGTTTTAAGAGTTACTCGACATGATGCCCACT
TTTAAAGAAATTGAGACATAAAGATTACTTGTAGACTAGTTTCCCAAAGCAACTGTTAGGGACAGAACTCCCAAGACA
CTGTTGTACTTTGTAAATATTGTATATAGCTCCGTGTCATTATCTGCAGGCAGTACATCTGCTTTGTAATGTGTTTGT
GTTAAAGGACAATTCATCTAGAAGAAATCATGTATGCTCATTTTGTTCAGGATTGTCTGATAAAAATGAATTCAGAAA
AAATAAGACCATAATGTTTTAGGAAATACAGGAAGGACTTGATATATGTCAAACTGATTGGTATTCCTGCTCTG

>Sequ20719EST2
CTTCCATTCCAAGACTGGAGATGCCATGGGGATGAATATGATCTCTAAGGGTACAGAGCAGGCTCTGAGCAGACTGCA
GCAGAACTTCCCAGACCTGCAGGTGGTGGCTGTCAGCGGAAACTACTGCACTGACAAGAAACCAGCCGCCATCAACTG
GATCGAAGGCAGGGGCAAGTCTGCCGTCTGTGAAGCCACCATCCCCGCTAAAGTGGTCAGAGAGGTTTTGAAGACGAC
AACACAAGCTCTGGTGGAGGTGAACATCAGTAAGAACCTGGTGGGCTCCGCCATGGCAGGGAGCATCGGTGGATTCAA
CGCTCACGCAGCCAACCTGGTGGCTGCCATCTACATCGCTTGTGGACAGGATCCAGCCCAGTCAGTGGGCAGCAGTAA
CTGCATCACCCTGATGGAGGCATCAGGACCAACAGGAGAGGATCTGTACATCAGCTGCACCATGCCTTCTATAGAGCT
GGGCACTGTGGGAGGAGGCACCAACCTGCCCCCCCAGCAAGCCTGCCTCCAGATGCTGGGTGTGCAGGGAGCCAGTCA
GGACTGTCCAGGGGAGAACGCCCGGCAGCTGGCCAGGGTCGTATGTGCCACCGTGCTGGCCGGAGAGCTCTCTCTGAT
GGCTGCTCTGGCGGCTGGACACCTGGTCAAGAGTCACATGACACACAACAGGTCCAAGGTGAATCTCCAGGAGACTCC
AGGAACCTGCAGCAGGAAAGCGTCCTGAGAGCAGTGTGGCCTCCTGCAGGAGCAGTAACTTCAGGAATCTATGGACAA
TCTAGAAAAAGACTAATCATTCATCAATAGAAAAAACAGACCTCAGACGTTCTCTGAGTGACACAGGAGTATCATGTG
ACCAATCTCTATGCTGGCTATGAAAAGGATTCATTCAAGCTAATGGGGACTGTTCTGTGGGAAATATAAATGTATCTG
GTTATGTCTTATGGAGCTAGAAGCAGCTAATTACTGGCTTCTTTCATTTCCAAGGTTAAGAATGTCGTTAGGCTTTTG
GGTTCAGATGAAACAGCTCTCCGGTCACCAAACGAGCACCGTGAACACAGAAACACTGATACCTAAAGGAGCCTGCAG

ACTGTACACAGACTATAAGTTTACTGCAGGTCACACTGTGCAACATGGGTCTAATAAACATGGCTACAACAAAAAAAA
AAAAAAAAACTAAGTAGACTAG

>Sequ20725EST2
CGAATGCGGCAAGGCCTTTGTTGAGAGCTCCAAACTCAAACGTCACCAACTTGTTCACACAGGGGAGAAACCCTTCCA
GTGTACCTTTGAAGGCTGTGGAAAAGGTTTTCTCTGGACTTCAACCTGCGCACACACGTGCGGATCCACACTGGAGAC
CGACCCTACGTCTGCCCTTTCGACGGCTGCAATAAGAAGTTCGCCCAGTCAACCAACTTAAAGTCTCACATTCTCACA
CACGCCAAAGCCAAAAATAACCAATGAGATCCCGTCCACCTGCGTCAAGCCACACAGACGAAAAAGAGCATCCTAGC
GGCACACGTGAAACCTCTTTTGAAGACGGAATGAAAAGCTTGAGACTTACTGATTTATATGAAAAAGAATCTGACAGA
TTAAAAGACTTTAAAACACTGAAATTCAGCTAGTTTTCCTGCGTCCCCTTTCTTCTGCTGTCATATTGGATGAGGAAC
ACAGTGACTATTCCCCAAAGCCCCGGCCAACACAGTCCCTTTGTTTCTCCAGTGGCACTCGGCTGCCAGAAGATGGAA
CCCATGGACACATCAGAGACTTATTTTTACCAGAGTGAGCAGAGAAGCAGCAATCTATTAGTATTTTTATTTTCTCGC
ATAGCTTTTATTTTCCTCAAGTGTGCATATTGTACACTTGACCCAGGCTATGTTTAGTAAAATTATGTATTTTTTTTT
TCCTCTTGCATTATGAGATCGTACCTACTAAAGGCCTCAGCCTTGTTTGACCTGTTTCACATGTATGAGAAGTTTAAA
AACAGCTGTATGTTTGCATTTCCATACCCTCTTTGGTTGTATTTTTCTCTAACCACAAAATAACCTTGTATACTTGTA
TTGTATGGCTGTATTAAATTACGTAGACATAGATAGAGGTGTGATTTAAAGTGTTAACCAATTAAACTTTTAGTCATG
AGTTGCTTTATATTTTCTATGAACTGTCTTTACACAGATGAATGAGTAATATAAACTTACTCACAGCCTGTCTCGGTT
AAAGTATCTGGAGCGTACATTGAATTGACTGCGAGACCATCACTTTCTTTGTTAGAATGTAATGTACAGACGTCAACC
ATTAACTTCCTTTGTTGATATTTACACCCATGTATCTCAACTGCCCACAATAAAATGGGTTATTCCAACCCTGTCGAA
AT

>Sequ20726EST2
ACATCCGCTCCATGGTCACTCGTGTGATTTTACAGGAGCACTGGCACACGAAGAAACGCAAACCTTACCTATACCGAG
AGCAGGAGATGATGGTCGGACCTTTTCTGAGAAACCTGTTAACGGGACTCACCTACAGTCTGACTAAATACAACCCAG
TGCTCCTCCTGTCAAGTCTAGATATTAATCCCCAGGTGCATTTTTATTGGAGGAGAGGACAGAGAATCATCCCAAAGG
GACACCGGAGAGGCCGGCAAGAGCCAACCAGGTTCCAGATTGATGACCAGCCGCTCTGTCAGATTCGGACCACTCAAC
AACTGCCACAGTTTACCCCGCTGGAGGCTTCGTATGCAGCTGAAGTACCAGAGATCACACTTTGTCCCAATATGATGC
CCCTGTTCAGAAGGCAGTACGACAACAACATCTTTACAGGCGCTAAGCTACCAGACCCAGCATGCTACGGTCACACTC
AGTTCCACCTGGTTCCTGACCGATTCCACCGAGAGCGGATGGCTCGGCAGCAGATGTCTGACCAGGTGGAGGTCTTTC
TCCGAGCTAACGGACTGGCCAGCCTCTCGCCTGGACGGGGGCTCAGGCCTTGTACCAGGGTTTCTGGGACCAAGAGGA
CGTCACCAGGCCTTTCGTGTCCCAGGCTGTGATCTCCGATGGCCACTTCTTCTCCTTCTTCTGCTACCAGCTCAACAC
GGTGGCTCTCTCCGTGGAGACAGACGCCAACAACCCCAGGAAGAACCTCCTGTGGGGCACAGAGAGCCTGCGACTGTA
CGAGACAGTGCAGGACGGAGAGGTGGTGGGTCTGAATGACAGTGTCATCAAGCTGCTGGTTCAGTTCCTCATGAACCG
GCCGTAGACTTCTTCGGGTGTCTGTCTGCTCTTGTGATTCTTTGCCTATATCTGTCTGTGTTTTCTAAGGCGTTGGAA
GAAGCAGGAATTTAAGAGAAAGAGCTCTTGGCAGAAATGAGGAAGTTTTGCCAGATTGTATAAAACTGTAATGTTCAA
AAAGCAGAAAAATCTGTGTTAAACAAATGAACTGAACAAAGTATTGAAAAATACTAAACACACATCCCTGGTTATTAT
TACTGTGCAAACTGTATTTATGCAAAAATAAAATCATGCTTTACAAAACAAAAAAAAAAAAACGAACGACTAGCGACGA
CGACACACGGGAGTAG

>Sequ20737SNP2
GCTTGTCCATCTTGTTATTTCTGTGCCAGCTCCAGCATGCATGTCTGCCTCTCTGTTATTTTGTTGCTTCGTGTCTGA
ATACACGTCTCACTTTGATTTCCAGCATCTGCTTCTCATCTAACAACTCATTCAGCACCTCACCAAGAGGACACAGCA
GCCGGCCGTCATTCTGGAGGGAAAGGCCCCAGTTGAGTCCAGCAGGGGGGATGGAGGAGGCGGGGAGGCCACTTCCTC
CAGCCCCAGCCCTGCTGCCCTGCCTCTGACCCAGGCAGAGAGCGCCGGGCCCTGGCAGAGCGAGAGCCTGCTGGCAGA
GTCCTGGTCCACGATGGGCGACGTGGACCACGAGGACACCAAGAGCCTCGACAGCAGCGACGGGGTGGTTCTGCCCGG
GGAGGAGAACCACTCCTCCAACTCTGACATGGTCCACCTGGAGCGAGAGGAGGTGGAGATGCTCGAGGAGGCGGAGAA
GGAGGCCGCAGAGGATGAGAGGGCAAGGAGAACAGAAGAAGAGGAGGAGGAGGAAGATGAGGAGCTGCAGACGAGTGTGTT
GAGTGTCTTAGGCGGGGAGAAGGAGCTGGTGGAGCTCAGGGAGGAGGAGCAGGACCTCCAGGCCCCAGAAACTGAGGA
GCTCCTGGTGTCAGCAGAGGAGCCGCATGTGAAGAAGACGCCGCAGAGTTCAGGCAAGTGGTTCCCCCGATGGCTCTG
CCTCCCCTGCCTATCGTCAGGTTTGATCCCCCTCCACGACGTCCACCCCGGTCCCTTCAACCACAACTACTGAAGCCG
AGGAGCTCTATTCTCTTCAGGGGCTCCACCCTCCGTCTATCCTCGCGCCGATGGCAGCCGAGCCCCCAGCAGAGAGTC
TCGAGCAACTAAAATTCTCACAGATCCCTCTTTCAGATGTGGAGGAACATTCAGTTAAAGAGTCTCAGGCAGCACCTG
AAAAGCCGGAGCCACAGCCCACCGCTACCAAGACTGGCAAACCTCTGACCTCCACAGAGCTGCTGTGTGGAGGGGCTG
CTTTAGTAGCCGTTGTTGGAGTAGTGGCGTACGGTGCTGTGGCCTACTGCAGAAAGTAGAAAAGTCTCCCCTTCAGCC
CTGAATCACCCTTTAAACACAGGTCTTCTCTGTTTTTAAATCTATTCCAAACGAATTATTCCACTTCACTCTCTGAAT
TCTGGATGTTACTCTCTGACACTCTTTGTATTGTAGTTACTATCAGACACATCAATTTGATTGTTTGGATTCGTTTTT
GTGCTACCTCAGCTCACAGCAGAGCTCATGGCCTTTGTTTTTTCTCTCTTTTCAACCGATCTCTGAGTAAACGTAGCC
AGTGAAATGTAAATCGAGAATGTAAAATCAATGTAAGGTACAATTTTAAAGACTCCTGAAAACTAAGAAAAAAAAAAA
AAACCGAACCTACCCTA

>Sequ20760EST2
ACAGACATCTCTCTGTCAGCCCGGGTGGTGGTGGTGGTGGTGTGGTAGTATTTCGTAATCGTAATCGGCCGTGGTGTC
CCTGCTTCTCTCAGCCGGCAAGAATGGCAGCGCTCAGGACTCTCACGAAACTGTGCCAACACTCCCAGCATCAAGTCT
GTAAACTGCACACGTCCGCCCTGAGACAGGAGGCGGTGGTCATCTCAGGAAAGAACTAGCACGGCAGATTCGGGAGGA
GGCCCGGGCCGACGTGGAGAAATGGGTCTTAGCCGGCCACAGGAGACCCCATCTGAGTGTGATTCTCGTAGGAGACAA
CCCAGCCAGCCACTCCTACGTCCTGAACAAGACACGCGCTGCAGCGGATGTCGGAATCTCTAGTGAGACGATTCTCAA
GCATTCAGACATCACCGAGGAGGAGTTATTGGACCTGATCTACAAACTCAACACAGACCATCGTGTGGACGGCCTGCT
GGTCCAACTGCCTCTGCCAGACCACATCGATGAGCGCACAGTCTGTAATGCAGTTTCCCCTACCAAGGACGTGGACGG
TTTCCATGTAGTCAATGTGGGTCGCATGTGCCTGGATCAGTCCACCATGCTCCCCGCCACTCCCTGGGGAGTCTGGGA
AATGATTAAACGCACAGGTATTCCTACTCTTGGGAAGAATGTCGTGGTTGCAGGACGCTCCAAGAATGTGGGCATGCC

CATCGCCATGTTACTGCATACAGACGGCCGTCACGAGAGGCCCGGAGGTGATGCCACGGTCACCATTTCTCACCGTTA
CACTCCAAAGGAGCAACTTTGCCAACACACTAAAATCGCTGATATCATCGTGGCTGCTGCAGGGATTCCAAACCTCAT
CACTGCGGACATGATCAAAGAGGGCGCAGCAGTGATTGATGTTGGAATAAACAGAGTGCAGGACCCGGTCAGTGGAAA
GAACAGACTGGTTGGAGACGTGGATTTTGAAGGCGTGAGACAGAAGGCAGGCTTCATCACTCCAGTGCCTGGAGGTGT
GGGACCCATGACTGTGGCGATGCTCATGAAGAACACTATCAAAGCAGCTAAGAACGTTCTGCTGTATCCGCCAGAGAG
GATCCGCATGGCGGCTGCGTCTTAATGGCCTGGCGCAAATCCCAGCAACAGTGACACATTCCACCCACTCCAACACTA
CCACCCACCCCCTCATTTTTTACTCGGAGCAACCTGATGATCTCCCCTTGCACATGGACTGGGAAGATGATAGCTTTA
AGCCTCTTTTGCTGCTGACATCTTTAACCTTTCCTAACTTCAAGTCCAGCCATGTTTAGACTGTTACATTCACTCATG
CTGCTGCATGTTTTATGTAACAGGGTTCTCAGTATTTATCATTTGCCATGTATTTCTGTGGGATTTTGGGAGTTTTAA
AGAATCTTGAGAGGTTGTTTTATTTATTATTACAATAGGATAATATTTAAACATGAATGGAGCCATGTTACAGTGTTT
AACTCAAAGCTGGTGAGCAGTTAAAGCAAAACAACTGAGGCCTGTTTTTTGGGTTTTTTTCAGATAGTTTAGCCTTAT
GTGTCAAGAGTGAAAAGGGCTAACATCATCAGGAACAAGTATGTTTATCAATATACAAGCTTTAAAGATTCACAATTG
CCTTTGTAAAAACTTTGAAATAAATATTACAAATATGTTCTAAAAAAAAAAAAAAAAAAAAACTAAGAGA

>Sequ20808EST2, Sequ20808SNP2
CTCATGTTGTTTGTAAAAGTAGCGTTTACTTTCCTCAGGACCGAGAGTGTTTTAGCCCAGAAAAGAATTAAATTCATA
TCTGTACTAAACCACGTAAACAGAAACTCATCCGTTTAGGCTGAATTATACAAAATGGACCTCTAAAAAGAAGCGAAC
GCCTAATGTACAAAATGACAGCTGGGTTGTCGTTGCTGCAGACTCTGTCATCGACACACAGAAGCCCGACAGTGATCC
AGCTTTTGTGAGACTAAGGAATCCATCTACAGATGCAGCATCTCTGTATATGCTGGGTAGTGGTGATGTGCAGCTGTA
TGAGGTCAAAGCATTTGAAGAAGATTTCCACTCCTGGTTTGTTGGTCAGACTGTACAAAGAGATGGGAGACTTCTGTT
CATAACACCAATGGATCCTCTCTATCTCATTTTGCCCTATTTGATTACGGCTGGAAAAGAGGGGAAGTTCCAGCCTGT
GGATCAGGTTGTTATGGATGAGGAATTCCCAGTTTGCTCAAGGCTGCTGAGCTGCACACGTTCCCTGGCCGCCCTGCA
CCACATTGCCGAGGAAAAAGAGGTGGGAAGCCGGAAATTTCATCGATACAGCCAAGAGAAGACAATGAACTGGTTAAA
GAAAAAGGTGGAGAGGACAGTCGTTGCCCTCAAGAAGAGAAATATCTCAGTGGGAGAAGGAGTCAAATCCACAACATA
TGTCAGAATGAAGTCAGAATCAGACTACCATGAGGAGGACTACCTTCGCTATGCCCACGGCCTGATATCGGAGTACAT
CAGTGAAGACCTGAGCAAAGCCCTTCTCAAACACCTGCAGTTACCAGAGCTCACAAGCCCAAAGGAGACAGAGCCGCC
CTCCAAGAAGCGGAAACTGTCAGATAAACCGGTGGAGGCCGGAGAGGACTACACCAAATTCAACAGTGCAGACTTTTC
GAGGAAACCACCCAAGAAGATGACGGCTGCTCAGAAAACTCTGGCTAAGGTGGACAAGACCGGCATGAAGCCTATGTC
ATCCTTCTTCAGCCCCAAGGGCAAGGCAGAAAAGAATGAATGTTGTATAGTTTCTGGTTTGTACATGATAAATTTGTA
AATAAAGAAAAGAAACATAAGAAAAAAAAAAAAAAAAAAACCGNGAACTGCCAA

>Sequ20810EST2
ACACACTGAGCTGTCTTCTTGATCCCTGATTTCACTTGAAGTCACCTGCAACACAGTGGACATGGCATCAGTGTCGCC
CAGCTCTCGCCTCAACAAGTCCGTGCGCCAGCAGTACATGAGCCTGCCGCAGGGAGACAAGTGCCAGGTCACCTACAT
CTGGATCGACGGCACCGGAGAGGGGCTTCGCAACAAGACCCGAACCCTGGACAAAGAACCTGAAAGCATAGAAGAAAT
TCCTGAATGGAACTTCGATGGCTCGAGCACGTACCAGGCCAAAGGCTCCAACAGTGACATGTACCTTATCCCGGTCTG
CATGTTCAGGGATCCTTTCACCCTGGACCCCAACAAACTGGTCCTCTGTGAGGTCCTCAAGTACAACCGTTTACCTAC
AGAGAGTAACCATCGAGCTAGCTGCAACAAGGTGATGGAGGAAATGAAAGAGCACTGCATGTGGTTTGGTATGGAGCA
GGAGTACACACTCCTAGGGACAGATGGACATCCTTTTAGTTGGCCCATAAATGGATACCCATCACCCCAGGGCCCTTA
CTACTGTGGCGTGGGGGCAAACAATGCTTATGGACGGGACATCGTGGAGTGTCACTACAAGGCCTGCCTCTACGCAGG
GATCAAAATCTGTGGCACCAATGCTGAAGTTATGCCATCTCAGTGGGAGTTCCAGGTGGGTCCCTGTGAGGGTATTGA
GATGGGCGACCATCTGTGGGCCGCACGCTTCCTGCTGCACCGCGTGTGTGAAGATTTTGGGGTTGTTGCAACTTTGGA
CCCCAACCAATTACGGGCAACTGGAACGGTGCCGGTTGTCACACGAACGTCAGCACCAAGCAGATGAGGGAAGAAGAC
GGACTGCAATACATCGAGCAGGCCATCGAGAAACTGAGCAAAAAACACATGGAGCACATCCGTATGTATGACCCCCGT
GGAGGCCAGGACAACACCAGACGTCTCACAGGTCTCCACGAAACCTCCAGCATCACAGACTTCTCTGCTGGAGTGGCC
AACCGAGGCGCCAGCATCCGTATCCCCCGCCAGGTGGGCCAAGAGAAGAGAGGGTACTTCGAGGACCGCCGCCCTGCA
GCAAACTGTGACCCATACCTCGTGACAAAGGTCATCGCGTGCACCTGCCTGCTGGATCCAGAAGAGGATGAGGAGTGC
AAGTAGGAGAAAGGTGCAGCCTGTTGTACTGGCTATGTAGCAATGTTTAATAATATACATTCCTCTGTGCGTTCACCT
GAGGTGGACTCTTGAAAAACACAAGATAAAGAAATTCTGGCATGGTTAATGGTTATTGTGATAGCATGTTTATTGATC
TAAGTTTAGCCATATAGTATAGACTTCCATCTGCACATACAGGTAATTTTCAGTACTGTAACTGTCTTTAGAAGGAGA
CATTTAAAGTTTTGCACAGCAGCTTTGTCAGAATGGGAAATAACCGTGTGTTATGGCTATTCTGAGCTTACATGTTCA
TCATTCTGCATTAAAATATGAATGAGCTTGTTTTACAGGTGTTAGTTTGAAATAAAGACAATG

>Sequ20814SNP2
TCATCCTTGCCACTGGCTCTGCAGACAAGACTGTGGCTCTTTGGGATTTGAGAAACCTGAAACTGAAGCTGCATTCCT
TTGAGTCACACAAGGATGAGATCTTCCAGGTTCAGTGGTCGCCTCATAATGAAACTATATTGGCATCCAGTGGAACAG
ACCGTCGCCTCAATGTCTGGGATCTCAGTAAAATCGGAGAGGAGCAGTCCCCAGAAGATGCTGAGGATGGTCCTCCTG
AGCTACTGCTTATCCATGGAGGACACACAGCCAAGATCTCAGACTTCTCCTGGAACCCCAATGAGCCCTGGGTGATTT
GCTCTGTGTCTGAAGACAACATTATGCAAGTCTGGCAAATGGCTGAGAACATCTACAATGATGAAGATCCTGAGGGTG
CAACAGACCCTGAAGCCACAGCGTAACACATTTACCCACATTTGTCTTAACCTTTAACCTCATCACAAACTGCCACTT
CAGAAGATCCATGCAGCTACTTCTTCCCATCCTGTTCTACTTTACTGGTGGGACACAATATGTCACAAATCACTGTAA
ATACAGGAAAGAGAGTAAGCATGTACAGCCCTGGTCGTGTTTGGAATATTCAAAGTGAAGACCTCTCCTTTTATTCAG
TGATTTATTGCCTCAGCCATGTTATTTGCACTTCATATAAAAAGTATATTTTTGTAACCAAATTGGCTTTCCCATATA
CACTGAAATGTTTTAGCCAATGCTTTTTTCAAAAAAGCTTTTCTACAACACAAGGGGGCGATCATACTGAGAGCCCAA
GAAAAATGCTTATTCACAGTAACCTGCTGTTGTGTACACACACAGCTCCCTCCACACATGTTAAAAGTGTAGAGAATG
TCAAGTTTAATGTAAAAGGCAAATAATGAGTCAGTTTGGTCACCACTGAATTGTTCTGAACTCCGATTTCCACACCAG
CTGTTTCTGAATCTGTGTTTGAATTGTCCAGTTGTTTTCCCATGTCCAGTCACCTTAACATTACATTACTCGTCTAAA
GGAATTAAATAATTTACTAATCATTTTTGTCGTTGTGAAATACATTTTTCTATGTTTTGAGATTTTGCTATTTGTACA
GTTAAATTGTTAGTGCATTTCTTAATAAAACTGTCTTTCATTAGTCTTTG

>Sequ20816EST2
CTTGTCCCCACTGAGCTGTGACCGGTCCCCGCTTTCATGTCGACTGCGGTGCTAATTGCCGCTGTTGTCGGCGAGGGG
TCCTATACGTTATGCGCCGTTTGTTCCCCGCTCAGAAAGCCGGAAAGCTGCTCCGGTATCCAGCCGACACCATGCGGG
GAAAGACAGTCATCGTGACCGGGGCTAACTGCGGGATAGGCAAGGCCCTGGCCGCAGAGCTCCTGAAGCTCCGAGCCC
GGGTCATCATGGCCTGTCGGGACCAGCGGAGCGCCGAGGAGGCAGCTCAGGACATTAAGAAACAAGCGGGACCTGAGC
AAGGGGAGGTGGTCATCAAACACCTGGACCTCGCCTCTCTTGGATCAGTACGAAGATTTTGCGAGGAAATTAATGAGG
AGGAGTCCAAAATTGACGTGCTCATCAACAATGCAGGCATCTTCCAGTGTCCTTACACGAAGACAGAGGATGGTTTTG
AGATGCAGCTCGGTGTGAACCACCTGGGTCACTTCCTCCTCACTCACCTCCTGCTGGACCTCCTGAAGACTTCTGCTC
CCAGCCGCATCGTTGTGGTTTCCTCTAAGCTTTACAAGTATGGCCACATCAACTTTGATGACCTGAACAGTGAAAATA
ACTACAACAAGGCCTTCTGCTACAGTCAGAGCAAGTTGGCCAACCTGCTGTTTACACTCGAACTGGCTCGTCAGCTGG
AGGGCACAGAGGTCACAGTCAATGCTCTCACCCCGGGCATCGTAAGGACCAGACTAGGCAGGCATGTTCAAATCTCCC
TCCTGGCAAAGCCGCTGTTCTACCTCGCCTCACTGATCTTTTTTAAGAGTCCACTTGAGGGGGCCCAGACTCCTCTCT
ATCTGGCCTGCTCCCCAGAGGTGGAAGGAGTGTCAGGGAAGTGTTTCGCTAACTGCGAGGAGGAGGAGCTGATGGCCA
AAGCTACAGATGAGCAGGCAGCCAAGAAGCTGTGGGACATAAGCAGCAGGATGGTTGGACTCACTAACTAAGCAGAGT
CCTGTTCTGTGGAGGGTGTTTCAGGGGACTTAAGTATTCCTGGACAAGATAATTAATCCTTAGCAGCCCCACAGACTG
TACAGAGAGAGATGGCTTGTGAAAAGATTGCCACCTTTCAGATAAAATCATTAATTGACTGACATATTGTGCCAGTGA
AGTGTGTGAATTAAGTCTCTGTAAGCAAGTGCGCTGCGAGTGTATTGCACAGAGATTGTATAAAATTTGAACAATTAA
AAAACTGTGCCAACTAACAAAAAAAAAAAAACCGACGAACTA

>Sequ20837SNP2
AAGGTAGGCACTGACGTTTTCCATGGACGCACAGAAGTTTGCTGTAGCGCGAGAGGTGGCCCGCTTGCAGTCCGGAGG
ACCTGTTCTGAATGCTGCCGTGGCCCCGTTCTGCCTGGGTGGGGTTTGGGTGTACAGCGTGGTGCTGAAGCAGGTGTT
CGGACTCCACGCTGGGCCTGCGCTGTTTCGGGGAGCTGCGAACATTGTGGCGCTGGGCCTCGGTGCCGTGTCCTACTT
GCTCACCTCGGATGCTGTCAGCCAGTGGATTGACTACAGCTCAGACAAAAATGCGGCTGCACTGTCACGCGATTATGC
CAAAGGAGGGGTTGAGTTTTACGATAAGATTTTGTCCAGAAACAAGACTCTGCGCTCTCTGAGGGGCCAGAAGGGAGA
GGAGATGTATGCTCCCAGTGGGAACTTGTTTCCTGCTCACCTCCTTCAGCTGAAACATACTCCGTATACATCCAGGAG
GGAAGGGATCCTAGCTTTACTGAAAGAAGAGAAAGTTTGAAAAGAAAGGGGAGGAGGGCTGTTATGTTAAGAGTTAAA
ATCATTGTAACTGGGCCTCACATGAACGACTGTAATTATCTACTTGGGCTTGTCTCTGAATAGACTCCAGTGGCGTAG
GAAGGTCTCTGAGCTTAACATTCTTTTCAAAGAGATATGTCATCCCACATGTTCTGAATGTTATTTTGTGAATCATGA
AGTGCAGAACAATTCAAATTACAAAGAAGTCTAGTAAACCCCTGGGGTCTCAGATTTTTAGTGGCGTGTGTGACTGGT
GACTTATTTATCCAACAGATTAATTCTGGATCCAGAGTACAAGTAAATTACTGTATCATTAACATGTGTTAATACAAC
CACATGTTCCAAACAGAAAATAAAAATAAAATAGAAGCTGTCTTTTTAAGGTGTTTTTTATGTCTCTAGATATTACAA
TTACAACCTGCAAGGAATGATCTGTGTGGCAAAACACTCGGTTAAACAGGCCAAAATTCTAAATTACATTTATTACAT
CCTATTATTGTTATCTAGCCATGCATATAATTTTATCCTGAGTAATATTGTTTTCAGAAACGCATCATTTTAAATTCT
GTGACTGCCATTTTTCATATCTCAGAACCTGAAACCCTTTATTTCTCCCCCATTTTTCAAAAAAGAGAGGGAATCATC
ATAATAATCATGCATCACTGGAGATTTCCTATTGCACATCGTAGCCTCACTTCAACAGACTAGTTAGACAAAAGCATT
TTCCTTCATTTTGAAGAATTTGTGGTGGAGAATGTGAGGCGTCCTTGGGTTCAGCATCTCCAGGTGTGTTACCACTCA
ATGAAAGTCAGGAAAAACCATTATGGAGGAAAATATCTGGATTTCTCTGGAATGGACTGGTACTTAAAAAGCTGTGTA
CTGTTTTTGTTTGGTGGAAATGTTAAGCCATATTAAATGAATGAATC

>Sequ20888SNP2
CCTTTTTTTTTCCTTCTCATACCGGCAAGGACAAGTCAACCTCGCCGCTGTCCATCCCAACACAACACCTACAGTCAT
CCACGGTTCAACAATTCGGAAACATGTCTAAGGGACCAGCAGTTGGTATCGATCTTGGGACCACCTACTCCTGTGTTG
GTGTGTTCCAGCATGGCAAAGTTGAAATCATTGCCAATGACCAGGGCAATAGGACCACACCCAGCTATGTGGCCTTCA
CGGATAGTGAGAGGCTAATTGGAGATGCAGCCAAGAATCAGGTTGCCATGAACCCCACCAACACAGTTTTTGATGCCA
AAAGACTGATTGGCCGCCGGTTTGATGACACAGTTGTGCAGTCAGACATGAAGCACTGGCCATTTAATGTCATCAATG
ACAACACTCGTCCCAAGGTTCAGGTTGAGTACAAGGGTGAGTCCAAGTCCTTCTACCCAGAAGAGATCTCTTCCATGG
TGCTGACAAAGATGAAGGAGATTGCTGAAGCCTACCTCGGAAAAACTGTCAACAATGCTGTAATTACAGTGCCAGCCT
ACTTCAATGACTCCCAGCGCCAGGCCACTAAGGATGCTGGCACAATCTCTGGTCTCAATGTTTTGCGAATCATCAATG
AACCAACTGCCGCTGCCATTGCCTACGGGTTGGACAAAAAGGTTGGGTCAGAGAGGAACGTTCTCATCTTTGATCTTG
GTGGTGGCACTTTTGATGTGTCCATCTTGACCATCGAGGATGGTATCTTTGAGGTCAAGTCTACTGCTGGAGACACTC
ATCTTGGCGGGGAAGATTTCGACAACCGTATGGTCAACCACTTCATCGCAGAGTTCAAACGCAAGTACAAGAAGGACA
TCAGTGACAACAAGAGAGCTGTCCGCCGTCTGCGCACCGCTTGTGAGAGGGCAAAGCGCACACTGTCTTCCAGCACCC
AGGCCAGCATTGAAATTGACTCTCTGTATGAGGGAGTTGATTTCTACACCTCCATCACCAGGGCTCGTTTTGAGGAGC
TCAATGCAGACCTCTTCCGTGGCACTTTGGACCCCGTTGAGAAGTCTCTCCGTGATGCCAAGATGGATAAAGGGCAGA
TTCATGACATTGTGTTGGTTGGGGGCTCCACCCGTATCCCCAAGATCCAGAAGTTGCTCCAGGACTTCTTCAATGGAA
AGGAGCTCAACAAGAGTATCAATCCAGATGAAGCTGTGGCCTATGGAGCTGCTGTCCAGGCTGCCATCCTGTCTGGTG
ACAAGTCAGAAAATGTGCAAGACCTGCTGCTTCTGGATGTCACCCCTCTGTCCCTGGGTATTGAGACAGCTGGAGGTG
TCATGACTGTCTTGATCAAACGTAACACCACCATTCCTACCAAGCAGACCCAGACCTTCACCACCTACTCTGACAACC
AGCCCGGTGTGCTCATCCAGGTTTATGAGGGTGAGCGTGCTATGACCAGGGACAACAACCTGCTGGGTAAATTTGAGC
TTACAGGCATCCCTCCTGCTCCTCGTGGTGTTCCCCAGATTGAAGTGACATTTGATATTGATGCCAATGGCATTATGA
ATGTCTCTGCTGTCGACAAGAGCACTGGAAAGGAGAACAAGATCACCATCACCAACGACAAGGGTCGTCTGAGCAAGG
AGGACATTGAACGCATGGTCCAGGAAGCTGAGAAGTACAAGGCAGAGGATGACGTTCAGCGTGACAAGGTGTCTGCTA
AGAACGGCCTGGAGTCGTATGCTTTCAACATGAAGTCAACTGTGGAAGATGAGAAGCTTGCTGGCAAGATCGCTGATG
ATGACAAGCAGAAGATCTTGGACAAGTGTAATGAGGTCATCAGCTGGCTTGACAAGAACCAGACTGCCGAGAGGGATG
AGTATGAGCATCAACAGAAGGAGCTGGAGAAGGTGTGCAACCCCATCATCACCAAACTGTACCAGAGTGCTGGTGGCA
TGCCTGGCGGTATGCCAGAGGGCATGCCTGGTGGCTTCCCTGGAGCTGGTGGTGCTGCTCCTGGTGGTGGATCCTCTG
GACCAACTATTGAGGAGGTTGACTAAACATTTCATCTAATCATTTCAGCTACCTCTGAGATGTTTGTTTTGTTTTTTT
AAGAAGGTAACCCTCTTATAGCAGTTACAACAAGCTAAAGAGTGCAATTTAACAAAGTAAAATAGGGTTCAGGGATCA
TATTTGCATCATTTGTGACAACTGGGAACAGCTTGTAGTTGGTAAATTGATGGTTCGTTCACATGTCAAGTGTTTTGT

ATTCTGAGATGTCACTGCCTTGACAATAAAAGTCTT

>Sequ20924EST2
GTTTGGCGGTTGCATTGAGTACCTCTGAAAAGCATCGTCTTAAAACATGTCAGAGAAAGGAGAAGTTGACTTAACTGG
TGCCAAGCAGAACACGGGTGTGTGGCTTGTAAAGGTGCCCAAATATCTCTCTCAGCAATGGGCAAAAGCAACTGGCAG
AGGAGAGGTCGGGAAACTCCGAATCTGCAAGAAAGGGAACCAAGGAAAACCAGAGGTCAGTTTCACTTTGAATGAAGA
GCTGACTGTGATTCAGGGTATAGAAGACAAAACGGTGTCTGCGCCCCGAGACCACCCGTTCACCATGCAGTCAGTGGG
AGGTCAGATGTTGGCAGTCTTCACAGAAAGTTCATCAGGCCAGTCAGAAGAGAGATCTGATGGCAGCAGCTCAGGTTC
GGGGCGGGGCAGGTCCAAATAAAATAGCCTTGGAGGGAGTGGTGGTACAGAGAGCAGAGTGCAGACCTGCTGTTAGTG
AAAGCTATATGAGACTAAAGAGGTTACAAATAGAAGAGTCCTCTAAGCCAGCCAGGCTGTCACAACAGTTGGATAAAG
CTGTCACCAGCAACTACAAACCTGTGGCCAACCATGCTTACAATCTTGAGTATGAGCGGAAAAAGAAGGAGGAGGGCA
AGAGAGCAAGAGCTGACAAACAGCAGGTGTTGGACATGCTGTTTTCTGCTTTTGAGAAGCACCAGTACTACAACATCA
AAGACCTGGTGGATATCACGAAACAGCCTGTGAGTTACTTGAAGGAAATCTTGCGAGATATTGGCATCTACAATGTGA
AGGGAACACACAAAAATACCTGGGAGCTCAAGCCAGAGTACCGACATTACCAAGGCGAGGAAAAGACTGACGAGTAGT
TTCTGACCCTTTGAAAGGTCGATGCACCACCTCTTCATTTGACAGAGGTCACCCCCAGGTCGGGCCTTGGAGCTCACT
CCTGATTTCCTTACAATCAGGTCACAAAACACACCAGATGATGAGGTCAAGCAAAGATTGAATTTTACAAGGAGAGGT
TGTTGTATCTTCTTGTTATTTTGTAGGAGACAAAAAGGAGGGACAGTGGGAAACAACTTGAGGTTTATGCTCAAGTGC
ACACCATCATAGCTCCTGTAAGGCCTCAAGGTAAAAGCCCCCTGGTATTTGACTTAGTTGTTTTGTCTGTCAGAGGCA
TAGGTTGTAGTATTTAATGAAGCAAAAGATGTGTTTATGAACTCTCGTTCTTGGCATTGAATTATTTTTCTAATGTTT
TGACAAGTATATCCAGAGGGAAGGAGCTTAATGTTTAATGGATAAATGTTAACAAAGGAAGGATGTGACACTGGTAAT
GGCCCCTCACCACTGAACTGATGTGTTGAGGTTTCTTTTTCTACATGTGTAATGTTTTTAGAAATACAAATTAACTGG
TTTCCCCACCATGACTTGCAAAGACAAATTCTGCCTGAACTTGCATTGGCAAAATTTTAAAATCTACACCATTGCTGT
TGTGTGTTATGTTAACTGTTAATTTTAAAGTGTTTTACATTTGATTGACTTATGCAATAAATGTTTCAATAACAAAAA
AAAAAACCGAAACCTCCCTA

>Sequ20931EST2
TACTTAATGTTTGGGTATTAGCTGCGTAAAGGCAGGGCAGGGCTTGGCCAGATCATAACTTGGGATTTTCACTGCTTT
CTTTCTTTTCGTTGCCGGGTCCAGGACAGGGGGGAGACTACATGGATTGGTGGACTTCGTTGGCAGCGTGTATTGCCC
TGCTGTGGATATTCTGCTTCATAGACGGCAACGCCGTGCTTGAGCTGCGGCTGCTCTTCCATGCTCTGACCAAAGCAG
CTTGTCACCTGTACCTGGCTCTGCTCCCGCTGCGCCGAGCCGCCACGCACTTCGCCGACTGTGCAAGATACCTTTTCA
ACCATGCCCAGCAGACCCCTCCCCTCACGGCTACCCCCACCAAGGCCAACCCCTGGCCAGGAAAGGCACGCTGCACTT
CCTGAGGGCATGTGCGTGGTGTGCGAGACGGTGCCTAACCTGATGGGCGCAGCGCTCAGTGTGGGCGCCGCCTTCTGC
CACATGCTGCAGGGAGGCTTCTACGTCCTGGCGGCGACGCTGCGCACCATCCAAGTCAACCTGTTCTCCCAAATGAAC
GGCGAGAAGGAGAGATGGGACAAAGAGCGACACAGAGCCAGAGAGAGCGAGAAAAGTTAAATTCTAAAGTGTTGGAAT
ACATCTCGGTGTTTTGTCAAGACCCTGTCAGCGCTTTACGCATCAAGGTCGACGTGCCACCTGTGTATAGATAATCTT
TGTCCTTGTAAAAAAATTCCTGGTTAATTGGCACATAAGAGGCCTTCTTCTAATTGCTGAGGAGTTACAGGTGCAGGC
AGAGTGTTGATTGGTTTGCAGAGAGGACAGGAGGTTGAACTTTGACCTCTGGTGTTGTTGAGGGAATAGTACCAACAG
AATCAGAAAAGAAATGATATTCAAGTGTGAAAAAGATAAAACAATCTATGGTGCATTTTGTTACATTTGTTTTACATT
TATTGAGATGTAAAGAATTTAAAATAAATTTCTCAAAACGAAAAAAAAAAAAAAAAAACTACGTA

>Sequ20932EST2
GGGAAAGATGAGGGGTTTAGCACCAAGCAAGAAGATTGCTGCTGTGCAGCAGAAAAATGTTGATCTGAAAGTGAAAAA
GGCAAAACAGAAGACCCCAGGGCCGGGTGAGGGTACCAGCAGTGGGGAAACGCCACAGGGACCACGGAAAAAGAGGGC
TCGGGTCGATCCCACTGTGGAGAGTGAGGAGATGTTCACCAACCGTGTGGAGGTGAAGGTGAAGATCCCCGAGGAGCT
GAAGCCCTGGCTGGTAGACGACTGGGACCTCATCACCCGACAGAAACAGTTGTTTCACCTGCCTGCTAAGAAGAATAT
AGAGACTGTCCTGGAGGACTATGCAAACTATAAGAAATCAAAAGGAAACTCGGACAATAAGGAGTATGCAGTAAACGA
AGTGGTGGCAGGGATTCGGGAGTACTTCAACGTCATGCTGGGTACGCAGCTGCTTTACAAGTTCGAGAGGCCGCAGTA
TGCTGAGGTCCTGACTGAACACCCAGACATGCCGATGTCTCAAGTTTATGGAGCCCCGCACTTGCTGCGCCTATTTGT
TCGTATTGGAGCCATGCTGGCCTATACTCCACTGGACGAGAAGAGTCTGGCTTTGCTGCTCAGTTACCTCCAAGATTT
CCTCAAGTACCTGGTAAAAAATTCATCCACCCTCTTCAGTGCCAGTGACTACGAGGTTGCACCCCCAGAGTATCACCG
CAAGGCTGTGTAAGAGCTCTCAGTACACCGTGCTCCTATCAAGGCACGTCACTCCCCCTCTCCCAGCCCACATGTAAA
ATAACCATTACTACCCTTCTGAGTGCAGACTTCGATGAGATTTTTCACTTGAACTGATGCGCTTCTACCACAGTGGTC
TCAGGAGCTTGGCAGAGTTGAGTAGTCAAAAATTTGAGGAAACCTTTTGTAGCTCACTTTTCTCTCCCTTTGCCTTTT
TTCTCTCACTTATTTGAAAAGACACAAGAAAAAAAGTTTCTGTTGAACAGTGACTGTTTCCACAAGCAGCTCTTTGGT
GTTTTTGTGCATTCCATCTGATAGGTATTTGTTACTGTTGCATGTCTGTATTTATTAGTGTTCCTTCTGCCACATGAG
GTGCAATTTAATGCTGGACCACATGGTCAGCACTCAGCAGTTAATGTTTTTGGATCTGTATCTTGTGTATTTGTATCA
AATTTAGTAAAAAAAAAAAAAGTTATACACAGACCAAATAAAAAAAAAAAAACCGAACCTACC

>Sequ20975EST2
CCGGAAGTAGCCAGGGTGGGATTTAGAACAGGACGAAAACGAAGGCATGGGTGTGCATATGTTGTGAGACGCCATGTC
CTCCTAGTTCATCGGTAGCTTTTATATTTATGTGTAGGTGTGTAGACTAAACAGCGCAATGGCGGCGTGGACCAGCCG
GTTTCTTCTTCAGCGTGGAACTCGACTCGTTTCTCTCCGAGCAGTAGCTGAAAGCAGTCCTGTCCAGTTGGTGGGTTG
TATTAGGACAGTAAAAACCACAACATGGTTTGAAGAACACCTCACAAAGGACAACCAGGAGTACATGAGGAAGAGCGT
GGCAGAGGAATACAGAAGACAGATAGCTGATAAGCTCAACCCACTCAAAGATGAGCCCTGGCAACGGCAGGAATGGTC
AGAGGGGAGCCGAAGAGTGGGATTAGTTGCTGTGAAATTGGGGATGGCTCCTATCTGGACGAAAACAGGAGAAAGACA
TGTTGTTACCATGTTACAGGTGCAGGACTGCCATGTAATAAAGCACTTGTCCAAAGAAGAATATGATGGACACACAGA
CGCTCTCATTGTAGGAGGGAAAAACGCATCACCATTCCATAGGTCTGAAAATGAATTGGAGATGTTCAGGAATGCTGG
AGTGCCCCCGAAACAGAAGGTTGTCACCTTTAGAGTCTCTGACAACGCTCTCATTAAGCCAGGCACTCCTCTGTATGC
AGCACATTTCCGTCCAGGCCAATATGTGGACGTCACAGCCAAATCCATTGGTAAAGGTTTTCAAGGAGTAATGAAGCG
ATGGGGGTTCAAGGGTCAGCCGGCCAGCCACGGCCAAACCAAAACTCACCGCAGACCAGGAGCTTCTGGACCTGGAGG

GGATCCAGCCAAAGTTTTCAAAGGGAAGAAGATGCCCGGCAGAATGGGGAACACCTACATCACAGCTTATGGATTGAA
GATATGGAGGGTCAATACCAAGTATAATGTGCTGTATGTTAACGGCTCCATCCCTGGCCACAGGAACTGCTTACTGAA
GGTAAGAGATACTGTGCTGCCAACCAGGAGCTCCACACTGCTCAACCCTCCTTTCCCCACCTACTTTACCGAAGAGGA
GGCTGACCTTGACGAAGACCTGTACGATGATGACTTCTTCATTCACACAGAACCATCATTAACACTGACCTGACGACG
CAACACACACACATTTCTCATTCATACCTAAACCTGCAAACTGTATCTGGATCCTGTAATAAAGAGTAATTTACTTAA
CGAAAAAAAAAAAAAAAAACCGCCTACCGTACCGACCTACTCGTCG

>Sequ20976EST2
AGTTTCGGGCGGGGCTATAAGGCTTGTTTTCCAGTCTTACGGACTCGACGATCTCCATTTTGTGTGAGCTGTGAAAGC
CGAGCAAGCGTCGTCCCCGCAAAAGGCAAAAACCAGGAAAGACATAAAAGTCAAAGTTAGCCGACGAACCGCTCGCTA
ACCAAGTTATTATCCGAAAGGAAAGCAAATCTTATCGTATGTCCGCTATCCAGAACCTCCAAACTTTTGACCCCTTTG
CTGATGCAACTAAGGGTGATGACCGCCTCCCAGCCGGGACAGAGGACTACATCCACATAAGAATCCAACAGCGGAACG
GCAGGAAGACCCTCACCACTGTCCAGGGCATCTCCGCCGACTATGACAAGAAGAAGCTAGTCAAGGCCTTCAAGAAGA
AGTTTGCCTGCAATGGGACAGTGATTGAGCACCCAGAGTATGGTGAAGTGATCCAGCTACAGGGAGACCAGCGCAAGA
ATATCTGCCAGTTCCTCATTGAGATTGACTTGGCCAAGGAGGAGCAGCTGAAAGTCCACGGCTTCTAGAAGCTGCAAA
TCAGCCCTCTCCCATCTCTCCTGGAAGGCCATTACACCCCCCTCCCCGTCCCCTCCTCCTATGTGCTGCTGTTGCTCT
TCCTCCCCCTTTCCCTCCTTGCAGCCCTACCAGCTCCTATTAGCTAACACGTGAATTACCTCTGACAAACATGGGACT
CTGTAACACCACTGCCCCCCTACAACACCAGGGGGCAGGCTCGCTCTCACAAAGACACACCACTGCAGCTCCACACAA
CATCAGGGATGAGTGGTGATGACCCTCCCACACACACTTCCTCCTTCCTTTCCCCCTTGTTTATTTCCTTTTTAATTT
TTTCTTTTTAGGTCTTGTGCCACAGTAAGGAGGCACCGTTTCATGTAACTTTTTGTTTGTACTTCAAGGTGTTTCAAT
AAAACGATGAGTCTCCATATGCTGGAGTGGAGTGGTGTCCTGGGGAATGGGGTGGGGGCTGGATGTGTACAGGAGGGT
CATACATTTTCTAATGGTGTTTACTGATGAAGTGGGTTAGAAGAACAAGGTCTTTAGGTGGGAGGATGGGGGAAAGGG
GATTGCCAACATAAGTAATTACCCTCTTTTTCAGTCTTTCAACATCCCACTGGCGTTTACATGTTCAGTTGCCTGCTC
TCAGTTTACAGAGCCAAGTTTGTAAAACGCTGACTGTCTCCACCACCGCTCCCCGGAAAATGTGTTTTTTGCTTTTAT
TTAATGGCACATTTTTTCTAATTGCTTTTTATTTTATTTTAATTTTTTCTTCTTTTGCTGCCCATGTGAGTTTTGAAA
CTAAATGGGTGTTCTCTGGTGGTCTTATTTTGGTCAAAGCGAGGCATGTGATATGAAAGGCTGAGTCAGTTCACTATG
TGATCAGATGAACCATCTAGAGTGGGAAATGAAATCAAATGCTGGTAATCTTTGCCTCTAGTGTAGATCGATGGCACT
AAGCGAGTATGATTTCTAGGTGGAAAAATTAAGAACATAATTATTTACCGGTAAATGAAGTTAGTTTCCTACTCTGGC
ATTTGACCAGCTTTCTGGTTTGGTCTAAAAACAAATCTGGTACCAAACCTGTGGTTATACACACTTGAATCTTTAATT
TCCTATAATGCAATAATTTATAAATACAAGTAAAGATGTAGTAATGCTGAAATAATACAGTAAAAAATAAAGCCATGG
CCAGGTCACACTGTGCTCCTGTGACTGGAGGTGAGAGAGCAAGTCTGTTTTGCAAAATCCACATTTGGTTCAATAAAA
TTTCTGCAGTTGATGAACCAAAAAAAAAAAACCGCCTACCCT

>Sequ20985SNP3
GAAGATGCGGTGTTGCGCTCCATCCGTTCAGCCCCCTGTTTCTCTGTACCCAGCCGTTTGCTCCAGCAGCCACTCTCC
CCTCTGGCCAGTTGTGTCCCTGCGTCCACCAGTCACCCCACCACCCTTCCCCTCCTGCCGCACTCCTGGAGCACTGGG
AGCCTGTGTGCCCCCCCGGTGCTGTCGTCCACGCCACTTCACCTCCATCCCCACAGCACAGAGGGGGGAACCTGCCAA
TGGACTGTGGGGATTGGATAGTTATCCTGAGTCATCACAGGCCAAAATGAGGAATGTATTTCACAGTGAATGTTTTTT
TTTTTACCAGAGAAGGTCAGAGTGTGTTAGGTTTGTACATATTGTGGAGCATGAATAGAGTGAATCGGTTTTATACTT
GGTCAAGTATGTTGATATATTGTGATTTTTTTATAATAAGTCTGAAAGAGCTGTGGATACAGGGTCTGACAATAATGT
GTACAGAATGAGGGATGGAGTCTTTTCTATTATATATTTCTGTCTTTTTCTTATAAGCTTCCCTGTAATATAAAATAT
GCTATGGGTCAGAAAATGATCTAGGTGCCTTTGAGTGGGTTGAGAATCTCAGGTAGTTACTGGTTCCTCTTTCCTCTG
ATGTGGGATTAATCATATAAAAAATGTTGATCATAATTTTGTTTCCTGTAAAATGTATTGCTGAGTTTACACCACTGA
AACAGTATCTTATTTATCCTCCAATAATACAATTGCTTAGGTATAATGGTTCAGTGCTGTCTGTAATGTGAAAGGTAT
AAATGAGGGACTACAGTTGGGAAGAAAAAGAAGTGTGAACCATACAACTACCAGAGTGAATTATGATGCACTGTGAGG
AAGATGTTCTGTGTGACTGTTAAACAAAAAAT

>Sequ21009EST2
AGCAACAATGAAAACCTCCAAGCAAACTACATACTCTATGCGCTCCTCCACCAGTAGCAGGGCTCCTGCTATATCAAT
CTCCCGTACCTCTTTTCCTGTCTACAAGGCCCCTTCCATCCACGGTGGGGCCGGTGGGGACCGCATCAGCATCTCCTC
CAGCGTCCGCAGCGGTCTGGGATCTGGGATGGGAGCTGGGATGGGAATGGGCTCCATGAGAGGTGGGGGCTTCTCCAG
CAACATCCAGGTGAGCTCCAGCGGGAACAGCGCCGACATCATGGGCAATGAGAAGTTCGCCATGCAGAACCTGAATGA
CCGCCTGGCCAGCTACCTGGAGACGGTGAGGAACCTGGAGCAGGCCAACCACAAGCTGGAGATTAAGATCAAGGAGGC
CCTGGAGAAGAGTGGACCCGACTTCAGAGACTACAGCAAGTACCAGGCCATCCTGGACGACCTGAGGAGGAAGGTGTT
TTGATGCCACCACCGACAATGCCCGCCTGGTTCTCAACATCGACAACGCTCGCCTGGCAGCCGATGACTTCAGAGTGA
AATTCGAGTCTGAGCTGGCCATCCGCCAGTCTGTGGAGGCCGACATCGTCGGTCTGAGGAAGCTCATCGACGACACCA
ACATGGGCCGCATGAATCTGGAGAGCGAGATCGAATCCCTGAAGGAGGAACTCATCCACCTCAAGAAGAACCATGAAA
ATGAAGTAATGGAGCTGCGTAACCAGATTGCCCAGTCAGGAGTCCACGTGGATGTTGACGCTCCCAAGGGACAAGACC
TGGCTCAGATCATGGCAGAAATCAGGGCCAAGTATGAGAAAATGGCACTGAAGAACCAGGAAGAACTGAAAGCATGGC
ACGAAACTCAGATAACAGAAGTGCAGACCCAGGTCAGCCAGAACACAGAGGCCCTGAAGGGCGCCCAGACAGAGGTGA
ATGACCTGCGCAGACAGATCCAAACCCTGGAGATCGACCTGGAGTCACAGAGGAGCCTGAAAGTCTCTCTGGAGGGCA
CGCTGAGGGACACGGAGATGCGTTACAACATGGAGATCGAGTCTCTCAACACCGTCATCCTGAGTCTGGAGGCGGAGC
TCACACAGCTGCGTAACAACATCCAGCTGCAGTCGCAGGAGTACGAAGCCCTGCTCAACATGAAGATGAAGCTGGAGG
CCGAGATCGCAACATACAGACAGCTCCTGGACGGCGGAGACTTCAAGCTCCAGGACGCTTTGGAAGACCAGAAAACAG
TGAAGACCAAAGTGATGACTGTCACACAGACCCTGGTGGATGGAAAGGTGGTTTCCTCCAGCACAAAAACCAAGGACC
TTTGAACACCAGAAGCAACCACCTGCCACCTCCCAACCATCACTTTCCCTGCATCATAACACTAACAAACGATTGGCC
AAAATTTTATTTCCAATGGGTTTTTCTGAGCTCTTAGTCGTACAGCACTAACCCCCTCTGAGCTGTCGCTGCCATTAG
TTTGACTTCCTGGGTATACAGCTCTTCCACAGGCTGATAATAAAAACTGGATCAGAGGAAAACTAAATCATCTTTTAT
TTATTTCATCATGGTGTTATTTATGTGTTTTTGTATTGACCGGCAGATGTTCAGAATTCACAATTGGAGAACTCCAAG
CTTTTTTAGAGAAAAAACAGATACAAGACATCGAATATTTCAAAGTAATAAATAAAGAGTCTTAATTAAATGCAAAAC

ATTTTTTTTTATACTTCATCATCTTTGGTTTTCTTTCTGTCCAGTTAAGTCTGCCCTGTTGCGCTGTCATGTTCAAAC
ATTTCAATAAAGTCCATTTGAAGG

>Sequ21019EST2
TTGTCGGCGCGTTTCTGTGCGGTGAGGCGGTGCCTTCCCTGCCTGCTCCAGACTGCGGCGGGTAGAGCTCTTCGGTAC
AGAAATGGCGGAGGGGGAGTTGGACGTCGACTCGCTGATTTCCAGGCTTTTAGAGGTGCGAGGATGTCGTCCAGGGAA
GATTGTCCAGATGACGGAGGCTGAGGTGCGCGGCCTCTGCATCAAGTCCCGGGAGATCTTCCTCAGTCAGCCAATCCT
CCTGGAGCTGGAGGCTCCACTCAAAATCTGTGGTGACATCCACGGACAATACACAGACTTGCTGAGGCTCTTTGAGTA
TGGCGGCTTCCCCCCAGAGGCCAACTACCTGTTCCTGGGCGACTACGTGGACAGAGGGAAGCAGTCTCTGGAGACCAT
CTGTCTGCTGTTGGCGTACAAGATCAAATACCCCGAGAACTTCTTCCTGCTCAGGGGCAACCACGAGTGTGCCTCCAT
CAACCGCATCTATGGCTTCTATGACGAGTGTAAACGCAGATTCAACATCAAGCTGTGGAAGACGTTCACCGACTGCTT
CAACTGCCTGCCCATCGCTGCCATAGTGGACGAGAAGATCTTCTGCTGTCACGGAGGTCTCTCTCCTGACCTACAGTC
TATGGAGCAGATCAGACGCATCATGAGACCCACAGATGTGCCTGACACAGGCCTCCTGTGTGATCTGCTGTGGTCTGA
CCCAGACAAGGACGTCCAGGGCTGGGGAGAGAACGACCGTGGCGTCTCCTTCACCTTTGGAGCTGATGTGGTCAGCAA
GTTCCTGAACCGCCACGACCTGGACCTCATCTGCAGAGCCCACCAGGTGGTAGAAGATGGTTACGAGTTCTTTGCCAA
GCGGCAGCTGGTGACTCTGTTCTCGGCTCCAAACTACTGTGGAGAGTTCGACAATGCAGGCGGCATGATGAGCGTGGA
TGAAACCCTGATGTGCTCCTTCCAGATCCTGAAGCCATCTGAGAAGAAAGCCAAGTACCAGTATGGAGGGATGAACTC
TGGTCGGCCCGTCACTCCTCCCCGCACAGCCCAACCTCCAAAGAAACGATGAAGGACGGAGGGAGGAAGAGACAGAAC
GAGAGAGAGATGGTTGTCAACAATTGTCTCCCGTTGGCTTTTCACAACATAAGTAAAAGCAACAGTTGGCAGAGGAGA
AAACAGATGAACACAACTGTTTTAAAAGACACACCCACCCAAACCTGTCTTTCATTAAAATTTTCTTTCTTTATCTCC
CTCTGAACTGCTGTCACCGCTCTTCTTTTTCATCAATGCTTTTTTTTTTCTCTGCATCTGTGTAACATTTTGAAAAAC
AAGCGTTAAAAGAAAAATGAAAAACATGAGGGTATCATTCTGTAGCATATTGGAGTGTTTCTTTTTTGTGTCTTTTTG
TCCTTTGCCTTTTATTTTTGAAAACAGAATGACAGCTAGTTTAGTTTACCACATTTCCTTACTTCCCTGCCTGTACAG
ACACAACAGGTATTAATGACGACTGCTGGGTTTTCAATAAATAATACAGGGAATAAAATACCAAAAAAAAAAAAAAAA
AACTAGAGAC

>Sequ21022SNP2
CCTGGAGTCCTCGCCTGCACGTTTGACACCTGAAAAACATATCGCCGCCACCGCCGTCATGTCTCTCGGAAAAGCTGC
CCGGAGTTTGGTGAATCCGGTCCGGGGGGTCCTGCGGCCTCCGGCCTCGGCGTCGAAGCGGACTTACGCCGCCGGTGC
CGAAGCCAGGGCGGTTCTGGAGAACGAGCTCGACGGTATCCGAGCCGCGGGGACGTGGAAGGCGGAGAGGATCATCAC
GTCCAAGCAGGGTCCTCAAATCAACGTGGATGGCAGTCGTGGCAGCATAATGAATTTCTGTGCCAACAACTACCTCGG
ACTGTCCAGTCATCCAGAGGTGGTGCAGGCAGGGATTGATGCTCTAAAGTCGTACGGAGCCGGATTGAGCTCAGTCAG
ATTCATCTGCGGGACACAGGATCTGCACAAAAACCTGGAGCAGAAGCTCGCAGAGTTCCACGAGAGGGAAGACTGCAT
CCTCTACGCCAGCTGTTTTGACGCCAACGCCGGGCTGTTTGAGGTTCTGTTGGGTCCGGATGACGCGGTGCTGTCTGA
CGAGCTGAACCATGCCTCCATCATCGATGGGATCCGTCTGTGTCGGGCGAAGAGGCTGCGCTACAAACACATGAACCT
CGGTGACCTGGAGAACAAGCTCAAAGAGGCGCAGTCGTCTCGTATGCGCCTGGTTGTGACGGATGGAGTCTTCTCCAT
GGACGGAGACGTGGCTCCTTTACAGGGAATCTGTGAGCTGGCGGAACAGTATGGAGCCATGGTGTTTATAGATGAATG
TCACGCCACCGGCTTTCTGGGGCCCCGGGGCAGAGGGACGGACGAGCTCCTGGGAGTGATGGACAGAGTTCACATTGT
AAACTCCACCCTGGGGAAAGCACTGGGAGGAGCAGCTGGTGGCTACACGGTCGGCCCCAAGCCTCTCATCGACCTGCT
GAGGCAGCGTTCACGGCCCTATCTGTTCTCCAACTCCCTCCCCCCTCCCGTGGTGGGCTGTGCCACCCGGGCCGTGGA
GCTGCTGCTCGCCTCCAACGAGATCGCGCAGAGCATGACGGCCAAAACCATGAGGTTCAGGAACAACATGACGCAGGC
TGGTTTCACCATCTCAGGCTCAGCTCACCCCATCTGTCCCGTGATGCTGGGCGACGCGCGGCTGGCCTCGCTGATGGC
TGACGACATGCTGAAGCTCGGAGTGTACGTGATTGGATTCTCATACCCGGTCGTACCGAAGGGAAAAGCCAGAATCCG
CGTTCAGATCTCAGCGGCGCACACGGACGAAGACATCGACCGCTGCGTCGACGCGTTCGTCCAGACGGGCAGAAAACA
CGGAGTCATCTCCTGAGCGGGAACAACGGTCCGCAGAATGACTGAACAACCTTGAAGTGCTCGCTCTTGGAGATCATG
CTGCCTGTTACTAAAGACCTATGAATGATTTTGATGGATATACTAGTTACATTTTTAGGTCATCGGAGATTAATGTTA
TTTTTGTGTTTGCTTGGTTGAATAAAGCACTTTTGCTAACAGGTGTAGGTGTGTATTTTGAAAAAAAGGGCTGTTTAT
TCCATATTTCCAACACTAATTTCATGAGAAAAAGTCATATACTGTGTCTCAACATAACATCTCCTCAGTCAAAGAGCT
GCTTTGCCTTTTTCCTCAACAGATGCAACAGCAGATCTGCAAATTCTGGATAATAAAGAAAAAAAAAAAAAAACCGAAC
TACCCTAAT

>Sequ21025EST2
GAAGCGAAAGTGAAAGACCAGGTGGAGCAAAAGAGGAATCCAATGACCAGGGAGTGCAAAATGTCTGTGGGCTGGATG
AGAGGCACTTGACCCAAATCAATACTTCAAGATTCGCTCACAGGCCATCCAGGACCTGAAGGGCACAGCCGGAGGACC
CGTACCCACACAAGTACCATGTAGACTTGTCGCTCATAGAGTTCATTGAGAAATTCAATCATCTACAGCCTGGAGACC
AGCTGACAGATGTTGTTCTCAATGTGTCAGGTCGCGTCCATGCCAAGAGGGCTTCTGGTGCCAAGCTGCTCTTCTATG
ACCTGCGGGGTGAAGGCGTTAAGCTGCAAGTTATGGCGAACTCAAGGAGCTACAAGTCTGAGGAAGACTTTGTGGACC
AATCAATAACAAACTGCGCCGTGGTGACATCATCGGTGTCCGCGGTAACCCAGGGAAGACTAAAAAAGGGGAGTTGAG
CATCATTCCTATTGAGATGACCCTGCTGTCACCTTGTTTGCACATGCTGCCCCATCTCCACTTTGGCCTCAAAGACAA
GGAAACACGATTCCGCCAGCGCTACTTGGATCTGATTCTCAATGACTACGTGAGGCAAAAGTTTATAACACGCGCCAA
AATCATCACCTACCTCCGCAGCTTCCTGGACCAGCTGGGGATTTTTGGAGATCGAGACGCCAATGATGAACATTATTC
CTGGTGGAGCAGTGGCCCGTCCATTTGTTACTTACCATAATGAGCTGGATATGAACCTGTTCATGAGGATCGCCCCTG
AGCTCTACCACAAGATGCTTGTGGTCGGTGGAATAGACAGAGTTTATGAGATTGGTCGTCAGTTCAGGAACGAAGGCA
TTGATCTTACTCATAATCCTGAGTTCACCACCTGTGAATTCTACATGGCATATGCTGATTACCATGACCTGATGGAAA
TCACAGAGAAACTACTTTCAGGAATGGTGAAACACATCACTGGAGGATACAAGGTGACGTATCACCCTGATGGTCCGG
AGGGACAAGCCTATGAGATTGACTTCACTCCACCATTCAGAAGAGTGAGCATGACACACGACCTGGAGAAGATTATGG
GAGTCAAATTCCCTCCTACTGACAGCTACGACAGTGAAGAGACACGTAAATTCTTTGATGACCTCTGCACACAGAAAG
GAGTTGAATGTCCTCCACCCAGAACCACTGCCCGCCTCCTTGACAAGCTGGTTGGAGATTTCCTTGAGGTTACCTGTA
TCAACCCCACATTCATCTGTGATCATCCTCAAATCATGAGTCCCTTAGCAAAATGGCACAGATCACAGAAAGGCCTGA
CAGAGCGTTTTGAGCTCTTTGTGATGAAGAAGGAAGTCTGCAATGCTTATACTGAGTTGAATGATCCAATTAAACAGA

GAGAGCTTTTTGAGCAGCAAGCCAAGGCCAAAGCTGAGGGTGATGACGAAGCCATGTTCATTGATGAGACCTTCTGCA
CAGCACTGGAGTATGGTCTACCACCAACTGCTGGCTGGGGAATGGGCATTGATCGTCTCACGATGTTCCTGACCAACT
CCAACAACATCAAGGAGGTGCTGCTCTTCCCAGCCATGAAGCCTGACGACAACAAAACATCAGCGCCCACAGAGGGTA
CCTCTGTCTGATGACGTGACCTCTGCCCCGTGCAGGAGTCAGTATGAAGTAGGAGCCTTTACCTCACTGTGGTCATCA
GTGGAGTCTGCAGGATTTGTGTTTTATCAATGTCATTCTGTCAAAAGTTGAATCCTTGCAGTTAAGGGAAATCATATT
GTCTCATTTCCAGTTGACTTTTCTTTGTTTTTCTTACACTTTTGCTGCATCCTCATCAGAGACATTTTAATCCTCATT
GTCATCCAGACAGTACATGGATGGTAGTAAATCAGGAAGTCGTGTGATCTGCTGTTGTATGCAGACCTGGATAAAATA
TTTCTGATTATACTTTGAGATACTACAAATGGTGCTTGATTGTGGCCTCCAAATAAACAGTTAATAGATCA

>Sequ21026EST2
ACATGGGGGCTCTTCTCTAGCCGGTGGGGACAGGAAGCGGACTTTGTTCCTAACTCTCCCTGTTTGACACCAACTTGC
GTTAGGGACAACGTTAAACCTCTGAAGTGTATTTTTTCCCCTTCCGACCCACGTCAACAGTTTAGGCTCTGAGAAGAG
GCGTTCTTGCGGCCACCAGGTGACGATAGCCATGAAGTGGGGCTGGGTGTTTTTGGCGGTCTTCCTCTCTTTGGGGAC
CCTGTCCTGGGGGGAGATGGGCTTGGAGATCCCCGAGTATGACGGAAGAGACCGCGTCCATGCCCTCAGCGCCAAGAA
CTACAAGTCCATCATGAAGAAGTATGATGTGATGGTGATCTACTACCACAAAAATGTGGACGGGAACCGCAGTGCCAT
GAAGCAGTTTCAGATAGAGGAGCTGGCTTTGGAGCTTGCAGCCCAAGTCCTGGACGATCTTGATGACGAGGACATTGG
ATTCGGCCTGGTGGATGAAAAGAAGGACACTGCTGTTGCCAAGAAGTTGGGTCTTGATGAGGTCGAGAGCATCTACAT
CTTTGCCGAAAATGAGATAATTGAGTATGATGGAGAGCTGGCCGCCGATACCCTGGTTGAGTTCCTCTATGATGTGAT
CGAAGAACCAGTGGAGATCATCGACAACGAGCGTGAGCTGAAGGGCTTCCACAACAACGATGAGGTCATCAAGCTGGT
CGGCTACTTCAAGAGCGAGAGATCTCCTCACTTCATTGAGTACGATGATGCTGCTGAGGAGTTCCACCCATTCGTCAA
ATTCTTTGCCACATTTGACCCCAAGATTGCCAAGAAGCTGAAGCTGAAGATGAATGAGGTTGATTTCTACGAGCCATT
CATGGAGGAATCCGTCACCATTCCAGGAAAGCCCTACATTGAGTCTGAGCTGGTTGAATACATCGAACAGCACGACAG
GCCCACTCTGAGGAAGCTTGAGCCTCACAGCATGTATGAGATCTGGGAGGATGACATTGATGGAGAGCACATTGTTGC
CTTTGCTGAGGAAGACGACCCAGATGGTTTTGAATTCCTGGAAATCCTGAAGGAGGTGGCACGCGAGAACACTGATAA
CCCCAACCTCAGCATCATCTGGATCGACCCTGACGATTTCCCCTTGCTGGTTCCATACTGGGAGAAGACCTTCCGCAT
CGACCTCTCTTCTCCTCAGATCGGTGTGGTTGATGTTGAAGATGCCGACAGCGTCTGGATGGAAATGGATGACCAGGA
TGACATGCCCACATCTGATGAGCTGGAGCAGTGGATTGACGACGTTCTGTCTGGAAAGATTGACCCAGATGATGATGA
TGATGATGAGGATGACGATGATGATGACGATGATGATGACGATGACGACGACGATGATGACGATGACGATGATGATGA
CGATGATGATGACGATGATGATGATGACGACGACGATGATGATGACGATGACGACGATGATGATGATGATGACGATGA
TGATGACGACGACGATGAATAAATGTTCACCACCATCTTTCACTTCACTATAATTTAGCCAGTAATGGCAAAACCCAA
AAGTAGTAGCAGTTTTTCTAACATCTCGCGGCGAACACACTGTGTTTGTTTGCTTCCTGTATGGTTTCCTGTAAGGGA
CAAACATAATGACCCTATGAGAAACTTTTACTGTCAGTTAGGGAAGGAGATTTTCAAAGAAGCAGAGCATTTGCATCT
CTGTTGATGAACAGCTCTCACCTCTGAGGTTCACAATGAATGTGAGCTAAGAGGCTAGCACAGGCTAACTTCCCTGAC
ATTTTCTACCTAATGTCACAGACAGGGGTTACCCCTCTCTCATTTTCTCCAATTTACTGTAATCCTGTTTCAATGACA
TGTTTTCTACCTGATGAAGAGAAGAAACGAACACTTTCTTGTACTATTTCTAAAGAGATTAAACCTAAACTCACTGGA
CAGTCTTAAAATTAGTGCAATCTTGGAAAGCTGCCAAATATCGGATAATAAATAAATTACAATACAATTTGTTTCTGC
TCTCACATTAAAAA

>Sequ21033SNP2
CCTGATAGGCCAAAAGGGATGCACTGCTCAAGATGCGGAGGGAAAGTGAAAATTGCTTTCTGGTGGCAGTGTTGCATG
TCTGTGACAGAAATGTCTCGAACTCAACTGGGGCTACAGGGGAGATATTAAAGGCACCAATGATATTTAGATGTTATT
GCCCCTGGAAACATAATTGAATATACGTGGACACAATGTTTTCAGGGCCTTTAAATGATTCAGATCTTATTGAATCTA
ATCATCTCATCAATTACAGACTTAACTGAACTCAACTAAAATTTCTCTCAATACATTTTTAATTTGTGTCCCCAAAGC
CACTAGTCATTATAACTCTGCAGGTTTGAATTTGCAATCTTATTTTATTTTTTTTTAACTTGTCTTAGGTATGAATGC
AGCCACTAGATGGAGTAGTCGGACACTTTCCCTCCCCATAGAGAGGGACAGCATTTTAGACCAGTCCGTTTCAACAGA
TCAGTATAAATCAATCAGTGGTGTGGTGTGATTGCCATTGAGGGAGTTACTGTGCTTCTTCTGCATTATAACTGTTTT
GTAACATTTTTTTGTAGGGAAAAAACTACAGAAACAGAATGTGCCATTTCTTTCTCCCTGAATGTGCAATACAAGCTG
TTTCTTGTGAAGGTGCAACTACATTGCTGTCACTCTGTGGTCGTGTAGCTCCACCTGCTCCGGTGTTATAATGCCCTC
TCCTGTGTGTGTGGGACTAGCACCACCTCACAGAGGTAGAGGGGCATCAGTTCAGACCTTCATCTGGATGTGGCGTGC
TGTCTCTAGTTTTCTGGTCTGTTTTGTAATAGAATGTGCTCCTTGGCTGGCCTGCAGGCTTTACAGCTCTGTGTTGTC
TTTTTACTGTTTACTGCTATTTAACTGTCCAGTCCTTCAAAAGTAAAGAAAATAATAATAAATGAATAAAAAATCAAA
AGAAACTGTAGCCTGCTTCTACAAAATCTTAAAGCTCTTCTGATTTGCATATATCGTACTGTAATGTTTTATTTAAGA
GTTTAGTGAAAAAAATGTCTGCATTTAAACTAAATTATGGGAATTAAAATTACAAAGAAT

>Sequ21053EST2, Sequ21053SNP2

GTGCAGAGAATCAAGGCCAGGGAGATCGTTCCCGGCGAGTGTGGTGGAGGTTCCGTTGGTGACAAAGTGCCAGCCGAC
ATCAGGATCATCTCCATCAAAATCTACAACCCCTGCGTGTGGACCAGTCCATTCTCACTGGTGAGTCCGTCAGCGTGA
TCAAGCACACCGATGCTGTCCCCAGACCCCCGAGCTGTCAACCAGGACAAAAAGAACATGCTGTTCTCTGGCACCAAT
ATCGCTGCTGGAAAAGCCACCGGTATAGCTGTAGCAACTGGCGTCACCACTGAGATCGGTAAGATTCGTGACCAGATG
GCTGCCACTGAGCAGGAGAGGACCCCTCTGCAGCAGAAACTGGATGAGTTTGGAGAGCAGCTCTCTAAGGTCATCTCC
CTCATCTGTGTGGCCGTCTGGATAATCAACATTGGTCATTTCAATGACCCCGTCCATGGAGGCTCCTGGATCCGCGGT
GCTATCTACTATTTCAAGATTGCTGTGGCTCTGGCTGTGGCTGCCATTCCTGAAGGCCTGCCAGCTGTCATCACCACC
TGCCTGGCTCTGGGAACGCGCCGCATGGCCAAGAGAACGCCATCGTCAGAAGCCTGCCCTCTGTGGAGACCCTGGGCT
GCACCTCAGTCATCTGCTCCGACAAGACTGGCACCCTCACCACCAACCAGATGTGTGTAACTAAGATGTTCATCATCG
ATAAAGTGGAGGCCGACAATGTTTCCCTCGGCCAGTTCGACATCTCTGGCTCAAAGTACACCCCTGAGGGAGAAGTTA
CAAAGAATAACTTGTCCGTGAAGTGCGGACAGTTTGACGGACTGGTGGAGCTGGCTACCATCTGCGCTCTGTGCAATG
ACTCTTCTCTGGACCACAATGAGTCCAAGGGTATTTATGAGAAAGTGGGTGAGGCCACTGAGACAGCCCTGTGCTGTT
TGGTGGAGAAGATGAACGTGTTCAACACTGAAGTGCGTGGTCTGTCCAAGGTGGAGAGGGCAAATACGTGCTGCACTG
TGATCAAGCAGCTGATGAAGAAGGAATTCACCCTGGAGTTCTCCAGAGACAGGAAGTCCATGTCAGTCTACTGTTCAC

CTGCCAAGTCTGCCAAAGGCCCTGTGGGAAACAAGATGTTTGTCAAAGGTGCTCCAGAGGGTGTCATCGAGCGCTGCG
CCTACGTCCGCGTGGGCACCACCCGCGTACCCCTGACTGGCCCGGTCAAAGACAACATCATGTCAATCATCAAGGAGT
GGGGCACAGGGCGCGACACCCTCCGCTGTTTGGCTCTGGCCACCCGTGACACACCTCTGAGGAAGGAGGAGATGAACC
TGGAGGACTCAACCAAGTTTGCAGACTATGAGACTGACTTAACCTTTGTGGGCTGTGTCGGCATGCTCGACCCTCCTC
GTAAGGAGTTATGAGCTCCATCGAGCTGTGCAGGGCTGCTGGCATCCGTGTCATCATGATCACTGGTGACAACAAGGG
CACAGCTGTGGCCATCTGCCGTCGTATCGGCATCTTCAGCGAGGATGAGGATGTCACTGGCAAGGCCTACACTGGTCG
TGAGTTTGATGACCTCGCTCCATATGATCAGAAGAAGGCCGTGCGAAAGGCTTGCTGCTTTGCCAGAGTGGAACCGTC
CCACAAGTCGAAGATCGTTGAGTTCCTGCAAGGCTTTGATGAGATTACTGCCATGACCGGTGATGGAGTGAACGATGC
CCCTGCCTTGAAAAAGGCGGAGATTGGCATCGCCATGGG

>Sequ21060EST2
TTTTCTTCTTTTCCGAAGACTCTGTTACTGCTAGCTCCTCCTCTTCCTCCTTAGGTTCCTCAGCTTCATCATCTGCTT
TCTCTCTTCTTCTTTTTCTTCTTGGGAGTCTCCTCCGCTGCTGCTGATGTTTCCGCTTCTTCCTCTTCTGTTACTGGT
TCCTTTTTGGGCTTTTTCATTTTGACGACGGGCGTTACAGGCTCTTCCTCCTCCTCCTCCTCCTCTACCTCCTCAAAC
TTCCTTTTCTTCGAGGTGGAGGGAATAGTGGAATCGCCAGATGGATCGTACATTCTTACTTCACTCTTGTGCTGGTAT
TTGTCCGACTTCGCCATTGCTTTGCCCGTTCCGCTGATTCTTCGGATTCCCTTCTCCTCCAGCTGCCGCAGCCTGGCC
TCCAGCTTGGCCCGGTTCTCTGCTCCCATTTCTGCGTTCGTGTCCTCTCCCAGGGCGTCATAGCGAATAGCCAGGGAC
GCTTTAGCTGCCAGCATCCTGGAGATCTTGCCCTTGTTCTTGGCAGTGGTCTGACCCACTAGAGAGGCGTGGTATATG
AGACCGTACTTGGGCGTGTCTTTGCGGGTCTTCAGGGCTCTAAACAGAGCCTTTTCTGCACCGAGGATCTGCACTGTG
GAGGCTGGATGCTTCGCCAGATTCAGGAGAGAACCGGCATGTGAGATGAGACGGGCGCCGACCAGCTCGCCCACCATT
ACTGTCAGGTTGGGGCGATCGCCATCATTCGGTTCTTCAGGTAGTCGTAGAGCTGAGCGCGGTAGTCTGTAATCTCAA
TCACCTGATCACACAGGTGCCTGATGTTGCCGATGTCCTGCTCCGACACTTCTGTTCCCATGGAGATCTCAGCGGCCA
GTTTGACCTCCGCCTCGATTTCCTCTGGGAGGAGGTCTGACAGATCAGAGCTGGACACGTTCGTGCGGTCACCAATCT
TGCGGACGGTTTTGCAGTAAGCCAAGTTGTCTGTGACGACTTTCCCGAGCTCAGGGAAGTGCCAGCCGTACCACTCCC
TGCAGCGCATAATGTAGTTGTTGAGCTCCTTATCCAGGTCGTCCAGAAGAGAAATGGCCTGCACAATCATAGTGTCCA
CCTTGTCTGGACTGAACTTCAGCTTGTAGCGTGATAAACTGTGAGCCAACCCCAACGACATGGCACTGATCTCCCTGG
GTGGAAGTCCAGTGATGAGACTCTCCATCTGGCTCCTGATGCATCTCATCAGTTCAGCCACAGCGGTGTTGTGGACAC
AGCTCAGGTCCAGCTTTTCTTTGATAACTCCGCCGAGTTTTGCATCGCTGATTGCCAGCTGCTCATGAGCCTCCTTGG
CAACGACCTTCTTCAGCGCTTTCTTCAAACTCTTGCCAATTTTTCCCTCAACAAGGGCAGTGGCAGCTGCTAAGGCCT
CTGTTGTGTCCTGAAACTTCTCAAAGTGCTTCAGCTTCACAATCTTGTTCGCCTTTTCAGGAGTTTCAAACTCCTTGT
ACAGGCTGTCGACCTGTTGCAGCTTGGATTCATCGAGGACCTTGAATATCGCATAACCAGCGGCGGTCTCAAATAACA
CGAGCATTTTTGCAGCGGCTCGGTTATCTGGTAAAGTTTAAGTTGTCTCTACGTAGGGAGCGATCACGTCTCTCTCAA
ACTCACCACGCTTCTTCTCTCACTCTGTCTC

>Sequ21065EST2
CCACGGTGGCTGAACAAGTGAAGAAATCATGAATCATCAGTGAGGGCCTTAAGTCGAAGCTTAGATTGTTTATAACAG
ACGATTTGAATTGTTAGTGTTGTTGCCCACAGTAATTTCCTTGGAATAGCTATATTGTAGTAAAATATTTAAGTTGTT
TGAATTAACGTTGAAAAGCTGTTGGCCTATTTCTTGGGCTTTGACATTAGCTAGCTAAACCTACCAAGCTCAGTTAAA
CTTGTTAACACTTTTGTGAAAGATGGCTGACAACGGAGCACACCCGGCTGAAGAGGATCCAGCTGCAGCTTTCCTGGC
TCAACAAGAGAGTGAGATAGCGGGGATAGAGAACGACGGCGAAGGATTTGGGGCGCTGGAAGGAGCGGACGGCCAGCA
GCCGGCTCAACCGCAGTCGGCCAACTATGATGGTTTCGAGGACGAGTCTGCCACAGTGAATGGGGATATGTTTCAGGA
GTCCAATGGCCCAACAGACAGCTATGCAGCCATAGCCCAGGTGGATATTCAGAGACAAGAGCCAGAGAGTTTACGCAA
GTGGAGGGAGGAGCAAAAGGCACGCCTTGAAGCATTAGATTCGGCATCCAAGGCGGCAGAGGCAGAGTGGAAAGAGAA
AGCCAAAAAGGAGCTGGAGGACTGGCATGTGCACCAGAATGAGCAGATGGAAAAGAACAAGGCCAACAACAGAGCATC
AGAGGAGGCTTTCCTGGCAGAGAGTGATGGCGACAGCCCAGGATCTGAATGGGAGAGAGTTGCCCGTCTGTGTGACTT
CAATCCCAAAACCAACAAACAGGCAAAGGATGTTTCTCGAATGCGTTCTGTCCTCATCTCTCTCAAACAGACGCCTCT
AGTTCGCTAAAGGAGGCCGCAGGGAGAATTCTTGTTCTAGAAACATTGCCTTTTCAGTTTTAACCTCACGTCACATCA
GATGAGTTTGTATGACCTTTTCCATAATATTCCTGGTATGTTCATAACCTTTGAAAACTTCCCGCCTTTCTTGATGCC
TGTTACAATTAAATAATACACTTAACCTCACTTTCTTGTGAGAAGATGAAGGACTGCTCTATTGTTACGACATCCTTT
AGCTGGTCAAACTGACTGCTATACATAAGAAAATATTGTTACTGTAAGTTAAACATATTTCCTTCCCCTTAACTTATA
AATTGTTATTTGGTCAACTTTATTGCATGCCTTTGCCAATATTTTGGATGCACAATTTTTAGTTGTTTTATAAATTAG
CATTTTGGTCAATGTCAAACACTTCACATATGTTATGAGCACTGTTAAGGATTAGATTGTAACCACTTATGCCTCTCA
ATGTAAAATTCTAAACACAAATTCTTTGTTCACATTTGTTGTATTATAGCCCTAATGACCATATTTAAGGTAGCTAGC
ATATTTTCCTCTTTCGCGCACATAAGCTTGTGGCCTTGTTAATCTCGCTCTTACTCCACTTTGAGTATTTAGCCAGAT
GGATTTCCTCTTCAATTAGGCACGATTTCAGCAACAACAAAACGAAAATCACAACCAAAGATTTCATCTAAATTTTCT
GAGTTCACATGATTAACAGCTTGTGCAATTTCTCATTTCACTGGTTCTCTCAGCAAATGTTTTATGCACCATTTTGTG
TCACACTAAATGTGTTTTATATTTTTAAAATGGCAATCAATAAGGTGTCAGTTTCAGCATTAATGTCTGCACATTGTT
TCACATGTTGCCTATTTGGACATTACGAATACTGCAAAAGTGAATGTTAAAAGGGAACTGGAAAATAAAAACAATGGA
GTATGATCACCTAAATTAATTTGTCCTTAAATATTTACCAGGTAAGACTGTTGCCATCAAAATACTCTGGAGATAATC
ACTTGAATCCCAGTTTGTCCAGCTGATGTATGAGGAGCTAAAATTATGTCTGACCCAGTGCCAGCATCCATCATTACA
CTTTTAATGCTACTGTTAGAGGATTTTACCCACAATTTTTATCATTCAAATGTTAAAAACACAGATGAGCGTGGAAGT
CCATGAAGTAAGAATATGTTTGCAGGTACACAGTAGCAAATAGTGTCTACTTCTTATTCTATGAATGCATGATTCAGA
TTTGTTGTTTTACAAGTGTCACCCTTAATGGGCCACCAAAATGCCTCTGCATAGGCAAAGTGGTTTCACAAAGTGCTG
TTCCAATCGTAAACCAACATTTGAAGCCCTCCTTAATCAATGATTTTTAGGTGACATAAAAGTCAGAGTTTAAGGTGG
ACATTGAGACTGGGTCAGTCTTTGTCTAAAATCCTCCTCCCATTTCAATCTACTCACCCCAGCCATTAATATACAAAG
TGTGTTCACAATAAACTTCCCCGGTGATTTTTTTAAATAACTTGTTTAATAAAGGTTGTAATAAGAAAAAAAAAAAAA
CCGACTACCCTAT

>Sequ21069EST2
ATTCAGCGGGACTGTCGCGGCATTTGAAACATCTACATCTCAAGCTGCAAAATACTGTCACTTTACGCCGAAATTACC

GCCTTTAGAGTGAACTGAAATGATCGATATGATCGAGAAGGAGGACCCGGTCATCAGCGAGGAAGAGGCGGCACAGTA
CGACCGCCAGATTCGACTATGGGGGCTTGATGCCCAGAAGAGGTTACGAGGATCCCGTGTTCTCTTGGCAGGTTTAGG
TGGTCTTGGGGCTGAAGTAGCCAAAAACCTAATCCTGGCTGGGGTTAAAGGACTCACTTTGCTGGATCATGAACAGGT
GTCAGAGGAGTCATGCCGAGCTCAATTCCTGGTTCCAGTGACGGCTCAGGGTCAGAACCGGGCCCAGGCCTCTCTGGA
GCGCGCACAGAACCTCAACCCGATGGTTGAGGTTCATGCCGACACAGACAGAGTCGAAGACAAACCAGATGACTTCTT
TCTGCAGTTCGATGCAGTGTGTCTGACAGGCTGCTCCAGAGACCTGATGGTGCGGGTCGACCAGCTCTGTTCTCAGCG
CAACATCAAGGTCTTCTGTGGAGACGTCCATGGTTACTATGGTTACATGTTCTGCAACCTCGGACAAGAACACAACTA
TGTTGAGGAGAAACCCAAAGTAGTGAAACCGACTGGAGATTCTAACGACGGCCCAGAGGCAAAGAAAGCCAAAGTTGA
CCCCAACGAGACCACCATGGTGAAAAAGACGACCAGTTTCTGCACCCTGAAGGAGGCTCTGGAGGTTGACTGGACAAG
TGAGAAAGCCAAAGCTGGTCTGAAGCGAACACCAGTGGACTACTTCCTGCTTCACGTACTGTTGAAGTTTCGCACAGA
CAAGGGCCGTGACCCCGACCCACAGTCCTTTGCAGGAGACAGTCAGCTCCTGAAACAGATCCGCGATGACGTCCTCGA
GGCCTTGGCAGTCAGCAGTGACCTCCTGAACGATGACTTCATCAGCTACTGCTTCTCTGAGATGTCTCCAGTGTGCGC
CGTCATGGGAGGAGTTCTGGGACAGGAAGTTGTCAAGGCTCTCTCCCAAAGAGACCCTCCCCACAGGAACTTCTTCTT
CTTCGATGGCCGTAAAGGCAACGGCGTGGTCGACTACTTTGGACCAAACTAAACACCTGTCGCACCTGTTTTACACAC
AAAGTCCAGAAAGCTTTTCTCTTTGCCTCAGTTTGTTCAGTCAGATTATTTATACCAGAACTTGCTCCACTAGTCATA
AAATCCATCTGTTGTTCCTGATCTTTTGTCATTTGATCCATTTTCCCACCAAGTCAAACACTTTCTTGGGCAAACAGT
GAGATTTTTTTACATTTTGCTAATAAAACATTGTTTAAGTAAAAAAAAAAAACCGAACCTCCCTA

>Sequ21083EST2
GAGTGTACAAGAAGTAAACATGAAATGAGTCTCGGCGGCGCAGTGAATTAAGGAACAAATTGGTCTTATTATAGTAAA
AATGGATCGTTTTTCGTGGTCAAATGGGCTTTTAGAAATAAATGAAACTTTAGTGATCCAGCAACGAGGTGTGAGACT
GTATGACGGAGATGATAAGGCTAAGCTGGATGTTGGAGTTGTCCTGTTGAGCACCCATCGGTTGATCTGGAGGGACGT
TAAAAATCATGAATGCTGCATAGCCATGCCCCTGTCACAGATCATCTTCTTTGAGGAGCAGGCTGCAGGAATAGGAAA
GAGTGCAAAAATCGTTATTCACCTGCATCCAGTGCCTGCCAACAAGGAGCCTGGTCTCTACCAACACAGCAAATACTC
CTACATTAAACTGTCCTTCAAAGAACATGGGCAGATAGAGTTTTACAGGAGGCTAACAGAAGAGATGACTCAGAAAAG
ATGGGAGAGTACACCAGTTTCACAACCCATCCCCACAGGAACTGGCTCTCAGGCAGGAAAGACACGTGCTGTGGGGAT
TGTTGGCATCGAGAGAAAGATAGAGGAGAGGAGAAAAGAAACAGACAAAAACATTTCAGAGGCCTTTGAGGACCTCAG
TAAACTGATGGTGAAGGCCAAAGAGATGGTGGAGCTGTCCAGATCTATAGCCAACAAGATCAAAGACAAGCAGGGAGA
TATAACAGAAGATGAGACAATCCGGTTTAAGTCCTACCTGCTGAGTATGGGTATTGCTAACCCAGTTACAAGGGAAAC
GCATGGATCAGGCACACATTACCACATGCAGCTGGCTAAGCAACTGGGAGATATGCTACAGGCCCCTCTCGAGGAGCG
TGGGGGTATGATGGCTCTGACTGAGGTGTACTGTCTCGTCAACCGTGCTAGAGGGATGGAGCTTTTGTCTCCAGAAGA
TTTGGTAAACGCATGCAAGATGTTTGAGTCGTTGAAGCTCCCACTGAGGCTGCGCGTGTTTGACAGTGGTGTGATGGT
GGTCCAGCTGCAGTCTCACAGCGAGGAGGAAATGATAGCCTCAGCACTGGACAACGTGTCAGACAAAGGCTCCTTGAC
AGCAGAGGAGTTTGCCAAGCTCTTGGGTCTCTCTGTTCTTCTGTCGAAAGAGCGGTTGTTGCTGGCTGAGAAGATGGG
CCACCTGTGTAGAGATGACTCTGTTGAGGGCTTGCGATTCTACCCAAACCTCTTTTGACCTGTTTACACTCTTACACT
TTACTCAGTATTCTTGAGATAGAGGAAAATAAACTGGGCCACACTGTCTCTTCCGCTAGACGGATGCATTAAAACATG
TTCTGACCTCTACATGTATATACTGTTGTACAGCAGTAGAGTGCATCCTGTACTTCGTGCTATCCAGTCGATCATTGA
ATGGTTAATGTTCTGGTGGTGGTACATCCTTCATCCACTGTACGTACCACCTGCTATTTTCTATCTAGGTCTTTAAAA
TGTGTCTGCAGACTGCAAATACTGTTCACGCAATAAAATGTTCCCAGGTTTACTCAACTTAGTGTGTCATTAGTAATA
AATGGGTTTGGGAAAATTGACTTAATGGGATGTAAACAGTCTATTTTTTTCACGGCTTTCATTTTGAATTTGTTATGA
AAATCCATAGCCACTAATCACATTTTCCAAAGTAAAATGTATATAATAAATGATTAAGGAATAAAGCTCTGCCCTGCT
ATC

>Sequ21104EST2
GACGAAACTTCAAAGCTCAGTCCACACTTTACAACCGTCGGATTTATGTCTTTATACCGAGTGATGCTGTGAGGGAAA
GCAGCGCACGTGTCTGCGGGTGAAACTCTAAACCTTTGCGGGAAGATGAGCTTCAGTTCGTAGCCGACGGTTCGAAAC
TAGAGCTTCTCTGTTCTCCTGTTTGTTGAAGTGCCTGTGAATTCAGTCATGGCTCGCGGCTGCATCTGCTGCGTGAAA
TACATGCTCTTTCTCTTCAACCTGCTCTTCTGGCTGGGTGGGTGCGGGTTGCTCGGTGTCGGCGTGTGGCTGTCGGTT
TCCCAGAGCAGCTTCGCCACCCTGTCGCCCTCCTTCCCGTCGCTCTCCGCCGCCAACCTCATCATCACACTCGGCACC
GTTGTCATGGTGACGGGCTTCCTGGGCTGCCTGGGTGCCATCAAGGAGAACAAGTGCCTGCTGCTGAGCTTCTTCATC
GTTCTGTTGATCATCCTCTTGGCCGAACTCATCCTCCTCATCCTGTTTTTCGTCCACACCGACAAGGTGAGCGAAAAC
GCCAGACGGGACTTGAAGGAAGGGTTGGTGCTGTACTACACGGATAACAACGCGGGCCTGAAGGATGCGTGGAACACC
ATACAGGGAGAGTGGAGATGTTGTGGAGTGATGAACCACAACGACTGGTACACCGCCCTGCAGGATCACGTTGTTCCC
GACCGCTGCTGCCAGCAGATTTACCCGGGCTGCGGCCGCAACGCCTCCAACGCCTTCTGGACACGGGGTTGCTATGAG
AAGGTGGAGGAGTGGCTGGATGACAACAAACACCTCCTGGGAACCATCGCTATGTGTGTGTTGGTCATACAGCTCCTG
GGTATGGCTTTCTCCATGACGCTTTACCAACAGATCCACCGGGCAGGGAAGAAGTACGAAGCCTGATGGTCGTTTCAA
ACTGTTTAATATCGGGTACATAAAATATATCACCTCTGTCATGCCTGTTACTCTTACCTATTGTTTATAGTCAGAATA
AGTCAGTTATCGTGTGTGTGCCACTACAGCCTTTTTATAATGTTTTCATCAGTTATTGTTGTGAATAGCATTGGAACT
AGTTTTTCTTATGGTTTAATTGTTTGTTTTTTTT

>Sequ21110EST2
ACTTTAAATGTTTGTCCTGCAGAATTTATCAGTACATTCGATGCCTCTGTTCCTAGTATGGCTCCACGGACAATCTCA
GCCAAATGGAGGCGAAAGCTCCTCAGCTAAATACTAACTTTGGTTAGAAGGTTTCTGTGACATATTTTACCCCACTGC
CATTCTGGAAGATAGCAGTGGTCAGCATGGCGTTGAAGGAAATGAAAAGCACCACCGTGTCTGACATCCCCAGCAAGC
CTCCCTTCATACCCATGGACCTTTACAGCCTCTCACCCGGTGGAGCATTTGACAGTGGCAAACACAATCTGACGGCTG
AAATGCCAGACTCAGATTCAGGAATCTCTTCAAACACAAGTCCGAATGCTAGTTCACCTGCAAAATCTGCATATGGAG
ATGGGTCTTTTGGTTACAGTGATTCAGACATGGAGGAGATGGACCACAACCCTGGAAGTGCGGAGTCTGAATACTCAG
AGATGTTCTCGCTAAATTTCCAACCTGATGATCCAGTTTCTGATTCGGCACCAATAGGGCAGCAACAACGGCAACAGG
GGAAGAAACCCAAACATCACAGGACGGACCCATCAGAGGAGAGTGGCCACAACAACGCTCCCTTCACCAAAGACAAGC
AGAAGAGACGCTCCGACTTGCGTCTCCCCAGAGACGAGCAGAGGGCTAAGGCCCTCAAAATCCCTTTCACTGTTGACA

TGATTATAAATCTGCCTGTTGATGACTTCAATGAGATGATGTCAAAGCACCAACTGAACGAGGCCCAGCTGGCCCTGG
TCCGGGACATACGCCGCCGCGGCAAGAACAAAGTAGCTGCCCAGAACTGCCGCAAACGCAAGATGGAGAACATAGTGG
GTCTGGAGGGCGAGCTGGACTCACTGAAGGAGGAAAAGAGAGCGCCTGCTGAGCGAGAAGAGCCAGAACATCACAGAC
CTGAAGGAAATGAAGCAGCAGCTCAACAGCTTGTACCTGGAGGTCTTCAGCACGCTGAGAGATGAGAAGGGGAACACC
TACTCTCCATCCGACTACTCCCTCCAGCAGTCGACCGACGGCAGCATCTTCCTTGTCCCTCGCATTAAAAAGACTTTC
AAGAGCGAAGACAACCACTTATCTCCTTTGTAATATGGTACTGCGTTAACATAGTGATACTATGCAATAACTTTGTAG
TCGCTCAGCTTTAAAGCATTAGTAGCTCAGCAGAACTATTGTATAGTTTTTGCTTACTTCTCTCGAATGTTGTAATAT
TTTTGATATTTTACTTCTTTTGTACTCTGATACCCTCACAGATTCTATAACCAATATTGTTTGTAAATATAAACATTA
CATGCTTTTGAGATTGATAAAAATGTTACTGTACGTGTATATATATATATATTGTATATATCATCTTTCTGTATGCTA
ATGGTTGTTTCAATCATTGCCACTCATATTTTATAACTTTCCCTTTATGATTTGCTGCCCAGGTTCTTTTTTTAAAAA
CTTCTGTGCTTTTCAATAAACTATTCCTATAGACTAAAAAAAAAAAAAAAAAAACTAAACGT

>Sequ21118EST2
ATTAAACATCAAGAAGAAGAAGAGTGGCAAACATAGTGGCTCTCATTTTACACAGTAACGAGATTGTTTACAAAGAAT
GCAGTAAGAAACAGGTAGCGTGTGCCGTGTTGCCTATTCCTGAGCACGATGGCTACTCAGAGAGTGCTCCCTCAGAGC
AAAGAGACTCTGCTCCAGAACTACAACAAGAGACTGAAAGATGACATCAGGTCCATCCTGGACAACTTCACAGAAATC
ATCAAAACAGCAAAGATAGAGGACGAGACGCAGGTTTCTCGAGCAACTCAAGCAGAGCAAGACCATTATGAAATGCAT
GTCAGAGCAGCCAACATTGTACGTGCCGGAGAGTCTCTGATGAAGCTGGTGTCTGATCTGAAGCAGTTCCTGATTCTG
AATGACTTTCCCTCCGTGAATGACGCCATCAGCCTCCAGAACCAGCAGCTCCGCACGCTGCAGGAGGAGTGTGACAAG
AAGCTCACCTCGCTCCGTGATGAGATCGCCATCGACCTGTATGAGCTAGAGGAAGAATATTACTCCTCCAGCTACAGT
CAGTGGGACAGCTCTGATCTGCCACTGTGCGAGGCCTACAGACAGCGGGACAGTTGGGCCTCCCCAGGCAGCAGCTGC
AGTTCTACCCAGGGCGACAGAGAGGATGTGGACGGACCTCCCTCACAGGAGACAAACCCCCAACACCACCTCAATGGA
CATGGGACCGCCTCTATAGAGAAACCATGAGGAGGAAGTTGGACACTGACTGGTATGGACTTGTGCTCAGTTTGGTTA
ATTTAGTTTCCATTACACATTGTGAACTAGTGTCTGATTAGGTTTTGCCACAGTTTTGTGTTGAGGCCTTGAAACTAG
ACTAAAACTTTGGCATTGAGGTAGTTTTCTTTTTGCATTAACAGATATTGCAGGAAAATCGTGAAATTAAGAAACTGT
ATTGAACAAGAACAGCCTGAAGAACTGATCAAAAGTAACATTCCCCTCAGTTTAAATGACTATTCAAAATGAATAGAA
AACTTGCATACACTATATACTGTATACACTACTGAAAAAAACATTTAAAACTACAATAAATTTTAGTGTACCGTTGCA
GCACTGAAATAAAGATATACTTTTTTATATTCCTATGTTGGACTTAGCTCAAGTATTGAATGAGTGTATGTTCTTACA
TTTATTTTGTAAAAGCTTTTTGGAATATTTTATTTCTGCTTTTGTACTCCACCAAGCACTGATCAAATTAAGAACAAG
TTCAATGAACGAAAAAAAAAAAAACCGAACCTACCCTA

>Sequ21122EST2
ACGTAAAAAGATGAATCTTTATTGCACCAAATTGAGTATTTACCATTAATAAACCCAGTGATTATTATTTTTTAAATA
AAGATAAAAACCTTCATTCATTGTAGTTATTTATATATTCTTCAATTTCATCCTCGGAAAGAAAAAAAAAAGTAAAAT
TAAAACAGTCCACAATCTAAAGAAACAAAGAAACAGTCATATAAATAAACCGTTAAGCCTGACATCTATAAAATAATA
ACATGATCAGTGATGACATATAAAAGGCATCTTTGCTATGTGATAATAAACTAAATCAACGACAGGATTACAAAAAGA
AGACAAAGACACAAAGACAATGGATGATTCCATCTAACCCCCTTAATAATGAAGAAAAGGACACAATTCATTCATTTG
GAAAAAGTCATGAAGGTAAAATACATGTTGCCTCCACTGTGGGTGTCCGTGGCAGAGGACTGGAGTGCTGAAACGTTT
CCACCCCGGGCGGCGGAGCGACGGATTTGGCCTCAGGCGTTTGGGTTTTCCTTCATTAGCTCCACGTCTGCGTCCACC
ATCTCCTTCACCAGCTCCTCAAAAGTCACCTTTGGCTTCCAACCCAGCTGCTGATACGCTTTGGTGCTGTCACCTTGT
AGGTAGTCCACTTCGGTGGGGCGGAAGTATTTTGGGTCCACCTTCACATGTACCACCCCTGTCTCCTGGCAGCGGCCG
ACCTCCTTCTCATCCTTTCCTTCCCACACAATAGTCTTCCCCACACGTTTGAAGGCTTTCTCTACAAACTCCCTCACA
CTGTGCACCTCCCCTCGTTGCTATGACGAAATCCTCTGGCTCCTCCTTTTGTAGCATCAGCCACATCGCCTTCCACGT
AGTCTTTGGCGTGGCCCCAGTCCCTCTTGGAGTCCAGGTTGCCCTAGGCTGAAGTTCTCCAGTTGGCCCAGGTGAATC
TTTGCCACAGAGCGGCTGATCTTACGCGTCACAAAGTTTGCACCTCTCCTCGGGCTCTCGTGGTTAAAGAGGATCCCG
TTGACAGCGAACATGTTGTAGGCCTCTCGAAAGTTCACCACGATCCAGTAGGCGTAAAGCTTCGCCGCACCGTAAGGT
GAGCGAGGGTAGAAGGGAGTGGTTTCCTTCTGAGGGATCTCCTGGACTTTGCCGTACAGCTCGCTGGTGGAGGCCTGG
TAGAACTTGACGCTGTTGGTCAGACCGCACGTGTTTATGGCGTCCAACAGACGGAGCGTGCCCACGCCGTCCACGTTA
GCGGTGTACTCGGCCAAATCAAAGGAGACCTTGACGTGACTCTGAGCGCCCAGGTTGTAGATCTCCGTCGGCTTCACC
TGGTTGATGATCTTCACCAGACATGTGCTGTCAGTCAGGTCGCCGTAATGCAGTCTTCATGTTGCCTTCAGTGTGTGT
CTGGGGGTTCTGGTAAAGATGCTCAATGCGACCAGTGTTGAAGGAGCTGGAACGACGCAAAATACCATGAACCTCGTA
GCCTTTTGCGAGCAGGAACTCAGCGAGGTAGGAACCATCCTGTCCGGTGATACCGGTAATAACCGCCACCTTCCTCTC
CTTCATCAGCTGTCCGTTCCCCGCGGAACTAGCGTTAGCAGGCTCGGAGCACAGGGCCATCTCTGACTGGCTGGATGT
TTAGTTTGCTACCACACCGACTGACACAACCGGCATAAACCGTGTGTGTGTGTGTGTTTACAGTGTAATGAGTAGCAC
CGACGAGCAGCTTCCTCAGCATCCAAACTACAACACCGCTGGTAAGTGAATGACGT

>Sequ21125EST2
GAGTGAACAGCTAGCGACGGCAGTATTCAGGGGAAAGGTCGCAGCTGTGTCCGGTTCTTATGTTCACCTCGCATTGAT
TCAGAGTGCCACAACAAAGATATCAGTGGGAAGGTTTCATCCGACAGCGGTGGGTCGTTGAATAAGCATCATGGCACT
CCTCAGAGGTGTATTTATTGTAGCTGCCAAGCGCACTCCATTCGGTACATATGGTGGCGTGTTGAAGGACCACAGTGC
AACAGACTTGGCTGAGCATGCAGCCAAAGCTGCCCTCGCTGCAGGGGGCGTGGCACCAGAGCTCGTAAACAGTGTCAT
CATGGGAAATGTCATGCAGAGCTCAGCCGACGCCCCCTACATTGCTCGTCATGTGGGTCTGAGGTGTGGTGTTCCTAT
CCCAGTGCCTGCTCTCACTGTGAACAGACTGTGTGGATCTGGTTTCCAGGCCATCATCAGTGGTGCTCATGAGATTTG
CCTGAAGGAGTCAGAGGTGGTCCTGTGTGGAGGCTCAGAGAGTATGAGCCAGGCCCCGTACGCTGTTCGCAACATCCG
ATTTGGTACAAAGTTTGGAGTCGATCTCAAGCTAGAGGACACTTTGTGGGCGGGTCTGACTGACCTGCACACCAAAAT
CCCGATGGGCATCACGGCAGAAAACCTGGCAGAGAAATACCAGATCACACGAGAAGACTGTGACAACTATGCATTCCA
GACGCAGCAAAAGTGGAAGGCTGCTCATGAGGGTGGTCACTACACAGCAGAAATTGCTCCCATTGATGTGAAAGCAAG
GAAAGGCAAGGTGTCCATGGCTCAGGATGAACACCCTCGGCCGCAGACCACACTAGAACAGATGGCCAAACTGCCTCC
TGTCTTCAAGAAGGGAGGGACTGTTACTGCTGCCAATGCTTCGGGTGTATCTGACGGTGCTGCTGCATTGGTGATTGC
AAGTGAAGATGCGCTGAAAGAGCACAAGCTCACTCCTTTGGCCAGAATTGTAGCCTATCATGTGTCAGGCTGTGACCC

GAGCATTATGGGAATTGGTCCCGTTCCAGCTATCACAGAAGCTCTTAAAAAAGCCGGCCTGACTCTCAACGACATGGA
TGTAGTTGAGGTGAATGAGGCTTTTGCCTCTCAGTACCTGGCTGTCGCTAAGGCCCTCGGACTGGACCCAGAGAAAAG
CAACGTTAATGGTGGCGCTATTGCCATAGGACATCCTCTCGGTGCTTCTGGAGCACGCATCACTGCTCACCTGGTTCA
TGAGCTCAGGCGACGAGGAGGCAAGTACGCTGTTGGCTCTGCCTGCATCGGCGGTGGTCAGGGTATCGCCATTGTTCT
CGAGAAATACTGACTGAAGAAACTGCCAGTATCGGATGAATTCCTGTCGAAGAATGTCCCACACTCACATGAAATAGA
ATTAAGTTGACACGTGTTAAAAAGAGAATTTTTTCTGCTTACTCTTGTTGCTTATTTCAGCCTATTTATACAAAAATA
CACACTAAATGTTTCAGTCAAGGTTTGCCTTGGGAGCCCTGTTAAGATAAAGAGCTAACTCATGTTTCTGATCTTTGT
CCCAGAGTACAGCAAGAGTCTGTTTCTGTGTGTGCCTGAAATACAGTGGTAGCTCCTTGGTCACATTAACCCATGTAG
CAAATTATTAACTCATAAAGTACAGTTTTTTGTTGGGTTATAGCGAGGTTTACAGGAACTCTGAAATATGATGTTTGC
CTTAAAATGCTCTCTATCACGCCTTTATCAGGGATTAGTACTGTGATGGCTCCCGCAAAAGACTGATTTTGTATTTCT
ATGTCAGAGAATCTCAATAAATTGCATATTTTAACGTAAAAAAAAAAAA

>Sequ21126SNP2
TTGACATCTGAATTAGCAATAATGATTTTTCATTGTTTCATTGTTGTGATCGTTGACAACGTTGGAAAACAGTCGTAA
ACATTAGCAAAAAGAATTAGTAATAGTTGTCACTCACGTAGCTGTAAAGTGTTTTTTTAAGCCTCTTTTAGCTCCTCA
ACAGTGTTTTTCTGCCCCTTTTCCTATTAAATCACATTAAAGAGTCTGGTTAAGGTTGGCAGTTAAAGAGCTTAGGTT
AGTTATTGTTTATTTTAGTAGGTAAAATAGTTTCATGTTAGGTAACCGTTCTTCAGTAACTAACCAGTTGTTAGTTGG
ACAATGAGACTAAATGTAAACAGTGTCAGACGTAGAAAAATCCCGATAAACAAAGAAAAAATCTGAAAGAGCTGAAGAT
AAAAGACAAGATAAAAGTATTCCAAACTTTACCACTAAAATCTGATAAAGAAATTCTGTTGAGAGTTTTTATTTTTAT
TTCAGATATTTCATCTATTTATTTCCAGTTCAGGACAATATCTGACATTTTATCTTGAAATCTTCTCAGTTTGGTGCT
GAGACTAAAAGGTTTTTGTGATCGTTATTGAAAATATTGAAGCTGAAGCCGTGTCTGTGTTTCCCGCCCAAACTGAGC
CACATGGTAACCTCATTCATCCCAGTGTCAGTCATTTTATTGTAACTGTGTCTCTATGACTTCCTGTAAACCGCAGTC
TTCCATCCATAACTGTAGATTAGACAATCGATTAGGTGATAAGTAGCTTAATGAACTAGTAGCTGTGTTAACGATAGT
AACGACCCTCGGGGCCCGACGTTAACGAGCCACCGGCCAAAGACTCTGCGGAGTCGATGATGAAAGAGTTAACTCTGG
GCCCCGATGATGTCGCCTGTGTTTCATTGACCTTTGACCCCTGCGAGGGTCTCCATGCTGTAAACCACTGTGATCACT
GTGCTCGTTCACCAACGACTAAACCAATGGCAATAAAACCAGCGTCATGAACAAAAACAAAAAAAAAAAAAACCGAACC
TACCCT

>Sequ21147EST2
CACATTGACTCCGACACTGGCTTAGACGCGCGTCTGGGTAAAAAGAGGCATAGGCGCAGGACCTCCAAGAAGAAGCGC
AACTGGAAGCCTTATTACAAACTTCCTGGGAGGAGAGGAAAGCCCTGGAAGAGAGAGAGACGGCCAGGGCTTCCCGAT
TGAGAGAGGAGATGTTTGCCAAAGGGCTCCCAGTGGCCCCCTATAACACCACACAGTTCCTGATGGACGAGCATGACC
GGGAGGAGCCCGACCTGAACACCGAGACCGGGGTCAGGCGGCCCTCGGGGGTAGGCAGTCGCATGGAGGACACTGGCA
GCGAGGAGGACCTCTTCGACAATGAAGAGGAGGACGATGATGACGGCAGCGGCGGA GGCAGCGATGGTATCGGGAGGC
CCGGGAACGCAGGTGGGGAGTTTCTCCAGAGAGTACTTTTCCGAGACCTACGAGATGTATCACGTAGAGAGCCTGCAG
AATATGACCAAGCAGGAGCTGGTGCAGGAGTACCTGGAGCTGGAGAAGTGTATGTCCCGGCTGGAGGAGGAAAACAAC
CGGCTGAGGCGCGCCGTGGAGCCCGGGGGTCTGACCGTGGAGAGCTCCCTGGTCCGGCTCAGAGAGCTGGAGAGGGAA
CTGGAGAGACTGAGGGCCCAAAACACGGAGCTCCTTCTGCAGAGCCAGCCGAGCAAGGACAGGGCCCAGGTCGCTACC
AACTAGAGGACACCAGTCAAGTTGGGAAAACCCCCTTTGAAAGAGGTAAAAACACGGGACTACTGCATTTTTAAAAAA
AATGTCATAATTTTTCTGTAACCAGTTTGTCATCTGGACGATGAAGTCCTTGCATTGCAATGCAATACTTGACGTTTC
TTTTCTATTTGGACTGTAAGGTTGTCCTGTTCAATTTTTTTCCAAGAAGAAATGTAAATATTTCAAAATAAGATTCTT
TTTATAAAGAGAATGTATTTGACAAAACAAGAATATTTTGTTTCCCTGGTCAACTTCGTATGTGTCCATTTAAATAAT
ACTGTTTAATTTTTGAATTTAAAAACAAACATTGTATGTTGCACCCCCTCCCCGCCCCCCGAAGAAACAAACCAACAA
ACAAACAGAACTGAATAACAAATTACCGTTAATTGGGGCAGCTTGCAAAGACTGTTTTGCAGAAGTAAATTCCAGAGT
GAACATTTTAACAGTTGTGTGTTAACATAAGTTCAAATAAAATTTTGAAAT

>Sequ21165SNP2
CAGCACGAAGCTCTTCACACAAGAATTATTACTGATCCACTGCTTTATTTTAATTAGCAGTGACGTTTGAGCTTCATT
TAAACTCGTGGTTTTCTGATGATTGTTTCTTCAATAAGACACATCAGGAATTAAAACTGATGGTGCTGTGATTTAATT
TATTTACTGTCTGCAGGTAATAGATGATATTTCAGCAGCACAACCAGCTCATAAGGTTCTGTTTTATTTAATGAGGGA
ATCAGTTACAAAGTGTGAATCCTACTGAAACACATGCAGTGTCTGGATTCATCATATCTCAAGTGTTACCACTGATCA
GGCTGCTCCAGTCTTTAATTTATTCATTTCCATAGTCCGTCTACAGCAGCTCAAATATCAGGGTTTTTATTTCTAATA
TCCTTTTTCCTTGTGACACTTTCAGGCGTGTTCATGTTTTTATGTGTGTGCTCTAGTGTCAGTTTTCAAGTAGGGAAA
ATAACTAACTATGAAATGTTCACCTGCAAAAGTGAACAAATAAAAGAAAAAATAACTCATCAGCAGCTGGTAGAGACAC
AGATTTACAATAAACACCTGATTTTAGGACAAATCACGGGTGAGTGAGGAGCCACACTGACTGACCTGAAAGTGTTTT
GAGTTTTGAGAATTCAGACGTTTTCACTGCCTCGTTTCTCGGCCTCTTTTATGTGCAAAATGTATGTGTGTTTTTTTT
CAGGCATGTGAGGATCTGCTGTTAACTCGTCTGTTCTGGCTCTTTCTCGTGTTTTGCACTCGCCCTGTGTGAAGAATA
TCTGCCTTAAAGTATGCAGTGGAAGTCATACATATGTTTGAGATCACTGATGTTTGCTTTGAGTATTTTATTTTTGTT
TGCTTGTCTAAT

>Sequ21169EST2
ATGGGGAGAGTTGTTGTCGGAGAGGGAGGAGGAAGGCTCAGAGTAGACAGAAGGGAGAGCTAGAGTGGGAATTAATAG
CACATTAAGTAGGAGGAAATATAAAATTTAGCATGGCATCGGGGGACACCCTGTACATAGAAACGGATGGGTCAGAAA
TGCCAGCCGAAATAGTGGAACTGCACGAAATCGAAGTAGAGACAATCGAGACAACAGTTGTTGGAGACGACGGTGAAC
ACCAGCCTATGATCGCTTTACAGCCTCTTGACACGGATGATCCGAACTCGATTCACCCGCACCAGGAGGTGATATTGG
TGCAAAACAAGAGAAGAGGTGGTGGGCGAGGATGACTCTGAACTGCACACAGATGACGGTTTCGAGGACCAAATCCTC
ATCCCAGTGCCCGCCGTGGAAGAGGACTATATCGAACAGACTCTGGTTACTGTCGCTGGGAAAAGCTCATCGACAGGC
CGGATGAAGAAGGCTGGAAGCGGAAAGAAAGCCGGCAAGAGCTACCTAAGCGGCGCGGAGGCTGGTGGAAGAAAATGG
GAACAGAAGCAGGTGCAAATAAAGACACTGGAGGGGGAGTTTTCTGTTACAATGTGGGCATCGGACATTGACCATGAG
TCAGTGGTGGAAGAGCAGATCGTCGGGGAGAACTCCCCTCCAGATTACTCCGAGTACATGACAGGGAAGAAGTTGCCC

CCTGGTGGCATCCCGGGGATCGACCTATCAGACCCCAAACAGCTGGCTGAATTTGCCAGAATGAAGCCCAGGAAAGTC
AAAGAGGACGATGCTCCCCGGACGATAGCTTGCCCTCGTAAAGGCTGCACAAAGATGTTCAGGGATAACTCAGCCATG
AGGAAGCATCTCCACACCCACGGACCCCGTGTTCACGTCTGCGCCGAATGCGGCAAGGCCTTTGTGAGAGCTCC

>Sequ21193SNP2
ACTAGTAGGGTAGGTTCGGTTTTTTTTTTTTTAATTATTATTATGGTTTATTACTCATCACAAATGTCATTATCATCA
TCAATTAAATACATCAGTTGTAGATCCGAAGGAATGAATTCAATTAGCTATGATTTTATTTTCACTTTCGCCAGAGAG
AACTGACATGTAAACTTAAAAACACAACAGACATTAACGGCATGGGAGGAAAGAAACAGCTGAAATGCTTTTGTCGAT
GTTTGCATCAGTAGGGTCATTCCCAATCAACTGAAATCAAGAGCGATGATTTCAGGATCAATGCAGAAATGCACTAAA
GGACCTGATAGAAGTGGATTTCTTGATTCGTCCTCAATATTGCAGAGCTAAGTGCCGATTCCACAGGAGGAAATCACA
AGCTAGCAGGAGATGAATTCGATCATCGCGACATCTCGAGCACCTCTGAACAATGAGAGAAATGATGTTATTTTCAAG
ACATGAGCTGCGAGGACGTATTGCTCATAAAGTATTGTAGTCCACGGGCCTGAAAGTGTGCTCAGAAGAAAAAAAAAA
ACCTAACCGAAGCACAAAATTCATTTTTCCCATGGTACCCATGTGATCATCACAATCAGAGGAGAAATCAGTGCTGCT
GTCACCACACACACTATCAGTCATGATGGAGGAAGGTTTGCATAGATGTGTTCAGATTTCCCTCACTAACACTACACT
AATCTGTGCCTAGAGATGCACTGGCGTCAAGATAGAGGAGGGAGTGAGTGTGTGATATTTATACATTTCAATGACAGC
CATGTAAAGACTTGAAATAAGTGAAAAACAGTGACTATGGGAGGACATGTTGGCTACAATTACACGCAAGTCCCTCTA
ATCCTTTAACTATGAAAACAGTACAGTTAAAGAGCATCAATGTTTTTTTGCCATCAAATAGTTTTACGCTAACTACCT
GGCACTATTCATCAT

>Sequ21204SNP2
GGTAGGTCGGTTTTTTTTTTTTTTAGTTAGTAAGTAATTTATATTAAAATGTAAGTACATGAACAGGTTTGCTCCATAA
TGAATCATCTCAAATTGAATCAAACAAACCACTTCTACACATCTGTTCTGTGCAAAAGCCCACTGAAAACAAATCATA
TTTTTATTTCCCTTCGCGTTTTAGAGATAGCAAATGAAGAAAGAGGTTTCATATGAATTAAATACAGGGTGTTCTGGC
AATAAACTCTTTGACTTCATTGTTATGACTTCCTCAAAACAACAGCATTAAGTGAGATAACAGCACAAAAATGGAACC
ACAAGCAGCAAATATCTTTCACGTGCAATGAAAATATAGCTCACTCTTAATTTATGCAGTTCATGTGAGTAGTTCTAG
GGAAACTAATACCAAATATGTTTAGAATAAACATACAGCGGTGAAGACATACAGCTGGAGCTATAACCTCAGAAGAGA
CTCGTTTCACTTGATTCCAGGACTTTAGGGAGCGGCACAGGTTTGAGATAGGCTACGTTGCTCTTTTTAATCTCACTT
AGGAGCTGATCACGTGGCGTGAGATCAACTTTTGGTGGCACTCGTCTGCGAGGGACGGGCTTCAAAGTCTTAATCACA
GCAGTCAGGTTGGGTTTCTCATCCGTCATCTCTCGACTGCTTGCATCCTTCACACTGGAAGTCCTCCTCAGTTGGAAC
CTTTCTGGTCTCCTGTCATCCCTTGGGTCCAGATCCAGAAACGGGTCACGTTTCTTGGGCGTCCTCTTCAGCTGGACG
TCCCTTAGTGAATTTGTTGGTTCGGCACTGGATTGTTTGGGAATTCTTTTTGTTTGAGCTGCATCTGAGGCTCTGGCT
GCTCTGTCTGGTGCTGCATTTCAGGCAAGGCGCTCAGGTCTGTTGAGGGTGGGACAAGCCCATGCTCCTGCAAGAGTT
CTAGGGGCGGTATGTACCCCAGCATCTGAAGTAAACCAGGAGGCAGGTTTAGGGTGTTCTCATACAGTCTGCATCAGC
TCCTTTTGCTCCTTCAGTTTGCTGCTGTTTTTGCTCCTCCTTCCTCACCTGCCTCTGACGGTCGAGATTCCTGGTTAA
AATATTGGTCACCACCATCCTGGGCCCTGGCAGCTCAAAGTGGTAGCCCATCTTCAAAAGGGT

>Sequ21258SNP3
GCCAATCAGAGCTGACCAGGAAGGAAGTGTCACCCATTGCAATAACACGAAAAGTTCCTTCCTTATAATTCACTGACC
GCGTCTACACGTCCTCGTGCTCTTTTCCAGAATCTTGTGGGCGGGAAACTTTAGGAACGTTTCAGAGTAAAAGATGGG
ATACATGATGGTAGAGGAGCACAGCTCCACTCTGCTCCACCTGAAGAGGTCCCCAGGTCTCCGGTCCTGGTCCCTCCT
GGTCGGTATAGTGTCAGTTGGGTTGGCAGCTGCGTACTACAGCTCAGACAGCGTCCTGTGGAAACTCTTCTACGTCAC
CGGCTGTCTGTTTGTGGCCATGCAGAACATGGAGGAATGGGAGGATGCCATCTTCGACAAGACCAGGAACCTGATCGA
GCTCAAGACCATGAGTCTGTACGCTTCAGTCCTGACTCTGTGGAGGAAGGGCCAGGAGAAAATCGTGCTGGACTTGAC
TCAGCTGTGTGACGTCTGCGTCCAGGAGGAGAGGGTCCGGTACCTGGGGAAGGGTTACCTGCTGATGCTGCGTTTGGC
CGCAGGCTTCTCCTACCCCCTCACTCAGAGCGCCACGCTGGGAGGACGCAGTGATGTGGAGGCCGTGGCCGCTCTGCT
GAAGCGTTTCTTGGGGCTGGAGGAGCAGCAGCATCTCAGGCAACAGGAGGAAGACTATGGAGAGGAGGAAGACTCTCT
GGACAACAGCAGCGATGAAGCTGATGAAGCTGATGAAGCTGATGAAGCTGATGAACTCTGAGACCTGTAACTGTAGCT
GTGAGTGGATTGAGTTGAGGCATTATGACTGAGGCTTTAGAGGGCGTGGCTTCAGTGATGACTTCATTACATGTTATA
CAGAAAGCATGTGCATGTGACACTGAGGGTGTAGGCTTTTTAAATGGCTCCTTACACACTTTCTTCTGTTGTTTCTAT
CTGAGTTCAGGTTATAACAATGAAACATTTTGAACAAGGATGAAACATCCTCCTCTTGACGATCTGGAGACAGAGACT
GAAGCTCATCAGGTCTAATGACTGTGTGAGGATCCAGATCCACATTAACTGTATTAACTGTGCCTTTTACTCAACTAA
TGACTGTTTATGTGGAAGCTGTTCCCACGGTTCTCTGGACCCCCCCACTAGCTGACGCTCGTCTATTCTAGACTGTAA
ACTGTTTCAGACATGAAGGGCGAGATGAGACGAACTCTTCAGCTGCTCTGTCTCTCAACATGTTAAATATGAACCAAA
GACCGAGTCTCAGGAAACACTTTGGTCCAAGGTTTTATCCGTTTATGTAACTGTTTGTTTGTGTGAACTGAATCAGGG
CTGATGACAAATG

>Sequ21265EST2
CAACAGTACAGACTGATTGAAAAGTGGCCGGAACGGCGTTCAGTGGCTGGTGAAGTGTTTGTCGATGCTTTGCCATAT
TTTGACCAAGGTTATGATGCAGCAGGTGTGAGAGAAGCGGCTGCAGCGCTGGTTGAGGAGGAGACCAGAAGATATCGA
CCAACCAAGAACTACTTGAGCTACCTGCCCACACCTGACTTCTCTACTTTTGAGACAGAAATTATGAGGAATGAATTT
GAGCGGCTCGCTGCTCGGCAGCCCATGGATCTCCTGAGCATGAAGAGATACGAGCTGCCCGCGCCGTCAGCTGGACAG
AAGAACGACATCACAGCGTGGCAGGAGTGCGTCAACAACTCGATGGCCCAGCTAGAGCACCAGGCGGTCCGCATCGAG
AACCTGGAGCTCATGTCACAATATGGAACCAACGCGTGGAAAGTCTACAATGATAACTTGGCCTTCATGATCGAGATG
GCACAAAAGGAACTGCAGAAATTCAGGAAACAAATTCAGGATATGAACTGGCAGCGTAAGAACGATCAGTTAGCAGGA
GGAGCTAAACCGAGAGAGCTGGAGTCAAACTGGGTGTCTCTGGTCAGCAAGAACTACGAGATCGAGCGGGCCATTGTC
CAGCTGGAGAACGAAGTCACCCAGCTCAGACAACAGCAGGGGGAAGAGAACAAGGAGAATATCCGACAGGACTTCTAG
AGCGGCAGCAGCTTTCATCAGGTATCATAAGACTTACAGGACTAAGGATGGAACGTTATCCATACGGAGGAGAGTTGA
TGTGAAACTGCATCATCCAATAACGTTGACATTCAGATGGAGCTGCCCAGAGACCTCAGTGACAGCACACATTTTTAC
CAGACCTTATTCTAGAGTAAAAACAGTGGTGGTTATGGTATAAAGCGGCTTTTTGAGTGATTTTTATTATCTTCCAAG
GATCACAGATGTTTAAAGCGAGACTATGTACAGTTTGCTAAAGGATGGAACACGTTCACCTTCTTACAAATGGAAAGT

TGAATTGAAAAGCAACTAGAGATCATCTTGTGATGATGTTGACTTTCTGACTCAGGAGTAGTAAGTGGTCATTACTAC
ATTAGTACATCAGAACTCTGTACTCATGGGTCGGACCACACCCATCTTCAAACTAAGCCTTCATCTTGATCTCACGCT
GGACTACACACCTGTAAAGTTTTGTGACT

>Sequ21289SNP2
GGCTCCTGCCTGTTCCAGACGTTTTCACACGATGGAACCAAGAGGAGTAGGTTCTTTGCATGCATGTATTATGTGTCA
AAAAAATATAATCTCTTACCTTTTGCATCTTTAGGTGGTTGTAAAAAGAGAAACCCTCCGAAAATTCATTTTAGTATT
GTTGCATGGGATGTACAGTATGTGACTCATCAGACCATTAGTTCACCTCCAGTAATGTGGGAGCATGCTTCCCTCCAT
TTGGTACACGTGTATGTGTGACACTTGAGTTTTTGAAAGATAGCATTTGGGTTGCAGTTGGCTAATTACCGAGAAAGA
CATAAACACCGTATTATGTTTTTGAGAACCAATATATTTACAGAGCATCATCCCCCTTATGATTTTATTTGTACTGCT
GCCACTTAATCTGGAGGAGAGGCAAAAATTACCGCTGTAATTAGGCACAGTACTCATGCATTATTCCCCACAACGATC
CCAAATTGGCTTCTTTTTTCAGGCTGCTTCTTTGATTTAGACTTAAATAACTTTACTCTGTACATATCAACATGTTTA
CAGAAATGAAGAGTACTGCAAAATGACTACAGTCTTGGCTTTTGACCCTGATGCTGGGAGCTCACTCTCTGGGCCGGG
GACCTTGTGAGAGGGGCTCCACTTCCAATGCAGCAAAAAAAAAAGAGCAGGGGAAGAAAAAGCCTCATATATGTTTACT
GGACACACTGCATTCCTGAAACTTTTTTGTAAGGAGAAAAACTTTTTATTGAACGGTCTCATCTTCTAGTTTGCAGCG
ATCATTAAAGATGCAGGACCCTCAGGAGAGGTGCTTGATGAAGCTTTTGAAAGAGCGTTTCGGTGACAGAGGCAGCAG
CTGGTTGATGATGTAGGCTAAAGTTAATGCGGAAATTCTCTTTCAACAGTTTTGCTTCCCCATTTCTAGGCTATGCAA
GCACGGACCGAGTGTCTGCTTGTAGTCCACTCACGGCAGTGGAGGTTGCAGAAGCTTTTCACTGTTGTTGTCGATAAC
ATAGCAGAAAACCAACCATCACATTATTAATGTCCATATTATTTATACATTTCAGTCCAGTCACCATGTCAGATTCTT
TGTCTAGTAAATGTTTATATCAAATACGAAAAATCATAATACAAGTGACTGTATTCTTTTATACATGTGGCATGTGTT
GCACCCAAACTTAATTCTATAGTGATTAAAATGAATAAACCATCAACAGGACTTTTTTACCTAAAAAAAAAAAAAAAA
CCGACCTACCCTAACTAAGT

>Sequ21305EST2
AAACTCGTCAAAGCTGGAGCTCAGATCGTGGGCATTAACTGCCACTTTGACCCGGAGACCTGTGTGAAGACTGTGAAG
ATGATGAAGGAGGGCGTGGAGAAGGCCGGACTGAAGGCTCACTACATGAGCCAGCCGCTGGCCTACCACACTCCTGAC
TGCAACCGCCAGGGCTTCATTGATCTGCCAGAGTTCCCTTTCAGTCTGGAGCCGAGGATCCTGACCAGATGGGACATG
CAGAAATACGCCCGGGAAGCGTACAACGCCGGCATTCATTACATCGGAGGTTGCTGTGGATTTGAACCCTACCACATC
CGTGCCCTGGCTGAGGAGCTGGCAGCCGAGAGGGGCTTCATACCTGCTGGATCGGAGAAGCACGGCATCTGGGGCAGT
GGCCTGGAGATGCACACGAAGCCTTGGGTTAGAGCAAGGGCTCGTCGTGACTACTGGGAGAAGCTGAAGCCTGCGTCT
GGCCGTCCCTTCTGCCCCTCCATGGCCACACCTGACGGCTGGGGCGTCACCAAAGGCCACGCCGACCTGATGCAGCAG
AAGGAGGCCACCTCCCAGGAGCAGCTGAAGGCTCTCTTTGACAAGGCCAGCAAAAGTCACTGAGCTCCTCCTCCGATG
TGTCTGTTTGGAAGAAGCAAAGGGGTGGCGTGTGAGAGGCGAGCTCACACATTCCCTTAAGTTTACATACCAAAATAA
AGATCGAAATGCTGAAGTTATTTCTGTCTCTTCTAAGGACCATTTACACTCACATTTCAAATAAGCTTTAGCATTAGT
GCCTGTTTAAGTAGGATGATCACATCTGTGAGCCGTGTTACTGACTACAAAGCTCAAACAGAGCAGCTGTGAATGCAC
CTGAGTGACGCCTTAAACACTCCGAAAGTGCTTGTTGTCTTATAAAAGAACTGAATCATGTGTGGCAATAAACTTCCA
GTTAGAAAACGAAAAAAAAAAAAAAAACCGAACCTACCTA

>Sequ21371SNP2
AAGTTCAGAGCCGAAATGTCCTCTAAGAGCACTGTGCAGGTGCCCTGTGATCTTGACACCTGTTATGAATGGGAGACC
TGCTCTGCATCTAAGAAGTGTGTCTGCAGATCTATCCGGGAGTGCCCCAGAGATCAACAGCACACGTTCTGTGTGAAG
CTGAAAGGGATCCAGACGACTCGCAGTCTGGAACTTTGTCCTATGGCCTCTCCTCAAATGTGCCAGCCGTGAGTTTGA
GATTGTCAATGAAGGCGCGTGTGTGTCCAGTTGATCTGTGCCATAACACTCAACACTCAAATAATGTTCCACCACTGC
TGGAAAAACTGAAAATAACTACACAGTTTAAAATACATATGATTTCACTAAAGGCAGCTGCACTGCACTGTGTACTGG
AAACACAGATTAAGACACTATAGACATATAGTACACCATGTTACTCTTTTAGAAAATACATTATTTTTATAAAGGCAA
ACTGTACTGGAAAGCAGGTATACCAAAATCCAAGCACCTGACACATCTAAGAGCCAAAAGCACACTTAATGATGGGTG
ATGGAAAGCCAAGTCACATAACTGGGCCTTAAACAGGTTGTTTAACATGTGGTACACAAATTCATGAAAGCACAACCA
TCTTTAAACTTGAAGATATTGTGGAAGCAAAACACCGTGACAGTGCTTTAAAAGGTCTGTAGTTGATTTGCCAAGAAA
GAGAAAAAAACATGAATTTGATGATTCAGTAAATCTCCTAACTTTTTTGACCTGATCTGAGCCACTTTAGTCTGTCGA
GTCTAATTATAGGAATAGAGTTGAATATTTACTTGGCTCAATGTTCTTCAC

>Sequ21439EST2
TGCAGATCAACTCCAGTAATCCTACCTGGTCCAGACCCACACTCTCCAACATGTCGTTCAGCACCAGATCCTACGGCC
AGAAGACCTTGAGCGTGTACGGCGGAGCAGGAGGCCGTGGCACCCGCATCTCCTCATCCCAGATGAGCTATGCACCAT
CACCTGCAAGCTTCAACCTGGCTGACGGCATAGACCTCCACGTCAGTGCCAACGAGAAGGCCACCATGCAGAACCTGA
ACGACCGTCTGGCCTCCTACCTGGAGAAGGTCCGCACCCCTGGAGAAGGAGAACGACCACCTGGACAAGCAGATCAGA
GAGTGGTACCAGAACAAAACCGTCATCTGCCATGACTACACCAGCTACTTTGCCATCATCGATGACCTGAAGGACAAG
ATTCGTATTGCTTCCAGGCTCAATGCCAAGACAGTCCTGGACATTGACAACGCAAAACTGGCTGCTGATGATTTCAAG
ATGAAGTATGAGAACGAGCTGGCCATGAGGATGGCTGTGGAGGCGGACATCTCTGGACTGAAGAGGGTGCTGGATGAC
ATGAACCTGGCCAGGATGGATCTGGAGAGTCAGTATGAGGCCCTGAAGGATGAGCTCATCATGCTCAAGAGGAACCAC
GAAGAGGAGATGGTTCTGCTGAGGAGTCAAATGGGCGGACAGGTTAATGTGGCCGTGGACGCTACTCCCTCTACAGAC
CTGAACCAGGTCATGACAGAGATCAGAGATCACTACGAGGGAGTCATCGCCAAGAACCGCAAAGAACTGGAGTCGTGG
TATCAGAACAAGATTTCAGCGGTGGAGCAGGACGTGATCACACACACACAGAGATTCTGGTTACATCCCGCACAGAGATT
AAAGACCTGAAGAGCACCCTCCAGAGGCTTCAGATTGAACTGCAGTCCCATTTGAGCATGAAAGCGTCTCTGGAGGGC
ACCCTGGCAGAGACTCAGGCACGCTACGCCGCACAGCTAGCAAGCCTCCAGAACATGGTCACAAGCCTGGAGGCTCAG
CTGTCCCAGCTCCACGCCAACATCTCCAGCAACAAGCAGGATTACGACATGCTGCTGGACCTGAAGACCCGGCTGGAG
CTGGAGATCGCAGAGTACAGGAGGTTGCTGGATGGGGAGGATGACAGCTCAAAACAAGTGGTCACAAAGGTCATCACA
GTGGTGGAGACAGTTGTGGATGGAAAGGTGGTCGAGAGCAGCAAGACTGTTGATGTGGATGTAGATCAGATTGAGTGA
AAACATTTGAAGCGTTAGCAAAAATCAATAAAGATCATGCAGTTAGAAACAAAAAAAAAAAAAAAACGAACCTACCTAC
TAA

>Sequ21520EST2
GTGTATATACATGTTTTATTGTACAAAACATGTACCATTGCTCTCTACCTGTCAGATTAAGCATCAGGGCGGTAGAGT
TTGAATTTTATCTGTATTAATTTTACTGGCCCTCAAGTAATTATCAGCTTATATTTCTGGAAAAAGGACAAATAAGAA
AAGGAATGTGAAATAGGTCATGTCACTATTTGTGTCTTGCATCCCTGTTTACCACCATAATTGACTGAAGAGGCAGGA
GTCTCCCACTGATCTTCCCGAGATGAAAAGATTTTCATTAAATGTACTCCTTGTTCTTGCTTGCTTTTGTGTGTACAC
GTATTATGGTTGTTTTACAGTATAGAAACCTGCTGCTACTGTGTGGTGGAATATCAAAGCCTGTGGTTCACTGTAGGT
CACTAATTTACTCCTTGTTACACTGTTCACTGTGTGTATGCTCTGTGTGTGAACTCGACGCCATCTCCACAGTCCTTT
GACCTTCTCCGTGCATGGTGTAACAATGACTGGAATTGAGTGGAGTGCTGCGTTCAAAGACGCTCACCTCAGGGTTTG
AAGAGCCACTATTAATTAAGCAGGACTACACAAATGGTATCCTGTCGTTGAAGCCACTATTAATGACAAGTATGGGAA
ACTCAAGAGGACATCTTAGCAATAACTTGATTTTCCGACAGGCTAGAAGTTTCACAACAACCTGTGTTCACAATTCAG
TGTCTTGTAAAACCTCATGAGCTTGAATTTTTTTTTTTTTTTTAGTTGAGTCATGTAATATCATTCCATGTTACTGGAT
TCAGTACATTTCCAGGTTTGCCTGTGAACTAGAGATGTCTTTTTAGATGAAGGGTGGACTTTGTAAAAGGCTGGACGG
CCGATGTATTTTCACCCGGTCCTCACTGACTTGCAGTTCTTCCCATGTTAAGGAACAAACCCATCAACCCATTGTATC
ATTAAAAGTGCTGTACTTACCAAAAAAAAAAAAAAAAAACCGACCTACCTACTAAGT

>Sequ21581SNP2
TGTAGAGGAGAAGATGGATGCCCATCAGATGAGCATTTCAAAACCCATCTGTCTTCGTACCCCCCCCCCTCTCTTTTT
ATTCTTTTGCCTTGCCCCCTTCTCTCTTTTCTCCCGCTGTCCTTCCCTCTCATCTTCAACCTCTGCTTGTCCGCCCCC
TTCAACTTTTTCCACTTCTGTTTTTGGCTTCTCTTTATCACGTGGCGGGCTTGAACAAGGCCTTATATAAAACTGTGC
TGCCGCTGAACGCCTTGTATTTCAGCACACATCTATACAGTACCAATGGTCTTGCTATTGATTAGTGTTTCTGTGACC
TTTTCTTCTCACATTGTAAAGAAAAAAAAAAAAATACAGCTTTTAATTTTCCTGTCTGAGTCACTGCAGGGGTGAAAGC
AGTCTGTCCCCGCCATCCATTTAATTGTGGTTAAGAGACAATGAGCTGGTCTCAATGTTCAGATCGATCTCAGTCCAT
CACAGTCTCATCCAATCAGCACTCGAATGACTCTTAAGTTCTCCGCTCTCCCTTCCTCGCTTTGTCCGACTTTCTCGC
TTCCCTTCCTTTCTTTCCTCTGTCCTCGTTTCCCGATGAAAATGCAATCAGAGGAGAGCATCTGAGGGGATGAAACTT
TGAATTTATCTCTTTACAGTTTTCCAGGAGGAGGCAAAGAAAGCAGGGGAAGAAATAGGGAATTGATGAGCTGAGGAA
GTGCCTGATCGAGTAAGTAATGAGGGAAATCGAGGAAGTGTTTAGTGACCGAAGGCCCCTCCTGTCTAAGCTGTGACA
TCCTCTTAAACATATGATTGGTCCCTCTGGTGTGGTTGCATTATGACATTATCACCTCCTCACTGATGTTTGTGCAAG
TTAATAGTTGTGTGCACTGGATATTATAGCCTGCTGTTTAACACTGATCATGTTGATGTATATTTTTACATCTCTTTC
TAGAAATCTGTGGTCTATTTCTTGAGATGAAAAATGTTTAAAAAGAAGTGCTGTAATAAAAAGAATACATCCTTTGTT
TTGACTTTTGATATTCTGCTCAAAAGATCTTGTTATAGAAATATAATGAAAGGTGTTTTACTGTTCTGTACTGTTCTA
CACAGATTTTGGTATTCACAGGACATGTGAACTGTCTGCTTACTCATTTTTAAGAATAAATATTATAAAGGAAAACTA
GAAAAAAAAAAAAAAAAACTAACGTAAGACGTA

>Sequ21587SNP2
TGTGTGAGTGAGTGAGAGAGAGTTAGTGTGAGTGCTGATGTTGCTGTCCATCTCATCCCCCTTTTCAGTCAAAATGCT
GAGGGCACACCGACCAAAATCACTCTCAACGTTTCCAGTGGGAGAGGGAATGGACAGCATCCACTTTATTTATCCATC
TTTGAAAGGAGCTGCTGAAAAAGGCCTTTCACCATTTTGGAATGAAATGTAAATTCAGGGTAGTATGGTTTACTAACC
CCCACCCCCCTTTATTAGATCTGCACTCCTTGCCTTTGTAATCCATTTATTTTATTGACTTTAATTTATCTGAAAAAA
AAAAAAGTTTACTTGAAAAGATTCTTGAGAAAACATTCCATGTTAATTTTGCCAAATTCAGCAAATAACGAAAATTGA
GTACAAGTAAATGTGTATGTGTGTGTGTGTGGGCTTTGAGTATTTTATCATTTGACTGGAGTGCTTTGCTGTGGGGAA
GTGGGGCAAAGTGGATGATGATGTCTCACGTTGGTGAATGTATCATGTGAAAAAGAAACGGTTCATTGGCCCCAGCAC
CTGGCTCTGCCCATGCAGCCCAGAGGAAGGCTTGAGCAGCTTCCTCTGGACCGGCCGGGTGGTGTTCAGAGTGTTCGT
TTGTGTCGGCTGATCGGCTGTCCAGCACCGAGTCCCTCCCACCCTTCCTTTTTATGAAGCTCTGCCTATTAAACCCCG
GCCAATGGGGCCAGGTGGAGTGGATGTTTGCCTTTGGATTGGGGTTGGACTGAGATTGCTGGAGGCCTAAAGCTGTGT
TTGTAGATTTCCTTTTCACAATCAATAAAACCATCATCAACTCAAGAAAAAAAAAAAAAAAAAAAAACCGAACCTACCCT
A

>Sequ21636EST2
CAACATAACACTGTCCAGACAGAATTTAACGTGTTTTTTTTGCGGTAGTTTGAAAACTATGACATAAATAGTTTGTTT
AAAGGTTTGTCTTGAGTCTCGTGAATAATTTAACTTGTGACAAGGAGCGTTNTAAAACTGTGGACTCAGTAAAGACTG
TGATCCACCGTGGTTTGTCTTAACCGTAGAAACTACACCTGCTAGCCTCTCCGGCTCGTTTCACGATGAATGAAGAAG
AGCTGGTGGAGTATTTCAGGGCTCAGATGAGGAAAGATCCGGACATGGCCTCCGCTGTGGCCGCTATCCGCACTCTGC
TTGAATTCCTCAAGAGAGACAAAGGTGAAACTATCCTGGGCCTGAGGGAGAGCCTGAAGTGGGCCACAAACTGCCTGA
CAGGGGTGGACTCCTCTGTGGCCGTGTCCTCAGGAGGAGAGCTCTTCCTGCGTTTCATCAGCCTCACATCACTGGAGC
ACCAGGATCTGTCGCGCTGTAAGAAGGTGATGGAGGAGAGAGGAGAACTATTTCTAGAGAAGATCTCAATGTCCAGGA
CCAAAGTTGCTAAACTCTGTCACACCTTCATCAAAGATGGCACCAAAATCCTGACTCACTCCTACTCCAGAGTCGTGC
TCAGAGTGCTGGAAAAAGCTGCAGCGGAGAAGAAACGCTTCTCTGTCTATGTGACTGAATCGCAGCCTGACTCAGCTG
GGCGACAAATGGCCGAAGCCCTGAGAAAACTCAATGTTCCAGTAACGGTGGTCCTGGATGCAGCTGTGGGGTATGTCT
TGGAGAAGGTAGATCTAGTTATTATTGGTGCAGAGGGAGTCGTGGAGAGCGGAGGAATCATCAACAAGATCGGCACTT
ATCAGATGGCGGTTTGCTCAAAAGCTCACAACAAGCCCTTCTACGTCGTGGCGGAGAGTTTCAAGTTCGTCCGTCTCT
ATCCGCTCAACCAGCAGGACGTGCCGGATAAATTCAAGTACAAAGCAGACACCCTGAAGACGGTCCATAACCTGTCAG
AGGAGCATCCGATGATTGATTACACACCTCCCTCCCTCATCACCCTCCTCTTCACCGACCTGGGAGTCCTCACCCCGT
CCGCCGTCAGCGACGAACTCATCAAGCTTTATTATAACTCCGTGCCTCACAATTCTCAAATAAAATTCTTATAGAAAA
AGACTGAAAAAAAAAAAAAAAAACCGAACCTACCCTAACT

>Sequ21644SNP2
GTAAAGTGAATATCGGAGCCTAATCACAAAAAAGAAGAAACAAACTACAGTTCATTGTGCAGTTACAACCTGCTTCAC
ATTGAAAGGTAGAGGGAAAAGCTGTTATATGTAACCTAGATGTATCTATAAAATCAATTTCAACCATGTTTGGCCCAG
TTTTAATCTATATATCTTGATGTCATTTGTTGCACAGGTTAGAGGTTCTTGCCTTATTGGTTTATTTCAGAAAATGCC

AAGATTTTTGAAAAGGATAGGGCTCAAAAAGTAGTTATTATATTGTTGTATATAAATACCACACGATATCTGAGGAAC
CAGCTCGCTCAGTAAGCTGCACTGTGTTGAAGAATACAAAACCACAACTGGATTAAAGACTGTACGATATGTCGTTGT
AGGTTTTAAGGAACACCTGCGTTTATGGACTGTTACTATTGAAGCAAAAAAAGCACATTAACAGCTAGAATGTGGATT
TGACATTTGACCTGGGTTGAATGTCGTATAAGAAATTGGGAGTTTAAGGCACAAAATGTGGGAAAAAACACAACCCGG
GTCTGATAGTTTATCTTATGGTTTCGTTGTTGTGCAAGAGCCATAGATATACCCAGTCCTAAAATTCAGCAAGCATAT
CTGGATCCAAGCGCTTCATGGCCTTACGGTGTCCATGAATGTAAAGCTGTGACTGTAAAACAATTTTTGTCTTGCTGA
ATGCGCTGATAAAACACTGGACCTGTTAATCTTACAAAGCTGGAAATACTTGTCCACAATCACCAAATTTTGGCGGCA
GAACGCAGAAAAACCTTATTTAATATTTCAAAAATGAAAACCCGAGGCACCAAAGGCAGAGAAATCAAACCAACCATA
CCTTGTGTATCTATGGCTACTGGCTTGTAAGAATACTTCATTATGTGTTGAAAATGTGAGAAATAAATCATGTTGTAT
AGAATTTTGGTAAGGAAGCCTGTAAAGTAAAATACATTTGGAGTAATATAACCTAGACAATGAAACAAAATAAACTGT
GTATTGCACACTAAAACCAACTGTAAGCAAGTATGCATGTAACTATAGACAATAAAACT

>Sequ21691SNP2
CACCCGGAAGCGGAGATTCTCAGGTAACGTTAGCTGCTGTGAACGGAGCAGTGAAGAGCTTTTAGTGAATTATTATGT
TGTAGATGAGCTGGAGGTCTGAGTCAAGATGGCGCTGGTACGAATGAGGAACGCGAAGGGCGGGAACTCCACAGAGAT
AGAGAAGAAGATTAAAAGGGGTCATGCTCTGAGGCAGGGGTCTGTGTGGAGGGGGTACCGAGGACTCGTAGGCCCTGC
TGCTGGTTGCCGTGTGTGTGTGCAGGACCCTGGTGGTACATGAGCTCTGTGGACAGTGACATCACAGCGACACTGGTC
AGCCAGGGGGAGCTGGTCTCCCCTCACCCCAGAGTCTACACCGTCCCGTGCTCCGAAGACTACGAGAACTACAAACGC
TACCCAGGATGCACCCCTCACAGATGTGGCCGTGCAGTCACAGACAGTGTGGTCAGCAGGGAGGAGGCTCAGGTGCTC
AGGAGGCTCGCTGAGAGGGGGCTGGCACTGGCCGGGTCAGAGGAGGGGCTTCCATACTGGACCTGCACTCTGGAGCGC
TGTCGTATGGGGGAAACAGTTCGTCAACATCTACAGGTATTTTGGGGATCAGATCAGGGATGTGATCACACCTGAAGA
TTTCCAGCTCTACAGAGACGTACGTGAGAGAATCCAGACGGTCATAGCTGAGACGTTTGATCTGGACCAGACTCAGAT
GTACCTCACCAAGCCAACCTTCTTCTCCAGGATCAACAGCACGGCAGCCCAGACCCAGCACGACGAGTACTGGCACCC
ACACATAGACAAGGTAACCTACGGCTCCTTCGACTACACCTGTCTCCTCTACCTGTCTGACTACGGCTCCGACTTCAC
CGGAGGAAGATTTGTCTTCATGGACCAGAATGGAAACCAGACAGTGGAACCCCGGGCAGGACGAGTCTCCTTCTTCTC
CTCCGGCCCTGAGAACCTCCACCGTGTGGAGAAGGTGACGTGGGGGACGCGCTACGCCATCACCGTGTCCTTCACCTG
CGACCCTGCACACGGCATCTCTGACCCCGCCCTGCCCTGAGGCACCCTGGGTGATGGCGCCGCCCACGGAGTTGGACG
AACCCTGTAACAAAAGAGGAAGGACAGAGTGAGGGGCTGTTTGTGAAGGTTTGACTGTTCAACCTTCAACATGTGTAC
ATGTTATTTTCAGGTGTTGACTCTCACTGACTGAGAGAGTACACCTTCATCCTCTCCTGCAGTCTCTGTTCTCACTGC
TCCGTCCTCATCGCTGGTCTCAGGTCTGCCTGGTTCTTCTTCATCATTGGGGATCCAGCTCTCTTTTCTTCAGAGAGG
CTGAAGCGGGATTTCCTGTGTCGCCACATGTTTCTCATTTGAAGCACAGATATAGATCTGTTGAATCAATCCTTCGTC
ATCCTCTCCCAGCTCTGACCCAGGAGGAGTTTTCTTCTAAGAAGAACCTTTTCTCTCAGTAACGTTAACATCTACTTC
AACATGGTGGAGAACCGCTGAAATAAAACACTGACAGTTCCTGAGAGGCTCTTCTCTCTTCTCTTCTTCTGTTTTATA
TCATCATTAATCTTCTCAGTGTCAAGTCTAATATTCAGTTCTCATTAAATGAACTAAAATACAAAAAAAAAAAAAAAA
AACCGAGAACTA

>Sequ21732SNP3
GAATATGTAAAGATAAGTACAGTATGTTCAGAGCGGAGTTCAACTGTTATGAGTTGTTTATTGCTGTATATGTACATG
TACCTTCTTCTTTTTTTTTCTCTGAATGTAGGAGAATGACACGGCCTGACGTTTTGTTGCACAGCCAGAATCTTCCTGT
CTCCTCAAATGACAAACTAACAATATTATAATTGCCTGTACTAAGTGTTTGTTCCCCGGAGGGCTGTGTGACGAATTC
TTGCTGTTGTAACTCCATTCCCTTGTGACGGTGTGAGTGTGTAGCTCAGTTTTGTGGTGGTGAATGGTTGTGGTCATG
CCCTTGTGTAGTTTGGTTTCGGAGAAGGGGGGGTAAAGAAAAAAAAAAACGGCAATGGCATGTCTAATTCAGGGTTTTC
TTTAAAGCTTGTTTTTGTGGTTAGAGAAGTGAGACGGATGAACGGGTCCTTTTCATGTTCTCGCTTTTGATGTCAGGA
ACTACTGAATCTACCAATGACCTGCCCATCAAACAGGGCATTACACAAAGTAACATCAGCATTAGAATAAATCCTGAA
TAGTCACATTGTTCCTTTTTTTTATCAAATAATTTAAAGCCCCTGAAAACGTGGCTTCAGATCTTTTAACATTTAGATA
TTCATCACTAAATGAGTTAAAGGATTAGTTTGACATTTTGGGAAATTTACTTATTTGAATGTTAGATGAGCAGATAGA
TACAGCTCATGTCAGTACAGAAATATGAAGCTATAGCTAGCAGCTGGTTAGCTTAGCATAAAGACTGGAATTCAAGGG
GTAACATGTGGCCTGGTCTTGTTCAAAGGAAACAAAGTGCAGCTTTTTTGTACAAACTAAAGGAGACAAGATATTAGC
GAGCTTCAGAAGCGCCAAGTTGGATTTTGTATACCTTCTTATGTACAGAGCTTGGCTAGCTGTTTCCCTCTGTTTCCA
GTCTTTGTGCTAAGCTAAGTCTGCAGGCTCCAGTTTCATATTTATGGCACAGACACGAGTAACCAAAGTAATCTAACT
GCTTATATACAAGCATATTTTTTTTAAAATCAGGACTCAGGTTTGGTTTGACAAGATTTTAATGACACTTACTGGCCT
GACCTGTCATCAAATTTTGATCTAGATGCATTGAAGTCCTGATTGGTGGATAGAAATCTGTGACATCACCCTTTTCCA
ACTGTGTCCTGCCCACTTCAAACCAGTGAGAGCACAAACAGGAAAAACAAATACATAAATCTTATATGAAATAATCAA
AACTCCCAACTTTGGCAGAAAATGTTGTGTTAATGTAGAAGCCTGTGACTCAGTAAAACAAAAGGTTAATCGAGAAAA
TGGTTTTCAGGGCCTTTAAGGAAACTGGATCACAGCTTTTGGAAACCTCATCTGGTTTATGTATATAATGGTAATATA
CTTTCTTTTTCCCCCCATGAGGCTTTTGTAGGACTTTCTTTCTTTTTTGTGTTATGGTTATTTAAAAAAACGTTTTCA
TGCTTTAATCCTGTGTTAATGCACTGCTGTCTTCATGAAGAAGAAATGTTAGTCCGTTTTTCTCCTGGATGTTTCAGA
TTTGATGGTCTGTGTATTAATCACTTACATCAAAGTAAGAGTGGTGTCTCTTTTTCCTACTTTTCCAGCTTCTTGTTT
TCAGGGCTCAAAGATTATGTTCGATTCTGTAAACGTGGCCGACACACGATGGGTCCATACGCTGAGGTTGTGATTGCA
TTCAGACTGGAGTGTTCGTTTTTAAAAATGTGCTGCTCGTGTGGTTGTTGAGGCGACTCTTGTTTCATCGCTCCTGCT
CTGAGCAGTGTAACGACTACAGGCCACTACGTCGAGTCTCAGAGGGAGGAACAGGAGTTGTTGCCACAGTCAGTGTCA
ATGTAAGACTCCAAAAACATAAATCAATGGTGCCTTTTGTTGTAAACCAAAAAGAAGGAATAAACGGCGTTTAATCCC
CGT

>Sequ21736EST2
GGCGGAGAGATCACCTCCACCTTCGACCACCCGGAGCTGGTGAAGCTCGCCCACTGCAAGCTGATCGAGGAGGTGATG
ATCGGAGAAGACACACTCATCCACTTCTCTGGGGTCGCCATGGGTGAGGCGTGCACCGTCGTCCTGCGAGGAGCGACT
CAGCAGATTCTGGACGAGGCGGAGCGCTCGCTGCACGACGCTCTGTGTGTGTTGGCTCAGACCGTGAAGGAGCCACGC
ACCGTCTACGGAGGAGGCTGCTCTGAGATGCTGATGGCCAAGGTGGTCACTGACCTGGCCAACAGGACGCCAGGGAAG
GAGGCGGTCGCCATGGAGTCGTTCGCCAAGGCTCTGAGGATGCTGCCGACCATCATCGCCGACAACGCCGGCTACGAC

AGCGCCGACCTGGTGTCTCAGCTGAGAGCTGCTCACCAGGAGAACAAGACCACCTTTGGACTGGACATGTCTGAAGGC
ACAGTGGGCAACATGGCAGAGCTGGGGATCACAGAGTCGTTCCAGGTGAAGCGTCAGGTGCTGCTGAGCGCCTCCGAG
GCCGCAGAGATGATCCTGAGGGTCGACAACATCATCAAAGCTGCACCCAGGAAGAGAGTTCCCGACCATCATCCCTGC
TAGAGGAAGAGGAGGATGAAGAACGGGGGAGGGGATCAGTTTTGTTATTTATCACCTCTGTGTAGTTCAAATTTAAAG
AAACGCTCCGCTGTTTTTTTCTCTGTTGTTAAACTCACTGTGGAAGCACTGTACAGGCTAGCTGGCTAACGCTAGCTA
ACCGTCAGACACAACCCTAACCGCAGCGGCTCTGATGCTAATGCTAGCAGGACAAAGTGGCTAAGTGTGAAACTTATG
TAAAACGTTGGTCAGTACAGTTTGTGATGCTAACATTAGCTGAGGTCGCAGCAAGTGCTGGAGGCTGGCTATGCTAAC
GTTAGCC

>Sequ21746SNP2
CTCTTATTAAACTCTAAGGGGAACAGGGTGTCTTGGATGTCTTTACAAGTACAATGATTGTGTGTTGTGCCTGTACAG
TTGTTGTGTATTTACAGTATGCACAGATGTCATGACAGTCTGTATTTGGTCTCTTAGTAAATAAAAACAACTGACTAA
TGTTTGAATTTGTAATTCAGGGCCTCATTTATATAAAAAACGCTCCCTGTCTTTATCTTTATCCCATTGTTCGATCAT
TTTCAAATAAGTTGATTAAATTCTTTTTACATTCAGTGCTTTCTATAATGAGAAGACATTTTAACAGACTTTTTTTTA
CTTTTATTCCTTATCAAGACCCTTACTGTGCATAAATGAGGCCTGAAAATGTTTCCACCTCAGAATTCAAATGCTGCA
ACTTTGCGTCATATGGCATCACAAGTTCAGTCAAATCCTGTACGATGCATCGCTTAAAATCTCAAGGAAGCTCAACAG
TGCTGATATTTCTGACTCATGTCATAACTGCCTTGCTGCTCAAGGTTCCCTTATGAGCTGCAACCTCCACCTCCTGCT
TTAATTCAGACCAAAAAGGCCTTCAAAGCAGAGGAAATATAGTTTCCTATGTTGGTGATCTAAGTCATCCTTATATTG
GCAATACATCATCGCATTACAATACACCGCCTGGTGAGGATGTTCATTCTCAATCATTTTGTGTGTAATTTAAGTTTG
CCTTTTGCAGTCTACCTACCATACTGAGAGCCATTTTCACTGATATCTCTCAAACTTGTGTCTATTTTTGTGTGTAAA
AATGTAAATGTACATATTGGTGGTGTGTGTGTGGCAAGAATAAAAATTTACAATAAAAAACGAAAAAAAAAAACCGAAC
CTACCTAAT

>Sequ21767EST2
GAGAAGTGCGGACCCACTGACTATTTGACGTTAGCTTAAAATTCAACACTTCACTCCCGAATAAACTTTAATTTCGCT
ACCATGAAAGATGTTACCGGGTACCTCAAGCAGCAGCAGAGCAACAGCTCAACACCGGAGATGGCGGCGGAGTGGCAC
AACCTGGAGGATTTGTATAACAAAAGATTGTGGCATCAGTTGACTCTGAAGCTGACAGACTTTGTTAAAGATCCCTTC
TTCAAAACAGGAGATGGCCTCATACAGCTCTATGAAAATTTCCTCAGTGACTTCGAACACAGAATCAATCCTTTGTCC
CTTGTGGAAATTATACTGTATGTTGCCAGACAGATCTCAGAGCCTAAAGATGCCATCACTTTTCTCGAGAAGACCAAG
GAAAAGGTGAAAAGCAGCGAAGAAGCCGTCATTCTCTGCAAGACAACTATTGGCAGCCTGAAACTGGAGATCAGCGAT
CATCCTGCCACAAAGAAACTCATCGAAGAGGTTGAGGAAATGTTGAATAACTTGCCCGGGGTGACGTCAGTCCACGGC
AGGTTTTATGACCTGTCCAGCAAATACTATCGCATCATCGGGAACCACGCCTCCTACTACAAGGACGCTCTGCGCTAC
CTCGGGTGTGTGGACATTAAAGACCTTCCAGAAACAGAGAAGCAGGAGAGGGCGTTCACACTCGGACTGGCTGGACTC
TTGGGAGAAGGAGTTTACAACTTTGGAGAGCTGCTGATGCATCCTGTGCTGGAGTCTCTGAGGAACACAGACAAACAG
TGGCTCATTGATACACTTTATGCCTTCAATGGAGGCAATGTGGAGAAATTCCAGGGCTTCAAGTCTGCCTGGGGCCAA
CAGCCTGACCTTGCAGCACATGAAGCTAAACTGATGCAGAAGATCCAGCTGCTCTGTGTCATGGAGATGACTTTCACA
CGCCCTGCAAACCACAGACAGCTGACATTCACTGAGATCGCTCAGAGTGCCAAAATCCCTGTTAATGAGGTGGAGCTC
CTGGTGATGAAGGCTCTGTCTGTCGGCCTGATCAAAGGCAACATTGATGAGGTGGACCAGAAGGTGCAGATGACCTGG
GTGCAGCCCAGAGTGCTGGACCTGCAGCAGATCAAAGGTATGAAGGAGCGGTTGGACTTCTGGTGCGGTGATGTTAAG
AACATGGCCATGCTGGTGGAGCAGCAGGCTCACGACATCCTCACCTAAACAGGTTTCAGTCCACAGAAGGAAATCTCA
CTTCTTCCCAGAGATTTGTTTTTTCTATTGTTTTCTTTTTTT

>Sequ21786EST2
GCTCCTCAGAAACACCCTTCCAAGATGGCAGCCAACAGCAGCAGCACGGGTAGCAAATCTAGCAGCAGCGCCGGAGGA
AAACAGTCCGGCCCTTCAGCCGAACAGGTGGTGGCAACATTTCAGAGGATGCGGCAGGAACAACGCAGTATGGCTTCT
AAAGCTGCTGAGTTGGAGATGGAGATCAACGAGCACAGCTTAGTTATTGAAACGCTGAAAGATGTGGATCCTTCGAGG
AAATGCTTTCGTCTAGTAGGAGGAGTGTTGGTGGAGCGAACGGTAAAAGAAGTTCTACCTGCCTTGGAAAACAACAAA
GAACAGATCTCCAAAATAATCGAGTCCATCAACACACAGATGCAGACGAAAGGACGGGAACTCACAGAGTACAGGGAA
CGCTACAACATCCGGTTGGTGGGAGAGGGCGAGGGAGAGGCACAAGGCCAGTCAGCGGCGTCCTCCAGGGACAGCGAG
GGAAGCGGGTCCAAAAGCGGTGCTGGTGTTTTAGTGTCGTAATTTATAATAAAGGGCTACAGTAGGGCCTTATGATTT
CCGCAGTACTGAAAACACAGTTGGAGTCACAAAATCTAAGTTCAAAAACGGAACCCTACACAGGAAAACTTTGGATTT
GGAGATCATTAAAATTTCTGCTAAATACACCTGGCATTGAAGTAGTTTGCAACAGACACAAATTCAAAGTGTACATAG
TACTCTGCTTACGTATGTTCAGAGTGAATGCTAGTGTGTGTAAAAATACGAGTTGTTTGTCTTATATTTGCCTTCATA
TTGTGGCTTAAATTCAACAATTAAATTCTGCCTTCTTGCTCTCATTTACACACGAGTGAGTTAATGTTATTTTACTGT
CTCAGATAAAACATTTTTTCCTCCATGTAAGATATAATTAGGGAGTTCAAAATAATTTAATAACACCTAGAACATACA
AGACAAACTAGATCACATTGCATTTTGAGTAAACATGCTTTTGCATTTTTTAAACTTAAGATTTTGGACTAACAACTT
AAAAATGTGAACACATAAAAGCTGATTTCATAGGCCCCTAAAGTAGGTTGAAAGTCACCCTTGTCAAGGCAGAACTGG
TCCTAGAAGGCATTTGCCAACATGTGTTAGAGATTATACTGTAAACATGTCACTGTAGATCAAAATGAATCTTGTCAG
CTTTATTCACCTTCCCTCTGTGACACATTTTATGTGCGTCTTCAAACATAAGCCATGCATTGAAAGACTGCTGGTTTG
TATACATAATACTGATGTGAGGTCAGTTTGTATTCAGTAGTTACTAAAGCTCAAAACAGTGATGTGACCAGGGAATGC
TACAGCACACCTTAATAATAAAGTCCTGTTATTAAAAACACAACATAATGTGTTGTATTCTTTTTGATCTGTAGCGCA
TGCTTGAGCGTGGAATGGTTAAATTAAATTGTTAACAGTCTAAATCGAGGAAATGCCTCCTAGGACCTGATGGGTACT
GTCACACTGTTCATGTAGTTGTTAGGGCAAAAATGTTTTGTAATGATCATCAATTAAAAGAATGTCACAATTGAACGA
AAAAAAAAAAAAAAACCGACCTACCCTAAT

>Sequ21811SNP2
GAGATGGATACTATTCAGTCACATGCACATTTCATATTTATAGCACTGTGAAGAATGATGTCCTGCAGACTGTTGCCT
TCTTAAGGTGTTTTATGCAATAGGGTACAATTGTATTACCTTCATATATTATCAGACTAATGCATAACCTAACAGAGC
ATTCACCACTGTGGCTCTCAGCACTATACGTGACTGTGGGACCTGGATCAGTGTGCCTTACGTTGAACGGTCGCTAGA
AGTAAGCTGTTGCAAAATGAAATTGTCTTAACGTAGCGCTTTGTTGCTGTATTTTCACCCGAGTTTTCCTTTCTTTTC

TGTGAACCTTTTTGACCTTTACTGAACCACCTAAACATTCAAAAGATGGTGTTTCGATCACTTTCACAACATTTTTAC
TCGTGCTCATGTTACTATGAAGCTATTACCTCAAAGAAATGCCTGACATTTTGTCGACCATTTTTCTTCTAAACTTTA
TGAGGTATGTTGTGATCCGTGTGAGGTAGAACTTAGTTAATGTCAGTGATGCCACTTTGTTGTCTTTGTTTCCTTCTC
AAAAGTTCATAAAAACATTTCCAGTCCTATGAGCATCACCTAAACCTCGGCTCTTTAGAGATCGGTACTGTAATCATT
GTGACTTCATTTCCACCTACTTTATAAAAGAGGAAATGTTTCACCTTTGCACTGAAAACAGGGGACGACATTGCTAAA
CAAAGGTACTTGTACAGTAGATTTAATGTAAATACCATAATACTGATGATAGGTTTTGTAGTACGGAACACTGTTAGT
TATATATTTTTACCTCTGGATGAACTTAATCTGTAAAATATGGATTCCTATAATCTTTTGTACGTATTTGTAAATATT
CACTGTGCTGTATAAATTCTTTAATAAACCAAAACTGACCAATAACCGAAAAAAAAAAAAAAAACCGACCTACCCTAT

>Sequ21821SNP2
CCGACCAGAGCCTCATAGTTGAGCTGGTGGAGTACCAGGGGCAGGTGGCAGACCAGGACTCCCCCAGGTATCACTTTG
AGGACATCGCGGGCAGTAACAAATCTCTGGAGCCAGGTGCTTTTGAGGTGACCAGCGTTGTGGCTCTTCCCAAATCCG
ATGTGTCCCTGTCAGAGTGCAGCTCAGCCTGGACGCTCACTGGCACACAGTGTGTATCCAAGTTCAACGAAGAGGCGA
GGAACACAGTGACCCTTCACCTGGGGCTGTTCCGCCTGCCCCAGTTCTCCACAGAGGTCCTGATCACCTTCAATGA

>Sequ21826EST2
GTCCATTCATGTGGTGCTGATCACTGATCACCCCTGGACAGACAGGCGAGCTGTCCCTCTTCCTCTCTCTTGTATCTG
AGCAGCAGAGAAGCGACCATGCCTGGACTTCTGCTTGGAGACGAGTTCCCCAACTTCGAGGCCGACACCACCATCGGC
AAGATCAAGTTCCACGACTTCCTGGGCAGCTCGTGGGGTGTCCTGTTCTCCCACCCAAGAGACTTCACTCCTGTCTGC
ACCACTGAGTTGGCCTGCGCCGCTAAGATCAGTGATGAGTTCAAGAAACGAGGTGTGAAGATGATTGCTCTGTCCATT
GACAGTGTTGAGGATCACCGCAACTGGAGCAAGGATGTGATGGCATTTAACAATGAGGCTGACAGCACGCTGCCCTTC
CCCATCATCGCTGATGACAAGAGAGAGCTGGCTGTCCAGCTGGGCATGCTGGACCCTGATGAGAGAGACAAGGATGGA
CTGCCCCTCACTGCTCGCTGTGTCTTTGTGATCGGCCCCGACAAGAAGCTGAAGCTGTCCATCCTCTACCCCGCCACC
ACAGGGAGGAACTTTGACGAACTGCTAAGAGTTATCGACTCTCTGCAGCTCACCGCACAGAAGAAGGTTGCCACACCG
GTCGACTGGAAGCCTGGCGACAAAGTCATGGTCATTCCCACACTCTCTGAGGCGGAAGCTGCTGCTCTCTTCCCCAAC
GGTGTGACAACCAAAGAGGTACCGTCTGGGAAGAAATACTTGCGCTACACCCAGCTCTGAAGACGCACTAAGGACTTC
ATCCTGTTCTACTTTACTGCGCTTTGACCCAGAAGTCCTTTGATAATTCACGTTAGAGAACTGACCAAAATCATAGAT
TGCACTTTCCAGGTTTGGGGACGAGTGTTTTCAGTCATGCCGCGTGCACAGCAACAAACCAGGGATAATTATAGTGTC
AATGTACAGTGCCTTTATCCTCCCTCAATAAACACCCCTCTTAAACACAAAAAAAAAAAAAAAAAAACTAACGTAGA

>Sequ21898EST2
GTCGAGATCCAAAGTCTCATGACTTAATAAGGGTTTTGCAGTGTCCGCCATGACTGAGGTGAGCCATCAGCAGTGGGG
AGAGGTGTCCGGCCAGGGCAGTGTGGACCTGTGGGTCCTCCAGTCCTCCCAGGTGCGGGTGGAGGTCCTCACCCTGGG
CGCCATCATCAGGTCTGTGTCCAGCAGGAGGAAGGACGGTCAGATGGAGGACATAGTCTTGGGCTACGACAACCTGGA
AGGTTATGTGTCAGATAAGCGGTACCTGGGAGCTGTGGTGGGCCGCGTGGCCAACAGGATCGCACGGGGGCGCTTCGT
CGTGGAGGGAAAAGAGTTCCAGCTAGATATCAACAACGGGCCTAACGCACTGCATGGAGGGCTGCGGGGCTTCAACAA
GGCTGTCTGGCTTGCTACAGCAGTGGGAAGTGGTGTACAGCTGAGTCTTACCAGTCCGGATGGAGACCAGGGTTATCC
TGGAGAGGTTCAGGTCTCTGTCACCTACACCCTACAGGGGGAAACACTTACTGCTGAATACCAAGCCAGGTCGACCAA
AACACCCCCATCAACCTGACCAACCACTCCTACTTCAACCTGGCTGGACAGGGTGCAGCAGATATCTTTGACCATCAT
GTGTCCATCAGTGCTCAGTCCTACCTGCCTGTGGATGACACATCGATTCCTACAGGAGAGATCAGAGCAGTAGAGGGC
ACGCCCTTTTGACCTCAGAAAGCCTGTTCTGATTGGCTCTCGGCTGAAGGAAGTTCCAGGCCCAGGGTTTGACCACAA
CTTCTGTCTGTCATCCCCTGGAGACGGCTGGACAGAAAGACACGCTGCCAGAGTGTGTCACCCAGCCAGTGGGCGTGT
CCTGGAAGTTTCTACCAGCCAACCAGGAGTCCAGTTCTACACCGCCAACTTCCTGGATGGCTCCATCACAGGAAAGGG
CGGGGCTAGGTATGGGAAGCACAGCTCCTTCTGCCTGGAGACACAGAATTGGCCTGACGCTGTCAACCAGGTTTCATT
TCCTGACTGTCTCCTGCGCCCCGGTGAGAACTACCAACATGTCACTCGCTTCACCTTCACCACGCTGTGATCTGACCT
CCACCTGCAGGGGGACACAGAAGAAGAAAACTGGGTACCTTGAAATAGAAAGTGGAGTCCAGTAATTCATTTACTTTT
TCAGGGCAATCTTACTTCCTGTCTGTGTAACTGTAATTCAATGCTGATTATTTTTGAATGTGAAGAACGTTCTCGGTG
CTGTGACGGGTTTGTCAGGCAGAATAATGGCAGCCTGTGCAGGGTTTGGTGTGGGGCAATGACACGATGCTCTCATGG
GGATGATCTGTGATGTGATGATCCTCTGCCATATTTCTTACATTAAACCAAAAAGTCATAAAGAGCCTCCTCATTCAA
GCTGGTTATTGCCATTCCGTCCCCTTCTACCTGAAATTTTTGTTTCGGTTTTTAAAGTCGTATTTATTTTCGGACTTT
CTTAATGAGAAAACAAAAGGTTTTTATCTCCCAAGTTGAAATCTTACTGGTTTTATAAGGTTCTACTTCTGAGCAAAT
CAGCACTTAGAAATGCACACAAGACGACCTCTGTCAGGTCGCCTCGCTGCTCAGTGACGGCGGGGACAGGAGCAGACA
AGACAGGGAACTAAGTGGAGATGAGAAAATGTCCTGTAACACGAGGTCTATAGGAACAAGAAGGACGGCAGTACAGAA
TAAAGTTCAGTCTCTTGTCAATTGTTTTAGATCTTGCTGAGGAGTTTATTTTCACTGTAAACATGTTGGCACACGGGG
CACCTAATGATTTAAAGGTAATTGAATAGTCTACATTTTAATTGATAGCTTCTTAATTGCACAATAAACCCAATTTTC
AATTAAAACAAAAAAAAAAAAAAAAAAAANAAAAAAAAAAAAAAANAAAAAAAAAAAAACGAAAAAAAAACGAAAAAAACCT
AAACCTAAACTAAC

>Sequ22045SNP2
GCAATAAATAAAATGTGTGGCCACGTATACAGCCCTCACTATAGACGTGTGGCTGTTTCACTCATTTATTAAAATGGA
CCAGTGCATTTACCTTAGTGATCCATTTATTTAGGTTACTTGGAAGTTAAAATACTTTGTTCCAGACTTATATCTTGT
GGGCACAGTAGCATTGAATGGATCGACGTTAACTGTGGGCTCCCTTTTTTAAAGAGAAGCATGGGGTGGTAGTAAAGC
AATGGGAGGAGAATTTTAATAGAAAAATAATACTGGCTATAACCCCAGCGAGGTTTGATGGAATCATGTAGATAGCAC
AATGGTCAGTTGATGCAAAGAAAACTATTTAAGTAAATGAAGCTTTTGAGTAAATAGCTTGATAATAACATGCAGTAA
GAGATGAAGGGAGGAATAAACCGATGGTTGTAACATCTAGTAGTCATTGATATTAATTGTTCATCCTACAGATGTAAT
TTGCAGCGCAGACCTTTTTACAGGGTGAATCTAGTGACCAAGCGTAGATAACAGAGGACACAATCCCATAGTGGGAGC
AGGTAGATAGACCTATGACACCTCTAAGAAAGGGACCATGTATATATGAAATGTACACGAACATCGTACATGGATTTT
GTATTTATAAGTGGTATCCTTTGATCTATGTGCATTACCAGAAACTATTATGCATGCCTTGTTCTTTTGATTTTTAAT
GACCATTATTCCAGAGTACAACAGTAGGAAAGAAGATGCCAAATTTACAATGAGAGCTGATTGTCATGTTTTTTAAAG
GAACAATGGAATTGCGCGTAATACACACTTTTTCTGTCCCTGTCCCTTTGTGTCTCTTTCATTCCCTCATGACTACGT

GATGAGCTGTTCCAACAAGGGTGGGACTTGCTGTTGTTTGCATCATAAAAAATGTAATAAATGTTTGCAATGAATAAA
AAAACCGAAAACCTCCCTA

>Sequ22049SNP2
GTGTGTTGATTGTACAGAAAGATAGTAATGCTTAAGAATGTCTGTGGGATTAAATGTTTACTGAAAAGCACACAGTAG
AGAGAGTTTGAGTTTTAAAAAAAAAAAATGGGAGGGTGAGACTGGATATACGACCCGATTTAAGGCAAAATATTAGGAA
CCCATGGCATCAGAATGTTTGGAAAGTAGTCTGGTTTAATATATTCTAAGGGCTAACCTCCAGCGCGCCGTGATTGAA
ACGCCCCTGAAAATTAAAGTGACAGGCAAAAAAACGGACATGCAGCGCAAGAATCACAGTCTTGTTATGGCACACCTT
CCTTAGCTTACGGCCACTGCATTAATCAAGTGCTCTGTGTTTTACATTGTTGAGGATCGCAGAAGCTACTATACTTAC
ATTGACCACCATAGTGCTTGCTTACTCAAACATTGGCTTAACACAGTCTCTCATTCATGTTTCAGTGCTTTTCCAGGA
GCAAAACATAAATTCATAAATCATTCTCTCTTTGTCCTCATGAATAGTTTAATAGACTCGATGGTCCAAATATTTTCA
GCTGGATACTAAAACAGATGTTTGTGTGAAAGCTATGAGCAAATTGTCCTGCTGGGTATTCAAATAACTTGTTTTGCC
TTTTATATAAATACCCTTGGAATAAAAAATAAATAAAATAGATATCCATGTCTTTGATTGGATGGTTGTATGTATGCC
ATCAGTTAAATGTTACCTTCATGTTTACTTATTTTAAACATAACCACTAATGTAGCCTATCTGTTTGTGAATGCAAGA
GGTGAAGCTTACATTTCAATTCATTTTTTGTGAGGTATTTGGAAATGGCAAATAAAGTGGATATTACTAATAATAAAA
AAAAAAAAAACCGAGACTGCCAA

>Sequ22051EST2
ACATGGGGACTCTGACGGAGGAAAGTGAGCAGGGAGGGTTGGTCAACGTGGCACGAAGGGACCCCCCCAACCCCTGTA
TTTACCAACAACAGGCGCCTGTTTCCCAACACTGTACCGTCCTAGCGCACGACTCTCTCTGAAAAAAAAGCTTCCCTC
TACCACGACTAATAAATGCCAAGAGAAACAAGACAAGCGCCAACAGATAAGGATCGCTGCTCAGAAGTGGAGAGAAAG
ACGAACAGTCGGACAAACACGTAGAGAAAAAAAAACAACCCAACAAAAGCGCACGGATCGTCGCCGCTGCCTTTTCTC
AATGCATACCCTTTGTGCCCGGGGAACGATGAAACCAGAGATCAACGCCGCCGTCGGATTTCTGTCGAGATTTCTGAG
GGTAAAAGGACACGTAAACGATCGACAGGTCCAAACATTCAGCCAAAGCTTACAGGACATTTTGGCAGAGCAATATAA
GCACCACTGGTTCCCAGACAGGCCCTGCAAGGGTTCAGGTTACCGCTGCATCCGCATTAACCACAAGATGGACCCACT
GGTGTGGCAGGCAGGCCAGCGCATTGGCCTGACCATCCAGCAACTCTACCTGCTGCTGCCCAGTGAGCTCACACTCTG
GGTGGACCCTTTCGAGGTGTCCTACCGCATCGGCGAGGACGGCTCCATCTGCGTCCTGTACGAGTCCCAGCCCGGCCC
CGTAGGAATGCCGATGGCCGCCACCGCCAGCTCCCCCTCAGGAAACAGCGGGTCGGTGAGCCCCATGGTGGACAGTCA
CATCAGCTGCAAGGAGGAACTGATGGTGCTGGGCAGAACCAGTCCCTCCAAAGCCTACAATATGATGACTGTGTCCAG
TTAAAGAGGCACACCGTCACCCCGGCCTACTTTTGTTCAGGGTCACGCCATGCTTGGTCAGATCATGTGACCAGATCA
GGGTGAGCCTTTTTAAAGCTAAGAATATGAAATGAAATAGTGAAAGAAGAAGAAAAAGAAAAAAAAAAAAAAAAACTA
AACGAGAACGTACGTAC

>Sequ22180EST2
AGTCCAAGTATGCGTCCTGTCTCAAGCTACTGGATGAGATGAAGGATAACCTCCTGTTGGACATGTACCTGGCCCCGC
ACGTCAAGACCTTGTACAGCCAGATCAGGAACAGAGCCCTCATTCAGTATTTCAGCCCCTACGTGTCAGCAGACATGA
CTAAAATGGCTCAGGCCTTCAACACCACAGTAGCAGCTCTGGAGGACGAGCTCACACAGCTCATTCTGGAGGGGCTCA
TCAATGCACGGATCGACTCCCACAGCAAGATTCTCTATGCGAGGGATGTGGACCAGAGGAGCACAACGTTTGAGAAGT
CGCTCCACATGGGCAAAGAGTTCCAGAGACGGGCCAAAGCCATGATCCTCAGAGCTGCTGTGCTGCGCAACCAGATCC
ATGTCAAGTCTCCACCCAGAGAAGGCAGCCAGGGTGAACTGACACCAGCCAACAGCCAGACCAGGATGAGCACCAACA
TGTGAGGAGGAAGTCCTCTTCATAGTCTGCACCCTGACTCCGCTGTCAGCCACAGACACACATCAACACCAACCCAAC
CCCAACCATCCCACCTACAATGTAAAGACTCTGATGTTCTCTGATGATCTCTGTCGTCCCTAAAGACGATGTGGGGAG
TAAAACCTTTTGTTAGTCTGATGGTTTGAACAGGATGAAAATGTTTTTTTGATGATAGTGCAGCACTTATGCTGTAAG
TTACTGGTAGGAGATGCACTTTGTTCTGCTTACACATGATGGAGGCCTCATTAACAGAAACAACTTTTAAAATAAAAA
AAAACCTAACCGAAAAAAAAAAAAAAACCGACCTACCCTAATA

>Sequ22200EST2
CGCTCCAACATCGACGCCATGTTCGAGGCTTACATTGCCAACCTGCGCAGACAGCTGGATGGGCTTGGCAATGAGAAG
ATGAAGCTGGAGGGAGAGCTGAGGAACATGCAGGGACTGGTGGAGGACTTCAAGAACAAATATGAAGATGAAATCAAC
AAGCGCGCCACCGTGGAGAATGAGTTTGTGCTCCTCAAGAAGGATGTAGATGGCGCCTACATGAACAAAGTTGAGCTG
GAGGCCAGGGTTGATGCCCTTCAGGATGAGATTAACTTCCTCAGGAGCGTCTATGAGGCGGAACTGCGTGAACTCCAG
GGACAAATCAAGGACACCTCAGTCATTGTGGAGATGGACAACAGCCGCAACCTGGACATGGACGCCATTGTGGCTGAA
GTCAAGGCTCAGTATGAGGACATCGCCAACCGCAGCCGTGCTGATGCTGAGTCATGGTATCAGCAGAAGTACCAGGAG
ATGCAGGTCAACGCTGGCCAGGCTGGAGCTGACCTTCACAGCACCAAGAGTGAAATCGCTGAACTCAACCGTATGATC
AGCCGTCTCCAGAATGAGATCGAGTCAGTCAAGGGACAGCGTGCCAACCTTGAGGCCCAGATCGCTGAGGCCGAGGAG
CGCGGTGAGCTGGCTGTGAAGGACGCCAAGCTCCGCATCAGGGACCTGGAAGACGCCCTGCAGAGAGCCAAACAGGAC
ATGGCCCGCCAGGTCCGTGAGTACCAGGAGCTCATGAACGTCAAGCTGGCTCTGGACATTGAGATCGCCACCTACAGG
AAGCTTCTGGAAGGAGAGGAATCCAGAATTTCCTCCGGTGGTGGATCTGCAACCATCCACATACAGAGCTCAAGCAGC
GGCTTTGGAGGTGGCATGGGCGGCGGCATGGGCGGCGGCATGGGCGGCGGC

>Sequ22211SNP2
GCCTGAGTGAGGGTGTGTTGCTTTAGCTACTGGTGAGAAGATTTTATTTCACGTAATATTTCCTCGTGTTCGTACAAT
CTATGAATCAGTTCCTCCATAGTGCAACTACACTTATAGAAACGAAGGACAAGACGACAGTTCTCTTTCTAAAATTCA
GTGTAAGCTGATAGCAGTTAGCCAGATCAAGTGTGTATGCTTACGCTAGCTCGTGGCATGTGTGTCTGTTGGTTCAGT
CTTTGGTTCAGTCGATGAAAGCCAAGTTCGCCAAAGTCCAATGGAAACAGAATGAATCATGACATATATATATGAAGC
ATGTGACTCGATGTCCACTGAAGTTTGTCCTGC

>Sequ22258EST2
AGAAGAAGAAGAAGTCAGTATGGCGCACTCTGATTCGCTCCTCTGTTAGCGAATGAAAGAAACTCTTGTACACGTGTG
GTCTCTCCATGTGAGTGGTGCCTGCCTGCTGACTACTAGCTAAACAAAAGAACATGCTTCGTCGAGAGGTGAGACTGA

GGAGGGAGTACCTGTATAGGAAGGCCCAAGAGGACCGGCTCCGAACAATAGAGGAGAAAAACAGAAGCTGAAGGGAGC
GCTTGATGAAAATTGTCTTCTTCCAACTGAAGTACGCAAAGATGCCCTGCAGCTACAGAAACTACTGGAGTATGACGA
TGAAGGAGCAGAAGGCGTCAGCTCTCACATGGATGATGAGTATAAATGGGCCGGAGTGGAAGATCCTAAAGTCATGGT
CACTACATCCAGAGACCCGAGTTCCAGACTCAAAATGTTTGCCAAGGAGGTGAAGCTGATGTTCCCTGGAGCTCAGCG
CATGAACAGAGGGAACCATGAGATTGCTGCACTGGTGCGAGCCTGCAAAGCCAACAACGTCACGGACCTGGTCATCAT
GCATGAGACAAGAGGACAGCCAGATGGCCTGGTGGTGTGCCACTTACCATTTGGACCCACTGCTTATTTCACACTTTA
CAACGTAGTAATGAGGCACGATGTTCCAGACATAGGCACCATGTCTGAGGCTTACCCCACTCATTTTTCACAACTTCA
CCTCACAGCTTGGCAGGAGGGTATCGAATATCCTCAAGTATCTTTTTCCAGTGCCGAAAGAGGACAGTAGGCGTGTAA
TCACATTTTCCAACCAAGAGGACTTCATCTCTTTCAGACATCACACCTACAAGAAAACAGACCACAAGAACATTGAGC
TGACAGAAGTAGGACCCAGGTTTGAAATGAAACTGTACATGATCAAACTTGGCACCCTTGAGAACGAGAGCACCGCAG
ATGTCGAGTGGCGTCACCACGCGTATACACACACAGCGAAGAAAAGGAGGTTCCTCAGTGTGCAATAAGATAATAAAG
GACCTAAGAAAGCATAACCGCACTGTCAGAGACAGTGCTCTTTTTTTCTTTTTGACTGATAACAGTCTTAAAATGTGG
AGTTTAATAAATAAGAAGTGAGGTTTTGTTAAAGTAACCTACCAAATAAGAATAGAAGTTCAAGTCAATTGTTTGATA
TGGTCAACAATTATGGGTGTTTGGACTGCATCACATACATCATTAGTGACAGCATTTTTAATAATAATAAAGTTTCTG
ACAAACTCATTGTTAAAACTAATGCATACTACACTGACTGTATTATAACTGTTCAACTGTTTAATAATAAAGGTTGTT
TTTAAGGAACGAAAAAAAAAAAAAACCGACCTACCCTAT

>Sequ22363EST2
CTTCGTTTGTCTTCGGCTGTAAGTTTGTAAATTCAAAATATTTCAGCATAAAATTTATTAACGGACCGTTTCACACCT
GCCGTGTGTCTGCTCAGCCTCTGGGTGCCACAGCAGCAGCAGTACAAGAAGAAGAGGGAGTGTGTGAAAATGTCCGAC
GAAGAGGACGCGGCTCCAGCCGTGAAGAAGAGTCGTGTTTTCTATGGAAGCCTCGAGGAGAAAGAGAGGGAACGTCTG
AGCTCAGAGGACACAACACCAATGTGGGTGCAGTTGTCTTCCGCCCACAGGCTGGAATCTATCTCGACCAATCAGATG
TCAGCTTGGCGTCGTGTGCAGCTGATGGGTCTGTGAAGCTGTGGAACCTAAAGAGCGACGAGCCAGTGGCGGACATCG
AGGGTCACAGTGAGCGAGTGTCTCGGGTGTCCTGGCATCCATCTGGAAGGTTCCTGGGAACAACCTGTTACGACAACT
CATGGCGTCTGTGGGACCTGGAGGTTCAAGAGGAAATCCTCCATCAGGAAGGTCACAGCAAAGGAGTCCACGATCTTC
ACTTCCACCCCGACGGGTCGCTGGCTGCTACTGGAGGGCTGGATGCGTTCGGCAGGGTGTGGGACCTTCGGACTGGAC
GCTGTGTTGTTTTCCTGGAGGGACACCTGAAGGAGATCTACAGTCTGCACTTCTCCCCAACGGGTATCACCTGGCGAC
AGGAAGTGGTGATAACACCTGTAAGGTTTGGGAGCTGAGAAACAGGAAGTGTCTGTACACAGTGCCGGCCCACCAGAA
CCTGCTCTCTGCTGTCCGGTTCCAACCTACAGACGGTCACTTCCTGTTGACTGGAGCGTACGACAACACAGCAAAGGT
CTGGAGTCACCCTGGCTGGACGCCGCTGAAAACGCTTGCTGGACATGAGGGGAAGGTGATGGGTGTGGACGTGTCACC
TGACGGGAAACTGATCGCAACCAGCTCCTATGACCGAACCTTCAAACTCTGGCTGTCTGAGTGATGTCATCAGTGATG
TCATGATGATGTCACAGCATCAGCCAAAAATGACACAAATTTTTTTCCCGGATAGTAGAGAAGTCACAAATGTTTGTG
TTGTT

>Sequ22370SNP2
ACGTACGTTCTACGTTAGTTTTTTTTTTTTTTTTTGTAACAAAAAATATTTTTTTATTATTACATGTACCTGCCATGG
TCACTGACATACAACAGTTACAATAACTGACATAGGTACTTTCAGGTTTTAAGATTAAAAATATGCCATTAGAGTTCT
TTTTCTCTCCATAACAATTCATTAAAGGCATTATCAATTCATACATATAGTAATGCTTTGTATCCACAGACCAAGTAT
GTTGTGCATAACATAAATTGTACGATGAGGCCAACAGAAACCGTGCATCTTCATAGTCTCAATCAATAATATAGTGTG
TGCTTTTAGACACTTGTGCTCCCTGTTTGAATCAGATTCAGTTCACATAGGTTTTGATTCTTCCAGCATTGTGCTGTT
TGTTGGTTTGCTCTGACTGTGATGAAGATTTTTAATAAAAATCATGAGGTGAAAATGGTGCTTTACTTTAAAAACTGT
GCCTTTATCAACCTTCATATCAACTTCTTTTCCTTAAAATCTGTTGTAAACATTAGTTGCTATAGGGATGTCCATCCC
TCTCCCTTTTACATGTAAATTCATTTCATTTCCGTGGTGGCGTTTATAACCTTTGATTGATCATTGTCCAATTTGCTG
TTCCCATCAGCAGATGATGGTTGAGGAGCGAGAACAAAGACGTCGGACGTAGTTTTCTATCAGTATTTCCCACTCACG
GTGGAGGGGTGTCTCTCTGTTGCCACCACCGGTTGATGTTTAAAGGGACTGTCGCTCTGTTCTAGACTTGTTTCCTCT
GTGAGCTTGAGCGAACTGGGAAAGTGCTTCCAATAACTCTGGGAAAGCCTCAGATATTCAGTTTAAAAGTTGGGTTTT
TTTATATGTACAATTCCTATTTTAAAATGAGCAACATTCATTTTGGTCTTACAGACACAATAAAACAAAAACGAAAAA
AAA

>Sequ22439SNP2
TATCTGAAGTGACTTCAACACTGTGATCCATGTTCCACTACAGTTGATCAACTGATCTTGTTCCTTTGTGTNTCCCCC
TCAGTTGCCTGTTATTTGACCTTTGACCCTAGCACTGCCAACTCAGAGCTCCACCTGACCGACTGCAACAGGAAGGCG
ACTCGCATGTGGTCGGACTGTCGATCCTTGGACCACCCGGACCGGTTCGAGCGCTGCCCTCAGGTCCTGTGCAGGGAG
GGGCTGCTAGACTCAGTGTACTGGGAGGTGGAGTGGAGCGGCGGTGCCGATGTTGGCGTCACCTCAAATAGCATTTCC
AGAGATGGAGATGCGGAGAGCTGTCTGCTCGGACACAACAAGTGGTCCTGGAGTCTGGAGTGCTCTGAAGGAAGCTAC
ACGCCCTGTCACAACAACAAGAGGTTCAGGTCCTCCTCACCTCAACCCTTCACCCACAGAGTTGGGGTGTACCTGAAC
TGGTCCACCGGATCTCTGTCCTTCTACGGTGTCTCTCAGGACGCCATGGTCCACCTCCACACCTTCACCTCCACCTTC
ACTGAGCCTCTGTACCCAGGGTTCTGGGTGTGGACCTACGGCGGCTCGGTGTCGCTGTGTCAGGTGGAGTTAGACTGG
GAGCGACTGCTGCAGTGAATTCACACAGGCTGAGACATGTGAGACGGACTTCAGCATCGAGCAAGCTGATTGGTCCAT
CCAGTTCCTGTCTGGAGGTGGGTTCGTAAACGTCACTTAGTAACAACCCTTAGGACCCGTTAGTAAAGCAGATAACCA
GTTACCTGCTTAACATGCTCCTTACTAATTCAAGGGAGACACGATGTCTTTCATTGGCCTGACAAACTTCATTATAAA
TCTTTGATCTGTGTTC

>Sequ22568SNP2
CCTGGGACAATCACTGTACATGGAGATTGCAACAGTTACATTCTATCCAGTGTGTTAACAGCTTTACAAACATAGATG
CTCAATACAACAGTGATTATCAAACCTTTTTACTGAGCAACTTTCATTGTACGAGAATTTGTTGTGGGTGACAGACTG
TTTACATGATGGATATTTAAGGTGTGTAAAACACTTCTGGAATCAACGTCCTGTTCATTGATGAGTTTCTGACACAAT
GTCTGTAAATAATCTAAAACTCTGGCTGTACGTCAGCCTTTGTAAACCTGTGAAAAAGTCTCATATCAAGTTGACACA
TTTAATGTAAAACAACTTCCTCTTCACAACTCTGTTCCTCTTCTCAACAACACTGTGATGACGATGACACGTTAAAGC
TTCCACTGACAAACTGACCAAATCAAATGAACTGCTTCCTGTTTAATGAACTGTAAATGTTTCTGCTGAGCTCCATCG

GATGAAGTGAAGACATGGAATAGAACTCACCTGTAATCACACCTACATGACTTTAGGTGTGATTACAGGTGAAACCTT
CAACCAGAGGAGCTCAGTAGAACAGTCTTTATCAGTCTGATGTTGTGTTACTCTGTCAAGATGCTGTTTCTTTAACTA
TAGGTTCTGCTCTTAGGGGAACTGAGATTCAGAGGTTTACTTTTGGACAGGAGACTTGTGTCTGTGAAAAAAACTGTA
CTGCTAAGGAAGAAGAATTGTGCCTACAAACAAACTTAACAGTTGCTTAGTTGTGTAACCTGACAACCGAAGAATTCT
GCATCTGCTTTGCACTATGTCCACTAAAAGTAGAAATGCTGAAATGCAGAAAATGCTGATGTTAACATATTCAATATT
AAACATTTCTGTTTCTGTGGAAATAAAATGAATGTTGATCTGCCTTCCTCTGCCTAATAAAATTCAGTTGTTACTGCA
GAAAAAAACTGTGTGAAGCCATTAAAGTGCTGTTGACGGAAAAACGAACTAAAAAAAAAAAAAAACCGACCTACCCTA
T

>Sequ22647SNP2
GCTTATAAATAAGCGTGACATACTGTATGTGGCCTTTCCATTTACATCGGAGCAAGTTTGTGCGCCGCTGCAGGTTTT
GAATTCGGCTTGAATGTGTCTTGGTTATGCTTGTATTGAGTGCGAATCTGGTTACAGCACTTCAAGAAGCATAAAGTG
GACTATATGGGACCCAATGGTAATACATCTGATGACTTTTGGTCATGTGGTTTTGTGACGATTTCAGGCTTTGAGACT
CCCTGTTTTGTGAGGTGAGTAGGAAAATCCCTCCTCTCATACAGTATTCTTCACCTTTTAGTGTCTCGACACAAGAAC
CGAAGCGCCGTCGATCAATAATCTGTATTCTGTCAGTCATATTACATTGGATAGGAAGCTGTGAATCTGTAAGCCTTT
GACACAGTATGCACATTTTTCCTTCCAACAGATGAAGAGAATATTCTTTGGAAAATGATGTTTAACTTGTTATTAATC
TCCCTCTTAATTGAAGATCCTCTGAAACAAAGACACATACGTCTCTTGGCTGTCTTTAAAAGTTTGTATGAATTACAC
TTTGCTTCATCAACAGTCCAACTGGTAGCAAAACAGAGAGAGAGAAAAAGCAATGATAGGAAACTGTCGCTTTCTGTC
AGGACATCAAGCTAAGGATTAACTCTGATGGGATTCTGCTCTGTCTTGTATCCAGGCGGATTAACCTGCCATTAACCG
CTCATACGTTGAGAATTTTTCAGTATCTCTATCTTATGGGTTGTGTTTAAATGAAAGTAAGTATTAATCACTTCAATC
TTTTTATCACATAGGCCTATAGGACTTAGGATGGGCATAGGGCTGTCACACTCTGTATATCAAGAAATAAAAGGGCAC
AGTCTGACTTGCAAG

>Sequ22843EST2
CGGAATACGAGCAACCTACACAAGATTCAACCATGGATCTTGTGCTGAATGTTGCCGACTACTACGTCCTCACCCCGT
ATGTTTACCCTGCGTCGTGGCCTGAGGAATGGGCCCTGCGGCAGATTATCAGCCTGCTGGTGCTGACCAACCTCGGGG
CTGCAATCCTCTACCTGGGCCTGGGGGCCATCAGCTACTTCTTCATCTTCGACCACACTCTGATGAAACACCCGCACT
TCTTAGAGAACCAGGTCCAGCGGGAGATCAAATATGCGATGACCTCTCTGCCCTGGATCAGCATTCCCACCGTGGCCC
TGTTCTTCGCTGAAGTTAGAGGATACAGCAAACTGTACGACAACGTCGGCGAATCTCCGCTGGGTTGGCCCGGCCTCC
TCCTGAGTATGATCTCCTTCCTGTTTTTCACCGACATGTGTATCTACTGGATTCATCGCTTCCTACATCATAAGCTTA
TTTATAAGCTGTTTCACAAACCACACCACTTGTGGAAGATCCCCTCTCCCCTTTTGCTAGCCATGCCTTCCATCCAGT
AGACGGCTTCATGCAGGGACTCCCGTACCACATCTACCCCTTCTTTTTCCCGCTCCACAAGGTGCTCTACTTGGCCCT
CTACGTTTTCGTCAACATCTGGACCATCTCCATCCACGACGGCGACTACCGCGTCCCCGGCGGTCTGACGAGCGTCAT
CAACGGCTCGGCTCACCACACCGACCACCACCTCTTCTTCGACTACAATTACGGCCAATACTTCACACTGTGGGACCG
CCTGGGAGGCTCCTACAGGCACCCGTCGGCTCTGATGGGGAAGGGTCCCCACGATCTGATCCGCAAACTTCAAGCAGA
GGGAAAGCTGGGAGATGACAGAGTGAAGGCTAAAGGGCAAGTGAACGGACACGCCCAGAGAGGAGTCACATGTAAGGA
GGAGTAACATTAGGGGAAATTACAATTAGCTGCTGAAAGCGATCACTACTTTTGTAAAGACAATGTGCTGCACCAACA
CTCAGCTAATTTGAGAGAGTGTACTTGCCAAATGAGATTTTGAATTCTAGTGGATAAGGCGCCTGACATCATCAGAGG
CGAGCTTTGGGACGCGACCTCCTCTGACGTACCAGTTATGCTTGTATCAGTGACAGACACTGACTAATGGGTCACGCT
AACTTTGCATTCACCACCTCTGTTACGTGCTCGAGCCTCGTATAACTTACGAAATAAACTTGAATTTCACATAACCAT
GAGTCATATGAAAACAAACACAATAATTAGCCTCCTAGCTTCAGCTGTTGTAGCTCACTGCATAAAGATTCACTTGCT
GAGCAACTAACTGACAAATACCCAGGTGGAAAAACGCTGACATTTTCCTTTTAAAGCTGCCTCCCATGCACAGGTAAG
CTGGTGCAGTTTAGCATTTTCAAAACCAGCATCTTTGTAGCTGCTCTGTTCGGGGGCTGAAGAAAGTTGAGTTACTGG
GATAAACTTTGGCTCCAGTAACCCAGTAACCTTTGTGCAGCCACCGCCTGCTGACACTAACTTGATATGGTGAGACAT
ACAAATCTGTTCTGTATCTCAAGAAGTGACTGAAGTTTCTTGTTCCTTTGGTTTACAAGTTCTGATCATGTAGATTAA
CACTGTGCATAAGGACAAGACCATAATAACAGATGATCTTATATTTAGCATTTTTATCAGTATGTTGTCGGTGACAGC
TGGAACATGCTTCTGTTAATTACATCACCTTTAGTTAGTTAATGTCCTTTCACTTTTAACCAGTGCACAGTTACACAC
TGGAGGAACAGGGATGCAAACAGTGTAAGATGAAACTTTTTTGGTTACATATTCATCACAGTTCATGTCTTCGCTCTG
TCTGTAGAGGTGATTGCATCATTACTATGAAAATAAAGCTAACCTTTAACCCCTAAAAAAAAAAAAAACCGACCTACCC
TA

>Sequ22852SNP3
CTGAAATCCGACCCCAGTCTCTGGTGTGTGTCAGCCTGGAATGACAATGGCAGGGATGGCTATGTGGATCCTGGCAAG
GCTAACCTGCTCTACCGGACAGACTTCTTTCCTGGCCTAGGGTGGATGCTACTCAGGGAGATGTGGGAGGAGTTGGAG
CCAAAGTGGCCTGCTTCGTTCTGGGACGACTGGATGCGTCAGCCAGAGCAGCGCCGCAACCGTGCCTGTATCCGCCCA
GAGATCTCACGGACTTTAACGTTTGGCCGGCAAGGTGTGAGTCTGGGTCAATTTTATGACAAGTACCTGCGTTATATT
AAGCTGAATACCGAATTTGTGCCTTTCACCAAGTTAGACCTGAGTTACTTGAAGGAGGAGACGTACAGAGAAACTTTT
CAGAAGGAAGTTTACAGTGCTCCCGTTGTTACATATGAAGATGTTAAGCAGGGGAAGCTAAAAGGAGCCGGGCCCTTC
CGCCTTCAATACTCAAGTAAGGACAGTTTCAAAGTGATGGCCAAAAACCTGGGAATCATGGATGACTTGAAGTCTGGA
GTCCCAAGAACCGGATACAGAGGAGTTGTTAGTTTCATCTCCAGAGGAAGGAGGATCTACTTAGCCCCTCCTCCAGGA
TGGACCCAGTATGATCCGACCTGGAGCTGATAATCCAGGAGAAAGCCTATGTACACTAGCCTTTTTGTAATCTCTAAA
TATCAACTGGGGAGAGCATTCCAGACCAAAGAGTGATGTCCTTAAACTTTCTCTTGGTTCGAATGATATGTACTCTAG
TTCATTGTCTTTGATAATGCAGAATGGATTGTCCCTCTTTCTGGTGTAGGGATCTTTGCCAATACTACTCAGGTCTTT
TTCATTATTCCAAAACTTTTCTTAAAGTGTAGGATGCAGCCTAAACAGATTGTTAGACAAAACTGCTGTAATTCACCA
GAGATCTATTTTTATATTATGCTAATTGTGCACATGACATTCATTAATGCATTCAATGCAATTACCTAATTTGCCACT
TCTGATTAAATATTTGCAATTTCTGGTAGCCTATATGAACTGTTTGTCTGGTATAAATATGAAATCATAATCTGTGGT
ATGG

>Sequ22983EST2
AGGGGGAACGATGCCAGGCTATCTGAGGCCTGAAACAGCTCAGGGAATCTTCCTCAACTTCAAGCGTCTTCTGGAGTT

AACCAGGGAAAACTGCCCTTTGCCGCTGCTCAGATAGGAAACTCCTTCAGGAACGAAATCTCTCCTCGCTCTGGACTC
ATCCGTGTGCGAGAGTTCACCATGGCTGAGATTGAGCACTTTGTGGATCCCAATGAGAAGGTCACCCCAAATTCTCTA
GTGTAGCTGACCTGGACATCATTTTATACTCCTCTAAGGCCCAGACCAGTGGACAGTCTGCACAAATCATGAGGCTGG
GAGATGCTGTGGAGCAGGGAGTGATCAATAACTCCGTCCTGGGATATTTCATCGGGAGGATCTACCTCTACCTTACTA
AAGTTGGTATCGCCAAAGACAAGCTGCGTTTCCGACAGCACATGGACAACGAGATGGCTCACTACGCCTGTGACTGCT
GGGACGCTGAAGCCAAAACCTCCTATGGCTGGATCGAGATTGTGGGGTGTGCTGACAGGTCTTGCTTTGATCTGCTAT
GCCATGCACGAGTCACCAAGGTCCCTCTGGTTGCTGAGAAGCCTCTTAAAGAACCCAAAGTTGTAAATGTCGTCCAGT
TTGAGCCCAACAAAGGAGCCATTGGAAAAGCGTATAAGAAGGATGCCAAGATAGCCATGGAGTATCTGTCTGTGTGTG
ACGAGTGCTTCATTACAGAGCAGGAGCAGCTACTTAACGAGTCCGGAGAGTTCACCATCGAGACGGAGGGCAAGACGT
TCAAACTCACAAAGGACATGGTCAGTGTGAAGCGTTTCCAGAAGACTCCGCACGTGGAAGAAGTCGTTCCAAATGTAA
TCGAGCCCTCCTTTGGCATCGGTAGGATCATGTACACCATCTTCGAGCACACATTCCACATCAGAGAAGGTGACGAAC
AAAGAACGTACTTTAGCTTCCCTGCTACTGTAGCTCCATACAAATGTTCCGTCCTGCCTCTGAGTCAAAACCAGGAAT
TCGTTCCCTTCGTGAAGGAATTATCCGAGGCCATGACTAAGAATGGTGTGTCTCACAAGGTGGACGACTCCTCAGGAT
CCATCGGGAGGCGCTACGCCAGGACCGATGAAATCGGAGTGGCGTTTGGCATCACCATCGACTTCGACACAGTGAACA
AGACGCCACACACAGCCACTCTGAGAGACCGAGACTCAATGAGGCAGATCAGGGCCGAGGTCAGTGAGTTGCCTGTGA
TTGTTCGGGATTTGGCCAACGGCACATTGACCTGGGCTGAAGTGGAGAGCAAGTACCCCATCTTTGAAGGACAAGAGA
CCAGCAAGAAGGACACGGTTGAAGAGTAAAAACTCACTCGTCTGCCAGCCACTCCGTGAAAATCCACAGACTTGTTGC
GTATGCATCACCAGGAAAGAAAATATTGCAACTGTATTGTAATAAGCTATAGTGGTGCACCGAGCCCTCCTTCTGTTC
TCCACCACTGCAATCACTAAAAATGTCATGTATTCATCATATCTAACAGTTGTGGAAAAGCTTGCACAGGACTGTTGT
TTCTTTCGATGAGCAACATTTCATCCATGTATGACCATGTCGATGTTTTAAAAGGGAGAAAATGTAATCAGGTTGTTT
TGAGTCGTCAGTGGAAGGAGGCGTCCACCATTCCAGTTTTCAGGCAGATTGGATTGAGATATTTAACAATTTCAATAA
AAACAGCCAGCACTTACTGGACACAGAAACAAAAAAAAAAAAA

>Sequ22984SNP2
CAGAGTACGATGGGGAAGGAGGGGAGAGGCAAGTTAGCATGACAGAGCTGGAGGAGATGCTTGGAGTGCGAGACATGT
TCTCTGAAGGCTGTGGAAGAGCAGATGTGGAAACTGCTGGAGTTGGAGTAGGTCTTCACAGTGAGGGGAACCAAGGAA
ATATAGAAAAGATGGATTCAGAAGCTTCTAGTGAAGAAACGGATGATAAAGATGTGGATTCAGATGCTCCTAACGAAG
AGACGGATGATAAAGATGTGGATTCAGATGCTCCTAGCGAAGAGGCGGATGATAAAGATGTGGATTCAGACACTGCTG
GTCAAGACAGTGAAGGGAAAACTACTCTTGATGCCCAGCCAGAGGAAGCTGATGAAGTAACACAGGAAACTTCAGAAG
ATGCGGTGAAATCAGCGAGCAGCAGTGAAGAGCAGGCTGATGTGAGGCGTTCAGGAGCTGCCAGGTCAGAATCTCCTG
AGTCTTCAGCTGAATATGAGTCTGTGGATGAAGAACAAAATTCCACCAGCACTCTGGTCTACATCCTCTCCACCACCA
TGTCCATTATTAAAGCTCCACTACGCCCTGTGGTCTCCAAGGTCACACAGTTACCTGGACAGGTGACCTATGTTCTTC
AGGAAGACCTTGGAGTTCTCTCTGCCTTGCCTGGAGATACCTACTACCTTCTCCACCTCTTGACCTCTGACCTCTTGT
CCTGGATGGGCTCAGCTGCAGAACTGGTGCTGGGTGTCGGGGAAGCCTGCTTCTTCAGTGTTTACTACTGCACGTCAT
CCATCCTGGGAGCCCTGATGAGCAGCTGCCACACTGGGGTCTCTGGCATGGGGACCCTGGCCGGTGACACAGTGGGGG
TATTTGGTGGTGTGCTAAATAGTACTTGGTGGGTGACCAAGTTCTTTGGAGAGCGGCTGTGGGAGCAAAGCGAGGGTT
ATGCAGAAACGATGATGTCAGAGATGGGGGGTCAGGCCAAAGCTGTAGGTGGAGGGTTGGGTAAGCTGGCATGGAGAA
GTGGAAATTGCGTGGGTAATTTGTTTAGGCTGGGATGGGGGTTAATTACTGGGATGGTGCATATGCTCACTGGTGCGA
TGAGAGAGGGATATGGGCAGGAGTCAGAGTGAAGAAGGAAAGGACCATACCCCAGATGAGGCCATCAGTAACAAACTT
TTTCAAATGTTTTTCTCCAGTTTTGGTTTGTAAATTTTGAGGTGAGAAAAAAGGAAAAACTATTTGAAGCTGATCTCA
TTTGATATGCCTTAGTTGTTATAGAAGGATAGCCACAATCAAATTTCGTATTATGCAGGTCTTACACTTCACTGTGGA
AAGCTGGCTGTCCAAATAATCTGCTGTACTTGAGAGCTTCCCCAACAGTGATTTAAAAAACACTGTCCACACTGTGCT
TTTGGATGTAATTGTTCAAGACTACTCTTGTATGATACTTTGGTTTGTGTGCAAGTTTGAATAAAAGAGACTGCTGGC
TTGCCATTGTAAAAAGAACAAAAAAAAAAAAAACCGAAACCTACCTAACT

>Sequ22996EST2
ACAGTTGGACACATCCTCAGCGCAGCCAGGCGTTAGAGGCGCTGGTGCCCCGGAGAGGAGCAGGAAGACGGAGGACGA
CTTCTTTCTGGTTTCTCAGCAACAGGGGACGGGCGGAGAGAGGAGAAAGATGCCCAAACCGATCAACGTGCGCGTCAC
CACCATGGACGCTGAGCTGGAGTTTGCCATCCAGCCCAACACGACAGGCAAACAGCTCTTTGACCAGGTGGTGAAGAC
GGTGGGTTTAAGGGAGGTGTGGTTCTTCGGCCTTCAGTACACAGACAGCAAAGGCTACGTGACGTGGCTCAAAACTCA
AACAAGAAGGTGACCCAGCAGGATGTGAAGAAGGAGAATCCGCTGCAGTTCAAGTTCAGAGCAAAGTTCTTACCAGAG
GATGTTTCTGAGGAGCTGATCCAGGAGATCACACAGAAGCTCTTCTTCCTGCAGGTGAAGGAGGCCATTCTGAACGAT
GAGAACTACTGTCCCCCAGAGACCGCCGTGCTGCTGGCCTCGTACGCTGTCCAGGCCAAGTACGGAGATTACAGCAAA
GACATTCACAAGCCCGGCTACCTGGCCTCCGACCGGCTGCTGCCACAAAGAGTTCTGGAGCAACACAAGCTGACGAAG
GAGCAGTGGGAGGACAGAATACAGACCTGGCATGAGGAGCACAGAAGCATGCTCAGGGAGGATGCAATGATGGAGTAT
CTAAAGATCGCTCAGGACCTGGAGATGTACGGCGTCAACTACTTTGAAATCAAAAACAAGAAGGGCACAGAGCTGTGG
CTTGGGGTCGACGCCCTGGGGCTCAACATCTATGAGCATGAAGACAAGTTGTCTCCAAAGATCGGCTTCCCCTGGAGC
GAGATCAGGAACATCTCCTTCAACGACAAGAAGTTTGTCATCAAGCCCATCGACAAGAAAGCTCCAGATTTTGTGTTC
TACGCCCCTCGGTTGCGGATCAACAAGCGCATCCTGGCGTTGTGTAT

>Sequ23093EST2
CGAACAAGTTGCAGGACATGCTTGATGGGTTCATCAGAAAATTTGTGCTGTGTACCGAGTGTGACAACCCTGAAACTG
ATCTGCATGTCAATCCCAAGAAACAAACCATTGGCACTTCCTGTAAGGCCTGTGGAAACCGCGGCATGCTAGACACCA
GACACAAACTCTGCACGTTCATCCTCAAAAACCCACCAGAGAGCAATGAGAGTGGATCTGCATCTGTAAAGAAAGAGA
AGGAGAAGAAGAACCGCAAGAAGGACAAGGAGAATGGACTCTGGCAGTGGCGAGGCTGGGAACCACGACAACTTTGAC
GCCCCTCAGGCTGTGGACGGAGACGACGACGACGAGGACTGGGCAGAGGAGACCACAGAGGAGGCGCAGAGGCGGCGA
ATGGAGGAGATCAGCGATCACGCCAAGAACCTGACGCTCAGCGAAGACCTGGAGAAACCCCTGGAGGAGAGGGTCAAC
CTGTTCTACAACTTTGTGAAACAAAAGAAGGAGAGTGGAACCATCGACGGGGCTGAAAAGGAGATCTTGGCGGAGGCG
GAGCGTCTGGATGTGAAGGCCATGGGCCCCCTCATCCTCAGCGAGCTGCTCTTCAACGAGAACATTCGCGACCAGATC
AAGAAGTACAAGCGCCACTTCCTGCGGTTCTGCCACAACAACAAGAAAGCCCAGAAGTATCTGTTGGGAGGCTTCGAG

TGTGTCGTGAAGCTGCATCAGGTCCAGCTCCTGCCGCGGGTTCCCATCATCCTCAAAGACCTGTACGACGCTGACCTG
CTGGAGGAGGACGTCATATTCGCCTGGGCAGAGAAGGTTTCTAAGAAGTACGTCTCTAAGGAACTTGCAAAAGAAATC
CACGCCAAGGCTGCTCCTTTTGTCAAATGGCTGAAGGAGGCAGAGGAGGAGAGCGAGGGCAGCGAGGAAGAGGAGGAG
GAAGATGATGAGAATGTAGAGGTGGTGTACTCGTCCTCTGCCCGCGAGCTCAAAGTGGAGACTGTGAAACCAGACACG
CCTGAAAAAGAAGAGGACGACATTGACATTGATGCGATCTGAGAGACTCGGATGATGATGATGATGATGATGATGCTT
TTCTTGTGTTGTGGCTTTGACTCACGATGCCCTGTAAGACCTAAATGGCTGGCCTTTCCCTCCTTTACTACCTGCCCC
CACCACCCTCATCCTTCTTCCCGCCCCAATCCTGCAGCCCTCCTCTGCTTTGTGGCATGACTTAACATAGCGCTTTAC
TTGCTGCACATTAACTCTGTAATTTTGAGATCTATTCAGTTGATGAAATGAAGTCGCTTTGGGAGTTTTAATCATGGG
TGTTTTGGGGGATCATTTTGACTGCTACTTTTCTTTTTTTCTTTTTTTTTTGGTCCCTTGTTATTTTTCCAGTCAATA
AGATGAATGTTTGCTATCC

>Sequ23165EST2
AAAACAAAAAACAAAAAAAAGATTCCCCACACATGGTCTCCAAAATGTTGGTTGCCTCTTCAACGGTGTAGTCTGGAT
AGTGTCACAGTATGACATTGTGTCTGTCAAACCCTTCCCGGGAGGTGAAATTTGCCTGTGAAGCGTCCATGGTTGTGC
AGATCAAATGGACTGATTGCCTTCCTGATACCCAGCATGTTGCCCTGCGCTATCATCTTAAAGGTCCAGGGATTCTGG
TTGTTCTGCTCCATATCCACCTGTTTCTTTTGCAGGGCTGCTAGACTCTCTCTGCTCATTACCTTGGCAGGGAAGGGT
AGCTTCTTTGCCACTTCAGTCAAGATATGCTCAACCTCCCCCAGCTCCACCTTTCCTCCCACCTCCACGATGAAACGA
CCATAGCGGACAGGTGTCACATAGTGGTCGATGGCTCCCTTCCCCCACCCATGCGCTGACCCAGGCCTTTACGTGTAA
TTGGCTTATACGGGGCATTGATGCGCCAGCGGGCAAATGTAGTCCGGGAATCCATCTTGCGGTTGACGGTCAGACGCA
TCATTTCTATGTGACCCCAATGGAGATAGCCTCCTCCCATGGCCACAATAGCATACTGTCCTGTAGTGAAGGCATTTG
CTGCCTTAGCTTGGCCTTGGATATCGCGCAGCTTCTTCATTTCTTTCCTAGCCCTTTTAAGGTTAGGAACCTTGTTCA
TAAACTTCAGTTTGGGTTTCTCTGGCAGCACCACATCACTGTAGTCTGGAGGGATTTCATACGTTTTAAACCCAGCAG
CTAGGACTTTCAAATGGTTGTACAAAGGACCTTGTCGGTGACCGGGGGCTTTACAGATACCGGTCAATCCGCCGACAG
CAGCCTTGACGAATGAAAACATCTTGCTAACCTTGAAATGGGGCACTATAAACACAAAACCGACACTTCGTGTGTTTG
ATATAGGAGCATTTTAGGTGGTTAACTAATGAAATTGTTGTGGACACCACGCTCTGTACAAGGTCG

>Sequ23258SNP2
TGGATGACAAGGTGAAGGAAGTGGAAGGCTCAGAGAGGAAGAAGGCCAAGATCCTGTCTTCTGTCTCGAAGAGGGACT
TCATCGACGTGCTGAGGAGGACGGAGGAGGAGGCGGAGGCTCCGCCAAGGCGCAGAGGGAGGAGGCTGCAGCAGATGA
GAAGCCTGCCTGGAGCGTCCTCAGAGACGACTACATGATGGGGGCCACCATGAAGGACTGGGACAAAGACAGTGACGG
AGAAGATGCTGATCCTCACACAGGAGGGGGGCGGAGGAAAGCGATTCAGACTGACACCATCCATACTCACTGAAGGGA
CTGTGTGGTGCCTTCAGGGACAGTCTGGGCAACACAAACCCTGACCTGCAGAGACCAGCAGCTCAGAGAGGACGGAGA
AAGTGTGGAAATGCTCCTGACTGATAAACACATAGGACAAGCTTCTCTCGGCCGGTCGCAGTACGATGTGTTCTGTTA
CCGTCCTCCACTCCGACCACAGGTCACATGTGGAGACGGGATGTAACAGATGAGACTGGAGGAAGTGTAAGAGGCTCC
TCCAGGTTTGATCTCCTGCTCCATCTTGGAGTCAGAGTCCATCTGCTCCTGTTCAGTCTGCAAATTGTTTTCTGAGTG
AACGACCTGTAAACTCTGTAAATAGACATGTTTTCTACTATAATGGTTGAATAAACAGCCAGTTCATGTATCACATAA
ACGAAAAAAAAAAAAACCGACCTACCCTAATAGT

>Sequ23460SNP2
ATAGGGAGGTCGGTTTTTTTTTTTTTTAGGTTCTAGCAGGTCTTTTATTTTCATAGGAACAAGAAAAGATGCCGGTCCA
GACCTGCTTATCACTCACCTGTGTGTGACCTACAGTGGAACATTAGAACTCTAGGAACTGGGACAGTCCATTCTTTTG
GGAACAGTAGTTTACACTTAGGTACACTCCCGCGACTTTCAACCACACCTCAAGGTCTTCATCAGCACTTTGCTCAGT
TTTCCTGGGGTTAGTTTAGACATCATCATGCGACCTACAGTAAATATGGAACCTGGGTCACGTGCCAACACGCAGTTG
TATGGTTGGCCTCTGTTCAGAGATGGTTGTCCACTTATCCCGCCCCATGACCTGTAACCCTTAATCCTGATTGGTTAG
AGGACTAGGACAGGGTCAAAGTCATGGTGTCTGGGGACGTCTGGAAAGGAAATCTTACAGTGGCTGACAAAGGCCAAC
GCGCTGCAACGGTCCAAAACACCTCCACTAGGAGAAACGAGCTGCAACCCCAGAAAGATGCAGATTCAAAAACACACA
CGAAAACACAAAACCAACACAACAACAGAAACATGCTACAAATGAGACAATGTGCTGCAGGGAGACAAAACCAACACA
TGAACAGCAAACACGCTGCAAACACAGAAAGAACACAAAACTGAGAAACAGAAAAACCACTGCATATTGACAACAGAA
GAGCTACCAAACCTCTAGGGAACACCCTGAACACCTTGAGCAACAAGCTTAGAAGCTTCGACTGAGAGGCCAGACCTA
CGACTGGACCTTTTACCCTTTTACCAAGCAAGAGAATATGAGGGAAGAAAGATGAGATGACTTCAAAAGGGTTGTGTT
CTTTTTTACATTTGGCCATAGTTTTTACAATATTATAAAAGAGCAACAGAAAACATAGCAAGTGTTAGCGTTAGCTTA
TACACAGTCCATGGGTTTTCCACGTAGCGCTCCCCCCAGAGGCCTGGAGCACCTCTGTTGTGTTGTCTTTTTCCTGT

>Sequ23511EST2
ACGGAACGTGTTCCGGTTGGTGATGTCGGTTAAGTGTGCATGCCGCGGAGTGTAGCCTCTGTTAAAGTTGTAGCTGGA
GCAACACCTGAAACGCCGCCATGGGGATCCTGGAGAAAATCGCGGAAATAGAGAAAGAAATTTCTCGGACACAGAAAA
ACAAAGCCACTGAGTACCATCTGGGTTTGCTAAAGGCCAAGCTAGCCAAATACAGAGCTCAGCTTCTGGAGCCCTCCA
AGTCAGCGGCGGCCAAAGGCGAGGGCTTCGATGTCATGAAATCAGGAGATGCTCGTGTTGCACTCATCGGTTTCCCCT
CTGTGGGTAAGTCCACTTTCCTCAGTCTGATGACATCAACAGCCAGTGAAGCTGCTTCCTATGAATTCACGACCCTCA
CCTGCATACCTGGCGTCATAGAGTACAAAGGGGCCAACATCCAGCTGCTAGATCTGCCAGGAATCATTGAGGGTGCTG
CTCAAGGTAAGGGCAGAGGTCGGCAGGTCATTGCTGTTGCCAGGACAGCAGATGTTGTCATCATGATGTTGAATGCTA
CCAAAGGAGAAGTTCAGAGAACACTTCTAGAAAAAGAGTTGGAGTCGGTGGGGATCAGACTCAACAGGACGAAACCCA
ATATTTATTTCAAGCCCCAAGAAAGGTAGGCGGCCTACTCCTACAACTCCACAGTTCCTCTCACCCACTGCTCAGAGA
AGCTCGTTCAGCTCATCCTTCACGAATACAAAATCTTTAATGCAGAAGTGCTGTTCAGGGAGGACAGCACTCCAGACG
AGTTCATCGACGTCATCGTTGGGAACAGAGTTTACATGCCTTGCCTATATGTGTACAATAAGGTGGATCAAATCTCCA
TCGAAGAAGTGGACCGCCTGGCTCGCAGACCTCACAGTGTGGTCATCAGCTGTGGAATGAAGCTGAACCTGGACTACC
TTTTGGAGACGCTGTGGGAATACCTGTCGCTGATTTGCATTTATACCAAGAAAAGAGGAGAGCGCCCAGACTTTAATG
ATGCCATCATCATGAGAAGAGGAGCAAGTGTAGAACATGTGTGCCATCGAATCCACAGAACCTTAGCCAGCCAGTTCA
AATATGCCCTCGTTTGGGGAACCAGCACCAAGTACAGCCCACAGAGGGTCGGACTGACGCACATCATGGAGCACGAGG
ACGTCATCCAGATCGTTAAGAAGTAAAACAAGCTTCTGATTGGACGAAGGACAGGCTGAGCAGGAAGAGCTTAACTGG

GGTCGGGGGGTAAAGTACTGTACAGTAGATATACAGCAGTGGAAAATAGCCGCATTCTTATTTTGTATATGATCATAA
ACAAAAGCAAAAACTGAAGAGAGACCAGATCTTATGGTTCTGGACGGAAACTGGTTTATTTTACCTTTTAAGATAAAG
AAGATGCATTGATTCTAAAAAAGAGAAAAACGGGGAAATAAAAGTCCAATAAAAAACAAGTAAAAAAAAAAAAAAACA
AAAACCGCCTACCTA

>Sequ23896EST2
GGTTTTAAAATAGCAGGCGAGAGCTGCAACTCCTCACTCTCCTCTCTGAGCAGAAACAGCATCCATCATGACCAGCTA
CTCTTCCTCCAGGTCGGTGAGGACCAGCACCAGCAGCTCCGGCGGCTCCATGGTCGGCATGGGCGGCAGCAGGCGGGT
GTCCTCCATGAAGCTCGGCAGCGTGTACGGCGGAGCGGGAGGCTCTGGCGTCCGCATCTCCTCCGCCTCCATGGGTGG
ACGCCTCGGCGCCGGCCTGAGCGGAGGGTACTCGTCCTCCATGAGCTACAGCGGGTCCGGCATGGAGGACAGCCTCAT
CGGCAACGAGAAGTTCACCATGCAGAACCTGAACGCCGCCTGGCCACCTACCTGGCCAAGGTGCGCTCCCTGGAGAAG
GCCAACGCCGAGCTGGAGCTGAAGATCAGGCAGTTTGTGGAGAGCAAGGTCGGCCCCACCACCAGAGACTACAGCGCC
TACTTCGCCACCATCTCTGACATCACCGGCAAGATCCAGAACGCCATCAGGCTGAACGGAGCCGTCCACCTCAGCATC
GACAACGCCCGGCTGGCAGCAGACGACTTCAGGACCAAGTTCGAGAACGAGCTGGCCATGCGTCAGTCGGTGGAGGCG
GACATCGCCGGGCTGAGGAGGGTTCTGGACGAGCTGACTCTGGCCAGGACCGACCTGGAGATGCAGATCGAGGGTCTG
AAGGAGGAGCTGATCTTCCTCAAGAAGAACCACGAGGAGGAGCTGCTCGCCGTGCGCTCTCAGATGAGCGGTCAGGTC
CACGTGGAGGTGGACGCCGCTCCAGGAGATGACCTCACCAAGGTCATGGCTGAGATCAGAGAACACTACGAGGCCATC
ACCGCCAAGAACCAGAGAGAGCTGGAGAACTGGTTCCAGACCAAGTCGGAGGCTCTGAACAAAGAGGTCGTCACTCAG
ACCGCCACCCTACAGACGTCCAGGTCAGAGGTCACAGAGGTCAAACGTACGCTGCAGTCGCTGGAGATCGAGCTGCAG
TCCATGTTGGGCATGAAAGCGTCACTGGAGGGAACTCTGGCCGAGACACAGAACCGCTACGCCATGCAGCTCTCAGGA
TACCAGATGCAGGTGTCGTCTATGGAGGAGCAGCTGGTGCAGCTGAGGGCCGACCTGGAGCGGCAGGGACAGGAGTAC
AAGATGCTGCTGGACATTAAGACGCGACTGGAGCTGGAGATCGCCGAGTACAGGAGGCTGCTGGACGGAGAGGGCAGC
AGCAGCATCCGCTCCTCCTCCTCCTCGAGCACGAAGACCACACGGGTGGTGACGGTGGTGGAAGAGGTGGTTG

>Sequ24343SNP2
CAGAGAAGAAGAAGCTCCGGAAGCAGGCGGTGGTAGCAGCTAACGTTAGCATCAGATGTTCGGCGGGAATAAATTGGT
CAAATATTCGTTATTCTGAGGTTCTGATGTTTAGAACTACACACCTTAAAATTGCCGCGGAACCATACCCGCCACCTG
TTACCTGCTAACGGGACAGGGAATACACTGACGGGTCCTCAGTCCTTGGTCGTCAGTCCTTGGTGCCTCTGATGATAT
GAGCAGCTCTGCTCATAGGAGGAGGAGGACCGCAGGAAGTCCTCTGGTTTCCAGACCTAGCAGCAAGAACAGCAGAGC
AGCTAACGCTAACAGAGTGGCTAGCAGGACGGCTGGCAGGACGGCTGGCAGGACAGTTAGAAGTACAGCTGACAGGAT
GGCTAACGTTAGCAGGATGGCTGATGGTGACCTTGCCCCCCCCACAGAGACCATTGATGTGATGGACAACACTGAGGA
CATTGTGGAGGAGGTGGTTGATTTGACCTGTGAAGGATCAGAAGCTGCTGTGGTCGACCTCACAAACAACGACTCAGT
GCTGCTGCTGGATGAAGGTCCTCAGAACAGAAGAGTCCCCACAGGTGAGAGCTACATCGTCAGCAGTGATGAAGATGA
GGACACGTCTCCTGTCCTAAACGCTGCCATAGTGTCCTCTGTTCACACTTCCAGGTCAACTCCGGGGACGATCAGCTG
TCCCGTTTGTTTGGACTCGTACTCTGAGATCGTCGACAGCGGCCGATTGGTTGTATCCACTAAATGTGGTCACGTATT
TTGCAGTCAGTGTCTGAGAGACGCTCTGACATCATCACACACCTGTCCTAACTGCAGGAAGAGACTCACCCATCGCCA
GTACCACCCCCTCTACATTTGACATCACTCTGACATCACCACACACATTTGCTGCTGTTGGACAACATATTTCCAGTG
ACATCATCAGTGACATTATCGGATGTTTTTTGCTGCTGAATTTTATTTTTTAATTTTTTTTGTGTTTTATCAGAAACA
TGAAACCTTCAGTCAGAGCTGATTGATTATTGATATTTGATTGATGTCTCATTCCTCAGTTTTCCAGCTCATTCTTTG
TTTTCATCTGTTAAATGTTTATTTTCATGTGTAAAAGTAGAAATTAATAAATACATTTATTGACTGTG

>Sequ25437EST2, Sequ25437SNP2
AAGAAGAAGAAGAAGGAAAACTGAACATAATCTGATCTACTTGTAACAAAGAAAATGGACACACGGTTCACTCGAGGG
AAATCCAACATCTTGGAGCGGTCGCTGACCCGACCCAAGACTGAGGTCAGCGTGAGCGCCTTTGCCCTGCTGTTCTCA
GAGATGGTCCAGTACTGTCAGAGCCGTGTGTACTCTGTGTCAGAGCTGCAGACACGCCTGGCCGACATGGGCCAGAGT
GTAGGAGCCAGCATGCTGGATGTGCTGGTGCTGAGAGAGAAGAACGGGAAGAGGGAGACCAAAGTCCTGAACATGCTG
CTCTTCATCAAAGTTAACGTGTGGAAGTCTTTATTTGGGAAGGAGGCTGACAAGCTGGAGCAGGCCAACGATGACGAC
AAGACTTATTACATCATAGAAAAGGAGCCGCTTATCAACGCGTACATCTCTGTTCCCAAAGAGAACAGCAGCTTGAAC
TGTGCTGCCTTCACTGCTGGCATCGTGGAAGCCATCCTCACACACAGTGGCTTCCCTGCCAAGGTCACTGCCCACTGG
CACAAAGGCACTACACTCATGATAAAGTTTAATGAGTCGGTCATTGCCAGGGACAAGGTCTTTGGATGGCAGATAAAT
AAGACTGGGGAAAGTGGCTGGTTATGTTAATCAATACAGGATGATGGTAAATCATCCAGGTCCAGGCTTGTGTTTGTT
CCTGAAGTGTGGGTGACTGACTGAATTTCCTTGTGGTAACTGATGAGATAGCAACTTCATGAATGGATAACTACAACG
TACTGAGCCACATTTAGAGCTGAAAAGGTGCAGCTAACTGGTTCAATCCCCAGGGTTTTGTGGATCATCTCACTCGTG
TTGTACCTCATGAAGGGACACAAACTTGTTTAGCTGTAGATCACTTAAAATTTGTAGCTTCATGTCACATTTTTACAT
CACAGATCATTCAGAAAAGATCAATATGTT

>Sequ25975EST2
GCTATCTCGACAAGACTTCGTTCGTCATTTTGAAAGAACAAGACGGAGCAGATACAGAGTCTCTAAATGGCTGCGAGG
AAGTCTGGATCAGATGCATACAACAATGGACCCATCAGCTACCTTGATGATGTTCCCTTCAAAATTAATGAAAAATTC
CGCTGCCCAGCCAAAGTGGGGGTGCCTGTTGGCTTCTGCCTGCCTGACTGTGGCTCTTTGCTTCTGGACACACAGTAT
GACTTTTCTTTGGAGAGGCGAAGTGTGCGTTGGGGGTTGAACTGGCTGAGGCCAGAGCAGCAGAGGCCAGAGCAGAAG
AGGCAGCAGCCAAGCAGGAGGCAGAGAATAGAGAGTGTTTGGCTCAGGCCCAGGACATTGATGGTGGTGGAGGGAAGA
TACCCCGGTCTGCTGCAGACGACCAGGACCTTCTACCACCAGCATTGAACCCCGTCCTGGCAGGGCTGAGCCATAATG
CCATCCTCACACCACTGCCTGCCCCAAGCCTTGGCCCCAGGAAAACCCAGCCGAGCACTCCTCAGCCACACAGCCTCA
ACCTAGCTGACTTTGAGCGGGAAGAGGACCCATTTGACAAGTTGGAGCTCAAAACTTTGGACGATAAGGAGGAGCTCA
GGAACATCCTCCAGAGCCAGCCCCAAACCTCAAACATCCTCCTTCTGTATCCCCAACAGAGGTCTCCCAGATGGGGTC
AGTGTCACGTGGAAACAGCCCATCTCCTCCCAGCATCAACACCAGCCTCCCGGCCAAACCAGGCTTTACCCACAAACC
CAATGGGTTGGTTGCCTTGCTGGACATGGACAGAGTTGGGCATCCCTGGGAGAGTGGGGTTTGACACGGATGACCGAC
CATGTAACATCCGCTCTCTGACTTTTCCCAAGCTCTCTGACTCTGGTGACCCAGAGCCAGTGAAGTACTGCCCACTCC
CTGCACCCATCTCTGCTCCACGACAGAACCTACCCAATGGCAGTCCGCCGGCAATACCCAAGACCCAAGTTATTGTTG

CCCCTGAGCCACAACCACCCAGCCACACTAAGAGTGGCACACTGAAACTGGCCAACCCAGGGTCAGGATCTGCTGGTT
TGCCGTGTGGCGGAGCCCTGCTCAGCATGACCCCCAGCGAACGTCAGTGTGTGGAAACCCTTGTAGGCATGGGCTACT
CTTACGAGGGTGTGCTACGGGCCATGCAAAGACAAGGGCAGAACGTGGAGCAGGTGCGACTCCCTCTCACACCAATGC
ACAGAAATCTGGATTCCTAGCGTACTTACTGTCCGACATGTGAAACAGTTCATTATTTGTGTGGTTGTGCAATTGTGT
TCAGTGACGCATCAATGCATCTGT

>Sequ26609SNP2
AAATATACTTTAGTTTTATTTGTAGGACGTTTCCTCCACGCAGCAAACAGAACACAGCAGGACGGTAACAGCATGAAC
ACCGATCAATAGATCAGCCAATAAATCAACCAATCAGTCTGAATGATCGGGCGTGTTCGCTCGGAGGATTTAAGGATC
GTTGGGTGAAATCATTCAGTTCTGCAGCAGCTGGTTTGTAGCAGCACAGACTCTTTTATTACTAACATCACCGTTCTG
TCAGTTTGCTCCTTTACATTCAGATTCAGATTCAGTTTTTGGACAGAGTTGTTGAGTGACAGGGTGCGCTTCATTTAT
CCATGTCAGCGTGCCGCTTGTTTTACGAAGCCGCAAGAGAAGAAACATGAAGATAATCAAAGCGCTGCATTCACTTCA
CTGTGTTTGCTTCAGTGATCAACCTGTGGTGAACCGATCTGATTGGACAGGGTTCAGAGACACACCTGTGACTCTCTG
TGGTAAATGATTCATTCAATATAAAACACAGTGGGATAAAAAAACTTCTGAACTGGTATGTTTTGTTCAGGTGCCACA
TTGAGACATTAGTTCAGTCTGACATAAGCAGCACTTCCTGTGATAACGTGAAAACCAGGTTCAGGTTCACCTGTCTGA
GTCCAGCACTGAGACCGACAGGTGTGCAGAAAAACAGTGACAAAAAAACGTTCTAGTAGAAAAAAGCTAGTTTGTGTT
TTTATATGAGTAAAAAGTTTTGTTTTGTGTTGAAGCAGAATTATCATATAATCACATAATCACATCGTCTGTAAAATG
GATAAATTCCTTCAACGTGAAGCAGAGAAAATCTTCCAGCATCTTTAAATCCACCTGGTTTCAGTCGTGTTGTTCGTC
CGGCTCGTCTTTGGCGTAACGTTATAAACAGCTGGCGTTCAGTCTCAGCTGTCTGACAGAGATCATCTCAAGGCGGGT
TAGAAAATAAAAACCTGATCCAGCAGAGACAGACACGGTCACTGGGACAGAACCCGGCGCACAGTTCACTTTGAGCCA
GCAGAGTCCCGTAAAGACCCGATCCTCAGAGAGTCTGTT

>Sequ28107SNP2
GAGAACACGGTGCTTTTATTTGTATTATGTCTTCCAAGTTCTAATTAAGGATTAACAACAGTGTTTGTTGTTGTAGTC
AATATCTTTAAAATCACAGCACAGCATATATTATATATATTGATTAAAAGAGGGGTAAGTGAATTGTAGAGTTTCTCA
ACACCACAATTTAATTGAATGTTTTATTATCCCTTATTT

>Sequ28112EST2
TTTAACTTTTTTAATTGGCATTTGCATTTACACTGCACGGAAAAGTTGCCACAAGCACCTTTTGAAAAACACGTCTAC
ACCTTAAATATACATCACAGCAGCCATATGGTGAGTTTCACAAACAGGTTTAACTACCACAATGAAATCGCTGTACTG
TCTGAAAGCACTGGACGGAGTCTGAACTGTGCAAATTAAAAACCGTTGATGTGCCTTTTAAAACAGATGTTTACAGTC
AGCAATTCAGCGATGACATTTTTCTTTGTGAATGTACAAACATGTGCCATTGCGATGTGAACATGAGGTCATGCTTAA
ACAAAAACAACACACTTAATATCATGCTTTCCGTCTTTATAACACTTCAGTAGCATTCAATTCCCTATAGTGCACTGG
ATTGTAGAGTGAAGCTAGATTCCACAACATAAAACATAGGTTGACTGGTGTGACAGATGAAAAGCCTCAGTGTTTTAG
TGTGTTTTAGTGTGTGTGTGCGTGTGTGTTGTGTGTATATATTCATCCTATACACCCTTGTGTGATAAAGGGTACAAG
GTACTGGTTGCTAACAGATAGTACACCGTGGTCAGTGCTGCTTGCTCGATGGAGTACAGGTGATGGTCTTCCACATGT
GGGCAGCTGCTCCTCATGAACTGGGCCAGGCGGAGCAGGTACTCCAGGTTGTGGCCCGTCTTCCCACTGGACACAGCG
ATCTGGGCGCCGATCGCCGCTGGGCTGGCCCTCCCCAGATACAGGGGGTTGTCAGAAGTGGCAATGTACACCAGTGCC
TGAACTGGTGACTGGTTCTCCCCCTGGGGGAAAAACTCCACCATTTTGGTGATGTAGCCACCGCAGACTGTCTCGCGC
ACGTCGAGGTACTTCAGGGACTCCTGAACTTGAGTGCCTGTCACCTCGAATGCCACACCCCAAGTGCTCGCGTCATCA
TCTTCAATCAGCGTCACCACTCTTCCGGGCAACTCGTCGTTCCCGCGATGGAAGTTGTCTCCGTGCCAGAAACGTCTC
TTGTAGCCTTGAATGTAACCGACCTGGCTCCTCTTGTACCTAAAGTCAGGCTTCCACACCAGTGACCCGTACCCGAAG
ATCCACAGGCTGGTCTTCCCGGCGGTGATGTCTTGAGGCTTCATTTCGATCGGTAGTTTTGATGACGTTCAGATGAAT
CGATGGCTCACTCTTTCACTTGATGTTACTAGCTAAGATGACTAGCTTGCTAGCGTTAGCTTCCTCCGGTCCAGGAGC
AGAGGCACCGGCTGGCTCGCAGGTATACCGCGAACGGTATTTGAGCCCAACTGCTTGGCAGTTGCGAAGATGGAATTA
CGCAGTTGATTTTGCCTTGCACGATGCATTGCCGTTGCATTAGAGGTGCCGCATTATGCCTTAAAAATCCATGGCAGC
CGCTTCGGCCTTCAGATCAGTGGACGAAAAAAA

>Sequ28119EST2
TACTTAGTTAGGGTAGGTTTTCGGTTTTTTTTTTTTTTTTTTTTTTTCTACTATTTTATTTCTTTTCATTCTCATACAT
AATGTTTCCCAATATACTGTAATGTACATGACGATTAAAATTAAGATGTACAAGTTTCTTCTTGCAAAGAAGAGAACA
TCATTGTGTTAGAAGGCTGGTTTCTTTACGATTCACTTGTTTAAGAGTTGCTGTGTAAAACAAATTTAGAAAGGTTTT
CTACATCATAATAAAGATGAGTTTTAATGGGGCGTATTTTGAGTGTGTTTACTCAGTCTTCAGGCCCTCCTTGATGAG
GTTGAGCTCATAGCACAGGGTCTCATAGCGATATGCCATACGGATCTGGGCCACGTTGGTCTTACGGATGTCGTTGCC
CTCTGGACCCTCCTTCAGGTAGTCGTGACCCAGGCAGTGCAGCTTCTCCTCATGAGACATGGCACTCTGCAGCACACT
GTTCCTGGAGATCTCACACATGTCGCAGGTGCTGAGCTTGAAGACCTGGGCTGCAATGGCGTACTCTTCCATGAGGGG
TTCCTTGGTGTAGTGGAACTGCATGGGGTCGTCGGTGGACAGAGACACGACCAGGCCTTTCTTGTGGAACTCCAGCAG
CGGGTTCTTGGCGTACTCCAGGAACAGGCTGTTGTTGCTGAGGGGGGACATGGCGATTGGGATCTGGGTCAGGAAGTA
CAGGTACTGCAGCACAGGGCTCTTCTTGAGGTTCAGGCCGTGAGAGATGTTGTCAGCGGTCATGAAGGAAGCCAGCAG
ATGGGTGACGGCACCGGCCTCGCCGCAGTGAGGCCTGAACATGAACGTGTTCATCCCCCTCTGTTTGCGGAGCTGGTT
GAGCACAGCGATGTTTGCATACATGTAGTAAATGTAGTAGGTGTAGGAGGGGTTCTTGACGATGTCCCACTCCTCTGG
CTTGGGGCTCTTAGTGCAGAACATGTGGCCGCTGTGCTTGGACTCATCGTCCACACTGTCGAAACCTGTCACATGCTT
GAGGAAGATGCTGAGCTCTGGGTTGGACAGTGGGTCGATGGTGGCCTGGAACACGGGAAGGAAAATGTTCTCCAGCAT
CTTGCCGAAGTGGGGCACGAAGTTCCTGCCTCTGAAGATGTCGTAGATCCTGGGTACTTGAATCATCCATTTGAGGTT
AGGGGAGAAGACTCTGTGCTTGACGAACCAGCCGGAGAGCTTGGTCCACTCATTGGGGTTGCAGCCATAGATGGAGAG
ACGAGGCTCAGCGTACTGGTACTTAGCATCCTCCAGGTCACTGGCTACTTCCCTGATAATGGTGGCAAAGTACTCTCC
GTTGATGTGGTTCTCTGTCTTCAGGTACAGGTCGCGCAGCTCGCTGGCTCCCACGGGGTTGTACTTGGCATTGAACTT
ATCAAAACGCTGGAAGGTTTGTCGGCCAGCGTGCACATCCAGTGAGTCCACAGTGAGGTCATAGGGGTGCAGGTTGAG
CGTCTGAAACAGCTCCCTCATGGTGACCTCCCGACCTTTAAGGTTGTGCACGACGCGGTCAGCGTCCACGCGGTAAGA
CTTCTTGATGAAGCGCAGCAGGTGCTTCTGGTTCATGCAGGCGGCAGCGTGGATGTGGGTGTCGACCTTCCTACAGTT

GTAAAAGTCCCTGTGGGGGTTCATCTTCAGCTCCTTCATCTCCTCCATCTCATTCAGCATCTCATGCACGTTGAACTT
GGATGTGAGGAACTTGAGACGGCGGTGGGTGTAAGTCTTAGTTGGGCCCTGTGCAATGAGAGCAATGAGGAAGTTCAT
ATCATCGATGAAGGTGTTGTAGTCAGGGCAGGGCAGGTCTTTGGGCTGATGTTTGTCAGCAGCTGCAGCGTCGTTGTA
CACATAGATGACACCATCCTTCATACGAGCGACGTAGCCCAGATTCTTTGGCAGGTCCTTGGTGTCAAAGGGGTCTTC
TCCATTCTTTGGAGGAATTGTGAAGACTGGCTGCAGCTGATCCTCAGCTTTATAGCTCTCTCCCTCGATCTCACGCAG
GTACTGGGAGGCCGTGTGTGGGAAGCGCTGATAGG

>Sequ28138EST2
CGGTACGTAGTTCGTCGGTTTTTTTTTTTTTTTTTTACGTTAGTTATATGCTTTTATTATAAATTTTAGCAGCCACTT
CTTCAGACGAAGCTGAAGTATAACAGCTTGAACATGTAAAACAAACATTAATTACACAAGTGACTCATTATTTCCTGG
ACCCTTTCCTCAAAACTCATCTTGGTTGCTCCTGGTGTCTCGTAGCTGTGTGAGGGATGTCAGCACCTCCTCTATCGG
CCTCCTGCCCAGCTGTTTACCCCGTCTGCTCCTCACATTCACTGTTCCACTTTCACTCTCCTTATCCCCCACCACAAA
TATGTAGTTGTACTGCGCCAGTTGAGCAGAGCGAATCTTCTTATTTAAGGTAGCTCCCTGATCGTCGCTCAAATCAGC
CATGAAGAAAGCTTCACGGAACTGCTGGACCACCTGCTTGGCGTATGACTCACTGTTGCCCCCCACAGGAATAACCAT
GACCTGCGCTGGAGACAACCACAGTGGCCATTTCCCTCCAAAGTTTTCGGCCTGTATAGCGATCATTCTCTCCAGTGA
TCCCAGCACTGCTCTGTGGATCATCACTGGCCTGTGCAACTGTCCGTCTCGCCCGACATACTGAAGATCAAATCTGAT
TGGCAGCTGAAAGTCCAACTGGATTGTGGCACACTGGTGTTGTCTGCCAATAGCATCTTTGATCTGGATGTCAATCTT
TGGTCCATAGAAGGCCCCGTCTCCTGGGTTCAACTCCCAACGTTCACCAAAGTGCTGCAGACTCCTCTCCAACTGCTG
CTCAGCATTATCCCACTGTTCAGGCTCCCCTAGACCATGGTGTAGGACGTGTAGACAGTTGGCAGTGGAAGGAAAACC
CAAACACTTGATACACACTCCTCACAAAGTCCAAACAAGCCACAATCTCTTCTTCCAGCTGCT

>Sequ28144EST2
AGTACTTTCAGTTTCACTTTATTGGGTTGTGTTTACTGTACACCAAATGTAGCCGGGGTTACTTTTATCTTCTGTATT
TTATTGAACTGTCATGATTTTTCTTGTGACTAATAAACACCTCAACCTCCCAATGTCGACTATCACAAGACAGTATCT
TTTTCTTCACTTTCTGTCACCCTTGCCAAGAATTGCATGTCACCATAGCGATGTCCGTAACAGATCAATTTATTGTTA
ACAATCACTGTTTTGGTATAGCGTACTTCAGCAAATGGAGTAAAGAAAACACAGATTAAAATAAGCTAGCTAACAATC
GCAATAAAAAGAAATAAATAGAAAATAATCCGATTAAGGTCTAGTAATTCCATGTTCATATGAATACATAACATCTAC
AGTATAAGTTAATAAATTGAAGCTACTGTATAAATACATCACCAGCATGCACAATATTGTATTATGGAGAATATTGGA
GCAGTTATCATCTTACACTGAAGAAACAGTATAATAGGCAAGTGCATTTCTTGACCAAGAAGAAACTGAAGACAAAGT
AAATCCACAAACCTCAAATCTGCTTTTAGAGCTTCCACTTGACATTGCATGTGCTCTATCCACAAATTGTACTTAGTT
GACTAATCAAGTCTGTGGCTCCGGATGCCGTTTCTCAGATCCCACTGCTCGTTATGAAGATAGCATAACTTGGACAAT
AAGGGTGGACTCACTTATTGTTTCATCATTTTCATCGGTTAAAATTTCCATTCATTTTAATTTTGCTATCTGGATTTT
TGTGAATCAAATTGTTCCCTATAAGCGTAAGGACCCAATAGAAGTTGTTATGGACAACAACAATACTGACAATAAGAA
TTAGGTGTCCAAAAACAGTTAATTTATTCAATAAAGCACATCAGCACATGAGAGGGACAAGCTGCACTAACTTTACTT
TATGAATCTGTATTTTTAGTTACAGTATGACTGTGTGACTGAGCTGGACACATACCTGACACAAAGCAAAAAAACATT
TTTTTTATGTCATCTTCAATAACAGAGACAACCTGCTTCTCACTCAAGAGATCGTAGTGTGGTGCAACTTAAGATGTC
GGGTACGTTTGAAGTTACGCGATGAAGTTCGACACCCCTAAAAGCCCGTCTTCACCTCCCGTCTGTAACCCGTCTGCT
CTCTCACTCATTCTCTCTCTCTCTGTCTGTCTGTCTGTCTGTCTTCCTCTCTCACTAAGCAAGGCCATGACATTCAAG
GTTACTTTAATTTTCTTGTTGCTCAAGCATCGGAACTCAATTTCTGAATGCTCCTCCCCTCACCAAATACAATAAGAC
AGCTCAGCTTCATTTGCGCGATAAGACCAGTCTCTGCCAGTGTGAAGGTATCGTTACCCAATAGTCACACTGTGATGA
ATGACATACCTCTTGCTTCTAAAGTGGTATTTTGGCAAATGCTGCAGAGGCTATCAAGGAGGGGGTTTCATTGTTTTA
TTATTTTTTTTAATTGTAGGTTATTTCTCAAACTAAAATCGTCTTCCTCCTCCTACATTTTTTTCACTTTTAATTGTG
GAAAATAAGAACAGCAGCCGCGTTAGTACCATCAGCTAAATTGGCCGCCGATGTTCCACTGCCCCCTCCCACCCTCCC
TTTTTTTTAAATTCTAAGAACTTTTTTAACTATTATTATTATTTTGTCATCATTTGTGAGCGCGCGTGTTGTGTTGT
TTTGGCAGAATGAGGTTTTGGACTCTTTAATTGTTGCCCTCTCCTCCCTCGTCGTCCTGCTGGTCGCTCGTCCACAGC
GTCAAGTTGTCTCGTAGCAGCTGCATGATGAGAGTGGAGTCTTTGTAGGAGTCCTCGTTGAGGGTGTCCAGCTCGGCG
ATGGCGTCGTCGAAGGCGGTCTTGGCCAGGTGGCACGGCCTGCTCGGGGGCGTTCTGGGATCTCGTAGTAGAAGACGG
AGTAGTTGAGAGCCAAGCCCA

>Sequ28145SNP2
AATGGTTTAATGATGTTTACAACAAAAGAGAACTGTACAGTTACAGTAAAAGATTATCTTTATATGTGTATAAAAACA
AAACAAAAAAAAATACAGCAGACAAATGCATCAAAATCTACTATGTATCCTGATTCACATACTTAAAACTCCTACACAA
TCTTTCAGTTCTATGCCGCCACAAGTGGAGGTCTTTTTTTTTTTTTCTTTTCTTTTTTTTTGTCTTGTAGTCAGATCG
GAGCAGTGTCCCTCAGTGTTGTAGTCCATAATGGAAACCAAAACTCCTTACATTAGAGTCCTGGAAAGTCAACTCTTG
TCAGACTGCAGAATAAGAAAAAAGGGAAATAAAACGGGGACACATTCCTTTAGATGAGCCCAAGCTTCCAAGGTGAGT
AGTAAATGTTTTACAGAATGCATTTCTTGAGATACACAGACTTAATATGTACATGGTTGGCGTTTTTTTTTCTTTTTC
TTATTTTTCCTCTCTTGTTGAAAGCCATGGTCACTTGGGCTGTGAACTGATAAATCACACACAACACACCCAAAAACA
TACAAAAATACTATATCTTTAACCTACCAAGAGTAAGACAGTTTTGCAATTAGTTCCATGCTGTGACAGAAAAAGGAG
ATTACAACAAACAAATGAGAAACAGACGCTTTTCACGCAAGTTTATCTCAGAAATATACCAAAATATACATCTAAGCT
TTGGAATTACTGAGGCTAGGACTAAAGCCAGTGCTGGGTTTGTCTCTTAATACACATGCTGCAGGGTGGAAGGGAGAG
AAAACAGAGAGAGAGACAGGACAGAGAGATATCTTTGGTATCTGCATTTGTCAAAAGTTAAATATTTGGATTTTTTGC
TTTGGGATAGCGCTGTGAGGTCCTACAACCTCACCTAGAGTAAAGCATTAAACAAAGTGTCACTGAAGCCCGTCGGCC
AGAGGTCTGAGCCGGAGGGACACAGTCCATGCAGAGTGAAGGACCATGATGCATAGTGTCACAGCTGATCAGAGTCTG
TAGTGAAGAGACAATGTCAAAGGAGAGAGCTGAAGAGGGGAAGGTAGGAGGGAGGAAGAGGAGGGGGGGGGACAC

>Sequ28147EST2, Sequ28147SNP2
TAGGTTCGGTTTTTTTTTTTTTTTTTAGTTTGGAATCTTCTTTAATTCGATTGTGAAAACATTCACATCGACACATCAGG
ATAACTTCAATACCAGTGGTAATACAGGATTGTAGATTGGCGCTTCAGTAAATGCGTCAGCAGCAGTTCCATTTTCAG
AAAACACACTCCAGATCAAATTAGACCAGTTCTTGAATGAAACTAGGCCAAACTTGCACACTAATCTCAGATTTCTGC
TGCATGCTACAAACCATACCATCAAGTCTTGTTGTGTGATTGAAACTTTTTTTCTTCATCTTCTTTGGCTGGAACTAT

ACATGAAAGTAGCTTTTTTGATCTGGATTAAATCTTCTGAAAGTAGCCTGCTCTTCAGTTTCATATCCAACAGCAACA
AAACACCGAAACCCATGCAGGATTTTAATGGCTGGAATGTGAAAGAGATGAAGTACAGCCACAGATGGGGAGGGGGTG
AATAAGATGGAGAGGAGATGAATTTTGGAAATTACACTTGTGAGACCCAACATGGGGCACACACACCTTTAATGCAAA
CAGCAGTACTACAGGAAAAACACTGAAGTTTGAGAATGTTTCCGTAATGATTAGAATACAGCTACACACAAAACTCCC
ACTCCACCAGCAACATACTAAAGGCAATCTTAATTATTAAACCATGAATTACATCCAGAGGAATTCCAACCACTTATC
ACAATTAACCCACAGTGCATTAAATCTATCTATATAGATATTTATATGTCTGTATATATATATGTATAATATATCCTG
GGGAATTAAAGACTTTTGCTGCATTAAACAGTTCAATGAACAATTTCTTATAAATAAAATCAGTGGAAAAAAAATCTGA
AGTCCATACACATTTCTCTTTACAAATAGATCCTCAAGATTAATGCAGAAATTGTGTCAACTCCCAGGTCGATATGGG
AGGAGAAAAAGCTGTGTGGCCCTTTGGTCAGCTCTCTAAGACCAGAGGCCAAGCTGCCTCAGCTGGCCTCAAGGCCTG
AAATAAGTGGAGGGAAAAAAGGAATCTTCATCATACAGTTGTTTTGAAACACACACACGCACACACAAGACAGACAAA
TCACTACATTGCGCATTTACAAGTTGACTATCAAATTGGGACGACTGTACACATTGTTGAGATAAGACCATATTTATG
GAGCCGTTTTAATTCATTTTTTCCTCGCCTTCACTTCTTTCATCTTGTGTGGCAGAAATTTTTACAATTTTTCCCCGT
TATACACTTTTTCTCCAGCAGCAACATAAAGGACAGATCTTGTAGTGGAGATGTTTGGATGTTTATTTCGTGGAGAGA
ATGAGGGCAACGATGAGCCCGTAGAGACCCAAGACCTCAGCAAAAATCAAGATGAGGATCATGCCCACAAAAAGCCTT
GGCTGTTGAGCTGTGCCTCTCACGCCTGCGTCGCCCACGATGCCAATTGCGAAGCCTGCTGCCAGCCCACTGAGGCCC
ACACTAAGCCCAGCTCCCAGATGAAGGAAGCTCTTGTAGAGGGTGACCTTCTCTGAGATGTTGTTGGCAATCAGCACT
GCTACTACCAGACCGTAGATGGCTATGATACCCGCCATGACCACGGGGATGATAGACTTCATGATGAGCTCCGGCCTC
ATCACAGACATGGCGGCAATCCCTGTACCGCTCTTAGCTGTGCCATAGGCTGCTCCCAAGGCGCTGAACACCATAGCC
GCAGAGGCACCCATCACTGCGAAGAACGGAGAGTATTCGGGGCTTTCGGCCGACATTCTTCCTCACACAGTATAAAAA
TAAACTAAATATACAAAATTAATCAGCAATGACGGATCAAAAATCGCACTTGGCGAAACCTATCGAAGGCCACAGTCT
TGAAATGCGAAACTGAGTACACCAGGAACAGTCTGACCACCTAACTAGCAATGTAAACGGGATAGTCACGACTCTTCT
GATAAATACCCGGCGCAGCAGCC

>Sequ28148EST2
AATGTGAAGTCTTTTAATTTATCCATCAGAATAATAAGGGAAAGTCTAATGCCACTTCATTCAGTCTACTCACAGCAG
CTGACACAGACACATTGAGATCAAAACTTCCAGAGCTTTATTGATGCCATGTGAGCTGAACAAGAGCAGGTCTCGTCA
GTAAAAACCCTTTAAACTGGAAGAGAAACAGCACAAAATCAAGTCACCTCTGACTGGTGTAGAGACAGACAAGCCTAT
GCGTTTCCCCATAAATCAATCACATCCAGTGACCAGCCAGAGATGAAGCGCCATGGATACTGAAGACACTGGGCTCTT
AAAGCAAGACGCAAGTGAAGGCCACTGCTTGTAAACAGCTTTTGTTGTTTTAGAGTTATTATTTATTCAAATTGGTGT
ACTTAAAAATAGAAACTATATTGGTGTGTAATTTGTAATTTAACGTTTCCTTGTGTCCATGTCTGGATAAGAAGACAG
TATTTTGAGTGACCATCCTCAGGCAAATCCTGCTAATGAACAAACCTGAATGTTTGCCAATGAACTATTAACGCCAGT
TAATGTTTGCAATGTGAAAGTTAACATCTGTTGGACTGAGCGACTGGCCAGGTCTGTCCACATTTTCTCTTCTGATGT
TTTTCCCTCCATCCTTATTCCGAGGACTGACTCTAATCCTCTGCTTTGGCCTCCATCTCCTCGGCGTCATCGTCTCCA
TCCACCTCAGCGTTCTCTCGCTCTAACCTCTCCAGCTGCCGGGCGAGCTCTGTCTCATCCGTGTCTGTCACCACCTTG
GGCTCCATCTGGATGTTAAAGACGCCTCTCTTCTCTTCAATCTTCTCCTTGATAGCAGCCATGGCCTGATTGAGGACA
GACAGACCCTCTGTGCGCTCCAGAGTGGTTGTTGTCATCACGTAGCGAGGGGGAGCGATCAGGTTGATCTTGATGGGC
ATGGCCTCTGTGGAACAGCCCAGCCCAGCCCTCAGAGCTTCCTTCACTGCATCAATGCCCTCATACCCGTAGCATGCC
ACTTCAATGTCTGCTCTGATTTTGACAGCCTGTGGAGTGAGTCGCCTGTTGATGTTATCAATCAGCACACTCCTCTCT
TCCTCTGTTAGGTCCAGACCATCCAGAATGGCGGGATCTGATACAGCCTGTTTGAAGACATCATAAGCTCCGTATCCT
GGCCGCTTATATTTTTCATCAAAGACCCAGGCAGTGCGCTGGTACAAGCTTTCTAGCTGCTCATCCTTACTGTAATCC
AGCACTTCTGCCACGTGTCGCAGGATGCTGTACACAGTTTTGGATTTGGTGAACTTATCTTCACACTTGATGGCCTCC
TCTGGGGAAACTCTTCTTTTTGATAAATCAATGTATCCCTTTTCTTTGTCTACTCGGATGACGACCACGCACTCATTG
CGGCCTATGCGGATGAGCTTGTTGATGGAGCGGATACGTCGACGCGACAGCTCGCTCAGAAGGATCATGCCCTCGATG
TTGCTGTACTCCAAGAGGCTGACATAGGCGCCCATCTCAGCAATGGACCTCACGTTCACCATCACAACATCCTCCACC
TCTGGGAACCGGTGCTGGTAAAATCGACAGCTGAGCCCCGGCATTGCTGTGTCATATGGTTACAGAACGATACACTGG
AATGTTCTGTACTGGCGGTGTTTAAATGTCCGATTATGTTTTCTTTCGGGGTTTTTTAAATGAAGAACTCGGGCCCGC
TAGCTCACATTCATGGGTGTGCTAATTTTTCGACC

>Sequ28162EST2
GGGTAGGTCGGTTTTTTTTTTTTTTTTAGTTTAAGTAGTATTTTATTTTACATCTGTGCTTTGAAAGGGTCAACCAAC
AAACTGAACAGCACATGACATTTACACATTTCATTTAGTTTCACTTCAACAAACCATCTTAACACTGGAATGTTATCA
AAAGGCAAGTTTTAAACTACAAGTGTTAAACTACAACTTCTCAACAAGTTGTTAGCTGTAAACTCTAAGACCAAGACA
GACAAGCTATATTAGACACTCTTCAATGTGTTAAGTGTTTCAGATACTGAATGTTATCCCAGCACTGTGCATGTTTAA
ATGGTGAGCTGTGCGGCCTCCTTAACCAAAGGCTTCAAGGCACCGAGGTCTCGCAGGAGGGAGGACACCCCCACACAA
TACCACAGATCCCCAGTGCGGTCTGAGGGAGCACTGAAAGACTCAATCTCCACTCCCTCTCCGACCAGCAGTCCAGCC
ACTGAGGACAGGAGCTGAGGACTCGCAGAGGCTTTGAGGAACAGCAGATTCCCAGTGAGAGAGACGGGTTGTCTGAAC
ACGCTGTCATTCAGCTCCAGCAGGACTGGCACACCTCCTTGAACTGAACCACGTGCTTTGTAGCTGCAACCACTGGCC
ACGATCTCCACCCTACACACACCATCAGTTGTCCCGTCAGATGCACAGTGGCCCTGGTTTACCATGATTCCAGACTCC
TTGGCGAGGCTCAGTGCGTTGATGAGGTTCGGGCAGCAGCCTGATTCATCATTCATCAGTCCAACCAGGACTGCTGAA
GTCATGTAACCAGTGGAGGACTTCATGCAGTCACCTTGAGTGGTTATTTTAACTTGGCTGAATGGTTTCTTAGAAGTA
GTGCATGACTGCAACACAGCTCCAACGGCTTCTCCGAGTTTAATCAGCTGCTGAGATTCCTGGGAGAAGGTGCTGGCC
AGAACTTGTGCATTCACTGCTCCAAACAAATTCTTGCCCTTCACCATGTCCACAATCTGCAGGGCGATGTCCTCCCCA
CAGCGCGCCTGAGCCTCCTTGGTACTGGCTCCCAGGTGAGGACAGCTGATGACATTGGGATGGTCCACCAGTGAGCGA
TTCTTAGGTGGCTCCTCAACAAAGACGTCCAGTCCTGCTCCTCCACACTGTCCGGACTCCAAAGCTCTGACCAGGGCA
GCCTCATCAATGATGCCCCCTCGTGCACAGTTCACAACCTTCACTCCTTTCTTGCATTTAGCAAATGTTTCGTCGTTA
AGCAGCCCCACAGTAGAGGGCATTAGGGGAGTGTGGACAGTAATATAGTCACACTGGGGCCAAAAGCTGCTCCAGAAG
CATCTGCTCCACCCCCAGCTGGCTGACACCTCAGGTGGAGTGATTGGATCATAGCCAATAGTCTTCATGCCAAATGAT
TGCATTCTTGAGGCGACTTCTTTTCCTATTCTTCCAAGTCCAACGATTCCAAGCACTTTGCCAAACAGCTCTGCACCC
ATGAACTTTTTGCGATCCCAGTTCCCTTGTTTCATCGACATTGCAGCTTGAGGCACATTTCTTGAGAGGCTCATCAGC
AGGGCACATGTCAGCTCTGCAGCACTGATCGTGTTACCGCTTGGTGTGTTCATGACAATAATACCCTTTCTGGTAGCA

GCATCAACATCCACATTGTCCACACCGGTGCCAGCTCTCCCAATGAGTCTGAGATTATTAGCAGCATTTACAACATCA
GCTGTTACCTTGGTTGCAGATCTAACCACCAGGCCGTCATAGTCCTTTATTTCCGCGAGTAATTCATCCTTTTTCATG
TTCTGCTTCTCCGTAACGCGGATGCCATTTTCCTCCAGAATCGTCCTGCAGCGAGGGTCCACACTTTCACTGATCAGG
ACAGTTTTGATGGAGATCGGGGCCATCTTGGAGTTTGTTCTCCGGCTTTGCTATTTTGGTGGGTAACTTGTGTTCGAA
TCGGTTGATGCCTTGGTGAGGTGATGGACACGGGGAGTAGCTTTTCCCTCTGTTGGTTTTG

>Sequ28178EST2, Sequ28178SNP2
TGACAAGTCTTTATTTTTTTTCATTTAATGTTGAACCACATTGAAACAGCAACACCAAAGTAACACGTTGGCTCAGTG
TGTTAGTAGATGCTTCATGCGATACGTACGAATGTTCTCTATAAACTGGGTCATATACAACAGTCGGCTGGATACAAT
ATTTAATGATGTATTTAGACATTTTCACCCATAGCCAGGTTCAGGTGACAGATGGTAGTAGTAGTGTTGTGCTGTCAG
ACTACAAACGAATCAACAGACTAACAGACTCGCTGGCAGATCAGATCATTTATTATCACTGTGACATCTCTACTGGGG
CTTTCAGATGCTGAGGTAAAAGACGAAACAGTGTTCAGAAGCTACTGAAACGTCCTGCAGTGTGAAAACAAACAAATG
TAACACTCATCTCCCTAGCGTCTTTACATGGCTGCGGGGTTGTTGATCTTGCCCATGAAGAGGATGCTCTTAGTGGAA
TGCTCCAGGATGAAGACCAGGAAGGGCCTGTTGAGAGTCATGGTGTCGGGCATACTCATGGGCATGACTTCAAGGGTG
GTGCCTGCTGCTGCCTCTGTTCCTGTTTCATCCACGCTCAGCACGGCCTGGTGGGACACCTTTGAGACTTTGAGCTTG
ACCTCATCAGACATTCCAGAGAAATCAGCGTTGTCTGAAAAAGCGTTTGTCATTCCTAATTCTTTAAGCGTGTCGTCC
AGGGAGGCGTCAGCGGAGATCGAAAACTTTGGCAGGAACAGATCCACAGAATTCCTGAAGAGCTTGTCATGCCAGTTT
TGAATGTAGTCCTTGTTGATGTAGCCCTCCACCTCTGCCATCTTGCCCTCATCAGGCAGGACGATCATCATGGAGGTG
TTGCCCTTGTAGGGCAGCATGATGACGGTTGTGTGGTTGTCAGCGTCCTGATAGAAGTCGTAGCGTCCCATCCTCCTC
ATCATGTCCACCTGGACTTTGGTGGTTTCGTCCACCTGGAAGTCTGCCTTGTGTGTCTGGTTACTATCGAAGGGTTTC
TCCCACTGTCCTCTGAAGTAGACAAAGTTGATCAGCACCATGGCCATTTCAGGGTCCAAGTCCTTCACCATATCTTTA
ATCTTGTCCTGTGTTTTGCCGGCAATGAATCTGTTGATCTCAGCTGCAGCCTCAACGGGTTTGGTAAAGTCGACGTTG
AAGCTCTCACCGGAGTAGAAAGCCTTGACATCCTTCAGGAACTTCTCCAGAGGAGTGAAGCCGGAGCGCACTGCCACA
GCGTTGCCGACATCCAGCTGCTGGTCCTCCTGGCTGTGGCCAAGCATGTGGAAAAGATGCTGATATGCTTCGTTGATC
TGTGCCTGGTCATGACCGCTGTAGCCCAAGGAGGAGAAGAGCTGGCTTTGGGTTTCACCACGGCCCCTGTGGACAGC
ATGGACAGGGCTGTGGAGATGCCCAGCGGTGAGTAGAAGATGTTCTTCCCAGCTGCAGCCTTGGCATTCAGACTTTTG
TAGAGTGCAAAGCCAAAGTCGGCATTGGGAGCGGACAGCTTGTGGCAGCTCATCTCTCCCTCACTGCTGTGGCTATGG
TGGTGATGGTGGTCATGGTGGTCATGGTGGTCTGCCCAGGTT

>Sequ28205SNP2
CCTGAAGGATTATTTATTTTTAAACCTTTTAATAGAAGTTGTTATGAGCATAAATGAAAGCTGGAATGTGGTGCTCTT
TTGGCTTCCAGATAACTGGACCAAAGAACTAAATGTCAAACGACACTAAGGCACTGTTTTGAAAAGAAACGTAGCCAC
TTTATTCAAGCAAACTCACTCTGTCTACATATAAGAAGGGTTGAGCGGTTGTGTTTAGCCAAATCCATTTGTTGGCTC
ATCTTAAGGAAATGAGAATACCAAGTTCACATCATGACAGGAAAGTCAAAAATCATTTGCATTGTCAATCCTGCTCTC
CTATTCAAATAGCAATCTGTTAAAATGTCTAAAATCACACAAAATTCAAAAAGGACACTGACATATCAAACTAACAAA
GTGTCAACATCACACAACATCTGTACATCACAAACTGAGCTTGAATCCTTCTCCCATTCACGGTACTGTTGTAACGCT
AATAACCGCAGTGCAATTTATCTAAACAGCCCAAAAATGAATTCTTAAAAAGGAGGACCCCACGTGAAAACCTATTAA
CGTCACCTGACTATCATTTGCACATAATAAACTCATTAACTCTGCTTTCAGTGCATTTAACATCACAGATAAAACAGC
ATGACAAAACAAGTTAAAAACAAAATACACAGTCCGTCTTCGGTCTTCGGTGTCCTTTTCGTTCAATTTCTCTAAAAA
CTCAGCGGCTGCTCCAGCGACCCTCCCATCTGCAGTAAGGCTGCACTGAGTCCGGCCTTCAGCGCTGAGTAGCCGGGC
TGCTCACTGTACTGCAGAGCCATCACTGCAGTCAGGTAGTTTTGAAATGCACTGGAAACTTTCATCTTCCCAAAGCAG
TGAGTCAACAGTGCAGGAACATCCTCCATGTACCTCTGTTTCTGTGTGGCTACCTGGTCCGGTTGAGTGAGGACGGTC
CACGGCAGCGTCCCTGTGTGCCATCGCAGCATGCAGTAACCCAGAGACTGCAAGTCACTGCGTCGGGATGGAGCTGCT
CCCTTATGTGCATCCAGGCTGGATGAACTCTATGGTCGCCCTCGTGTGGTGTCCTGCTGGCTTCACGGTACTCAACAT
GTTGGCCTCCTGGACAGTACCTGAAAGCATGGCAGTATCCTACAAGGTAGACCTGTGAAGTCTGTCCTGGTTTGATGT
AGATGTTTTCTGCAGTAATATCAGCATGAACATATTCGTTTGAATGGATATACTCCAGAACATCCAATAGTCTACAGG
CAAGCTGAAGAACAGCTTTCTCAGTCAGAAAATCATCCTCATCCTCAATGATGGACTGGAGAGACTGGCCCA

>Sequ28217EST2
AAAAGTGTTGAGTCAGAGCTATCTGTTGAGTGTCAGCATGGTAACCATGGTTCACACTCAGCAGCCACAGACACACAC
AGGCTCCAGGACACCTGACAGAGCTGTGAAGCCAGCTGGAACCAGGCCTCAGGATCTGGACCCACCAGGCCCTCTCTC
TGTTCAGGTCCAGCAGTAAATTAGTTTTCATGCTCAGGTATGTGGTCTGATATAAGCCGAATGAGGAAGGTAGCTGGT
GCAGTACAGATGCTGCAATCTAGCTGTTACACATGATGAAGGCCCAACAACAAAGAGGTGCAGGTCTGCAGCAGCCCC
CCCCCCCTTCAGGTCTGACTGAATGCTGTGTCCTAAATGCGTCCTACATCCTGGATGTAAGCAGGTTGTTAGTACAAC
ATTAAGTTTACTCACAGCAGCCAGGACAATACTCTAAACTGCGCTGTTAACACAATGTTCTCCCACAATGCACTGCGC
ACAGGAAACGGAGGAGCTGCTCACATCTGGTAAACATCCAGATGAACTGTGTTGGATTTAATGTTGAAGCTGTGTGAA
CAGACAGGAGGTTTGTGCTGCACAGACTGGTTAAATGAAGCTGGGTGGCTTTGAGCTCCTGCAGCTCCCGTCAGTCCC
AGTCTCAGACCCTGATGGGCTGCAGAAACAACTAAAACATTCAGATGTAAGTTAGGATCAATGATGAAGACCTGGTCT
TTATGTTCTGGGCCTGACTGCTGTTTTTCTATTAGTTTGGGTTAGAGCCAGTTAACACAGTCTTGTTCTTCAGCAATA
CTGTGCTGATTAAATGTTAGTAAACACACTACTGGGGTCAGACCAGGCTGTGTGTACTAACTCTACATCGGGACTAAC
TTCCTGGTCTGTACTCTGGATCCTGGAGCACCACATTCACACCGACACAGACCCTCTGTATCATCACTTTAATACATG
GCTATTTTTGGTCTGAACAGGGCTGAAGGTGTCTGGCGACAGCTTCTGTCACTTACATACAGTTTGTATTAACACGAG
GAGGAGGCAGACTGGTGGCTGGGCCCCGTAGGGACCACACTAAGGGCCAAAGTGAATGGACAGTAACACCACACACAC
AGACATGTGAGTGGGGGCCGTGAGCTCATATCTGGATGGATCTCACACATGCAGAAGGACAGAAGAAAACCCAGGAAG
ATGTTGGACACAAACACCAGGCACATGTCAAACATACATTTTGTTGAGTGACTACTGCACAATGAAATCACCACGGCA
ACACACTTTGACCCCAAGAATGTGAAAACTTTTAAATACTGTTACTGTTGATACAAGAACATGAAAGGAAACAAAAAA
ACGACAGTCTTTAAAATGTTGCTGCAGGGGAGCGTCAAACTATAGAACAGACTCCCAGTCACACGTCACCTGTCCTCT
ACTACAGACACACTAACAAGGAGGAGACACTAACGCATCCTGTACTCTGACGTCTTCGACATCTCACACAGCTGACCC
TTGAAGTCGATGTCTACGGTGAAGTCCAGGTCCCTATTGTTCTTGACGTTTGGCTTCATGCTGATGGTGCCGAAGATC
TCCTCTCCAGTCTTCACAGTCAGGTAATCATCCAGGTAGAACACAGTCTGCTTCCAGTGGGTGTAGGGAGACTCTGGG

CTGGTGGAGAAGCCGGTCCTCTTGTGACAGCGGGTGAACTCAATGTTGAAGTAGGTGACCAGAGCGTGGATGTAGTCG
TTCCTCTTCACCTGCAGGCAGAACGGGGAGGTGAAGGTCAGGTCCTCCGCCTTCACTGTGTAGATATCCACCTCCTTG
ATGAGACAGGCGCTGCTGACCAGCTGCTTCGGGTCCACCACATCAACCAGGGGCTCCTTGATTGCCACCTCCTTGATG
CATGACATGTCAAACCCATACACGTTCTCCCACCAGTGGATTTTGTAGTCCTTGTACTGCCTGTCCTCGATGGCAGTG
ACATAGAGCGTCGCCCTGTCTGGGAAGATGAGTCCATCTGGTTTCAGCCATTTATCCCTGGCGTAAATGACTGTGTTG
AGCATGGACTCATAGAAGAGGCAGTAACCCATCCACTCTGATATGATGATGTCTACTCCTTCCACAGGCAGCTCCACC
TCCTCCACCTTCCCCTTGATAATGGTCACCACGTCATCCAGCTTGTTGGCCTTGACTATCTTCACAGCGTAGTCTGAG
ATGCTGCTGCACTCGATCCCTATAACCTTCTTGGCCCCGGCTTTGGCTGCAAACATGCAGAGGATACCCGTCCCACTG
CCCACGTCCAGCACCACCTTGTCCTTGAACAGGTGCTTGTTGTGGAACATGGAGTTGCGGTATGTCAGAGTGCGAACC
TCGTCTTTCAGCATCTCCTCGTGGATGCCGAAGTGGGCGTATGAGTCAAAGTAGTAGTCCTTTGACGTCATATCCTCA
GCTGCAGGCTTGGCTGAGCTCTCCCCCTGAGAAACCTCCATCCTCTCTGCTGGCGCCGCCATTTCGATCTTCTGCCTG
GCTGCC

>Sequ28224EST2
CCTACGCTCAGTTATGGAAGTGCGGTGGACAAAATAATAGACACATCTGGTGGTATAATAACCCCCACAACCCTCTTT
AGTACTTATTAGCAATATATAAACATATGTGCTTGATGGACTGGCTATTAAATCACAGACGATAAGTCCTGATGACCT
TGGTGATCCCCTGACTTTTCATCTAATGCCACCAAAACATTTTGATTGAAATATCACATCATAATACCAACTGCTGGC
TGGATTAACATCAGATTTTCCTCTCAGACATTCGTGGTGTGCAGAGGAATATTTTAAAAAAAAACTTCAATACCTTGA
CTTTTCATCTATTGCCATAAACTAAGATAATAAGCGTTATACTGAACCGTCTAAACATTAACACAGTAGCATGATCAC
TGTAAGCGCGTTAGCATGCTGTGCTTCGGTACGTCCTCACAGAGAAGAGAGACACTCAGTCTTATCAGCTGGTACCTT
GGTGTTATTTCTATCTACTGTGTCTTCTCATCTCAGTATGTTCCACAATATGTTCTTAGGAAATGTAGTCCATCGTTA
TTTATGTCCGTCTGTCTTCACGATGTCACTCAGGAATTAAAAACATGACTGTTTCTATCTTTCAATCTGCTTCCTTCC
TTCCGTTCTTTCTTTCTTTCTTTCTTTCTTTGAGACTCTTCTCTCAACATGTTGAATGATAAATGTATCTTGTACTGT
AACGATATGGTGCAGGATTTTTAACTACTTTTCTAACCTATAAATGGCTTTAACTGCTTCATGAGACATTTTAAATAT
TTATAGAAAAAAGAGTTTAGAATCAAATCAAGTATTAACACAAACCTAAACAGGGTTTAATTCAAAATACTCATGAGT
GCACAACACTTTTAAACCCAATTTAATTTTATTAAGAAAAAAAGAAAGACACTAGGTTGATTCTGATTGGCTGGAAAC
CGAAGTGATATCACAGCTCATCTTTAGATGTTGTGTTGGTCGCAGACTCGTCAGTTTCCTTGCTGTCATCACCGGTGT
CGTCACCGTCCTTAGCATCATCATCATCTTCCTTGTCTTCCTCTTCATCCTCATCTTCGTCATCTTCCTCCTCATCGC
TTTCCTCCTTAGGCAACACTCCTCCATCATCCAGGAACTTAGACATCGTCTCCAGATCCCTTTTCCCAGTATAGTCAA
CCACCTCTTTGCCAGCAGTTGGGAAATATTTGAGCGTGGGGAATCCACGAATATCGAGAGACTCCACTTCGTTGGCTG
TGGCATCCATCTTGGCTATGATAATGTCATCATGGTCTGAGTACTTTTCACCCAGCTGATCCCAGATGGGAGCCAGTT
CCTTACAATGTCCACACCATGGAGCATAAAACTCCACAAAGACGTTTTTGGTCGGGTCCAGAGCCACAGACTCAAAGT
TCTTCCCCACCAGGACTTTGACTGGTCCTTTGTTCCAGTCCTCTGGGATCTCTTCTGTGCGTAAGTAGGGCTTGGCAG
TGCCATCGACAACCTCCTGGCACATCTGTCGCAGAGAATCTGCTGTGAGATCCCCGGCGGCGATGTTGAACTTTTTTC
CTGTATCCATGTTTATGATGCGTGCAGTGGGGGTCTCGTTCTCGGACACACCGAAGTATGTCAGCACATGAGACACGG
CTGTTGTCACGTCAATTACAATGAACAACATCTTGCCCTTGAACCCTTTGGCAACAGGCCTGGATTTTTCCACCAGGG
CTGTCTGACTCTCCACTGTGGAGTTGATGAATAGCAGGCTGTGCAGGTGGATGCTAGAGGAGAAGATCTTTTCTGCAG
TCTCCTGTTTGAATGGGATGATCAGCTCCAGGCTGTTTTCTTGGATGAACGTGGTCAGATTACTTTTGTCCAGCTTCC
CTTCTTCTGACAATGTAAAGTCTGCTCTGCCGTCATCAAACTTTTTGAAGAGGACCACTGAGTTGGCTTTTACTTCAT
ACTTCTGGAAAACCTCAGGAGTCGTTGTCGTGGCAAACTCTGTGTCAGTCGTATCGAAGGCAACTTCCTTGAACACCT
TAGCCGCCTCACTCTCCAGACTATCGAAGAATCCGACAACAGTGATGCTATGGGCATCGATGAACTGAGCTGCAGACT
GTGCAGAGTCGAGGACTGCAGCTCCAGGGCCTGTACGCCGCTTCAGCCACTGGATGATTCCCTCTGCTGACCTCTTAC
CAGTGTAGTCGATGGGCTGCTCGCGGTCTCCATTGATGAAGAGTTTCAGAGAGGGGAAGCCTCCGATCTCAAACTCTT
CAGCCAGCTCCCTCTCCTCGGTGGCGTCCACTTTGGCCAAACGCATCGCAGACTCCTCCGCCTTCAGGTTCCCAGCAG
CCTTTGCATACTCTGGCTCCAGCTGTTTACAGTGACCACACCAGGGAGCATAGAATTCCACCAGCACGTACTGATTCT
CGCTGAGAGCTCTGGCAAAGTTGTTGATGTGCAGAACCATCACATCTCCCTCCTCCTCTATCTCTGTTGTTTTTCTTT
CTTTGGTGCCTCTTCGGTTTCCTCCTCCTCCTCTACTGACTCTGCTGAAGTCTCCTCTGCTGTCTCCTTTTGATGTCT
CCTCTGACCTGTCCTGGTTTTTCGTCTGCGTCGTCGGCCTGGGCGCAGGAGGCCCACAGCAGCAGGCCCAGCAGGGTT
ATGGACAAAAGCGTGCATGTCCTCATGTTGAGCTAC

>Sequ28237EST2
ATTTTGTCCTTTTTTTATTTACAAGTGCTGAAAAAAATGACATGGGAGACAACCAGTGCAAAATCTGTACATAAATTC
AGTTGCAGGTATCTTATATACATGAGTACTGAACAGGAACGTGAATACACTCTTACTGTCATGCATGTAACAGAGTTC
ACAACACAGCAAAAGACATGGTGTAAAGATGTCCGATTTAGTTGTAGTCCGTGATGCTCAGGCTGCTCATTCTCCCAA
GGAAAGGTGGGGTGATCACACTGGAGCCCAGACAAACTTAACAGAACAGATCAGGGGCACAACAGAAGAGGATCCATG
ACTTCATTGATACACCCTCCAAGACATTTAACATAATTAGTCATGGGGAGTTATTACAGCAAGTTAGAACACACCAGA
ATTTTTGGGTAATGGCCCACAAGTCCATATTGACAAAACATCAAGGCAAGATTCAGCACAGTTCCCCTGATGGACTGG
AAAAGGGGGATAGTATAGAAAAAATAAATCCCTCCTGGCGGGTAAAAAAAAAAAAAAACATTCTATGAAAGGAAGTTG
AAGGTAAAGTGCATAAAAGCTTAAAATCAAAAAAGGTTGGGGGGCCCAAAAATTAAGAGGTACTCCACTAAAACTCGC
AGCACACACAGCTTTACAATGGAGAGAGCAGCTATCATGATTGTCAGGTCAACTGTCTCTCCAGCTTTGTTCTACGAT
GGCGGACACACGCTTCTCGTACTCCCGCTTGTTCTCCTGGTAGAGCTGGGCTGCCTGACTGTTGGCTGGACTGTTGGG
GTTTGGTTCATCAAGCAGAGACTGGATTGATGTAAGAATAGAGGAGACATCATAGGTGGGACTCCAACGATTCTGTAG
GATGTCCAGACATATACTTCCATCTGCATAGACATTTGGATGAAACATCTTTGACACAAATCGTACTGTGGGGGGTTT
GTTGGGGTATTCTTCTGTGAACTCTACAATTAGTTTAAATGTACCATCCTCAAAGGGTGTTCCTTCAGGCCCAAATAT
GACTGCATTCCACACCATGATGTTGTTTTCAGATGGGGCACCACTAACACCAGCTGGAGGATCCTCTTGTAGCCGTTT
AAAATCTCTCATAAGCCTCCTTCTGGCGGGAGTTGACATGGTTAAACTTGTGAAACACGTACTAACAACACCGATACG
TTTACAGGG

>Sequ28258EST2
CACACACACACACAGTGACACACACTCACTCTGTGGTCCTGTGTGTGTGTTTCAGAGCGACCTGTCTCTCTCTCTGCC

TGTCTGTTTGACTACAGGTAAACGATGTCATCAATCACATGATCTAACATGATGAGGTCTGACATCATCAACCACATG
ACGTCCGACAGCTTTGAATCAGGTAGTTCAAGATGTCTATCAGCTCCTCTGACCTCTGATAAAATCAATGATTAAAGT
TGATCAGCTGTCAGTTGATTGGTCAGTGCTGTGAGCTGGATGTTAGTCGGGCTCCACACTCAGGATGAACTGAAATAA
ACTGTGTGTTATGGTTCAGTGTCCGACCTCTGACCTTTGACTCAGTATCACACATCCGGCCTGAATCACAGGAGCTGT
TACCTGCTTGTCACTGAACATCACATCTGACTCCTCATCAGTCAACCAACCTACGTTTCCATGGAGACAGAAAATGGC
TGGTAGCTAGACGCCCTTAACTTCATCCTCACTTGTCTCACCTATCTCACACCTGTCCCCTCTCTTCCTCCTCTTATC
TGCCTACAATCAGCTGGAACAGGAAGTGCCAGAGGACCTACACCTGCTGTGAAGCCCACCCCCTCGTCTCCTCTGGTC
AGCTGTCGAGGACCCTCCTCTTCACTCTCACCTGTTTTAAACTAGTCTGACCTCTGAAACACACACACCTACCTGTCT
CTCTCTCTAACACACTCTCTCTCTCTCTCATCCAGCTGCAGCTCGTCATCTCTACTCGTCGTCGTCCTCCTCTTCATC
TTCGTCCTCTCCCTCGTCGTCCTCCTCTTCCTCTCCTCCCTGAGCTGAAGAGCCGGGGTTCCTGGCATATGTGCCGCC
GCCGCGGTACGCCACCATGTCCCGGTCGTATTTCTCTCGTAGCTTCTGGGCCTTCTCTTCGTACGGCTGTTTTTCAGA
CTGGGACAGTTTGCTCCACATCTCTCCCAGTTTCTTGGCACAGTCTCCTATAGACAGACCAGGATACTGCTGCTTCAC
ACTGGGACGGTACTCGCTGCAGAACACGAAGAACGCAGACGGGGGTCTTTTGGGTGCGTTGGGGTCTTTCCTCTTGCG
GCCCCTCTTTCCGAAGCCCTTTGGTGGGACGTAGTCTCTCATCTCCCTGTTGTAACGCACCTTGTCGGCCTTGGCCAT
GTCCTCGAAACACTTCTTATCACTGGGAGACAGAGCCTTCCATCTCTCAGAGCATTTCTTGGAGAACTCCGCGAAGTT
GACCGACTGCTCAGGATTTTTCTTCGGTGCTCCTCTCGACACGTCTGGACGAAGAAGGCGTACGCCGACGTCTTCCCC
TTCGGTTTGTTGACGTCTTTACGCATCATAATGGCGGCTCGTCAGGAGGGCCGGCTACAGGCGGAGGCAGACTTCGAA
CCGGTTTTCGGGATTTGGGTCCTGGGGTTCTTGTTGTTGTTTGGAGGCTGAAGCAGCGACTGGAGCCC

>Sequ28315SNP2
AAAAGAAAAAAAACACAACAAGACTGACGGGTTGATTGACAAAAATTACTAAAGTTAAATCATCGAGGGAATTTTACT
GTCGATTGTGCCTGCGAGGCTGAATAATGTGTGGAAAATGAGATGAAAGGTTCTTTTCACATTAGACAGAAAAGGCAC
TTTTTGAAATAAAATAAAGAGGTCTAAATAAACAAAACACAGACTTGTTGGAGGTAGGATGACATGGATGGAAGGGCA
AGATGAGAAACAATACATAAATATTTTGGAAGAAGAAAAGAAGCTGGAAGTGTGTTTCTGAACTCCTCAAACGCTCAT
TACCATCCGGACGAGTTCTTCATAGTTAATGCAGCCATTGGCATCTTCATGTCCTGCTAAAAGAGTCTCCACCTCCTC
CTCTGTCATTTTTTCACCTAGTGTAGTGAGGACATGACGTAGCTCTGCTCCCATCACCGTACCATTTCCCTCCTTGTC
AAAGACTCGCAGGCCCTCTACAATGTCCTCAAAGGAGCCCTGGTCCTTGTTCTTAGTAATGGCCTGAAGCATGGGCAG
GAACTGTTCAAAATCCAGCAACTTGTGGTTCATCTCTTCTAATTTGGGGTTTCCCAGGACTTTGAGCACCTCAGCATT
GACAGGGTTCTGTCCAACGGCCCGCATAACATCACCACACTGGCTGTAGGTTATCTTCCCCTCACCCGTCCGGTCAAA
CAGAAGGAAGGCTTCCTTGTACTCCATGATCTGGTCCTCAGTGAAGTCAGACATGTCTGTTTTTGGATATAAGACACA
GACAGGAGGGAGAAGGTCTTGTATCTTAAGCTGTTAAGAC

>Sequ28317EST2
TTTCTAGACAATATAGGTTTATTTTTACATTATTTTGCTTCCACAATGACAAAATGTCAGCTGTGATGTCAGACTGAA
ACAAGTGCAACAGATAAACTGATGGTCTGATCAAAGACATCATCATCCGGTGTGACGAGCTGTGTTAACTTTAACATA
CAGCACCGGCAGAATTAAAAAACAACGCTTGATTCTTCAGACAACAGAAAACCCAGAATAAAGTCCAACACTGAGAGT
CAGCAGCAGGTTGAAGATGGTGGTGGGGAGTGAGTGGGTGGTTAGTGGGTTCAGCTGGCAGCAGGGGGGTTGTCTTGC
TGCAGAGTCGACCCAGCAGACCGGCCATCTGCAGCAGGGAGTTCACGCCTTCAGCCACCCGCATGTGAGTGTAACCGA
TCTCCTTGATGAACTCCAGTTTGAGGTACTCGGCCATCTGGTAGGTCTTGCAGACCCTGAAGATGTTTCCGATAATGT
CCTCTGGAGAGTAGCCCAGCGCCCACAGCTGCTCCACCACCTTGTAGGCCTGGTCGATGTTCCCGTCCACACAGTGTC
CCAGCATGCTTTTCACCATCAGAGGGTGAGGCTCGTCGCACACCTTGAAGACATTCTCGCTGTTGATGTAGCCGAAGC
CAGAGTTTGTCGACTGCAGGTTGTTCAACGCCTGTCTCATGTCTCCCTGTGCGGTGAAAATGACTGCCTCCAGCCCGT
CGTCAGACACAGACAGACGCTCCTTCTCCACCACCTCCTGCAGCCGGGAGAGGATCTGTCCGTCCGTCAACTTGGAGT
AACGCAGCACGGCGCAGCGGGACTGGATTGGCTCGATGATCTTATCTGAGGCGTTGCAGGCGAGAGCGAAGCGCGTCG
TCTTTGAGTAGATCTCCATGATCCTCCTCAAAGCCTGCTGAGCTCCATCCGTCATGCTGTCAGCTTCATCCAAGATGA
TGATCTTGTGTCGTCCTTTGGGCAGTGTGACTTTCTGCTTGCGCAAACATCTTGATCTTGTTCCTCACCACGTCAATT
CCTCTGTCATTTGAAGCGTTGAGCTCCAGCACGGCGTCCTTCATGGAGGCACCCAGCAGCGCTCGGGCCAAACACAAG
ATGCTGGTGGTCTTTCCTGTGCCGGGAGGACCTGCGATGATGATGTTGGGGACGTTTCCCTCTCTTGCGAACACCTCC
AGCCGGCTCACTGTCTCCTCGTTCCCTACGATCTCGCTCAGCTTCAGTGGACGGTATTTCTCCACCCAGGGCAGCTCG
TAGGCGCCGCCGGAGCTTTTAGACGAACTCTCCCCAGCCTCCGTGCCCGTCTTTGGCCCGCTTTCTGTCGGTTTCTGC
ACACGCTCAGGCTCCGCCATCTCCACGTCCATGTTGATGGCTGTCTCTACTCTGTCA

>Sequ28339SNP2
GACAGGAGGAGTACAGCCGAAGTTGAGTTGAGGCAGACGGGAAAATCATTCAATCCGCTGAATCAACCAGAGGCAACA
TCCACCGGCTGAACACAGGAAACCGTTTACATCATGGAGCAGAAACAAACCATCAACTTTGGCGCTGGACCTGCAAAA
CTCCCCCAGTCCGTGCTGCTACAAGCACAGAAGGAGCTCCTCAACTACAGTGGCGTTGGCATTAGTGTTCTTGAAATG
AGTCACCGATCATCAGACTTCAACAAAATCCTCAACAAAACAGAGAGTCTCCTGCGAGAGTTGTTAAATATCCCAGAC
AACTATAAGGTGATGTTTCTGCAAGGTGGCGGCTCTGGACAGTTCAGCGCCGTTCCTCTCAACCTGATTGGCCTCAAA
GAGGACAAGTGCGCCGATTACCTGGTGACTGGCACGTGGTCGTCAAAAGCGGCAAAAGAAGCAGAGAAATATGGCAAA
GTCAACATTGTCCACCCGAAGCTGGATAGTTACACAAAAATCCCTGACTCCAGCAGCTGGACCCTGAACCCCTCAGCC
TCCTACGTGTACTACTGCTGCAACGAGACAGTCCACGGCGTGGAATACAACTTCACACCTGAAACAAACGGGGTGGTC
CTCGTCAGTGACATGTCCTCCAACTTCCTGTCCCGACCTGTGGATGTGTCGAAGTTTGGGCTGATTTTCGCCGGAGCT
CAGAAGAACGTGGGCTGTGCCGGGGTAACTGTGGTCATTGTGCGAGAGGACTTGTTAGGCCACGCTCTGAAAGAGTGT
CCCATTGTCCTGGACTACAAGGTGCAGGCAGAGAATAACTCCCTCTACAACACACCGCCATGTTTCAGTATCTACATC
ATGGGTCTGGTGCTGGAGTGGATTAAGAACAACGGTGGCAGCGCTGCCATGGAAACGCTCAACAAGCAGAAATCCTCC
ATGATTTATGACATCATCAATGCTGCTAATGGTTTCTATTCGTGTCCTGTGGATCAGGGTTGTCGAAGCCGCATGAAT
GTAACATTTCGCGTGGGGAAGAAAGAGGGAGATGACGCCTTGGAAAAGGAGTTTCTGGACGGAGCATCTAAACGTGGA
ATGATATCACTGAAAGGACACAGGTCAGTTGGGGGAATTCGTGCATCTCTGTACAACGCTGTAACACTGGAGGACACT
GAAGCCCTGGCTGCCTATATGAAAGAATTCCTCAAAGAGCACCAATAAAACAGGCCTAATGTCACACAGTGGCATGTC
ATTACTTGGTGCAGTTTAAAAAAGTTTACCTCTAGTTGAGATCCATGTTGGATCTGGAAGGTGTGCCTTTAGACACCT

GTTTTATAACACTCATCCATAGTTCCTAATAAGCTTTAACCACTGCTGTTCCCAGTAATGTGTTAATTACTTCTGTGT
TTCATAGAATGACTATTTAAATGGATTTATTGACTAACAGTGCTTTGATATACATACTGTAGGTAATTCTTGTTACTT
GTTAAAAATGTATTTACATTCCAGTAAATCTAACTCAGAAATTACCCAAAAAAAAAAAA

>Sequ28348EST2
GGTGGATACGAAGAAGAAAATAAGAGAGAAGCGAAACATTTCTCACGACTTCTGCTCACTTTTCCCCTAGGTGATATT
TAGTAGCCATGGCCCGTACCAAGCAGACTGCCCGTAAGTCCACTGGAGGCAAAGCCCCACGTAAGCAGCTGGCCACAA
AGGCTGCCCGCAAGAGCGCCCCTTCCACTGGCGGTGTGAAGAAGCCCCATCGCTACAGGCCTGGTACCGTGGCTCTGC
GTGAGATCCGTCGTTATCAGAAATCCACTGAGCTGCTGATCCGCAAGCTGCCCTTCCAGCGCCTGGTGAGAGAAATCG
CCCAGGACTTCAAGACCGATCTGCGTTTCCAAAGTGCAGCCATCGGAGCTCTGCAGGAGGCCAGTGAGGCCTATTTGG
TGGGTCTGTTTGAGGACACCAACTTGTGCGCCATCCACGCCAAACGTGTCACCATCATGCCCAAAGACATCCAGCTGG
CACGTCGTATCCGCGGGGAGCGTGCTTAAATGCTCTCTCCTCCTCCTCTTTGTCTTTAATCTTTTCACCTCCTCCTGC
CCTTCCTTCACCAGTCTGTCTCTCCGTCCCTCTGCCTTCCACCTGCCCCCTTCCTCCCTCTCTTCCTGTATTAGTAGT
GTGTAGATGATAACTGATTATATTATGAATAAATAAAGTGTATAGTCGTGTTTTTCTTAGTGCTCTCGGCAGCAGACT
TCTAGGGTACTCTCTTTTTGTGCATGTACCAACTCTTTGGTGATGAGATCCCCTCAAGCACACATCTCCTATGTTGCA
CTGTTTCTACTCTGACTCTGTGGAGTAAAGGATGATTTATGGATAGAAAGAGGAAAAGCAATGGACTAGAACAATGCA
AATTTGGGAGGGACAGACAAGAGGAGAGGATGGGTATTGTCTGCGGGGAGTCTCTTTTCTGCAGCAAGGTGCTAGAAG
AACTTACCAGTCCTCTTCTCCTGCTTCCTCTGCTCTGCCAGCCTCCGCTCTGAAATGTGCAGAAACAAGAGCGTTAGT
CTCTGCTCATGAAGGCCGAGGTTGGAATTTCTGTCCCTGCATGTGTCTGCATGAGTGCTGGCTCTGATCACCACAGCA
AATTGTATACAATCCATCTGCATCCTTAGAGAAGTTGATTTATGATTATGCTGTCATTTGTCATGTTAGCTTTTTTTC
CCTTCAAATTAAGTCTCATGCACAGGGGTTTTTTTTTTTTTTTTTCACTTCAAACT

>Sequ28352SNP2
ATTTTATTAAGAGTTGCTGTATATTGGGTAAACTTTAAATAAAGATTTGTTTTAGCATTTGTAAGTGTCCTTGATTTT
TAGATTTAATTCACATATATGTATCAGCTAAACCTGGATAAAACTAATGCAGTATATATATAATGTTCAGTATTTACT
GAATCTGTTTGAGAGGTGTTAGTTTATACACAGTGGTGTCAAAGTGTGAGACACTTTCCCATGAAGCCTCACATGTTG
CTCCACAGCAGCTGTAGTGTCGCTCAGTTTCATTTACACAGGAGAATAAATAATGGATTTAATAATGTGTGATACAAA
CACACTTACACTATAAGAACAGCTTTTATTGTGAGTCTGAATCCAGTGATGTTTGAACAGAGACTTCGCAAACGAGTT
TATTTCGTCACTTCCTGTCTGTCAGCTGATTGGTTCTCAGCTTCATACCAATGTTTGCACCTGAACTTTGTGATTAAC
ATGAAGGACGGTGAAGAAAGTCAGAGAAGCTCCACGTAATTGGCGGGAAACATGCCGTAGTGTCCGTCAGGTCCGTAA
CCCCTCCACCAGCCCTCGTCCAACATGTCTATCCCGGTGATGATGTCATCGGGGTCAAAGGTGATCTCAGTGTCGTCC
GCAGCCTGGTAGTCATATATCGCTCTGGCACAGATGTTCTCTCCGTTGGTTTCTGCCTCCTGTTCTTCAGCTGTGTCC
TGTTGCTCGTGAACGTTTTCATACAGGTCGTCCTCTGCTGCCGCCGGCTGCTCCGCTCTGTACAGGATCCCCGTGAGA
GCGATACTCTGCTGTCGTCACTTCCTCTGCGTCGTCATCAAACTCGTCAGACCACTCGTCCTCGTCTGCAGGTTGAAC
AGGTGAGCCTGTGGTCTCTGGGGGGGCACAGCTGCAGCAGGTGAGCGAGACAGCACAGGTGAGACAGGTGTGACAGGA
GATGGGGGGGTCACAGCTGCAGCAGGTGGATGCTGAGGCTGCACCGGGGTTTCTCTGTCGAAGGCGTTCTGAGATACA
AACGGACTCCGCAGCCTCCCTGGTCTGGGCGCAGCAGGAGACGCTCTGCCGCCGGCCTCAACGCTCTGCTCCCTCTGC
TTGAAGATGTCTCTGGGGTTAAACGATCTCTGAGAGATCAGTGATCTGGCTTCGTTAGCTTTCTGCACCGACGCTGCA
GAGTTGATGCCCGACTTCCTGGTTTCTTTTTCCGGCTGGTTCTGTCTCTGCTGCTCCCTCTCTCGTTCTTCTTCTTCT
CTCTGCTTCTGATGAATCCTCTCCTGTTCCATCTGCTGCGCTCTCTCCCTCTCCTTCCTCTCCCTCTCCTTCCTCTGC
CTCTCGTCCAGCTCCCTCCTCTCCCCCTCCAGCCTCTGTCTCTCCCGCTCTGCTCGCGTCTGCTCCTCCTGTCGACGA
ACCCGTTCGTCCCTCTGAGTTTGGTCCCAGAAGTCGTCCTTCCTGGTTTGTTGAATCTCCTCCACAGCGTTGACTTTG
CGATAAACTGAACCCACGGGTCCTCGCGGGGCGTCTCTGTACTCTGTCTGTTTGAAGAAATTAAAGTTTGCTCCAGAC
GCTTTGGACACTTTGGTCAGGATGGTCTCCGGCTCCACGTCCTCCTCCCCCGGGCGTTTATGGTCACATGGGCTCCCT
TCAGGAAACTGGCCATGGAGCTCACATGGTTCGCACACATTCCTTTACGAGAGTCCTTCACTCCTTCACCCGTCCAGT
TTATGAGGACAAATTTGGACAGACCAGAGTTTGGGTCCGGGACACGACAGAAAGCGTACATCACCTTCCCACTGCTGA
GCTCCTCCACCATCTCCTCCAATCCACCATCAGTTTGTGTCCGACTTGCCGTCCACCACCTCTTTGTAGGCGGCCATG
AGCGCAGGACCGTTCTTACTCAGGTTCACGGCCATGTTAGTCCAGGTTTCCTCACTCACAGTCTACACACACACACAC
CACACACACACACACACAGGAGAGAGAACCCTGAGCAGAAGAAGAGAAGAAAGTGCAGGTAGGGTCTGTCAGAGGAGA
GTCCTCGTCTCAGACGCC

>Sequ28354EST2
AGTATTTACGTCAGTCCCACGCTCAGCCTCTTCATAACACTGAGCGACGATCAGGTCCAGCTGGGTGGACTCTGGACT
CTGAGGCCCTCCTGTGGACCGTGTCAGCCTGTGATGACCGTGGGTAGACGTACACATGACTACACCTGAGGGGTTGAT
GGAGACCGTGAACTCTGTGGTGACATTCAGCCCCGTCACCGTGGAGCCAGTGAACACGTCGTAGGCCTCATAGTTTCC
GGCTTCATAGTTGAGTTTATCCAGAGAGCAGTGGTAGAGGAACGGCATGTCTGTCCTCAGGCTCAGGAACACAAGGCT
GCTGGCTGAGTGAGACAGAGGCCTCCAGTACACCTGAACACGACTCTTCTCCTGCAGCAGGCGTCGTCCCTGGATACC
CAGGGGGTCCTGGTTGATGGCGATGGCCACTTTGTTCTGCAGGATGGCCCTCGCACTGTTGTCCAGATTTCGCAGGTC
ATTAGACATGATCAGAGGCGCCGCCATGATGGCCCACAGAGCCATCTGAGAGCGAGCCTGGTCCACGCTGAGGCCGAA
GTTACCGATGATCAGCATGTCAGGGTCATTCCACCGCCGGGTCCTGCTGCCGGCTGCAGATCATCCTGGTTGTTGGAG
AACCAGTTGACGATTCCCTGCACACTGTCCCAGGAGTCCTGGATATCATCGTAGTTCCTCCACAGGTGACAGATCTCC
CCCAGCAGAGAGTAGTTCACACTAGGGGGAAGTCCTCCCATGTAGACGGGCCAGCTGCAGGAGTAGACCATTGGTCTG
CCTGTAGCATTCAAAGCCTTTGACATCAGAGGATAACCCAGCATCTGTTCCACAGGATTAGAGTAACAGCCGTCAAAC
TTCAGGTAGTCACCCCCCAACTGGCAAAGGTTTGGGCATCAATCTCAATCTTGTCCAGTGTGGTCCCAGGGTATCCCA
TGCATGTGAGATAGCCCATGTCTGCATAGATGCCCAGCTTCAGTCCACGGTCGTGGACGTACCTGGCCAGTTTTGCAA
TGCCCCCTGGGAACCTGGAGGGTTCAGGCTGCAGTCTCCCCCCTTTGTCTCTCAGCCTGGACATCCAGCAGTCGTCTA
TGATCACATAATCATAACCCAGCTCCTTCCAGCCATCCTCAGCCAGCCGGTCAGCCATGTCTCTGAACAGACCCTCAC
TGATGCAGTTCTCTGGGTCGTCCTTACAGTCC

>Sequ28364EST2

CAAGAACATCAAGCACAGTGGAAGTGTGACCTTCGATGAGATTGTCGGCATCGCTCGTGTCATGAGGCCTCGCTCCAT
CGCCAGGGAGCTCTCTGGAACCATCAAGGAGATCCTGGGCACCGCTCAGTCTGTTGGATGCACCATTGATGGCCGTCT
TCCCCATGATGTCATCGATGACATCAACAGCGGCAAAGTCGAGTGCCCATCTGATTAAATGGAGTCCAAAACAGTAAC
GGTGTGATCACCACTCCCACCCCCGGTCAGTACTTCTACAACCGGGGCATCACCGATGAGCAGGAGGGGTTCGCAGAG
GGGTTTGTAAAAGCCCTGGACGAGCTGCACAAGATGAACCAGATGCCCCCTCCTAACGTGTCCATCGGTGCCGGTGGA
GTTACGACGTGTTCAGCGGCGGCCTCTAGTGTCTTCGGCTCCGCCTTGCAGCCGGAGCCTCCTATCTACACAACACTG
AACGCCTACTGCCCGAACACGAGCCTCTCTTCCGCATCCAGCTACCCTACAGCCACCATCAGCTACCTGCCGCCACAC
CAGCAGAGCCACCCGCAGACCTCGACGCACGGCACGCACCCGTTCCAGCACTCGCTCCCTGCCTCCGGACTCCATCCG
CAGCGGCTCGTCGCTCTGAAAGAGGAACCTCAGACGGTGCCCGACCTCCTGAGCAGCGACGGCTCGCCTCCGATGTCT
CCCATCGACTTGGAGACCCAGGAGAGGATCAAGGCGGAGCGCAAGAGGCTGAGGAACCGACTAGCGGCCACCAAATGC
CGGAGGCGCAAGCTGGAGCGCATCGCCCGGCTGGAGGAGAAGGTGAAAGTTTTGAAGAACGACAACGCGGGGCTCTCC
AACACAGCGTCGGTGCTCCGGGACCAGGTCGCCCAGCTCAAACAGAAGGTCCTGACACATGTGAGCAGCGGCCGTCAG
CTGATGCTTACAAGCAAGATGGAGGCGTTTTAAACAACAGAGACTTATTAACTGACTGAAAAGTAATAACACTGGAGT
CATGTTCTGCATGCGCAGCACTAGAATACCGGCTGATGCATTGTCAGCACTGGCAGCATCACAGGGCCGGGGGCTATG
TGTAGACAACTCTATAGGCTATTTAGGGTGAAATGACTGATTGAAATGGTACTTCCGGATGCTGGACACCGCTCAAAG
GACCATTCAAACCCAGAAACCAGGGCAGCATCCAGACTGAGAAAACACAATGTCGGCCACTGGCCCATGAGTCGGGTT
TACTAATGGTAGCCTAGAAAATGGGAATGATTATTTGTACTTTTGCAACTGATATTGTCATGCAACATGCTACATTTA
TTCCTTGTGTAAATTATTTTTGTTTGTCCGGTTGATCCAGACAAACGTATGATGTCCAATGTTTACAATGTCTTTTTT
ATTTGTTATGTGTATTTAAGTAAAGTGTCTTCACATG

>Sequ28372EST2
GGAGGAATGAACAACAATAGACACCTTCACAGCGACCACTTATCTGGAGACGCTTTTCAGCTTTTAATTTTTTCTACA
CTTAACATAAGGTCTTGGGGTCAAGAAAATGGCCACCTCAGCAAGTTCAAAACTGAACAAAGCTGTAAAGCAGCAGTA
CATGGACCTCCCTCAGGGGGACAAGGTCCAAGCCATGTACATTTGGATAGATGGCTCTGGAGAGGGACTACGCTGCAA
GACCAGAACTTTGGATTCTGAACCGAAGACGATTGAGGATCTTCCTGAGTGGAACTTTGACGGCTCCAGCACCTACCA
ATCGGAAGGCTCCAACAGCGACATGTTCCTGGTCCCTTCAGCTATGTTCAGGGACCCGTTCAGGAAGGACCCCAACAA
GCTGGTGCTCTGTGAAGTGCTCAAGTACAACGGGAAGCCAGCAGAAACCAACCTGCGTCACACCTGCAAACAGATCAT
GAGGATGGTGGAGAATAACCACCCATGGTTCGGTATGGAGCAGGAGTACACCCTCCTGGGCACAGATGGACATCCCTT
CGGCTGGCCCTCAACGGCTTCCCAGGTCCACAAGGGCCGTATTACTGTGGAGTGGGAGCAGATAAGGCATACGGACGA
GACGTAGTGGAGGCGCACTACAGAGCCTGCCTGTATGCTGGAGTTCAGATCTGTGGAACCAATGCTGAAGTCATGCCA
GCTCAGTGGGAGTTCCAGGTCGGGCCGTGTGAAGGTATCAACATGGGGGACCATCTGTGGGTCGCCCGCTTCATCCTT
CACAGGGTGTGTGAGGATTTCGGCGTGGTGGTGTCTTTTGACCCCAAGCCTATCACAGGGAACTGGAATGGCGCCGGC
TGCCACACCAACTTCAGCACGAAGGAAATGCGAGAGGATGGTGGACTGAAGGTCATCGAAGAGTCGATCGAAAGGCTG
TCAAAGAGACACAGGTATCACATCGGGGCCTACGATCCCAAAGGCGGGCTGGACAACGCCAGACGCCTCACTGGTCAT
CACGAAACCTCAAACATCGAGGAGTTCTCAGCGGGCGTGGCCAATCGGGGCGCCAGCATCCGCATCCCTCGCTCTGTG
GGGCAGGACAAGAAGGGCTACTTCGAGGACCGCCGTCCGTCCGCCAACTGCGACCCTTACATCGTCACGGAGGCTCTG
GTGCGCACGTGTTTACTGAAAGAAGAAGGAGAAGAGCCCACAGATTACAGCAAATGAACAATGCGAAACAAACAGTCA
TTTCCAGTACTGTATTTAGCCTCGTGTCTAGTTCAGGAGCATGGATTTAATTTTTTTATTATTTTTTTACTTGACGGA
TGAAACAATGAGGTGTTCCTGCATTCTCTGTGGATTCAGTTGTATGCAGAAGTTCTTTGATAACATAAACACTGCCTT
TCTGTCAGCAAGGCGTTCTGTCACGGTAGCTGATCATTTGTATAGAAGTACATTATTGTAATGATTTTAACTTGTACT
AATAAACTGTCCAAGTGCAGCAGGAGGTTGCTGTAGATCTTGTACCTCTGACACAACAACGATCCCAAAGGCATCTGA
ATGTTTTTAGCAGTCCTTGGCCTCTGTCATGTGTAGATAGTTAAGGTCATTGTGTAAGCCAATCTACTTTTCTATGTT
TTTGTTTTTTTAATTAGCATGTCTTGTTTTGTTTTTTTCACTTGTAAATGTGACTTGCAAGAACTCTTTAATAAACAG
CTAAACAAAAATTTTTTAAAAAAAAAANAAAAAAACCGAAAAAAACCTACCTAACTAAGTAGTAAGTACGTAGG

>Sequ28393EST2
GAAGAGCGGGAACGCAACAACACGAGCTCAAACCCACCTGTACTTACCTGAAAGCTACTACACAAAGATCAGCATGGC
AAACAAAGGTCCTGCCTATGGTCTGAGCCGTGAGGTTCAGAGCAAGATTGATAAGAAATACGACCCGGAACTGGAGGA
AAGGCTGGTGGACTGGATCGTGCCCAGTGTGAGCCTGGTGTGGGCCGACCCGAGCCAGGAAAGACTGGCTTCCAAAAC
TGGCTCAAGGATGGATGTGTGCTGTGTGAACTCATCAACAGCCTGTGCGGAGCCAACAAGCTGGTCAAGACCATCAAG
ACCTCCGGCATGGCATTTAAGCAGATGGAGCAAATCTCCATGTTCCTCAAAGCTGCAGAAAGCTATGGAGTCACCAAA
ACTGACATGTTCCAGACTGTAGATCTCTTTGAAGGCAAAGACCTGGCTGCCGTCCAGAGGACCCTGATGGCTCTGGGT
AGTTTGGCAGTCACAAAGGATGATGGGAATTACAAAGGAGACCCCAACTGGTTCCATAAGAAAGCCCAAGAGAACAGG
AGAGACTTCTCAGAGGAACAGCTGAGCGAAGGCAGAAATGTCATCGGCCTGCAAATGGGCACAAATAAAGGAGTATCT
CAAGCCGGCATGACAGGTTACGGAAGACCCAGGCAGATTATCAACAACCCCTGAGGCCAAGCAGTGCTTAAACGCTCC
TCCAATACCCTCCTCCTTCTGTCCTCTGAAAGGCCAGTCAGCGAGGAAGGAGAAACTCTTACTCTTACCTTGGCCATT
AGGTGGAATGTGACCTGACTGCTCTCAAGACAAAACTGGACCAAAACAGGGACCAACAGGAACATGTGCACATACACA
ACACACACTCCATGTTGAGGAAAACACACAGAGATAAAGACTTTTAAGTGATTACACAATTAGACAGAGAATTTTACA
TAGTATTTTTTACAGCAACACACTTCTAGGCCACCTTGTAAATAATACTTTTTCATAAAGAATCATTCCAGATGTTTT
GCATGTCTTAACTCACTACCTTAAAATCAGATGTACTTTTTATTTCAACCATAAGAGTTTAGATTTCTTACCTTCCAG
ACATCCACTGGTTCCAGTTCACTTCAGTACACTATAAAAATCAACAGGTAGTGACTTGTAACTCGCTTGAGAGAAGTA
ATCCATCACATTCAACATTAAGTTTCCTACATTAATGCAACATGGTAATGCATTCAGGAACTCTCTTCATAACTCTTC
CTGCATTCAAGGGCACTCTGACATCTGAGATTTTAGACTTTTTAATGGAAGAAACAACCAAATCCATATTTATTTTCA
TGGTGCCCCTGTTTGTGTTTCGTCAATCTCCCAGTTTTGCTGTTCTGACAGAGTAGCTGTCAATTCTTGAACGTCAGG
ATGTCCTTGAATGCACCACGGAGGACAGTTTTGAAAAAGTTCCCTGCTCATATCTGCAGAACAACAGCATAGCATAAC
TGTAATGTTGCGTTAACAAACATGAATGATTAAGAGTAAACTGAGCAGAGTGTTCAGTGTGATAGATTACCTGTCTCA
AACAACTTTCAAGTCTGTGCTGATTCATTGATTACTGGAATGAACTGAAACAAGTGGCAGCCTGGAAGGTAAGAAGTG
TCTGGAGTGCTGTGGTTTACAATAATGTTAAGTACATAATTTGAAGTATACATAGATCCACCTGTATAATTTAGAAAG
TGAATTGCCTAAAAAGTTTCATGAAAAACATAAAATACAGTTGTGCGTGTTTTACCTGCTTCATAACACAGTCTGTTA
ACTAATTTCTAATCGATGTTGGACCTTCTTTTTAGAGTTGAATGGACGATGTTGAAATAAAGTTTGATTTAACTGAAC

AAAAAAAAAAAAACCG

>Sequ28408EST2, Sequ28408SNP2
AACGTTTCGAGGTCGGCTCACGTTTTTACTCTGGAAGTGTTGGCTCTCGGTCTTTAAGATGTACGGATCCTTTCGGGT
GCTGCTGCAGGTGCTGTGGGCCCAGCTGCTCTGTGGGTGGTGCTGGGCTCAGAGCAACTTTTTGAGCTGCCAGACAAC
GCCAAGCAGTGTTTCTACGAGGACATCATCATCGGCACTAAGTGCACACTGGAGTTTCAGGTGGTGACCGGTGGCCAT
TATGATGTGGACTGTCGTTTGGAGGACCCAGATGGAAATACACTCTACAAGGAGATGAAGAAGCAATATGACAGCTTT
ACCTTCACAGCATCCAAGAATGGCACCTACAAGTTCTGCTTCAGTAATGAGTTCTCCACTTTCACGCACAAGACGGTT
TATTTTGACTTTCAGGTCGGTGATGATCCTCCACTCTTCCCCAATGAGAACAGAGTCACTGCTCTCACCCAGATGGAA
TCAGCTTGTGTGTCCATCCATGAGGCCCTGAAATCAGTCATCGACTACCAGACACATTTCCGCCTCCGTGAGGCTCAG
GGACGCAGTCGGGCGGAGGACCTCAACACCCGTGTTGCGTTCTGGTCCATTGGAGAAGCCATTATCCTTCTGGTGGTC
AGCATTAGCCAAGTGGTCCTGCTGAGAAGCTTCTTTTCTGACAAGAAAACCACTACAACACGTGTCGGATCATAACAG
TCTCTGAGCTCTAGGATCTGATTCATCACTTTCTTTATTTTTACCTGGGCTTGTGTGCATAGAGTCGTTTAGAACAGC
AGTGCAGATCTGAAATTCGATCATATCAAACTGCCAGGTCATACAATCTGACATGTCATCCCCATTCAGCACTGCTGT
TCTTATAAACCTTATGCAACTGAGTCTTGAGCAAAAAGGTTGTGTGTGTATAAAATGTTTTTGGGGGGTTAAAGAAAA
AACCTGAAGAAAGGCTTGATGGTGTTGCTTGGAACTATCTGTGTTTGAAGCAGTTCAAGCAGCTGGTCATATAGTTAA
AGCAAACCCTATGTACTTTGAATATTTGAAATAATTTCTGAATGTGTTCTTATTTTATTTGTGTCCTCGGGCCCCATC
ATCCCATACCCCTCTCTCCAAGATCTGATGCATTTTTTGGCCCCGATTGTGCACTGAAATAACCAAGGGAGTGGAAGT
TGTAACTAAATGTACTCATAATATAAGGATGGTCTGGTATTTTCTTCCTTATCTGCAGAGCATTTGGTCAAAAAAAAC
ACACTGGCAAGGCAAAGATTGCATTTGGTTATGAAAAAACATCTGTTTTTTTTTTTT

>Sequ28411EST2
ACATGGGACCTTCAGCCATTTTGACCATGTTAGGAGCTGTGGGACGCTGCTGCACCGGGGCTCTGCAGGCTCTCAAGC
CTGGGGTCCAGCCCCTGAAGGCTCTTGTTGGATCCCCAGCCGTCCTTTCACGCAGAGACTATGTCGCACCTGCCGCCG
CTGCCAGCGTCGCCAACGGGCGTATTGTGGCTGTCATCGGTGCCGTCGTCGACGTCCAGTTCGACGAGGGCCTTCCTC
CCATCCTCAACGCTCTGGAAGTCTCCGGCCGCGAGTCCAGGCTAGTCCTGGAGGTGGCACAGCATCTTGGGGAGAACA
CAGTGCGTACCATTGCTATGGATGGTACTGAAGGTCTGGTCCGTGGACAGAAAGTGCTGGACACCGGTGCCCCCATCA
GAATCCCAGTGGGTCCCGAAACCCTGGGCAGGATTATGAATGTCATTGGTGAGCCCATCGACGAGAGGGGTCCCATCA
CCACCAAACAGACTGCACCCATCCACGCTGAGGCCCCTGAATTCACTGACATGAGTGTGGAGCAGGAGATTCTGGTCA
CTGGCATTAAGGTTGTGGACCTGCTGGCCCCCTACGCCAAGGGAGGCAAGATTGGTCTGTTCGGTGGTGCTGGTGTCG
GCAAGACTGTATTGATCATGGAGCTGATCAACAACGTGGCTAAGGCCCATGGTGGTTACTCTGTGTTTGCCGGTGTGG
GAGAGCGTACCCGTGAGGGAAATGACTTGTACCATGAAATGATTGAGTCTGGTGTCATCAACCTGAAGGACACCACCT
CAAAGGTGGCGCTGGTGTACGGACAGATGAACGAGCCCCCCGGTGCCCGTGCCAGAGTGGCTCTGACTGGACTGACAG
TGGCTGAGTACTTCCGTGACCAGGAGGGTCAGGATGTGCTGCTTTTCATCGACAACATCTTCCGCTTCACACAGGCTG
GCTCTGAGGTGTCTGCCCTGCTGGGTCGTATCCCCTCTGCTGTGGGTTATCAGCCCACTCTGGCCACTGACATGGGTA
CCATGCAGGAGAGAATCACCACCACCAAGAAGGGCTCAATCACATCTGTGCAGGCCATCTATGTGCCCGCTGATGATT
TGACTGACCCCGCCCCCGCCACCACCTTCGCTCACTTGGATGCCACCACTGTGTTGTCCCGTGCCATCGCTGAGCTGG
GTATCTACCCTGCTGTCGACCCCTTGGACTCAACCTCCCGTATCATGGACCCCAACATCGTCGGATCCGAGCACTACG
ACGTCGCCCGTGGTGTGCAGAAAATCCTCCAGGACTACAAATCTCTGCAGGATATCATTGCCATCCTGGGTATGGATG
AGTTGTCTGAGGAGGACAAGCTGACTGTGGCCCGCGCCCGCAAGATCCAGCGTTTCCTGTCCCAGCCCTTCCAGGTGG
CCGAGGTTTTCACTGGCCACATGGGAAAGCTGGTGCCCCTCAAAGAGACCATCAAGGGCTTCAAGAGCATCCTGGGTG
GTGAGTACGATCCTCTGCCCGAGCAGGCTTTCTACATGGTCGGCCCCATCGAGGAAGTTGTTCAGAAGGCCGAGAAGC
TGGCTGAGGAGCACTCATAAACATCTAATCAATCTCAGTTCTGTGGAGAAGAAAATCAAAGAGAAGGGGGGGTCCTTT
GGTTAAGGAGCACTTGGTTTGTAAAACATGTCAAAAATGCAAATGTACCTGTCCTGTCATTCTGAGAGAAAGTTCTAT
TTAATGTTTCCAATGAAAGATGGAATAAAACACTGTCTTTACACAAAACCTGTCAACTGTATATTTCCTATTATAGGC
TTTCATATGTTGTAGTTGAAAATTTTGTTCCTAATGGGAAGTTTCATGAAATGGCTGTCATCTACTCTGACCACAAGA
TGGTGTTCTTGAGCCATGTAAAGCTACTTGAACTGTGTTGCAGCTTGTCACGTATTGAAATAAAACGGCGGACAAAAC
AAAAAAAAAAAAAAAAAACGAACGACTA

>Sequ28458EST2
GACGGCAACATGGCAGCCTGCTAACAAGAGCTGTGATTCCCTCCGCTCTGACGGTCAGACTCTTAAACTTCATCATGG
AGCCAGACACCGACAGCACGAGACCGGCTGGTCGGTAACGGAGGGCTCGGGAAGGTGATGACGATGATGATGTGTTTT
CGGAGGATTGCTAATGTTCGCTCTCTGGTGTCGCCGCGATCTGCGGGTGTGTGCTGGCTCGAGCCTGCGTCATGGCGG
CACAGACAAACACCTGTCACTGAGTGCGCGAGGCTAAAGCGGTCTCAGGTGAGCGGCTCGTGGCTCGTCAGACTCAAA
GGGACGGACAGCCGCGTCGGGTGCTGGAGTGGGATCCTCCCGCAGGTAAAACCGACAGGCTGGCACGAGCAAGGTACT
GTGTTTCTGTACCGGGGAGGTCACGAGAGGTCAGCGGCTAGCTTCCGGTCTCTGCTGTCACCCAATGCTCGTGGGCTT
TGCAGCGGAAAGACGAAGGAGACACCGGACAGCTCCCCGGCAGCGGACCGGCCTGAAGCCGGTGCGGTACCTGGACAA
GGACTGTTCAAGTTCAAAGAGCTGTATGAGAACCCATGGACAATCCCCAACGTGTTGTGTGTTTGCCGGATTTTGCTC
GCTCCATTCTTGGGTCACCTGATCATCCAGCAAGCACTTTCACCTCAGTCTGGCTCTGTTTGCACTTGCTGGCGCTAC
TGACCTGTTGGATGGTTACATTGCCAGAACGTGGCCAACCCAGAAGTCGGCGTTGGGCAGCGCTCTCGACCCCTTTGGC
TGACAAAATTCTCGTCAGTATTTTATATGTCAGTCTCACCTATGCTGAACTCATACCAGCTCCGCTGACGGCTCTAGT
GATTTTCAGAGACATTGGTTTGATAGCTGCTGTCTTCTGGGTCAGATACAAGACCGTACCTCCACCGGTGACACTCAG
TAAATTTTTTAACCCCTGCTACACCACAGCTCAGCTCAAACCCACACTGTTCAGCAAGGTAAACACAGCCATCCAGCT
CTTTCTGGTCGCAGCCTCTCTAGCTGCTCCAGTCTTCCAGTATACAGACAGTGTCCTGCTGCAGTGCATATGGTATGT
AACAGCGGTGACAACTGCGGCGTCAGGCTATAGCTACTGGCACTACGGGCGCAAGACTGTCCAGGTACTGAACACCAG
ATCGCCATGACAGCCACACCAGGAAACACCCAGAGGCCAGAACAAAAGCCGGGAATAGCCGCCGCAGAGACTCTGATC
GTCCGTGGCAAAAGTCGGACGGACAGATGGACGATCGCCAGGAGCATTGAAACCAAGTAGCTGTGACAACCGTTCGTG
AGTCACCGTGGCAATGACACGTAGAAACCAACCAAGGCCGCAGATGGTGTACTGCGAAACACTACAGAGACGCCTAAC
AACAGCTCTGCTGACTGGCTGTGGACAACACACACAGATAGAAACAGCTAACACACACATACACACACAACTATACAC
ACACATGTCAAGTATATGTCAAGT

>Sequ28469SNP2
GTCTCTGGATACACAACACACAGCTGCCTGCACTAACCCTGAGGTGAAAAGACAGGTGACGATGACTGACCTGGAGAC
CTCGATGGCCACCATCATTGCAGTATTTCAGAAGTATTCAGAGAGAGAAGGAGACAAACACAAGCTGAAGAAGAGCGA
GCTGAAGGACCTGCTTCACGATGAGCTGCCGGAGCTGATGGCGCATGTGAAAGACCAGGCCACGCTGGACAGCCTGAT
GGAAAGCCTGGACACTGACGGCGACGCCGAGTGTGACTTCCAGGAGTTCATGACATTCATCTCCGTGGTTACCGTCTG
TTGCCACGAGTTTTCGAGCATGAGGACGAGTAAGGGGGGAGCTACAGTAAATTATAAAGAAGCAGTTAAGCCTAGCAA
CCTGCCGATGATGCCTTCAGAGCAGCGCAGGGTGGGAAAACACAACTCCAGCCAGCAGCCAAAAGACTATGGGTCAGG
TTGGCTGGTGGCTGACAAACAGTCCAGTCTCGTCCTCAGCTTAGTCGTAGCGATCTTACCATTTTCTATTCTTCATCA
ATATCTACATCACTTTGAAAGCTGAGATTTGGCATCGCCACGAAGCTCGTTACAGACCAAGCCACTGATTTCTAAAAG
TCTACCACTTAATTAAAGTAGTTGAAATTCCTTAACAACATTTCACACGTCAGACAGATGTTTAGAGCTCCAGTTGGC
TGCAGAAGTTCAGCTTGTAACTTTTTATTTATGTGCTTTTTAAAAGCGGAGTGGCTAACTAGCTACTTGGCTGTAGCG
GCCGCACGAATCAATCCACTATCTAGAGGCGACTGTTTGAAAAGTTTGACATAACATAGCTGACTCAAACTTTAAAAG
TTAGTTCTTATATTAAAAACATTAGATTGGCTAGTGAAAGATTCAAAAATGGCTTCTGAGTTTCAGCCACTTACTTTG
GAAACGGCCAACAAGTTCGCTCGCCCTCCACTCATCACCGTGGCGATCAACCAGCTAATTAGCAACACCTGTGCGAGA
AACAGTTTCAGAATGACTTTGAGTTTTAGTTAATCTAGCTGCCACTTTGGAGGCCACAATTTAGGGGTTTACCAACTG
TTATATTATTTTAAGAAATGGCAGATTTTAAAAAATAATGAAATAAAAGCAACTGGCTGGTAGGTTTTGGATGTGGCT
GGAAAGTTCTACTGGCCTTGTAAAATAATTTAAGTGGTTGATAATTGCCCTTTGCTCTTGTTTTGCTTTATTTCTTGC
CCAGAGGCTCTTTTTGGAAAAATCTTTTGACTTCATACTTAGGTAAATAAATAACGACAACTATTTGAGACATGAGAC
CTTCCAAACGTCTAGTCTGCTGTTGCCTGTAGACGAGTTTCCCTCCCTGCTTCTGCTGCAGGTTTGATCCTGTTAGAT
TCAGTAGAAAACAATCTAGAAAACAAATCTAAGGAACATGTACATGCAAAAAA

>Sequ28503SNP2
ACAGGGACTGGGTGGTGGCTTTAGACAGACAGTGGAGCCCAGCCCTCTGACCCTCCACCCCGGGTAGCCATGTAATGC
CAGTCACTGCCCTTTCCTTCATGCTGTATAAAAACACACACATATATCTGTGTATTTGTAACCTGAATCTTCTTGTGT
CTGTCCATGCAGACAATAAAAAACTGTACAGTAGGTCTAGTTGCATGTAATCTGTACTAATTATTGACAATGGCATTA
TAAAATGCAATAAAATACTTATTACACTGCCAAAAAAGAAGAAACAAAGAGATTTTATTGATATTCCTCAAAACAAAA
TTTTTTACACAGACCATAAATATGACAGACCAACTACAAGTGAGTATGTGTTAGTTTCATTTATTCTGTTCAAGCAAA
TTACTTCTTTGTACAGTGAAGTGTTTGGATGAATTGGTCTAAGCAGCAGGTAACAGGCCGCCGATCTATCTAGACTAC
ATCAAGATCAGAGCATTACCAGCAGAGGGGAGGGGGGTCGCGGATGACATGTTACGCCATGCATATACTGCAAAAGGT
GTGTGCAGTGAATTTCCCCGTTGCAGCCTCGCCGTCCTGTCACGGTGAACAGTGTCAGCGCAGCATCCATAGGCCGTC
TGCAATCACAGACGAGCTGGGTTTGGGGAACAGTGTAGCCCAGCCAGCGACCCCCCCGCCGGGTGCCCTAGCTGTCTG
TTGAGGATGACTATGAGGATGGAGAACAGAGGCGGTGAACCAGGTGGACAGGAGAGAGGAGGAGGAGGAGGAAGAGTG
CTGGGGGGGGTAGTTTGAGGACGTCAATGGGGAGTC

>Sequ28542EST2
AACCAATTCACATTTTATTAGATCTTTTTTCATTAAACAAACTGGTCCAGAGTCATGGAAAACAAAAAGGTCCCGATC
TCCTGACTTGAGATACCACTGTGGCTTCACATAGACTACAGTTTTACAATGAACAGATACGCACAACAGTGCCAGTCT
CAAGTGAGGACTCTGCCCCTGACATTGTGTTTGCTAGGTGGAGCACTGTTTACAGATGCAGTCCAAACCACTGTGCAG
CATCAGCAGCTCCACAGCACGGCTGTTTGACTCAGCTCTGCTGTCAGGTCTGAAGTAGACAGACTGACTCAGTGGGTC
TTTGACCTGTCGAACAGCTGTTTGAGTTGGTCCTGAGAGGTGGCTTCTCTCTGCTGCATGAGATCAGCATGGCCCTTA
GTAACACCCCAGCCATCGGGGACAGCCATGGAGGGACACTGGGGCCGACCGGAAGCTGGCTTCAGGTTCTCCCAGTAG
TCACGGCGGGCTCTGGCTCTGACCCAGGGCTTGGTGTGCATCTCCAGACCAGCACCCCAGTTGCCGTGTTTCTCTGAT
GCAGCTGGGAGGAAGCCTCTCTCAGGGGCCAGCTCCTCTGCCACAGCCCTGATGTGATAAGGCTCAAATCCACAGCAG
CCACCAATGAAGTGAATGCCAGCATTGTAGGCCTCCCTGGCGTACTTGTGCATGTCCCAGCGGCTCAGAATCCTGGGC
TCCAGACCGAAGGGGAACTCTGGCAGATCGATGAATCCCTGACAGTTGCAGTCGGGGGTGTGGTATGCCAGGGGCTGC
ACCATGTAGTGAGCCTTGAGCCCAGCCTTCTCCACTCCCTCCTTCATCATCTTGACAGCCTTCACACAGGTCATGGGG
TCAAAGTGGCAGTTGATTCCAACAATCTGGGCACCAGCTTTGACCAGCCTGACAGCGCACTCTGCAGGAGAGATACCG
TGCATGTCTCCGTCTGGTCCGATACACAGAGAAGCAGCCACGGGCTTCCCCGTCTCCTTCAGCACCTCCACAGCCCAC
ACGGCCTCTTCAACATGCTCAAAGTACTCAGCAATCAGGAAGTCCACGTCTTTCTTGATGAACACATCCAACTGTTTC
TTGAAGATGGCCTTCACGTCTTTCTCACTCTTGCAGCTCAGGTAGGATGGAGTCTGAGACACTCCTCCGGCAACCAGA
GCGTCGCCCTCATTGGCAACCTCACGGGCCAGATCGCAGGCGGCCTCATTGATCTGAGCACCAGTGAAGGTGAGCTTG
TTGCCCCTGTTCTCCAGTTTGTCATCACTGGCATAGAAGGTGAAGGTCTGCATAACGTTGGCCCCGGCCCTCAGGAAC
TCCCTGTGCAGCTGTCGCAC

>Sequ28622SNP2
TACTTAGTTAGGGTAGGTTTTCGGTTTTTTTTGTTTTTAAATCTTTTTTATTGTTTAATTTTTTATTATTTCAGCTTA
AAAGTTATCCATTCCAGTGTTTGAGGACTTAGTGCATCACTGACTATATGTAACAACCATCCATTCACTTACCCCCAG
AGAAAAATTATTGGAAAATAGCTGGTAACAGATCTGGTGTACCTCCAGTATATACTGTATACTTAAGGTAGAGTTATA
CTTGTGCGTCACAATGTACAGCAACCTTGTCAAGGGGTCTCTGGTGTTTACTGAACAGGAGAATATAATACCATGTAG
TCATTCATAGCCAAAGCAAACCTGCTTACGTTCTTTTCACTTTGCAATAGACAGTGGCAATAAGAATGAGTAGGTTTG
TAACAGAAAGAGCTTTGCTACATCACAGAATATGTACGGTGATTCAGTCATTTTAAGATTTAAGCTTTAATAACTCAC
TTAATGTCGTCTTCACTATCGACACCATGCCATCTCCAGCAGGTTTGGAAACTTGTGAAAGAAAACACAAGAAACCCA
AGCTCGCCAATCACTGTGTCTAGCTGCTGTACATTTTTAAAGAGGGTGCACGTTAGTGTAAGTGGCTGTGTGCATAGG
CTCTATGTTTTTACTGCACAGCCTCCCAACACACAAGTATAAATCCAACTTTACACTCATAAACACTGACTCACTCTC
TCTCTCACACACACAGTAATGCAGGGGTTTGTGGAGTGCTTTATTACAGGCGTGCTTCTGGAGTTTTGGGGCTGTCTA
AGTGCTGGTAGGGAGGACCTGCAGGGTTTAGTCATACACCCAGGAAAGACACAGCTGTGTCCCTCTCTTCCTTCAGCT
CCGCTGCTGTGGCGTCCATCTTCAATCGGGAAAA

>Sequ28651EST2

GTGGGTCTGCCTCTGTGACTGAGACAATATATGACAATGGTTTAGTTGCTGACATATAGTAGAATCCAGGAAACACAA
AGAAATGTTAAAATGATCTTGCTATTAAGTTACCTTTAATGTAAAATTATGCAATACACATGGGTTGCCTCAAATGTA
ATCATACATTAATGGAAATGCTATACAGTGTGAAAGAAGTGGAGCGGGAGAGACCATATTATAGAGTAGAGCAACACA
ATGCACCAAGGCCAGGAGAGGAGAGCAAATGCCCAAATATTGTGCACATATCTTGAGTACTGAATGACAATGAAACAC
ATCAATCCATATCTCTTCACTCACATATTACGTTTAACTTCTTTAGATTCACCCCTCAAAAATAAGGGACAAACTTAG
TAAGTTGAAATCTTGCTCTTCCAAGGCGTGTGACCATCTGGTGAATCTAAGAGGAGGAGTTGGGAGCTCCAGCAGCTG
AACCCACCGTGTGGTGAAGTACAAGATGATCCCAGCCTACGGTCGTCAGGGTGTTAGAGCCCCCGTGGAGCCAGGATA
CACCTTTGACAAAGTCCTGGTGTCGCCGATCTCTCAGTATTTCTTGCAGTTCGGAGTCCATAACAGCAAGGGAGCAGT
CATCGCTGACAGTAGCTAGCAAGGGGGCACTATGTGTGGAGAAGGCGAGCCCATTAACTCTGCGGCTGTGGACGTTCT
CAACTTGGGCCGGCTCCGTTCCAAGGAAATCTTTCACTGTCACTCTGCCGAGCTCATCACCAAAAGCAATGGTGCTTC
TGTGGTGGGGGTGCCACGCTACAGTGGTAGGTGAGCAGCTGGGGGCCTCTATATCTATTCTTGATGCTGGTTTGTTTG
GCTTCCTCCTGTCCCACATCAGCACACGGCCATCTTGACCACAGGAGATGAAGAGAGACTCGTCTGTGGGGACTGCAG
GCAACACATCTGTACCGGCTGTGTGTGCACATTGTAGGTAGTGCAACTGTCTCCTGACTGAGATCCCATACTTTAACT
CTGCAGTCCATGCCGCCAGTGACGGCACTGCTTGCTCCAGTTATGGGGCTCACTGTAGTGACAATGTGGTCATGCTCA
TGTTTGGTGAAGCAATTCACAAGCAGGCGTTCATCCTCCGCCAGTTCCCAGAGCTCCAAGGCACCTGAGTCTGATGCA
ACAACAACACCCTTTTCTGACACCCATTTCACATCTGTGACACTAGCCTCAGTCTGCACACCAGCTTTGCAGAAACCC
TCATTGGGGGCTTGCTCAGGGTCACTGTAGATCCACACTGACCCCTGCCAGCTCCTGCCAGTGAGGCTAGAGGCACCA
AGCAGCAGGGTGCCATCTGCTCTGTATTGCGCCGAGCACAGATGCTTCTCCATGCACGCTGGGGCATTGGGGGGTATG
TTCCATCGGTTTTCCTTGATCATGCTGGTGTCTACTGCACTTTTCATTCAGGTATCGCAGTAGACTTCCTGTGATTTT
GCAGGCAACTTAAGCGCGAATTCTA

>Sequ28656SNP2
CTACTCCCTGTGTCGTCGTTGACAGTCTCTAGTTTTTTTTTTTTTTTTGACAAGAGAATCTTTATAAGAACAATTTAT
TAAAACAAGTACATTTTCAATATTTAAATCATAGTGTGAAAAATAAAGAACTTTCTTCTGTACTGATTTTCAGCAACA
AACAGTCTCAAAAACAACCATCACCGGTGGCTGATGCCTTCACAGGCTGAGGCACTGATGGCAAATGGATGCTTGCTG
CCCTTCTGGTGCTTCAGCGCAATAACACATTCAGTGTCCGAGTCCAGAGAGAAGGGCAAGTTCAACCTTTTCCCTTCC
TCTGTTTGAATCCTTGCTTCACTCCAGCACCTTTCTTGAAGCCCGGCTTGCCTTTCTGACCTTTAGATTTGAATGTTT
TGTCCTTATTTTTCTTTCCACCAAATGTTTTTTTCCCTTCAAATTTCTTCTTCCCACCAAATTTATTTTCTCTATCGC
CAGGTTTCTTGCCTCTGAATGTCTTTCCTCCAGGATTCATTTTCTTTCCAAATGCTTTCTCCCTGTCCCCGGATTTCT
TCCATCCTTTTCCCATTTTGTCTCTGTGCTGTCCTCCTGGACCGTTTCTCTTTGGCGATTTAGTATCACTGTCTTTTC
TCGACATCTTCCTCTTCTTGGCAGGTCCGTGAGACTTTTCTGAGCTCCGCCTCTTCTTGGCACTAGGGAACATGTCTT
CGTCTGGCTCCATTCCTACAGCTTGTCTGTAGTACTCCACCTCATCATCAGATTCAGCGACAACTTCCTGATCGTCCT
GCATGCCGGGATCCTCCACCTCACTGTCCTCTTCAGCTTCAGCATGTTTCCGCTTGATGCCCTCCAGGCTGTTTTTCT
TGTGCTCTTCTCGTTTCTCTTTTTTCTGAGCAACATTCCGCGCCTGTTTTTTCCGCCGATCCTCCTTCTCCTTTAACT
GAGCCTCCTTCCTGTTCAGGATCTCCTGTATTTCCTCCTCTGTCTTTGAGACGAGAGCATGATAAAGGATGTTCCCTT
CTCCCATGCCTTCCTGGATCTTCGTCAGCTGCAGAGTCATGCGAGGACCAATCTCGGTCAAACGAACCGCACTCTGTT
GGGACGCCATGTTGCCTCTCCCAGAGTAGACCTGCGGCAGCTCGGTGATGTTGTGCTCCCCATCTTGTTCTGCTTCAC
TTTCTGAGAGGTTCGCCCCTTTCATCAGCAGCTCACTGATGTCCTCAAACTTGCTCATGTTGGGGAACTTCTCCTGCA
TCAGCTTCTTGACTCCACGGCTCATGCCGACAGGGACGACCTTCAGACTGTAATGTCGAAATTCTATCTCCTGAGACG
CTGGGTTGTAATTCAGCAGCACACACCTCTTTGATGTTGTTGAGGCTTACCTTCTGCACATTAATGGAAGGAAACATG
TTTTGGAACATGGTGGCCATGAGTTTTATGTGCATGCCATCAGATCCAAAGTTATTGAGGATGAGTAGTGGATGGTGT
GTGAACTGCTGCTCGTGCATCCTGTGCTTCTTCAGAGATGAAACCACGTCTTTGATGAGCGAGTACTTGAGGACTCTG
AAATGAAGCATGGGACCTTTGGGAAGTCGAGCAAGTCTCATGTTGATACTGCTGGTGGTCTTGCTGAAGATCATGAAG
TGTGTCACTCCCAGTGGTCCTGCAATGGCCACAAAGTCTTTCAGCACATTCTTTTTCTTAACCTTCAGAGACTCTGCG
GTGTAGGGCTCCATGACTCTCCGCACGTCCAGGATCAGCTGACCCACGTTTTTCCCAATCTGACCCCGATGAAAGACG
AAGGAGTGGGGGACAGCTCCGTAGATCTGCTCGGCCACATGGTTGGCTGTTGCTCGGGATTTCTTCTGGTTCTTGGTC
TTTGATTTCCCCATAATTCAGCGCGGCTGGAGAGAAGGTGAACAAATCAACATTAAATGTTACTACACTATATTATTC
TGCAAAGAGAAATAACGTTCATAATGACGGCAAAACGTGTGTCTACAACC

>Sequ28666EST2
TTTTTTTTTTGGTTTTTCTACTGCTCAGCTTTATTTCAGAGAATATTACATTGAACCTGCCTCCAAAACACAGTTGTT
TAAATACAGTCTCTTATTTACAGTGTGTGGACGTGTCAGCGGCGCTGTGACTTGTCTGCGCTGGTCGGCTGACTGACC
TTCGACAGATCCCCGAAAGATGCTGTGGCCGAGCCTCGCTTCGCGGGTCTGGACTGTGACTCGTGAGGCTTCCAGCCA
ATCATGTAGAGGATCTCGAAGGTGGCGGGAACGGACCCGTCCTCGTCACCATACATCTCTTTATAAATGGCTGCTGCT
GCCAACACGCTGTCTCTGTGCAGCAGCGACCTCCTGTTCCACGCACAGTTGCTCTCGCCCATTCCTTGTAAGTCAGTC
ATGACCTCGATGATTCCTGGATATTGAACCTGAACTTCATCAATATCCACAGTGAGCATGTTGAAGCCCGCCTGGCCC
AGCAGGTTGCCCAGGTCGGTGACGGCGGTGTAGGGGGAGACGTGGGGGGAGAATCCCCCCCTCCCTCTCAGTCTCGGC
GAGCTGGAGGGAACACCTGAGCTCGTAGAGCGTCTCCCCGCCCACCATCGCCCCGATGAAGACCCCGTCCGGCTTCAG
CACCTGGTGGATCTGTTTTGAGAGCACCTGGCAGGTCGTTGATCCAGTGCAGGCTCAGGCTGCTCACCACCAGGTCGA
AGGTGTTTTCTTTAAACGGCAGAAACTCCTCATCAGCCAGGACGCAGGTGGTGGGGATCTCCGTCCGTCTGCTCTGCT
TCAGAGTCTTCTCTGAGACGTCGGTGAGGAACAGACGCTCCACGACGTCTTTGTTTAAATGCTCTGCAATGTGACTTT
TTCCACCGCCGATGTCCAACGCCAGGGGAAACGTCCTCGCAATGTCATAGACCCGATCAGCCACTCGACTGCCGACCT
CGTCCCTCAGGTAGTCGTACTGGTGACCGTCCTGCAGCGACGCGGCCCAGTTCTTCTGCCTCTTCTTCATCTCCCTGT
TGAACACATTCATGGGGCCCCTGCCCGTCACTGCCAGTCCTCTGCTGGGGGGCTCAGACCTCGGGCCAGCCCGGAGTC
TGCAGGAGGGGGACGAGATCCGGCTCCAGCTGCCCGAGGGGGCCGCAGCAGGAGCGGATCCGGGTGTGTGCAGCCTCT
GCAGGACTCTCCCACACACTCTGCTGCTCATGGTGCTGTTCGGAGCTCCAGGTCAGAACTACAGACGGAGACACTCTC
GCATCAGAGCACTGAACAACATCCGAGGTGACTTCTCCTGTCCTCCACACGTCCCCATCGTACTCTGTCGTTGACTAC
CACG

>Sequ28722EST2

GGGAGAGCTTCTGCAGCAGAGGTTGAGTTTTTTACAGTTTACTTAGCTAAAACTTAACTTTAAGTTTTACATCTTACA
CAACCAACGTCAACATCGTCCACTGACACATATGGATTAAGAGACTTCATTTGAGCCTTTTTTTTTTTGTTTTACTGA
TCATAAAGAAAGAAACTTTAGTTCTAGTTATGCGTCATCGTGAAACATCATCTCTGTGTGGCGACTAAAATTTGCACA
TTTACAAAATACGAGATTACAACATGGGTAAAAAAAAAAAAAAATGTTTTAAATTGATATCAGGAAGACATTTAGTTTC
CGTTTTAAACAACACAGTCCAAGTAGCAGCTGTGAACAACAACAAAACTATCATTCCAATACACGAGGCGGGATCGAG
GCTGTGTGGATTATATTGGCTTGTACAGAAGAGTGGTCACATACTTCTTTTTGTATCGGTGTGTTGCACTGAGAACCA
TCACCCCGTCCTTGATGGAGAGGGCGTACAGGTGATTAAGCATCACATGGTTAGGCTCCGGAAGTAGCGTCGGGTCAC
AAGAGACACCTGTGTCCTTGTTGAGCAGCACTTGCAGCAGGTGGGGAGGTAAGATAGGAGGGTGTTTGATCTTGTCTT
CTGATCTGGTCACATACGCGTCCTGTTGATAGGGGCCGGGTGGGGAACTGGACAAGTCTGAGATATCTGCAGAATCCT
GCGAGTCAATTCTGAGGGCGTCAAAGACTTCAAAGTCAGTCGTTTTCACCTGGATAATGTTATTGACTGTTCCGGTTT
TAGACGTCATCACAGCCCCAGTCGGATCCAGAGTCCACTGACCGTCCACACAGAACTTGTACTGGTGCTCTCCCTCCG
GCAGGTCCACTATAGCCACAAAGTTCTTCTGACTTCTGTTGAGCGGGATCTTTGTGGCCCAGTTGTTAAAGGAGCCGG
ACACGAAGACTTCTTTGGCGGCCCCTGACCATCTGAACACGGTTGGCCTGGCCTGAGTTGGACTCTTGCTGTCACTCT
CCAGGTCCTGCTGCCAGGCCAGAACTCCTGTATTTCCTGCGGAGCCTTTTGAATCGTCTCTCTGGAAAAGATCGGCGT
CCTCTGTGCTGTCCATCAGGATGTTGGGACGAGCCTCTTTCCCTCCCTGCTGTCCATCCCTGTATGACCTCTCACCCT
GACCCCCGCTGGATCTGTCGCTGCTGCTGTTCCCCATCCTGGAGGCATCAACCACTGTTTCTGTCTTTAGGCTCCTGT
TGTCAGCCGCCGCTGCGTTTTCGCCATTTAGTGGAAACTTCAGTCTG

>Sequ28786SNP2
ATACATCAACCATAAAATGGATTTATTTTACCAGAGGTCCAAACATATTTCTGTGTTGCCCGAGGATGCATAGAAGAT
GTCATTGTGTCATAGGAATTCAACTTCCACTACTACAAACCAGAGTTTACAATCTATATCTTCTTTTGTTTGACCATT
GGTTTGACAATAAAGCCATGTGTAGCTGTTATGTTACACAATATCAAAGTGTGTAACTGGGACAATCTTACAATCTGT
AAAGGAAAAAAGAAAGGAGAAAAAAAAGGCAGCTTAAAAAAATAATTCCATCTTGATTTGAACAAAGGGAAGAGTGTGT
TAGTGGAGGTCGATAAAGGAGAGTAAGGCTACTGGTATAGGTTGGTTTGGAGAAAAAAAAAACCCTCAGTGTTTTGTGA
TACTGTTTCACTTGTCTCTCCAAGAATAGTATGGGAGAGAAAAGGAAACTTACAAAACCACACTATTGTCATGTACTT
CTAATTTAATAAGACAAGTATTCCTAATGTGGTTATGGTTTGTGCTGCTGAGGCAGAAACAAACTGGGAATCTGCCGC
TGGTCTAGCGAGGCTCCAGCATCTGATAGTAAACGTAGCTAAGAGGCCAAATGAAGGTCAATAGAAAAGGAAAAATGG
GGAGCAAGTTAATGAAAAATAGAGCCAGGTTTAGTGAGCATCTCTGCAACCCAACTGCTCGTCAGAAAGCTGGACTGC
TGCTGTGTTTTTGGGTTAAACCATCTCCTGCTGTCAGGTAAACACGTCCCCCATCACAACCGCAATCCAACAGCATCT
GCAGCTTTACAGAACTCTGAGAGAACTCTGCTCCATTTTGGAAGTA

>Sequ28807SNP2
ACATGTAACAAAAGTTGTTTTATAATCCATCAAATTTTATTGTAATCGTCTAGTTTATGACAGGATGCTCGTGTATGA
AACTAGTAGTCATGTTTTGTGCTAGCAGTACTTTCTGCTAGAAAACTTTGGAATTTTAAATGTAATACAAAAGAAATG
GAGATTTCTGACAGGATTAAAGCTTAAATGTAAATGTAATAGAGACTGGAGCTACAGTGGGAGAAAAAATAAACACCT
AAAAACAGCAAAGGCACTGTAGCAATACTGAATAATTAAGACAAGTCTGTTTCAAACTGTGGTATAAGGTGTGTGTCC
ATGCCTGCTGTGAAGTCATAAGAACTGTCTTGATTTGAGGAGTGATACATTCAACAGTTATACGCTCAAATATACACA
TGAAACAACACACCATCAAAATTGGCATGATAATGTTTGTTGTAATCATCCGTCCAGCAGCCTCAGTGTTTACAGCTC
GGGTAGGCCAGGCATGGGGAACTGGCCGGCGAAAGCTTCCACCTCAGCCCTGATCTCCGCCACTCGCTGCTGGAACTT
CTCTCCCTGCGACAGCGCCTGAATGAACTCCTTCAGAGTGGCCTTAGGATCCAGGCTCCTCTGCACCTCCAAGGCCAG
CTGAATACCTCTGTGAATGAACTCCGCCACCTTCCTGAAGTCATCCTCCATCAAGCCTCTGGAGGTCAGAGCTGGAGA
GCCAAACCTTAGACCACTAGGGCGCAAAGCACTCTTATCCCCTGGACAGGTGTTCTTATTGCAAGCAATGGCACAGGC
TTCCAGAACCTTCTCAGCTCGTCCTCCGTCAGTTCCCTTGCTGCGCAGGTCCAGCAGGATCAGATGGTTGTCAGAGCC
GCCAGTGACGATCTTGTAGTCATGGTCAATAAGGGCACTGGACAGAGCTTTGCAGTTAGCAAGAACCTGAATCTGGTA
GGCCTTGAACTCCGGTGTCATGGCTTGTTTGAGAGCGACAGCAACACCTGCAATAGCGTGGTTGTGTGGTCCTCCCTG
CAGCCCAGGAAACACAGCCTGATTGATCAAAGACTCCAAGTTGTACATCGTCTCTTTCCCCTTGGCGTCCACACTCCG
CACACCTTT

>Sequ28865EST2
ATTAGGGAGGTCGGTTTTTTTTTTTTTTTTCTAAAAGTTAGAATGGCTTTATTTGATGTTTGAAGGCGAGGATGTTTTG
TACTTGTATCAAAGGATCAACAGTCTTTTCAAATGAAGGACCTGAGCTTCGGTTATTGGATAGTTACATGGACGTTGG
AAAAGCTATGGAGCGTTGTGTTTCACACTACATCGCCTACACATCTCAAACTAAGACCAGTAAACATCAGATGGAACC
ACTCAGTGAATTTACTCTGTCTGTTATGATTCAGATCTTGTTGAAAGCAGCCACGTCCACGGACTGAACATACTCCTC
AAAGGCGGTGATGGCTTCCTCCAGGAGATCCGTACCCACTTTTTCGTCCTCCACTACACATCCTATCTGGAGCTTCTT
GATGCCGTAGCCCACTGGGACCAGCTTGGACTGCCCCCACAGCAGCCCGTCCATGCTGACGCTGCGGACGCACTCCTC
CAGCTTGGCCATGTCCGTCTCGTCGTCCCAGGGCTTGACGTCCAGCAGGATGGAGGACTTGGCGACGAGAGCTGGCTT
CTTGGACTTCTTGGCGGTGTACTCGGCGAGTCGCTGCTCCTTGATTCTCGCCGCCTCTGCGCTCTCTGCCTCGTCGTC
CGAGCCAAATAGGTCGATGTCGTCGTCGCTGGCGTTGTTTGTGGAGGCGGCAGGGGCGGCTGGGAGGACAAACTGACT
CTTGGCTGACGCCAGGCTAGCTCTTTCCCTCTGGAAGGACTTGATGTGCTTGTACCAGCGCAGGAGGTGGCAGAAGGT
CAGCGACGGAGCAGAAGGGATGGCATCAAACACTGCTATGTCAGCTTGAGATGCTGCAAAGCCCTCCACGTAGCTTTT
GTCTGCCAGAAACTCATCGAGAGCTTTGAGACCGGCTGGCGAGCTCATGTCACCAAACACAGTCTTTGTAGAGGTCGG
GCGGGAAGCCGGGGCCTGACAGGAGTCGCTTAG

>Sequ28896SNP2
TGATGACGACTTCACTATGCACAGACAACAGAGCAGGTTTATACAAAGTGATCTGGACCTGATATATTCACATAGTTT
ATTAAACAATTTCATGAATGATTTGATTTTCTTTAATTCTGAGAGTTTGTTTCACAAAAAAGTTAATTCTGGGTAAAA
TGAAAACAAAGTGGCATCACTCAGTGAGCAGGAATGGAGATGGGAACAAAGTCATAATTTTCTATTATGAACTCCAGC
TGCAGGTGAACTGTGTGTACACAGAGGAAACACGCAGTACAGTATTACCACAGCTCCATCAGAGGTACTTATACACAA
CTTTCTCGTACAGCGTCTGGATCTCTAACTTGACGGGACATTTGTAGAGGTGCGACAGTATCGACTCCGTGTAACCTA
TTAAGAAGTAAAACTTAACAGGAGGGAGTTTCTGTATCATCAGAGCGCACACGATGAGCATGTTGCCGCGCCTTTTGA

TCACAATCTCATTGGCCAAGCAATTGTGGAAGGTCCCGAACATGAATCTCCTGATGAACACGTCCTCTACGGTCCGCT
CAGCCGCCCCCTCCTCTCCTTTCAGGTTACTGGTATGCTGTGAAAGCCATCCTTTGCGGTGGCCAATGTGATGAGGGT
GAAGTGCTTGCTCATAGGTCAAAGGTCGGTCACCTTTCCCCACTCGGATACGAGCTGCTCGATTCTTGCAGCACACTG
CAGTCACGTGGAGGCCTCTGCTTCCAGAGGAGCTGCACAATGACCCGGCCCTGGGTAAGACACTCAGTAGCCTCACCA
CACCAGCGTTGCTCAAGGACACCGCCATGTTTTCTGAATGTTTGTTAGCGCACTATGCTACAAGAGCTAACGCTAATC
TCTAATTTGTTTATTTTCCAGTGCCGTCGTAGTTTTTAAATATAATTAACTCTTTTTGAGCATTGCGATGATTTATCA
CCCACCAAAATGACAGAAAGTTTAATTTTCATGGAAACTGCGTCTATAAACTGGGACTGTTTGTTCGGTAATTG

>Sequ29016EST2
AAGCTGCTGTAATCATGAATGAAAATATAAGAGAATGTCTTTGTGTTAAAGTCTGAATAAACACTTCACGTCTTTCTG
TCTGCGTCAGTCTGTTGAAGGTCATGAGTCCTGATGTTCTGCTCATTAAGGGGAAACACAGTAAAGTGACACTTACAA
CAGTTGTCACCGTTTTAATCTGTTGCTATGACGACTCATAAACGACTGGAGCGTTTCTCTTCCTGACTGATTTCCTCT
GCAGCATCAGTGACATGAAACCTTTCAACAGGATCTGAAGTTTTCACCTGATTCGTTAAAAACAAACAGGAAGTTGAT
CCTGCGTCATCAGGATAAAGTCTCAGTCCGAAGCTACACGAGCTTCTGTTTCTTCCGAGCCTGAAACTGCTCCGTTTT
GCTCTTGATGGACTTGACGAGCAGAGACAGGCCGGGGTCCATCGGCTTCCTCGCCGCCGGAGCGCCAGAGTCATTTTC
CCGTGTGGCGCCAGCGGTGGACTCGGTCGCCCGAGCGGCGAGGACGTCGTCCTTCTGCCGCCGCTCCTCCTCCCGTCT
GCGCTGCTTCTCGGCCAGGAGGCTCTGAGTGGCTTTAGTCTTCTTGTAGCTGGGGTCGGAGGGGTCCAGGTTGAACAG
GTGGGAGGTGAACATGGCCTGGAAACGAGGGTCTTGAACGTCCACCTGGAATTCGTCGTCCTCCGCAGGCTCCGCCCC
CTTCTTCAGCAGCTTCTTCCTCTTCTTCTTGCTCAGGTTCTGCTGCTCCACGATCTTGTCGTAGTTAAAGTGTTTGTG
TTTGGCCTCGCCGGCGTCGTCCTCCATCAGCAGCGCCATCTCAGCCTTTTGCCTCTCCAGTTCCTCTTCCTCCTCAGC
CGTCCGCTCCTCTTCCTCCTGCTTCTTCTTCTTTCCCTTCTGTTTCTTCTTTGGATCTGTGGCGCCGAGCTCCTCGGC
GAAGAAGGGGTCACTGAGGTCGACGTCTGGAGGGAGCTCGTCATCGCTGAGGTCGTCCTCCTGTTTTCCTCTGAGACT
TCTTCTGCTTCTTCCTCTCTTTCTTCTTCTGCAGGAACTCCTCCCACGGCGTCAGCTGGTCGTTCCCCTCCAGCTTCT
CTTCACCAGCTGCTCCGTCGTCTCCTTCAGCCCTGGCACCCAGGTGATCTCCATCTGCATGTCTTTGTCCTCCTGCAG
CTTCTTCTCTCTGTCCTGCAGACCTCTCAGCAGCTCTCTGTACTTGGAGATCTGCTCCTCACTCTTCTTCTTCTTCGT
CATCTTCGTCTTCTCCTCTTCCTCCTCAGGTTTCTCCTCTTCCTCTTCTGTCCGTTTAGCCTCCGCTGTGACACCTCC
GAACGCAACCTCTCCGTCCTCCTCCTCTTCCTCGCTGGAGGAGGCCAGGTAGGCGTTGAAGTCCATTTCCAGCAGCTC
GTCCTTATTGAACTTCCTGTTGAGGGCGGTCACCCGCTCGTGGTCGGTCTCGTCCCACGTCAGCTGCACCTTCGGATG
TGGCCGCGGCAGATGAGGTGAAGAGTTTGGGCGTGTACGCCGTCAGGTTCACGTCCGTCGCCGCGTCTTTCGGCTCCT
CGGGAAACGTGATGTCATCAGGAATAACCGGAGGTCCATGACGGAGCAGCTGCTCTCGTACTCGTAGCCGTCACACTC
CTCGTAGATCTTGGCGGCCGTGTCGACCGAGTCACACTCCACCACGGCGTAGTAATACTTGAGACGCTTGAACTGGTA
GTCGCGCTTCTTCTCCCTGTAAACCCTCTCCTCCTCAGTGTCGTCCTCCGAGTCGTCGGGCAGCGCCCTCAGCTCCAT
CGGTCCCTGACTCTCCTCCACCTTCAGCCTCTCCTTTCCAAAGTCTGACGGGTAAATCTTGACAGACAGCACGGCTCC
TCCTTTCGGGGTGAAGGAGCTGCACAGAGCCAGCAGGTCTTTGGCCTTCATACGGTCCCAGTCCATGTTGCAGATGGC
CAGCCGGACAGACACCTCGTCACGTCGCGGCGCGTCTTTGCACAGTTCTCCCCAGTCGTGTTCGATCTCCTCTTCCTC
TTTTCCTCAAGATGGCGTCCACGTCGTCATCGTCGTCTTCGTCTGAGCTCGTCTCTACGTT

>Sequ29158EST2
GGACTCTTCAGGAATTGAAGGGGGTCACTGTTACCAAGTGCTTAGTGCTGCAGCTGAAACTGGGAAAAAGAGATGGCA
CCTGTCAAGAAGGGTATCCTTGAACGGCTGAGCGCTGGAGAGGTGGTGATTGGCGATGGAGGTTTTGTGTTTGCTCTA
GAGAAGAGGGGGTACGTAAAGGCTGGGCCATGGACTCCTGAGGCGACTGTCACCCATCCTGAGGCTGTGCGACAGCTG
CACAGGGAGTTCCTGAGGGCAGGATCTAATGTCATGCAGGCATTCACCTTCTACGCCAGTGATGACAAACTGGAGAAC
AGGGGTCAGACACTGAGAATCACTGGAGCACAAGTCAACGAGGCAGCCTGTGACCTGGCAAGGGAAGTCGCCAGTGAG
GGCGACGCTCTGGTAGCTGGTGGAGTGTCTCAGACTCCATCCTACCTGAGCTGCAAGAGTGAGACAGAGGTGAAGGCC
ATCTTCAAGAAGCAGCTGGAAGTGTTCATGAAGAAGAATGTGGATTTCCTCATCGCTGAGTACTTTGAGCACGTTGAG
GAGGCCGAGTGGGCCGTGCAAGTGCTGAAGACCAGCGGGAAACCTGTAGCTGCTTCTCTGTGCATCGGACCAGAGGGA
GACATGCATGGCATCTCACCCGGAGAGTGTGCTGTCAGGCTGGTGAAGGCTGGTGCCCAGATTGTTGGAGTCAACTGC
CACTTTGACCCTATGACCTGTGTGAAGGTGTTAAGATGATGAAGGAGGGAGTGGAGAAGGCTGGGCTCAAGGCTCACT
ACATGGTGCAGCCCCTGGCATACCACACCCCCGACTGCAACTGTCAGGGATTCATCGACCTGCCAGAGTTCCCCTTCG
GCCTGGAGCCCAGGATCCTGACCCGCTGGGACATGCACAAGTACGCCAGAGAGGCCTACAAGGCTGGCATCAGATTCA
TTGGCGGCTGCTGTGGGTTTGAGCCTTACCACATCAGGGCTGTGGCAGAGGAGCTGGCCTCTGAGAGAGGGATAATCC
CTCCTGGCTCAGAGAAACATGGAATGTGGGGTTCTGGTCTGGAGATGCACACCAAACCCTGGGTCAGAGCCAGGGCCC
GTCGTGACTACTGGGAGCAACTCATGCCTGCATCTGGTCGTCCCACATGCCCATCCTTTTCCACACCAGAAGGCTGGG
GTGTGACCAAGGGCCACGCTGACCTGCTGCAGCACAAAGAGGCCACCAGCACCCAGGAGATGAAGCATGTGCTGGAGA
TGCAGAAGAAGGTCAAGACATCAGCATGAGCGAGAAAACCGTTGTCAACAACAGCGGGACAAAGAGGAAGGAGTGAAG
TCTTTCCCAGTTCAGTTTGGAGAGAAAAAGCACTTGTAACAGCGATGCTCCAGTTGTGGAGTATTACTTTGTAACCCC
CAAAACAATAAACCTGACTTTAATAAACTAAAAAAAAAAAAAACCGAAACCT

>Sequ29769EST2
TAAAAAGAACNTAAAAAAAAACAGCTGTGATGTCAGAGGACAAGCTCAATGACATGAGGAAGGAGGCGGGGTTCAGGG
CGGCAGCAGTCTGTTTTAGCCAATCAGGGGGAGGACTGGGGGCAGAGCTAAACCAAAGAGCTGTCTGTCAGAATGTTG
CCTAGCAACAGCAGTCCTCCAGCCAATCAGGGCAGGGGTGTGGACTTTGAGCAGGGGAGCAGGTTTGGGGGGTGGTTA
AAGGACATCATCACTCTCAGCCGTGTGGAGCTGCGTGTCGTCAGAGTCCGTCATGATGCTGCTGTCTTGACTGTCGTC
CACCTCCGCTGCTTCGTCCTCTGGCAGGTATCTCTTATCGATGGCCTCTTTGATCTGGTTGTTTGCTCCTTTAGGATC
CAGATCCATTGCCCAGCTGAAGTTCATCAGAGCCAGGTGAGTCTGACCCAGCTTCTTATACACCTTTCCTATCAGGAA
GTAAACCAGAGACTCTTTGGGAACAATCTGTTTCAACTCCTCCAACTCCTGAAGAGCTGCCTTGTACTTGTCATTGGC
GAACAGTATAGATGCTCGGTGGAACTTGCAGAGCGGTTTTTAGGTCGATCCCGATCGCTCTGTTCAGAGTTTCTAAAG
CTGCATCAGACTTCTTCAGCGCATGCTGCACCACTCCGATGTGGCAGAGCAGCACAGAGCTCTGAGGGTTGATGCTCA
GAGCTTTCTTGAAGTGGATTTCTGCCAAATTGAACTTCTCCTGTTTGTAGTAAAATCATCCCCAGACCGTACCATGCA
TTGTAGTGTCGGTTGTTGACTCTGATGGCGTTGCGGAAGCAGGCAAGTGCTCGGTCCAGCTCCTCTGTCAGAACAAAC

TCATGACCCAACAGAGTGTACGCATATGCAAAGCCTGGGTCCACCTGGATGGCTCTCTGGAAGAACTTTATGGCAATG
TCATGCTCCTCTGCAGACTGAAACAGTTTCCTGCTACACACCAGGCCTCAGGACAGTTCTTGTCCATGTCGGTCAGGT
CTTTGGACAGGGCTGACAGAGCCACGTCTTTCTGCAGGTGCCA

>Sequ30450EST2
ATGGTGTGGTGATGCCCGATGCCAACAAGGAGAATACTCTGAACCAGCTCGTGGGTGCAGCGTTTGGGGCAGCTGGAC
AGCGCTGTATGGCTCTGTCCACAGCCATCCTAGTAGGCGAGGCACGGAGCTGGCTGCCGGAGCTGGTGGAGCGTTCCA
AGGCTCTGCGCGTGAATGCAGGAGACCAGCCTGGTGCAGATGTGGGGCCTCTGATCTCTCCCCAGGCCAAAGAGAGAG
TCTGCAGTCTGATCCAGAGTGGCGTGGACGAGGGAGCCAAGCTGCTCCTTGATGGCCGAAATGTTAAAGTTAAGGGTT
ACGAGAACGGCAACTTTGTGGCTCCCACCATCATTGGCGGTGTCACATCTGAGATGAAGTGCTACACTGAAGAGATCT
TCGGGCCTGTGTTGGTTGTTCTCGAGGCAGACACTCTGGATGATGCCATCAGCTTGGTTAACAGGAACCCCTATGGCA
ACGGTACAGCTATCTTCACCACAAATGGCGCCACTGCACGCAAATTCACTCACGAGGTGGACGTGGGCCAGATTGGAG
TCAATGTTCCCATCCCTGTTCCGCTGCCAATGTTCTCCTTTACTGGTTCAAGAGGCTCCTTCAGAGGGGACATGAACT
TCTATGGAAAACAAGGCATCCAGTTCTACACACAGATCAAAACTGTAACCTCGCAATGGAAAGCTGAAGATGCCACCT
TGAAAAGTCCTGCTGTTACCATGCCTACTATGGGACGCTAAATACTCCATACAAATGCTACTGTTCAGTCTGATAAAC
CAGAACACCTTAATAACCCTTTTACAATGCACAAAATCGACTAACTATGTTCACTGTGAATAGTTCATGAATAGTTCA
TGAATACTAAATACTCCCCTTTTGGAAGATTTTATATTAGATGTGTGTATATATAGATATTCTTATATGTAAACCACT
GTGGCCAAGATTTCAACAATGCAAAAGATCTGTACAGATTAGGCAATGTTTGTACGGATCCAAGTCACTTCCTCAAAT
ACAATATTGTCAAGGACTTAAAAAAAAAAAAAACCGACCTACCCTAACTA

>Sequ36281SNP3
AGTCATTTAATTCGTCACCTATTTTGCCTGCATTTATTTAGTTCATCACCAGTTATGGTCTATGTTTTATTTCTAATT
ATTAAAAAAAAAACTTAGTTGTTGTTAGTAAACGTAGCTAGGAACCTTTTTTCATTGGTTGATAACTAGGAAGTTATT
AGAATAATTTTAGGGGCGCGAAACTAAAGAGCAGATTCTGTTATCGTTTTTCTCTGGAGCTACTTGAACTTTTGAAGA
GGCTCGTTATTTTGGGGTTTTGCTGCCTTTTTTGTCCGCCATGTGTGAAAAGATAACGAAGGTGAACAGCTGGCTCAG
CGCGGTGTTTGGGGATCAACCGGTGCCGCAGTTCGAGGTCAACACCAGAACAGTGGACGTCCTGTACCAGCTGGCTCA
GTCCAGTGAAGCCCGGTGCAGTGACACGGCTCTCCTTATAGAAGACCTCAAGCAGAAAGCATCAGAGTATCAGGCTGA
TGGTGCACATCTCCAGGATGTCCTTCTACAAAGTGTTGGTCTGTCCTGTGCAAGCCTGTCGAAGCCCGCTGCTGACTA
CCTGTCTGCTTTAGTGGACAACGCTATGGTGCTTGGAGTGAGAGACACATCACTGGGCAGCTTTATGCCAGCAGTGAA
CAACCTCACCAACGAACTTCTGGAAGCAGAGAAGTCCAACAGAAGACTTGAGAGGGAACTCAGGGCCCTCAGAAAGAG
ACTCGGAGCTACTCTGGTGCTGCGGAGCAACTTACAAGAGGATATCAACAAAACTGTTAAAGCTCAGGCAGTGGAGAG
CGCTAAAGCGGAGGAGAAACTGCTCAACATGGATTTTGTGACGGCGAAGGGTAAAGAGCTCAGCAACAGACGGGAGAG
GGCAGAGGCTCAGCTTGTGTCCAGGAACATGGACAAGTCTATCACCCACCAGGCTATTGTGCAGCTCTCTGAGGAAGT
CACTACACTGAAACAAGAAATAATCCCCTTGAAAAAGAACTGGAGCCTTACATGGACCTGAGCCCAAACCCGTCTCTT
GCTAAAGTGAAAATAGAAGAAGCAAAAAGAGAAATGGCTGCCCTTGATGCACAGTTTGAAATGAATGTGAATTTCAAG
TGAAGATGTT

>Sequ36290SNP3
TCTCCAAAAGATGTGAATGGCTTTTTGCTTCGGAAAAATCAAGACTTAGGTCAATACGCGCTGACATGAACAACAAGA
GGATGGAATTTTTTTCCCACTGCATTGTTCAGCGTTTACTTACTGCTCATGTCAGCTGATGCATGTACATGTGTGACA
CACAGACGTGCACAGCAGGGTGTGTCTTCCTGTGACTCCTCTGCATTACTCGCTCTAGTCATCGCACAAGGATTACCA
TTAAAATGCGTGAAAATTTTCCACCACTTGTGTCGACCACAATGCGGCATCGTGTTGTTGCAGGTCTGCCCTCATGAG
AACAGAATGTTTTTTTTTTTTCTCTTCTCTTTGGGTTGCTATGGGGAAACATATGAAGGCGGTGCTAGTACTTTTCCC
ACTCTGTCATGCCAGCCTTATAAGTTTTTTTTTTTTTAGTAGTTTCCTCATCTCTGCAGTGAAGCAACTTAAAGTTA
AGTTTTACAAAAGAAGGTGCACTAGCATCCAGGAGCTACCTCTGACAGCACCCTCTGACTTCACAAGCTCTCCCTAAG
TAAGAAGTAAAAAAAAAAAAAAACTGGGGGACCCATGGCAGAAGGCGTATGACTTTGCTGTTGCAGTGGCAAGAAAAGC
TGGAGCGGAAATTAGGAAAGCTGGGGAGAGTGAAATAAGGGTCATGACAAAAAGCTCCACTGTAGACCTTGTCACAAA
GACTGATGAGAGGGTGGAGAAAATCATCATCGGGTCTCTTAAAGAGGAATTCGGAGAAGGCACACACTGCTTCATTGG
GGAGGAGTCGGTGGCGAAGGGGGAGCCGTGTGTCTTAACCGACAAACCCACGTGGATCATTGACCCGGTGGACGGCAC
CACAAACTTTGTACACGGATTCCCATTTGTGGCTGTGTCAATTGCCTTTGCGGTCAATAAGGAGTTGGAGTTCGGTGT
GGTGTACAGCTGCTTGGAAGACAAGATGTATAAAGCAAGGAAGGGGAAAGGAGCTTTCTGCGACGACGAAGCAATTCA
GGTGTCCGATGTAGCAGAAATCAAGAAGTCCATTATCATTTCTGAGCATGGAACCGACAGGAGCCCAGAAAAAGTAAC
CAAGATCTTCTCTACCATGCAGAAGATCCTCTGCATCCCCGTGCACGGGCTCCGTGGATCAGGGACAGCTGCCACCAA
CATGTGTCTGGTGGCGTCGGGGGCAGTGGAGGCCTTCTTTGAGATCGGCATCCACTGCTGGGACATCGCTGCTGGTGC
GGTGATAGTCAAAGAAGCTGGAGGAATATTACTGGATGTTGATGGCGGACCATTCGATTTGATGTCCCGAAGGATGGT
TTCAGCAAACAACGATGTTATTGCTAAGCGCATCATCAAAGAAATTGAGGCGTTCCCAGTGGTGAGGGACGATGCTCC
TGTGCAGAAGAAATGAGAATATTCTCAAACGCTGTTTACACTGTGCACAAGTGCAGTCTGCCGCTATTTGTGTCTGAA
TAAAGTTACTGTTCTGTAATCACCACTGATAATCTTAATTTGCATGCTGAATGTGTTTCATTGCCTGCACCTTTTTC
ATTCAAAGGAAAAATTTCAGCTCAACTGAAGCGACCTTGAGGTCCAATTTGATGACTCTTCATGTGATTTGTCTGTCA
AAAAATATTGATTGAAGTGGGGAAACAGCTGTAATTTTGAACTCGGCATTTTTGTACATCAACATAATTTGCACAGTT
TGTCTTTGGTCTGTGGCTGTCCATGTTTTCACTCGATCTTTCAATACACATCAAATACTCCGCTTGTGAATCCGCT

>Sequ42205SNP3
TGAAAAAGACTAATCATTCATCAATGAAAAACAGACCTCAGACGTTCTCTGAGTGACACAGGAGTATCATGTGACCAA
TCTCTATGCTGGCTATGAAAAGGATTCATTCAAGCTAATGGGGACTGTTCTGTGGGAAATATAAATGTATCTGGTTAT
GTCTTATGGAGCTAGAAGCAGCTAATTACTGGCTTCTTTCATTTCCAAGGTTAAGAATGTCGTTAGGCTTTTGGGTTC
AGATGAAACAGCTCTCCGGTCACCAAACGAGCACCGTGAACACAGAAACACTGATACCTAAAGGAGCCTGCAGACTGT
ACACAGACTATAAGTTTACTGCAGGTCACACTGTGCAACATGGGTCTAATAAACATGGCTGCCATGGTCTCCATCAAG
GCAGAGAAAGATGAGGTTATCCGGCCCCTGTCCACCCCAGCTGCAGATCCCCAGCCAAAGAGCCTCTTGTTATGGGGG
ATGAAGAGGCAGAGCTCAAATCTGAGGCTGCTCAGCCGAGCCTCCCCACAAACCCTAGAGACCTGGACCAATGTGTGG

CCATCTTGAACAACCCTGAGTTGGGGCCTCGTTTCCTGAGTGATGCTGAGGTGATGCTGCTGGTCAACTCCAAACACA
TCCCAGCGTACAAACTGGAGGCCACCATGGAGAGGCCAGAGAGAGGAGTGGCCATCCGGAGACAGATGATCTCTTCGA
AGCTGCCCTCTCCTTCTGCGCTTTCCTCTCTGCCATACACACACTACGACTACTCCAAGGTTATGGGCACCTGCTGTG
AGAATGTAATTGGCTACATGCCCGTACCAGTGGGCGTGGCTGGACCGCTGCATCTGGATGGGAAGCATTTCCAGGTTC
CCATGGCAACTACAGAGGGCTGCTTGGTTGCCAGCACCAACCGCGGTTGTCGAGCAATTGCTCTGGGAGGTGGAGCCA
GCAGTCGCATTCTGGCTGACAGCATGACTCGAGGACCCGTGGTGAGGCTGCCCTCCGCCTGCCAGGCTGCAGAGGTCA
AGGCCTGGCTGGAGAGCACAGACGGTTTTCATGCCATCAAAGAAGCCTTTGACAACACGAGCAGGTTTGCTCGGCTGC
AGAAGATGCTGGTTGGTCTGGCTGGAAGAAATCTGTACATCCGCTTCCATTCCAAGACTGGAGATGCCATGGGGATGA
ATATGATCTCTAAGGGTACAGAGCAGGCTCTGAGCAGACTGCAGCAGAACTTCCCAGACCTGCAGGTGGTGGCTGTCA
GCGGAAACTACTGCACTGACAAGAAACCAGCCGCCATCAACTGGATCGAGGGCAGGGGCAAGTCTGCCGTCTGTGAAG
CCACCATCCCCGCTAAAGTGGTCAGAGAGGTTTTGAAGACGACAACACAAGCTCTGGTGGAGGTGAACATCAGTAAGA
ACCTGGTGGGCTCCGCCATGGCAGGGAGCATCGGTGGATTCAACGCTCACGCAGCCAACCTGGTGGCTGCCATCTACA
TCGCTTGTGGACAGGATCCAGCCCAGTCAGTGGGCAGCAGTAACTGCATCACCCTGATGGAGGCATCAGGACCAACAG
GAGAGGATCTGTACATCAGCTGCACCATGCCTTCTATAGAGCTGGGCACTGTGGGAGGGGGCACCAACCTGCCCCCCC
AGCAAGCCTGCCTCCAGATGCTGGGTGTGCAGGGAGCCAGTCAGGACTGTCCAGGGGAGAACGCCCGGCAGCTGGCCA
GGGTCGTATGTGCCACCGTGCTGGCCGGAGAGCTCTCTCTGATGGCTGCTCTGGCGGCTGGACACCTGGTCAAGAGTC
ACATGACACACAACAGGTCCAAGGTGAATCTCCAGGAGACTCCAGGAACCTGCAGCAGGAAAGCGTCCTGAGAGCAGT
GTGGCCTCCTGCAGGGGCAGTAACTTCAGGAATCATATGGACAATCTGAAAAAGACTAATCATTCATCAATGAAAAAA
CAGACCTCAGAAGTTCTCTGAGTGACACAGGAGTATCATGTGACCAATCTCTATGCTGGCTATGAAAAGGATTCATTC
AAGCTAATGGGGACTGTTCTGTGGGAAATATAAATGTATCTGGTTATGTCTTATGGAGCTAGAAGCAGCTAATTACTG
GCTTCTTTCATTTCCAAGGTTAAGAATGTCGTTAGGCTTTTGGGTTCAGATGAAACAGCTCTCCGGTCACCAAACGAG
CACCGTGAACACAGAAACACTGATACCTAAAGGAGCCTGCAGACTGTACACAGACTATAAGTTTACTGCAGGTCACAC
TGTGCAACATGGGTCTAATAAACATGGCTACAACATCAAGGCTGATGTATGTGGAATGTAGGATGATTAACCCCTGAA
TCAGCAGCTATGGCACAAGTCAATAGACAAGATTAATGTTTATATCAGTGAGCGTCATCCATCTGAATCGCTATGGTG
GTAAACAATGAAGCTTAGCACAAATCGATTAGATTGGATTAATAGTGATTTTTATTGTATGGAAAAAAAATCCCAAAA
TAAGCGGAGGAGAATCTGGACCCGGGTGTGGTGAGGGAAAAAGAGGCAAACGTAGGACCTAGAGATTGGGTTAGGGAG
TTGGGTTAGGAGAAGTATTTCATAATTTCATCCTATTGGTTGGTTTGTAGACGTAGGTCACAGCAGCATCTTTGAATC
TCTCTCAGTGTGACGCTGGACCACGACCAAACATATCACCCAAGTTTGTTTCACATCATCTTTGGTCCTGGGCGGCCC
AAAGTCGCACAGTGTGTGCCTGCCTTAAAAAGTGAAGGTGTCTGTTGAATGTGTGTGAAAACTGAAGATCATTGCTGC
TCACTACAGATTCTGCATCACTGGACCGACATCAGGAAAGAATAAATACGTGAACGATTATCACTTTAAAACAGACTG
CAATTCAGAGGCTGAGTTCATCTGCAGGTACAGAACAGGTGAGATAATCTCAGGACTGTGTCTGTACTGCTGATAGGA
CATACAACAAATCATTAGGCTGGCCATTCCCAGACTTCCCAGAATGCCACAGCCCAATTTAAATACGCTCATCTTCTG
TGGCCCACCCAAACCTTTAATGACAACACAACTCACGAAACTAAGATTAAGTGTTTCCTGATACATATTTTTGTTTTA
TAAACAGAACAATTCTGCAGGTTAATAATCCATCTATATACACATAACAATTAGTTAAGTAACTAATTAAATCACAGT
GAATTGTTGAGCCTGTATCCCGGCTGTTTGACGCTGGATAATAAAACACTTATAAATACAGTTTCACAATATCCAACA
TAACATCAGCATGAAGCAAAGAGATAATATGACAGTCACCGCTCCATTTGAACTGTTTTCCCACCAATGACAAGATAT
AAGAAACAGGGCCAGATCAATGATTTACTTCCATAACTTTGTTTTACTTTTTCTTATTTAAATATTGACTCATGGATA
TTTTATGGGCATTTCAAACTTAATTACCAAATTAATTATTGAAAACAATTTATTTATTTATTGTAAATGACCTCTCAC
TGCCCACCTGCAGTAGCTCTACGACCCACCAGCTTACAAACGTGCATGTGCGTGTGTGTGTGTGTTTGTTATCTGTGG
AGATGAAGTGACTAAACGTCTGCTTGAGTTGCATCTGTAATTCATTAATAATGTCTGCTTCTAATCAACACAAAGTTG
TGTTGTCACTGTGTGTGTGTGTGTGACAGTGGCATCAATCAGTCTGTGTTTTTAACTGCATTAGCACAGTGCTGATGC
TTCAAGAGTAACATGCTGCAGACGTGTGAAATGAATTCACTGACAGTGAGTTTTCCTGTTGAACCAGCAGACAGCCGG
AGGTTCTGGATATATTTTGTAATAAACACAAAAGGACTTTA

>Sequ42207SNP3
AATTGCATATGCGTGTGCACGTGTCTGTGTCGCCGCCACCGTTACGTCATCCATGTTCGCCGGATGATTGTCCACAAC
GTGTTGAAGGAAGATTCACGTGTGATATTTTTTGATCTTCCACCGTACAGCCAGACGTTGGAACAAAAAGTGACAGAT
TAATCGGGTATCTGTGTAGCTCGGAGTCATGGGTCGGAAGTTGGATCCCACCAACAAGGTGAAGAGAGGGCCAGGGAA
AAAATCCAGGAAACAACAGGGAGCAGAGACCGAGTTGGCCAAGTTTATAACTGATGAGGAAACTGGACCAAAACGTCT
CTCAAGTAGAGGCAGGAAAAGAGCTGCAAAGAGAGTCCAGAATTTGAAAAAGCCAAAAGATGTAACAGAGGAACAGCC
CAAGAAAGGATTCACTGATGAGAACAGTAAATGGTTGAAACCAGCAAAGAGGAAGCGCAAAATGAATGAGCCTGAAAG
TGAGGATGACAGTGAAGAGCACTGGGAGGAAGAGGAGGATGAGGAGGAGGAACAGGAGCTGCAGCAGCAGCAGAAAGG
AGGAAAAGACCAAGGAAAGAAGGGTGCAAAATCAGGAGTGAAACAGGTTGAGGAGGAGGAGGATGATGATGATGATGA
CGGTGATGGTGATGATGACGACGACGATGATGATGATGATGAGGAAGAAATGGTTGATGACTACGGCACACTTGATGA
TGGCAGTGCAGATGAAGCAGCAGAAGAAGAAAGTGAAGGAGAGGAACTCCTTCCTATTGAGCGAGCAGCAAAGAAAGA
GAAAAAGCTGAAAGAAACCATGGCTCTAGGGAGTGATGACGATGACGATGATGAGGAGGAAGACGACGAAGGTGATGA
TGATGAAGAGAAATCAGATGCTGACATGGAGGAAGAAGACACAGTACAAACCAACATAGATGAAATGGACATATTTAG
GCTACCTGGGGCAGAGGAGACTGAGAAGGAAGGTGTCCTGCCTCTAGACCTGAAGACAATTTATCAGAGGATAAAGGA
CAATATTGATGTCCTTTGTAATTTCTCAACAAAAAGGGAGGAGGGCAAAGAGAGAGCAGAGTATATCTCGCTCCTGAA
GAAAGATCTCTGCACCTACTACAGCTACAACAACTTCCTCATTGAGAAATTAATAGACCTCTTCCCTCTCTCAGAGCT
GGTTGATTTCCTTGAGGCCAATGAAATTCAGAGACCTGTTACTATTCGGACCAACACACTTAAAACGAGGAGGCGGGA
CCTTGCACAGGCCCTCATCAACAGAGGAGTGAACCTTGATCCACTGGGGAAATGGTCTAAAGTGGGTTTGGTGATCTA
TGACTCCTCAGTACCTGTAGGTGCAACTCCAGAGTACCTGGCTGGTCATTACATGCTGCAAGGAGCCTCCAGTTTTCT
GCCCGTCATGGCGCTTTCTCCACAGGAGGGAGAGTTAGTGTTGGATATGAGCTCAGCTCCAGGAGGCAAGACCTCCTA
TATTGCTCAGCTGATGAGAAACACAGGCACGATCGTGGCTAATGACGCTAACGCTGAAAGATTGAAGAGTGTGGTGGG
AAACATCCACCGTCTGGGCGTCACCAACACTGTGGTCTGCAACTACGATGGCAGGCAGTTCCCAAAGGTAATGGGTGG
GTTTGATAGAGTACTGCTTGATGCTCCATGCTCAGGCACAGGAGTCATCGCTAAAGATCCAGCTGTGAAGACCAGCAA
GGACGAGGCAGACATCCAGCGCTCTGCTCACCTGCAGAAAGAGCTGATTCTATCTGCCATAGACTCTGTCAATGCTGA
GTCACCTTCAGGAGGATATCTGGTCTACTGCACATGTTCAATAATGATGGAGGAGAATGAATGGGTGGTGGACTATGC
CTTAAAGAAAAGGAACGTCAAATTAGTTCCCACAGGACTTGACTTTGGCAAGGAAGGCTTCACCAGGTTCAAAGAACG

CAGGTTCCATCCTTCTCTGCGACTCACTCGGCGATTTTACCCTCATTCCCACAATATGGATGGGTTTTTTGTGGCCAA
ACTGAAGAAGTTCTCCAATGTAATTCCAACCGCACCAGCAGGGAAAGAAGAAGAGAATTCTGAAGCTCCTGAAGCTGT
GGAGGTCGTTGCAGAATCTCCTCAGGAGAAACCGTCCAAAAGTGACAAGACGAACAAGACGGTCCCCGGTAAGGCAGG
CAGCTCAAAGAAAAAGGCCAAACCAATGGAACAGCAGCCAAGGAAAAAGCAAACATCAAGTCAAAAGGCAAAAAGAAC
TTACCCACTGGACCAAAAAGGCAAAGATCGCCAAGATGGATGGAAAGACTGTGAAAGGGGCGGAGGCCAAGAAGCCCG
CAGTGAAGACGACTGAGGAGACTAAAGCATCAAAGGCTGGCAAAAAGGAGGGAGCAGATTTGAGAAAAAGCAGGCCAA
AAAGATAAAATCACCCATGAAGGCCAAGAACAGAATTGGAAAGAATAAATTCAGAAAGTTGAAGCACATGCTGCAACA
ACAAGACACAGAGTGACATTGTACACAGTTTAAAATGTACTAGGTAATGTAAGAGCTTTGTAGAACTTCATTACAGAC
CCAAATCCTCATCCCTTCAGTCGAAGTTGGTGTGCTGTGTTCTTTCAGAGCAACAATACTGTATGTGTCAAATATGAT
TGTAACACGGCCATGTACAGGTGGATTGGACTGGTCAATGATGTTCACATTTAAGTTTAGTCCTCCCTTTTGTATTTG
TATTACAGAATATTCATTGTGTTTGATGATTTAATTGCAGGTTGGGACAACTTTAAGAAATCTGTGAGACAATT

>Sequ42422SNP3
TAAACACAGACCTTATCCAGGGAAGTTCCTCTGCTGACCTCCTCAAATCACTCCACACTGGGAGTTTCACTGGCCAGC
GTTTGATCATCTCGCCTCTTGTTTTCTGGAGAAATGTTGGAAAAGATCATTTTCCTGCTTTGTTTGTTGCCTGCGTTG
GCAGAACAGCTCTGTGCTCCAGATGCTTCAGATGGATACCAAGTTCGACTCAGCATCAGGACTGCTCTGGGAGATCAA
GCTTACGTCTGGAATGAAAATGAAATGTTCCTTTTCCGAGCGACTCTGGCTTTTGCCATGAGGAATCACATGAGCGGG
GAGGAGTTTGGAGTGTCAAACATCATTGTGTGCAATGAGACTCCCAGAGTGTCCTTCTGGTTTGTGGTGACGTTTCCT
CTGAACTCTTCACGCCTTGTTGATAAAGAAGCTGTGGAGGAGGCCATCAGGAATTCCCGGGGTCGGATCAACAGTGCC
TTCCTGCTGACTGATAAAACCCTGGAGTTTATCGGCATCCCTCCCACCCTGGCAGCACCAGTCAACCCTGACACCCCT
CCATGGCTCATTGTGTTTGGGGTGGTCATGGGTCTCGTGGGTGCTGGCATCATTGTTCTGCTTGTGTCCTCTCTGGTG
AAGAAACGCAAAGGGGAGAAGCAAACCGGCGGCGATGACATCGAAGAAGAAACACGGGTGAAACCGGTGGAAAATGGC
GGCACAAATGAGGGAGTTTACAACATGTCATTCTCAGATGATGAGCGATTAACGCAGATGTAAAACAAAAAATCTTCC
ATGTAAACAATAAGATGGGGAAAAAGTTATTGATGCTGCTGTTGCTGCACTGTAAAATCGGATCTTTTTGAAGATTAT
AGATTTATAGTTTTGTGATTATAGATGTTTCTCATTTAAATGATTCAAATTTTTTTAAATCTATATATTTGTACTGC
GTCATACAAAGAAACAAGCACGTGCACAGTGTTTTCATTTTCCACATGAATGTAAACTTTTTGTGTCGATGGATCAAA
CATCTGTGCAGCAGTCTATCTGTGATCTGCTCTTTGGAAAGAAACTAACTCTTCTATACGGATCATATTTCACCATGT
ACCATATACTTATTTTTTTGGCAAACAGAATGAGATGGAAACAAGAAATGAAACTGGTCTCATTTAAGATTTTAAGAC
AGATGCTAATCATGTGTGTGTAGTGCTGGAATTTATGAAAAAAAACTGAAATCTTGATGTTTGTAAGACATTGATACA
CAGTATATATGTATATCTAGATGTATATCTATATTTTTCTAATTGGAGACAGTTGAAGGCGATTGACTAGTTGCTTAT
TATATTTAACCACATTTTTTGCTAAATGTACTTGATCCTTTTGCTTTACATTTGTTTGTCATCATCTGTCTTTTAGCA
GACTGGAGTGACAGTGAACCCCCCCCCTGAACTTAGCGATGGGTTTAGCTGCAGTATTTCAAGCAGTGCTCGAGCTCA
TTATCACCATGGTTGGCAAAAATGTTACTAGACACAATTTACATACTGTCATGACAGTCAGAGCAGAGCAGCATCCTT
TGTACGTCGTGTCCTCTGTGTTGTTTAAAATCGAGTCCCTGACGTGTGCTGTGGTGTGAAAGTGAAAGGCCTCTGTAC
TATTACGCTGCAGTGAAACATACAGCTTCACTTCTCACTTCTAGGCCACTGAACACGCTTAGAAACACGATGTCTTTC
ATTGTTCCAGATGAAACATGCTCACATGATCTGTGTTCAGCAACTAAAAGTTTGTCAGTTTCAAACTTGACTTTACTG
CCTCCGCTGGACACTTTGGGGAAAGGATAGGTTCATTATCTTTTCTTTTT

>Sequ42551SNP3
GGTACGAAGAGCACGCCCTAGTGACACAATGGAGAGAAGAGCCAATAGGAGACGATTGAACTTAGAGTAACACGTCAT
TCTAGTAAAGTGCAGAAGCCGGCTGATAGGGAGCGATCCCCTCAACGTCGTCTTTCTGTGCCCCTGAACACCCGTTGG
CTTATTACCTCCAAATGCTGTAACTTCTTTTGAATACAAAGTACTCTTGTGTAAAACTGAGGCGAACCTGACATAAAA
GCTTGGTGATGGCGACCTACCAAGAGTTCATCCAACAGAACGAAGACAGGGATGGGGTGAGGTTCAGCTGGAACCTGT
GGCCTTCCAGCCGTCTGGAAGCCACCAGGCTCGTGGTCCCCGTCTCTTGCCTCTTCACACCCATCAAGGAGAGGCCTG
ACCTGCCGCCGGTCCAATACGAACCAGTTCTGTGCAGCCGGGCCAACTGCAAGGCGGTGCTTAATCCACTATGTCAAG
TCGACTTCAGAGCAAAAATATGGGCATGCAACTTTTGCTTTCAGAGAAACCCATTCCCTCCCTCTTACGCAGGCATAT
CTGAAGTGAACCAGCCAGCTGAACTCATGCCACAGTTTTCTACTATTGAGTACATAGTACAGCGTGGACCTTCAGCTC
CTCTGATCTTCCTCTACGTGGTGGACACATGTTTGGAAGAGGAGGACCTTCAGGCTCTCAAGGAGTCCCTGCAGATGT
CCCTGAGCCTGCTGCCACCCAACGCCCTGGTGGGTCTAATCACATTCGGACGCATGGTTCAGGTTCACGAGCTCAGCT
GTGAGGGGATTGCCAAGAGCTACGTGTTCAGGGGCACTAAGGACCTGTCCTCCAAACAGATCCAGGAGATGCTGGGTT
TAATGAAACCAGCAGCATCCGCACAGCAAGGTCGCCCAATGGCCCCTCAGGATTCTGCAGCCTCCTGCAGGTTCCTTC
AGCCCGTGCACCGAGTCGATATGAATCTGACGGACTTGCTTGGTGAGCTTCAGAGAGACCCGTGGCCTGTCCCTCAGG
GCAAACGTCCGCTACGCTCCACTGGTGTGGCACTGTCTGTTGCTGTTGGTCTGCTGGAGGGTACATTCCCAAACACAG
GGGCTCGTGTGATGCTGTTCATCGGAGGGCCCCCCACCCAGGGCCCTGGTATGGTGGTGGGCGATGAGCTGAAAACCC
CCATCCGCTCCTGGCATGACATACAGAAGGACAACGCTCGCCACCTGAAGAAAGCCACTAAGTATTATGAAGCCTTGG
CCAACCGTTCAGCTGTAAACGGCCACAGTATTGACATCTACGCCTGTGCCCTGGACCAGACTGGACTGCTGGAGATGA
AGTGCTTATCTAATCTCACCGGGGGCCACATTGTGATGGGAGATTCCTTCAATACCTCCCTGTTCAAGCAAACCTTCC
AGAGAGTCTTTAGTAAAGACTACAATGGAGACTTCCGCATGGCCTTTGGAGGTGTCTTGGAAGTCAAGACATCAAGGG
AGCTAAAGGTTTGCGGGGCCATTGGACCGTGTGTTTCGCTCAACTCCAAGGGTTCCTGCGTTTCAGAGAATGAGATGG
GTATCGGTGGCACCACCCAGTGGAAAGTGTGCAGTCTCAACCCCTCCACCACTCTGGGCATGTTTTTTGAAGTGGTGA
ATCAGCACAATGCACCAGTCCCACAGGGTGGCCGAGGGGCAATCCAGTTTGTAACCCAGTACCAGCACTCCAACACAC
AGAGGAGGATACGGGTCACAACCATAGCCAGGAACTGGGCAGATGCGCAGTCCCAGATTCAGCACATCGAGTCGTCAT
TCGACCAAGAAGCGGCTGCCGTGCTCATGGCTCGTCTGGGAGTCTTCAGAGCCGAATCAGAGGAGGGACCGGATGTCC
TGCGTTGGCTCGACAGGCAGCTCATCCGCCTGTGTCAAAAGTTTGGCCAGTTCAATAAAGATGATCCTTCATCCTTCA
AACTGTCAGAGTCTCTGTCCCTGTACCCACAGTTTATGTTCCACCTGCGGCGGTCGCCCTTCCTGCAGGTGTTCAACA
ACAGCCCAGATGAGTCGTCCTATTACAGGCACCACTTCGTCAGGCAGGACCTCACCCAGTCCCTGATCATGGTCCAGC
CCATCCTCTACTCATATTCCTTCCATGGACCACCAGAGCCTGTGCTCCTGGACAGCAGCAGTATCCTGCCAGATCGAA
TCCTGCTGATGGACACCTTCTTCCAGCTGGTCATCTACCATGGAGAGACCATAGCCCAGTGGCGAAAGGCAGGCTACC
AGGAGATGGCAGAGTATGAGAACTTCAAACAGCTGCTGCAGGCTCCTTTGGACGACGCCCAGGAGATCCTTCAGACAC
GCTTCCCCATGCCACGCTACATCGACACAGAGCACGGAGGCTCACAGGCTCGCTTCCTCCTCTCCAAGGTCAACCCAT

CACAGACCCACAACAACCTCTACGCCTGGGGACAGGAGACAGGAGCCCCGATCCTTACTGATGACGTCAGCCTGCAGG
TCTTTATGGACCACTTGAAGAAACTGGCAGTTTCCAGCTCTGCATAGATGTCTCTATTTCACCACAACGAAAGGAGTG
ATATGACATTCCTGTTCTGACATTGGGATCCATTAACATTTCCCCTGTTTTTGTAAAGGAGCCAAACCCAGAGAGTCT
ACTGTATGTGCTTGCTTGATTTTTATACAGTGCTCTTTCATATGTGACATTTCCATGACGTAAAAGGAGCAGAAAAAG
GGAGAGAATATCACATTCCTAATAAGAAAACAAGTGTCACTTCCACTCGTGTTCTTTAACCTGTTATTTATAAGAAGT
GGCTCCAATACTCTGGAACCAGACTGTCCTCATGAAAGCAATATCATCTCTATTTCTACTATGTATTTGATATCACCT
CTGACTTGCAGATTGATTAGACTAATAATTTAATTTGTTTTCTTTTGTTTGTTTATCCTAAAATGTGTATCTACTGCA
TCAGACTTGGTTGAAGGTGCTGTTTAATAGGACACTTACACGTATTTGCTTTGTCTATAGCTAAAGATGACATGTCTA
GACTTTTTCTCGTAAATAAATTATAATAGTGGACCTCCTAATGCTGGTTGAGAAATGTTCACACACAGGGCTTATCGA
TCTTCACACTGATGCTTTGTTAATCACAGTTTGACAAAAAAATCCTTCATGGATTATAAGTTAAGTGTTTTCCAAGAA
AATTCCCTCTGAATAATGAGGATTAATGTTCTGAAGAATGGAAGTCAATCACATCAACTCAACTGAGACTGACACTGA
TTTCGATGACGAACATTGACATTTATTTTTAACCTAAACTTCACAGGGCGGTCTATAGTTTAGTCCACTGATCCCGTC
TTGTTCACCATCTGGTATTTTGCACCTGATGACTGCCCAAGTGTCTTTGTAATCAAACTTTAATATACTGTGCTACT

>Sequ42615SNP3
GAGACGACAGGGGATGAGGGGAGAGGCTTCAGGCTGCAGCCTTACCCAACTCAAATAACCGAGTGCTCCATCTTACCG
AGCGTAGGCGATCTTCGGCGGAGCTAGCCGGCTAACAGCTAACGCGTAGAGACGCCATTAGTTGGGTGAAAACCAGGC
GTTCGCATCAGGTTGAAAGGGGATTTATTCCACAGTTTAGACCCATTTGTCTCACATCTGTTGGTCGATATTCATAAA
CGTCGGATTCTCTGATGCTGTCGAGGTGTTTTTCTACTGATTTTTAGCTGTTTTTTAAAGTCGGAAGCCGATGTGAGC
TACGCAGGACTTCAGTTCCGACGGGGGTTAGCATTAGCAGGGCTAGCTTGACATCGCTTGTTTTGATAAACGCTGATA
AATTAGTTTAATATAACTTCCAGCCATGGCGGCCGGAGGGGCGGGCGGCAACAGCGGCAAGACCTACTCCTTCAAGGT
GGTGCTGCTGGGGGAAGGCTGCGTGGGGAAGACGTCGCTGGTGCTCCGATACTGCGAAAACAAATTCAACGACAAACA
CATCACAACTCTACAGGCGTCCTTCCTCACAAAGAAGCTCAACATCACGGGAAAGAGAGTGAACCTGGCCATATGGGA
CACAGCAGGTCAGGAGCGTTTCCACGCGTTAGGTCCCATCTACTACAGAGACTCCAATGGAGCCGTACTAGTGTACGA
CATCACAGACGAAGACTCCTTCCAGAAGGTGAAGAACTGGGTGAAGGAGTTGAGGAAAATGTTGGGCAACGAGATTTG
TTTATGTATAGTAGGTAATAAGATTGATTTGGACAAAGACAGACATGTTTCAGTGGAAGAGGCTGAGAGTTACGCGGA
GTCGGTGGGAGCCAAACACTACCACACATCAGCCAAGTTAAACAAAGGCATCGAGGAGCTCTTCCTGGATCTCTGTAA
AAGGATGATGGAGACGGCTCAGGCCGAGGAGAGGTTGAAGGGCAACGGAGCCAGCCAATCAGCTTCGAGTAGGCGGGG
CGTACAGATCGTCGACGACGAACCACAGGCCACACCTGCCGGAGGATGCTGCTCTTCTGGCTAACACACACACACACA
CACTTTTACACAAACACACACACACACACACACACACTCTCTCTGTCATACATGCTTGCAGACATTATACTCACACAC
ACATTTATCAGTGTCTCTGTCGTCCGGTTGGTCCGCTCTGAAATAATCTGCTGTGTTCATTCAGCAACGCCTCTGCCG
CTAAGGAGCATGGGAAATCATATTTAAATGACTTTGTTCAGTGTACCCTTTCAAAGTAAAAGTAACTGAAGTCCTCAT
GATTGAAATCAGTGCTTTTAGTTACAAAATTCAAAAAAAAATCACGTTTTTAGCACTTTATTTACTGTCATTTCTTAT
AAAAACAGACGATAACTCGACAGTAATCCGAAACAAAGCAGCGACTAATATCACAGAAACATTGAAGGCCTCTTCCCT
CCCTCCTCCAGCTGCCTCCGTGTGTTTCATACTAAAAATTATTAATAAAACAGTTAAAAATAAGTTTGAAGACACAAA
AAAGCGGCTCAAGCTTCAGCAGAGATTTGTTTAGTTTTTGTTTTTTGGGGGTGGAGGGGCTCTTTAATGGTTAAACAT
GCAATCCGCACACACAAAGACACACTGTACAAACCGCCATTAATCTTCATGTTGATCAGTTTGTGGTGTAACAGCGTC
GCCTGCTGGACGAAAGTGGAACTGCAGGGAGACGATGAAAGTTAAATCCCGGCCGCGGAGTTCTTCACAGGTCCGGAC
GACTGAACTCTCCTGCTTCTTTATTCATTCATCACATATTTACTGTTGCTGCAGTGAAATCCTCCAGCTCATGTTTTC
CTCATGTTGCATTTAGTGCCAAAATCTTTCCCCCCGAATTTCATGATTGAACACTGGTGTCTGATCAATACACATCCC
ATAAAAACCTTAAACATCCCCTACAGTCAAACATGGAGGTGCAGACGTTCGGACCTGTGAGGAACCAGCAGCAGCTAC
AAACAGACTCCAGGTCGATGCACAAACATCATCAGATAAAAGCTGCAGTCACTTGTTCTACAAACAAACTATAAATAA
AATCATTCTTCTCATTGTTGAAGCCAAATGTAAAAAAAAAAAAAAACGTGATTATTTAATAAATAAAAAACAACATTTC
TGCAACAACCAGAAAGAAAAACTTTAAACTCTAAAATGAGAAAAAATAAAAAATTTCACAGTTATAAATAAATTAAAA
AAGAAAAGAAAAAACAATGAATCAGTTTGAAATATTCAGCAAAATCAAGGTAATTAAATATAAGATTAATTTAGTTTG
TTGTTTAATTCATAAAATAGTTAAAAATATCCCAATATTTTGGTTTCTAAATGAAAAATATTGTTAAAATAAAAAAAA
TTGTAGATAAAAATAAAACATGAGCAGCGAATACAAACG

>Sequ42920SNP3
TGTAGACTTTGAGCAGAGTGGGCTGGTTCGGGGGAACGACGCCTCTTGGTCGGAACGCGGATGTTTTCTGCAGCTTAT
ATAGCAGAGCTGAGCGCACACATCCTGGGCGGAAAACCTGAGAGAGCAGCAGTTTCACCTTCTTCACATCCAACATCT
GTCTTCTGTCCGCACCAGGCCTTTAGCACTTATGGCGTACCACAAAGAGTTCGAGGCTGCAGGGAAGAAGCCCGGCCT
GCAGGTGTGGCGGGTGGAGAAATTGGATTTGAAACCTGTCCCCTCTCAACTCCATGGAAACTTCTTCACCGGAGACTC
TTACATAGTGCTTTACACCACCCCTGCTCCTTCTTACAACATCCACTCGTGGATTGGCAACGAAGCTTCCCAGGATGA
GAGGGGCGCTGCCGCCATCTTTATGACCCAGCTGGACGATTTCCTGCGAGGAGCCCCAAGACAGTTCTCTGAGTATCA
AAACCAAGAGTCAGTCACCTTTCAGGGCTACTTCAGGTCCGGCATCAAATACAAGAAAGGGGGAATAGCTTCAGGCTT
CCAGCATGTGGTGACCAACGCGTAAATGTCAAGCGCCTGCTGCACGTTAAAGGTCGCCGGATGATCAGAGCCACAGAG
GTGGACTTGTCCTGGACCAGCTTCAACAAAGGAGACTGCTTCATCATCGACTTGGGAAAGGACATCTACCACTGGTCT
GGCAGTGAAAGCAATCGCTTTGAGCGCCTGAAAACCACTGAGCTGGCCATAGATATCCGTGACAATGAGCGAAAAGGC
CGTGCTGAAGTACACATGATTGAAGAAGGCTCTGAGCCGGAAGATGTCATTAAAGTGCTTGGAGCTATGCCCAACCTC
CCACCAGGAAGCTCAGATGATGTATCTGTTGATAAGAAGAACAAGGGCCAGGCATCTCTCCATTTGATTTCTGATGCC
GCTGGCTCCATGAAGACAACTATGGTGGCTGATAAAACCCCATTCAAACAAGACATGCTCTCCAGCAGCGACTGCTAC
ATCTTGGACAACGGAGGAGACAATAAGATATTTGTCTGGAAAGGAAAGGATGCAAATGCTGATGAGCGCAGAGCAGCT
TTGACTGCTGCAAACAAGTTCATTAAAGACAAGAACTACCCCAAAAATACTCAGATCCAGGTGATGCCAGCAGGGGGT
GAGACCACCCTGTTTAAGCAGTTCTTCTTCAACTGGCTGGACAAGGATGAGACTACAGGCCCAAGCAAGGCCTACACC
ATCGGTCGCATCGCCCAGGTGGAGCAGATTCCCTTCGATTCCTCCACGCTCCACAGCAACAAGATCATGGCTGCCCAG
CATGGCATGGTGGACGATGGCTCCGGGAAAGTCCAGATTTGGCGTGTGGAAGGAGGTGACAAAGTACCTGTGGACCCA
TCAACTTATGGACAGTTCTTTGGAGGCGACTGTTACCTGGTGCTGTACTCCTACAACACAGGAGGCAGAGAGAAGCAT
ATCATCTACACCTGGCAGGGGCAGAAGTGCACTCAAGATGAGCTGACGGCTTCGGCCTTTCTCACCGTCAAGCTGGAC
GACTCCATGGGTGGAGTAGCTACACAGGTTCGTGTTACTCAGGGCCAAGAACCCGCTCATCTTGTGAGCCTGTTCAAG

GACAAACCTTTGGTCATCCACCTGGGTGGGACATGCCGCCATGGCGGTGAGAGCAAGCCTGCCACTACACGGCTCTTC
CATATCCGCCAGAGCTCCACCAAAGCCACACGGGCTGTAGAGGTGGAGCCCACTGCCTCCTCTCTGAACACTAATGAT
ATTTTCGTGCTGAAGTCACCCAAGTCCCTGATCCTGTGGATGGGGAAGGGAGCAAATCCAGGGGAGATGGCAGCAGCT
AAGCATGTTGCCCGCCTGCTTGGAGGAACTGCCACTGAGGTGGAGGAGACCAAGGAGCCGGCTGATTTCTGGGCTGCG
CTTGGTGGGAAGAAGGACTACCAGACCTCCAGGACCCTGCAGAAAACAGTTAGGCCTCCTCGACTGTTTGGCTGCTCC
AACAAGACAGGCAGGCTGATAGCAGAGGAGGTGCCAGGTGATTTCACACAGATGGATCTGGCAATCGATGATGTCATG
GTTTTGGACACCTGGGATCAGATCTTCGTTTGGGTCGGTGCGGAGGCCAATGAGACTGAGAAAAGTGGATCTCTCAAG
ATTGCCCAAGACTATGTGAATTCTGACCCCTCTGGCCGTCGTGGTACCCCCATCACCACCATTAAGCAGGGGGAGAGC
CATTCTCCTTCACCGGCTGGTTCCACGCCTGGGACCCCAAGATGTGGGACAAAGATGTTCTGCAGCGCATACAGGACC
GTATCAAGAAGCATTAGTAGAAAACCACAAAGCAGTAGCTTCAGCACAACATCAGGAAAGATTGAATCCTCATGGTGG
GTTTGGAATTTCAAAGTTCTGTGTGGGTGGACGGCAGCTTGGAGATACACGGAGCTTTAGAAGATTTGTCATCCCATT
TTGCAAAACAGGTGTAATTAGCATCTCAAAGGCTGTATTTGAAATTACACGGCAAAGTCTGAAATAACATTCACACTG
TCATATAAGAACACAGACAGGATCTTATTATTGCACCCTCACAGAATTTTAAATATATTAAATTGAACCTTTGCTGGA
AAAGTTTCTGGGATTTTTCTTTGCAGCTTGTTTTCATTCTTTCATGTGCATTAAAACGATAAATGGTAAACTGTTTAC
GATCCAGTTGAAAAAAACCTGATGCATTATGTTTCATATGAGGTGCTGGAATTTTAGACAACACAAATTTCTTGTGAC
GAAATAATGTATATTTTACTAAATTGCACCTTTCACAGCCTCT

>Sequ42953SNP3
TCGCCCTGCTGTCGGTGCCTGTTGAGCAGCGAGCAGCACAGCAGCCACACGGAGCAACTGAGGAACAGACAGAGCGAC
AGAAACTCAACACGTAGTCCTCCACGCCCAGACATCACCAGCCTCCTCGCATCACCTCCCGGTTTCAGGTTCCCAACA
TGGCCGCCACCCCCGCGCAGATCTCTCCAGAGGAACTGGAGGAACTTAGAGAAGCTTTCGCAAAGATCGATGTGGACA
ACAATGGATTCATCAGCAAAGCCGAGCTGACCGAGCTCTTCAGAGCCGCTAACCTGGCGCTGCCCGGGTACAGGGTCC
GAGAGATCGTCCAGGAGCTGACCAAGACCAGCGACCAGCTCTCCTTCGACGAGTTCACTCAGATCGTCCACAGCCTGA
AGAGCAGCGAGGTGGCGAAGACCTTCAGGAAGGCCATCAACAAGAAGGAGGGAATCTGTAGCGTGGCAGGAACCTCAG
AGCAGTCCGGCACCCAGCACTCCTACTCAGAGGAGGAGAAAGTGGCCTTTGTGAACTGGATCAATAAAGCTCTGGAGA
AGGACGGCGACTGTAAACACGTTCTGCCGATGGATCCCAACAACAACGACCTGTTCACCGCCATGGGAGACGGGATCG
TCCTCTGTAAGATGATCAACCTGTCCGTCCCGACACCATCGACGAGAGAACCATCAACAAGAAGAAGCTCACACCTTT
CACCATCCAGGAGAACCTGAACCTGGCTCTGAACTCGGCGTCGGCCATCGGCTGCCACGTGGTGAACATCGGAGCTGA
GGACCTGAAGGAGGGCAGGCAGCACCTGGTCCTGGGTCTGCTGTGGCAGGTCATCAAGATCGGACTGTTTGCCGACAT
CGAGCTGAGCAGGAACGAAGCTCTGATCGCTCTGCTGCGTGATGGAGAGAGTCTGGAGGATCTGATGAAACTGTCCCC
TGAGGAGCTGCTGCTGCGTTGGGCCAACTATCACCTGGAGCAGGCCGGCTGCGGCAAGATCAACAACTTCAGCTCCGA
CATCAAGGACTCGAGGGCGTACTACAACCTCCTGGACCAGGTGGCACCTAAAGGAGACGAGGAGGGAATCCCCCCCGA
TCGCCATCGACACGTCAGGACTCAGGGAGAAAGAAGACCTGAAGCGCGCTGAGTGCATGCTGGACCAGGCCGACCGGC
TCGGCTGCAGACAGTTCGTCATGCCGGCGGACGTCGTCCGGGGAAACCCGAAGCTCAACTTGGCTTTTGTTGCCAATC
TGTTCAACAAGTACCCGGCTCTGAAGAAACCAGAGAACCAGGACATCGACTGGAGCTCCATCGAAGGTGAAACCAGGG
AGGAGCGAACCTTCAGGAACTGGATGAACTCACTGGGGGTCAACCCTCGAGTCAACCACCTCTACGTAGACATTGATG
ACGCCCTGGTGATCTTCCAGTTGTACGAGAAGATCAAGGTTCCAGTGGACTGGGACCGAGTCAACAAGCCTCCCTACT
CCAAACTGGGCAGCAACATGAAGAAGCTGGAGAACTGTAACTATGCAGTGGAGCTGGGGAAGAAGGAGGCCAAGTTCT
CCCTGGTGGGCATCGCGGGTCAGGACCTGAACGCAGGGAATCGAACCCTCACCCTTGCCCTGCTGTGGCAGCTCATGA
GACGGTACACCCTGAACATCCTGGAGGACCTGGGCGACGGTCAGAAGGTGACCGACGACACCATCGTGTCCTGGGTCA
ATGACGTGCTCACGCAGGCTGGAAAACCCACCATCTCCAGCTTCAAGGACGGGTCAATCGGCAGCAGCATGCCGGTCC
TGGACCTGATCGATGCCATCCAGCCCGGATCAATCAGATACGACCTGCTGAAGACAGAAGACCTGACTGACGAGGAGA
AACTCAACAACGCAAAGTACGCCATCTCCATGGCGAGGAAGATAGGCGCCCGGGTATACGCTCTGCCCGAGGACTTGG
TGGAGGTCAAACCCAAGATGGTGATGACGGTGTTCGCCTGCCTGATGGCGCGCGGCATGAAGAGAGCCTAAAGACCAA
GACGACACCCCGTCAGTAAACTGGACGAACATGAACCTGAAGACCGGCCTCAGGAAGTGAAGGGGGAGGGGTCAGTTG
TATTCTTGTGACTTAACATTTCAGAGGGAAAAAACTCTGTCACAGTCTCATAACCTGAAATATTGGACAGACAAGGTT
CAAATAATGTGAACTCATTTCAAAATAGTGGGGTGACAGAAACAAAGTAATTATATTATATGCAGAAATCCACTGCAG
ACTGTTATATTTCTCTGTTTTAAACTCAGGATCAGCTTCAGTTAAACATCATCATCATTAATAATAAAGTACATAATC
ACATCTGCAGGCTTCCCACTCTCTGCTGCACTGTTGTTGGTTTATCTGATGTCTGTATCATCATGAAGGACCTGCGTA
ATGTCCCTGTGTATTTCTGTGGCACACATTCAGCCAAGGTCTCACTTTTTCACCTGATTACAAACATTTCAAATAAGA
AACTTTATGACAAAATATGCAACGAATAAACGGACTT

>Sequ43398EST3
TTTATTGAATTGAGCAGTTTTTTTTTCTTGTTTTATTTTGTTTTTTGATTGTGTGTCCGTCACATTTGCCTTTTGTAT
TCCAAATATAGTGTATTTGAACCTCTGTCATTGGAGTAACATGTACAACTGTAAATCAAAAAGAACAAAAAAAGAAGC
TACGATTTGTTACGTGCAATACTTTGTAGAATACTTTTATTTTCATGCTGTTTTTCCAAAAGGTGGTACTGACCTTTG
AAGTAAGATGATGATGATGATTATAATGATGATGATAGCCTGTGAAATCTTGAAGAAGACAATTTGAATCTTCAGGGT
CCATTTGAGTGAAAGCGCTCTGCAGTGTGGAACTAAAACCAAAATGAACCACTATTCCATCAGAGATCGTGTGGTTTG
AAATGCACTGAGAATAAAGTGATGACGAAACTTTTGTCACATCAAACTGAACTTCTTAGCAAAACGCTGTTTTTTTAA
AATTTTATTTGTGTGACAGACTGCGAGTTGTAGTGAGTTTATGAGGGGAATGAAACTCAGTAACACTGCCACTCACCC
CCCTGCCGCCACCCCCACCCCCACCCCCACCCATCTTTCCGAAAAGCACGAAGCCACCAAAGCTTACCGTTAAAGCGA
TATCTGCGAAATCAAACCATTCTCGTGTTTCTTAATTTGCTTGTAATGGAACAAAAAAAACTTCTCGGCTGCTTTTGG
GGGGGGGGCTGCCCCTCAGTGACAGCAGCAGTAGATCCAATAAAATCCAGATTACATCATATCAAACCTTATTTATGC
AATTTCTACATTCTGCTGATCTGAACCCGACTCCACCGAACCCTGCGACCTTAGTGCTTTATCAGAGAAACCTGAACC
ACATCGACACAGGAACAGATTTTAAATTCACGCTGCCGACATTGTGTTCATGTGAAAACGTCACCACATCTGCAGGAA
TCTGTCTGTTTCTGTGACGTAGATGCAACATTAAACCGCACGGCTGACGTCACTCTGTGCGGTCGCAAAGGTAGAAAA
GTAGGGCTCCGTATCCCGGCAACATCCTTAACGCAAAGATCATCTTTGTTCTTTAAAATTTGAAAAAGATTTTGTCTT
AAATTTGAATTTTTTGTCATTTTTAACTTTGAGTCAAGATTCAGATAAAGAATACTTAAACGATGGCGATCTCGGCGC
TAAGACTTCTGGGAAACGGAGTCCTGCTCGTCCTTCCCCGTCCTCTCTCGTTCTGAGCATGGTCTCCAATGACAACGC
CTCCGTCTTTTCCTGCATTAAACCTGAATACCAGCCCTTCCTCTTTTCAGAATGTACATGGGAAAATGTTTTTAA

>Sequ43412SNP3
ATATACCGGTAAGTCATTAGCTCAAGTCGACACACCTGCCTTGAACTTAACGAAAGAAGCACGCGAGGTGCCCGTGAA
AGCGCTTTGTTGTAAATCTCGTCTGGAATGACTCTATTTAAAAGTGTGGATTGTAAACATTGGAAACATGATCGACTT
TAAATATGCGATGTGGCCTGATGAGAGGTGCATTTCATTGGTTATTTTCATCTTGATGTTGAGCTCCAAACCCACGGA
TGCTTTTCCACGCTCTGGACTTGACTTGAGAGGTAGCAGAACCCAGCGGGATGTCCCCTGCTCAGCCAGCCTGGAGTA
TCGGCACGGCAACATCTGCTGTCTAAACTGTCCAGCTGGTACACGTGTGGAATCACCCTGCACCGGAACACGAGAGAA
GGGGAGGTGTGAAGAATGCGAGCATGGGCACACATACACTGAGCACAGCAATGGGCTGAACATGTGTTTCAAGTGCAC
ACGGTGTCGCTCAGATCAGGAAATGGTAACGGAATGTAATCAAACTCATAATACTGAATGTCGGTGCAAATCAGGGAA
ATTCTGTGCTCCTGACCAGGCATGTGAGGTGTGCAAGAAATGTTCAAGTTGTGAGAAAGATGAAGAGATAGTGAGGAA
CTGCACCTCTACCATCAACACAGAGTGCAAGAAAATCCAGTCCAGCTCTGGCACTTCCCAAGCAAATGCAGCAATAGT
GGTGCCATTCATACTCTTGGCTAGTGGGCTCATTATCCTGGGAGCAGTTTGCTGTTGGAAGAAGAGAAACAAAGCAAC
AGACTCTCAGAGACCCAACGGCAAAGCTGGACAGCGTTATCCTGCTAACTTTCCCACTGAGGAAAGGAAGACCGGAGA
AACCAAAAGCCAAGCTGGCAACTGGTGAGACCTAAATCCTCAGCCGGCACGGAGGATGAACGTAAAGTGCTGTGCGCA
AGCCTCAACAGCTCAGCCAGTAACTCCCAGCACAGCCTAACCAGCCTGCCTCCCTCTGCCTTCCCCGTACCTCCCCCC
CAAGTCAGTGCTGTGGTCCCCCGGCAGCCCAACAGGAGGGAAGACTGGCAATTTCCCAAACTAGTTCCTCTGAAAGGT
GAAGATTCTCTGAGGAAATGCTTTGACTTCTTCGAAGAAGAATTGGAGGTCGACTACCACAAGAAATTCTTCCGTCAC
CTTGGGCTTAACGACAATGTGATCAAAAGCAAAGAGAACCTTCACTATGAAGACAGGGTCCATGAGTTGCTGAACATT
TGGTTGGAGAAAGTGGGCAGAGAAGCTAACTTAAATTACCTGCTGAGGGCATTAATTGATCTGAATCAGAGGCGAACA
GCTGAGAATATCAGGGACAGAGCTATTGTCAATGATCATTACATCTATGAGAATCAAATAGAGGTGAACTCCGTCTGA
AATGATGCATTTTTAATATAGTATTTACGTTGTGAGGTCCTTCCATCCGATACTGGTTGGAGTGGCATCGCACAGGGC
TTACGATCTGTAGTGCTGTTTCAGCCAGTCATAAATCTATTTTGCCAAAACGTTTATACTGCAGAGAAAGTGTTAGTA
ATGTCTCATTAATATCATGTTTGGTGTCAGGTCTAAGCTTGTGTGTTGCCCAGCAAGCATCACACGAGGCCGCCACCC
ATAGTGGTCTGCAGGCTGTCATGAGAGAAAGCATGAGTAATGATGTCTCATGAATCTTATGGCATGAAGTCGTCGCTC
TGTGAAAGCTTCTCCCTTGTCCCGTGCTGGCCAGTGGAGTAGCAATGCACAAAGCTTCCACCCATAGTGGTGGTATGT
TGAGTAGTCAATCTATTCAGCCAAAAAAAAAAAAACGGTGCACAAAGTGCGTATTTTACTGACACTGCCGGTTCTGCCG
ATTCAGTAAGGGCATGTTACTACTAACCCTGATGAGGAAATGCAGGATCTTGTGAAGTTAAACCAGGCTGAATTATCT
GCCTACTTAACCATACTTAGACTCCAAAATTACAACTTGTATTGAGTTGTTTTTTTTTAGTATGACTGAAAATGTTT
CAGCTCTACACGGAGGGTTGATAAAGTATTTAAATACAAAAAGTGTTTGCTGTCCTGTATACTACGTGTACAGACTGC
TGTTTACTTCCACACATCTCAAGACAAGTTGATTGTTTCTCATAGCGAGTGATGTTCAATAGACCTTATTGATGTCTT
AATCCTTGAAGATGTGCCTTATTTATGTAATGTCCTCAGGAATTAATGTCTATATGGTTGCACTGATTGAGCAACAGA
ACACGTGAAGCATCATCATAAATGTCTAGAGAGCACAAGTATGTTGAACTATTATGGTGGAGAGCAGTCAAGTTAATA
ATTATTTAATGACTATGGTCTCTGCCTGACTGTGTCTTTTATACTTGTTGATTGACAGTGTTAATGATCGTGGTTCTC
ATGGATCATGATATTTTAATCTTCTGTCCGCGTTGTTTCCGGTCAGTTGTTACACTTTTGTCATAACTGATCACAACT
GCAACATAAAAATGACAGTAGGTGCTCCTGGGTACCGCTGATGGGATAAATATGGCTTTGCCCAAAAGCACCTGAGAG
AAAGTGAATTTGATTCTTAATGAATTATGTGCCTGTACAATATGAGGGTTCTCTTGCATTGAGCTTTATGGCTCTCAG
TGGAAGTCGTGTATACATAGAGTCGGTGACTAATTAAGTTAAAAACCAACATAGGGTGTCTTAGTAAGGTGTTAACCA
CCAGAACAGCTTCAATGTCCCTTAATATCGATTCTACATATTTCTGTAACTGCTGGAGGATGATCACTATTCTTCCAA
AACATTCCCTCATTTGGTGTTTTGATGATGGTAGTGGAGTCCAAGTCTCTCATAGGTGTTCAGCCTGGGTTGAGATCT
GGTGACTGAAGGCCACAGCATATGATTTACGTCATTTTCAAACTGATTTTTTTTTTTTATCAGGTTTTTCCTTTGTCA
CCATCTCTCCTTCTCTGCTTTATACAAGTTTAATCACAATGTTACATAATGCTTTTCTGTTTTGTTTACATTTGTTGC
CACTTTTGAGTCGTGTGTGTGTGTATATATCTATCTATTTATCTATATATATAAATATATATATCAGCCTGTTGCGCT
GATGTTTTCTTTTATCTAATCTGACAACCTACTTGAGGTTTTTTCATAGTAATTGCAGCTTAATTACTGTAAAGGACC
T

>Sequ43602SNP3
AGCACCTTTATTTAACATTGCACTCACTTCTAGTCGCCGTGGATACCAACAGCAGCTGGACGACCACAGTGTAGAAAA
GTACATGCAAAATGTTCTGCAGGATGAAAACATGAAAACACCGTCCTCTCCAAACGTCCCGGTCTCTTTAAACGCCGC
TTCTGTTTCCTGCATTGTGATCAACAGAAACATCTTCCTGAGAGGATTCGTCTGACACAAAGACCTGGTCCAGGATCT
GCTGGTTTACTGAAACAAACTGGTCTTTGGGATCTTTTGGGATCCATTCTAAAAACTTGACTGCTCCATGCTGGAGAG
TGAGGAGGATTTAAAAAGCCTGCTTAGAAAATATTTATGTTCTGGAACACTGAGCACACAAACACTGCGTGTAAATTT
TGAATGTGAAACTTAGTTTGACAAAAAAACTACTTAAGGTACACTGTGTTTTTTTTTTAGATGTATACCAACACCTCT
TGATGACAACTGAGCCAACAACCTAATATTTAATGAAGACTGAGATTGTTTGGAAAGCTCTGGAAAATGTTTGTAAGT
GGATGTATTCAACACCCATTTAAGTAAATTTTTGACAAAATGTTTAATAAAAACATCGGCCATGTCATGATTTATCAT
TGCGAGTTATTAAACTGAATTTCAGACACTGAATCATCGTTTTGCATATGCACGTCTCAGGAGCGAAGTTCAAGTTCT
GCATTGATTCGTAACAAGAGCTACAGTCGTGTCCATGTCATTATGGATAATATACTTTGTTCCTTTGTGTAATGTGAA
GGCACCTATGTAGCACATAACTTTTACACACTTAGTCTAGAAGTTCACCAAAAGGCTAATAGCCTGAGGTAGTGAAGA
CTGATTTCAGACACATCTTCATTTCTTTAATTCTAGGATTTGTGTGTACTGTGTTCCAGAACCTTCAGAACCTTCAGA
ACCCTGCAGGCCCTGTGTTCTCGTATCTGGGTTCTAGCAGAGCTCACAGGTCCTGGCTGATCTAGAACATTGTGGACT
TGATGTCCTGGCTGAAGAAAGTTCCAAAGTTCAGGTCTCTGGACATCAGCTCCTCCTCTACGTTCTCCAGTGCCCACT
TGTGTTCCACGATCTCCAGCGCCTCCCACTCGCTCTTAAAAGCCTTGTTGGGGTCAGGGGGCATCGCCATGGCAGCAC
CTGTCATCTGGTCCTGCATGATCCGTGACTGGTCAGCAGCGTTGTCCTGTCCCAGTATGAGGCTGTACATGCTCCTCA
GTCCAAACACGTTGAGGAAGTACCAGGACGCTGAGCTCACCCAGGAAGCATCCAGTGACAGCAGGTCGATCCCTCGCT
GTAACATCGGCTTGAACCTCAGAGTCAGAGGGAACGGCACCTTGGTTATGACGAATCCAGAGAAAGCCCAGTTTATCC
ATCCACCAATCACAATCATGGGCAGGACGTTGGTCAGGTTTCCCTTCATCATGTCCGTCAACATGCTGGTGTCTGTCA
TGGGGTTCTTGGGGACGACCTTCCTCTTGACCTTCTTGAAGAAGCCAGTTTCTGCGTTGTTGAAATAATGTTTCCTCA
TGGCGAAAGACTGTCGGGGAATATACTTTCCATTCTCTCTGAGGATGCGGCTGCGCAGGAGCACCTGGCTGTCAGAGA
CCTGCTCCAGGTCGACCTTTTTGTCGCTGTGGAGCAGCTGGGTGACATAGTGACGGATGATGCCGACAAAGAAGGTGA
TGAAAACAATGGGCAGGACCACCCACAGACGAATACTGGAGTCCAACAAGAGCTCTGGACCGGCCATCGCTCTCTGTT
CAACCAGCCAATCGAAACAGACGAGGAGACGCTCACATTTATGAACTTTGAGAATCAGCAGCGCGACAAACACGTTCC

GCAGTTTAAAAAAGACGAAGAAGAAGCAGCTGTTTCGCGGTTCATCGTTAAGTTTACAGTCACGGAGGTTTTTTATCA
GGTCTGGACCTGCCGCCTGCTGATCCACAGACCAGTTTAGCTCCTGAGCTAACTAACTACACGACTTGGTTCGGTTCG
GTTCGGTTCGGTTCGGTTCAGACAGCAGCTGAACCAGGACCGACAGGCTCCGCCACATCTGTGATTATTTTACAGGGC
TAACGTCCGCCCAACAAGACTAGTTTAAGTGTCGTGAACTTCAGAAGACTCCCTGTGTTTGTGTGGGTGCAGCAGCAG
GATGGACATATCCAGCAGACTGGAGGTTTGGCAACAGCTGGCTGAGAACTGTGGTCTCATCACGGTCCAGCAGGGTCT
CCAGCAGGTCTGGACTCTACTGCTGCTCTGCCTGATCTGCAGACTCTGCTTTAGACTGGGAGGTGTGTCCACCCTGAA
GCATGTAGTGTCAGTGTTGGCTGGGATGTACAGCCTCTTCCTGTTCTTTGAGTTGCAGATGCTGTGGGTGCTGTTGTT
CAGTGCTCTCTGTTACCTCGTTCTGCTGCTCACTCAACACTCCAGTACCAGAGGCCTCTTCCTTTCGGCTGTTATCCT
CGTCTACCTCCTAGTTGGAGAGTTGCATTTAATTGACATGGTGACCTGGCATAAAATAGAGGCTCTCAGATGGTGGTG
GCAATGAAGGCCATCTCTCTGGCCTTTGACCTGGACAGAGCAGCAGTGGCAGGCCTGCCCT

>Sequ44862SNP3
GTCAGCTGAGGACAGACCGACGTACTCCAAAATGGCGAACAGCAGAAAGGACGTAGAAGAGGAGATTTACGACAAGGT
CGTGGACCTGACAGAATATGCCAAACGTCAGCGATGGTGGAACCGTCTTTTCGGAACCAACTCCGGGCCAGTAGCAGA
GAAATACTCCGTGGCCACACAGATAGCCATAGGAGGAGCGAGCGGATGGTGTGCAGGATATCTCTTCCAGAAGGTTGG
GAAAGTTGCTGCTACAGCTGTAGGGGGCGGTCTTCTGCTGTTGCAGATAGCTAACAATAGTGGCTACATCCAAGTGGA
CTGGAAGAGAGTAGAGAAGGATGTCAACAAAGCGAAGAAGCAGTTAAAGAAGAGCACCAATCAAGCAGGCCCAGAGCT
AAACACATTTGTTGAGAAGTCCACAGAGTTTGTGAAGAAAAACATTGTTGTCACAAGTGGTTTCATTGGAGGATTCCT
GCTCGGCCTGGCGTCTTAGGGCAGAAGCGTCTCTTTTTCTTCGATGTCATGATGAGCTGTAGGTAAAAAGTAAATCAT
TGCTGGATGGTTTTAAGTATTTTGACAGCAAGAAACCTATTTCAGATTAGCCTGTGGCTATGTGTCTTTACATAAGCT
GCACATCTACTACTTCAATACTGTACCTCCATGTGACAAGTAAATACGAATGTGGGACATCTGGTTAGACCTGAATGG
CCTTCGTAAACCACTCCACGTTCCAGGCCAAATAATGACACATGATGCAAAATTAATTAAGTTTATCAGAATGTTGTA
ATTTTTGTGTATTTATTTATACCACACCGACATTGTACTAGAGGGATAAATTATGCACTGTTGTGCACAATGGAGCCA
AACACAGTGAAGCCAAGTGCAAGTAATAATGATAATACAAATTCACCTTCACACGGCAGATGGTGCCCATAGGAATCT
ATCAATACCAGTGTTGTGTTTTCAACTCCAGACTTCATAAGCAATCATGTTCAGGATTAAAACTGGAATTTCATTCTG
AAGTGCTGATGGATATGAGATTGTTATGCCTTTTTAGAGGTGCTCCATGTTTGATGGGTTATGTTAGCCTCCCCTTCG
CAGTGA

>Sequ45876SNP3
ACCGACTCGCTCTCTTAGTGAAGTGCTCATGGAGTCCACAGAGAGCAGGTACGCTCACCTGCTGCAGCCCATCCGAGA
ACTCACCAAGAACTGGGAGATCGATGTGGCTTCAGAGTTAAACGACTATTTAGAAGAGTTGGATGAGATGTGCATCAC
GTTTGATGGAGGAGAAATTAGACTGAACTTTGCAGAAGCAGCGCTGCTGATCCAGGGCTCAGCCTGCATCTACAGCAA
GAAGGTGGAGCTGCTGCACAGTCTCGTTTACCAAACTCTGGAGTACATCAATGACAGAAACAAGAAACGTAACAAACA
GGCGGCAGAGTCTCAGGAGGGCGACGCAGACGCAGCAGCGAGCGCCCACGACGCTGAGGATGTGGCCGTGTTCACTTC
CCTGGACATCAACGTCTCAGAGAACTCACAGAGAGGTGACTCCAACACGACTGTGAATGTGGCTCCTCTTCCTCCCGA
GTCTCTGATTCCTCCTGAAACCCACGAGAAGCACAAACTTCCTCTCATCAGCTTGAAAGGTGAGGTGCTGTGCAGTCA
GAAGGACTTCAGGATCAACATGTTCCTCCCAGGAGACGAGGATCTGATCCTCCTCACGCTCAGATCAGGGTCCAGGTT
TCTACAGGACAGTGAGTCTCTGCAGCAGCAGGAAGGTGCTGTGGCTCGGGTCACAGGTGAGGCTGCAGCGGGTGCGGC
TGATGTGGGAGACGATGGAGGTGACGCTGCAGACAACTTCCTGCCGTTAGACGACAACAACATGGAGCTGGACCAGGA
GCCAGAGGAGCACATCGACAGGCAGCAGGCTCCAGGTGAAGGCCGGATGATTCGAGAGAGACGACAGGTGGAAACTGA
GGAGGAGGCGCCGCCTGCTGTGAACCTGTGGATGTTTCATGACCCGTACTCTGTCGTCGGGGAGGACAAACCGTTTAA
ATCAGGGAAATGTTACAAGGTTCCTGATGGTCTGGATGACGGAGGGAAGAGGAAAAGGAAACGACCGTCGTCACTTCA
GGACTTCAGGAGCTGGTTCAGAGGAACCTACGATCCTCCTGAACACAAGCTGAAGAATGGACCCACATTTACAGACCT
GAACTACATTTATCTGAGCACGATGAAAGACAAACTGAAGACCAGGAAGAGGATCTTCAGGAAAGCAGGCGTGGTGGT
TTCTGATGACGAGCTGAGGAGAACCTTCCTGCAGCTGGAGGAGGGGCCACAGCAGCAGGGGGAGGAGCCTCTGGAAGG
ATTCAGACACCCCGACCTGCTGGGTGGAGACGACGTCAACTCAGACAACGAATATGAAGCGTTTCCTGATGACGTTCC
AGCTGAGTTTGGACCAGACGTCATATCACCAGAAGCTCCGAGAGACGAGCTGAGTTATGAAGATCTGGTGAAGCTGCG
TGTGGAGCAGCTGGTGGTGAACTGTCAAGGTTACACTCAGGAAACTGCTCTGTCCCGACGGGTTAAAGACTGGGAGGA
CATGATCCGACCTCAGCTGGTTCTGCAGGAGGAGCGTCTGGCGTTCGACATCCACGACTACGGAGACCGGGTCGTCGG
AGCGTTGAACGGCGTCGGTCAGCGCAGACCGTTCTCCTCTATCGTTCACGGTCTGGACAACTTTGAAGCCTGTAAATA
TCTGCTGGCTTCACTGCAGCTGGCCAACGACTACACGGTGGAGATCGACAGTGCCGAGGGTCTGGAGGACAGTCTGGA
CTCCATGGGACTGACCCTGCTCAGCACTCACAGAGCCACAGACAGATTCAAGACCATGACGGCTTTGACCTGAAGCAG
CTGAGTTCACACTGATGCTCACACACATTGAGCTTCACTCTCTTCCTGTCTGCTCCTCTTCTCCTGGACTCCTCCTCT
CTGCATCATGTGTTGAATCTCATATGAGACTGTAAATGTCCCTCTCTCCCCTCTCTCCGGGAAAATGTGCTGACGTTG
TTTGCTTTTTTGAAATATGAATCTTTGTAATAAATAAAATGAGCATGTTGTAAAAAACTG

>Sequ54786SNP3
AGCCGCCATGTTTCATTCACCGCAGGTCATCGTCACAGTTCTGCTTCTTCCAGGACGAAGACGTCGCAGCAGGATGAA
GCTCCTTTCAGCCCGACTGTCGGAAATGTTTCTGCTGATTCTTTGTGTCTTAGTTTCTTGTATCGTGCAGCTGTCGTC
TCTGTTGTGACAGCTTCTTGTTCTCAGGTCTCTACTTGGAAAAGAGATTTTAACCCTGATGAAATAAAGATTCAATAA
AAGATAAACTCGGTGATAGATCTGATAATAAAAGCTAAGGACTGTGAACCAGGCAGTTTTTTACAGTGTGGAGGTGTT
TTCATCCTCTTCCCTCTCTGCATCATGACGTGGCTCTCACGGATCTGAAGTCCATCTCATCTCCTCCTGCTCAGACTC
TTGGCAGAGCATCACATCAGCGTTTTATTAAAAGTGAGGAGGTGAAATGAAACCTCCCGCACTCACCTTCACCTGCGT
TTGTCGCCGAGACACGACAAGAGGAGACGACTCAAGAAAAAGACTGTTCAGCATTTTAATATTTTCTAAAACAATTCC
ATTTCACTGTGGTTAAAGATCAGGAAGTGCTTGTGTGTCTTTGTATGTGATTAATACGAAGGTCCACAGCACAGGTCT
CCACGGCAACACTCCCAGCGTGTTCTGAGGTTGAAAATGAGGTGTGATCCTAATCCACGACCAGGGTGCTCCTCACTG
CGGGGAACAACTGGAAGAAACCCTTGAAGAGGGTGGTGCTCTGCAGGACGCTGGAGGTGCAGCACATCTCCCTGATGG
AGTGCATGGACAGCTGCGGAGCGCCGATGTCCACCACGGGGACGCCGAGGCGGGCGGCCAGGATGGGTCCGATGGTGG
TTCCACACGGGCTGTCGTTACGGACCATCACATCCTGCAGGGGCACGCCAACCTGGCTGGCGACCTCCCTGACGATGG
CGGCGGTGACGGCCGTGGTGGCGTAGCGCTGGTTGCTGTTGAACTTGATCACTGGCCCCTTGTGGAAAGCCGGCCGGT

GGTTTTCTTCGTGCTTCTCCTGGTAGTTGGGGTGGATGGCGTGCGCCATGTCAGCGCTGATCATGAAGGAGAGCGGCG
CCGCCTGCTGGAAGGCCGTGAGGTTGGAGGCGGAGGCGGAGAGGCGGGTGAGGATGAGCTCCGTCAGGTTGGACTGAG
CACCCTGAGCGCTCTCTGACCCCACCTCTTCGTTGTCGTACAGAGTGATCATGCGGACGTTGGGATCTTTGGTCAGAG
AGTCTCCGGAGCAGGACGCCATCAGGCCCTGCAGGGCGCAGTAGCAGCTGTGAAGGTTGTCCAGACGAGGAGAGTAGA
TGAACTCCTCGTACACACCTCCCAGAGCCGCGGGCTGAGTGTCGGTCAGGCACAGCTCAAAGTCCATCAGCGCCTCCG
GCTCCACGCCCAGCTCCGAACACAGCACCTTCACCAGCGCCGGGTGGTGCTTCTCCGCCGTGTTGGCGGCGGCGGAGG
CGTCTCCAGAGGACGCACAGCCCGTCTCCAGCTCCTCCTGGACGACCGTGGCGATGACGGGAACCAGGTGGTTCTCCT
TGTTGGGGCCGAAGGAGTCGTTGACGTCCCGCTGCAGGTGGATGGCCAGGTGAGGGATCCTGAGCAGAGGCCTGGGGA
CGTGAACCAGACGGTGGAGCAGCCGGCCGCCACTCTTGACCATGACACGGCCGGCGATGGTCAGGTCACGGTCGAACC
AGGTGTTCCAGATGCCGCCGCCGTAGCACTCCACCCCCACCTGCAGACAGCCCTGCTTCGTCCTCTTAGACCTCGGCT
TCACCCTGAGGCAGGGGCTGTCTGTGTGGGCGCCGATCATGGAGAAGCCATTTCCCGGCAGGTAGCCTCCGCCCACAG
CGAAGGCGATGAGGCTGGAGAAGTTCCTGGTCACAAAGTACTTGCTGGCCGGCTTGATGTCCCACTGCTCCGACTCCT
TCAGCTCGATGAATCCCGCCTCCAGCAGCCGCCGCTTGCATTCTTCCACCACGTGATACGGAGACACCCCTCTGTTGA
CAAACTGCAGGAACTCTTTGGCAGCGGACTGCACGGCCTCTTTGGTGCTCTTCATGATCAAAAACT

>Sequ54874SNP3
AAATGCACAATTTACTCTTGTTTGAGTCTGTTTGCCCTGGTGTTTAAGGAAATTCAACTTTTAAAGAAGTGGGACAGC
GCTGGGAGGCGGACAGACAGCGCTGGTTTTGGCCTTTCATGCAATTTGCTGACAATATGAAAAATATAGAACATTACC
AGCCACAGCCATGACCTTTAAAATGCTAACATTTAGTAGCATTAGCATGCAAAGTGGAAATTCAAAAATGGGACTGCA
AGTGTCCAATAAACAGCTATTTCCCTCCTTGTTGTGCAACTGAAGCAATATGACCCACTGACATTCACTAAAGCCATT
CTGCATACACAATCAAACTCCAACAATAAGAAACCGAAGGCAAAATGATGCTCCAACAATATATTCAGTGGCATAAAT
TTATATGGGTCAGGTGAATCCTATACACTGTATATCTGCTATTCCATATTATCTCTATGTACAAAATAAACAACTGGC
TCCTTGACCAACCCTGATAATTCCAGTACTTCCAGCTAAATGACATTTATCAAATGCACTGCAGACATTTGTTTAGAC
TTTTCCCCCCAAAATAAAAATTAAATTAAATTAAAACAATCACAATACAATTGTCATTAGGAGTTAATCCAACAACAG
TAAATGATAACAAAATTGTGTCATGCTGGGACGGGCTACAGTTTATATCAATTTACAGTAAAACTGAATAAAAATGTA
TGTTTCCAAATTATGAAAATAAATCAGCAGTCAGTTCTGTTAGTACTGAAGAAATTCATACAGCATGTTTTTCAGATA
ATCGTGCTGTGATTATAACCTGCACTTGTACTATCCTATTATTTGATAAGATTAACACAATGGCAATTTTTCTAGAAG
CTCATATCAAGTCGACTCTCACTTCATCTACGCCCTCTGAATTCATGCCACCACTCACTCCACTCTAACACACTCCTT
GCTCAACTCAGAGATACTGTGGCCAAACTTAGTAGATCCTGTTTCAGAGCCTCCACGCAGTCGGCCCCTCTGGCTAGT
CTGCGAAGGCAATCCCCAGCAAGGGAGGAGGATAAGAAGGGGCGAGGACAGTCCTGACAGCCCAGCAGCAGAGACAGG
CAGGCTCGACCCAGTGCCGGGTAACTGTAGTGCTGGGAAAACCAGTATACCTGCGGGAGGAGCACAGCCAGTAGCCCA
CC

>Sequ55413SNP3
AGTTCCTGATGATGTTAGCCCTTTTCACTCTTGACACATAAGGCTAAACTATCTGAAAAAAACCAAAAACAGGCCTCA
GTTGTTTTGCTTTAACTGCTCACCAGCTTTGAGTTAAACACTGTAACATGGCTCCATTCATGTTTAAATATTATCCTA
TTGTAATAATAAATAAAACAACCTCTCAAGATTCTTTAAAACTCCCAAAATCCCACAGAAATACATGGCAAATGATAA
ATACTGAGAACCCTGTTACATAAAACATGCAGCAGCATGAGTGAATGTAACAGTCTAAACATGGCTGGACTTGAAGTT
AGGAAAGGTTAAAGATGTCAGCAGCAAAAGAGGCTTAAAGCTATCATCTTCCCAGTCCATGTGCAAGGGGAGATCATC
AGGTTGCTCCGAGTAAAAAATGAGGGGGTGGGTGGTAGTGTTGGAGTGGGTGGAATGTGTCACTGTTGCTGGGATTTG
CGCCAGGCCATTAAGATGCAGCCGCCATGCGGATCCTCTCTGGCGGATACAGCAGAACGTTCTTAGCTGCTTTGATAG
TGTTCTTCATGAGCATCGCCACAGTCATGGGTCCCACACCTCCAGGCACTGGAGTGATGAAGCCTGCCTTCTGTCTCA
CGCCTTCAAAATCCACGTCTCCAACCAGTCTGTTCTTTCCACTGACCGGGTCCTGCACTCTGTTTATTCCAACATCAA
TCACTGCTGCGCCCTCTTTGATCATGTCCGCAGTGATGAGGTTTGGAATCCCTGCAGCAGCCACGATGATATCAGCGA
TTTTAGTGTGTTGGCAAAGTTGCTCCTTTGGAGTGTAACGGTGAGAAATGGTGACCGTGGCATCACCTCCGGGCCTCT
CGTGACGGCCGTCTGTATGCAGTAACATGGCGATGGGCATGCCCACATTCTTGGAGCGTCCTGCAACCACGACATTCT
TCCCAAGAGTAGGAATACCTGTGCGTTTAATCATTTCCCAGACTCCCCAGGGAGTGGCGGGGAGCATGGTGGACTGAT
CCAGGCACATGCGACCCACATTGACTACATGGAAACCGTCCACGTCCTTGGTAGGGGAAACTGCATTACAGACTGTGC
GCTCATCGATGTGGTCTGGCAGAGGCAGTTGGACCAGCAGGCCGTCCACACGATGGTCTGTGTTGAGTTTGTAGATCA
GGTCCAATAACTCCTCCTCGGTGATGTCTGAATGCTTGAGAATCGTCTCACTAGAGATTCCAACATCCGCTGCAGCGC
GTGTCTTGTTCAGGACGTAGGAGTGGCTGGCTGGGTTGTCTCCTACAAGAATCACACTCAGATGGGGTCTCCTGTGGC
CGGCTAAGACCCATTTCTCCACGTCGGCCCGGGCCTCCTCCCGAATCTGCCGTGCTAGTTTCTTTCCTGAGATGACCA
CCGCCTCCTGTCTCAGGGCGGACGTGTGCAGTTTACAGACTTGATGCTGGGAGTGTTGGCACAGTTTCGTGAGAGTCC
TGAGCGCTGCCATTCTTGCCGGCTGAGAGAAGCAGGGACACCACGGCCGATTACGATTACGAAATACTACCACACCAC
CACCACCACCACCCGGGCTGACAGAGAGATGTCTGTGAGAACTG

>Sequ55559SNP3
AGTCCTAGTGAATGTTTGTGTCAATTTGAAGAAGTTTCATCAAGGTGTTGCTGAGATATCGTGTCCACGAGTACGGGA
CGGACTGACGGACAACCTGAAAACAATATGCCACCGGCTCTGACTGTCGCCGGTGCAGAGGATAAAAAAAAGTTGTTT
TGTTAGATATAATGTTGATGTATCATTTTAATATTTGTGATATAGGATCATGAATGTGCAGTCATTCATAAAAACACC
AGGAAAAAACGAATACACATGATATGTCTCTTATGGTTTCTACAGGAAGAGATTTAACAGCACGACTGTCAGCTGAGT
CTTTACCCAAATTAGAAAAACAATTAAAGTATTTATTCACAATGCGAGTTCACAACTGCAGCTTTAACAGCTTTTAAT
ACCTGGAAGCTGGTTAAATGATTTTATAACAAACGTGAAGAGGATAAAAGTCACCATACAACCACAGAATGAAAAACA
ATCCACAACGATGTCTGACGTCAGCGACAGTCGTGTTAAACGTCCTCACTTGAAAAGCTTCTGAAACTTTGGTTTGAG
CATCTCCAGCTCTGTGTTGTTCTGCATCATCTGCGGCAGACTGCTCACGTTCATGATCGTCTTGTGAGCTTCGTCAAA
CAGCTTCTCTCCAAACTCCTTCGTCACTCGCGCTCTCCAGGTGAGGAGCTTCGGCCGCCCTTCGAACGCGTCCACACC
GGCTCCAACAGGCTGCATGATCTCAACTATAGCCACCAGATCAGCCAGAGAGATTTTGTCTCCGATGATGAACGGTTT
GTTCTGCAGGAATTTCTCCTCCAGCATGTCGAGTGACTGCTTCAGGTCCTCGATCGCAGCGTCCATCTTCTCCTTTGG
GACCTCAGAGCCCATGATGACAGGAAACAGAGTCCTGAGCAGGAAGACCTTTGACCCGTGAGCTCTGAGGTTCATGTG
CTGCCAGGACAGGTATTCATTAACGCGAGCTCGCTGCTGCAGGTCAGCTGGAAACCAGTGATCTGCCACTAACGTTGA

ATATTTCTGCACCATGTACTTCAGGATCGCAGTGCTTTCAGTCAGAATGAAGCTTCCATCCTTCATCACAGGGACTTT
CCTGATCATGCTGATTTCACCAAACTCCTCGCTGTGCTGCTGACCTGTAGAGAGGTCGACGCGCTTGAACTCAAAGGG
AATCCCGATCGCTTTGGCAAACAGGAAGACGGAGCGGCAGGGCTGGGAGAACAGGTCCAGATAGAGCTCCATTATGTC
AAGAGTGTGAATGCTGCAGATGCTTCGTTTCCACCGCGGTTTCCGCCTCTGTGTCCTCAGTCCTCTCCTGCCGCGCCT
CTTGTCCTCAGCCTGCGCAGGACTCCGCCTCTCCTCGCTGTAAAGGCTCATTGATTTCTAAACGGCGGCGACAGTTCA
CAGTGAGCAGGCCGTCGTGGTTGAGCGGTCTGACCTTTGGCCTCGGCCCTGTCCAACCTTTTGTAAACACAGCTGATA
TTATCACGTTTTTATCTTGCAACGGTTCCGGTCTGTATTTGTGCATGCCTGTATTCTTGGTGCAGCAGGTACTGAGGG
GCGGAGACGCCTGGTTTGGACTCACCTGGGCCCGGCGTTGTCCAACCCGTAAAAACCCCGCCTTCCGCTCTCTCTTAT
TTTCATATATTCATAGGTATTTGGTGAAATAAATGTATTTTATTCAGGGGGGGCTTGTCCAGTTTTTTGGCTTCACTA
CCTTGTAGTTTAATTGGCTGATTTTTGCTTGGTTTCGTTGCATTGCTTTGTTTCGCCTCACC

>Sequ56035SNP3
ACAATGGTGATTAAGAATGCACATCTCCATCTTTGTTTTCTGGCCTTTAATCATGTTTAATCGTGCTTATCACAGTGA
GCAGCTGCCAAGGAAAGATCATCACTTGCTGCAACCGTTGCTATATGATGCAATCGAAAGAAATGAAAGAAAATGCCA
AGGTAATCGCCTCAACAAATAAAAGTGCGTTTTTAAGATCATGTAGAGTAAGTACATACACTAAGTACATTACCCGTT
GACTTAATTATTCATTAAGCAGATATTATAGGAGTTTACTTAGACTACTTATGGGCCAAATCATTCTAAAAATGTAAG
AGTGGTTGCAGCAGAGGTGAATAGAGGTGTTACTGAAGGTCCTCCACTTGGGTGTCAACAACAGAGGACACCACCTTT
CCATCGACAATCTCCTCCACGATGGTCTTCACTCTCCTCTCGATGTGAGGTTTATGCTCCTCCACTTTTTCCACCACC
TTGCTGATGACAATAGCCTTTTTCTCAACTTGCTCTCCCTCCAGCAGCCTCCTGTACTCGGCGATCTCCAGTTCCAGC
CTCATCTTGATGTCCAGCAGCAGCTTGTAGTCAGCTTGCTGCTGCTCGATGGAGGCTATCAGCTGCTGCAGCTCTACC
TCCAGCATGTTAATGGTGATCTGCAGCTGGCTGAGCTGAGAGCCGTATCGACTCTTCACTTCTTCCAGATTCTGCTGC
AGGCACTGAAGCTCTGTGCGGACACTTTGCCGATTTATCTCCAAACTCTGGTAGGTCCTCTTCAGCTCTGACAGCTCA
GAGTGAGATGTCTTCACCTCTACTGTCCAAGAGGTGATGTCCGACTGGAGAGAAGTAACCTTAGACTGGAACCATTTG
TCGAGTTCCAGCTTGTTCTTCACAACCACAGCTTCATACTGCTCCCTGACTTCCTGCAGGACCTTGGTCAGATCAACA
GACTCGGCACTGTCCACATCCACATTCACAGCACCAGACTGCTGGACCCTCACCTGACTCATCTCCTCCTTGTGGGTG
CTCTTCATGTACACGAGCTCCTCTTTCAGGCTTTCTATCTGCAGCTCCAGGTCACTGATGTTAAGAGTCAAGCTGTCA
CGGACTCCTCGGAGACGAAGCACATCGGCCTCCACCGTCGTACGCATAGTCATCTCCATCTGATATTTCATCTTAAAG
TCATCAGATGCCAGTTGAGCGTTGTCCATCTGCAGAAGGACTCCTTGATTCTCTAGGCATCTCCTGGAGATCTTAGCT
TGGAGGTCAGAGATGGTGGCGAAGTAGGCGCTGTAGTCTCTGGAAGCAGTCGGAGATCTCTTCTCATAAAACTCTCTG
ATCTGCAGCTCCAGCTTCCTGTTGGCTGACTCCAGGCAGCGCACCTTCTCCAGGTAAGAGGCCAGGCGGTCGTTGAGA
TTCTGCATGGTCACCTTCTCGTTGGTGATCACTGAAGTGTCGTCGTAGGACCCTAAGCCAGACAACATGGTGGACTGA
GAGATGCGGGTCCCGAAACCACCCGCGCCCCCATACACGCTGGGCGTGCGGTTCTGGAAGGTGCCCGCGGTGGAGATG
CGTGTCATGCCGCCTCCGACGAGCTGCCTCCCTCCACTCAGGCTGAATGCCCCAGAGCTGGAGCTGCTGAACGAGCTG
TAGCTCCTGGACTAACCGCATGGTCTCA

>Sequ89765SNP2
GTTGAAGGGTCCACAAATTATTTGGAGGAAGTTGAAGGACAGATCTAATAGGCTTATGACTTTAGTAGTACCTTCTTA
AAATGGGGAGGGGGGACATACCAGGAAAGCTGGTAGCCTGGGTTCAATGCCTGATACAACTCTTGAGGTTTGCTGTTG
ATTTTAGCATCAAGCTGTGAGCTACAATCTGAAATGTTTCAGTCTTCGTTACTATACACATACAGATCAATACAGTGG
CCGCCTGTATGAAATGATCATGTTTAGTAACAGAACATTTTCAAGTATTATGTTCATGTCCAGGCAAAAGTGCTTCGC
TTGTTTCTGCCAAAGAGATTTTATCTGTTCAGCTACTTAAACCTTCTTGACTGTGTTCTCTGGTTGTTTCATCTGTGT
CTGCTAGTTACAAAAACTACAGTCTGAAAAAGCACACACACTAGAAGATACACCAAATATCACTTACAATGTGCCACA
CCACTTGGCTTCCTTATAAAGTAGTATGTCCTCTGTAATACAAATTGCTAATGACACTGGTATTACTAAAAGAGCAGG
GATGTAATGCTGTTGGAGCGTGTCTATAACTGGACTTACATACATCTATGGTGGCATTTGTGAATAAAGCCTCATTGT
TATTCAGATTTATCAGACCCTCAGACTGTACTATATACACAAAACAGATTTTACATTTTAATTACCCTTGTCAGTTTA
CCTCCTGTGTTGTTCTGTGCCATCTAAATAATAATCATGCTGCACTTCTGCGGCCTGAAGCTTGCCTTATTGCCTGTG
TTATGTGGTGACTGTTAATGTCAAATGCGTTTCTTTTTATGAGTCAGTGATGAGGAATGGGTCTGTAGGGCTGCTGCA
GGAGGGGAGCTGCAGAGCCAGG

>Sequsox9bEST
GTCAACAAAGAACCACCCAGATCAAGACAGAGCAGCTGAGCCCGAGTCACTACAGCGAGCAACAAGGCTCCCCACAGC
ACATCACCTATGGGTCCTTCAACCTGCAGCACTACAGCCCCTCCTCTTACCCCTCCATCACAAGAGCACAGTATGACT
ATTCAGACCACCAAGGTGGTGCCAACTCCTACTACAGCCACGCGGCTGGCCAAGGCTCCGGCCTATACTCCACCTTCA
GCTACATGAGCCCCAGCCAGAGGCCGATGTACACCCCGATCGCCGACACCACCGGGGTGCCCTCTGTGCCCCAGACCC
ACAGTCCGCAGCACTGGGACCAGCAGCCCATTTACACACAGCTGTCCA
